# Aromatization-Driven Borylene Generation from Borepin Precursors Enables Fused Borirane Synthesis and Selective Alkene Functionalization

**DOI:** 10.1021/jacs.6c11662

**Published:** 2026-08-25

**Authors:** Jay Prakash Maurya, Dmitrii Bushmin, Jonathan R. Church, Kartick Dey, Yael Diskin-Posner, Liat Avram, Linda J. W. Shimon, Samer Gnaim

**Affiliations:** † Department of Molecular Chemistry and Materials Science, 201485 Weizmann Institute of Science, Rehovot 7610001, Israel; ‡ Department of Chemical Research Support, 34976 Weizmann Institute of Science, Rehovot 7610001, Israel

## Abstract

Borylenes are among the most intriguing low-valent species in main group chemistry, yet their broader use in synthetic organic chemistry has remained limited by the lack of mild and modular methods for their generation. Here, we report a new class of borepin precursors that undergo aromatization-driven, redox-neutral borylene formation under synthetically practical conditions. This platform enables efficient interception of the resulting borylene intermediates by tethered alkenes through formal intramolecular [1 + 2] cycloaddition, providing direct access to previously unreported fused borirane frameworks. These strained borirane intermediates serve as valuable synthetic linchpins, enabling divergent olefin functionalization through C–B, C–O, and C–C bond-forming processes with notable regioselectivity. The synthetic utility of this platform is further highlighted by the selective functionalization of substrates containing multiple alkene units, guided by a native alcohol directing group, as well as by its application to the formal synthesis of natural products. To the best of our knowledge, this work represents the first application of borylene chemistry for the preparation of valuable building blocks relevant to natural product synthesis, establishing low-valent boron intermediates as practical tools for complex-molecule construction. Mechanistic studies combining DFT calculations, NMR analysis, and X-ray crystallography support a sequence involving Lewis-base coordination, borepin rearrangement, and concerted borylene extrusion driven by naphthalene formation. Overall, this work marks an important milestone in borylene chemistry by demonstrating that these low-valent boron species can be generated and harnessed as practical intermediates in synthetic organic chemistry, thereby opening the door to new reactivity patterns and broader applications in molecular construction.

## Graphical abstract

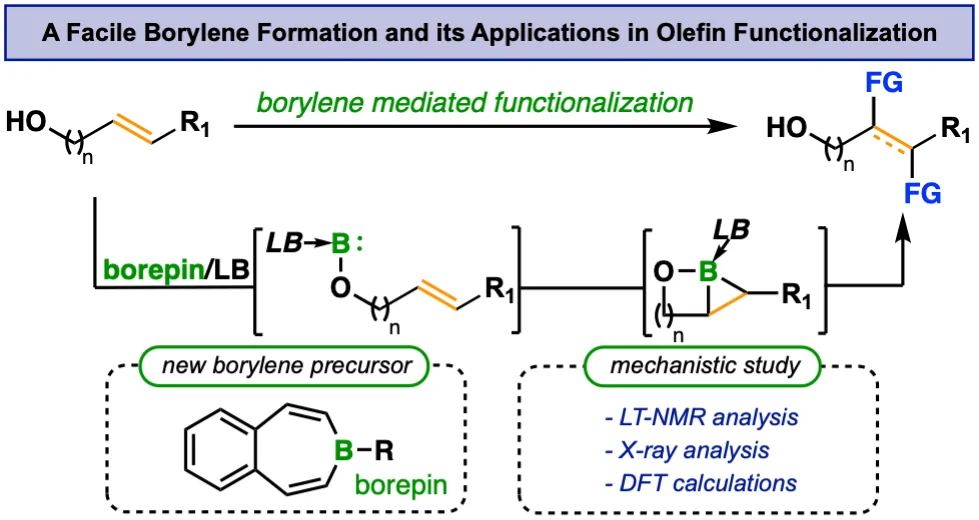

## Introduction

Low-valent main-group elements, most notably carbenes and nitrenes, have emerged as a powerful platform for developing synthetically valuable transformations that address long-standing challenges in molecular construction (Figure 1A).^1,2^ Continued advances in understanding carbene and nitrene structure, reactivity, and selectivity have significantly broadened the capabilities of synthetic chemistry, providing numerous methods for forming C–C and C–N bonds. This impact is reflected in a wide range of reactions, including (i) insertion into ubiquitous yet traditionally unreactive bonds such as C–H, C–C, C–O, and C–N bonds, (ii) rearrangement processes that streamline access to heterocyclic architectures, and (iii) cycloaddition reactions that enable the efficient synthesis of strained and structurally complex ring systems.^3^ Collectively, these developments have established carbenes and their analogues as indispensable intermediates in contemporary organic synthesis and as key drivers of innovation in reaction discovery.

**Figure 1:**
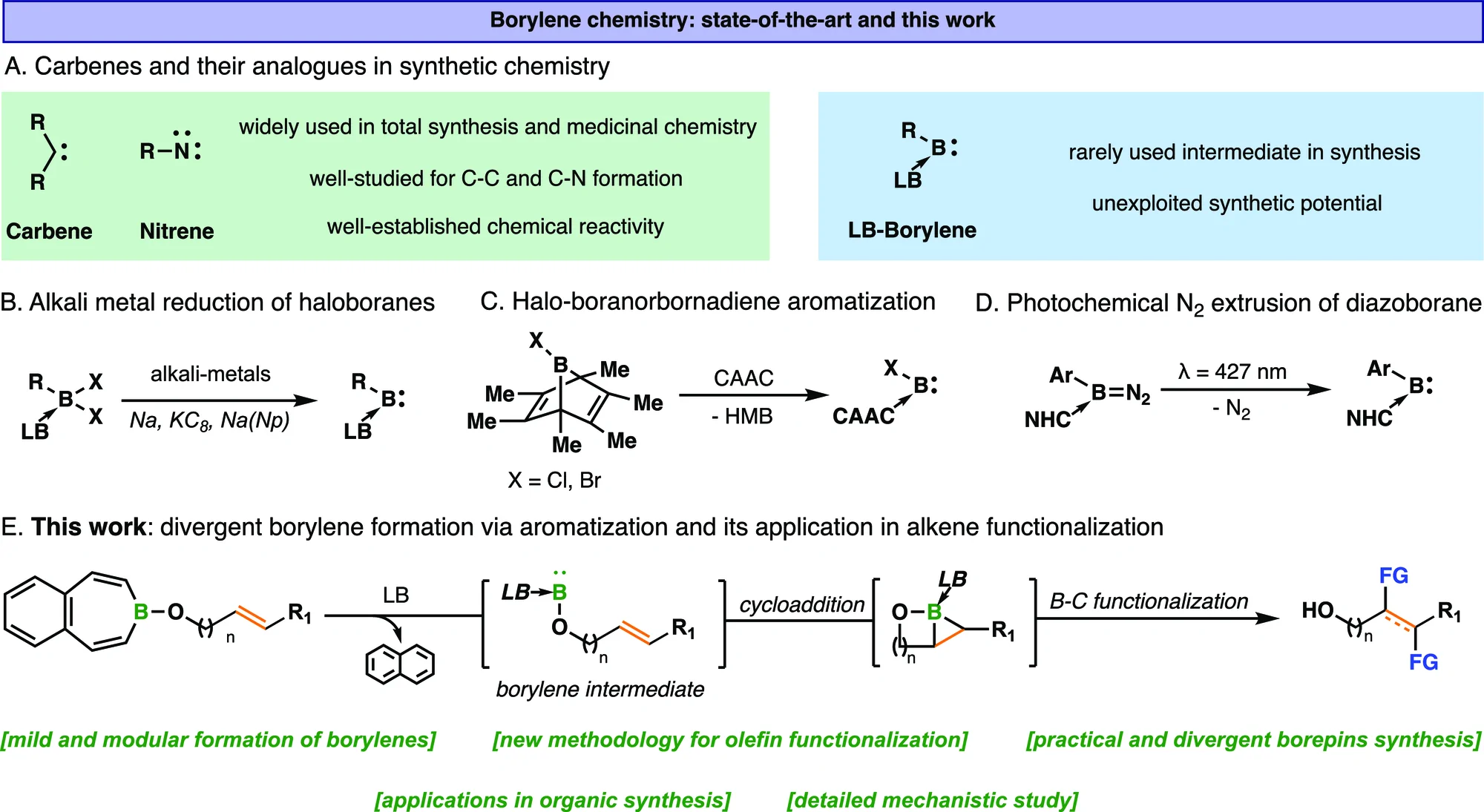
A) Carbenoids in organic chemistry. State-of-the-art of LB-borylene formation via B) alkali metal reduction, C) Bridged-borane aromatization, and D) photochemical nitrogen extrusion. E) This work: mild and modular LB-borylene formation via borepin precursors and its application in alkene functionalization. *n* = 2, 3, 4. HMB—hexamethylbenzene. CAAC—cyclic­(alkyl)­(amino)­carbene. FG—functional group.

Contrary to the essential role of carbenes and nitrenes in organic synthesis, the boron analog, borylene, has received limited attention in synthetic chemistry.^4^ While free borylene proved to be too elusive, the Lewis-base-stabilized borylene (LB-borylene) species were shown to be more accessible.^5^ In these species, coordination of the Lewis base to a vacant *p*-orbital of the boron center forms a three-center-six-electron (3c-6e) structure, making the boron center isoelectronic with carbenes. This development has spurred investigations into the reactivity of LB-borylene across diverse areas of chemistry. The impact of LB-borylene’s unique reactivity has enabled the construction of unprecedented boron-based inorganic materials. In 2007, Robinson showed that the alkali-metal reduction of carbene-stabilized boron tribromide can lead to the formation of a B=B double bond via boron dimerization, making it the first example of a neutral ligated diborane with a B=B double bond.^5c,6^ Beyond bond formation between boron centers, LB-borylenes have also been shown to engage π-systems in a manner reminiscent of transition metals; recently, Braunschweig and coworkers elegantly demonstrated reversible π-coordination and formal cycloaddition pathways with olefinic substrates.^7^ Furthermore, LB-borylene has demonstrated pronounced reactivity toward small-molecule activation. In a series of elegant reports, Braunschweig and coworkers have demonstrated that the LB-borylene intermediate facilitates challenging nitrogen activation, leading to its reduction to form a hydrazine adduct, its dimerization to a N_4_-based structure, or its fixation to form an ammonium salt.^8^ Similarly, Bertrand and coworkers demonstrated a LB-borylene for the mild activation of molecular hydrogen.^9^ Nevertheless, within the realm of synthetic organic chemistry, the exploration of LB-borylenes for innovative borylation and functionalization techniques is still in its nascent stage.

LB-borylenes are typically generated by reduction of a dihaloborane with an alkali metal or an alkali metal salt (Figure 1B).^5a,7,10^ From a synthetic perspective, however, this strategy presents a major limitation that has restricted the broader exploration of LB-borylenes in synthetic chemistry. Alkali-metal-based reductions are often quite harsh and can promote numerous undesired redox processes which narrows the reaction scope and reactivity study. Consequently, generating and studying the fundamental reactivity of (LB)-borylenes remains challenging. As a partial solution to this challenge, it was recently reported that aromatization of a bridged-borano-precursor enables the formation of a carbene-stabilized borylene.^11,12^ This approach was shown to be limited to halide substituents on boron, such as Br and Cl, and, apart from self-reaction, interception of the generated borylene was not reported (Figure 1C). Recently, Gilliard, Cummins, and their coworkers elegantly demonstrated that aryl-substituted diazoboranes can serve as precursors to carbene-stabilized borylenes upon thermo- or photochemical activation (Figure 1D).^13a^ In that case as well, the borylene was trapped only through self-reaction, where it underwent C–H insertion on the coordinated carbenic-Lewis base. Building on this work, the same groups reported an appealing synthetic approach for the C–H borylation of alkenes via [3 + 2] cycloaddition with diazoboranes followed by nitrogen extrusion, to furnish products corresponding to formal borylene insertion into a C–H bond.^13b^

With recent advances in borylene chemistry, it has become evident that several key challenges must be addressed before these species can be more broadly integrated into organic synthesis. These should include: 1) the mild generation of LB-borylenes, ideally under redox-neutral conditions; 2) the development of an inert and modular precursor that enables broad electronic and steric variation; and 3) the identification of reaction conditions that allow efficient interception of borylenes by synthetically useful functional groups to make new C–B bonds selectively.

Herein, we report a new class of borepin compounds that enables a mild, modular, and synthetically practical generation of borylenes (Figure 1E). Using this platform, we explored the cycloaddition of borylenes with olefins, providing direct access to previously unknown fused borirane structures. Importantly, these strained borirane intermediates serve as a versatile entry point for downstream olefin borylation and functionalization processes. In this way, the present strategy establishes a borylene-mediated platform for olefin functionalization through reactivity modes that remain largely unexplored in organic synthesis. Supported by extensive DFT calculations, NMR spectroscopy, X-ray diffraction analyses, and other experimental studies, we propose a detailed mechanistic picture for both borepin-to-borylene formation and subsequent cycloaddition with olefins to form boriranes.

## Results and Discussion

To probe the concept outlined in Figure 1E, we began by synthesizing benzoborepins bearing varied substituents. We envisioned that coordination of a Lewis base to the boron center of the borepin would initiate a cascade sequence involving naphthalene extrusion and borylene formation.^14^

Using a modified literature procedure, phenyl- and chloro-substituted borepins were prepared via a simple tin–boron exchange strategy.^15^ Borepin **3** was isolated as a white solid in 73% yield from the reaction of dibutylstannepin **1** with dichlorophenylborane. Similarly, borepin **4** was obtained in 85% yield from dimethylstannepin **2** and boron trichloride. Importantly, this transformation is operationally simple and can be carried out on a gram scale using the commercially available boron precursors, as illustrated in Figure 2A. Furthermore, borepin **3** exhibits notable stability under ambient atmospheric conditions, remaining unchanged for several weeks (Figures S6 and S7).

**Figure 2:**
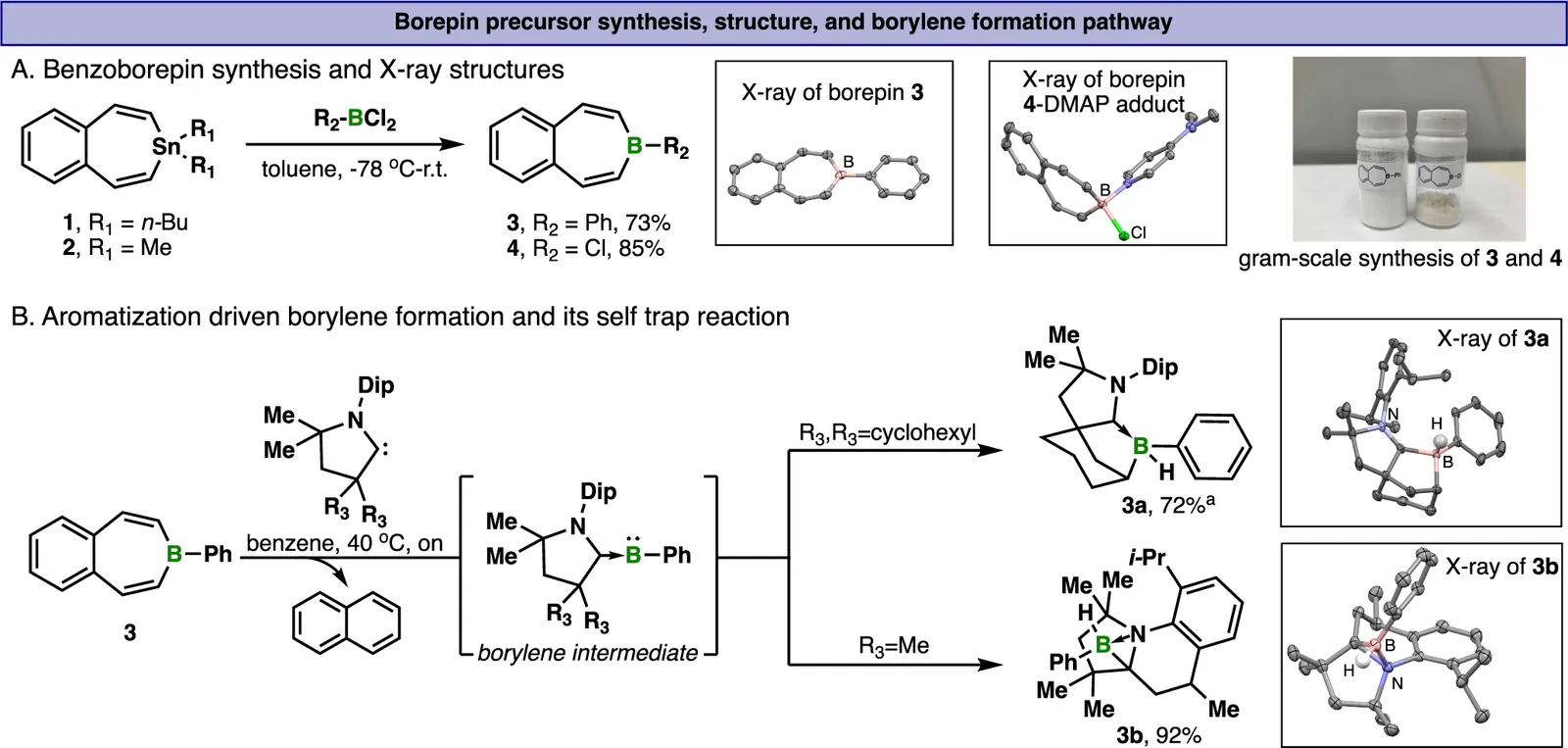
A) Synthesis and X-ray structures of benzoporepins. B) CAAC-borylene formation from borepin **3** via an aromatization-driven process. ^a^NMR yield. Dip—2,6-di-isopropylphenyl. On—overnight.

Single crystals of borepin **3** suitable for X-ray diffraction analysis were obtained by slow evaporation of a saturated toluene solution at room temperature. For borepin **4**, a single-crystal X-ray structure could not be obtained; however, its identity was confirmed by crystallographic analysis of the corresponding 4-dimethylaminopyridine (DMAP) adduct (Figure 2A). The nearly planar structure of **3** (the dihedral angle of the fused ring planes is 174°, Figure S10, see SI) is consistent with delocalization across the benzoborepin ring and supports its aromatic character, with the boron center incorporated into the π-conjugated framework. By contrast, in case of borepin **4**, coordination of DMAP at boron induces pyramidalization at the boron center, displacing it from the plane of the ring (the dihedral angle of the fused ring planes is 135°) and interrupting conjugation (see SI, Figure S11).

Next, we examined the stability and reactivity of borepin **3** in the presence of carbene-type Lewis bases. Notably, when CAAC-type Lewis bases were employed at room temperature (in benzene), the aromatization process was initiated, leading to the formation of naphthalene. Because this aromatization occurs under redox-neutral conditions, it provides initial evidence for the intermediacy of a borylene species.^12^ In the case of the cyclohexyl-substituted ^Cyh^CAAC, the reaction reached completion within 12 h and afforded product **3a** in 72% yield, arising from formal boron insertion into a C_sp3_–H bond of the cyclohexyl substituent accompanied by naphthalene (82% yield) as evidenced by ^1^H NMR spectroscopy (Figure 2B). The formation of this product supports the involvement of a borylene intermediate, as such species are well-known to undergo formal C–H oxidative addition in the absence of an external trapping reagent.^5a^ Notably, although several examples of intramolecular C–H bond activation involving CAAC ligands have been reported previously, activation of this specific C–H bond in the cyclohexyl-substituted CAAC ligand has not been reported to date.

We next examined the analogous reaction with the ^Me^CAAC as the Lewis base. As in the previous case, aromatization of the borepin framework occurred readily, releasing the naphthalene in 92% NMR yield (Figure 2B). In this system, the putative borylene intermediate underwent C–H insertion into a Dip substituent, followed by rearrangement to furnish the corresponding nitrogen-coordinated boron product, **3b**, in 92% yield. This product formation is consistent with pathways previously reported for related phenyl-substituted borylenes supported by ^Me^CAAC.^16^

These results demonstrate that aromatization of the benzoborepin framework provides a highly efficient route to borylene intermediates under exceptionally mild conditions (without reducing agent, 40 °C reaction temperature, and 12–18 h reaction time). It is particularly noteworthy that, in contrast, related bridged-borane systems bearing a phenyl substituent at boron have been reported to remain stable and not to undergo aromatization to generate the corresponding borylene species.^12^

To further explore borylene reactivity and its interception by organic functionalities, we examined its intramolecular cycloaddition with alkenes. This idea was motivated by the observation that borylenes can undergo intramolecular C–H bond activation (Figure 2B), prompting us to question whether they could similarly react with alkenes through a cycloaddition pathway to form strained borirane rings, a recently emerging class of unique compounds.^7,17^ In addition, olefin functionalization is a fundamentally important transformation in synthetic chemistry, making this mode of reactivity especially attractive to investigate. In this approach, alkene-containing alcohol substrates **5** (1 equiv) react rapidly with chloro-borepin **4** (1 equiv) in benzene to afford the corresponding alkoxy-borepins **6** in high isolated yields (81%–97%), typically within 10 min at room temperature (Figure 3A). Borepins **6a**–**h** were obtained by evaporation of the solvent from the crude mixture, followed by a wash with pentane. Similar to borepin **3**, borepin analogs **6a**–**h** exhibit an aromatic nature with characteristic ^1^H NMR signals (benzene-*d*_6_) of two doublets in the ranges of 7.78–7.75 ppm and 6.83–6.74 ppm. In addition, X-ray diffraction analysis reveals that the dihedral angle of the fused ring planes is 170° and 176° in the case of borepins **6a** and **6b** respectively which indicates π-electron delocalization across the borepin rings and is consistent with their aromatic nature (Figure 3C). However, in comparison with chloro-borepin **4**, alkoxy borepins **6** exhibit less aromatic character (for discussion, see “Notes” section in SI).

**Figure 3:**
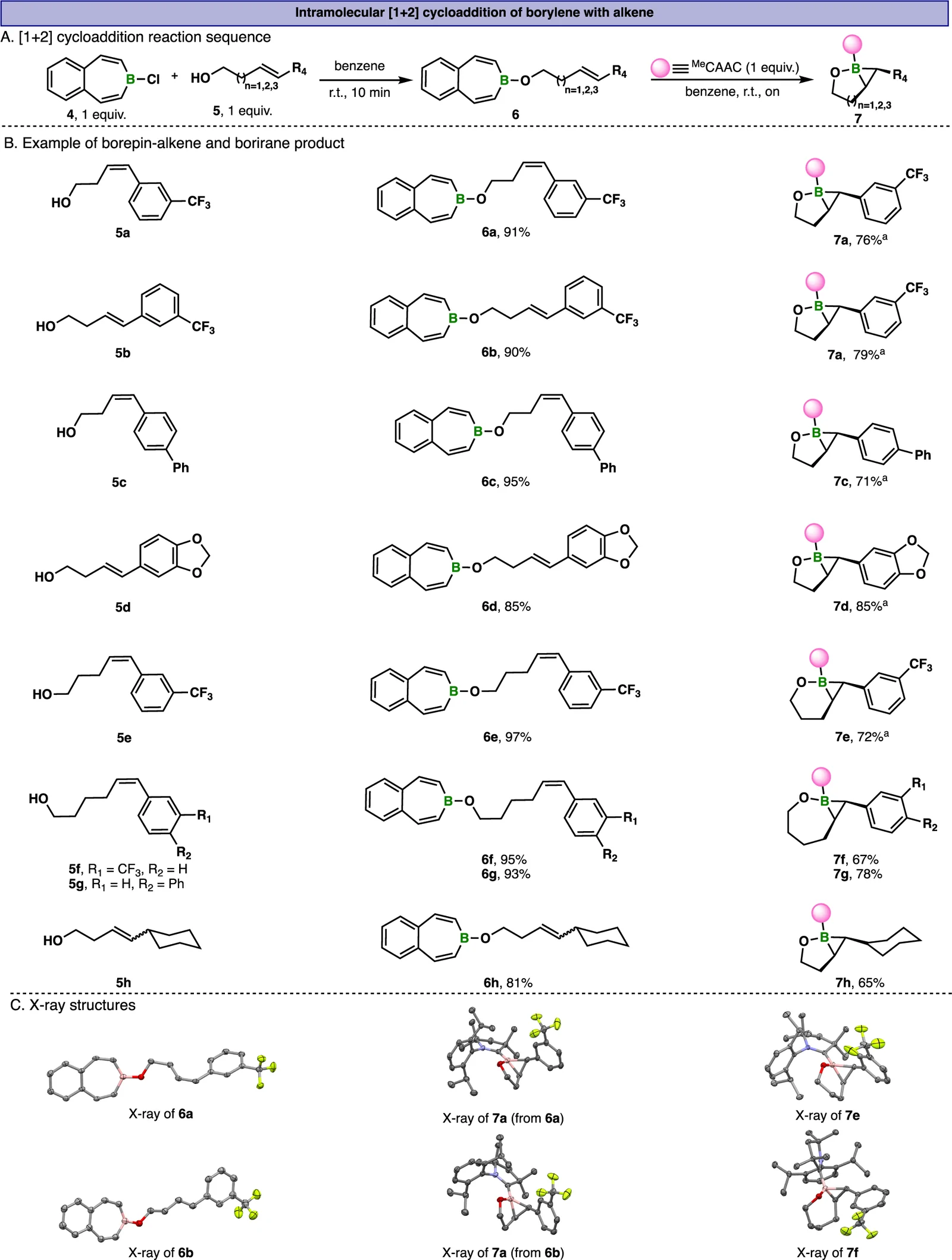
A) reaction conditions for borepin **6** and borirane **7** formation. B) Scope of the [1 + 2] cycloaddition reaction. C) X-ray structures of selected borepins and boriranes. ^a^Traces of the *anti*-isomer were detected in the NMR spectra (see SI, Notes).

Next, we turned our attention to evaluating the cycloaddition reactivity. Upon treatment of borepin **6a**, bearing an internal *cis*-alkene, with ^Me^CAAC, it underwent aromatization to release naphthalene and generate the corresponding borylene intermediate, which is then trapped intramolecularly by the tethered alkene through a formal [1 + 2] cycloaddition, furnishing the corresponding borirane product **7a** in 76% isolated yield. The structure of **7a** was confirmed by ^1^H-, ^13^C-, and ^11^B NMR spectroscopy. During the course of the reaction, the olefinic proton signals disappeared and were replaced by new resonances at 2.28 ppm (doublet of doublet) and 2.50 ppm (doublet), which were assigned to the borirane protons. In addition, the two CH_2_ protons of the fused five-membered boracycle appear as an AB system at 3.50, 2.83, and 2.12–1.97 ppm, supporting the fused cyclic structure. The ^11^B NMR spectrum of **7a** showed a sharp singlet at −6.4 ppm, consistent with borirane formation. The molecular structure of **7a** was further confirmed by X-ray crystallographic analysis, which revealed that the aryl substituent and the fused five-membered boracycle are arranged *syn* to one another (Figure 3C). To the best of our knowledge, this type of fused borirane scaffold has not been reported previously.

To further probe the stereochemical course of the reaction, we also examined the corresponding *trans* isomer **6b**. Surprisingly, this substrate likewise underwent efficient cycloaddition to afford the same borirane **7a** predominantly in 79% isolated yield. Notably, despite starting from the *trans*-alkene, the major product was again obtained with *syn* stereochemistry across the borirane C–C bond. NMR analysis of the products obtained from both **6a** and **6b** additionally revealed minor signals (approximately 6–8%) that were tentatively assigned to the *anti* diastereomer of **7a** (for discussion, see “Notes” section in SI). A more detailed discussion of the observed stereochemistry is presented in the mechanistic section, *vide infra*.

The scope of the transformation revealed that both electronically neutral, deficient, and electron-rich arene substituents are tolerated under the reaction conditions, as illustrated by boriranes **7a**, **7c**, and **7d**. In addition, the cycloaddition could be extended to form larger fused-ring systems, providing six-membered and seven-membered borirane-fused products in decent yields (67%–78%; products **7e**, **7f**, and **7g**). Finally, the method was also applicable for unactivated alkenes, with substrate **6h** delivering the corresponding borirane **7h** efficiently.

Single-crystal X-ray diffraction studies of fused borirane systems with different ring sizes provide important insights into the bonding behavior of the fused borirane unit. A clear trend is observed in the C–C bond length within the borirane ring, which increases with increasing size of the fused ring. In the five membered fused borirane **7a**, the C–C bond length is 1.50(3) Å, whereas in the six- and seven-membered fused systems (**7e** and **7f**), the bond lengths slightly increase to 1.51(2) Å and 1.52(2) Å, respectively. In addition, the B–C bond lengths within the borirane ring exhibit distinct variations with ring size. In the five-membered fused system **7a**, the external B–C bond length is 1.63(3) Å, while the internal B–C bond is slightly longer at 1.64(3) Å. In the six-membered fused borirane **7e**, both B–C bonds are nearly identical, with bond lengths of approximately 1.63(2) Å. However, in the seven-membered fused system **7f**, a significant difference is observed: the external B–C bond is longer at 1.68(2) Å and the internal B–C bond is shorter at 1.58(2) Å when compared with **7a** and **7e**.

Following our in-depth investigation of borylene formation and its cycloaddition reactivity with various alkene classes, we sought to expand this chemistry beyond cycloaddition and explore its potential toward broader alkene functionalization. This idea is inspired by the rich field of transition-metal-mediated alkene functionalization, in which native functional groups embedded in organic molecules often serve as directing groups to control reactivity and selectivity.^18^ In a similar spirit, we envisioned that borylene could be harnessed to activate alkene motifs toward downstream functionalization, with the alcohol functionality acting as a native directing group to guide this transformation. Through this strategy, we aimed to establish a new mode of alkene functionalization that merges the unique reactivity of borylenes with the substrate-controlled selectivity commonly exploited in modern organic synthesis (Figure 4).

**Figure 4:**
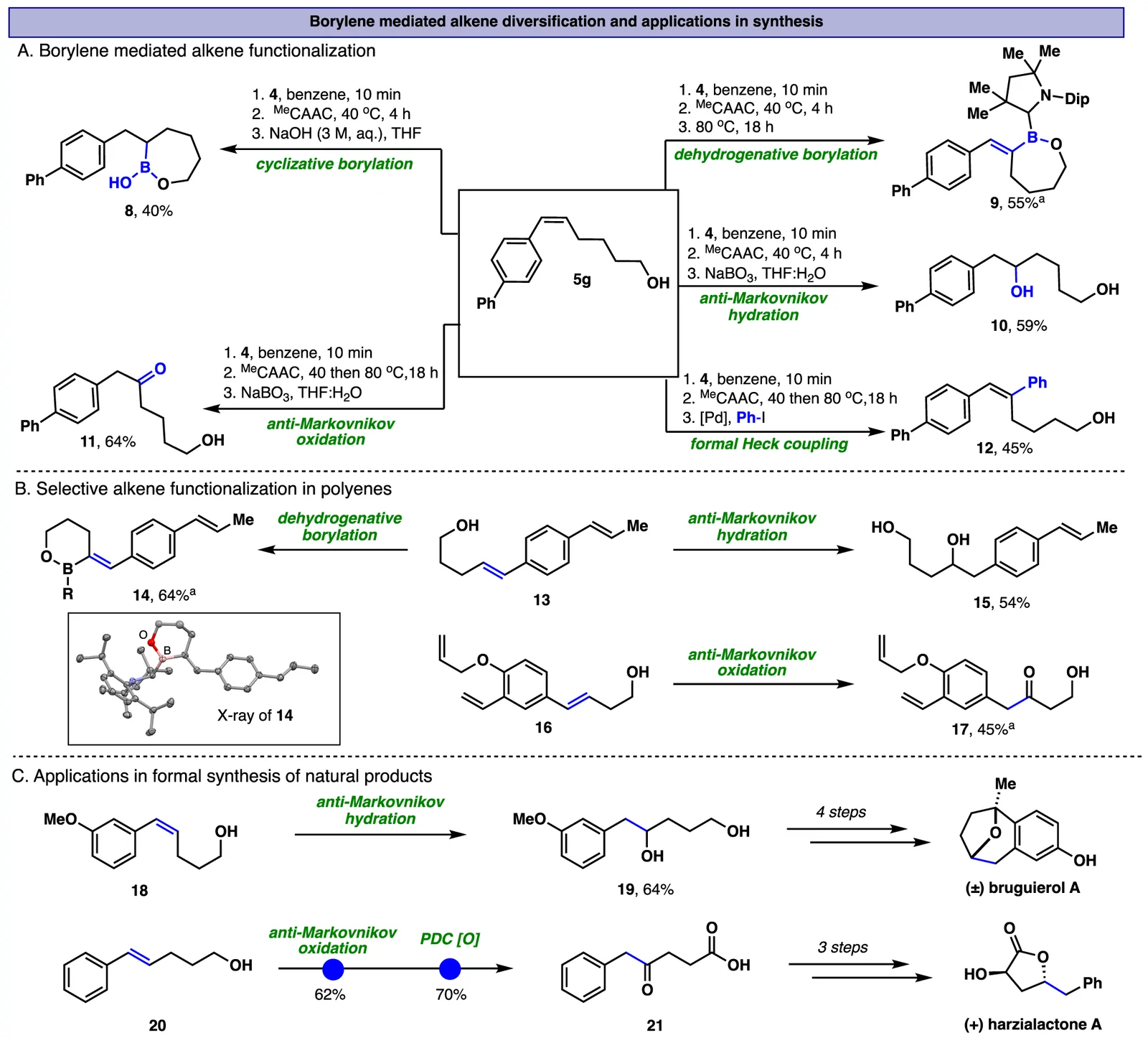
A) One-pot protocols for the modular alkene functionalization mediated by borylene. B) Selectivity study of the alcohol-directed alkene functionalization. R = ^H^CAAC. C) Applications in the formal synthesis of (±)-bruguierol A and (+)-harzialactone A. ^a^NMR yield. PDC—pyridinium dichromate.

Following this logic, we developed a one-pot sequence consisting of three consecutive transformations. First, the alcohol with tethered alkene functionality reacts with borepin **4** in benzene for 10 min to generate the corresponding alkoxy-borepin. Subsequent addition of ^Me^CAAC at room temperature or heating at 40 °C for 4 h affords the corresponding borirane intermediate. This intermediate can then be diversified through several transformations, enabling the formation of new C–B, C–O, and C–C bonds (Figure 4A).^19^ Notably, the functionalized alkene products are obtained directly, without the need for intermediate purification or workup.

To demonstrate this strategy, alkene **5g** was selected as a model substrate (Figure 4A). First, we focused on applying this strategy to establish borylation methodologies. Treatment of the reaction mixture (after borylene formation and cycloaddition) with sodium hydroxide induced formal cyclizative borylation to furnish the seven-membered cyclic 1,2-oxaborepan-2-ol **8** in 40% yield (for proposed mechanism see “Notes” section in SI). Notably, while the synthesis of five- and six-membered boracycle is well established, access to seven-membered analogs remains significantly more challenging.^20^ This structural motif is also attracting increasing attention due to its emerging relevance in medicinal chemistry.

Next, dehydrogenative borylation could be achieved by applying a higher temperature conditions. Thus, heating the reaction mixture to 80 °C led to borirane ring rearrangement and formation of product **9** in 55% yield. It is worth noting that related transformations have previously been achieved using transition-metal complexes, whereas the present system proceeds without the involvement of transition metals.^21^

Beyond borylation, the borirane intermediate could also be converted to other valuable functional groups. In the context of C–O bond formation, treatment of the crude borirane mixture with sodium perborate led to oxidative ring opening to afford the corresponding 1,5-diol **10** in 59% yield, with overall hydration of the original alkene. Interestingly, transformation of crude borirane to vinyl borane at elevated temperature (80 °C) then treatment of it with sodium perborate led to formation of ketone product **11** in 64% yield. Notably, the oxidation proceeds with complete anti-Markovnikov selectivity, showing the unique regiochemical control imparted by the borirane intermediate. This type of selectivity remains underdeveloped in synthetic chemistry, underscoring the potential of this platform.^22^ Finally, the vinyl borane intermediate could also be engaged in Suzuki cross-coupling, enabling C–C bond formation and providing product **12**. Together, these results highlight the synthetic potential of borylene intermediacy and demonstrate how its reactivity can be harnessed as a platform for diverse alkene functionalization pathways.

To further emphasize the synthetic utility of the developed methodology, we demonstrated selective functionalization of a specific double bond within a molecule containing several alkenes (Figure 4B). To this end, in diene **13,** we borylate the alkene that is in proximity to the alcohol via dehydrogenative borylation, affording product **14** in 64% yield. Similarly, the same diene **13** undergoes selective hydration to form diol **15**. This site selectivity was further demonstrated in the more complex triene **16**, where the olefin located near the alcohol was selectively converted to ketone **17** with complete chemo- and regioselectivity.

Next, we applied the developed methodology to the formal synthesis of two natural products (Figure 4C). In the first example, the hydration protocol was used to access 1,4-diol **19** in 64% yield. This compound represents a key intermediate in the reported synthesis of (±)-bruguierol A, thereby establishing a direct connection between our borylene-enabled transformation and natural product synthesis.^23^ In a similar manner, (+)-harzialactone A can be approached through anti-Markovnikov oxidation of **20**, followed by oxidation of the resulting keto-alcohol. The corresponding keto-acid **21** can then be advanced to the natural product through a previously reported three-step sequence.^24^ To the best of our knowledge, this study represents the first application of borylene chemistry toward the synthesis of a natural product or a biologically relevant small molecule, demonstrating that low-valent boron intermediates can be harnessed as practical tools in complex-molecule synthesis.

To gain a deeper insight into borylene formation via the proposed aromatization pathway and its cycloaddition with double bonds, we conducted a comprehensive mechanistic study combining DFT calculations, experimental investigations, and NMR analysis. For the computational studies, borepin **6a** and ^Me^CAAC were selected as the model system. The calculations indicate that **6a** and ^Me^CAAC interact via **TS-1** (Δ*G* ^‡^ = 8.4 kcal/mol), involving B–C coordination between the boron of **6a** and the carbene carbon of ^Me^CAAC, to afford **Int-1** (Figure 5A, step A). The formation of this adduct was supported by low-temperature NMR analysis at −10 °C. As shown in Figure 5B, the starting borepin **6a** exhibits a resonance at 38 ppm (Figure 5B-purple). Upon addition of ^Me^CAAC to the solution at −10 °C, the original ^11^B NMR signal disappeared completely and was replaced by a new major upfield resonance at −2 ppm (Figure 5B-red). The sharp line shape and strongly upfield chemical shift of this new signal are consistent with quaternization of the boron center, supporting the formation of intermediate **Int-1** through coordination of ^Me^CAAC to boron. To further substantiate this assignment, ^1^H NMR analysis of the intermediate (red spectrum) was performed (Figure S1). In this case, the α- and β-protons of borepin **6a**, which resonate at 6.75 and 7.76 ppm in the starting material, respectively, shifted upfield upon formation of **Int-1** to 6.15 ppm and 7.30–7.10 ppm, with the latter signals partially overlapping with other resonances (see detailed analysis in Figure S1). This change is consistent with a loss of aromatic character in the borepin ring following coordination of the boron center by ^Me^CAAC. Such spectroscopic behavior is in line with that reported for related borepin systems in the presence of carbene-type Lewis bases, and together these data provide strong support for the formation of the proposed boron-bound adduct **Int-1**.^25a^

**Figure 5:**
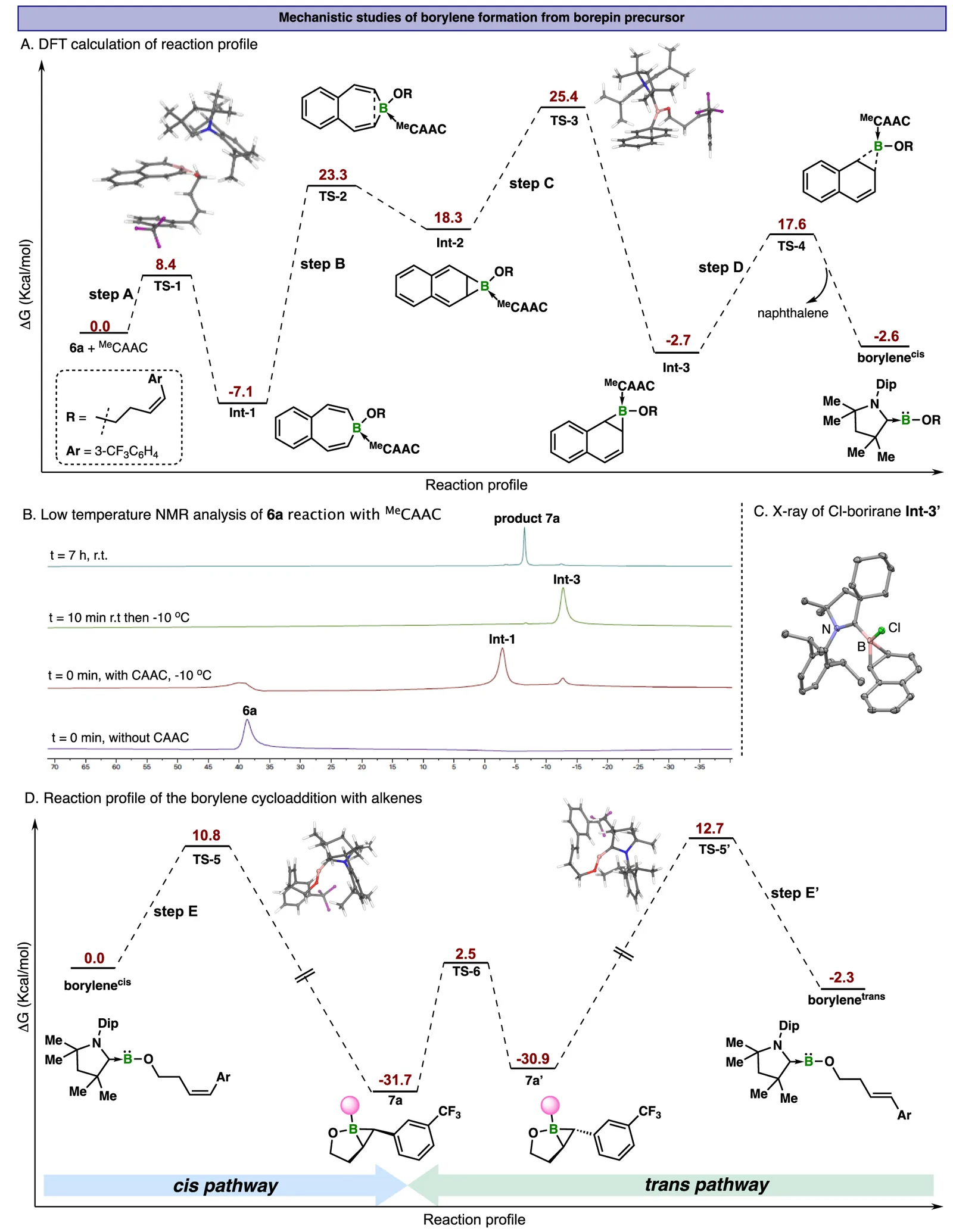
A) DFT calculation of the borepin **6a** reaction with ^Me^CAAC and the extrusion of borylene. Methods: ωB97X-D3/cc-pVTZ for the optimization and frequencies and ωB97X-D3/cc-pVXZ (X = D, T, Q) for the CBS extrapolation of the potential energy. B) Low temperature ^11^B NMR (160 MHz) analysis of borepin **6a** reaction with ^Me^CAAC. Reaction performed in toluene-d_8_. C) X-ray analysis of borirane intermediate obtained from borepin **4** and ^Cyh^CAAC reaction. D) DFT calculation of the borylene cycloaddition reaction with *cis* and *trans* alkenes. Methods: ωB97X-D3/cc-pVTZ for the optimization and frequencies and ωB97X-D3/cc-pVXZ (X = D, T, Q) for the CBS extrapolation of the potential energy.

Subsequently, warming reaction mixute to room temperature and keeping at it for 10 min, **Int-1** was converted into a new species displaying a ^11^B NMR signal at −12 ppm. ^1^H NMR analysis indicated that this product corresponds to the strained borirane–naphthalene intermediate **Int-3** (see detailed discussion in Figure S3 and Additional Data Section in SI). Two characteristic sets of signals support this assignment. First, the protons of the newly formed conjugated endocyclic double bond resonate at 6.35 ppm as a doublet and at 6.23–6.21 ppm as a multiplet, the latter overlapping with the olefinic signals. In addition, the characteristic borirane ring protons were observed at 1.90–1.79 ppm, further supporting formation of the proposed three-membered borirane motif. Additional evidence for this structural assignment was obtained from 2D-COSY analysis (Figures S4 and S5). In particular, two key proton–proton correlations were identified that are fully consistent with the structure of **Int-3**. First, a correlation was observed between the two endocyclic alkene protons, confirming their connectivity within the cyclic alkene fragment. Second, a correlation was detected between the borirane proton and one of the endocyclic alkene protons, establishing the expected spatial and bonding relationship between these two motifs within the molecule. These spectroscopic data are also consistent with those reported for structurally related compounds.^25^ Although attempts to crystallize this intermediate were unsuccessful, further structural support was obtained from an analogous system bearing a chloro substituent on boron (from the reaction between borepin **4** and ^Cyh^CAAC, see SI). In that case, we were able to obtain single crystals of the corresponding intermediate, and X-ray analysis confirmed the formation of the related side-borirane intermediate **Int-3′** analogous to **Int-3** (Figure 5C).

To gain further insight into the formation of **Int-3**, we revisited the DFT analysis. The calculations indicate that **Int-1** converts to **Int-2**, in which a borirane ring is formed at the center of the framework (Δ*G* = 18.3 kcal/mol). This transformation proceeds through **TS-2** and involves the formation of an internal C–C bond, with an associated activation barrier of 30.4 kcal/mol. Next, **Int-2** undergoes migration of the borirane unit from the center to the adjacent ring carbon, giving **Int-3** (Δ*G* = −2.7 kcal/mol). This rearrangement occurs through **TS-3**, with an activation barrier of 7.1 kcal/mol. This step occurs by first breaking a single B–C bond in tandem with a pendulum motion of the other B–C bond, leading to the transition state structure (**TS-3**). Finally, the boron continues to migrate, and a new B–C bond is formed with the furthest edge carbon, yielding **Int-3**. A related rearrangement mechanism has been reported previously, through both a closed-shell and an open-shell singlet.^25a^ In this work, we also located an open-shell singlet (OSS) solution for **TS-3** using broken-symmetry DFT (see Computational Section of the SI). This transition state has an activation barrier of 13.6 relative to the asymptotic reactants, 4.7 kcal/mol below **Int-2**, giving a formally submerged barrier relative to the preceding intermediate. The lower energy of this diradical state compared with the corresponding closed-shell solution suggests that an alternative diradicaloid pathway may be accessible along the reaction coordinate. Because broken-symmetry DFT provides only an approximate single-determinant representation of diradical states with multiconfigurational character, the resulting relative energies may be affected by spin contamination and static-correlation errors.^26^ We therefore interpret this result qualitatively as evidence for a possible open-shell pathway. The last step is the elongation of the B–C bonds, leading to the borylene extrusion and the release of naphthalene. This step involves a concerted but asynchronous double B–C bond cleavage of **Int-3**, leading to formation of the ^Me^CAAC-stabilized borylene intermediate (Δ*G* = −2.6 kcal/mol). This process is driven by the formation of naphthalene and proceeds through **TS-4** with an activation barrier of 20.3 kcal/mol. An additional pathway for the formation of the ^Me^CAAC-stabilized borylene intermediate was identified from **Int-2**, proceeding with an activation barrier of 9.6 kcal/mol (Figure S12). In this case, the transition state is characterized by a single remaining B–C bond, which can either undergo cleavage to generate the borylene intermediate or reform a second B–C bond with the central unit of the borirane to give **Int-3**. Notably, both proposed pathways are thermodynamically feasible and could therefore operate in parallel. However, the spectroscopic identification of **Int-3** and lower free energy barrier of **TS-3** provide support for a stepwise borylene formation pathway via **TS-4**.

Next, we sought to understand the pathway for the intramolecular cycloaddition of the borylene intermediate with the tethered alkene. DFT analysis of the cis-derived borylene intermediate (**borylene** ^**cis**^) revealed that its intramolecular engagement with the tethered alkene to form borirane **7a** is a highly favorable process, with an overall free-energy change of −31.7 kcal/mol (Figure 5D-*cis* pathway). In this step, the low-valent boron center engages the pendant alkene intramolecularly, leading directly to formation of the strained three-membered borirane product **7a**. The ring-closing step occurs via **TS-5** with an associated activation barrier of 10.8 kcal/mol above the borylene intermediate, indicating that the cycloaddition is kinetically accessible under the reaction conditions. The calculated transition state corresponds to a concerted asynchronous cycloaddition process, in which the new B–C bonds are formed in a single elementary step, rather than through a stepwise pathway. This proposed mechanism is also consistent with the fact that the borylene intermediate has a singlet-state character (the singlet state is calculated to be more stable than the corresponding triplet state by 13.3 kcal/mol). This behavior is in line with related singlet carbene systems, which are known to undergo analogous concerted cycloaddition pathways.^27^

As described above, both the *cis*- and *trans*-alkene isomers yield cycloaddition products with identical relative stereochemistry, indicating a convergent reaction pathway. To understand this behavior, the corresponding energy profile for the *trans*-alkene pathway was calculated (Figure 5D-*trans* pathway). Overall, this pathway displayed profile similar to that observed for the *cis*-derived borylene intermediate. Formation of the initially generated *anti*-borirane isomer **7a′** proceeds through a low activation barrier of only 15.0 kcal/mol, indicating that the *trans* alkene can also undergo efficient intramolecular cycloaddition with the borylene intermediate. Interestingly, the DFT analysis revealed an additional low-energy pathway connecting **7a′** to the experimentally observed *syn*-borirane **7a**. This interconversion occurs through the transition-state **TS-6** (Δ*G* = 2.5 kcal/mol), suggesting that the initially formed *anti* isomer can rearrange to the thermodynamically favored *syn* product. Structurally, **TS-6** involves cleavage of the C–B bond proximal to the aromatic ring, generating a five-membered ring that helps stabilize the developing structure during isomerization. This geometry allows the system to undergo a C–C torsional motion, thereby enabling conversion between the *anti* and *syn* configurations. Although both **7a** and **7a′** are highly exergonic species and can be considered viable products of the corresponding *cis*- and *trans*-alkene pathways, respectively, the barrier connecting them indicates that they are not necessarily kinetically isolated immediately after product formation. The activation barrier for **TS-6** is large when measured from the relaxed product wells, lying 34.2 and 33.4 kcal/mol above **7a** and **7a′**, respectively. Thus, once the products have fully dissipated their excess energy to the environment, thermal interconversion between the *anti* and *syn* isomers is expected to be unlikely. However, **TS-5** and **TS-5′** lie 42.5 and 43.6 kcal/mol above their corresponding product wells and 8.3 and 10.2 kcal/mol above **TS-6**, respectively. This suggests that, immediately after borirane formation, chemically activated **7a** and **7a′**, the system may retain sufficient excess energy to access the **7a/7a′** interconversion pathway before complete vibrational cooling by the environment. Our interpretation therefore corresponds to a scenario in which excess energy persists after initial borirane formation and enables interconversion between chemically activated **7a** and **7a′**. Moreover, because **TS-5**/**TS-5′** and **TS-6** involve structural changes centered on the same boron atom and two carbon atoms, coupling between the product-forming motion and the interconversion coordinate may facilitate passage through the **TS-6** region.

Within this interpretation, one or more passages between the **7a** and **7a′** regions may reduce the stereochemical influence of the *cis* and *trans* starting materials before progressive energy loss prevents further crossings. This interpretation is consistent with prior work on solution-phase hydroboration, where deviations from canonical TST were rationalized by considering how a vibrationally hot intermediate can continue reacting while it undergoes stepwise collisional cooling by the solvent.^28^ Using a weak-collision RRKM/master-equation model, Glowacki et al.^28^ showed that hot intermediates may access product channels before full thermal equilibration and emphasized that collisional relaxation should not necessarily be treated as instantaneous, even in solution. Experimental studies have reported vibrational cooling and solute-to-solvent energy transfer over several to tens of picoseconds, with some processes extending toward 100 ps.^29,30,31,32^ Vibrationally excited organic intermediates have also been observed to undergo intramolecular rearrangement at rates competitive with thermal relaxation in solution.^33^ Importantly, **7a** is calculated to be more stable than **7a′** by approximately −0.8 kcal/mol (−1.1 kcal/mol with ωB97M-V), which corresponds to a thermodynamic preference for the *syn* isomer. Ongoing studies in our group aim to further elucidate the dynamical behavior of this system, including the relevant pathways and time scales of interconversion.

Overall, these combined experimental and computational studies provide a coherent mechanistic picture for the borepin-to-borylene transformation and its subsequent alkene functionalization. Coordination of ^Me^CAAC to the borepin initiates a low-barrier rearrangement sequence, in which aromatization to naphthalene serves as the key thermodynamic driving force for borylene extrusion. The resulting singlet borylene then undergoes a facile intramolecular cycloaddition with the tethered alkene to form a strained borirane product. Importantly, the calculated energy landscape provides a possible pathway by which chemically activated products formed from the *cis*- and *trans*-alkenes can interconvert before cooling, providing a plausible explanation for the similar, predominantly *syn* product distributions. Thus, the mechanistic data support borirane formation as the central step that links mild borylene generation to stereoconvergent alkene functionalization.

## Conclusion

In summary, we have introduced a new class of modular benzoborepin precursors that enable the generation of Lewis-base-stabilized borylenes in a mild, redox-neutral, and operationally practical manner. This aromatization-driven strategy provides a simple entry to borylene intermediates without relying on the harsh reductive conditions traditionally required for accessing low-valent boron species. More importantly, it demonstrates that such borylenes can be harnessed as productive intermediates in organic synthesis rather than being treated only as highly reactive curiosities. In this work, the generated borylenes were efficiently intercepted by tethered alkenes through intramolecular cycloaddition, providing access to previously unknown fused borirane architectures. These strained borirane intermediates could then be diversified in one-pot sequences to form C–B, C–O, and C–C bonds, thereby translating borylene reactivity into a platform for synthetically useful olefin functionalization. Mechanistic studies support an aromatization-driven pathway for borylene formation, followed by rapid intramolecular cycloaddition with the alkene. Overall, this work establishes benzoborepins as practical and tunable borylene precursors, demonstrates the synthetic utility of borylenes in olefin functionalization, and provides a foundation for developing new classes of borylene-mediated transformations that were previously difficult to access.

## Supplementary Material





## Data Availability

Data are available from the corresponding author on request.

## Notes

Cartesian coordinates for structures optimized by computational methods (PDF)
Full details of the experimental procedures for the synthesis of all new substances, together with characterization data, including details of X-ray diffraction studies (PDF)
