# Supplementary material for: Aromatization-Driven Borylene Generation from Borepin Precursors Enables Fused Borirane Synthesis and Selective Alkene Functionalization
Source: J Am Chem Soc. 2026 Aug 25;148(35):38316–27. doi: 10.1021/jacs.6c11662 (PMC13564424; doi:10.1021/jacs.6c11662)
Supplement: Supplementary file 1 [file ja6c11662_si_001.pdf]

Aromatization-Driven Borylene Generation from Borepin Precursors Enables Fused Borirane Synthesis and Selective Alkene Functionalization

Jay Prakash Maurya[#a], Dmitrii Bushmin[#a], Jonathan R. Church[#b], Kartick Dey[a], Yael Diskin-Posner[b], Liat Avram[b], Linda J. W. Shimon[b], and Samer Gnaim[*a]

[a] Department of Molecular Chemistry and Materials Science, Weizmann Institute of Science, Rehovot 7610001, Israel.

[b] Department of Chemical Research Support, Weizmann Institute of Science, Rehovot 7610001, Israel.

[#] These authors contributed equally to this study.

cis-6a

F	16.83240495477423	5.30538820817225	8.16570719497708
F	16.90360097931749	3.16600792439122	7.90596574355596
F	18.66356148281209	4.35932837516411	7.53861532065781
O	10.96642348260624	4.39011513435798	1.33364853648067
C	8.69494349785472	5.42612487692572	1.85801525335800
H	9.14753040473865	6.13279677766586	2.54886948634252
C	7.36059580075090	5.55546380721415	1.75389277363043
H	6.90499506630006	6.33427852228428	2.36057025567960
C	6.36006042658796	4.84106163796924	0.96859327654436
C	5.04291497804387	5.29478127561934	1.16028233494959
H	4.88168104543681	6.11147146711683	1.85300547950090
C	3.96294888637609	4.74528220746602	0.50766353239393
H	2.96696086814749	5.12780265270258	0.68764365695435
C	4.16985940227539	3.69706168650083	-0.38192450265411
H	3.33755041449744	3.24779066366170	-0.90755884468946
C	5.44913117737552	3.23336277893590	-0.58940591267588
H	5.60963152667225	2.41702347242651	-1.28268092252785
C	6.57021310492378	3.77478072336674	0.06344876895155
C	7.83811214624650	3.14605575521902	-0.29063390349910
H	7.70148895777267	2.34992993276416	-1.01824176579715
C	9.09561723781541	3.37771061823259	0.11840148123325
H	9.84729881457493	2.73258002650969	-0.33151806233419
C	11.64351719623074	5.25984851620785	2.22622852717453
H	11.22894651016245	5.15874511910619	3.23366428345428
H	11.51202620839065	6.29699401477379	1.90721875931708
C	13.12252164762476	4.90964602678100	2.23213997178101
H	13.49031993842977	4.98372256677640	1.20400697109925
H	13.65458666332585	5.66152059389324	2.81710590874981
C	13.37248471170325	3.53344850816488	2.77086359893499
H	12.63769299554780	2.78900975944202	2.48165311908751
C	14.35246105546508	3.13769442671390	3.58053175722319
H	14.33356131440845	2.10984585519650	3.92871807955184
C	15.46609118670504	3.95151473257118	4.10578908086416
C	15.88147789547262	3.75820949236687	5.42363871039305
H	15.38067819644599	3.02057516044089	6.03689680160147
C	16.92204988928719	4.50193636996836	5.95078627159763
C	17.58036248683266	5.44878562991824	5.17624272424870
H	18.39619704901075	6.02338749996150	5.59357409621688
C	17.19095056373821	5.63107811278149	3.86142611280286
H	17.70969060264590	6.34979311195385	3.24110359668834
C	16.14738414130657	4.88739934269523	3.32986339406958
H	15.87667123264846	5.01862163562028	2.29087680631340
C	17.32938799195389	4.32591222944879	7.38663286302588
B	9.60975786676060	4.42124277054971	1.12896138477163

trans-6a

F	2.87294550909644	25.83578196263170	7.74249167589754
F	0.75157542066867	26.08894066293332	8.04667186097984
F	1.51701528700502	25.64501259684993	6.07975395468549
O	6.91483482749360	17.48754958505672	7.53410593886070
C	8.12328642595238	15.48436445333371	6.81297058854295
H	8.97053709471989	16.15291917858709	6.67611406990055
C	8.36910864853394	14.19841178745502	6.51682693563140
H	9.37374131747787	13.96062081722144	6.17601179419834
C	7.52181478375384	13.01154926185867	6.56386099546672
C	8.16044619982028	11.82267778199300	6.17104464561257
H	9.20030010192416	11.87841842286723	5.87379577934687
C	7.51996675518762	10.60430432840166	6.15066720691770
H	8.05373309736427	9.71550594543623	5.84083146029701

C	6.18492306200781	10.53195077337818	6.53144211217771
H	5.65936931557366	9.58619792157985	6.52370431821232
C	5.53478481122841	11.68113764858646	6.92020856937358
H	4.49468237828546	11.62486172248936	7.21665944872079
C	6.16396465362908	12.93819044547536	6.95167474110473
C	5.30219772087025	14.02930747580444	7.39350964614227
H	4.30074801246440	13.67335082383041	7.62289339062054
C	5.51604621998809	15.34600384153610	7.56138312738561
H	4.63742963980785	15.88353893039768	7.90896060115883
C	5.84310904857863	18.29532836561689	7.98350575396826
H	6.28604933372461	19.16962366666485	8.46226881538385
H	5.25207483592094	17.77153132623735	8.74016643328597
C	4.95683420692526	18.73727479526957	6.82330091432667
H	5.55752654704139	19.30313906998006	6.10933664909079
H	4.59262856105636	17.84351741865376	6.30690742220914
C	3.79521220849886	19.55112151340600	7.29702902290807
H	3.09353426056938	19.04012829176025	7.95118451316030
C	3.59986767041222	20.82884074755292	6.98759401706381
H	4.32888110196705	21.32473398269488	6.35243177928350
C	2.48220570850501	21.67690823802758	7.43558511814475
C	2.56138583068159	23.05102942915737	7.23600585853112
H	3.43581351061351	23.47041708416496	6.75348408488446
C	1.53840159436826	23.89055641194624	7.65505279135021
C	0.41244910105124	23.37659700014669	8.27398964681709
H	-0.38319575619646	24.03254356007838	8.59717130758261
C	0.32004513422492	22.00485009173885	8.46919285563762
H	-0.55820481641325	21.58961427832256	8.94535621830083
C	1.33720793359232	21.16660956855432	8.05370418911085
H	1.23474055165380	20.09975015566271	8.20319872518609
C	1.66557689597849	25.36398930300414	7.38784742920337
B	6.81988125439138	16.13562033365027	7.31744459333436

^{Me}CAAC

N	4.99313611105444	9.65810593823599	10.88287145561126
C	4.94464266378708	10.34653240850341	9.77764329719030
C	3.49920726513654	10.33572847459347	9.31719555427274
C	2.78529380479685	9.29522769316747	10.20735818541673
H	1.79135642175047	9.61857933712401	10.51733763404441
H	2.66717804389972	8.36005250301699	9.65733902244582
C	3.70108170870296	9.06446071786143	11.41438993236091
C	3.43318456667700	9.95991831788361	7.83786280392379
H	3.96437539754513	10.69308506488229	7.23016881879283
H	3.88808625772747	8.98313671926489	7.66184773031817
C	2.94614718880716	11.75344504156193	9.51032102351857
H	2.97512715838737	12.06219470785032	10.55640902094684
H	3.52922980024434	12.47300533990371	8.93483811732293
C	3.84706444890456	7.58766586835874	11.76312164861551
H	4.12404455417145	6.99083651889643	10.89668721837827
H	2.88823419265396	7.21841448705661	12.12811913110123
H	4.58628817471330	7.43191292086504	12.54992967938178
C	3.23197102651869	9.80912408712058	12.66294213452652
H	3.96628568723151	9.73303436279330	13.46546672539213
H	2.30531785796906	9.35631106655259	13.01618713783214
H	3.03616505331615	10.86163055083893	12.46601237110656
C	6.23295669846050	9.47980101106073	11.60000330968240
C	7.03641766243430	8.37257812099727	11.29642421882406
C	8.19475230816362	8.18014858232788	12.04141685311602
H	8.83025531405344	7.33062602546187	11.82652613008455
C	8.56176502277587	9.06781817756086	13.03618367671838
H	9.46395028335055	8.89790747693114	13.60987089917901
C	7.79553343503022	10.19485713024737	13.26630361092358
H	8.12004303271302	10.91619901523515	14.00528187845515
C	6.62878797650305	10.43332957657562	12.54640320356066
C	6.75600048142826	7.45931851908292	10.11597748673018
H	5.74050279759241	7.64438072107890	9.77336038571772
C	6.86971648986165	5.97545126753777	10.46280210453178
H	7.89407395460298	5.69674568859060	10.71483817304341
H	6.56809573000059	5.36798691420549	9.60815983618776
H	6.23345141372190	5.71194194955558	11.30861635246883
C	7.68355198623385	7.82887002155772	8.95377680965041
H	7.55448498582514	8.87552319345674	8.67636588634745
H	7.46460494658986	7.21189622304098	8.08040785480227
H	8.72999642645994	7.67385431095472	9.22465728833042
C	5.91523903357911	11.76229352494011	12.71800923943709
H	4.95170213441063	11.69838894078153	12.22050654412946
C	5.66243554414339	12.12918383990189	14.17960671128857
H	5.12172585179278	11.34096383048975	14.70503720482992
H	5.06904069297116	13.04288617167704	14.23733318036783
H	6.59390372265950	12.31137885139800	14.71794614711760
C	6.70684085199772	12.86171320570920	12.00223857058310
H	7.69660233438643	12.98408101037020	12.44712712789594
H	6.18249028024860	13.81632261575347	12.07441978053998
H	6.83382069128048	12.61853679186431	10.94700022634700

H	2.39500093362731	9.91645010011298	7.50008463937206
H	1.90860959910592	11.79988506520941	9.17308602723678

Naphthalene

C	8.58269123164508	12.08176375345986	6.26824482949189
H	8.41514001914067	11.03921630525347	6.03032457184240
C	8.30607616796582	13.07134678438290	5.29903121437029
H	7.92929877958027	12.77744895065490	4.32782313293768
C	8.51208265061339	14.38995629189235	5.58380164634652
H	8.30110094065060	15.15212250026914	4.84312128856133
C	9.00501615278195	14.78898540547310	6.85100403057340
C	9.22908077562264	16.14986216581710	7.17626556671339
H	9.01561377333492	16.90603191535191	6.43015939456001
C	9.70459940445643	16.50427643770930	8.40531866418010
H	9.87215130786167	17.54682388150708	8.64323809802577
C	9.98121516172089	15.51469331147121	9.37453218561363
H	10.35799423067083	15.80859179748821	10.34573948220961
C	9.77520729558415	14.19608415436615	9.08976207456399
H	9.98618983704171	13.43391796069245	9.83044238073833
C	9.28227443508836	13.79705419962140	7.82255955330189
C	9.05820973236120	12.43617714682049	7.49729808380149
H	9.27167810387926	11.68000703776879	8.24340380216814

cis-Int-1

F	11.62549667000400	1.83581303227725	4.50868443362336
F	9.77344271186209	2.61174885631661	3.72770594895277
F	11.46792739269120	2.35358060849304	2.41967232079406
O	8.84435497897190	8.51737250850250	8.13695296757019
C	7.70658054564562	9.36172864959340	6.01923432379684
H	7.52685913832214	10.35490984492854	6.43658601267541
C	7.86756095344512	9.38600843053584	4.69325999808490
H	7.79057209182925	10.35754556513097	4.20809525313205
C	8.10292885107383	8.31833040446725	3.70683884560409
C	8.11378089647411	8.74955226307637	2.37274896355917
H	7.96793151968623	9.80587440388486	2.17930260327430
C	8.29711951195686	7.89165820971805	1.30595213367466
H	8.29585041462169	8.27482135698916	0.29357499223485
C	8.47863259926912	6.53948815641795	1.55393490446225
H	8.62241943138361	5.84039405642752	0.74002693972915
C	8.48254466531721	6.09228219316498	2.86007362871484
H	8.63918868445792	5.03854621783287	3.05553854059802
C	8.30474268927304	6.94329990207369	3.95942090148843
C	8.36751571028082	6.27607579951984	5.26731159620083
H	8.64392078511048	5.22874965516411	5.16301847557234
C	8.14163541258219	6.72190297908884	6.50492015531307
H	8.27607082352959	5.94870024709591	7.26043543747269
C	9.98073518513142	9.26228041480626	7.81163724490109
H	10.45895227750595	9.54286029283966	8.75797332429820
H	9.71309480621002	10.19766505454480	7.30381046640358
C	11.00811874320353	8.54786920326409	6.93366696010276
H	10.54623878862841	8.26152807077141	5.98784094280471
H	11.78665687585678	9.28262169507394	6.69580802020950
C	11.63186500142785	7.36509603057448	7.60546807972575
H	11.74702051846365	7.45678060209595	8.68267109595760
C	12.03012494755312	6.21827684594395	7.05843504519797
H	12.40607964605122	5.44552623277403	7.72174271378500
C	11.96693354044894	5.84019309291392	5.63506010813473
C	11.63999131626032	4.52884841552409	5.29932969298753
H	11.43767042212016	3.81298081876888	6.08641547574859
C	11.55530858250891	4.13779526202236	3.97270549970290
C	11.80954452460305	5.04008717684773	2.95081882041925
H	11.73768334427988	4.73160683726405	1.91744786031253
C	12.15890171722657	6.34039361845960	3.27481356587948
H	12.36775059799206	7.05214374558066	2.48738917900644
C	12.23864339629415	6.73577529939470	4.59993638787925
H	12.53327864385560	7.74910953750270	4.83468063171723
C	11.11622860844276	2.73836686955673	3.65485390702894
B	7.82319018800555	8.18736309292676	7.11014289608967
N	6.13067382954951	7.83512901753949	9.14712534418025
C	6.35775628390080	7.97572909177358	7.87202773590141
C	5.03876059644870	7.84107656251309	7.11709745349118
C	4.12344928797694	7.15322284699300	8.13599377634077
H	3.09001935735734	7.48688895387549	8.05249041802270
H	4.14013116633508	6.07652725994071	7.96124186528698
C	4.70351111958591	7.45075047436997	9.51512822677884
C	5.09019283439728	7.04283132579708	5.81727718784809
H	5.72084998055267	7.52088186575789	5.07146987619487
H	5.46181734067013	6.03316288547938	5.98066439608208
C	4.54683151239217	9.26523264486387	6.78871578323231
H	4.49484236197651	9.90842307658963	7.66599342809698
H	5.19756121524460	9.74058167628517	6.05861843006991
C	4.66991680598675	6.21949523023656	10.40738923626055

H	5.15454075789849	5.36775607900516	9.93630568022483
H	3.62577720312820	5.95740644842112	10.57905205601901
H	5.13098083561514	6.40889549077085	11.37598573292263
C	3.98137642879588	8.58065376781593	10.24217867179495
H	4.47647146778576	8.81881431206875	11.18232199726783
H	2.97277930439933	8.24075740630662	10.47522430731141
H	3.89683829201060	9.48606550079598	9.64701783357938
C	7.06894733502170	8.10130830383028	10.22851626671009
C	7.80697165501026	7.05095340457736	10.78871088195135
C	8.59817623453364	7.33163117261866	11.89806678215848
H	9.17829211378131	6.53503347279313	12.34555268969187
C	8.67951557147579	8.60529262646805	12.42402294899615
H	9.30210783691190	8.79953236012143	13.28788536041983
C	7.98331846592117	9.63545508344895	11.82259694462429
H	8.08079346773578	10.64082945839360	12.21130153616529
C	7.17159509242828	9.41270004579625	10.71633546149078
C	7.89233649969174	5.65224114846666	10.20054920194724
H	7.20092055848125	5.58701394993997	9.36040873042324
C	7.54069158103200	4.55318490285021	11.20681125413553
H	8.27784233616460	4.51009741815121	12.01003745295085
H	7.54330292517749	3.58157924732330	10.71034215620693
H	6.56273560593265	4.69840336866416	11.66236680634834
C	9.30190237778988	5.41288942781923	9.65056958502130
H	9.59466112986623	6.21152295806587	8.97399911942892
H	9.34420104463794	4.46523126327887	9.10982417128719
H	10.03111914638075	5.36701252768404	10.46206999312967
C	6.52411403901773	10.62148771872518	10.05965389831804
H	5.84260242572050	10.27330822886068	9.28818775758780
C	5.72381271816152	11.47903990460837	11.04256654459203
H	4.98900477293455	10.89700171288690	11.59741456050841
H	5.19511567465965	12.26605917989705	10.50264359996991
H	6.37899641950442	11.96505567718781	11.76691227555012
C	7.58153664361373	11.47850174987208	9.35956697655883
H	8.28877038726609	11.89236064689277	10.08097886146538
H	7.10697776524510	12.31099185286901	8.83714895910385
H	8.14038686178409	10.88688339462776	8.63951207425049
H	3.54468534314713	9.19491897288047	6.36389337511677
H	4.07660983910134	6.97660535304770	5.41609704215952

***trans*-Int-1**

F	1.79993496331049	25.37521453128791	9.74030524019488
F	-0.26484989766886	24.98246732883450	10.22097359205991
F	0.32257902473053	25.32660412177835	8.17318400257517
O	5.19922978756328	18.19047070628633	3.89493886737589
C	7.61610481446681	18.09111593025871	4.61335148369945
H	7.78417379524334	19.09995549320370	4.23026068639567
C	8.41248827526234	17.78848838174847	5.64235395858277
H	9.12566098696016	18.54967764010573	5.95390114938063
C	8.55000220047885	16.56668126962656	6.45122850984407
C	9.64207109468860	16.56572966153252	7.33026137576245
H	10.28295716977361	17.43947192301361	7.34438333144874
C	9.93076878677186	15.50831307969265	8.17014568857980
H	10.78869149267088	15.55517400645830	8.82846927304311
C	9.10342203805772	14.39551015088911	8.16045866185398
H	9.30000817694623	13.55300700674013	8.81094011311639
C	8.01568219685865	14.37709237584189	7.31013065380892
H	7.36420330195569	13.51107665648251	7.30726037646413
C	7.70824885430971	15.43281201193757	6.44041949607283
C	6.51482392947391	15.21643883514542	5.60891257972393
H	5.99407520519548	14.30384551185609	5.89406999788326
C	6.01279814943032	15.89859449026962	4.57646406871888
H	5.11843538498321	15.43449528646040	4.16119386784919
C	4.65287137595200	18.57883624208780	5.11723872307974
H	5.38127138423216	19.13457153607903	5.72570274826618
H	4.35477262047602	17.70484274314759	5.71723180662819
C	3.43363370111427	19.45868535921647	4.85688112247600
H	3.74314594247312	20.35532194960029	4.31599004381747
H	2.75714159076573	18.90485838628305	4.19808456871223
C	2.72349121487686	19.82685978461984	6.11785314861025
H	2.32089427563183	18.99695877900834	6.69331881449227
C	2.57264889656608	21.07050121173924	6.56299275390318
H	2.96842319728064	21.88575616302475	5.96317911407846
C	1.88802556000877	21.48128956051377	7.80044585937808
C	1.60154006936324	22.82942409880547	7.99354430663998
H	1.88892307553751	23.54800096310536	7.23600921600520
C	0.94511124289666	23.26109522385345	9.13733626949516
C	0.56819531683195	22.35936040519036	10.11807242012554
H	0.05845425494142	22.69859388452094	11.00870726922314
C	0.85770844726709	21.01358951743498	9.93913733920760
H	0.57409079421538	20.29891640396912	10.70034533441101
C	1.51037956573873	20.58056207507989	8.79995921261838
H	1.73753374905128	19.52821398312206	8.69052673786023
C	0.69469146181608	24.73138097287327	9.31865664686438

B	6.39472581679053	17.31878262717366	3.90627016886228
N	6.00313076377588	17.16632577533758	1.28535161733051
C	6.75424968085892	16.96916861137835	2.33017489952506
C	8.04720110823732	16.29152488804208	1.89720392460533
C	7.70109364998457	15.73971923476062	0.50982824659832
H	8.55242139116413	15.76674824095214	-0.16889876808619
H	7.38599455862864	14.69974202176035	0.60649020064563
C	6.53513998027362	16.57475970791329	-0.01264803631330
C	8.54248367978463	15.17959098226082	2.81783154426532
H	8.81062247577392	15.55873480362888	3.80151862606385
H	7.79259830524049	14.40080571450444	2.94528820634623
C	9.13906431447320	17.37723561294616	1.81779140558542
H	8.85356267729757	18.21892653346898	1.18832711618527
H	9.37621364272079	17.76013179017400	2.80760105699787
C	5.49229390462051	15.70333288592643	-0.69522790298127
H	5.15758087545597	14.89708820248828	-0.04668065666701
H	5.95231292303785	15.25535186721062	-1.57620024399855
H	4.63151579239152	16.28381864280074	-1.02499176828096
C	6.96374893458680	17.66340004393514	-0.99098765318594
H	6.11666336120811	18.28339837826031	-1.28044570401992
H	7.34444123038232	17.17981502389077	-1.89005021336469
H	7.75090686289516	18.30150025099790	-0.59759897593284
C	4.78606160582983	17.96452317065987	1.21940726582814
C	3.53574066069256	17.34267783317783	1.32635512096904
C	2.40244827093874	18.11954686136665	1.11061294934739
H	1.42565926355221	17.65995521222250	1.18878027383046
C	2.49461569055841	19.46808705816266	0.82810564029792
H	1.59892055645377	20.05275476318946	0.66275963093524
C	3.73588378512271	20.07256783678859	0.79553736026102
H	3.80283970008247	21.13951827761495	0.62685619884540
C	4.90294074287193	19.34455119825827	0.99842456239231
C	3.33115278257156	15.90906258055842	1.78777447704481
H	4.30727159527916	15.43286256464918	1.88344083790951
C	2.47892048563088	15.07983217195407	0.82331139102594
H	1.45496002795624	15.45462089068923	0.78574265251854
H	2.43403557165833	14.04408522919018	1.16345618030241
H	2.87120349158205	15.08657209568285	-0.19209833244258
C	2.67977874308237	15.90447986108859	3.17608543528281
H	3.24253214301755	16.52190246948306	3.87260621589707
H	2.63315745059533	14.88753753987716	3.56971269001641
H	1.65988548318197	16.29017349531809	3.12424792453359
C	6.21188660856801	20.11369610114764	1.07909577564144
H	7.03008393898888	19.40053068213668	1.14727182222100
C	6.46292314092667	21.00399473735417	-0.13984775238857
H	6.40893577420339	20.45006194047791	-1.07621293119349
H	7.45318934944927	21.45673699423506	-0.07056973723882
H	5.73683709704437	21.81661038682090	-0.19152846798409
C	6.24745240388958	20.95519856598637	2.35943442113027
H	5.48569854453295	21.73717698628487	2.32946324874226
H	7.22033511679421	21.43844576668528	2.46591970152409
H	6.06315702049236	20.33100414517680	3.23130289767683
H	9.43210632942046	14.73168936455331	2.37031724780259
H	10.03967322694403	16.93099573665280	1.39404777875984

cis-Int-2

F	8.94892029221115	2.81095560105766	6.34616286383585
F	8.49853835580477	3.28788112315600	4.29014820378145
F	10.03208914709399	1.84734448702664	4.75002682385524
O	9.32872953570720	7.79630360121739	7.76808219209701
C	8.47596611113806	8.95464653784795	5.60080115353405
H	9.33434071967122	9.58537700978761	5.81768848518226
C	7.51925697433181	9.61186680363178	4.72120086198900
H	7.37425094058302	10.68010533387981	4.85182613689049
C	6.87753565497972	8.99644583940665	3.70357565315535
C	5.91710814486860	9.68294796788487	2.86040935414129
H	5.67698963697602	10.71291353165767	3.10000441642796
C	5.33062647876891	9.06850395735631	1.81917043262129
H	4.61192614377596	9.59822929232024	1.20655767243530
C	5.65682746284647	7.69424036611557	1.48826416396741
H	5.17539932449836	7.23073386642297	0.63659823783132
C	6.55025038059704	7.00893830157430	2.22200928771367
H	6.79725309140654	5.98207844315158	1.97599145254594
C	7.19806052771600	7.60148827002931	3.37490592249996
C	8.11232660409945	6.93123065943303	4.11405190014127
H	8.43136737729971	5.95232265722176	3.77006127865899
C	8.76542783497808	7.46216695848715	5.29540634061099
H	9.79646554617989	7.13646851574601	5.38776370867038
C	10.24713382721185	8.80500184131811	8.06101612919205
H	10.36201782842503	8.85914531415627	9.14997533123364
H	9.89027434221858	9.78646401047689	7.74003918787002
C	11.62064122080064	8.53982243482311	7.42791616976243
H	11.48905069823402	8.36050248673095	6.36092606960137
H	12.22552404178479	9.44449444636991	7.53635807920346

C	12.31741049293170	7.39887265503385	8.10177699374175
H	12.68669098064096	7.61447010377368	9.10107224373225
C	12.45225570122232	6.15018981034227	7.66752987490274
H	12.90332055925576	5.42001676170200	8.33250069413702
C	11.97191366932574	5.62373691312571	6.36970554691737
C	11.01857653829704	4.60688209142041	6.35871850737839
H	10.64065712219504	4.22143891256116	7.29610381934134
C	10.53165158808808	4.11682758988428	5.16040759230724
C	10.99851048511188	4.61272180553171	3.94894124600882
H	10.61221969380926	4.22473764333911	3.01572915200064
C	11.95517225488430	5.61110393708779	3.95245279328776
H	12.32430370835024	6.00639508476080	3.01563307190752
C	12.43876337341021	6.11139822430617	5.15392963627424
H	13.18860930245509	6.89221610216898	5.14995919601705
C	9.50423756114750	3.02140758311383	5.14420211233510
B	8.25448818985642	7.88002197989758	6.78764039370394
N	6.54420001503635	7.88796302787192	8.70042824019809
C	6.84602677537315	7.53542576400721	7.46900022810093
C	5.71132429110670	6.66853958154378	6.91754064522880
C	4.84968541137589	6.39298945484943	8.15896419002842
H	3.79003693523333	6.32375784310404	7.91918861241869
H	5.15342228793267	5.44519079460961	8.60712958898923
C	5.13930811682958	7.51518604526946	9.13852905906211
C	6.26030948697742	5.34292713753990	6.38293024714262
H	6.81703621914243	5.47581147229961	5.46082965709468
H	6.91436415518052	4.86710666108205	7.11526256155810
C	4.89408000484921	7.37520031744496	5.82931117576806
H	4.60338811119514	8.38681009191952	6.10902876371496
H	5.44592098828597	7.43480525528559	4.90045955336834
C	5.04359357896334	7.04898784919722	10.58231950027799
H	5.59037983372412	6.12710533964533	10.75211513781992
H	3.99293815655468	6.85387091825804	10.79774329053712
H	5.39044851589998	7.80935534348532	11.28160334995102
C	4.19815824247459	8.70718374332682	8.97331616888849
H	4.42305983106986	9.47764034076264	9.70667414980718
H	3.17822853791498	8.36792101428345	9.15216200691074
H	4.23958406511117	9.14659922533538	7.98031761986778
C	7.38635207642981	8.64253312300577	9.60939535871344
C	8.13534509112656	7.93395292714703	10.56786894971352
C	8.86846421663226	8.66585691363031	11.49203800529011
H	9.45502637613419	8.14001780392007	12.23430257446922
C	8.87478753656836	10.04906969653424	11.47842296051691
H	9.45115645570752	10.59850229436792	12.21154748793457
C	8.15483461347878	10.72189164240801	10.51490356377720
H	8.17670446154207	11.80385014909929	10.49564659571200
C	7.40045139549958	10.04287685666638	9.55865826874490
C	8.22721177091564	6.41536481422591	10.63702170372690
H	7.51872130567690	5.99422240394399	9.92460323187542
C	7.89001551358970	5.88320992899955	12.03520741476366
H	8.68683761963783	6.11867395741333	12.74239742143845
H	7.79353388968649	4.79677498246193	12.00618661531853
H	6.96477786355605	6.29807422045303	12.43215870634392
C	9.61507405234156	5.91317636587145	10.22920701955318
H	9.85434368939212	6.21893982467318	9.21447864669054
H	9.64703224015920	4.82349199142238	10.29241567494795
H	10.38350292065397	6.30631366727455	10.89890469711770
C	6.66882958989152	10.88360373666696	8.52269090038558
H	6.12521458835353	10.21730651985565	7.85487616578167
C	5.67300454377111	11.85207708506991	9.17179381039677
H	5.01297930509488	11.36264577289558	9.88565452528985
H	5.05588984315378	12.32375295227233	8.40575193986278
H	6.19865421692240	12.64615326073934	9.70490356247756
C	7.64625633180103	11.68786066164176	7.66185742524061
H	8.21982413179352	12.39056250311933	8.26860518206714
H	7.09691756049762	12.26427506838507	6.91570837897218
H	8.34276768378388	11.03744052200023	7.14052606011279
H	3.98604008017248	6.79914914040395	5.64706908702155
H	5.42467601061337	4.66813513597065	6.18622393556482

cis-Int-2 (Configuration 2)

F	12.13554619990892	3.59000019420493	10.62482093871122
F	13.24421269451354	2.21170833210973	9.39378620965798
F	14.29126898936095	3.61456071425899	10.65566556774575
O	8.49346371472110	6.95604214901055	6.18379662092233
C	7.46436303711608	8.97541984176983	4.92561068965396
H	8.51848923427554	9.15656891459711	4.73159046123523
C	6.65783412154192	10.17215430135069	4.73768817859851
H	7.09986351445146	11.12092486255635	5.02741518232592
C	5.44252207279451	10.18434716682567	4.14653239136925
C	4.66410348894790	11.39915065547048	3.99738379099863
H	5.06153106923421	12.31157888867946	4.42789477367940
C	3.48308355550249	11.40306540048139	3.35652768718806
H	2.91569902613960	12.32032527516653	3.26167004675535

C	2.95455738692810	10.18683136836480	2.76899840649036
H	2.00641444754167	10.22467157450448	2.24789424525184
C	3.63534795763247	9.03173380152899	2.86018331178200
H	3.24246094251708	8.12274495389981	2.41833872820940
C	4.90023269369432	8.95031199303941	3.56340726680091
C	5.60826400270434	7.79998276189177	3.64976832617982
H	5.25594040369387	6.93747709304436	3.09102467889115
C	6.85966294005975	7.65654597254001	4.37084280532424
H	7.55142479313846	6.97234272824333	3.88705840742197
C	9.79596404264471	7.45834534666855	6.11923456524608
H	10.35787415648376	7.04839030553375	6.96254318261216
H	9.81592124720706	8.54461453015476	6.23135628621435
C	10.50868922902051	7.08676463404283	4.81379796557042
H	9.90435311038882	7.43051868777629	3.97327630704681
H	11.44304237944066	7.64872542903429	4.76274725995276
C	10.75685818253291	5.60949883835903	4.66372622662383
H	10.19391124466200	5.11708682384597	3.87812231723468
C	11.53261693320208	4.81465174383443	5.40128723225061
H	11.51078589246574	3.75480064646296	5.16774332854369
C	12.37207799381684	5.15247577691084	6.56844116669549
C	12.48288484438887	4.21134374215981	7.59002841325634
H	11.97332963434786	3.26097878726348	7.49263770106529
C	13.22127451007050	4.48122425061028	8.73111365919377
C	13.89117989826561	5.68681552088537	8.87118237676594
H	14.46887645138548	5.89324937973398	9.76123456761732
C	13.82014657637822	6.61434361892116	7.84392209723670
H	14.35456465549088	7.55120722108968	7.92869882821142
C	13.07103126802684	6.35217346162690	6.70920323394756
H	13.04214001723380	7.08747456761323	5.91852284790767
C	13.23317342491038	3.47455665471855	9.84532884319596
B	7.28615717017033	7.73190652042063	5.94311413593938
N	6.78185652254655	7.78676757560406	8.46195637386452
C	6.32574438840086	7.74257937390841	7.22884188902037
C	4.80281361983046	7.58494501188957	7.27872099628707
C	4.53544122527417	7.27983043318194	8.76060853246045
H	3.58309987540915	7.68283333168726	9.10070015025609
H	4.51311256392613	6.19860678623973	8.90914195586286
C	5.70684335252628	7.85794010947347	9.53223318455273
C	4.35975369511245	6.38879835840001	6.43209755992525
H	4.47052634127855	6.58354988657370	5.36973015072905
H	4.93591786292139	5.49720331248369	6.68468052380915
C	4.05191192372070	8.84523119819110	6.82968187145497
H	4.45878365564280	9.75874975370811	7.26041730418214
H	4.07488338516054	8.95084557392426	5.75275931815216
C	6.02614175053711	7.05218893881247	10.78115124137740
H	6.08218825945726	5.98746419098622	10.57957363867639
H	5.21693714435206	7.21110878442081	11.49401041690028
H	6.95310432078474	7.37883156923188	11.25194545204787
C	5.47316986523440	9.30597251852890	9.96106938679766
H	6.32319524925158	9.68434682893539	10.52283046940190
H	4.60442517290491	9.33221965068706	10.61833215215106
H	5.28302915400023	9.97168286333488	9.12353246524334
C	8.17694025443601	7.82258085429351	8.86034430122088
C	8.80036012061124	6.60844219026844	9.20572804944630
C	10.11338986502929	6.65545546343419	9.65414202055290
H	10.61737805014933	5.73351797678523	9.91361533624552
C	10.79660530064381	7.85331057226519	9.76275569670315
H	11.82015594156033	7.86313307833549	10.11416108888506
C	10.17186903703806	9.02852090823574	9.40468940200403
H	10.71501037198465	9.96198973820353	9.47764349258949
C	8.85594933846105	9.04502196200005	8.94337873785510
C	8.14033964847080	5.24122521531284	9.09013433060133
H	7.09031003522697	5.38458230720946	8.83786884784880
C	8.22486546253023	4.45467144296528	10.40397476111492
H	9.24707974507866	4.12319040815432	10.59153454189833
H	7.60022239872597	3.56202081663551	10.34388842219615
H	7.90458720133654	5.03879807612740	11.26562762207623
C	8.75865318382688	4.40572193861150	7.96654104999059
H	8.68137060605956	4.91921334224132	7.01176636248810
H	8.25140694056261	3.44127353242130	7.90040791875456
H	9.81286419683409	4.21320552247428	8.17208176760776
C	8.26678192834238	10.39321349136916	8.55604522569469
H	7.23859747505910	10.24203084689866	8.23241733403438
C	8.27315961550790	11.37145365657609	9.73716262225035
H	7.86432313339467	10.93419830241392	10.64673675587662
H	7.68947914458644	12.26015585566564	9.49290878052624
H	9.29009117391519	11.69745454457924	9.96227720879270
C	9.01799787508899	11.02986734811098	7.38415945575539
H	10.06611195715528	11.20310158795141	7.63414096292657
H	8.57097271437700	11.99438208190463	7.13754423747949
H	8.97632446295755	10.40336576344196	6.49752358365345
H	3.00954310858660	8.75866919964978	7.13844180396262
H	3.30730123523567	6.18247049405317	6.63764198829190

***trans*-Int-2**

F	-1.08597611727491	24.58074612453175	6.80290166837436
F	-2.18834312227705	23.98521375323947	8.55891465738949
F	-2.33601875736619	22.82802579826438	6.74366776370022
O	6.10716745962788	18.83609312758251	4.14890811561841
C	7.81826307277519	17.31256981871674	5.28254538024869
H	7.89216133591077	18.28701796574900	5.75918881522747
C	9.00025807754595	16.50023449877314	5.46741994234844
H	9.95271200592423	17.01669191252878	5.54120256391166
C	8.98189096181959	15.15618868036347	5.64442731384533
C	10.19325636536662	14.38105166488203	5.81381327625143
H	11.14160712729345	14.90365161746304	5.75835794359945
C	10.15324171929973	13.05336012759615	6.02138217933270
H	11.06978425974511	12.48954101036888	6.13919448416488
C	8.88484973059979	12.35725099412143	6.10092919341376
H	8.88369420811675	11.28875482832367	6.27544042893219
C	7.72528942967761	13.02448041252083	5.97083778642054
H	6.77714983844548	12.50271479941837	6.03515225622217
C	7.69808128874065	14.45297338498597	5.72740480493832
C	6.54257189754926	15.14586905177969	5.61376274111500
H	5.60680795295403	14.62689901651031	5.79777965715416
C	6.47263533392631	16.57814051617673	5.37307128823402
H	5.69009493003912	17.03934205196331	5.96908336533746
C	5.41422080747601	19.33853251337376	5.24738879686226
H	5.50166579273608	20.42972657686196	5.22392937027647
H	5.85982095863681	19.02177656239753	6.20132628632869
C	3.92375278200137	18.98869191072414	5.23244730932796
H	3.50181970198058	19.31839580008915	4.28379879263742
H	3.81214201456618	17.90256011340142	5.27537369250616
C	3.19435664925432	19.62042214964590	6.37163901710618
H	3.47354458625081	19.28579727702128	7.36774475389337
C	2.26294030216529	20.55863550982583	6.22643946999819
H	1.99128005580903	20.86056047758932	5.21805668318490
C	1.52051702168368	21.23588243321398	7.30243048525219
C	0.42166512632204	22.02033260676149	6.96910847364229
H	0.13477968417598	22.12066263032339	5.92919472114621
C	-0.31432779887381	22.67029204380131	7.95086524119128
C	0.03329498006983	22.55658380848727	9.28507280776940
H	-0.53969392457244	23.06532539948353	10.04696364828086
C	1.13416266814603	21.78173617499288	9.62779350128534
H	1.42141049717734	21.69009901919580	10.66687680014241
C	1.86842651465610	21.13404064565869	8.65290526758273
H	2.73034036390111	20.54814601537140	8.94420507980028
C	-1.48129038387837	23.51433521419919	7.52311006741506
B	6.71821542475153	17.51473921899069	4.06338908041000
N	6.22543206541729	17.69863665175128	1.56939100865195
C	6.89531939586456	17.10370862517391	2.55024365358719
C	7.88313733011301	16.11365766496717	1.92589334158757
C	7.45133699905254	16.06814316505998	0.45351631762844
H	8.29785253675167	15.92080133333226	-0.21550378159130
H	6.76404601270025	15.23277054727421	0.30615955122765
C	6.71755466518522	17.36822184136410	0.17571364801484
C	7.75675614676429	14.70070161151117	2.49644395218486
H	8.25711454277702	14.59860821649914	3.44929225642322
H	6.71340586493318	14.40630127481422	2.62188805447764
C	9.32883446484013	16.60044456667539	2.08986178152606
H	9.48142720097869	17.59714526631098	1.68022693976630
H	9.61116193130595	16.62015245550421	3.13903696276338
C	5.58249423290659	17.18766116272854	-0.82194620828568
H	4.90181869531058	16.39386190225976	-0.52510379496243
H	6.01810669494566	16.91204239574367	-1.78265454960003
H	5.01337114159056	18.10635157042360	-0.96111713727520
C	7.66669491858225	18.44219242054038	-0.36210737649559
H	7.13225785224836	19.33985660013080	-0.65747535804796
H	8.16070560068702	18.04595501545491	-1.24945529016081
H	8.43378179167801	18.71764994295372	0.35769741816328
C	4.96915807964035	18.41061423607931	1.71040200991555
C	3.80626689541778	17.63513004180703	1.84682786969673
C	2.58076373900123	18.29307291180450	1.89680256523126
H	1.67376485075649	17.71391180066033	2.01053236311011
C	2.49856743236746	19.66796983596175	1.80551478568438
H	1.53537176548292	20.16044554427307	1.84480845914908
C	3.65587643406196	20.41140093962153	1.66873512041446
H	3.58891637970907	21.49014534320003	1.60563042061393
C	4.90681992333896	19.80740943524873	1.61679798094949
C	3.80271764152927	16.11627872530137	1.94928562806819
H	4.79105310296071	15.74855117591750	1.67777766379161
C	2.79713183614298	15.46825486427036	0.99296826722848
H	1.76912719268097	15.65980232005330	1.30339000213911
H	2.93837525508340	14.38657226133794	0.98611532992928
H	2.90694654838988	15.83255228185300	-0.02877601593530
C	3.52989663422601	15.65866301039833	3.38339600269628

H	4.28523662299570	16.04625629468795	4.06370707702541
H	3.54384220962604	14.56875089670018	3.44176886411358
H	2.54989912661506	16.00279478545580	3.72062878417271
C	6.12231339015839	20.70303131691402	1.46106592358615
H	7.00470236421980	20.06907388255000	1.42852359509902
C	6.04137496074646	21.51067177401601	0.16001489398527
H	5.79279664733976	20.88776257434487	-0.69993227370537
H	6.99244274693089	22.00709002665139	-0.03911571780535
H	5.27443106022027	22.28396942853143	0.23176926074209
C	6.30893415186668	21.65221956906796	2.64582436847447
H	5.42721124556796	22.27661504582812	2.80223396112655
H	7.15540063766661	22.31537360096357	2.45816003849420
H	6.50582016689971	21.09026553240822	3.55406312189228
H	8.22292044193133	14.00551057341161	1.79550631634603
H	9.99812626461438	15.91306724912478	1.56976394946431

cis-Int-3

F	9.16407561965468	2.58906750269350	6.84165884132300
F	8.85219576038560	2.81208661027891	4.71798741745838
F	10.38488659382859	1.48641641517830	5.44741993750954
O	9.40114385097877	7.84346656204673	7.72155675044767
C	8.92446259859449	7.20280613277467	5.28338917152281
H	9.98468837368493	7.00789196173175	5.41104366853754
C	8.49292980390728	8.65848858264656	5.39722974738850
H	9.28978054551512	9.38877355119185	5.50700770236701
C	7.45458040619888	9.13532006096606	4.44761439913469
C	6.97065550456739	10.43885236456711	4.52152664728455
H	7.34873304370482	11.09219308929346	5.29681085685347
C	6.01437794147064	10.91435329178969	3.63637654386209
H	5.64821089937656	11.92905687674241	3.73220959778594
C	5.53244284512404	10.08753868073883	2.63167716369045
H	4.78558208189216	10.44638274182757	1.93503674943714
C	6.02497829467417	8.79719683876685	2.52428107188815
H	5.66414809771907	8.14476497924442	1.73679836878469
C	6.98055641594027	8.30971070179990	3.41377097620214
C	7.48943546649298	6.94393062429779	3.28870863530821
H	7.12908633254783	6.34346456409351	2.46127425221756
C	8.36650258705818	6.44373458397989	4.16325810742097
H	8.73916406979888	5.43568386084492	4.01629354050994
C	10.30966380502863	8.88746290017965	7.87429867393838
H	10.34646913783219	9.15972466904769	8.93617323521754
H	9.99777561870539	9.78301103121530	7.33155535920574
C	11.71832601102160	8.50233769130707	7.40483884510760
H	11.66495759115782	8.17875942101981	6.36590686134635
H	12.34221697360967	9.40033931352489	7.43593383017606
C	12.32066865509453	7.45113862771607	8.28306291160017
H	12.59223701058625	7.78655638121397	9.28077245484229
C	12.47500827922877	6.15774829753488	8.01852074688804
H	12.84246825025753	5.51068065201908	8.80900774719008
C	12.10826305309824	5.47916033497191	6.75543654296816
C	11.18765793455992	4.43324102034772	6.79145435213312
H	10.76229720366164	4.13108127982249	7.73857612829307
C	10.79410538417076	3.80429314577182	5.62441358372613
C	11.32695016515853	4.18549778982746	4.39891916681399
H	11.01402146071279	3.68963722899285	3.48968282826539
C	12.25668077938638	5.20842829643645	4.35754964523232
H	12.67975255684589	5.51329538031533	3.40978151809902
C	12.64491092872112	5.84882830028612	5.52651991007783
H	13.37574930139561	6.64633485784486	5.48596139632699
C	9.79987331213434	2.67933745224717	5.66434303234261
B	8.35532892326774	7.73107032991994	6.71895549125245
N	6.59486739790705	7.91008740277750	8.58028017927469
C	6.94224797857053	7.42237331590724	7.40906104068305
C	5.83923793241943	6.47168487438836	6.93070946035417
C	4.90267703908199	6.38416582882120	8.14881917290131
H	3.85848112617931	6.27868817219549	7.86074526804841
H	5.17013820933831	5.51765057634414	8.75668308453662
C	5.14578553180510	7.64961632713387	8.95013478132156
C	6.46235803024629	5.09444855919001	6.66823848769023
H	7.09504896932119	5.10161271072942	5.78561886828908
H	7.06231294104292	4.76398041894780	7.51788556553277
C	5.08503732883338	6.93778170505665	5.68118713576800
H	4.79945827929898	7.98785179272766	5.71876785201835
H	5.68314079440012	6.78660863658221	4.79204740661866
C	4.92088713822324	7.46151573023189	10.44114034975015
H	5.44922858546484	6.59695860774647	10.83153068982558
H	3.85481470417926	7.29895597335643	10.60053775343926
H	5.21373712774197	8.34388537051492	11.00972959158953
C	4.26526495186873	8.80488291313242	8.46907782723648
H	4.40307241509859	9.68938783804472	9.08409700717927
H	3.22168204487155	8.50220389833118	8.55421762659442
H	4.45923945989226	9.07103038943703	7.43251378594343
C	7.45963215216057	8.58295492987982	9.53259569117373

C	8.13510565871581	7.77406303405865	10.46582034157424
C	8.92282267920960	8.40044647864741	11.42058252198145
H	9.46384330200380	7.79701713571018	12.13776980537653
C	9.02702321273309	9.77968236342521	11.47889224754508
H	9.63954816144867	10.24782812014852	12.23860331307124
C	8.35003276681673	10.55117646516703	10.55989011615768
H	8.43711373311407	11.62906848047058	10.60543372514092
C	7.56084999129798	9.97599093353086	9.56354533464509
C	8.06652059366109	6.25310408878152	10.47681086437518
H	7.26720807617267	5.93975290003858	9.80713471600787
C	7.74901239682623	5.69854737077476	11.86943345261146
H	8.59331875747455	5.82742575495188	12.54813745665977
H	7.54592140499144	4.62866347408761	11.80406337088797
H	6.88461498539086	6.18330060289641	12.32364046970131
C	9.35801981287694	5.62448193893524	9.94993086762303
H	9.58676541925938	5.99141857525935	8.95233899914907
H	9.26172626306219	4.53684860121784	9.92844528755738
H	10.20132563050470	5.87400966081939	10.59817942478047
C	6.86691768932053	10.91167387579031	8.58923504063435
H	6.32441731147859	10.31182118833775	7.85961333203870
C	5.87442361583922	11.82639797512148	9.31730064280055
H	5.22245320401546	11.27544437075857	9.99471849472074
H	5.25132099368681	12.35983086457732	8.59804518063971
H	6.40476193255991	12.57114898940296	9.91356166844686
C	7.86762492362702	11.77227825899439	7.81556547623688
H	8.47840915576575	12.37500144381531	8.48920497770288
H	7.33616236170756	12.45591007096525	7.15151465340537
H	8.53371355990815	11.16069931764796	7.21090283746852
H	4.17689942494739	6.34216322691132	5.58095988286476
H	5.66168337488279	4.36761348821815	6.51673246444401

cis-Int-3 (Configuration 2)

F	11.83032284780202	3.35747345865376	10.55483209301907
F	12.96112551108805	2.00462391007109	9.31640399393985
F	13.97791105523587	3.27848792529288	10.73050455158559
O	8.78513034095404	7.23527087099704	6.18473364602101
C	7.23569742905300	7.82030934378277	4.24919478815477
H	8.02088114294919	7.21234316948233	3.80926393276901
C	7.67256064161532	9.16680721515123	4.81435101378972
H	8.71090831268709	9.44514481824823	4.65400263453239
C	6.76366853112310	10.32036227509388	4.59109865800826
C	7.07375931583336	11.57732933783280	5.10431807383034
H	7.97948948521451	11.69531521078929	5.68424375178137
C	6.24987632821644	12.67394677013999	4.89932576440956
H	6.51349815364360	13.63339839245588	5.32687116293405
C	5.09225805402454	12.53671494338331	4.14658916127727
H	4.44052887689087	13.38484356862168	3.98005795454735
C	4.78466372257623	11.30157706050074	3.60059753488571
H	3.88973793190627	11.18402854814341	2.99956896571904
C	5.60391071824078	10.19319321821845	3.80723908575115
C	5.26732303095270	8.89785066788364	3.21698605166447
H	4.38719105242631	8.84297170328702	2.58663009431250
C	6.01920699625602	7.81572502602601	3.43603338175921
H	5.75548921666167	6.88319239340070	2.94664462659705
C	10.07526658326886	7.75620913729183	6.11110593532290
H	10.62826925233944	7.40057746859395	6.98550448917719
H	10.07533206807204	8.84775711543507	6.16146731020849
C	10.81490684535980	7.33309783646047	4.83706314802046
H	10.28852683504260	7.74141245263029	3.97316532167027
H	11.79927301257667	7.80561764973149	4.84029882740179
C	10.92359387548856	5.84049897867965	4.67309144646782
H	10.35672970168997	5.42356448734426	3.84749040694014
C	11.56662959348616	4.95140349826812	5.42997870665579
H	11.44360354678484	3.90640226843743	5.16303858502683
C	12.37564422385096	5.16151792168571	6.64628782066863
C	12.36587302952552	4.16356721298736	7.61835725331802
H	11.78287474566512	3.26633444584710	7.45154075456173
C	13.08303704869303	4.30676366531134	8.79570402247195
C	13.84773124245053	5.43969229987324	9.02389694056216
H	14.40902641486590	5.54703966472334	9.94139834032951
C	13.89178606109610	6.42418149486245	8.04880673598355
H	14.49952412425198	7.30574788428285	8.20372071082485
C	13.16646163395990	6.28845644291969	6.87756050874775
H	13.23018376285890	7.06414609340428	6.12847452602135
C	12.97354224440249	3.23782463196668	9.84471570711493
B	7.55362704559267	7.92313264038780	5.84645195045460
N	6.93055998619293	8.00023515520825	8.33757082859123
C	6.53500607486248	7.91919165150138	7.08609873541655
C	5.01523202475104	7.72616998820132	7.06586365892497
C	4.67378038495175	7.50399726779413	8.54918264391704
H	3.70031084271356	7.91144006597821	8.81540300621551
H	4.65815352013400	6.43383073480001	8.76470330069657
C	5.79669840757199	8.15321915465095	9.33623717782660

C	4.68255847673834	6.45461361276324	6.27639051309727
H	4.85802677919428	6.58633861124624	5.21289895029280
H	5.27797936554422	5.60880179836969	6.62454072557575
C	4.23651203419664	8.90943118983323	6.48043115676719
H	4.58848391152667	9.87511961022251	6.83994774768234
H	4.30072707200589	8.91507998472045	5.39984115617767
C	6.06144889765082	7.46585392244294	10.66522948435186
H	6.17283923382203	6.39136380436473	10.55634691970099
H	5.20312032919884	7.64717896958549	11.31234825756036
H	6.94524010272261	7.86690367440939	11.16084877680291
C	5.51896438287092	9.63361411974785	9.60372296110177
H	6.29754821972604	10.07767863217357	10.21652639148989
H	4.58097578111425	9.71308457550225	10.15305808134980
H	5.42632756611893	10.21259160794651	8.68786292754100
C	8.29465309034347	7.89162892170557	8.82617380648069
C	8.76857653977075	6.60049333626533	9.12820805980283
C	10.04750148699133	6.48200007056370	9.65242480834099
H	10.43849605721244	5.49877527722788	9.87749812829398
C	10.83424880570918	7.59450259964665	9.89421282385780
H	11.82671059772764	7.47660476092866	10.30898808706388
C	10.34870718545711	8.84824227446443	9.59428593736711
H	10.96896812747238	9.71559330598815	9.78015684043638
C	9.07677492611963	9.02833460067324	9.05004422343689
C	7.97070153600551	5.32356784905855	8.90192024850430
H	6.94352222691350	5.59508512594871	8.66133002993679
C	7.94265066724134	4.43775291191035	10.15279057955367
H	8.92012601890956	3.99040092930184	10.33807518686146
H	7.23251539106395	3.62134139607424	10.01320763653910
H	7.65809294300142	4.98909952485331	11.04896049110141
C	8.51109151216768	4.51644087043957	7.71897436342593
H	8.51930344095056	5.11278680917711	6.81006750431273
H	7.90058372345714	3.62438169669854	7.56650014969614
H	9.53394678290510	4.19116537892376	7.91907363859163
C	8.63776714506285	10.45238231652282	8.75311874515191
H	7.64562131472922	10.42344555405329	8.30456652419785
C	8.57961935424952	11.28736642498394	10.03875547710330
H	8.05282305900340	10.77576780935550	10.84368629294376
H	8.08155671818290	12.23983321289017	9.85183787121043
H	9.58643244975354	11.50474289299667	10.39990166216712
C	9.56725265200856	11.14496297071727	7.75436548038008
H	10.59380680188574	11.17583218715741	8.12231286919154
H	9.24170963759532	12.17441102085719	7.59595629479775
H	9.56863730867733	10.63895502174230	6.79154060004945
H	3.62855645209626	6.21244408986299	6.42798757116559
H	3.18597106705044	8.80662560896448	6.75472429771460

***trans*-Int-3**

F	-0.60892576930966	25.00879017226800	6.50234785467791
F	-1.31273169989680	25.14724786303486	8.53766682178472
F	-2.12129606457964	23.64968403920461	7.21361682774305
O	6.04476313931653	18.75740262102799	4.24892791089005
C	6.16929648350856	16.46220570620660	5.47363150235081
H	5.41293904582016	16.96648226090808	6.07012467970432
C	7.55511401710293	17.07560696712686	5.46352859052248
H	7.66125588849846	18.01127035091867	6.00676274201519
C	8.70365328598350	16.18198266254976	5.71739297965154
C	9.99278662621904	16.70265107316133	5.82381623333747
H	10.13744622536096	17.76826369020725	5.68432275603780
C	11.08432572389598	15.88887483543139	6.08283022395865
H	12.07587326673259	16.31978432668837	6.14367639930430
C	10.90355819239279	14.52347115766570	6.25985757668514
H	11.75025453314389	13.87916830103097	6.45880455130573
C	9.62530994364661	13.99453620593993	6.19073868808017
H	9.47001328748719	12.93262912541091	6.34599607468706
C	8.52092017377089	14.80468186019123	5.93131131932767
C	7.16938008017156	14.24557180353946	5.89279246636134
H	7.06009482329197	13.18016002606216	6.06050642387779
C	6.09954818097001	15.00895422122407	5.66243180690854
H	5.11319976954143	14.55676464814544	5.67969586534582
C	5.40408626862643	19.33228099763474	5.34427951852711
H	5.62596854667393	20.40490218230397	5.33798492546476
H	5.78692991895819	18.95095863545732	6.30057703856970
C	3.88297322023123	19.16681444640804	5.29759756564383
H	3.52092196480276	19.55697261759387	4.34684353684064
H	3.64317707012955	18.10118344410014	5.32210515489316
C	3.21155272794373	19.86410883643382	6.43409197167966
H	3.43000343660118	19.48342632301554	7.42880389505379
C	2.39972295311987	20.90751310681719	6.28892957645489
H	2.18793368692487	21.25642538903646	5.28134237197796
C	1.71837387468933	21.65050906776965	7.36192418635297
C	0.75141623928939	22.58963451911708	7.01709426734909
H	0.52380757222675	22.75939948112874	5.97189704376062
C	0.07884364451674	23.31112997606052	7.99341108032034

C	0.35537408418344	23.11138955019356	9.33491374429958
H	-0.16874229088423	23.67561499964591	10.09315907508168
C	1.32449468864527	22.18225605341543	9.68915977638690
H	1.55824138295517	22.02428108846543	10.73367720662249
C	1.99799423946496	21.46456910432815	8.71875739472616
H	2.75879476919969	20.75656288355607	9.02020559852935
C	-0.98603235933670	24.28178068698368	7.56773688175546
B	6.56056628779857	17.39345043120438	4.19851538596221
N	6.11298448241950	17.57796219084362	1.67708785808275
C	6.74405051495817	16.97584214664363	2.66789770506430
C	7.73198905299009	15.97319640563494	2.07351045406496
C	7.30770637160138	15.89008004652450	0.60234494318664
H	8.15621647608597	15.72299086573691	-0.05927222120771
H	6.61848867925604	15.05291826358590	0.47644869916551
C	6.58026787202469	17.18460876337152	0.28593457677799
C	7.62780677527358	14.57458732382358	2.67461832128987
H	8.09239734463876	14.51155002302440	3.64918568815241
H	6.59008244111063	14.25037876698846	2.76182572645510
C	9.16522416938363	16.49609976180539	2.24555867852611
H	9.29503052329290	17.49754041762402	1.83847481271539
H	9.44007648693539	16.52389296628844	3.29681778168839
C	5.42288320397931	16.97214111404172	-0.67905350955664
H	4.74765874070653	16.19270882642060	-0.33693171657370
H	5.83766387410773	16.65596040198487	-1.63638063309443
H	4.85511791565700	17.88739434071388	-0.84348429100688
C	7.52351009145466	18.22444178747667	-0.31975504307637
H	6.99368682268710	19.12587836221740	-0.61101690507343
H	7.96044913478659	17.79406969672431	-1.22084749705833
H	8.33510588368196	18.49652852955180	0.35047949536730
C	4.91936179355978	18.39872209343373	1.79402330036694
C	3.69289119967266	17.73046138800696	1.93508632225076
C	2.52940026397890	18.49414998030777	1.95271050823705
H	1.57426700329075	17.99882056581673	2.06833540342932
C	2.56852238407005	19.86803443928350	1.82884758074878
H	1.65168862471488	20.44330194206555	1.84308022443556
C \	3.78810999288078	20.50464517561909	1.69625053344729
H	3.81661381051328	21.58373419052054	1.61444857041480
C	4.98291130068133	19.79428968432506	1.67591762876860
C	3.55040685732648	16.22207609321404	2.08555636022268
H	4.51160068628880	15.75647198164319	1.87002483714671
C	2.52213784210361	15.63341021838195	1.11427000046242
H	1.50685349950033	15.92572697993403	1.38565156825727
H	2.56569344549412	14.54383266860820	1.14497361399445
H	2.69321667294498	15.95296868619570	0.08636182183487
C	3.18002198574938	15.84598813354268	3.52152523348584
H	3.93728603921893	16.19480287126173	4.21979376279317
H	3.09710682205644	14.76198631320870	3.61837987418366
H	2.22030088487465	16.28448502018569	3.80247309977081
C	6.27202978954072	20.58611736950078	1.54025536438562
H	7.09935963964941	19.88245700489917	1.49971648111388
C	6.27083939063800	21.42695156886207	0.25739079998815
H	5.95644169414567	20.85600648689859	-0.61660020578247
H	7.26784922742596	21.82656065627403	0.06600478142507
H	5.58992968210775	22.27472415653034	0.35121185408601
C	6.52987742839681	21.49485581231518	2.74396318864219
H	5.69534215115903	22.17797561631767	2.91389526225756
H	7.42176245371499	22.09848137554078	2.56588400536698
H	6.68566329774220	20.90165014035156	3.63980566689409
H	8.14608849685677	13.87989773816392	2.01048389049861
H	9.85447770283741	15.82627490913570	1.72891825217077

***cis*-7a**

F	2.69090540013977	6.14232323320994	18.24436141870389
F	4.20094933238195	6.57943794622110	16.77014506226265
F	3.00883486229949	8.17513372625081	17.59354458107109
O	8.39342008469989	9.52610353784823	17.78139724617134
N	8.05131610923885	12.47158327424450	17.17275624015955
C	9.01586965619264	8.41133745273944	18.38371014337916
H	9.86002302923689	8.09609320067655	17.76294403097771
H	8.31248760120602	7.57273623255399	18.42740953756805
C	9.45916969502753	8.80523789280947	19.80504833077929
H	9.36503636463544	7.95013182496842	20.48047294036213
H	10.50842891008677	9.10491175279232	19.81900865882985
C	8.60439996963092	9.99382231780190	20.19867322380044
H	8.99506033071894	10.64117541283671	20.97206970476375
C	7.11981260868708	9.88754127899987	20.04795581594207
H	6.58788436143995	10.61363270398269	20.65030018079093
C	6.37616102111529	8.59680128940154	19.99681721925843
C	5.38568140044432	8.40234203952020	19.04006746757164
H	5.19432788357530	9.18818816411022	18.32200116750268
C	4.65891882568600	7.22228553490157	18.99536263546436
C	4.90742254407341	6.20299274931675	19.90289676983847
H	4.34351754112065	5.28153119545620	19.86009713650603

C	5.88283726768255	6.39019206447252	20.86979962699673
H	6.07780366079435	5.60904820189903	21.59317006754794
C	6.60313436574022	7.57373492348346	20.91766061067794
H	7.35484413718178	7.71379681798414	21.68453458120025
C	3.64011916080578	7.03082225583744	17.91012834156089
C	7.77305837934739	11.96990653655789	18.35804041823219
C	7.16918422495729	13.07350858708187	19.22769096465808
C	7.52529408715233	14.35124103169274	18.45352799993843
H	6.75467428872981	15.11509326477896	18.54796669051969
H	8.45287279297781	14.76543060219695	18.85212839127757
C	7.74769938004837	13.94187449481381	17.00188249155570
C	5.64333352249735	12.88927229743141	19.30787938665964
H	5.18897142833679	12.80713938762714	18.32153576685011
H	5.20632565623807	13.75414384702829	19.80952342152257
H	5.37549952640304	11.99634955175913	19.86608324970019
C	7.75907544301963	13.15628392853516	20.63632511185306
H	7.45131261930332	12.32952837855580	21.27162217348846
H	7.41566646526583	14.08053234986677	21.10522172698808
H	8.84874505946713	13.17756711692277	20.60383460432002
C	6.52094967853371	14.15209437149624	16.11786315831421
H	6.72465039497200	13.82687208650264	15.09932788165133
H	6.29436119431348	15.21757606713747	16.08732705827398
H	5.63991639559030	13.62937445140308	16.48114045874104
C	8.90569732271441	14.71141621389651	16.37848731976792
H	9.80248723622602	14.66321684787492	16.99033020951037
H	8.61122667458510	15.75833561452385	16.30256496314991
H	9.13730236432365	14.35525634941107	15.37506684097619
C	8.61110521756308	11.74205218425399	16.05331203099082
C	10.00474836413844	11.71183033370727	15.90658010545829
C	10.52915793173379	11.10256123495289	14.77315003485701
H	11.60270033457863	11.06272271436639	14.64116680184603
C	9.70656941674536	10.53230804033799	13.81995516347960
H	10.13388767358579	10.06779330109135	12.94053465349636
C	8.33917360385595	10.52964610150591	14.01101057683711
H	7.70280437644161	10.03926109100824	13.28559932465576
C	7.76124247852702	11.11714999135807	15.13210084972251
C	10.96938765007362	12.21901363876474	16.96563971730700
H	10.40513909034402	12.78228921873977	17.70762605620972
C	12.05442656443738	13.13501634435928	16.39636739271663
H	12.73772908814683	12.58416993773272	15.74838292525096
H	12.64769073538003	13.55933859938691	17.20786379037482
H	11.63560238611490	13.95611994488940	15.81483418349622
C	11.60293111880968	11.03457017875274	17.69900985065320
H	10.83200222768927	10.39057948037561	18.11445567753578
H	12.24110073211302	11.38565954599862	18.51182350725511
H	12.21553696655455	10.43804231525443	17.02008652812032
C	6.26611230762620	10.93996346391611	15.33778251551695
H	5.97408302050444	11.48661506322428	16.23354508862755
C	5.94693963281744	9.46160691255611	15.58514651898322
H	6.15472254938021	8.86579580242340	14.69414372450018
H	4.88960522900518	9.33846469073586	15.82528903260856
H	6.54899303401992	9.07194815891136	16.40400359702835
C	5.43272990050298	11.45854855986980	14.16334688585828
H	5.65159329990670	12.49738995305549	13.92010381057940
H	4.37033180941516	11.38263506478830	14.39984718693605
H	5.60977142876878	10.86376861344320	13.26580016679114
B	7.96151157237411	10.48018911882423	18.78678927466770

***trans*-7a (7a')**

F	1.46183160423348	9.16278609672850	20.83294538768257
F	2.53208615378585	8.30768674257265	19.16553670717407
F	2.21569193550392	10.43667594741725	19.26454248849079
O	9.04104427514142	9.67325587426924	17.87587015549598
N	8.04041473646689	12.40064676252545	17.33579718694861
C	9.77145232642040	8.62148424777889	18.46613379053930
H	10.71842989435996	8.49884875039923	17.93262867010801
H	9.22263031340473	7.67746875912614	18.36074355139600
C	9.97520437816193	8.95454138642272	19.95311106906069
H	10.03436239467393	8.04066925108541	20.55126506677575
H	10.90578822029209	9.50799150451120	20.09995348512352
C	8.80040655871606	9.84362271644537	20.30689779910304
H	8.87425755266625	10.43206570114838	21.21007698390461
C	7.45087913590146	9.37057164676741	19.87932497657158
H	7.43493003316588	8.40418130319974	19.38214539980359
C	6.22062753513534	9.56305616734369	20.67064690793615
C	4.98534978928649	9.32609457713834	20.06360035884054
H	4.96345333200699	9.01432387163751	19.02677756470021
C	3.80165918374551	9.52204187156970	20.74948828762107
C	3.80649686090329	9.94875844663738	22.07241351732916
H	2.87833344793053	10.10784163864607	22.60272413342690
C	5.02514976275471	10.14951107234777	22.69792613645988
H	5.05065124434691	10.46009995130456	23.73446336714399
C	6.21425782617257	9.95305329741217	22.00913764200916

H	7.15533207091951	10.10706419725035	22.52143014961203
C	2.50558777422874	9.35016295532951	20.01200946003681
C	7.86806897815708	11.87049802084725	18.53030575223803
C	7.22871103096784	12.92584910443981	19.44003126511761
C	7.46037933057252	14.22359104132031	18.65188618070153
H	6.67091144885073	14.95361143682895	18.82306849385336
H	8.40449239823148	14.67192771536824	18.96740990389915
C	7.57019016561490	13.82931184591468	17.18691679743801
C	5.72355528467383	12.66632757350059	19.62805935271359
H	5.21872302844163	12.39639635597347	18.70221474249330
H	5.25724142536153	13.57334433599636	20.01470819476677
H	5.54515926250166	11.86976746023168	20.34108149027976
C	7.89481792076084	13.03214239182029	20.81069503190707
H	7.65510790129873	12.18279513970906	21.44432967656709
H	7.53228452118310	13.93298907797231	21.31013407605026
H	8.97919762513553	13.10321565137731	20.72037514468156
C	6.24370478487505	13.88760945639052	16.43006868379200
H	6.37742932702840	13.55305408628440	15.40218432198200
H	5.90590777051317	14.92316690133210	16.40131046847695
H	5.46128198723659	13.29124395607863	16.89063672293859
C	8.56114150270106	14.71420316753023	16.44244813253627
H	9.51314421678018	14.79079973704433	16.96006131680427
H	8.13440380588147	15.71563865228704	16.38405831229909
H	8.73223228162803	14.36507895569468	15.42460663949670
C	8.53502341720941	11.70891100308908	16.16410292031838
C	9.88834907334229	11.83741788306097	15.82374171174141
C	10.32126158042289	11.24158493077891	14.64620357561084
H	11.36320108902363	11.32084943362602	14.36376412229965
C	9.45178764384032	10.53034605311769	13.83953231217806
H	9.80850740876989	10.07700702657541	12.92352995488904
C	8.13498214823265	10.37338710645211	14.22359405109714
H	7.47067788638495	9.77611737742779	13.61213962915075
C	7.64971740932110	10.94324901977515	15.39683461388347
C	10.91930308529179	12.50205723548106	16.72219572075136
H	10.39433022048995	13.01998787954276	17.52341620583277
C	11.78358647489461	13.52186479229167	15.97697025326750
H	12.44136420419356	13.03108087770592	15.25802072645872
H	12.41835172730141	14.05988597942012	16.68280358175621
H	11.18413478863703	14.25150228106975	15.43324423387840
C	11.80761616586645	11.44927805808499	17.39129199747734
H	11.20447968066180	10.75562219456533	17.97159762021725
H	12.52868697543174	11.93136789465519	18.05381955324503
H	12.36403279105954	10.87746104030112	16.64587822921165
C	6.23100184236055	10.60522818363001	15.82539422374252
H	5.99299564541383	11.17623376177481	16.72084722875769
C	6.13638202339480	9.12281977640293	16.20311940973836
H	6.32274005788973	8.48841911571010	15.33427110084960
H	5.13592627086808	8.89333019366077	16.57495712946940
H	6.86717461355472	8.87330092965071	16.97054713169287
C	5.18778641946026	10.94421325178330	14.75896561903347
H	5.24449447356771	11.98559187994311	14.44363485761741
H	4.18510551827426	10.76258451618492	15.14885253830812
H	5.31208973503286	10.32074143741724	13.87214474713230
B	8.28567529108378	10.40292208585811	18.88976405605628

borylene^{cis}

F	2.15949850593783	9.37839365299188	18.38549841195365
F	3.06126625411666	8.60423324959119	16.58683189329708
F	3.83707672170334	10.39938080627474	17.49868512930541
O	9.53125008680618	9.61192379288468	18.98092906746257
N	8.06348053338657	12.33847877714296	16.95847349742015
C	9.03250544360300	8.28417635359207	19.21574584475019
H	9.70549441989999	7.60955046654582	18.68876838190056
H	8.03510108472117	8.19754886169507	18.78512398128089
C	9.02829876327339	7.96875253215332	20.70135937731616
H	8.79280860247381	6.90802556920338	20.80796171495908
H	10.04156917060481	8.09631055618210	21.08261287077016
C	8.08042329856710	8.82017396704413	21.51006686229605
H	8.53504537875494	9.43259554900180	22.28053975204167
C	6.76414321448469	8.93651534303596	21.35419803894955
H	6.24295613666527	9.64908346382583	21.98524947556922
C	5.91685577079797	8.23581315201216	20.36321980060453
C	5.06097473751840	8.97990151324208	19.55549109762957
H	5.02927332756527	10.05589741860094	19.66141136386719
C	4.27349551155273	8.35474113732463	18.60282089919222
C	4.30870420164649	6.97654222920855	18.44802292441581
H	3.69231162002321	6.49453944776626	17.70207308602986
C	5.14260313870730	6.22820393421225	19.26232824866515
H	5.17145136454731	5.15185922672080	19.15777907221050
C	5.93635870010341	6.85127014791762	20.21281681759926
H	6.57154668891017	6.25379227738035	20.85366463208947
C	3.33778547655728	9.18197640758985	17.76653385894423
C	8.23679089910868	11.88177467896337	18.28301222808654

C	7.75656868987440	12.96693456041537	19.24994118676542
C	7.50531996682978	14.15212005793489	18.29000893706108
H	6.67621049841057	14.77753043482623	18.62436531805427
H	8.39839598605976	14.78007569115130	18.25578456727253
C	7.26605435061921	13.56885454090170	16.88582221433206
C	6.48275301631925	12.57789510750194	20.00760849758579
H	5.67769454293219	12.29618281209754	19.33070098973153
H	6.13308756131877	13.41045169667751	20.62357618845159
H	6.67914337136691	11.72891570209076	20.66381266930135
C	8.83868661556386	13.31443697774920	20.27654438419684
H	9.04632830867844	12.45933014247695	20.92511339325676
H	8.52183011681483	14.14476410683615	20.91310246919284
H	9.76894278570566	13.59438036408247	19.78103685726292
C	5.77373138471576	13.30077697528844	16.63714231925369
H	5.61128396237693	12.94035109724155	15.62176798150791
H	5.19752142281214	14.21965082945214	16.76157781214561
H	5.37767773540732	12.55490694854214	17.32330253979404
C	7.76581685525147	14.50630948498620	15.79185488309367
H	8.80159561994673	14.79766442389713	15.95637420552904
H	7.15610797153359	15.41054472281188	15.77584827006801
H	7.69163051514093	14.03522612600946	14.81006689351383
C	8.77260493334832	11.71700825218037	15.89963042313573
C	10.12703396496423	12.04151840973445	15.67492174327035
C	10.80291216337810	11.42333218632277	14.63002520336814
H	11.84074960566456	11.67281732572611	14.44471696504668
C	10.17549634764749	10.49130576847177	13.82136777906274
H	10.71556712594375	10.02780062777938	13.00521342423313
C	8.86402209955018	10.14068509626560	14.07522077814214
H	8.38509520613238	9.38888764063124	13.45920025137605
C	8.15193181013989	10.72532227748025	15.11984546221946
C	10.88221207734653	13.01250191454632	16.56308201237525
H	10.15682456010584	13.46793586164497	17.23506727136644
C	11.56139479273829	14.12862107811010	15.76708646497839
H	12.36457057625771	13.73982018098014	15.13836970326733
H	12.00188539996189	14.86132453667772	16.44554593683399
H	10.85537722076697	14.64770273959255	15.11783636657085
C	11.90634544903325	12.27189962370966	17.42817750713682
H	11.42684227630001	11.49228623047192	18.02025896891177
H	12.39957009874923	12.96502477278463	18.11260741450780
H	12.67708265375062	11.80574913250025	16.81014464441788
C	6.74634656835960	10.23695423884365	15.41066825797475
H	6.38907149595675	10.78432122442402	16.28074137123263
C	6.74670414070956	8.75005817286847	15.77357431015982
H	7.10492601844540	8.13444060382902	14.94603143957365
H	5.73780276307814	8.41779740681498	16.02129265890272
H	7.38577015854637	8.55824626657471	16.63643745211455
C	5.79225620250404	10.50045111608588	14.24459399789318
H	5.80675663843483	11.54876083606684	13.94423990277383
H	4.76980918833419	10.23956532840337	14.52320003967439
H	6.06347421815109	9.90345182945053	13.37115089245954
B	8.76012391598278	10.62771200594984	18.64617045103679

borylene*trans*

F	1.30070641380041	10.02573882209617	20.09458383686224
F	2.21089293867886	8.37859874949631	19.03770288549450
F	2.73865301201717	10.40547415656699	18.53193134564607
O	10.20807165444184	10.13749025393393	19.12167968073582
N	8.18309431798647	12.60733207840464	17.17624992735449
C	9.91241408998653	8.73421861367773	18.97767252818343
H	10.81694306410494	8.27848695577573	18.57802531313499
H	9.10400470650512	8.61622508606086	18.25467999266160
C	9.53699844003720	8.10666189168334	20.31034782781592
H	9.40282871704509	7.03750279498752	20.13289867753376
H	10.36972178784904	8.22280915023319	21.00624879407138
C	8.28422399383595	8.70037379198023	20.88106828614590
H	8.39253732806696	9.61826372538059	21.45118500493168
C	7.06761916596546	8.21710451958504	20.65557679312152
H	6.95323784538569	7.32818511251291	20.04058505931544
C	5.82318270655339	8.87081860759079	21.09187387384717
C	4.71911633083309	8.84500943988362	20.24749935200449
H	4.77020995011415	8.29115998272541	19.31865579123174
C	3.57378458140405	9.55821650622727	20.56460339351094
C	3.49455411869929	10.28047559453325	21.74454133193930
H	2.59855636145684	10.83365731018323	21.98788799979326
C	4.57719184280003	10.27497143125980	22.61198373866547
H	4.51984172242889	10.82047215566417	23.54438538120436
C	5.72994540426873	9.58009137261566	22.28951044735482
H	6.56941804640227	9.58471557795800	22.97309985878560
C	2.45154469902121	9.58932137310771	19.56655256296203
C	8.44164067813163	12.10401052114302	18.47536814087390
C	7.57811604684331	12.89610721643048	19.44416601818013
C	7.49997380249985	14.24466881381229	18.70086881015926
H	6.59765590585824	14.80365005389501	18.95461265785293

H	8.35852545654699	14.85317902737476	18.99272703970958
C	7.58457291899914	13.95632264791200	17.18751061427916
C	6.18832659600154	12.26914068231818	19.61280808479678
H	5.70678528146269	12.07131944123052	18.65753670670212
H	5.53360655672254	12.92833405161782	20.18887258119583
H	6.26548056940676	11.32029579564080	20.14234469715920
C	8.21245305812862	13.05026944432107	20.82313262818076
H	8.32382337225069	12.07745383196391	21.31042470423419
H	7.58544804980174	13.66661374579002	21.47140709351521
H	9.19845082097254	13.51041362473829	20.75342395211814
C	6.20857310239788	13.98784624253175	16.50899106955134
H	6.28843936416282	13.65363794070739	15.47303938692018
H	5.82314611361525	15.00846990547572	16.49897837153316
H	5.47875386480323	13.36067133981734	17.01653298563035
C	8.44769463249735	15.01168279284602	16.49171922013485
H	9.43159188077441	15.09130339870255	16.95007161808008
H	7.96431547561986	15.98783034580836	16.56077530804520
H	8.57071906105843	14.77610217951655	15.43320207906085
C	8.60266866271367	11.88094093899160	16.02894634311668
C	9.91601604421847	12.00389380245860	15.53114105348095
C	10.28246701520509	11.27606677292578	14.40151395894357
H	11.28641782625963	11.37829442500838	14.00747272056850
C	9.39759300856019	10.41535671097993	13.78225763474181
H	9.70099833389805	9.86021061098668	12.90342521419653
C	8.12921851349457	10.24980823809917	14.30805788957069
H	7.44785805689020	9.54811356337225	13.84212523603216
C	7.71645601370566	10.95952195653632	15.42963766936618
C	10.97748873228274	12.85633907679809	16.20561201122076
H	10.51346825436005	13.33379997420730	17.06637449495642
C	11.52843924347278	13.94065535493277	15.27632201078594
H	12.08127449537358	13.50013334386749	14.44420447915046
H	12.21582754706089	14.59147063300692	15.81984374836382
H	10.73570511566959	14.55905341095839	14.85778131475285
C	12.12842469786220	11.99125341039128	16.73026454230596
H	11.77254838955488	11.21969422657626	17.41221757728475
H	12.84840769904760	12.61010771587271	17.26884886003583
H	12.65873410359967	11.49964466408094	15.91192257447447
C	6.34276332244580	10.67261188300064	16.00453825253236
H	6.17311135238654	11.39780610685240	16.79287756004940
C	6.29493420488843	9.28744232637128	16.65436397597066
H	6.51248369998731	8.49822869732372	15.93124158218170
H	5.30165222727452	9.10152204722025	17.06767264002308
H	7.01690572066894	9.21770175534683	17.46917249432400
C	5.22474918788773	10.82181373257861	14.97252281528970
H	5.25677548699122	11.79746396227793	14.48667090756343
H	4.25202670923126	10.71569044836089	15.45596277603483
H	5.29023798488187	10.05904114822068	14.19414072196817
B	9.35279050220968	11.09080188495200	18.81006718982540

borylene^{cis} (triplet)

F	2.28475214777033	9.36792386536018	18.53242713035750
F	3.07522871519017	8.58875596135766	16.68255796642368
F	3.94229911853939	10.35282858999654	17.57285154962306
O	8.81845481876828	9.90625252841888	19.66179939183874
N	8.13262734453033	12.37760905306211	16.89239500587501
C	9.03933025492918	8.51873693533630	19.86092565691983
H	10.02674922947853	8.24291501372378	19.48184989337033
H	8.29794345195225	7.95167471271050	19.29696177258571
C	8.96996051680320	8.20772872502569	21.34855059037333
H	9.06297750843297	7.12467519244883	21.47254741582197
H	9.85072861693686	8.63980355451429	21.82562072044244
C	7.75507194618715	8.72249166924892	22.08120893760355
H	7.97536106494787	9.22014037196322	23.01963198844945
C	6.47141859463955	8.69302165890252	21.73299238308465
H	5.76806416001232	9.19428505595703	22.39074659780922
C	5.86659520281388	8.09067781156619	20.52663823250743
C	4.96285342196098	8.83150901601782	19.77285631368216
H	4.72217056008923	9.84443950012297	20.07024575653644
C	4.38264219931495	8.29083448565667	18.63535556664326
C	4.66948117285592	6.99322548772548	18.24332136855849
H	4.21147082029399	6.57462816508453	17.35849740676667
C	5.55255781257841	6.24015660839023	19.00217655175068
H	5.77888879188215	5.22335340628499	18.71039293432426
C	6.14377660344689	6.78264167368598	20.13066784366594
H	6.82473730928943	6.18298161338775	20.72125216710308
C	3.42297348688870	9.14169643175011	17.85317058721580
C	8.22507853332874	11.82945988186338	18.13685175400519
C	7.72083019007472	12.82723651895285	19.16904552373404
C	7.49908730199618	14.10092362516217	18.32719343292424
H	6.61923283696245	14.66049533923631	18.64556752783362
H	8.36387890099052	14.75856247718399	18.43568664565077
C	7.39079094262332	13.66459002653543	16.85895126163909
C	6.43296082156361	12.32813664009101	19.83508868589078

H	5.64984227470487	12.12491508873040	19.10468758649726
H	6.05664911264933	13.07214602371965	20.54109277591285
H	6.63047649932460	11.40594772324123	20.38300810012966
C	8.77374223895246	13.07527004537575	20.25419505665569
H	8.94883072749008	12.17253637042983	20.83879224371640
H	8.43949635699148	13.86507787725296	20.93152476679824
H	9.72222800318816	13.38443659360465	19.81126806817312
C	5.93362800948293	13.45536359725962	16.43588903887396
H	5.87369751893432	13.12386351080824	15.39992342449476
H	5.39448126352508	14.40012994023521	16.51623952068276
H	5.42843172272137	12.72145415985756	17.06137729483416
C	8.02348582745532	14.67723390141131	15.91144727932380
H	9.04583663898657	14.91488777680996	16.19770512135112
H	7.44350844834743	15.60006678825764	15.94045451018152
H	8.02383185363493	14.31311869472221	14.88325111875620
C	8.73361481705281	11.78099464451346	15.73928960578289
C	10.06911577255734	12.10344592910023	15.43992734319547
C	10.63404672067876	11.57994683263349	14.28360097867794
H	11.66006010415950	11.82088744319014	14.03559364437804
C	9.91199627250755	10.74476169215602	13.44969129252957
H	10.36539293239955	10.35247743503621	12.54816071689914
C	8.62161987677979	10.38821172169755	13.78917649561683
H	8.07738981467714	9.69733538152019	13.15729755695272
C	8.01452838124153	10.88057256685368	14.94147498392730
C	10.93622197966308	12.92791292642722	16.37551952204713
H	10.28614627494823	13.39080878769920	17.11617082067421
C	11.69832830561344	14.04171330258330	15.65676910870695
H	12.45027102934408	13.63951542598373	14.97594967363132
H	12.21810913171166	14.66874405332384	16.38290059191021
H	11.02942141278900	14.67724956068224	15.07518987911360
C	11.90223543436481	12.01437543782321	17.13644961481895
H	11.35750552534147	11.23949002928672	17.67824758245897
H	12.48673125762797	12.59076069760921	17.85595379610473
H	12.59644694784435	11.52369210652139	16.45082097525024
C	6.64934798234429	10.34515816494500	15.32957673102618
H	6.31771439133459	10.87844538988096	16.21748676653915
C	6.75744757885125	8.86568985485581	15.70959307358341
H	7.11980026116448	8.26734082880941	14.87116094915836
H	5.78123184703769	8.47701260253275	16.00048607536685
H	7.44510998371789	8.73219074330250	16.54756971262022
C	5.60127878323596	10.54014096488890	14.23319178289448
H	5.54206007613434	11.57948394277245	13.90855017888298
H	4.61774905502510	10.24137777085673	14.59874601840209
H	5.82608555162133	9.93182912106074	13.35502850427231
B	8.72986160376570	10.43965495300763	18.40653355717738

borylene^{trans} (triplet)

F	0.96262613255827	10.68158201415355	19.25157333889576
F	1.93512897848941	9.29996076917290	17.90795589927146
F	2.37945836725811	11.40593692688278	17.79769987106318
O	10.43827523317609	9.86392331425218	19.13955909201881
N	8.17421059385493	12.06780101545455	17.73290071746012
C	9.78717550807451	8.78541251441744	18.48043617059035
H	10.56840782578109	8.16919624871295	18.03535021678541
H	9.15244845912732	9.16481578796877	17.68018352462672
C	8.97213011752075	7.95445770676241	19.47072061676977
H	8.61435718380006	7.06002871992879	18.95554324042480
H	9.64116851153203	7.62808246533963	20.27097748584123
C	7.81604394717953	8.71884034182304	20.03457612156956
H	8.06031703805080	9.58931598076763	20.63853631055947
C	6.54209914050618	8.44669768773310	19.77276248657062
H	6.30339407920410	7.60173702581204	19.13441072902376
C	5.39977893577091	9.27576382664325	20.18999558336015
C	4.30309316389792	9.38135845043464	19.33732885055389
H	4.28704289589769	8.81307760027041	18.41628376088291
C	3.24966989920484	10.22583356299718	19.64315522675061
C	3.25502925392812	10.96639506721120	20.81651228488726
H	2.42916493972655	11.62311388494748	21.05253829986445
C	4.32621116593231	10.84038997407834	21.68660483780123
H	4.33416874810891	11.39921092317925	22.61292983467004
C	5.38702932961363	10.00383291922807	21.37838966545421
H	6.21522038851524	9.91153844299816	22.06930048626422
C	2.12993549369539	10.39633137869674	18.65672595013540
C	8.89346506688092	12.04860032999846	18.91008323936209
C	8.50961050278715	13.25319959458459	19.77001487722210
C	7.67050261356864	14.10032690834351	18.80382479544035
H	6.86512971569347	14.63622973910628	19.30594078932642
H	8.31114519095184	14.84476342387418	18.32588306314663
C	7.13105017690802	13.14571508349742	17.73023410160508
C	7.72455400743149	12.82761991546842	21.01715011407186
H	6.81657107365845	12.28302978993794	20.76720488010196
H	7.44388186550679	13.70335921379214	21.60705094154189
H	8.34224019296024	12.17839159918981	21.64104830915907

C	9.74650625626744	14.02672391942932	20.23891758222247
H	10.36743687423166	13.40275057048654	20.88302108322427
H	9.44163030231702	14.91158591268593	20.80306665843578
H	10.35825193218171	14.35548047278723	19.39791934259242
C	5.75888817918828	12.59813290954192	18.13880302606795
H	5.29823971329427	12.01473729756581	17.34733543063854
H	5.08858628192292	13.42818085693624	18.36541002349635
H	5.82711457361057	11.96588126045610	19.02236242440708
C	7.02560844276832	13.84306155091451	16.38086672406953
H	7.97617758711838	14.28639269818736	16.08769508508622
H	6.28769513863418	14.64257689222460	16.45426350822374
H	6.70745399166468	13.16443774950323	15.59059889835501
C	8.68628680532326	11.49433663123806	16.51790015938984
C	9.89611153566499	11.99322592576222	15.99114194633736
C	10.40978944445095	11.42366138233282	14.83178902818140
H	11.34713253012442	11.79159804657765	14.43398247004385
C	9.73880060544175	10.41852078739855	14.16457291495208
H	10.14767519399902	9.99679254177368	13.25535472550193
C	8.53625154452003	9.96258298501834	14.66520446606246
H	8.00779150241876	9.18136528570163	14.13489617573995
C	7.99485944207772	10.46947743353747	15.84315825391306
C	10.66643411062597	13.15372785589969	16.59824063760280
H	10.07706887640495	13.56026883805522	17.41473550885163
C	10.87834578706391	14.28542483058162	15.58821627820488
H	11.54856318308859	13.98579575338584	14.78102204345153
H	11.32645544480244	15.14738146924378	16.08518415384963
H	9.93833076115320	14.60523513180138	15.13708006125717
C	12.00746314678957	12.69952477451533	17.17783981936321
H	11.87562209430656	11.91604465313706	17.92428033723500
H	12.51877011457486	13.53801858275133	17.65385756732415
H	12.66013118147159	12.31052682266627	16.39350403523305
C	6.68172479567342	9.87335581945711	16.32342388609076
H	6.52170349629552	10.20678283878874	17.34901047825959
C	6.70146146432455	8.34191346686764	16.32746418614107
H	6.77636749435477	7.94014866180198	15.31655523109777
H	5.77380441632135	7.95943378335688	16.75543307000126
H	7.52999725447387	7.94163360238811	16.90890958521594
C	5.50869047663918	10.34116079863473	15.45394289847794
H	5.46813739607837	11.42439131057990	15.35443605193722
H	4.56006591833923	10.00576294967904	15.87805624605632
H	5.59309500981509	9.92416869446958	14.44839358970781
B	9.92357196375639	11.07910702449174	19.44751239595562

cis-TS-1

F	5.03846501161034	-4.12233801224431	-3.68220957275963
F	2.92488148262854	-3.99461166336244	-3.29605351337264
F	3.71183087366858	-3.36502385786005	-5.20438993655318
O	1.29823675421814	0.08896299342241	0.64281705199492
C	0.54449134929444	1.34050493735338	-1.47878517915971
H	0.49941373560030	2.31651297354474	-0.99992921592109
C	0.55825719574768	1.39864049446084	-2.81607991459751
H	0.52653672239458	2.39033729896846	-3.26177366332477
C	0.63816499645915	0.35343472574235	-3.84289871817441
C	0.78281833125392	0.83869531891574	-5.15124103487517
H	0.81916152306995	1.91214583710295	-5.29377114303113
C	0.88626604370888	0.01224631290704	-6.25111450733478
H	1.00465972860617	0.43546252886012	-7.24018384766740
C	0.83076738974317	-1.36218337363689	-6.06874889846463
H	0.90837079445948	-2.03667060777905	-6.91145561207809
C	0.67638896954662	-1.86398923719261	-4.79342970795723
H	0.63877064056911	-2.93677372281722	-4.65082344076717
C	0.58644723647403	-1.04665608535295	-3.65704658897888
C	0.44227795770097	-1.77690467192243	-2.39369137799485
H	0.35832026019050	-2.84785993586916	-2.55970409103624
C	0.41223274109356	-1.35578810879739	-1.12545758393469
H	0.30557818083656	-2.15023378469519	-0.38985478791663
C	1.87663957580277	1.23084808453609	1.22454493696514
H	1.95945040548869	1.03505514457031	2.29691637902228
H	1.23515871748283	2.10375114345628	1.10382932054860
C	3.25025869377447	1.51833869130961	0.62197474721756
H	3.12307323476531	1.68626190761980	-0.44599982138784
H	3.62582207003318	2.44885951518343	1.05745763482854
C	4.21275938189364	0.40731997661266	0.90255605668462
H	4.45890319359206	0.27621360017631	1.95315485216001
C	4.73231379231868	-0.47705408798020	0.05470036199040
H	5.34454850344339	-1.26910115311059	0.47479276887647
C	4.53047866756348	-0.56964617162253	-1.40653118973680
C	4.33600491301391	-1.82618246248212	-1.97183890326475
H	4.32558520515080	-2.70140289546245	-1.33349950083056
C	4.14225354961891	-1.96864635694522	-3.33762343737515
C	4.14228972751952	-0.86269344913187	-4.17098438864863
H	3.97460103691351	-0.97551901405718	-5.23240854963079
C	4.35663255438451	0.39121980430631	-3.62037302540582

H	4.36901121374436	1.26230714033393	-4.26182178213014
C	4.55788747996324	0.53598785785037	-2.25815214978185
H	4.75715750848927	1.51833908701983	-1.85184983118797
C	3.95131651892664	-3.35337383482705	-3.88626718089429
B	0.61613887922025	0.08212672689119	-0.56084837042567
N	-2.23494991955122	0.15291693813910	1.55673998723676
C	-1.80802715615741	0.28545311293474	0.33069529107758
C	-3.02285997252918	0.19828989987370	-0.57649025610996
C	-4.14545690333066	-0.37836970493008	0.30485673371227
H	-5.11479445007889	0.07707933218896	0.10064348632855
H	-4.24023153839560	-1.44852928611740	0.11400869487686
C	-3.71458379648393	-0.15396161926608	1.75606762666316
C	-2.79579450536974	-0.65510551296698	-1.81970077953430
H	-2.00772531036606	-0.23773799908187	-2.44474717143150
H	-2.51058574910295	-1.67354601540458	-1.55699892942635
C	-3.31091224867632	1.64166634424946	-1.02408027927900
H	-3.52911163610868	2.30274067797395	-0.18490754560209
H	-2.45353720551895	2.05049846282743	-1.55958479435939
C	-3.91011197727823	-1.40059463075206	2.60543592627478
H	-3.40001075996687	-2.26206668908828	2.17975547918796
H	-4.97540111841888	-1.62864070358977	2.65029084997801
H	-3.56005752671713	-1.24379869229062	3.62562877049838
C	-4.46898764341185	0.99176816201835	2.42721931246778
H	-4.03645113663725	1.23625232050074	3.39723242695058
H	-5.49700281385002	0.67080508355754	2.59592038512481
H	-4.50346895474489	1.89140135998088	1.81692285836114
C	-1.35800362872975	0.39264212662280	2.68857551529712
C	-0.65383007445771	-0.66360296995793	3.28872904951588
C	0.15721117117627	-0.36959739343732	4.38146952660843
H	0.70523490111885	-1.17120759980097	4.85977759207917
C	0.29712415276488	0.91949516301568	4.85572585227712
H	0.93791774245236	1.12134443663697	5.70454317193360
C	-0.36998111865638	1.95179561617124	4.22566294239694
H	-0.23655535698751	2.96583656133695	4.58000899527573
C	-1.20408816448158	1.71558989244962	3.13844077096868
C	-0.66600849716069	-2.10263951599218	2.79540496028001
H	-1.28737347413765	-2.14966098382153	1.90139207588659
C	-1.22686781675738	-3.06971606213515	3.84398007301738
H	-0.56548096141043	-3.11929241319308	4.71091453117517
H	-1.29492045579763	-4.07552805368424	3.42602500068180
H	-2.21437787254768	-2.78259411485758	4.19842267769613
C	0.74186652760386	-2.56440605776437	2.40024127852002
H	1.20786580934784	-1.86543621711283	1.71045601498205
H	0.69126397446131	-3.54791842531778	1.92905428660420
H	1.38218669607231	-2.65621242196932	3.27994258453881
C	-1.87040141666676	2.91421214662693	2.47886549798497
H	-2.61229197576722	2.54612191795755	1.78003923933441
C	-2.59124919918727	3.80815176425166	3.49042765941045
H	-3.26743242261381	3.23720933657376	4.12704026949411
H	-3.17625027864760	4.56552499168601	2.96646510765333
H	-1.88631341203537	4.33117827904856	4.13847268309697
C	-0.87858739542991	3.73608683226245	1.65452432832560
H	-0.03121774985033	4.06637798094605	2.25868847919865
H	-1.37127432445023	4.62169661431836	1.24948840235720
H	-0.49903801687972	3.15355490173096	0.81652549566516
H	-4.17352884306054	1.65145951740085	-1.69303061400748
H	-3.71615893856804	-0.69790630474447	-2.40708516493204

***trans*-TS-1**

F	-4.11568003434129	6.31060746975792	-0.06714709399375
F	-5.41279015120191	7.37651555501114	1.28832913741313
F	-6.15124360081598	5.66214366189233	0.20558997880282
O	-0.39359037880473	-1.59658256228956	1.33260300903629
C	2.07593012208763	-1.23527322550831	1.97031068218239
H	2.01076256139675	-0.15439820490192	2.07484441043732
C	3.14589703990310	-1.76927908096575	2.57152061467794
H	3.80771668920358	-1.08016595508740	3.09151080316415
C	3.62417758338848	-3.15350910628269	2.68234778070427
C	4.75782705561142	-3.30953997127925	3.49459340501433
H	5.17276234606915	-2.42488455205738	3.96255332833833
C	5.35770833044394	-4.53082075810040	3.72099332887031
H	6.22881606828588	-4.59850946126027	4.35962279630315
C	4.82992367336328	-5.66322773041133	3.11737023006699
H	5.27966608658180	-6.63499605608494	3.27455334694988
C	3.72140262889757	-5.53580843346465	2.30642534662612
H	3.31287514986894	-6.41890514341424	1.83005204808181
C	3.08814894993308	-4.30718716114723	2.06476626679651
C	1.93590273230562	-4.38432844041858	1.15919582188171
H	1.79363868879213	-5.39377407273555	0.78009175437207
C	1.07505976240858	-3.45246967967471	0.73876644778463
H	0.31209858424745	-3.82091333870490	0.05571375243860
C	-0.80947031392204	-0.36300455445715	1.85637153136424
H	-0.75747245836816	0.39753677852823	1.07527891795998

H	-0.16190709462740	-0.04481695899746	2.68065854126512
C	-2.24901595379575	-0.46138498621592	2.34533689403334
H	-2.88573506774088	-0.77157460555913	1.51452602107924
H	-2.31010007602029	-1.22936047742325	3.12037359333225
C	-2.68915018657554	0.86706169525862	2.87373662484605
H	-2.21913154149670	1.19991792667496	3.79585320979416
C	-3.52602379357730	1.67490889021514	2.23183810854185
H	-3.94585881899404	1.33765625189648	1.28909290259342
C	-3.88936997635470	3.04395947498857	2.62966471243750
C	-4.33454627773222	3.92667350703444	1.65193076009052
H	-4.43250566754910	3.58321928727130	0.62894084070458
C	-4.63979190197904	5.24290039921685	1.96845165778837
C	-4.52227227645837	5.70151022697084	3.26897806655385
H	-4.76438176802596	6.72588901709845	3.51330192384362
C	-4.09752227240397	4.82123440918291	4.25580725555665
H	-4.01523670995540	5.16372573991362	5.27878790674068
C	-3.78669618239404	3.51096436164837	3.94248943743870
H	-3.47169514136407	2.83605170004630	4.72793873663165
C	-5.08207739137640	6.15254614494604	0.85788924441717
B	0.93922444673201	-1.95968370180878	1.17996688960437
N	0.85565647569016	-0.34964904515125	-1.95759607747862
C	1.55869857213719	-0.92107980133788	-1.01759203688022
C	2.95308808023387	-1.14465346867418	-1.58786209704805
C	2.78945051446763	-1.00137593648571	-3.10999704096789
H	3.64635937021203	-0.51648818308578	-3.57799700101447
H	2.68949329122998	-1.99244420192350	-3.55565611947880
C	1.50212860490188	-0.20897700320743	-3.33211626663895
C	3.57324351537057	-2.49088265287231	-1.23160817846417
H	3.73477624983032	-2.58362092738041	-0.15977132629242
H	2.94033465702603	-3.31854687311364	-1.55068722654318
C	3.83094788170721	-0.02919382913720	-0.99313729229123
H	3.46880476980593	0.96669735452939	-1.24915429679094
H	3.86253966632533	-0.10700817795655	0.09312856208165
C	0.63103342177309	-0.83421456642633	-4.41208215739632
H	0.42700402042931	-1.88258065863410	-4.20584348411264
H	1.16189192702447	-0.77636252489716	-5.36265486964970
H	-0.31264108735613	-0.29998379133721	-4.52401837734748
C	1.76131586893533	1.24411373665078	-3.72040811663746
H	0.83626821279905	1.81884197109986	-3.74508273416094
H	2.18362516364500	1.25649477152426	-4.72522646551033
H	2.47016458329967	1.73919623567676	-3.06073455617796
C	-0.46971090082473	0.19089611207410	-1.72659942971993
C	-1.60401630810958	-0.61148541569418	-1.92473250850134
C	-2.85584213425096	-0.02548313082233	-1.75823148794810
H	-3.74326130309188	-0.62578709618731	-1.91247580238719
C	-2.99085707536592	1.29820554819398	-1.39110944989482
H	-3.97593886580117	1.73312046536159	-1.27776596792722
C	-1.86325831536859	2.05703016554266	-1.14519333087812
H	-1.97493176531354	3.08193128445983	-0.81485563859988
C	-0.58730248467681	1.52648157865472	-1.30096473340582
C	-1.56014934953018	-2.09569421897533	-2.25344112061656
H	-0.51626289860770	-2.39567564810406	-2.34046202151906
C	-2.27910052265481	-2.42438883817231	-3.56578854046362
H	-3.35166971049109	-2.24401635892346	-3.47522777218079
H	-2.14488659474019	-3.47940807954426	-3.81000540135912
H	-1.91388941432839	-1.83318084981720	-4.40336444179371
C	-2.17828624002102	-2.91885459811826	-1.11821414671984
H	-1.71306370786612	-2.68399219203153	-0.16372221814499
H	-2.05353460396539	-3.98466296134289	-1.31896178716061
H	-3.24943374258352	-2.72272572115100	-1.03508466990243
C	0.59859308249754	2.41449550978540	-0.95014447954361
H	1.50375637154533	1.92298306814735	-1.29006529537355
C	0.52232378646228	3.78294414322128	-1.63205855941608
H	0.34529823785736	3.69723223659404	-2.70403178088423
H	1.45828672023933	4.32360278971339	-1.48455406485105
H	-0.27691186669266	4.39483192609125	-1.21105018722404
C	0.74641014598608	2.59384946567127	0.56343177241820
H	-0.17309702596665	2.97634594413417	1.01217794696965
H	1.54785800919572	3.30306646213269	0.77648262142658
H	0.99464156496491	1.65233376853989	1.04955109050503
H	4.53926819660089	-2.58669791337232	-1.73308137872005
H	4.84851149176849	-0.12620415322324	-1.37618102995001

cis-TS-2

F	1.12930301288001	-4.80215911643751	-2.47428242404880
F	0.55714830299309	-3.35114731625588	-3.96495283862384
F	2.02713053635002	-4.85258989684361	-4.43546144755953
O	1.52715127328433	-0.20589227874370	0.56413772546697
C	0.74010574891343	1.74661103348812	-0.99440438039508
H	1.57471747644687	2.32350510175158	-0.59967765404815
C	-0.19383607792296	2.50304712413421	-1.72172602309283
H	-0.22762921688472	3.56810585299115	-1.51530936586478
C	-0.96542401658687	2.07146454850568	-2.79345209804202

C	-2.03712710455669	2.88320886452635	-3.30147695581911
H	-2.34558347399421	3.74177984804256	-2.71451004946522
C	-2.66705168727612	2.59341719756493	-4.46466930824986
H	-3.48768806546329	3.20970999789588	-4.81019446116784
C	-2.23158420828295	1.48736429560473	-5.25865611141723
H	-2.72482169892376	1.27598812903659	-6.19901999020182
C	-1.19632441723688	0.72082143065841	-4.84042456176488
H	-0.85549892944337	-0.11262559049858	-5.44520587380281
C	-0.54633692343961	0.94204890823488	-3.58136445884004
C	0.55531060860885	0.19112556728114	-3.20385795011256
H	1.07238743965165	-0.35328690073314	-3.98878517226868
C	1.12628215077237	0.15088293204235	-1.92395029638822
H	2.17110530121195	-0.15405470538403	-1.91619557158971
C	2.49100067570570	0.58071210346200	1.19595805208448
H	2.50315376442494	0.32219770950220	2.26136276844627
H	2.24880650027043	1.64582682649333	1.14132112121615
C	3.89137281820630	0.34240100812521	0.61544210097215
H	3.85913876755184	0.50513467671201	-0.46140645277629
H	4.57205274140393	1.08527188765070	1.04071217178283
C	4.37705088789921	-1.03128410643596	0.96173622377382
H	4.64236987488727	-1.17033339413739	2.00666623551208
C	4.42577638018086	-2.10735286699852	0.18239752447837
H	4.70897533826537	-3.05399256184959	0.63234030965472
C	4.06532413811032	-2.16528450723317	-1.25176592936955
C	3.07008081099554	-3.04810532802829	-1.67004137987407
H	2.57452994537466	-3.67547384681484	-0.94135636135064
C	2.69782790295310	-3.10368582351560	-3.00078089872772
C	3.31673726344912	-2.29336471937122	-3.94557170974774
H	3.01866770044814	-2.34276989975865	-4.98463281140831
C	4.31637251850646	-1.42854823919857	-3.54103048662711
H	4.80863591472289	-0.79626856199874	-4.26772311702354
C	4.68963235820972	-1.36796019911672	-2.20481093185601
H	5.47859994767841	-0.69397805786413	-1.89649587329572
C	1.60623516106468	-4.02814165246024	-3.45940806698177
B	0.58014419260884	0.27732435397649	-0.44222111905624
N	-1.44164204841728	0.19311622245569	1.17534943018025
C	-0.91350093602285	-0.19946607735808	0.04893177059179
C	-1.80285634479205	-1.27539174374515	-0.57256736428746
C	-2.92163462796812	-1.45638312064880	0.47267845789391
H	-3.89203675294720	-1.62819499608628	0.01160573703742
H	-2.70544087523183	-2.30892829135641	1.11898874606327
C	-2.91338033416884	-0.17355664065890	1.29091989766894
C	-0.90469764060836	-2.52211001996632	-0.65521991134523
H	-0.09808658930483	-2.36179861969483	-1.36818956335676
H	-0.46294110368645	-2.77393328862063	0.30954371900046
C	-2.37952657401929	-1.01327305961621	-1.96444766227857
H	-2.88222653264274	-0.05119065487787	-2.04808136305141
H	-1.60472257102516	-1.05211248496289	-2.71974439792193
C	-3.38997063352794	-0.35090478553599	2.71934242704177
H	-2.90447320772943	-1.18626518820209	3.21423470352323
H	-4.46036322480217	-0.55471232273272	2.69541410001815
H	-3.23398755062148	0.55070353066861	3.31163328799250
C	-3.74172078349870	0.91966415882723	0.61081094697073
H	-3.75977016325353	1.83197393754515	1.19977385577390
H	-4.76821652911068	0.56788061860527	0.50897276108793
H	-3.36105941846684	1.16132757276847	-0.38034271768347
C	-0.71742113819514	0.79103908418420	2.28871558335319
C	-0.14448929064556	-0.09819850665408	3.22050238688604
C	0.55026388875762	0.44226905502404	4.29416623281276
H	1.01001940601008	-0.22493067797511	5.01153057376623
C	0.66826095319491	1.80812728027805	4.46590946548928
H	1.21626876948675	2.20667054669337	5.30986511078176
C	0.07325977994533	2.65858546346177	3.55943393034137
H	0.15102592922155	3.72735712587466	3.70665635535696
C	-0.63330757446444	2.17693660565730	2.45849752750212
C	-0.24479650451170	-1.61696815606071	3.15344424356983
H	-0.93012807496888	-1.88380506578401	2.35185801962589
C	-0.80559786117059	-2.20068821199259	4.45690953186954
H	-0.07134188418367	-2.13916849665615	5.26136214551246
H	-1.04697287219467	-3.25522916062666	4.31640537019598
H	-1.70445968326463	-1.68565732541859	4.79488105078096
C	1.09815352398269	-2.28169406378914	2.83855280809897
H	1.48571311267648	-1.94412922866092	1.88023559616381
H	0.97549163215875	-3.36624601375331	2.81352839807316
H	1.83470858053430	-2.05020143186474	3.61124743628348
C	-1.30305486515388	3.19517719298001	1.55635053303108
H	-1.64226567701006	2.68596459128203	0.65460939741599
C	-2.50978700024322	3.81947419888414	2.26848877688483
H	-3.18028133751710	3.06742048513424	2.68396235584478
H	-3.07962877208100	4.44500675301263	1.57955021504714
H	-2.17774279484467	4.44829511644213	3.09681158196310
C	-0.34320891760834	4.30678240909594	1.12597076718324
H	-0.07150419259696	4.94572712042592	1.96717404248748

H	-0.81987230599190	4.94036603992153	0.37691132699633
H	0.57236542040663	3.90239949645700	0.69822045858509
H	-3.10722039662500	-1.79674069445307	-2.17922896201102
H	-1.50441704527673	-3.37003610592956	-0.98938122334140

cis-TS-2 (Configuration 2)

F	6.00446773633781	-4.51003422257579	2.71540413262280
F	4.06520398131529	-3.70884524064596	3.20239674711131
F	4.25248224907087	-5.22053240311105	1.67500719930090
O	0.62103178028897	-0.58759942374965	-1.40140002535367
C	-0.35757370847334	1.74373827649771	-2.00609520841568
H	0.67771706874881	2.06931403074493	-2.09247202586205
C	-1.29564460231618	2.78764620249302	-2.00264474390639
H	-0.93461490378597	3.76796663406856	-1.70719390399571
C	-2.59496992068301	2.74483645151857	-2.49314115996745
C	-3.51330008405448	3.81846109582052	-2.23074459680493
H	-3.22972063703168	4.55876546424403	-1.49034200127765
C	-4.70622100389149	3.91282582859571	-2.86434636642390
H	-5.38823424559724	4.72029130129569	-2.62901676831238
C	-5.05790139018759	2.96197905803505	-3.87170911553743
H	-6.00185390828431	3.05870371956444	-4.39310597457450
C	-4.20444075262469	1.95711703060119	-4.18174808425438
H	-4.46383676316126	1.24182866273099	-4.95472284915598
C	-2.96428691342571	1.77007282839503	-3.48567181614354
C	-2.07496587536539	0.77533350360340	-3.86194134773254
H	-2.20828453619062	0.34854584137981	-4.85217223713917
C	-0.95390127464456	0.35201578348696	-3.13172757385744
H	-0.18029939456042	-0.11888912780642	-3.73851149010674
C	1.89076243628034	-0.04471684228963	-1.17732921410275
H	2.39883518797583	-0.66687978825984	-0.43662412859487
H	1.82855968339108	0.95942093375780	-0.74882501046020
C	2.74654602812013	0.00592516038734	-2.44734986693192
H	2.19697374543101	0.53782996709442	-3.22504427604405
H	3.63290997657988	0.60547120371683	-2.23370854645383
C	3.12884682400861	-1.36084039170015	-2.95231439868444
H	2.68421371845598	-1.66619668881806	-3.89320697939728
C	3.90094168120379	-2.25973842578767	-2.34354647153148
H	4.00967517812252	-3.23328812185553	-2.81086688366484
C	4.57230707895050	-2.11718080222751	-1.03256477949953
C	4.46233409837010	-3.15595797332392	-0.10818362138682
H	3.91639683752734	-4.04992529105657	-0.37792195066366
C	5.01803440042393	-3.03913906531503	1.15306157920727
C	5.72301099543290	-1.89792057705803	1.51506849907506
H	6.15519105693053	-1.81368350121730	2.50348589141570
C	5.87357449503846	-0.88069707346761	0.58999620219965
H	6.43746659565412	0.00511699392486	0.85042683012849
C	5.30566909794893	-0.99034685848688	-0.67120163014838
H	5.44291024048304	-0.19059350004459	-1.38579552983646
C	4.83701272326975	-4.12482075143417	2.17457444545172
B	-0.57409739066408	0.23356585056953	-1.59799498070537
N	-1.35969198383900	0.12782556266054	0.87714691156367
C	-1.63909720973639	-0.07912069050174	-0.38065080828902
C	-2.98696556928497	-0.79587583414372	-0.47874733133110
C	-3.37815992987321	-1.01793507452450	0.99557559736548
H	-4.44755896271690	-0.90234216430882	1.15950239236127
H	-3.09952406431494	-2.02314830223205	1.31653373105579
C	-2.57917871192514	0.01183408719721	1.77925528955016
C	-2.67679880595921	-2.13088852864821	-1.17777249387556
H	-2.39236074459303	-1.96487960204638	-2.21563745294576
H	-1.86907412908572	-2.67194753436692	-0.68350059824132
C	-4.11095797810433	-0.10202651612436	-1.24898858019756
H	-4.28574683830772	0.92251235414145	-0.92518181538537
H	-3.90271695132458	-0.08449350608352	-2.31109046167366
C	-2.25244422483684	-0.41321789240865	3.19792657300522
H	-1.82781353683458	-1.41137839627779	3.24173772340291
H	-3.18028617992308	-0.42034870140179	3.76974760607989
H	-1.56738875319916	0.28404339174275	3.68003810656647
C	-3.29517459488312	1.36465954332988	1.80882262650151
H	-2.74399387071662	2.09491401878205	2.39405342387824
H	-4.27155010141818	1.23263038428770	2.27473924672592
H	-3.44316239477806	1.77031833391596	0.80928201615697
C	-0.02722476150185	0.30916358490358	1.43594107401436
C	0.67554524846034	-0.86224135886798	1.78166958913395
C	1.95170901865988	-0.72454953516406	2.31139888262388
H	2.51822399836124	-1.61225558046675	2.56044576588321
C	2.51446597219201	0.51942770983389	2.52236030990492
H	3.51288290353069	0.60148051056447	2.93231569026727
C	1.79224812201705	1.65290352802838	2.21488734280469
H	2.22865174560377	2.62539760520392	2.39965073208767
C	0.51188912402281	1.57868933897512	1.66906939241434
C	0.13227676645982	-2.27659358351090	1.62906246260445
H	-0.91789119253359	-2.21640309157339	1.35209586697013
C	0.22204115990677	-3.06002621386168	2.94480013293361

H	1.25581749421958	-3.31923523109628	3.17615765049939
H	-0.33595861155268	-3.99341195031722	2.85761869650779
H	-0.17481820949029	-2.50204315534757	3.79276881935936
C	0.84981872191646	-3.05828028941200	0.52684223994124
H	0.77838258069781	-2.54591127662662	-0.43041143431958
H	0.41418757835238	-4.05508659072320	0.43537989617691
H	1.90459841686857	-3.18041430967815	0.77687951418135
C	-0.21734668630243	2.88162814271277	1.40386974659599
H	-1.11075166483100	2.66272866375806	0.81942804474962
C	-0.62909924806410	3.53821564167760	2.72736802517749
H	-1.15666074868580	2.84764503166521	3.38513693809551
H	-1.27431655507644	4.39879844812890	2.54367517727969
H	0.25223337047161	3.88862936824446	3.26790000304729
C	0.63162878645636	3.86553566757398	0.59563985879028
H	1.48187522224621	4.22694248651315	1.17545095445570
H	0.03245419378673	4.73419391757055	0.31944656977262
H	1.01304917908940	3.41078814277868	-0.31678728215323
H	-5.02844620089232	-0.66950270731556	-1.08825758786850
H	-3.57074777915270	-2.75629359945553	-1.15283074379098

trans-TS-2

F	-6.19008290728569	7.27744260544537	2.81936932542900
F	-7.03452566796359	5.69738631877253	1.61741232636833
F	-5.10583866906024	6.51433640215527	1.11685768779358
O	-0.47337240682646	-1.33857159309161	1.13141658780795
C	1.97727269688422	-0.50175560637033	1.46065093189493
H	1.54542498946286	0.30246414824455	2.05243122235601
C	3.33037035656695	-0.31656012683021	1.13377949121838
H	3.69637318180128	0.70578517232132	1.14344974670012
C	4.29612292810795	-1.30057576782671	0.96558207206341
C	5.59082059081347	-0.97284196728286	0.43494313707209
H	5.72656200774124	0.01183074466277	0.00058972843558
C	6.61743329082392	-1.85536710422471	0.45266722888605
H	7.57561628022808	-1.58839349845632	0.02472407508533
C	6.44972710133706	-3.13581623228607	1.06554567557863
H	7.28279474482885	-3.82654175083233	1.09895340463780
C	5.25879157298060	-3.47221745741934	1.61559085771778
H	5.13282085287483	-4.43809945822951	2.09262035524224
C	4.11996794429914	-2.60200267560628	1.55490914121571
C	2.92923673369778	-2.93524695926507	2.18096186511862
H	2.97506283609170	-3.71231121998125	2.93888964446575
C	1.69730146696170	-2.28476396683713	2.01050297455614
H	1.01268694615235	-2.40868355048895	2.85075698286752
C	-0.89632346727338	-0.25344042215492	1.90232575220742
H	-0.70126868273367	0.69601603172822	1.38528425239848
H	-0.36207943537979	-0.21006684627076	2.86320026988815
C	-2.39056625147209	-0.35752469532879	2.18553199142495
H	-2.92977740482433	-0.41766590958648	1.24122991326088
H	-2.57799241776451	-1.28507429880563	2.73435503750048
C	-2.87154967469718	0.82081424662821	2.96892133843512
H	-2.46987791427104	0.93173270102178	3.97296630669163
C	-3.70018508778281	1.73974525807094	2.48264440926582
H	-4.05969969982119	1.61903331784416	1.46417652870732
C	-4.16974159758460	2.95386848276065	3.16904264405858
C	-4.78680696918753	3.94963456443729	2.41993608248008
H	-4.92069657577345	3.80852455364579	1.35401454322336
C	-5.22583943698045	5.12292652982622	3.01835537482704
C	-5.06604653543026	5.32429113670806	4.37777538952414
H	-5.41069435782064	6.23730293126822	4.84168258691599
C	-4.46067407990038	4.33058556206997	5.13673524537682
H	-4.33729934308193	4.47176179473653	6.20229231457315
C	-4.02162477749183	3.16227038061165	4.54352166464783
H	-3.56573713961009	2.39662358684635	5.15766540879476
C	-5.88774780408953	6.15643941639759	2.15193276905101
B	0.95154383973795	-1.52957858517904	0.83577981190378
N	0.74379569377562	-0.96088301103598	-1.67774987355982
C	1.12058614248820	-1.81961381322997	-0.77075553485058
C	1.57795859637405	-3.10525902411424	-1.45776596489099
C	1.31049215079290	-2.82292431303605	-2.94951368348092
H	2.07921093435988	-3.24661198338336	-3.59246293766614
H	0.35265611418839	-3.24955688030873	-3.25228588472210
C	1.25975117690450	-1.30622379178107	-3.06659143584608
C	0.63816297106140	-4.19734495790318	-0.91867605964499
H	0.82330178696873	-4.37507446735864	0.13978097440724
H	-0.41179261845397	-3.93017799458571	-1.04337824466030
C	3.01895867691947	-3.56130183154247	-1.22980499459062
H	3.75397339446551	-2.78996140175377	-1.45223008862686
H	3.16966176182441	-3.88102989513567	-0.20627476397358
C	0.37291326741845	-0.80403888155145	-4.19015919561415
H	-0.60496142980774	-1.27588951881121	-4.18779902541445
H	0.85686668544246	-1.04613463007397	-5.13617830672523
H	0.24318098527888	0.27730075152167	-4.14827507226848
C	2.66410935728849	-0.71777559690745	-3.22821988130441

H	2.63418968824954	0.36069094626723	-3.35250372425123
H	3.11929368350975	-1.14374166731800	-4.12215470184683
H	3.30217457861351	-0.94529809840202	-2.37568767878460
C	-0.18425929612576	0.13965962489101	-1.45682336730265
C	-1.55500311872687	-0.16601284987021	-1.56718513883261
C	-2.46928149019884	0.85034497214390	-1.32712318030624
H	-3.52763499208602	0.63095780250194	-1.38594187776537
C	-2.05798747930215	2.13485112866894	-1.02376674811277
H	-2.78787735902974	2.91250233834109	-0.83874865828051
C	-0.71106740241092	2.42260519194924	-0.97416774927223
H	-0.39362387359579	3.43429256192042	-0.76058160362284
C	0.25470060969590	1.44008316795576	-1.18764579854786
C	-2.10329511217976	-1.52732870699007	-1.97729932024912
H	-1.26985077869648	-2.15282774557089	-2.28830306182608
C	-3.05533157292420	-1.40492446682248	-3.17362260533106
H	-3.99751297178444	-0.94033345041361	-2.87969401078342
H	-3.28837168960456	-2.39621204600843	-3.56507038804409
H	-2.63351658900870	-0.80760363223907	-3.98185581567099
C	-2.80927291425512	-2.26321617095930	-0.83759836890651
H	-2.14710762912117	-2.39362254293306	0.01501721572367
H	-3.14741497842797	-3.24092924616946	-1.18618341734097
H	-3.69003118898516	-1.70942186914062	-0.50531127454108
C	1.70981826846926	1.86584816897144	-1.16476015122239
H	2.32894464442265	0.96942177049631	-1.14904294644774
C	2.03854456907776	2.68395141004537	-2.42002369370335
H	1.72874983080691	2.18078066213051	-3.33585642276270
H	3.11062985352542	2.87808417383537	-2.48044952280143
H	1.52416823770381	3.64635973108500	-2.39066316037029
C	2.05880983213035	2.68908638966102	0.07705320730866
H	1.57386866381368	3.66580273588218	0.05738636758376
H	3.13518251172500	2.86136978823807	0.11738069520142
H	1.75956787315711	2.18252763674770	0.99261840348866
H	0.82136893498135	-5.12196636342304	-1.46845735536323
H	3.20972095200661	-4.41099629833018	-1.88656827125122

***cis*-TS-3**

F	0.82221515830416	-5.04355297584590	-1.30250202932538
F	0.27152866372169	-4.19501597729825	-3.20930603584102
F	1.73814552756413	-5.76902586191830	-3.11560866430451
O	1.48940518940228	0.30726562562803	0.48952087237123
C	0.47116534285692	1.40963890475694	-1.62206559658787
H	1.28781180112930	2.13899393879469	-1.53173284001761
C	-0.69701797765716	1.98956086088980	-2.36788327296245
H	-1.06422562796969	2.95466685908541	-2.03488176275339
C	-1.23181893853406	1.45020414284640	-3.49433990844623
C	-2.34422881090509	2.05523168835258	-4.20341756031116
H	-2.75142564322058	2.98481706408220	-3.81955325181854
C	-2.87711260712599	1.48581955270422	-5.31064512210263
H	-3.71153151736073	1.97180142745421	-5.80488747827551
C	-2.36837685926466	0.26383397339427	-5.85439801459943
H	-2.81188518567102	-0.16397861908007	-6.74317298916011
C	-1.31276479687058	-0.33353662712602	-5.23025006028341
H	-0.90088398620452	-1.26003072342601	-5.61998289189600
C	-0.72373728981257	0.21178358191724	-4.06660269618792
C	0.37038896653309	-0.38254879030696	-3.42555615339637
H	0.81038725313309	-1.26733013990521	-3.87775712887283
C	0.95516622747648	0.10934038257130	-2.28569990485927
H	1.90096689507983	-0.31567863098569	-1.97992252221913
C	2.56324666531612	1.21431739151314	0.65235294947856
H	2.68688599561107	1.36258123347542	1.72943163199197
H	2.32571746985216	2.18438797622218	0.21383734091765
C	3.85494104379210	0.67561572865728	0.04455986857973
H	3.69990134955861	0.49810597833192	-1.01854794180617
H	4.61257609972698	1.45880651459674	0.13397345183113
C	4.32269996134756	-0.55706618603255	0.75447928879777
H	4.64751582014430	-0.40111111255346	1.77968429511771
C	4.30350080072915	-1.80879367997956	0.30844225516638
H	4.59408864071037	-2.60024751365430	0.99235650382766
C	3.85726653861789	-2.25435560341302	-1.03009297262033
C	2.84692899462123	-3.21050595238077	-1.12810125517041
H	2.40101577253530	-3.61000246631327	-0.22703056791467
C	2.40294372068649	-3.63362819352578	-2.36774408787787
C	2.96169810519584	-3.12382976922960	-3.53381760350379
H	2.60717612707171	-3.45976882704765	-4.49943987315278
C	3.97307342368682	-2.18583848419370	-3.44397268805949
H	4.41708059894556	-1.78382898739122	-4.34458408894095
C	4.42020541456210	-1.75830850910628	-2.20133935158519
H	5.22171547497780	-1.03352802547633	-2.13885788102519
C	1.31050626945213	-4.65756754229021	-2.49024488931456
B	0.39330460165687	0.52491448561675	-0.33009359189401
N	-1.35857703936524	0.33373420938426	1.45908397353624
C	-0.94850498578002	-0.08502131234279	0.29312853676418
C	-1.87786491589552	-1.16597670455487	-0.22711042768694

C	-2.84771468405124	-1.37793651042422	0.95219497293928
H	-3.86324139016702	-1.57571067447099	0.61519477409684
H	-2.52219476944638	-2.22928356107652	1.55264117958012
C	-2.77246232618354	-0.11036948571753	1.79299499011668
C	-1.02415033269817	-2.42007040718614	-0.46315931668399
H	-0.34096417222673	-2.26784030040523	-1.29618033848668
H	-0.43871699868652	-2.67795015554771	0.42191237843686
C	-2.61760030365659	-0.81119741571449	-1.51784343475066
H	-3.17811380139901	0.11841695321524	-1.44379728312043
H	-1.93472863201425	-0.72341938428010	-2.35483892474953
C	-3.00392337080552	-0.35259075237086	3.27384432626210
H	-2.42702591494980	-1.19106780070994	3.65137445881469
H	-4.05890431031949	-0.58746128090748	3.41300692850400
H	-2.77902508325855	0.53372472216231	3.86728209567748
C	-3.74994139128476	0.96310441958061	1.31414851609194
H	-3.68030153229029	1.85654526514266	1.92993022281472
H	-4.76383010890289	0.57434400119713	1.40388608665883
H	-3.58092924788796	1.24305659758534	0.27644190201005
C	-0.57174468737638	1.13815174136812	2.37669777490216
C	0.17284030383067	0.45132740447156	3.35242809420191
C	0.92230439541503	1.20626436344784	4.24420215763112
H	1.51033544141774	0.70135894978493	4.99946618372756
C	0.93730148336535	2.58803331506988	4.18321164633584
H	1.52511828464086	3.15699196357339	4.89181464056652
C	0.20837497126172	3.23673008536289	3.20915803064126
H	0.23343319540255	4.31734980843888	3.15926506774751
C	-0.55449880176998	2.53314755691156	2.27830560625556
C	0.23228339544352	-1.06508281994345	3.47465373612738
H	-0.50626458215440	-1.49840358976160	2.80203896961759
C	-0.09196778702365	-1.53438265674258	4.89689115435851
H	0.71264139862029	-1.27859939246583	5.58767161933640
H	-0.20285425108311	-2.61944653572979	4.91386312784181
H	-1.00992419871046	-1.09012610269406	5.28094125527743
C	1.59451611725316	-1.61374618229720	3.04412134377045
H	1.81402831047572	-1.35390514379918	2.01185049118682
H	1.60636004161556	-2.70062803054498	3.14503456139396
H	2.39247011491045	-1.21236553858705	3.67284078789955
C	-1.28983882766885	3.33230798523278	1.21720899515133
H	-1.77018116078321	2.64012413562519	0.52588809104529
C	-2.36593586986544	4.22702578954610	1.84160167817271
H	-3.02488241670498	3.67646411674893	2.51227898990664
H	-2.97555162063048	4.68450308617176	1.06149262408643
H	-1.90906615154648	5.03094853830708	2.42154351136679
C	-0.32164138300642	4.19127634144884	0.39976830440459
H	0.16077681995221	4.94574538059630	1.02271405631611
H	-0.86118948658477	4.71162276065628	-0.39218038346830
H	0.45469740864262	3.58588998974454	-0.06384916491515
H	-3.32003417504190	-1.61711961250649	-1.73479541714771
H	-1.67934964442997	-3.26109924240393	-0.69486993155743

cis-TS-3 (Configuration 2)

F	4.11848157602126	-3.83522582343154	3.36391862711390
F	5.12937411306689	-5.33658340303695	2.19405946191572
F	6.27295127187747	-3.86616491387191	3.28344506492237
O	0.49249372985791	-0.53773422584424	-1.20390754649963
C	-0.65676183680677	1.47764203267939	-2.33417475409255
H	0.36279675940749	1.77357567491619	-2.61998502027050
C	-1.57598346901670	2.66551931809373	-2.28488011392435
H	-1.24440219352637	3.52205450698996	-1.70701108723324
C	-2.72309148138204	2.77731022530877	-3.00628508576042
C	-3.57467998360758	3.95026466901179	-2.95103848310632
H	-3.26790353120064	4.77638363344331	-2.31796948346413
C	-4.72882919724464	4.02800548338707	-3.65744667868882
H	-5.33519867823121	4.92420861854342	-3.58013658952845
C	-5.16880013484756	2.96751107765069	-4.50822955767810
H	-6.09089931127436	3.06051794801225	-5.06558581326921
C	-4.39039012258470	1.84930645104179	-4.60364129314661
H	-4.69329822957011	1.02836257301125	-5.24737511783379
C	-3.18488520271701	1.71137244719684	-3.88227793452154
C	-2.36616234121400	0.57445743928017	-3.98669945442012
H	-2.65456713953877	-0.20004153237089	-4.69318657181828
C	-1.20005589700763	0.40449157126476	-3.29079978739129
H	-0.55920521696994	-0.42501184560457	-3.55819238070766
C	1.82622531927525	-0.13632695380857	-1.47488239280443
H	2.41729065176954	-0.44369951540828	-0.61077504908107
H	1.89430140373624	0.94971234371651	-1.54595367006959
C	2.37365984265307	-0.76216983297018	-2.75152828197374
H	1.72196718076652	-0.48738496752764	-3.58104281951332
H	3.33662709476428	-0.29502513093364	-2.96598061683673
C	2.50142291495224	-2.26221498071130	-2.69893260788477
H	1.87231436340819	-2.80614232500993	-3.39482182226951
C	3.24991189267298	-3.01299429843360	-1.89052921014637
H	3.13858338084007	-4.08834389638953	-1.98732013405385

C	4.16927313156423	-2.60723221141622	-0.80923081827931
C	4.31153984852100	-3.46446618047820	0.28031479673874
H	3.77122709809725	-4.40278538730672	0.29102400859708
C	5.12295975309352	-3.12804186147769	1.35189959832733
C	5.83500911201289	-1.93877727649262	1.35317430504253
H	6.46951601290305	-1.68036405352616	2.18931759712608
C	5.73300584858665	-1.09713444079257	0.25673205948228
H	6.30016364057218	-0.17623667440840	0.23207600147407
C	4.91227632075386	-1.42544283892834	-0.80912013268596
H	4.86375700141709	-0.75677854248942	-1.65599457953456
C	5.17139265142262	-4.04246861633146	2.54247396953026
B	-0.58462016750957	0.32493050385333	-1.26979865800336
N	-1.24383525946635	0.25375273473625	1.14544120038962
C	-1.64070547446881	0.10087286265199	-0.08782697536740
C	-3.10384045220822	-0.29963265738149	-0.11945375895494
C	-3.43766621642052	-0.47881828606534	1.37566971027043
H	-4.44578346290438	-0.14386329773852	1.61118795431926
H	-3.36199253500434	-1.53251629015127	1.65005420497775
C	-2.38871650198106	0.31802594368582	2.14133971671416
C	-3.19413265386231	-1.64164076707809	-0.86131103381131
H	-2.96822326806129	-1.51420973801580	-1.91850358254822
H	-2.50322446377098	-2.37656956420174	-0.44352802148339
C	-4.03115127170855	0.71006537119125	-0.79770865507741
H	-3.95603899458569	1.70819702670642	-0.37178227059522
H	-3.82620864026090	0.78625819061351	-1.85970991722375
C	-2.04701741871393	-0.27017060683109	3.49894515053389
H	-1.86413015180310	-1.33937264424965	3.45500329922478
H	-2.89857671066287	-0.10621489247426	4.15877610308211
H	-1.18183898341153	0.22224539506897	3.94320415278315
C	-2.79806594140684	1.77926546817022	2.32963490163854
H	-2.03975126219275	2.33044084157750	2.88029678527891
H	-3.71912166474929	1.80807112250377	2.91101579846714
H	-2.97522561265881	2.28444903208413	1.38236958396070
C	0.14773851035803	0.32761869070269	1.55649746779235
C	0.77275888346577	-0.88297920229156	1.90474250845744
C	2.10883994585372	-0.83662890264354	2.27880134910613
H	2.61948022601444	-1.75476544121743	2.53784765316748
C	2.80339423289808	0.35902619443778	2.31676327222545
H	3.84503422481694	0.36971331710007	2.61025557848974
C	2.16740958136218	1.53229076760227	1.96911331900222
H	2.71843262264041	2.46321036679740	1.99559643261101
C	0.83060712906968	1.54755285526579	1.57372690136662
C	0.08305531334982	-2.23946534244054	1.87110955877527
H	-0.98078130761654	-2.08585700019704	1.69898209123979
C	0.24187857647122	-2.98741065135911	3.19891850221173
H	1.27149521278963	-3.31741113698066	3.34240673458970
H	-0.38942073239093	-3.87706807474383	3.20199279711813
H	-0.03049200932221	-2.37104904368341	4.05563820620927
C	0.59653187397120	-3.10846313047909	0.72188989830773
H	0.45670639496203	-2.61785952445869	-0.23830207232201
H	0.07069639560095	-4.06476920212778	0.71444384005460
H	1.66086702255353	-3.31266526683619	0.84488590967900
C	0.21642022662589	2.88089292233444	1.18638128246944
H	-0.79542742772992	2.70675152304846	0.82102603581248
C	0.14561319031150	3.82131997997693	2.39444681870865
H	-0.32090552564710	3.34903999037429	3.25875407605216
H	-0.42347471963717	4.71741761247378	2.14399880334992
H	1.14662844885764	4.13499762478224	2.69616299785779
C	0.99475313536921	3.55680250540011	0.05538447719421
H	2.01324704614109	3.79818930650235	0.36259170722870
H	0.50446442924166	4.48864694415442	-0.22840527231002
H	1.04632232888712	2.92435389384400	-0.82864099068753
H	-4.20827712175713	-2.03207280728013	-0.76151372330057
H	-5.05713494697230	0.36117119873874	-0.67231045081442

***trans*-TS-3**

F	-4.13575525785647	7.21504358797545	2.69332775105830
F	-5.78934641997929	7.40650300446036	4.06514601199208
F	-6.06824978546544	6.42559088652137	2.16327480679937
O	-0.56217556316916	-1.11926803872939	0.76936685928434
C	1.91930428285183	-0.95353254228518	1.45707414333173
H	1.58239295576215	-0.17271444060679	2.15187169168418
C	3.37418803670086	-0.79529368756650	1.11936125940344
H	3.70260945090702	0.19031378528858	0.80523746948865
C	4.31244018325386	-1.76153306382582	1.30355534591030
C	5.71499321624800	-1.56413455567170	0.98991703360093
H	6.02298364112236	-0.59628891163645	0.60817482429631
C	6.62849505753397	-2.55112245391550	1.15528804202330
H	7.66559051259808	-2.35813544437312	0.90243558993620
C	6.26873277534506	-3.83898080795416	1.66138376581867
H	7.02116250555877	-4.60516269915347	1.78943781576950
C	4.96230584129778	-4.06531531258531	1.98604751572610
H	4.65841577357138	-5.03081260028533	2.38035966130152

C	3.96704571167670	-3.07612382151202	1.82394304962123
C	2.62529536657591	-3.28946913737695	2.17598238122138
H	2.36350815829674	-4.23601208916007	2.64244929326349
C	1.63392440261767	-2.35692436723717	2.01783163202576
H	0.67704370456461	-2.54348235258177	2.48921622860518
C	-0.96020400886590	-0.24701226105180	1.81193347664908
H	-0.54872354461233	0.75119990754289	1.63509177764846
H	-0.58231958923314	-0.60028571554635	2.77703446251300
C	-2.47825798164890	-0.16445965208408	1.83590437897428
H	-2.82679652363545	0.12799532503670	0.84516876879848
H	-2.88237038401452	-1.15789208184496	2.04947839156092
C	-2.95226830309042	0.82229587200221	2.85456433872850
H	-2.75172164632229	0.58055486204385	3.89487617507558
C	-3.55313587962851	1.96546398452636	2.53960805268093
H	-3.71342452946683	2.18754281859448	1.48763972075405
C	-4.01818663963283	3.00463984506498	3.47257398896109
C	-4.40307169055669	4.23859136059468	2.96008846786925
H	-4.36200885185624	4.40762904566336	1.89078079942592
C	-4.83681711475025	5.25459425628035	3.80072721126527
C	-4.89800324736548	5.05976778073086	5.16919834118254
H	-5.23797488018444	5.85151901829903	5.82128439779875
C	-4.52471672910618	3.82726821060060	5.68972368162478
H	-4.57796350928345	3.65877569150055	6.75705546878507
C	-4.09388564632985	2.81362921118442	4.85514522959775
H	-3.82222450318765	1.85758476194534	5.28317108220431
C	-5.21141085945204	6.57486807912432	3.18894219375327
B	0.77236997154065	-1.29336705994284	0.44329899063021
N	0.62709941554260	-0.83726362642953	-2.01912091995536
C	1.00462157973554	-1.67298285287926	-1.09126565419671
C	1.60981676892896	-2.91359894855526	-1.72302901727629
C	1.39288514326207	-2.66676754355734	-3.23017188936074
H	2.22772185880826	-3.02886666779010	-3.82665265626167
H	0.49189313994974	-3.18249984762607	-3.56733489436246
C	1.20353665112873	-1.16317168368513	-3.38627250936127
C	0.77678977227735	-4.11064926557310	-1.23908229146332
H	0.93677043917936	-4.28652886160204	-0.17653531919805
H	-0.28993199602303	-3.95177027874734	-1.40885852377512
C	3.08062640149212	-3.16578312696893	-1.39029838181328
H	3.72323062064280	-2.32432376306911	-1.63931879726756
H	3.21750641891218	-3.38788279954014	-0.33789771234542
C	0.29483115499825	-0.77670924063360	-4.53965901368391
H	-0.62582190567089	-1.35188672531217	-4.55570629205324
H	0.82794351756165	-0.97922449703294	-5.46819426692344
H	0.05215920942792	0.28586871436729	-4.52001237107197
C	2.53596159503458	-0.42894497792384	-3.54302053320931
H	2.38242632156146	0.64128971053980	-3.65695819837680
H	3.02887775284346	-0.79313897410043	-4.44394544033879
H	3.20037183547353	-0.59547368150407	-2.69745584457583
C	-0.27462952786187	0.28029978282710	-1.80277317741443
C	-1.64392463001406	0.03843859573699	-2.01124005740359
C	-2.52506965927458	1.09020627137064	-1.80237204338375
H	-3.58522704929182	0.92888428781329	-1.94812917861348
C	-2.07669617922018	2.33481163883744	-1.39778123730591
H	-2.78128600278332	3.14091548791500	-1.23847901699291
C	-0.72884743741266	2.54388794803785	-1.19408497750519
H	-0.38532302874086	3.51856594073847	-0.87360023967064
C	0.20437847801498	1.52663183455486	-1.38785882083310
C	-2.21756760614220	-1.31073134565524	-2.42227231444693
H	-1.39533535133034	-1.96696818747223	-2.70095147964812
C	-3.14643503876295	-1.18866005478598	-3.63417124190661
H	-4.07496922386358	-0.68070524872973	-3.37028367836237
H	-3.41123605227744	-2.18129719931677	-4.00074975922280
H	-2.68634879111362	-0.63297217617274	-4.45127509772664
C	-2.95207747991155	-1.99643631493948	-1.26774617709141
H	-2.29053116021069	-2.15292608525640	-0.41873323355935
H	-3.33924840435022	-2.96172103411798	-1.59828407565669
H	-3.79881276858082	-1.39402922287810	-0.93210449749552
C	1.66678718332760	1.84420219663784	-1.13455752861041
H	2.24611667052101	0.92632448230757	-1.23276303411874
C	2.18643325713815	2.86360621512508	-2.15435436301265
H	1.96401247906674	2.57200062177565	-3.18064849001889
H	3.26643765270851	2.98022733132619	-2.05676637221912
H	1.72837239719120	3.84021643655226	-1.98741584608578
C	1.89436777862048	2.37486284060852	0.28270921097407
H	1.39009761305463	3.33005056071775	0.43535580789804
H	2.95965250094007	2.53250217530226	0.45434649195459
H	1.53323480378450	1.67635504399252	1.03485310845927
H	1.08148197561361	-4.99961862195426	-1.79375938466694
H	3.41108844676497	-4.03066346132911	-1.96729033808689

cis-TS-3 (OSS)

F	-0.01321942664683	-4.75758344000635	-2.78043447105789
F	-0.17428430350579	-3.30265924635863	-4.36561228965563

F	0.83224583973860	-5.17336943523753	-4.72191179447023
O	1.98250437757209	0.25791660552552	0.64034840082690
C	0.82913890942729	1.39170278101257	-1.38909281996489
H	1.45570886055296	2.26865601234041	-1.15992079681898
C	-0.52033401063245	1.84578598281610	-1.81477621033468
H	-1.05658662249067	2.52361182152850	-1.16769164906145
C	-1.15625556363287	1.40733964031476	-2.97356509196153
C	-2.48702526236979	1.78910538436930	-3.28817338286965
H	-3.00713920830808	2.46213041997872	-2.61627441267373
C	-3.11487391091798	1.30430294839723	-4.41046706383068
H	-4.13407889974705	1.59908069914041	-4.62677322548849
C	-2.44671237886080	0.42901296647807	-5.27547786711709
H	-2.94761388691994	0.04740276295537	-6.15522754662134
C	-1.13838389975682	0.05653453039924	-5.00138715831334
H	-0.61542734128083	-0.61769948126640	-5.66967033882600
C	-0.48149424256145	0.52836964594362	-3.87308144439285
C	0.89266331502224	0.17494215726900	-3.57110380451324
H	1.43169136578443	-0.43223571342368	-4.28946161670010
C	1.50676752828075	0.58564127373928	-2.45776066520663
H	2.53953655186697	0.30722052358413	-2.30309913381061
C	3.28273884632422	0.78773747381038	0.45026613655412
H	3.64157334261519	1.05275024056026	1.44664213235733
H	3.25561199502867	1.69982637135442	-0.14990378389322
C	4.22782730472768	-0.22629369512359	-0.19145229315062
H	4.04857625654031	-0.27211371563851	-1.26180731752724
H	5.24455233960914	0.15557235399555	-0.06104265134173
C	4.10995167964819	-1.59115114113646	0.41261882975157
H	4.26403571904517	-1.64911447480192	1.48619262481621
C	3.77436113251584	-2.71218697225285	-0.22122212364954
H	3.65309701257153	-3.61523617285658	0.36832594264199
C	3.47085008183115	-2.84743347032696	-1.66390756685511
C	2.29341123008859	-3.48698661316090	-2.05234468305629
H	1.63463499845792	-3.89809682248972	-1.30060649360125
C	1.95126560131052	-3.57999254763625	-3.38832399125334
C	2.78798774140316	-3.06856105927038	-4.37269758518290
H	2.51390791257832	-3.14767654883622	-5.41650377311509
C	3.97683260917060	-2.46880795097100	-3.99988987402456
H	4.64666908054375	-2.08293888133184	-4.75666457891902
C	4.31556816940765	-2.36066577667724	-2.65774391634838
H	5.25586672765753	-1.90262148257130	-2.37967648889835
C	0.65080795350091	-4.20674066560034	-3.80492383740642
B	0.81815598963470	0.51080540591258	-0.01529313527706
N	-1.30344503665612	0.59741506502901	1.47378100983191
C	-0.47623266357070	-0.11659284143984	0.60511643368567
C	-1.08804524698866	-1.46449592502950	0.26750770772981
C	-2.37250082560579	-1.46452260772294	1.12606050386737
H	-3.21546956726719	-1.91648845131407	0.60241659610653
H	-2.20879844003899	-2.04426627454782	2.03690780501950
C	-2.66227250216713	0.00016168938103	1.50683745764966
C	-0.14598523604744	-2.60994216943970	0.67024604256230
H	0.81659602455056	-2.49893837626726	0.16927748832710
H	0.04448293264691	-2.61858783711293	1.74258673344482
C	-1.39758547232590	-1.63259007029371	-1.22744601633593
H	-2.08163196509457	-0.86960186952704	-1.59513140858159
H	-0.48325080459956	-1.56903385165318	-1.82099583521243
C	-3.32321932878764	0.11779761279167	2.87220518840284
H	-2.74612991121269	-0.38759937435011	3.64424819807002
H	-4.31186336777600	-0.34088170851853	2.83494465161379
H	-3.44824847609923	1.16119400448639	3.16494911187862
C	-3.56399506565380	0.65874192695696	0.45456299933145
H	-3.83727854501017	1.67240962601590	0.74681292944944
H	-4.48536565390492	0.08586214506522	0.33731127816274
H	-3.06445424368109	0.70522064602320	-0.51289118270832
C	-0.75984175321898	1.44426329351268	2.49510643210461
C	-0.05277603194972	0.89053519124964	3.58446200188933
C	0.52397785121944	1.74372818460059	4.51762954758805
H	1.07657002252005	1.32211166709941	5.34844128277389
C	0.39242367128390	3.11684867714405	4.42079043990327
H	0.84372522882690	3.76324838564347	5.16288515308999
C	-0.33050026367875	3.65291728603574	3.37482667691043
H	-0.44644433009758	4.72749561978523	3.30391865604573
C	-0.90660528821117	2.83970924541440	2.40223969500173
C	0.08179372346459	-0.60334595242407	3.81564876092771
H	-0.51817687956562	-1.10528997946086	3.06210561504954
C	-0.45965222150646	-1.01575533096496	5.18807087534875
H	0.16555508911218	-0.63207483340146	5.99619740973340
H	-0.47470951354644	-2.10383130347458	5.27199703974265
H	-1.47318317321464	-0.64860887920614	5.35282746211709
C	1.52590649434143	-1.08038638299929	3.65158148340806
H	1.89784151704317	-0.84872071583743	2.65530023095882
H	1.58815543883100	-2.16026116959286	3.80251065068612
H	2.17960855532053	-0.60434561484209	4.38613120103119
C	-1.67439452864766	3.50468034444802	1.27877218844758

H	-1.95426279700628	2.71952696125498	0.58076871883184
C	-2.96194821721631	4.15421659643904	1.79060422293272
H	-3.59079045432741	3.43295956679547	2.31427861005498
H	-3.53854837962841	4.56752964892553	0.96091040932588
H	-2.74245202056099	4.96805165128894	2.48473240401998
C	-0.81738571317871	4.52585140445931	0.52736346175667
H	-0.57112866774226	5.38219811977858	1.15739395258043
H	-1.35196855672820	4.90252698449475	-0.34667548414705
H	0.12032055554290	4.08418976757853	0.18664472073215
H	-1.84454095881579	-2.61153170423523	-1.41484865970590
H	-0.56698451819931	-3.57898378249300	0.38864895880602

cis-TS-4

F	1.55859275295429	-4.85592330353506	-1.19874613845602
F	3.21870279173829	-5.86874543164642	-2.12888532778936
F	3.24854861981958	-5.46420788112404	-0.00909125129140
O	1.19642855369762	0.65551219164729	0.04942532239196
C	0.84456307678206	-0.87700698651163	-2.23051956404401
H	1.64371603182080	-1.04587726038017	-1.52488938915256
C	0.46827336506330	0.41514246039377	-2.57414256262254
H	1.07466846667586	1.26445405515307	-2.29696834675090
C	-0.45571135880886	0.62484365194286	-3.65797149234117
C	-0.84902831789985	1.91767554530543	-4.04416514330216
H	-0.45833982517709	2.76806685702793	-3.49821180545744
C	-1.73851079325698	2.09916759715301	-5.07169505570904
H	-2.04656813366852	3.09794530421527	-5.35226904406696
C	-2.25973729959902	0.99026459647514	-5.75834365150794
H	-2.96742038583614	1.14085387733688	-6.56309592169604
C	-1.87090227272862	-0.27810553336205	-5.41320087467084
H	-2.26551997867259	-1.13654234899901	-5.94314335485816
C	-0.95729403726545	-0.48778690684249	-4.36123098001758
C	-0.52226358013223	-1.79973944149982	-3.99130654879829
H	-0.89963698659167	-2.64584462274172	-4.55282139926738
C	0.32778634213670	-1.98793337338822	-2.95303207979814
H	0.64236670231796	-2.98359198980954	-2.67290496515510
C	1.94856864306277	1.84503937246737	-0.05104650859143
H	1.93432479053297	2.33276148783274	0.92637945572198
H	1.49687647907364	2.53471665472579	-0.77238040305604
C	3.38041326574360	1.51520209606080	-0.45351090883443
H	3.36550291415433	1.00493161647783	-1.41548508104572
H	3.91837669160417	2.45709059124477	-0.59127957366240
C	4.06058335257305	0.69754222884182	0.60122830962111
H	4.19129416304885	1.20316462572581	1.55411770059266
C	4.43979203509391	-0.57581102201539	0.53729317804791
H	4.83632865851125	-1.03130548480034	1.43898401058271
C	4.29539857654200	-1.48763174898281	-0.61662864421079
C	3.75777176699873	-2.75457349063513	-0.40280527266727
H	3.48372933733831	-3.05399478639996	0.60009406052997
C	3.55544821921020	-3.62476221242435	-1.46149855196829
C	3.89852697370602	-3.25785889769807	-2.75469120854913
H	3.73630634627089	-3.93894278969165	-3.57834005877739
C	4.46950716053608	-2.01445822936535	-2.97118383342628
H	4.76335279868410	-1.72537331359374	-3.97131745099913
C	4.67224278261651	-1.14175505114025	-1.91360292521674
H	5.14083330590797	-0.18350520949561	-2.09446860970809
C	2.90548742664212	-4.95391349246196	-1.20052885269688
B	-0.04009872677526	0.42406268335729	-0.48781439753104
N	-1.65107000745710	0.67781330726306	1.36437923229449
C	-1.37049868400676	0.22276174078048	0.04429683577083
C	-2.67234609120974	-0.36148041983329	-0.51935872970674
C	-3.57188702412797	-0.44384088633161	0.72772913646261
H	-4.62722148012456	-0.33146830649793	0.47588601796141
H	-3.44752703802725	-1.42620827116143	1.18927444646175
C	-3.08576801924582	0.61686947590685	1.71350911625653
C	-2.52001804363149	-1.77419318472430	-1.08135964644525
H	-1.96463582414660	-1.78042813286361	-2.01251005532975
H	-2.00334049375473	-2.42374613409405	-0.37312898616959
C	-3.30471842280724	0.52668254434670	-1.59956864090422
H	-3.42856000859817	1.55479537707878	-1.26574438441233
H	-2.68780437647752	0.54600446630031	-2.49504672791340
C	-3.30428138110228	0.17548283990507	3.15694833246017
H	-2.86404311534987	-0.80217170527781	3.34426671782526
H	-4.37465455715594	0.11085450386095	3.35771394331369
H	-2.87306436050725	0.88754648009903	3.86191371012345
C	-3.84107403462570	1.94149494800224	1.50278861254630
H	-3.63120495284218	2.65259695403228	2.29881925748117
H	-4.91601714407844	1.75288339325483	1.50745129020012
H	-3.58394058020746	2.40904529136469	0.55430388311358
C	-0.61305378538736	0.85852379505113	2.32333643902841
C	0.07827213638553	-0.26341289041557	2.82562588392462
C	1.13005759553813	-0.06797221268827	3.71147326447136
H	1.67170762432165	-0.92667044395130	4.08984852600080
C	1.49704885077919	1.19998065083347	4.12686258193853

H	2.31606355403687	1.33130986449639	4.82295360524090
C	0.80444532051509	2.29474853551026	3.64949565688310
H	1.08726545751585	3.28754830667769	3.97877551975114
C	-0.24185505983715	2.14946660261391	2.74143493345543
C	-0.27320939449275	-1.68796907540971	2.43455998012787
H	-1.14609233445265	-1.64632655216799	1.78630487659010
C	-0.62903406516568	-2.54285655823025	3.65424267247180
H	0.23751294932723	-2.69259140819543	4.30111567860298
H	-0.97592995895776	-3.52741638123555	3.33530265070576
H	-1.41411703077906	-2.08476230334651	4.25650335625904
C	0.85831671149983	-2.34747239797851	1.64394513729390
H	1.12976874243161	-1.74740451591798	0.77755034710014
H	0.55968502135840	-3.33928191702916	1.29879837267327
H	1.74908050362091	-2.46463041501998	2.26642378518888
C	-0.95368475955104	3.40022289605601	2.25932710390686
H	-1.66518572643838	3.08964834490798	1.49915512718226
C	-1.71860771640707	4.06026167054161	3.41111099885319
H	-2.35964621764536	3.34755045083861	3.93100223254089
H	-2.34302509042228	4.87673392796171	3.04317508439631
H	-1.02638590934848	4.47481956071964	4.14725130038774
C	-0.01941569396484	4.42066083489840	1.60633318696368
H	0.77152670112793	4.74346740877055	2.28617092361985
H	-0.58504163348218	5.30744921092515	1.31442903987537
H	0.44773759000565	4.01565595059440	0.71053757230026
H	-4.28728919994917	0.13773924896801	-1.87878056915190
H	-3.50652023714419	-2.20156115502998	-1.28113348976685

***cis*-TS-4 (Configuration 2)**

F	4.16108236761728	-4.76157445010393	2.66027510756575
F	6.26597951724452	-4.37331524095166	2.93329472333542
F	4.80633903776658	-3.05406490787207	3.81071407548819
O	0.29558496778177	-0.69796791779254	-1.35609452930117
C	-1.33798129296654	-0.13752861208885	-3.47077005899013
H	-0.70230950604612	-1.00595194448727	-3.40183553937123
C	-0.87302255412442	1.11092808386133	-3.07883480883505
H	0.16088301558145	1.26673373199022	-2.80816345658993
C	-1.63623728260815	2.28982069629272	-3.38797755181806
C	-1.18656524806030	3.57175062806188	-3.02516568621691
H	-0.25254239242476	3.66651517277612	-2.48523512058678
C	-1.92880514110632	4.68455407158133	-3.32680510231127
H	-1.57972489893678	5.66514548144210	-3.03068732803628
C	-3.14598576177958	4.55568421418959	-4.01459672795227
H	-3.73089569238148	5.43725310864226	-4.24214998231131
C	-3.58806034299323	3.31769100454132	-4.40495379166675
H	-4.52074100779587	3.21384419774470	-4.94604823265965
C	-2.84017138480007	2.16173088293338	-4.10899385639898
C	-3.26592254009317	0.86044103532436	-4.52971804265317
H	-4.18052939838994	0.77840293367148	-5.10445797102113
C	-2.55696734534565	-0.24497906989588	-4.20400563749313
H	-2.89042791679113	-1.22413600322400	-4.52034467864906
C	1.59114654963861	-0.12708618845256	-1.28255805619283
H	2.05875567414020	-0.47839710725796	-0.36025204885546
H	1.53053535238977	0.96096023061910	-1.22542174885145
C	2.43203921728830	-0.53398299243144	-2.48717763219879
H	1.95514475532345	-0.16558215153253	-3.39724275489091
H	3.38440214039297	-0.01174223059445	-2.40624310938981
C	2.61867665904029	-2.02333818533201	-2.60075518445248
H	1.99666917992657	-2.51734307574367	-3.33906537575246
C	3.39407901397790	-2.81394945742022	-1.86204980229065
H	3.32509738152068	-3.88222667833375	-2.04143876465905
C	4.28906155058854	-2.43939484992628	-0.74675434172789
C	4.29713702762847	-3.24840222683442	0.39010030900029
H	3.66036264145822	-4.12203725580399	0.42380058547694
C	5.09336051523183	-2.93382392543170	1.47512494920369
C	5.92268087033152	-1.81969922255591	1.44628348522226
H	6.54422013663405	-1.57958904408234	2.29883385782247
C	5.94611432381770	-1.02955280483068	0.31166238171734
H	6.60017228751918	-0.16895170206821	0.26817406983249
C	5.13934314589629	-1.33732445398961	-0.77465243996430
H	5.19274766900128	-0.72180746188737	-1.66165868728808
C	5.07726319965731	-3.78476981018819	2.71295457265291
B	-0.89220395359009	-0.03317878015353	-1.21896165155467
N	-1.50882755324495	0.04247209403685	1.17768082523531
C	-1.85675458969586	0.26183227250748	-0.18571446813706
C	-3.29151053305657	0.78860944541611	-0.17831337127468
C	-3.81932685708278	0.25350772829713	1.16361616342571
H	-4.61893774167803	0.87864728376342	1.56426084584008
H	-4.23341804811504	-0.74375826558135	0.99920093251610
C	-2.63900819432911	0.14251386498750	2.12619712227216
C	-4.18824598506629	0.25059148812971	-1.28538494514431
H	-3.95189784979245	0.69304166401092	-2.24558082140331
H	-4.09533414387202	-0.83246019436835	-1.37503458514257
C	-3.33753315679202	2.32368526942062	-0.22070178214805

H	-2.71426005846312	2.77743934548872	0.54715638676200
H	-2.99024777284773	2.69224869066400	-1.18475488819136
C	-2.79049541963085	-1.09319566633832	3.01479507601252
H	-2.90810244705619	-2.00079578737310	2.42659473074055
H	-3.67701594299393	-0.98049341574918	3.64115822353282
H	-1.93108050902450	-1.20840269649845	3.67610307744388
C	-2.54561366937267	1.34512573348271	3.07596613524762
H	-1.63348992985156	1.29236902343047	3.67124930190309
H	-3.39059739803531	1.32461562372990	3.76553805017100
H	-2.56636531917027	2.30039294224263	2.55764259868295
C	-0.15838150398928	-0.11749674442396	1.60918480329331
C	0.38534471568655	-1.40663136177382	1.78710677772248
C	1.69533655818487	-1.53071560008065	2.23918294204923
H	2.11066525414726	-2.51917198435070	2.39020131515283
C	2.48406748442097	-0.42252666121389	2.48132014672070
H	3.50288084272603	-0.54424697414640	2.82682754312631
C	1.96384335180628	0.83859776039334	2.26244772483322
H	2.58816458185860	1.70724231651838	2.43372633444305
C	0.65314670937924	1.01612885430442	1.83022559777421
C	-0.37364376743384	-2.68304218608100	1.46244310498420
H	-1.36842562921060	-2.39263001089716	1.13033509642174
C	-0.49838388179332	-3.60578901965729	2.67874102527437
H	0.47737418220795	-4.00398688775619	2.96494317443147
H	-1.14201405480427	-4.45539338080715	2.44311420472105
H	-0.91461118872519	-3.09605651032461	3.54591552879651
C	0.28253525031388	-3.45308740942855	0.31294545114759
H	0.36452370688505	-2.83499476437633	-0.57769511874431
H	-0.30791924937611	-4.33969165176106	0.07308493559167
H	1.28441149146859	-3.78904848307294	0.58933772575460
C	0.16451163349199	2.43771541086967	1.60858655803990
H	-0.89496422318789	2.38079643423684	1.38524804086360
C	0.34871989442333	3.31143677802619	2.85200634946839
H	-0.06971229627995	2.84346429666514	3.74254781117988
H	-0.14534794655272	4.27448355185987	2.71096769963737
H	1.40449262631567	3.50996510259197	3.04610713656187
C	0.83219980406820	3.10207144727065	0.40337492273084
H	1.92041917585277	3.10846368793391	0.49771740537266
H	0.49543632710170	4.13652325117702	0.30997164584797
H	0.56645845631573	2.58294944116519	-0.51604382391961
H	-5.23107625242845	0.49105866188167	-1.06223829400761
H	-4.36177347086284	2.67586846915325	-0.07345679000062

***trans*-TS-4**

F	-5.61644341043926	6.83789088758055	2.90155470441942
F	-7.29380034803609	6.08249133568966	4.02697991820323
F	-6.92032566519534	5.36318029037710	2.02686629510394
O	1.31486558629426	0.75101231649557	0.72298881747999
C	1.60696088369341	-1.59939777593964	2.22906150857678
H	0.83450609721891	-0.91032926716157	2.53094288842462
C	2.89166345198620	-1.15806979437961	1.95429014101112
H	3.13105675114591	-0.10451236664251	1.95936742287044
C	3.97791027339447	-2.09025259446700	1.91142583129212
C	5.29431420673941	-1.66914806005175	1.64749078751967
H	5.47979861307518	-0.61887394438702	1.45873968422544
C	6.32021779911345	-2.57688016213352	1.60469501909931
H	7.32815790249961	-2.24679987763314	1.39011892791128
C	6.06680378568325	-3.93958125913152	1.83246504275962
H	6.88113573400916	-4.65101381771284	1.78942073213549
C	4.79482310365271	-4.36837030400609	2.11286156778638
H	4.59703558324362	-5.41781183578972	2.29421902761909
C	3.72551478847901	-3.45427497083149	2.16372291693951
C	2.38850742816197	-3.87287973321660	2.45923832473775
H	2.21007511703720	-4.92132713500003	2.66502353396422
C	1.36735986291475	-2.98736019061633	2.47299515397778
H	0.36001404203061	-3.31110733087504	2.69832637803105
C	0.44537967088852	1.29806291806625	1.69608470495954
H	0.48281009878760	2.37997045128223	1.56102020097216
H	0.84139938682743	1.09418035635778	2.69845516713107
C	-0.99797835477259	0.83272417599437	1.58185323095889
H	-1.36988773477450	1.07811383638322	0.59013161062339
H	-1.03810729006855	-0.25571504637485	1.67778004852199
C	-1.85875106365293	1.46390900528518	2.62765636304016
H	-1.62678980201582	1.21864963071937	3.66098132990622
C	-2.85186923307714	2.30246037495676	2.34777865440765
H	-3.04133288312508	2.53923844796724	1.30426758476139
C	-3.73899969297327	2.97307675394418	3.31195140789151
C	-4.59578282355044	3.96718710631470	2.85072952143777
H	-4.59744406274380	4.22184888135344	1.79793626073135
C	-5.44705649502846	4.63326368663913	3.72131932111690
C	-5.46211701205219	4.32181560360324	5.06976603741948
H	-6.12766275937422	4.84039156520290	5.74507291428862
C	-4.61642230079161	3.32508255962758	5.53846591779940
H	-4.62642441846033	3.06471361771460	6.58853593549396

C	-3.76931435135812	2.65856660320435	4.67340101670495
H	-3.13008913897287	1.87604868461577	5.06086538983418
C	-6.32319491583328	5.72506796796845	3.17498565198172
B	1.46570060669932	-0.54477698961104	0.30454254731140
N	0.76167722946992	-0.52456257131062	-2.04354128004095
C	1.39938665905587	-1.22200935435045	-0.96814205928401
C	1.96584498105605	-2.53330991318744	-1.54922405169164
C	1.54861474253420	-2.46723671513927	-3.03377226811191
H	2.31889537217713	-2.88005237604659	-3.68646452443617
H	0.64335804132724	-3.05773838576347	-3.18962279065455
C	1.23751632605909	-1.00305703331798	-3.35400156765711
C	1.36619236071789	-3.79690454908359	-0.92720004692384
H	1.67572813350626	-3.92003497329697	0.10602319818594
H	0.27676304720658	-3.77428330808198	-0.95168096888660
C	3.49309470259495	-2.62419989486979	-1.44564516981318
H	3.98175552783432	-1.75217019081789	-1.87720286439492
H	3.81570010151791	-2.70564383711181	-0.41234062910570
C	0.17949748827674	-0.88850788891959	-4.44477973479453
H	-0.72009316137964	-1.44758212100237	-4.19167890987968
H	0.57929462614709	-1.29347855536495	-5.37540941146021
H	-0.10342221455651	0.14890703899983	-4.62492357304213
C	2.50411560188329	-0.26352986719342	-3.81555526395996
H	2.26876907675367	0.73718747828271	-4.17358636403352
H	2.98167875719403	-0.80588176475185	-4.63378226375316
H	3.22297201031260	-0.16884877527337	-3.00257705740704
C	-0.40126308813825	0.27997066734793	-1.88678408186550
C	-1.65715266826215	-0.32700157362776	-1.66789958119977
C	-2.78473291885139	0.47453801351930	-1.53181522702601
H	-3.74882167211168	0.01158957556295	-1.36032180405180
C	-2.70230110710644	1.85263854152054	-1.61938423944617
H	-3.59276111824700	2.46072685309887	-1.51843613461213
C	-1.47398928968708	2.44470783440882	-1.84236947828904
H	-1.41115864983771	3.52359919987229	-1.91693825885868
C	-0.31606062505237	1.68261895310614	-1.97595173122205
C	-1.83171977607334	-1.83224476278245	-1.59020698649831
H	-0.89665616263766	-2.27885211609724	-1.91610282452935
C	-2.93426510217335	-2.33830837520821	-2.52352771593851
H	-3.92421940753697	-2.02299235536897	-2.18924669284412
H	-2.92982596338968	-3.42938449263330	-2.54913988744697
H	-2.79723514302914	-1.97459355165351	-3.54270908725798
C	-2.08876281232742	-2.30351088516922	-0.15751523519290
H	-1.24202274039504	-2.06000724489853	0.48359834211994
H	-2.23231922895845	-3.38577728199907	-0.13220317464764
H	-2.98486049955031	-1.83540918840077	0.25649892461704
C	0.99174033779485	2.39942810962588	-2.25362295790805
H	1.77886839510350	1.64944668574867	-2.21230333317537
C	0.97811442747626	3.02564293466958	-3.65193577201706
H	0.71491173205506	2.29900268388038	-4.42098333611561
H	1.95788743744705	3.44010502537229	-3.89704748991452
H	0.24919776044969	3.83739301353578	-3.70520507022843
C	1.32331863436776	3.46710007621393	-1.21075498846189
H	0.55871654324340	4.24507815098420	-1.16663897832165
H	2.26899822386794	3.95102184578916	-1.46217187551778
H	1.42530831156229	3.02502914689998	-0.22243254018822
H	1.69884290233688	-4.67822711393411	-1.48244549545199
H	3.85066684069207	-3.51085541316488	-1.97527112874654

***cis*-TS-5**

F	-5.54928231987176	-1.90765660302661	0.81836422891230
F	-4.59321894473635	-2.93857768127832	-0.81329588347428
F	-4.01914414780976	-0.91411872304297	-0.33185631702061
O	1.55177139083947	-1.47765262156203	0.51968505177265
N	0.22767320316120	1.34643648308567	-0.58019952596227
C	1.41470484347091	-2.81851155876744	0.94057341453687
H	2.12740984670185	-3.41255976808629	0.37290732960535
H	0.40732593758071	-3.18414201038806	0.72974004942902
C	1.71724073525039	-2.87669130450671	2.44317869268716
H	1.39250444723765	-3.83888737354038	2.84064751608785
H	2.79235341823729	-2.80454940726990	2.59994255398123
C	1.03751292295807	-1.72022481310857	3.11603935496668
H	1.66335964211332	-1.01572330093947	3.65104427719979
C	-0.26838391302503	-1.42395017968223	3.00270108831392
H	-0.60485114487879	-0.51343577669872	3.47905218547755
C	-1.35364791491435	-2.30359358775831	2.49302926536939
C	-2.29456670182899	-1.81785729006365	1.58666854845328
H	-2.18189194527713	-0.81649220580771	1.19841039578835
C	-3.33045046640739	-2.62478941449796	1.15713965589286
C	-3.45410357329268	-3.93024045628501	1.61872663635774
H	-4.26471139292168	-4.55699812593942	1.27136971924996
C	-2.53205731672339	-4.41321803808831	2.52857085339662
H	-2.62252588904313	-5.42367260952041	2.90393695704639
C	-1.49422910412929	-3.60265437036315	2.96936201287279
H	-0.79273665935221	-3.98249663723748	3.70085414907698

C	-4.36539064083277	-2.09162546417729	0.20639489445311
C	0.25011324348851	0.82958288653506	0.75230858402038
C	-0.26378152999739	1.93153476413046	1.68851717130387
C	-0.28158083213892	3.16065394062031	0.75124027517001
H	-1.05389728419017	3.87743241352059	1.03420508742545
H	0.68141236858669	3.67266814093533	0.81361125566685
C	-0.45517002639190	2.63990331838105	-0.68132297174551
C	-1.67878616669658	1.71234446993693	2.24985124873247
H	-2.40158528465567	1.48579938348278	1.46908610732343
H	-2.01480832170442	2.61779117420299	2.76072624441300
H	-1.71950377877125	0.90356950838475	2.97625180859592
C	0.68912111764744	2.18297583990147	2.86148480106366
H	0.72402730818071	1.32784348193446	3.53904230201793
H	0.36684092151799	3.05116255173684	3.44285629047869
H	1.70245354771475	2.36476505119152	2.50188928951596
C	-1.93497926504637	2.50218221457167	-1.07889732763318
H	-2.01988700372268	2.22178215864548	-2.12886637655427
H	-2.45828272138441	3.45084668864915	-0.94332633748036
H	-2.44737713536597	1.74213603762379	-0.49267748961158
C	0.20625160370389	3.57025911189197	-1.69374001988059
H	1.24094431728263	3.78019514995231	-1.43120169421144
H	-0.33484005565748	4.51701305721736	-1.72502487157533
H	0.18477746966488	3.13926163216434	-2.69597498367820
C	0.99815433971440	0.74703417390499	-1.60977741471465
C	2.38102656312760	1.00447945508949	-1.70201063420621
C	3.11077924708415	0.40574284954059	-2.72202060965056
H	4.17308564229815	0.60231589314082	-2.80395087702853
C	2.50723292228868	-0.44174375220320	-3.63475899377785
H	3.09088331986800	-0.89205326642194	-4.42806188703375
C	1.16059555916741	-0.72496020625738	-3.51454548323265
H	0.69681079223281	-1.41322873864211	-4.21131803290412
C	0.39466612274725	-0.15686156107607	-2.50015166763276
C	3.11139132597153	1.87800767719632	-0.69841136366363
H	2.35786864379727	2.33968533048985	-0.06265827931243
C	3.92106134956120	2.99077815892936	-1.36769836663194
H	4.75705769410955	2.58629842646801	-1.94135389610577
H	4.33614773225046	3.66130372803307	-0.61301616422224
H	3.30979554132348	3.58325566223793	-2.04922111387683
C	4.01617268695553	1.03387592644848	0.20434930022031
H	3.44698084995761	0.24986888693237	0.70234004215093
H	4.48357312987291	1.66054853861528	0.96679420391534
H	4.81282331440501	0.56043673400415	-0.37428937841696
C	-1.05230794784816	-0.58395534300475	-2.34954900475316
H	-1.45862826317361	-0.04185019374930	-1.49805648577209
C	-1.14877351991581	-2.07773338610590	-2.03003903961750
H	-0.73000774385863	-2.68679027053021	-2.83374398364784
H	-2.19007223018752	-2.37389903553785	-1.89713501750684
H	-0.60904161393892	-2.31341721940666	-1.11246521350868
C	-1.88982858917788	-0.24351897342684	-3.58272103137804
H	-1.80854265361817	0.81278198633594	-3.84230464688909
H	-2.94209189925354	-0.47009226042287	-3.40136959470034
H	-1.56921939659373	-0.82264497416012	-4.45141272528746
B	0.74028027626250	-0.49148438348121	0.96553186135742

trans-TS-5

F	-6.03075258459515	-1.21610232212899	1.23869369578627
F	-4.47983519103439	-1.04659270716250	-0.25299010737415
F	-5.04813092860784	0.67390192970835	0.91852608991163
O	1.85701462564948	-1.84215951874270	0.31341889343576
N	0.37671683350050	0.98191035703433	-0.72496034565805
C	2.04516394385633	-3.12633017884914	0.86966309658672
H	2.99465405994583	-3.50086957133357	0.49213979338045
H	1.26257061998212	-3.81431433149248	0.53719011598506
C	2.07285342998249	-2.99445038070141	2.38895979688974
H	2.10300873253573	-3.98936617014737	2.84151540797764
H	2.98586990625902	-2.47818150913087	2.68986877353260
C	0.87333244780944	-2.22525354109968	2.86074056831283
H	0.95631675217892	-1.64420736271419	3.77214316885461
C	-0.29059506108078	-2.25265740419477	2.17468443201736
H	-0.36514198590075	-2.89497115879623	1.30492432516839
C	-1.60389702878611	-1.74770475597486	2.61605759023021
C	-2.57173072180022	-1.47995172871471	1.65060903368645
H	-2.32655336305682	-1.61720249954823	0.60680745863923
C	-3.81366190872182	-1.00429226805335	2.01919923352720
C	-4.12353390239178	-0.80147885342869	3.36045943032552
H	-5.09794963077652	-0.42559108197772	3.64326882498223
C	-3.17831241759323	-1.09890807913760	4.32469503722177
H	-3.41536283955079	-0.96212649910593	5.37116902390686
C	-1.92732237624827	-1.57527286171466	3.95544432879727
H	-1.19814791621643	-1.81260252750703	4.71954991239524
C	-4.83804109677608	-0.65465360947821	0.97788173591932
C	0.51717066735779	0.41921204615234	0.56827841846521
C	0.32524810964046	1.56190444068270	1.57935060474568

C	-0.01751582451805	2.76751864856062	0.66534973989951
H	-0.78285749024646	3.40738275537019	1.10698593568288
H	0.87573318761139	3.37835542658327	0.51711014878605
C	-0.44814863067202	2.19569823367007	-0.69067043533599
C	-0.75803867245035	1.36201187991121	2.63680558635190
H	-1.73247448038453	1.14798481288192	2.20079805402086
H	-0.84928769007801	2.26709277850905	3.24182565908672
H	-0.50227320932771	0.54587938252633	3.30969851404755
C	1.64564205895069	1.83402634698503	2.31669160429409
H	1.89865591746237	0.99978199687858	2.97434359407287
H	1.56958825563288	2.73883079114363	2.92736934848875
H	2.46688268489812	1.96523077909165	1.61168243774214
C	-1.95812668422563	1.90737284284336	-0.69030974613848
H	-2.29584112509796	1.55037639940204	-1.66204284301806
H	-2.51551282966125	2.81637222110143	-0.45708931245140
H	-2.21313011950768	1.15524071051709	0.05660340953812
C	-0.12369425026179	3.13160248930394	-1.84671700029430
H	0.92950495626363	3.41028117417877	-1.85081391772754
H	-0.71754518403767	4.04245360969859	-1.76120635321168
H	-0.35953232992893	2.66606510778197	-2.80534667253913
C	0.92607874612038	0.36598067800246	-1.87901553356562
C	2.25120012313986	0.66963469341969	-2.25036212461705
C	2.79465663309959	0.06495157700626	-3.37713703600638
H	3.81148669906253	0.29742739222077	-3.66956742229875
C	2.06125751706136	-0.83510258234828	-4.13012388382384
H	2.49740361614554	-1.29256960779692	-5.00942787218405
C	0.77547009148245	-1.15859196346800	-3.74283558306986
H	0.21169196778818	-1.88127073143595	-4.32083798203980
C	0.19585513310277	-0.58277473745398	-2.61526159416567
C	3.11757682664445	1.59769837660144	-1.42169520002833
H	2.46290954171002	2.08366664701556	-0.70082461635887
C	3.78864303996634	2.68760222785876	-2.25765671604382
H	4.51934125669934	2.27101630044739	-2.95352023657501
H	4.31730287008783	3.38782970235334	-1.60847652668086
H	3.05813317529562	3.25063274394796	-2.84050287966665
C	4.15623721609364	0.79578701231946	-0.63228581352009
H	3.67269141639155	0.03498590063986	-0.01900029374284
H	4.73309161430291	1.45345633501466	0.02141543626549
H	4.85523006979042	0.29415830384341	-1.30579610369815
C	-1.19087896443110	-1.03297223645603	-2.20073800406198
H	-1.43534533322952	-0.50635724601588	-1.28070979358910
C	-1.21981354943258	-2.53197435997586	-1.89183531860813
H	-1.01014534048112	-3.12945852703719	-2.78109008021421
H	-2.20392466475031	-2.82630281636572	-1.52078016130550
H	-0.47595549151518	-2.78793960107633	-1.13571463151727
C	-2.24231747364188	-0.68277595725439	-3.25524243225062
H	-2.21054484405526	0.37636590219705	-3.51373794528195
H	-3.24431002556445	-0.91408377923807	-2.88999435464525
H	-2.08127806157520	-1.25074759214910	-4.17395210185273
B	0.93527647870963	-0.93951829419796	0.73835071620298

TS-6

F	-5.58941643277806	-0.66020385805301	0.99262222885867
F	-3.84241958086644	-0.57155407463898	-0.25575831178342
F	-4.10064631818491	0.85606407658048	1.33425652412304
O	1.33240454421023	-1.61513326310863	0.11954918153922
N	0.31141504050615	1.06003407945421	-0.65649473594057
C	1.86475079836553	-2.80751322951386	0.75010964505593
H	2.68451766894580	-3.16646506184118	0.13141679282508
H	1.06967012337324	-3.55393594360767	0.77674915629908
C	2.26206236181778	-2.34394194964435	2.13924502454156
H	2.32845969800376	-3.16735865459968	2.84936992684859
H	3.23716318978036	-1.84843643777234	2.09160072881747
C	1.14236460646760	-1.33238466078104	2.52015258723884
H	1.56038806918934	-0.59853424165730	3.21838458645471
C	-0.01674416650692	-2.06895684727559	3.16311788168591
H	0.31486163723424	-2.69165129851632	3.99054989182193
C	-1.38138550146134	-2.07047207038805	2.94969687949890
C	-2.14665403711875	-1.35454167797642	1.96472917584061
H	-1.64410592617370	-0.75975466216042	1.20768088451743
C	-3.53692543939136	-1.35813737022554	1.97396624010041
C	-4.28689658325880	-2.10554961384058	2.86741337430041
H	-5.36511734105598	-2.09247203972655	2.85404697052671
C	-3.56820493843993	-2.89216362485778	3.78378623672601
H	-4.10989421545867	-3.51848588087574	4.48417213208769
C	-2.20598502528632	-2.88308110920250	3.82351801820526
H	-1.69557534620539	-3.49590251435974	4.55896145744437
C	-4.26528199856432	-0.45720576805329	1.02418676373590
C	0.47156517954304	0.72406843038245	0.57960066206200
C	0.25439050965812	1.89693426260668	1.50726373743510
C	-0.03620219393285	3.05747192552818	0.53292157828682
H	-0.86855455381547	3.66967299912750	0.87323558968457
H	0.83932898601636	3.70248808993290	0.45143254102150

C	-0.33878997246226	2.42907094450728	-0.82782133982984
C	-0.85613801170375	1.66556539022714	2.53476428830121
H	-1.83064314028472	1.55205900920764	2.06627702660745
H	-0.89325365675155	2.52776515881948	3.20131105427581
H	-0.66592078066004	0.77497397984606	3.13404498120753
C	1.57889944930810	2.15612798879958	2.24991572643974
H	1.79445626950610	1.38035419472857	2.98027223205855
H	1.48732220687517	3.10478005463730	2.77973614946801
H	2.42355061408008	2.23568213686491	1.56395477781874
C	-1.83776061611534	2.23266871640322	-1.03475716319975
H	-2.05269155110842	1.75367963195812	-1.98696871745376
H	-2.30827666079330	3.21530056104817	-1.04150148692581
H	-2.28802440000621	1.64682256006105	-0.23568603913541
C	0.24029518737718	3.20433145402358	-1.99807160594655
H	1.30135169249386	3.40694945941961	-1.87639946025758
H	-0.27715304776281	4.16168784900698	-2.05148016476492
H	0.08203906721891	2.68350691220826	-2.94179804684342
C	0.79824684843723	0.30600820239349	-1.80511219011817
C	2.13232505886024	0.53526809915776	-2.17549528498257
C	2.61807751080919	-0.15017200940971	-3.28065810807920
H	3.64474621444142	-0.00106572504218	-3.58720217778607
C	1.81876809718713	-1.02858116585144	-3.98876420215873
H	2.21742801375062	-1.55170738198297	-4.84817522126139
C	0.51812116077818	-1.24936758032816	-3.58561036611885
H	-0.09413341184821	-1.95898777153285	-4.12636842823662
C	-0.02199612438111	-0.59763998035766	-2.48074168623929
C	3.08804108363204	1.42939920838422	-1.39995739233682
H	2.51244726156440	2.05312136011179	-0.71746903545412
C	3.87601940561573	2.37606085308717	-2.30772199229219
H	4.58941154085358	1.83444923280342	-2.92949770786833
H	4.44320034763248	3.08327954660419	-1.70146899817827
H	3.21986304263166	2.94210580235942	-2.96947716384519
C	4.04395826575307	0.58796563225216	-0.54852665190889
H	3.50558770238607	-0.05749368538881	0.14492440688188
H	4.70637744440958	1.23432567167702	0.02901768020762
H	4.66096274226125	-0.05228535513161	-1.18114239480991
C	-1.42566732896049	-0.96065549687501	-2.04548640050159
H	-1.68993287487362	-0.34944096461329	-1.18717331589511
C	-1.50421935396165	-2.42194175882497	-1.59397929470297
H	-1.26830070606435	-3.10070365361631	-2.41565885275762
H	-2.51177963273374	-2.64620612055256	-1.24504906701489
H	-0.81410524244263	-2.62142823372186	-0.77634213679056
C	-2.44527432950595	-0.68929903037270	-3.15452945006248
H	-2.37641880720016	0.33159515962786	-3.53273672047383
H	-3.45604793140053	-0.84827948708648	-2.78054917799457
H	-2.29306008849618	-1.36297537076515	-3.99936096943557
B	0.93475862704130	-0.74957200970749	1.07303673853456

***cis*-TS7**

F	2.76321279605959	-5.71089599866585	-1.31130815210694
F	1.04531702237123	-4.41197506572183	-1.25600675366122
F	1.81859157899792	-5.09468022804513	-3.15124955841379
O	1.32723013017791	0.32743808042005	0.29734081231989
C	0.54386390034569	0.93024888790945	-2.16142181851328
H	1.24891213360472	1.62522470610006	-1.72813431888036
C	-0.33815652321556	1.47136850431973	-3.11795991422178
H	-0.53001483473332	2.53780981193570	-3.10925172990955
C	-0.91231430576845	0.69248418156255	-4.10424632691014
C	-1.81453421392935	1.23761544814301	-5.07517670397039
H	-2.06418782698536	2.28991188740495	-5.01375080092231
C	-2.34869589052949	0.45344622840290	-6.04005542850684
H	-3.03400703537485	0.87257315020169	-6.76522979928946
C	-2.01651720048761	-0.93446314153565	-6.11532121961555
H	-2.45603116575989	-1.54083542798750	-6.89657553646869
C	-1.16294465727361	-1.48917286410650	-5.22159104768955
H	-0.91318078558846	-2.54192269792060	-5.27223235394106
C	-0.58072993413440	-0.70157702604382	-4.18070041041920
C	0.28243991841301	-1.24543765672304	-3.24031665075464
H	0.54399550114279	-2.29350209548505	-3.31041654699886
C	0.85149606742073	-0.46638916074139	-2.23864092814762
H	1.65222522287868	-0.88202680689562	-1.64745992718510
C	2.14413815726022	1.45458195760782	0.52818254954592
H	2.14713794733549	1.65842420640127	1.60217472286253
H	1.74176482640866	2.34377565676733	0.03764248611164
C	3.56603979921008	1.20103881943364	0.03254686748175
H	3.52596120584165	0.91308069055972	-1.01803880479061
H	4.11211637296920	2.14644344982484	0.09061001018361
C	4.27051087392403	0.17119422450470	0.85987439486532
H	4.51369652614366	0.48204000355750	1.87219608217118
C	4.54819979369113	-1.08451410689808	0.52624430131352
H	4.98309431957379	-1.73323589611153	1.27978199820324
C	4.23069905692914	-1.72875007837765	-0.76747535879411
C	3.43062398667049	-2.86589364643426	-0.77972403506555

H	3.05684546877629	-3.26658216502427	0.15446406947475
C	3.08046558959755	-3.46859399911217	-1.97933356033507
C	3.53958448037420	-2.96457632552861	-3.18556618704916
H	3.26314593899972	-3.43718337450217	-4.11717092575439
C	4.35628174276369	-1.84393601613699	-3.17957847595385
H	4.72577546586777	-1.44439878343023	-4.11449533245943
C	4.69849115036741	-1.23359150002110	-1.98434767504238
H	5.34214137171551	-0.36327973508569	-1.98892969822502
C	2.18030446096917	-4.67050852580087	-1.93144634090651
B	0.09767275571864	0.32965377948308	-0.34943681654717
N	-1.52406527317845	0.31914884415683	1.54311567547193
C	-1.25375156000514	0.14740278257386	0.16126684926859
C	-2.60167534125506	-0.15044388263869	-0.49793605640836
C	-3.44436400283626	-0.65616804377093	0.68356089267486
H	-4.50756199378156	-0.45874815897205	0.53736722735382
H	-3.31947234945823	-1.73852173254880	0.75936350995784
C	-2.90735061013071	-0.00958126451439	1.95641439909026
C	-2.59416090784935	-1.24570456238913	-1.55848621024160
H	-2.19201402301610	-0.89683687399518	-2.50109994548495
H	-2.00918855318523	-2.10645914677814	-1.23189017260178
C	-3.20744422169762	1.12252635604636	-1.10833997389369
H	-3.29926740996254	1.92425496598452	-0.37831713885795
H	-2.58722205836346	1.48885545092438	-1.92539822012572
C	-2.96310653986242	-0.99837442275876	3.12232111129554
H	-2.44202737895196	-1.92480577317991	2.89127410006762
H	-4.00485138940064	-1.24297926006274	3.33588342836384
H	-2.53331527979101	-0.56512903609075	4.02613968121124
C	-3.73835247815285	1.20663508296787	2.38935359721340
H	-3.27029759975002	1.70909770077803	3.23627392637066
H	-4.72421597615622	0.86847206892624	2.71104834455395
H	-3.88331808169378	1.93174856151572	1.59289010659797
C	-0.59682258287646	0.92283725380621	2.44510847944581
C	0.24940727619666	0.11759159782239	3.23406972285307
C	1.12736029116115	0.73007049962912	4.12275167910270
H	1.77765861377255	0.11505149721219	4.73307086133126
C	1.19810024314265	2.10659971049102	4.23278230294731
H	1.88738718532242	2.56414323232895	4.93144863162249
C	0.39332499732135	2.89286180935712	3.43108963108367
H	0.46485875874232	3.97144158624339	3.50221152139424
C	-0.50354131878452	2.32764253938538	2.52884162160150
C	0.28414909657616	-1.39843767864751	3.12708442724570
H	-0.47685966261228	-1.68764215782953	2.40557524071487
C	-0.01181467039391	-2.08032007857400	4.46608562386239
H	0.79522789545672	-1.90372690190411	5.18013633322317
H	-0.09520523122129	-3.15992130768984	4.32797189024627
H	-0.93661166282871	-1.72460596329164	4.91751533951048
C	1.62539436550136	-1.89767590783658	2.58578260481972
H	1.83860775015629	-1.45466129140463	1.61644730478673
H	1.60372550386313	-2.98499543122019	2.48301641142873
H	2.44545216668739	-1.64510259126062	3.26252641751583
C	-1.33023647477972	3.26671881720877	1.66501190904708
H	-2.04779064641594	2.65919949198085	1.12471685035540
C	-2.10016897289845	4.29200543995537	2.50177486049041
H	-2.66840906398061	3.81954826452317	3.30247310529235
H	-2.79861289598820	4.84355936498228	1.86990905208948
H	-1.42660160128981	5.02013693954382	2.95766649727371
C	-0.48662705233069	3.98716913253334	0.61076293946823
H	0.34492524749576	4.53166677228499	1.06373782442215
H	-1.10250086528098	4.70594505892718	0.06661333258732
H	-0.08560105565473	3.27907608638372	-0.11258598939009
H	-4.20301338145648	0.91489048335212	-1.50919773301198
H	-3.61879941286399	-1.57588927667150	-1.74767698232121

cis-TS-7 (Configuration 2)

F	4.26075630853315	-4.10057261892957	3.10687698598717
F	5.67225075062540	-5.21396424154825	1.92006006893464
F	6.37506061278738	-3.72963327071558	3.31990743149640
O	0.43421499332622	-0.51245542162145	-1.20295514875767
C	-0.81817303057015	1.18861130526578	-2.81713183185971
H	0.23545727011242	1.39482928205673	-2.69213641835239
C	-1.63048003031026	2.30228607954409	-3.10530040279870
H	-1.27475542512005	3.29158658340835	-2.84275726373616
C	-2.83844076980905	2.17482238903249	-3.76451230903600
C	-3.67254031786548	3.30555910829322	-4.04567158566165
H	-3.34719315530269	4.28265049640571	-3.70971059373326
C	-4.84367241157717	3.15412395835815	-4.70665237293365
H	-5.47035914211035	4.01297338899285	-4.90862153448719
C	-5.26766233672141	1.86256197971780	-5.14643981182018
H	-6.20747772242048	1.76679584966310	-5.67441266747358
C	-4.50597549429208	0.76789501771047	-4.90787293781340
H	-4.82757623364726	-0.21279855326321	-5.23647158631385
C	-3.26596572008684	0.87836100167050	-4.20606537136557
C	-2.46986000519288	-0.22599428037274	-3.93314947480954

H	-2.78002128481170	-1.20156105900962	-4.28676249422228
C	-1.26487458932359	-0.10218019018105	-3.25087894043132
H	-0.59944241722243	-0.94962541013147	-3.21111302280493
C	1.71990161830886	0.07011758834290	-1.26527662145478
H	2.27227311263267	-0.25661585646578	-0.38159941880344
H	1.66176997031803	1.15955621008781	-1.21931853745160
C	2.45876253481921	-0.34751667815961	-2.53545456984440
H	1.88054795816018	-0.02742429935394	-3.40378976216800
H	3.39382260482917	0.21212634561232	-2.57779009178866
C	2.69832049878992	-1.83039598901530	-2.63914864057047
H	2.11296295032662	-2.34173464038252	-3.39559343548109
C	3.48569845618786	-2.60930614926016	-1.89837434675609
H	3.44658101387502	-3.67383664539650	-2.10670879129365
C	4.36189076872984	-2.26126811457893	-0.76293446641235
C	4.53967541884384	-3.21564148067192	0.23535402205025
H	4.04704341795245	-4.17601109964490	0.14527691625498
C	5.32532183151443	-2.94977629673804	1.34596885849233
C	5.97691171034886	-1.73450356260187	1.47541706837024
H	6.59133789549830	-1.53058737326606	2.34073584821139
C	5.83587832740831	-0.78974541110659	0.47031548165822
H	6.35250508515637	0.15752967738064	0.54852700574531
C	5.03887839119682	-1.04704308226187	-0.63137024552286
H	4.95996417260073	-0.29733318335495	-1.40454065208114
C	5.41769902925226	-3.99418472644248	2.42154616388384
B	-0.77011863784723	0.17306552154706	-1.13831616315013
N	-1.28730968621060	0.14919577574373	1.29233272030100
C	-1.71405869533253	0.35162125742081	-0.04488865974220
C	-3.18124128667296	0.77664124972597	0.03105762677587
C	-3.60339702559806	0.25508834286990	1.41389836196592
H	-4.40660162784438	0.85418095647719	1.84596543346841
H	-3.97957777883608	-0.76395863824875	1.30095221769590
C	-2.36506025072557	0.22493634937293	2.30552025211499
C	-4.09891907015418	0.13886069623672	-1.00634937866494
H	-3.97506496743276	0.58146990544382	-1.98713677957900
H	-3.91327052101939	-0.93287675993084	-1.08717148068471
C	-3.33342359781172	2.30308630628703	-0.05197972665242
H	-2.77820063905949	2.81659918590601	0.72995773686450
H	-2.97741814940436	2.67621918193909	-1.01080304103966
C	-2.40582776445356	-0.99406643607677	3.22837038436265
H	-2.50709079818648	-1.92143504107768	2.66857604768366
H	-3.26417546106669	-0.90872223945454	3.89687594853318
H	-1.50981234225064	-1.04854942134236	3.84688472735108
C	-2.27189906711071	1.45174053846407	3.22322096823519
H	-1.32531943305378	1.45322777178127	3.76456872587765
H	-3.07365293219499	1.40749273711361	3.96140965457864
H	-2.36671834922358	2.39403615591532	2.69040686742080
C	0.09110359144005	0.12200039796448	1.66845561826843
C	0.73876008504787	-1.10864725393181	1.89920802518548
C	2.07012026415844	-1.10626684076574	2.30474580584220
H	2.57016205837899	-2.04869734963289	2.48992215625926
C	2.77552878714580	0.07232368354856	2.45368790810364
H	3.81182852491924	0.05067409948423	2.76570673200535
C	2.15180694765934	1.27438728854688	2.18036164858443
H	2.71166044759523	2.19686649515444	2.27705248090647
C	0.81765363388976	1.32607578150325	1.78854233801514
C	0.07005357836367	-2.45458588524257	1.67255704853385
H	-0.96039101778879	-2.25928175862293	1.38417813033374
C	0.08333724399885	-3.32915531507468	2.92985567270335
H	1.10064901254017	-3.64075375131588	3.17544973966803
H	-0.50457007664013	-4.23405418070832	2.76505766368687
H	-0.32393011907395	-2.81509945642332	3.79897789869093
C	0.72574374913322	-3.21942397895496	0.52028114245029
H	0.70967988254461	-2.63417385163008	-0.39588401218731
H	0.19818233949042	-4.15991239502253	0.34802814357172
H	1.76414683235786	-3.46084187035875	0.75587309040255
C	0.21490360725135	2.69356870926675	1.50811476692162
H	-0.84411753874098	2.54750943256574	1.32727702390130
C	0.37397363881741	3.64627076687708	2.69653100848026
H	0.03149565029434	3.19605676380967	3.62753132743052
H	-0.20336865119735	4.55713020925972	2.52740732671563
H	1.41632787083586	3.94024524885706	2.83341887907966
C	0.78941197719759	3.34354146650870	0.24740175456241
H	1.87755716813287	3.42472326149635	0.29657876844479
H	0.38304438183192	4.34978251184830	0.12706801908115
H	0.52326853559549	2.77115662016879	-0.63964454545282
H	-5.14123553263236	0.28791729079346	-0.71297217399586
H	-4.38564640680748	2.58213583684883	0.04640766704530

trans-TS-7

F	-6.37348565053224	6.06849851095151	2.63316779828435
F	-7.33448858315862	5.76433372180323	4.54110889725618
F	-7.53856295357229	4.29056356108745	2.97896423165960
O	1.39970994398483	0.90812632160530	0.60458409422330

C	3.07099670373628	-0.93857503480906	1.75086772797011
H	3.29879735919042	0.11590124471712	1.78487502789203
C	4.11288816340215	-1.85542236736391	1.71304382311099
H	5.12708122183690	-1.50121911043720	1.57564008675005
C	3.88918371147058	-3.21660475731047	1.88941212622520
C	4.95053418161077	-4.17103256203587	1.84402040669044
H	5.95539357146732	-3.81743267443510	1.64819816441652
C	4.70475408642003	-5.49021650224052	2.03490451830982
H	5.51551660737239	-6.20580341876306	1.99352091850482
C	3.37935865388192	-5.95323257174525	2.29432050324964
H	3.20937636824522	-7.01120142441870	2.44551870121848
C	2.34461557952366	-5.08190609852085	2.34946584716872
H	1.33613772077905	-5.42827564188892	2.53941020883029
C	2.55646899984222	-3.68150376358495	2.13936648747279
C	1.51719673747166	-2.77064979505751	2.16942257895962
H	0.51800666660421	-3.11562254611716	2.40625405609626
C	1.73108786770300	-1.39827520423532	1.95279903556925
H	0.98314016151039	-0.74195880739905	2.37110549522599
C	0.59994946372587	1.43216971410799	1.63907522605375
H	0.65288720415426	2.51842847165498	1.54767820939039
H	1.03137568210810	1.18616998030476	2.61899193983814
C	-0.86046833889210	1.00392082537860	1.57549718579727
H	-1.26777762439017	1.29412542659168	0.60980861242206
H	-0.92218678852148	-0.08629574549718	1.63301042264125
C	-1.66057688795114	1.61475587322881	2.67932016410485
H	-1.39384569700426	1.32215348708114	3.69184120278249
C	-2.64971002168338	2.47977742815564	2.47617470611946
H	-2.90348766107876	2.73570572285748	1.45085699849689
C	-3.47912178958465	3.12571755552299	3.50661294100721
C	-4.62307401085337	3.81099501549072	3.11270780359170
H	-4.87836041646357	3.86101530812333	2.06097173505177
C	-5.44145295962233	4.42811635573597	4.04935229951790
C	-5.13413432488739	4.37785896545017	5.39725112783174
H	-5.77177156782000	4.86090554743858	6.12365742515699
C	-3.98837617551408	3.70344131571878	5.79999316410862
H	-3.73132421773191	3.66510931271995	6.85024095343291
C	-3.17164598320352	3.08927298951783	4.87006367412288
H	-2.27657593800445	2.58299789269692	5.20722036715645
C	-6.67084583560134	5.13975532521607	3.55942172223754
B	1.59483709297881	-0.41969175649530	0.24448393573476
N	0.92074133611199	-0.27004894010590	-2.12139158054716
C	1.59744908787601	-0.99010358338574	-1.09276812826516
C	2.24071472077279	-2.22660387622376	-1.74912784485781
C	1.76251892823130	-2.13761028473936	-3.21163520478196
H	2.52562041575580	-2.48962585174324	-3.90713919208115
H	0.88308873142999	-2.77033209077911	-3.34973576826339
C	1.36202048025805	-0.68439515460902	-3.46889411218446
C	1.77262983592150	-3.56506245239322	-1.17093179040164
H	2.19759941392884	-3.75440244410510	-0.19209749383317
H	0.68673057740829	-3.60778066862047	-1.08156509615057
C	3.77435964605578	-2.19168597581531	-1.69922665130445
H	4.17465270199943	-1.29042170223961	-2.15963988066566
H	4.13524006083751	-2.22888656073940	-0.67521384111663
C	0.25394413214103	-0.59627845518650	-4.51184386209949
H	-0.60279591258773	-1.21130706308021	-4.24131953378275
H	0.63690498745179	-0.95175691109417	-5.46944406347552
H	-0.09189668560004	0.42848740219328	-4.64859577665540
C	2.56278556811918	0.14067417920610	-3.96092857282055
H	2.25285537128170	1.13115747010902	-4.28909883719293
H	3.03935542847519	-0.35580259957197	-4.80833949097315
H	3.30546911478862	0.26567900402632	-3.17447631323414
C	-0.28558231973141	0.45827238498338	-1.91185494704103
C	-1.49311616382520	-0.22936232595892	-1.66222156299834
C	-2.66521986445737	0.49829339494820	-1.49101361850121
H	-3.59266885697979	-0.02674138019111	-1.29748623957400
C	-2.67449987734381	1.87893902327792	-1.57565902862078
H	-3.59916859585494	2.42823323496921	-1.44902519295890
C	-1.49268122492002	2.54884855470638	-1.82778968016840
H	-1.50012285343626	3.62993572664159	-1.89754595952990
C	-0.29199855460587	1.86326281156754	-1.99353815965555
C	-1.57376410979229	-1.74381020190769	-1.59551193795002
H	-0.61915140754350	-2.13157066658877	-1.93920115563390
C	-2.65914828427925	-2.30956266803006	-2.51480979039607
H	-3.66025602678050	-2.05328532828884	-2.16377739408092
H	-2.59169408558202	-3.39848389681108	-2.54551237108221
H	-2.56001719026768	-1.93524244815615	-3.53441447298086
C	-1.77929349345328	-2.24161607311934	-0.16368358212832
H	-0.94553482612180	-1.93843136892036	0.46875369738358
H	-1.84139268192018	-3.33181530178158	-0.14407517036513
H	-2.70294253939062	-1.84417658805787	0.26325908413710
C	0.96083778872353	2.66591918858121	-2.28892469571278
H	1.79214651533991	1.96467220781800	-2.29771491353191
C	0.86673057030252	3.33819365266827	-3.66233896951324

H	0.62152485507087	2.62447394092859	-4.44938432094489
H	1.81317184600208	3.81690797154648	-3.92097840699927
H	0.09199357153744	4.10812314236143	-3.66585387682768
C	1.26253329427223	3.71270508645504	-1.21630445510262
H	0.45026960540910	4.43473751476478	-1.11187334161520
H	2.16420027905402	4.26827689514763	-1.48116520204438
H	1.43086582280005	3.23828047881850	-0.25249822784720
H	2.09092036886955	-4.37847888288763	-1.82818634213083
H	4.18505017529860	-3.05428761138901	-2.23045928257942
