# Supplementary material for: Aromatization-Driven Borylene Generation from Borepin Precursors Enables Fused Borirane Synthesis and Selective Alkene Functionalization
Source: J Am Chem Soc. 2026 Aug 25;148(35):38316–27. doi: 10.1021/jacs.6c11662 (PMC13564424; doi:10.1021/jacs.6c11662)
Supplement: Supplementary file 2 [file ja6c11662_si_002.pdf]

SUPPORTING INFORMATION

Aromatization-Driven Borylene Generation from Borepin Precursors Enables Fused Borirane Synthesis and Selective Alkene Functionalization

Jay Prakash Maurya[#a], Dmitrii Bushmin[#a], Jonathan R. Church[#b], Kartick Dey[a], Yael Diskin-Posner[b], Liat Avram[b], Linda J. W. Shimon[b], and Samer Gnaim[*a]

[a] Department of Molecular Chemistry and Materials Science, Weizmann Institute of Science, Rehovot 7610001, Israel.

[b] Department of Chemical Research Support, Weizmann Institute of Science, Rehovot 7610001, Israel.

# These authors contributed equally to this study.

TABLE OF CONTENTS

1.	**Experimental details**			**S4**
	1.1.	Materials and Methods		S4
	1.2.	Synthetic procedures		S6
			Compound **S1**	S6
			Compound **1**	S6
			Compound **2**	S6
			Compound **3**	S9
			Compound **4**	S11
			Compound **^{Cyh}CAAC**	S13
			Compound **^{Me}CAAC**	S13
			Compound **5b**	S13
			Compound **5d**	S13
			Compound **5h**	S14
			General procedure for Sonogashira coupling and cis-reduction	S15
			Compound **5a**	S16
			Compound **5c**	S16
			Compound **5e**	S17
			Compound **5f**	S17
			Compound **5g**	S18
			Compound **13**	S19
			Compound **16**	S22
			Compound **18**	S24
			Compound **20**	S25
			Procedure for the borylene insertion into C_{sp3}-H of ^{Cyh}CAAC: Compound **3a**	S26
			Procedure for the borylene insertion into C_{sp3}-H of ^{Me}CAAC: Compound **5b**	S28
			General procedure A: Preparation of alkoxy borepin **6** with tethered alkenes	S30
			General procedure B: Intramolecular [2+1] cycloaddition of borylene with alkene	S30
			Compound **6a**	S31
			Compound **6b**	S33
			Compound **6c**	S35
			Compound **6d**	S36
			Compound **6e**	S37
			Compound **6f**	S38
			Compound **6g**	S39
			Compound **6h**	S40
			Compound **6i**	S41
			Compound **7a**	S42
			Compound **7c**	S46
			Compound **7d**	S47
			Compound **7e**	S48

			Compound **7f**	S50
			Compound **7g**	S52
			Compound **7h**	S53
			Compound **8**	S54
			Compound **9**	S56
			Compound **10**	S58
			Compound **11**	S60
			Compound **12**	S62
			Compound **14**	S64
			Compound **15**	S67
			Compound **17**	S69
			Compound **19**	S71
			Compound **21**	S73
**2.**	**Additional Data**			**S75**
	2.1.	X-ray data collection and structure refinement		S76
	2.2.	Low-temperature NMR experiment		S79
		Borirane-naphthalene intermediate		S82
	2.3.	Stability test of compounds **3** and **6g**		S86
	2.4.	X-ray structure plane of compound **3** and **4**-DMAP adduct		S90
	2.5.	Procedure for a formation of **Int-3'**		S91
**3.**	**Computational Details**			**S93**
	3.1.	Geometry Optimizations		S93
	3.2.	Thermochemistry and Free Energy Calculations		S94
	3.3.	Energy profile for alternative borylene formation from **Int-2**		S96
**4.**	**Notes**			S101
**5.**	**NMR and HRMS spectra**			S110
**6.**	**References**			**S299**

1. Experimental details

1.1. Materials and Methods

All air- and moisture-sensitive reactions were performed under an inert nitrogen atmosphere using standard Schlenk line or glovebox techniques. All solvents were degassed by purging with nitrogen and stored over activated 4 Å molecular sieves or metallic Na. All the glassware used for such reactions was oven-dried overnight at 140 ^{0}C or flame-dried prior to use. Other reactions were carried out in the open air, as noted in each case.

Reagents were purchased at the highest commercial quality and used without further purification unless otherwise stated. Isolated yields refer to chromatographically and spectroscopically (^{1}H NMR) homogeneous material unless otherwise stated. Reactions were monitored by thin-layer chromatography (TLC) on 0.25 mm E. Merck silica plates (60 F_{254}), using short-wave UV light (254 nm) for visualization and *p*-anisaldehyde or potassium permanganate as developing agents. Flash column chromatography was performed using E. Merck silica gel (60, particle size 0.043–0.063 mm).

NMR spectra were recorded on Bruker Avance NEO-300, Avance NEO-400, Avance III HD-500, and chemical shifts for ^{1}H- and ^{13}C NMR are reported relative to the solvent peaks (for C_6D_6, ^{1}H = 7.16 ppm, ^{13}C = 128.06 ppm; for $CDCl_3$, ^{1}H = 7.26 ppm, ^{13}C = 77.16 ppm). The following abbreviations were used to explain NMR peak multiplicities: s = singlet, d = doublet, t = triplet, q = quartet, p = pentet, sept = septet, m = multiplet, br = broad, brs = broad singlet.

ESI-QTof-HRMS: The analyses were carried out on a Waters Xevo G2-S Q-Tof mass spectrometer (Manchester, UK) equipped with an electrospray ionization (ESI) source.

The sample was diluted/dissolved in acetonitrile and directly injected via a bypass into a Waters Acquity UPLC system using 0.1% aqueous formic acid in acetonitrile:water (95:5) at a flow rate of 100 µL/min.

Spectra were recorded in positive-ion mode over a mass range of 100-2000 m/z. The parameters were set as follows: capillary voltage at 3 kV, cone gas flow at 50 L/h, source temperature at 120 °C, and cone voltage at 20 V (+). The desolvation temperature was set at 280 °C, and the desolvation gas (N_2) flow rate was set at 800 L h^{-1}.

Mass accuracy was maintained using a lock spray with leucine enkephalin (m/z = 556.2771 in positive mode) at a concentration of 200 ng mL^{-1}, delivered at a flow rate of 10 µL min^{-1}, acquired every 10 s for 1 s. Data acquisition and processing were performed using Waters MassLynx v4.2

software.

ASAP-MS: The analysis was carried out on a Waters Xevo G2-S Q-Tof mass spectrometer (Manchester, UK) equipped with an ASAP-MS probe (Waters).

Samples were introduced to the source via borosilicate glass capillaries (Waters, US), which were loaded by dipping the sealed end of the capillary into the sample solution to a fixed depth of 10 mm to ensure consistent loading.

The ASAP probe conditions were set as follows: N_2 elution gas temperature gradient from 50 to 500 °C at 20 °C min^{-1}; cone voltage, 40; corona discharge, 2 μA. Data were acquired and processed using MassLynx v.4.2 (Waters) over a mass range of 100-1200 Da.

GC-MS (EI) was performed on an Agilent 7820A GC system and a 5975 Series MSD.

1.2. Synthetic procedures

Compound S1

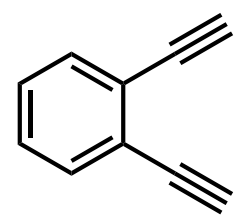

Compound **S1** was prepared following the reported literature procedure.[1]

^{1}H NMR (400 MHz, Chloroform-*d*) δ (ppm): 7.54 (dd, J = 5.7, 3.4 Hz, 2H), 7.33 (dd, J = 5.8, 3.4 Hz, 2H), 3.36 (s, 2H). Data are consistent with the literature values.[1]

Compound 1

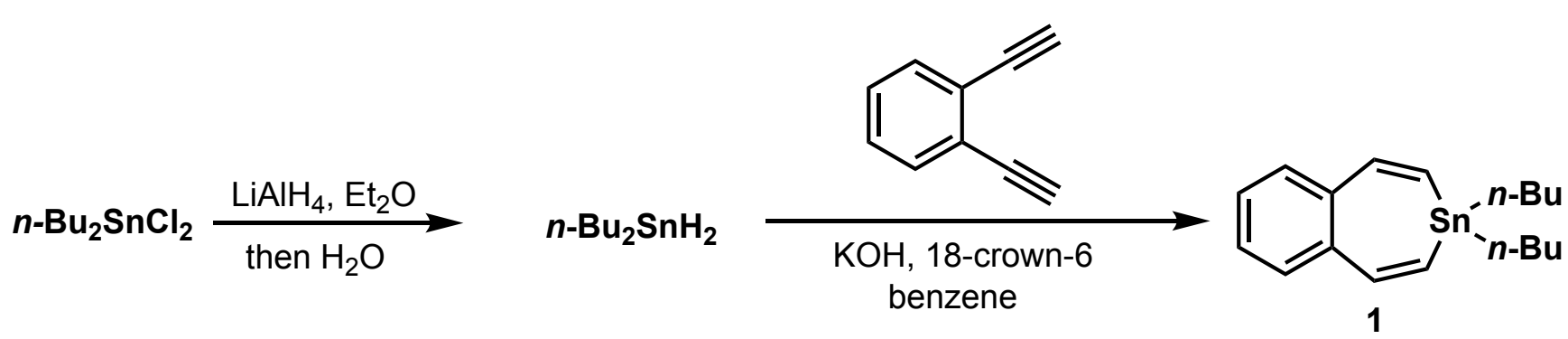

Step 1: In a nitrogen-filled glovebox, an oven-dried round-bottom flask was charged with $LiAlH_4$ (900 mg, 22.3 mmol, 1 equiv.) and 30 mL of dry diethyl ether. The flask was sealed, removed from the glovebox, and placed in an ice bath at 0 ^{0}C under a nitrogen atmosphere. Dibutyltin dichloride (7.2 g, 22.3 mmol, 1 equiv.) was dissolved in dry diethyl ether and added dropwise to the stirred $LiAlH_4$ solution. The reaction mixture was stirred at 0 ^{0}C for 1 hour, then allowed to warm to room temperature, where it was stirred for an additional 1 hour. After completion, the reaction was carefully quenched with degassed water (10 mL) and extracted with degassed diethyl ether. The combined organic layers were washed with water, dried over $MgSO_4$, filtered, and concentrated in vacuo. Dibutyltin dihydride was obtained as a colorless oil (4.6 g) and stored under a nitrogen atmosphere at -30 ^{0}C without further purification.

Note: The workup must be performed promptly after exposing the reaction mixture to air. Dibutyltin dihydride is air-sensitive and slowly decomposes upon exposure to air. Therefore, it is recommended to store the compound under an inert atmosphere at low temperature.

Step 2: In a nitrogen-filled glovebox, an oven-dried 250 mL round-bottom flask equipped with a stir bar was charged with dialkyne **S1** (1890 mg, 15 mmol, 0.66 equiv.) and 80 mL of benzene. KOH (84 mg, 1.5 mmol, 0.1 equiv.) and 18-crown-6 (200 mg, 0.75 mmol, 0.05 equiv.) were added sequentially. To this mixture, dibutyltin dihydride (3.88 g, 16.5 mmol, 1.1 equiv.) dissolved in 5 mL benzene was added dropwise. The reaction mixture was then stirred for 2 h at room temperature. Upon completion, the reaction mixture was quenched with 100 mL of water, and the organic phase was separated. Next, the organic layer was washed with distilled water, and the aqueous phase was extracted with hexane (2 × 50 mL). All organic layers were combined, dried over Na_2SO_4, and concentrated under reduced pressure. The crude was purified by column chromatography (100 % hexane) to afford compound **1** as a colorless oil (3.80 g, 10.5 mmol, 70% yield).

^{1}H NMR (300 MHz, Chloroform-*d*) δ (ppm): 7.63 (d, J = 13.9 Hz, 2H), 7.32 – 7.26 (m, 2H), 7.22 – 7.17 (m, 2H), 6.47 (d, J = 13.9 Hz, 2H), 1.44 – 1.34 (m, 2H), 1.29 – 1.17 (m, 2H), 0.94 – 0.89 (m, 2H), 0.81 (t, J = 7.2 Hz, 3H).
^{13}C{^{1}H} NMR (75 MHz, Chloroform-*d*) δ (ppm): 146.3, 139.5, 137.3, 132.2, 125.7, 28.9, 26.9, 13.6, 10.0.
HRMS (ASAP): m/z calcd for $C_{18}H_{27}Sn$ $[M+H]^+$: 363.1135; found: 363.1150.

Compound 2

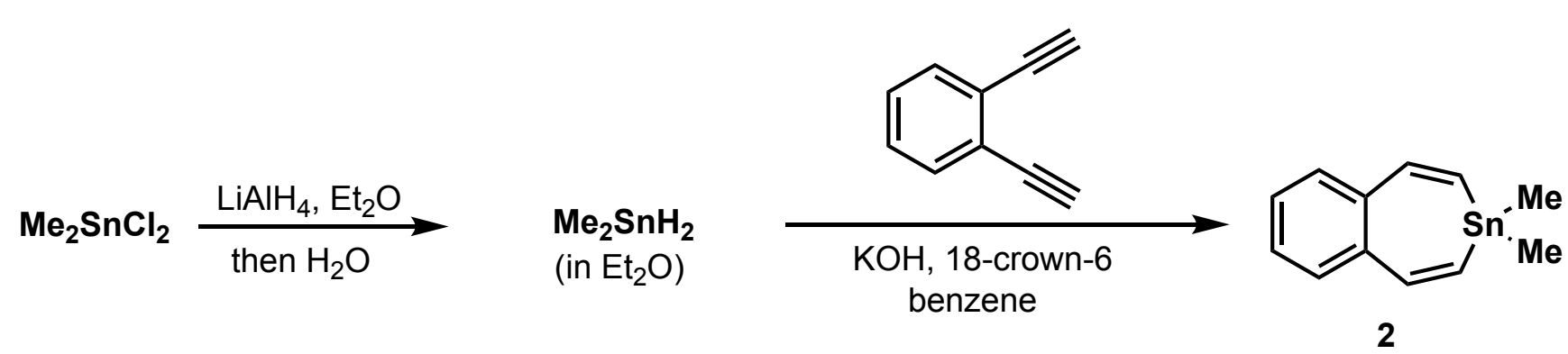

Step 1: In a nitrogen-filled glovebox, an oven-dried round-bottom flask was charged with $LiAlH_4$ (945 mg, 24.9 mmol, 1 equiv.) and 30 mL of dry diethyl ether. The flask was sealed, removed from the glovebox, and placed in an ice bath at 0 ºC under a nitrogen atmosphere. Dimethyltin dichloride (5.7 g, 24.9 mmol, 1 equiv.) was dissolved in 10 mL dry THF and added dropwise to the stirred $LiAlH_4$ solution. The reaction mixture was stirred at 0 ˚C for 1 hour, then warmed to room temperature and stirred for an additional 1 hour. After completion, the reaction was carefully

quenched with degassed water (10 mL) and extracted with 10-15 mL of degassed diethyl ether. The diethyl ether solution of dimethyltin dihydride was degassed with N_2 and used directly for the next step.

Note: The workup must be performed promptly after exposing the reaction mixture to air. Dimethyltin dihydride is air-sensitive and volatile, and decomposition begins if it is exposed for more than 15 minutes. Due to its volatility and sensitivity, the ether solution was used directly in the subsequent step without isolation.

Step 2: In a nitrogen-filled glovebox, an oven-dried 250 mL round-bottom flask equipped with a stir bar was charged with dialkyne **S1** (1890 mg, 15 mmol, 0.66 equiv.) and 80 mL of benzene. KOH (840 mg, 15 mmol, 0.66 equiv.) and 18-crown-6 (790 mg, 3 mmol, 0.13 equiv.) were added sequentially. To this mixture, the previously prepared diethyl ether solution of dimethyltin dihydride was added slowly. Next, the reaction was stirred overnight at room temperature outside the glovebox. Upon completion, the reaction mixture was diluted with EtOAc (30 mL), filtered through Celite, and the filtrate was washed with water (30 mL), dried over Na_2SO_4, and concentrated under reduced pressure. The crude product was purified by column chromatography (hexane, 100 %) to obtain compound **2** as a colorless oil (2 g, 7.19 mmol, 48% yield).

^{1}H NMR (300 MHz, Chloroform-*d*) δ (ppm): 7.60 (d, *J* = 13.9 Hz, 2H), 7.33 – 7.28 (m, 2H), 7.24 – 7.19 (m, 1H), 6.46 (d, *J* = 13.8 Hz, 2H), 0.13 (s, 6H).
^{13}C{^{1}H} NMR (75 MHz, Chloroform-*d*) δ (ppm): 146.3, 139.2, 137.5, 132.3, 125.7, -10.6.
HRMS (ASAP): m/z calcd for $C_{12}H_{15}Sn$ $[M+H]^+$: 279.0196; found: 279.0189.

Compound 3

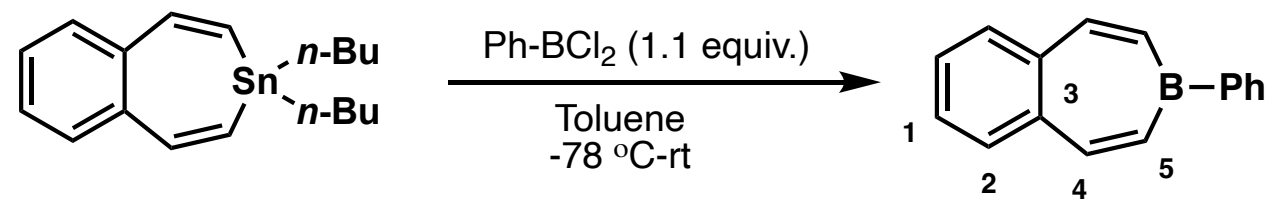

An oven-dried 100 mL Schlenk flask equipped with a stir bar was charged with stannepin **1** (2.0 g, 5.5 mmol, 1.0 equiv.) and dry toluene (20 mL) in a nitrogen-filled glovebox. The flask was then removed from the glovebox and cooled to −78 °C. A solution of phenyldichloroborane (1.05 g, 6.6 mmol, 1.1 equiv.) in toluene (10 mL) was added dropwise, and the reaction mixture was stirred at −78 °C for 1 h. The mixture was then slowly warmed to room temperature and stirred for an additional 1 h. The flask was returned to the glovebox, the volatiles were removed under reduced pressure, and the resulting solid was washed with dry pentane (3–5 mL). Drying under vacuum afforded borepin **3** as a white solid (870 mg, 4.0 mmol, 73% yield). Crystals suitable for single-crystal X-ray diffraction were obtained by slow evaporation of a saturated toluene solution of **3** at room temperature.

^{1}H NMR (400 MHz, Benzene-d_6) δ (ppm): 8.21 – 8.19 (m, 2H, Ph), 8.05 (d, *J* = 13.7 Hz, 2H, 4-C*H*), 7.83 (d, *J* = 13.8 Hz, 2H, 5-C*H*), 7.48 – 7.44 (m, 2H, 2-C*H*), 7.41 – 7.31 (m, 3H, Ph), 7.18 – 7.14 (m, 2H, 1-C*H*).

^{13}C{^{1}H} NMR (101 MHz, Benzene-d_6) δ (ppm): 153.9 (4-*C*), 140.4 (5-*C*), 138.4 (3-*C*), 135.1 (2-*C*), 134.6 (Ph), 130.5 (Ph), 128.4 (1-*C*), 128.1 (Ph).

^{11}B NMR (128 MHz, Benzene-d_6) δ (ppm): 49.81 (s).

HRMS (ASAP): m/z calcd for $C_{16}H_{14}B$ [M+H]$^{+}$: 217.1189; found: 217.1168.

Crystal data: $C_{16}H_{13}B$, colorless plate, 0.13 x 0.06 x 0.02 mm^3, Monoclinic *P2₁/C*, a=5.8144(2)Å, b=7.8342(2)Å, c=25.2717(7)Å, α=90°, β=96.037(3)°, γ=90°, from 8188 reflections, 2Θ range for data collection 7.034° to 150.348°, T=100(2)K, V=1144.77(6)Å^3, Z=2, Fw=216.07, Dc=1.254 Mg·m^{-3}, μ=0.520 mm^{-1}.

Data collection and processing: Rigaku Synergy R diffractometer equipped with Hypix-Arc 150 detector, CuKα (λ=1.54184Å), -6≤h≤7, -9≤k≤9, -31≤l≤31, frame scan width = 0.25°, scan speed 1.0° per 1.6 sec for low resolution and 6.0 sec for high resolution, 8188 reflections collected. The data were processed with CrysAlis^{PRO}.

Solution and refinement: Structure solved with SHELXT program. Full matrix least-squares refinement based on F^2 with SHELXL on 202 parameters with no restraints gave final R_1= 0.0666 (based on F^2) for data with I>2σ(I), wR_2= 0.1897 on 8188 reflections, and final R_1= 0.0753 (based on F^2), wR_2= 0.2004 for all data; goodness-of-fit on F^2 = 1.122; largest electron density peak 0.23 e·Å^{-3}. Largest hole -0.20 e·Å^{-3}.

* Twin structure

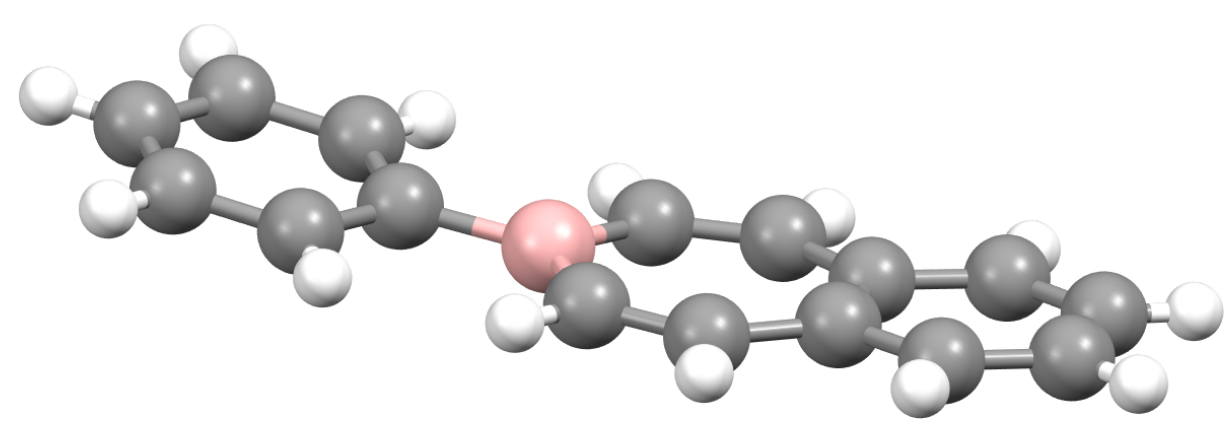

Compound 4

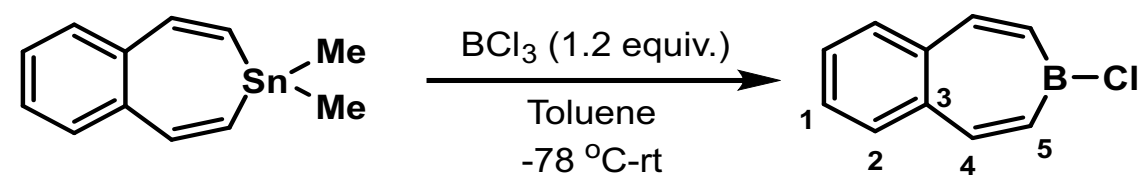

In a nitrogen-filled glovebox, an oven-dried 100 mL Schlenk flask equipped with a stir bar was charged with dimethylstannepin **2** (2.0 g, 7.2 mmol, 1.0 equiv.) and dry toluene (20 mL). The flask was removed from the glovebox, cooled to −78 °C, and a 1.0 M solution of BCl_3 in hexane (8.7 mL, 8.7 mmol, 1.2 equiv.) was added dropwise. The reaction mixture was stirred at −78 °C for 1 hour, then warmed to room temperature and stirred for an additional 1 hour. The flask was then transferred back into the glovebox, the volatiles were removed under reduced pressure, and the resulting solid was washed with cold pentane (3 × 7 mL). Drying the filtrate under reduced pressure afforded borepin **4** as a white, fluffy solid (1060 mg, 6.12 mmol, 85% yield).

Single crystals of the DMAP adduct suitable for X-ray diffraction analysis were obtained by co-crystallization of borepin **4** with 1.0 equiv. of DMAP through slow evaporation of a benzene solution.

^{1}H NMR (500 MHz, Benzene-d_6) δ (ppm): 7.67 (d, J = 13.7 Hz, 2H, 4-CH), 7.28 – 7.22 (m, 4H, 5-CH and 2-CH), 7.10 – 7.07 (m, 2H, 1-CH).

^{13}C{^{1}H} NMR (126 MHz, Benzene-d_6) δ (ppm): 154.4 (4-C), 139.1 (5-C), 137.5 (3-C), 135.4 (2-C), 129.0 (1-C).

^{11}B NMR (160 MHz, Benzene-d_6) δ (ppm): 50.37 (s).

Crystal data: $C_{24}H_{28}BN_4$ + $C_{17}H_{18}BClN_2$+$CHCl_3$+Cl, colorless prism, 0.524 x 0.383 x 0.043 mm^3, Monoclinic *P2₁/c*, a=10.2985(3)Å, b=29.1124(8)Å, c=14.6202(3)Å, α=90°, β=106.941(3)°, γ=90°, from 47141 reflections, 2Θ range for data collection 4.992° to 53.464°, T=100(2)K, V=4193.1(2)$Å^3$, Z=4, Fw=834.72, Dc=1.322 $Mg{\cdot}m^{-3}$, μ=0.385 mm^{-1}.

Data collection and processing: Rigaku Synergy R diffractometer equipped with Hypix-Arc 100 detector, MoKα (λ=0.71073Å), -13≤h≤13, -36≤k≤33, -18≤l≤17, frame scan width = 0.5°, scan speed 1.0° per 7.00 sec, 47141 reflections collected, 8893 independent reflections (R-int =0.0314). The data were processed with $CrysAlis^{PRO}$.

Solution and refinement: Structure solved with SHELXT program. Full matrix least-squares refinement based on F^2 with SHELXL on 502 parameters with no restraints gave final R_1= 0.0387 (based on F^2) for data with I > 2σ(I), wR_2= 0.0871 on 8893 reflections, and final R_1= 0.0522 (based on F^2), wR_2= 0.0920 for all data, goodness-of-fit on F^2 = 1.040, largest electron density peak 0.30 $e{\cdot}Å^{-3}$. Largest hole -0.29 $e{\cdot}Å^{-3}$.

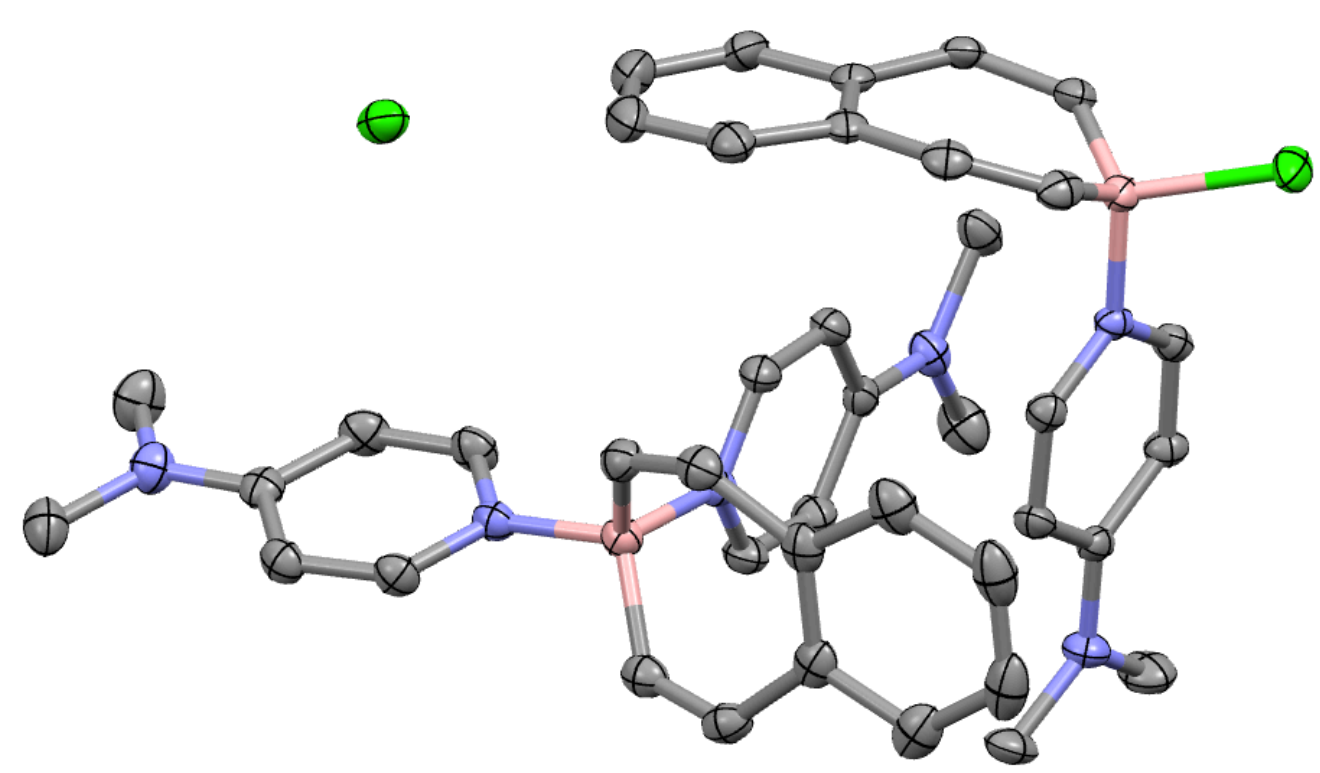

^{Cyh}CAAC

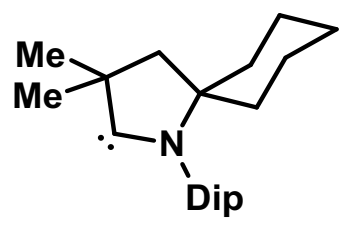

Compound **^{Cyh}CAAC** was synthesized according to the literature procedure from ^{Cyh}CAAC•HBF_4.[2]

^{1}H NMR (300 MHz, Tol-*d*$_8$) δ (ppm): 7.24 – 7.12 (m, 3H), 3.14 (sept, *J* = 6.8 Hz, 2H), 2.27 – 2.13 (m, 2H), 2.06 – 1.97 (m, 2H), 1.66 – 1.34 (m, 8H), 1.27 (d, *J* = 6.9 Hz, 6H), 1.18 (d, *J* = 6.7 Hz, 6H), 1.13 (s, 6H). Data are consistent with the literature values.[3]

^{Me}CAAC

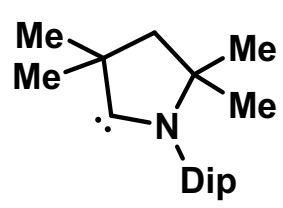

Compound **^{Me}CAAC** was synthesized according to the literature procedure from ^{Me}CAAC•HBF_4.[4]

^{1}H NMR (300 MHz, Benzene-*d*$_6$) δ (ppm): 7.20 (dd, *J* = 8.8, 6.3 Hz, 1H), 7.14 – 7.07 (m, 2H), 3.08 (sept, *J* = 6.8 Hz, 2H), 1.49 (s, 2H), 1.41 (s, 6H), 1.21 (dd, *J* = 6.8, 1.1 Hz, 12H), 1.04 (s, 6H). Data are consistent with the literature values.[4]

Compound 5b

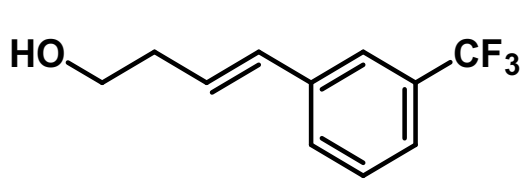

Compound **5b** was prepared following the reported literature procedure.[5]

^{1}H NMR (300 MHz, Chloroform-*d*) δ (ppm): 7.61 (s, 1H), 7.53 – 7.38 (m, 3H), 6.53 (d, *J* = 15.9 Hz, 1H), 6.31 (dt, *J* = 15.9, 7.0 Hz, 1H), 3.78 (t, *J* = 6.3 Hz, 2H), 2.51 (m, 2H), 2.05 (brs, 1H). Data are consistent with the literature values.[5]

Compound 5d

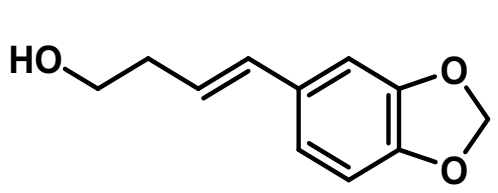

Compound **5d** was prepared following the reported literature procedure.[6]

^{1}H NMR (300 MHz, Chloroform-*d*) δ (ppm): 6.91 (s, 1H), 6.80 – 6.73 (m, 2H), 6.42 (d, *J* = 15.7 Hz, 1H), 6.00 (dt, *J* = 15.7, 7.0 Hz, 1H), 5.95 (s, 2H), 3.74 (t, *J* = 6.0 Hz, 2H), 2.49 – 2.43 (m, 2H), 1.58 (brs, 1H). Data are consistent with the literature values.[7]

Compound 5h

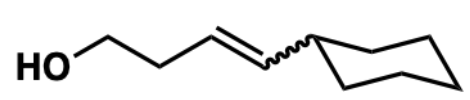

Compound **5h** was prepared according to the reported literature procedure.[6]
The compound was isolated as a mixture of *cis* and *trans* isomers (2:1), as described in the literature precedent. The data are consistent with the reported literature values.[6,8]

^{1}H NMR (300 MHz, Chloroform-*d*) δ (ppm): 5.54 – 5.20 (m, 2H), 3.64 – 3.54 (m, 2H), 2.37 – 2.20 (m, 2H), 1.89 (brs, 1H), 1.74 – 1.57 (m, 6H), 1.31 – 1.25 (m, 1H), 0.99 – 1.23 (m, 4H).

General procedure for Sonogashira coupling and cis-reduction

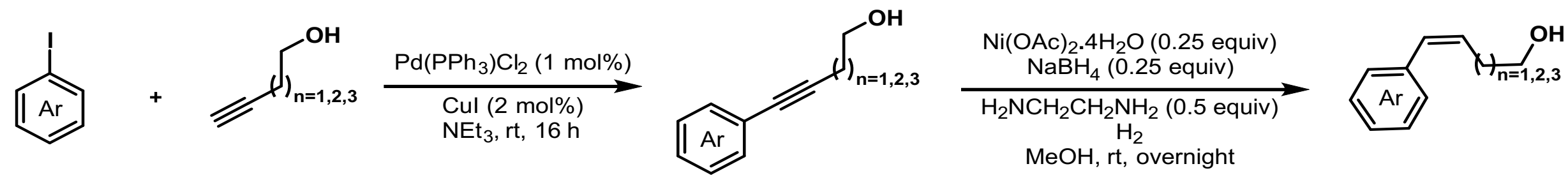

Step 1: An oven-dried 50 mL round-bottom flask was charged with a stir bar, $Pd(PPh_3)_2Cl_2$ (1.0 mol%), CuI (2.0 mol%), and the appropriate aryl iodide (1.0 equiv.). Triethylamine was then added, and the resulting solution was degassed at room temperature for 5 min. The terminal alkyne (1.0 equiv.) was subsequently added, and the reaction mixture was stirred at room temperature for 16 hours. Upon completion, the reaction was quenched with water, and the mixture was extracted with DCM. The combined organic layers were washed with water, dried over $MgSO_4$, filtered, and concentrated in vacuo. The crude product was purified by column chromatography on silica gel to afford the desired product (72-96% yield).

Step 2: A dry 25 mL round-bottom flask was charged with a stir bar and $Ni(OAc)_2 \cdot 4H_2O$ (0.25 equiv.) and purged with nitrogen. Methanol (10 mL) was then added, and the reaction mixture was cooled to 0 °C. $NaBH_4$ (0.25 equiv.) was added in one portion, resulting in an immediate color change to black. The mixture was then allowed to warm to room temperature and stirred for 5 min. Ethylenediamine (0.50 equiv.) was added, and the resulting mixture was stirred for an additional 5 min. A solution of the alkyne (1.0 equiv.) prepared in the previous step in methanol (5 mL) was then added in one portion. The reaction mixture was purged with H_2 (balloon) and stirred under a hydrogen atmosphere overnight. Upon completion, the reaction mixture was filtered through Celite and washed with dichloromethane. The filtrate was concentrated in vacuo, and the crude product was purified by column chromatography on silica gel to afford the desired product (76-96% yield).

Compound 5a

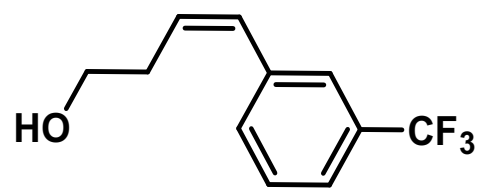

Following the General procedure for Sonogashira coupling, 3-iodobenzotrifluoride (1 g, 3.7 mmol) was coupled with 1-butynol (259 mg, 3.7 mmol), yielding the desired product in 95% yield (750 mg). The subsequent step was conducted on a 750 mg (3.5 mmol) scale to afford the corresponding *cis*-alkene (643 mg, 85% yield).

^{1}H NMR (500 MHz, Chloroform-*d*) δ (ppm): 7.56 (s, 1H), 7.51 – 7.45 (m, 3H), 6.61 (d, J = 11.7 Hz, 1H), 5.82 (dt, J = 11.7, 7.4 Hz, 1H), 3.77 (t, J = 6.3 Hz, 2H), 2.62 – 2.58 (m, 2H), 1.64 (brs, 1H).

^{13}C{^{1}H} NMR (126 MHz, Chloroform-*d*) δ (ppm): 137.9, 131.8 (*q*, $J_{C\text{-}F}$ = 1.1 Hz),130.6 (q, $J_{C\text{-}F}$ = 32.1 Hz), 130.2, 130.1, 128.6, 125.4 (q, $J_{C\text{-}F}$ = 3.8 Hz), 124.1 (q, $J_{C\text{-}F}$ = 272.8 Hz), 123.4 (q, $J_{C\text{-}F}$ = 3.8 Hz), 62.2, 31.8.

^{19}F {^{1}H} NMR (471 MHz, Chloroform-*d*) δ (ppm): -63.68.

Physical state: colorless oil

TLC: R_f = 0.4 (Hexane:EtOAc 4:1)

HRMS (ESI): m/z calcd for $C_{11}H_{10}F_3$ $[M\text{-}OH]^+$: 199.0735; found: 199.0732.

Compound 5c

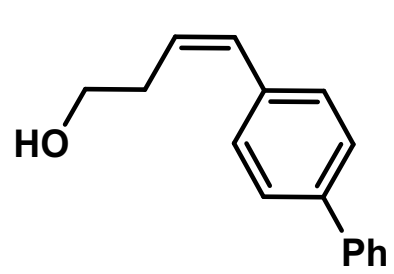

4-Iodobiphenyl (500 mg, 1.78 mmol) was coupled with 1-butynol (125 mg, 1.78 mmol), affording the desired product in 78% yield (320 mg). The subsequent step was conducted on a 222 mg (1 mmol) scale to afford the corresponding cis-alkene (170 mg, 76% yield).

^{1}H NMR (500 MHz, Chloroform-*d*) δ (ppm): 7.64 – 7.59 (m, 3H), 7.47 (t, J = 7.6 Hz, 2H), 7.42 (d, J = 8.1 Hz, 2H), 7.37 (t, J = 7.3 Hz, 2H), 6.64 (d, J = 11.7 Hz, 1H), 5.75 (dt, J = 11.7, 7.4 Hz,

1H), 3.81 (t, J = 6.5 Hz, 2H), 2.73 – 2.68 (m, 2H), 1.48 (brs, 1H). Data are consistent with the literature values.[9]

Compound 5e

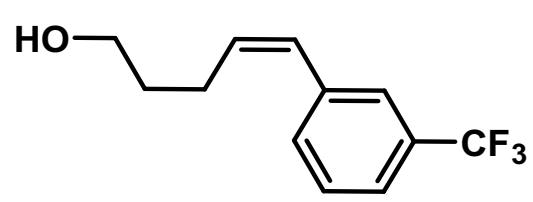

3-Iodobenzotrifluoride was performed on a 2.1 g scale (7.7 mmol) with 1-pentynol (631 mg, 7.7 mmol), to obtain the desired product in 80% yield (1410 mg). The subsequent step was conducted on a 255 mg (1.1 mmol) scale to afford the corresponding cis-alkene (230 mg, 90% yield).

^{1}H-NMR (300 MHz, Benzene-d_6) δ (ppm): 7.53 (s, 1H), 7.21 (d, J = 7.7 Hz, 1H), 7.15 – 7.13 (m, 1H), 6.91 (t, J = 7.8 Hz, 1H), 6.16 (d, J = 11.6 Hz, 1H), 5.47 (dt, J = 11.7, 7.4 Hz, 1H), 3.19 (t, J = 6.1 Hz, 2H), 2.13 (ddd, J = 15.0, 7.5, 1.8 Hz, 2H), 1.38 – 1.25 (m, 2H), 0.45 (brs, 1H).). Data are consistent with the literature values.[10]

Compound 5f

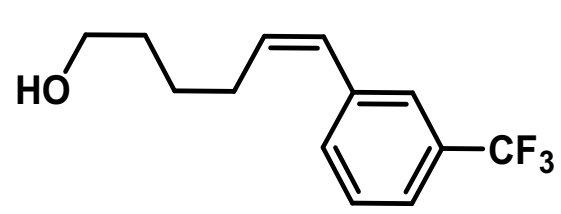

3-Iodobenzotrifluoride was performed on a 2.1 g scale (7.7 mmol) with 1-hexynol (724 mg, 7.7 mmol), to obtain the desired product in 72% yield (1350 mg). The subsequent step was conducted on a 280 mg (1.15 mmol) scale to afford the corresponding cis-alkene (230 mg, 82% yield).

^{1}H NMR (500 MHz, Chloroform-d) δ (ppm): 7.52 (s, 2H), 7.50 – 7.45 (m, 3H), 6.46 (d, J = 11.6 Hz, 1H), 5.78 (dt, J = 11.7, 7.3 Hz, 1H), 3.66 (t, J = 6.3 Hz, 2H), 2.38 – 2.34 (m, 2H), 1.65 – 1.53 (m, 4H), 1.33 (brs, 1H).

^{13}C{^{1}H} NMR (126 MHz, Chloroform-d) δ (ppm): 138.3, 134.3, 131.8 (q, J_{C-F} = 1.1 Hz), 131.01, 130.5 (q, J_{C-F} = 32.1 Hz) 130.2, 130.1 (d, J_{C-F} = 8.6 Hz), 128.5, 127.8, 125.3 (d, J_{C-F} = 3.8 Hz), 124.1 (q, J_{C-F} = 272.9 Hz), 123.1 (d, J_{C-F} = 3.8 Hz), 62.6, 32.2, 28.1, 25.9.

^{19}F {^{1}H} NMR (471 MHz, Chloroform-d) δ (ppm): -63.68.

TLC: R_f = 0.3 (Hexane:EtOAc 5:1)

GCMS (EI): m/z calcd for $C_{13}H_{15}F_3O$: 244.1; found: 244.1.

Compound 5g

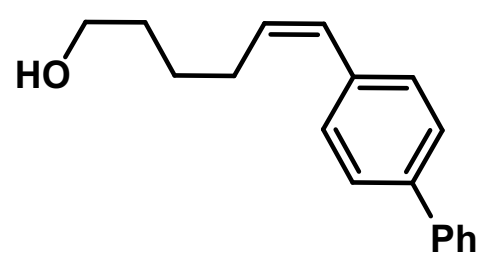

4-Iodobiphenyl was performed on a 1100 mg (3.94 mmol) scale with 1-hexynol (370 mg, 3.94 mmol), affording the desired product in 96% yield (950 mg). The subsequent step was conducted on a 950 mg (1 mmol) scale to afford the corresponding cis-alkene (920 mg, 96% yield).

^{1}H NMR (300 MHz, Chloroform-*d*) δ (ppm): 7.65 – 7.58 (m, 4H), 7.47 (t, J = 7.4 Hz, 2H), 7.38 (d, J = 8.4 Hz, 3H), 6.49 (d, J = 11.7 Hz, 1H), 5.72 (dt, J = 11.7, 7.2 Hz, 1H), 3.68 (t, J = 6.2 Hz, 2H), 2.49 – 2.42 (m, 2H), 1.71 – 1.55 (m, 4H), 1.43 (s, 1H).
^{13}C{^{1}H} NMR (75 MHz, Chloroform-*d*) δ (ppm): 140.8, 139.3, 136.7, 132.9, 129.2, 128.79, 128.73, 127.2, 126.9, 126.8, 62.8, 32.3, 28.4, 26.1.
Physical State: white solid
TLC: R_f = 0.3 (Hexane:EtOAc 4:1)
HRMS (ESI): m/z calcd for $C_{18}H_{19}$ $[M-OH]^+$: 235.1481; found: 235.1487.

Compound 13

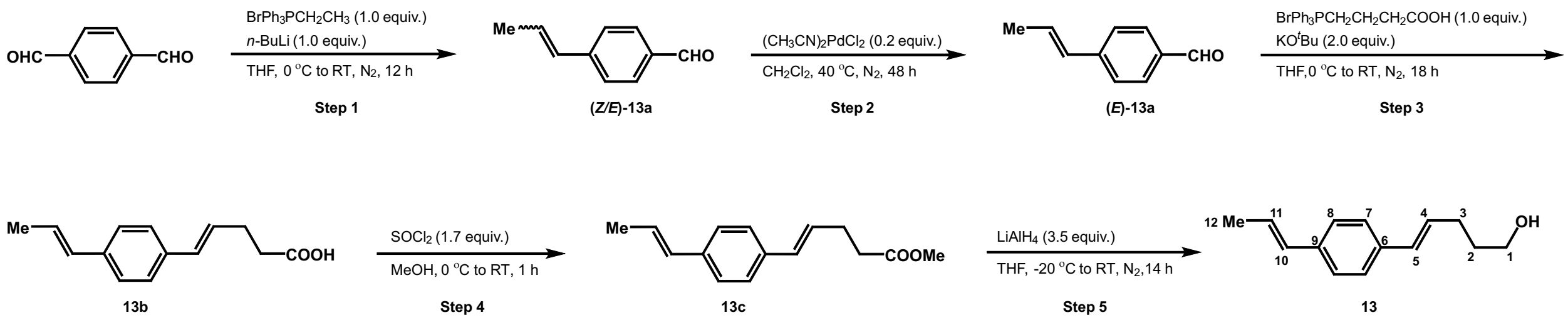

Step 1:[11] To a stirring suspension of ethyltriphenylphosphonium bromide (3.46 g, 9.3 mmol, 1.0 equiv.) in anhydrous THF (40 mL) cooled at 0 °C was added *n*-butyllithium (5.8 mL of 1.6 M solution in *n*-hexane, 1.0 equiv.) under nitrogen atmosphere. The resulting reaction mixture was stirred at 0 °C for 30 minutes, followed by the addition of a solution of terephthalaldehyde (1.25 g, 9.3 mmol, 1.0 equiv.) in anhydrous THF (15 mL). The reaction mixture was then allowed to warm to room temperature and stirred for 12 h. Upon completion, the reaction was quenched with saturated aqueous NH_4Cl solution, and the aqueous layer was extracted with ethyl acetate (3 x). The combined organic extracts were washed with brine, dried over Na_2SO_4 and concentrated under reduced pressure. Finally, the crude residue was purified by column chromatography (silica gel; hexane:ethyl acetate, 98:2) to afford a *E*:*Z* (40:60) mixture of styrene derivative **(*Z*/*E*)-13a** as a colourless liquid. **Isolated Yield:** 0.84 g (62%). This mixture was carried to the next step for the synthesis of **(*E*)-13a** through Pd-catalyzed alkene isomerization.

Step 2:[12,13] Inside a N_2-filled glovebox, a 150 mL pressure tube was charged with monoalkene mixture **(*Z*/*E*)-13a** (0.73 g, 5.0 mmol, 1.0 equiv.), bis(acetonitrile)dichloropalladium(II) (0.26 g, 1.0 mmol, 0.2 equiv.), and dichloromethane (40 mL). The flask was sealed, taken out of the glovebox, and was heated on a preheated oil bath at 40 °C for 48 h. Upon completion, the reaction mixture was allowed to cool to room temperature and concentrated under reduced pressure. The resulting residue was dissolved in pentane and filtered through a short pad of celite, which was subsequently washed with copious amount of pentane. Concentration of the combined filtrates under reduced pressure afforded (*E*)-selective styrene derivative **(*E*)-13a** (*E*:*Z* = >98:2) as colourless liquid. **Isolated Yield:** 0.59 g (81%). **^{1}H NMR** (400 MHz, Chloroform-*d*) δ (ppm): 9.95 (s, 1H), 7.83 – 7.76 (m, 2H), 7.46 (d, *J* = 8.2 Hz, 2H), 6.50 – 6.37 (m, 2H), 1.93 (d, *J* = 4.8 Hz, 3H). **^{13}C{^{1}H} NMR** (101 MHz, Chloroform-*d*) δ (ppm): 191.9, 144.2, 135.0, 130.4, 130.3, 130.1,

126.4, 18.9. Herein, the described NMR data is of (*E*)-isomer only and is consistent with the literature.[14]

Step 3:[15] To a stirring suspension of (3-carboxypropyl)triphenylphosphonium bromide (2.02 g, 4.7 mmol, 1.0 equiv.) in anhydrous THF (80 mL) cooled at 0 °C was added potassium *tert*-butoxide (9.4 mL of 1.0 (M) solution in THF, 2.0 equiv.) under nitrogen atmosphere. The resulting reaction mixture was stirred at 0 °C for 10 minutes, followed by the addition of a solution of aldehyde **(*E*)-13a** (0.69 g, 4.7 mmol, 1.0 equiv.) in anhydrous THF (10 mL). The reaction mixture was then allowed to warm to room temperature and stirred for 18 h. Upon completion, the reaction was quenched with 80 mL of water at 0 °C. Hereafter, the reaction mixture was acidified to pH 2 using 2(M) HCl followed by extraction with ethyl acetate (3 x 100 mL). The combined organic phases were washed with brine, dried over Na_2SO_4 and concentrated under reduced pressure. Finally, purification of the crude residue by column chromatography (silica gel; hexane:ethyl acetate:acetic acid, 74:25:1) afforded **13b** as an off-white solid. **Isolated Yield:** 0.79 mg (78%). **^{1}H NMR** (400 MHz, Chloroform-*d*) δ (ppm): 7.26 (br s, 4H), 6.46 – 6.32 (m, 2H), 6.29 – 6.12 (m, 2H), 2.55 (br s, 2H), 2.54 (br s, 2H), 1.88 (dd, *J* = 6.5, 1.4 Hz, 3H). **^{13}C{^{1}H} NMR** (101 MHz, Chloroform-*d*) δ (ppm): 178.9, 137.1, 135.9, 131.1, 130.8, 127.6, 126.4, 126.1, 125.7, 33.9, 28.1, 18.7. **HRMS (ASAP)**: m/z calcd for $C_{14}H_{15}O_2$ [M-H]: 215.1072; found: 215.1079.

Step 4:[16] To a stirring suspension of carboxylic acid **13b** (270 mg, 1.25 mmol, 1.0 equiv.) in methanol (6 mL) was dropwise added dichloromethane solution of thionyl chloride (2.1 mL 1.0 M solution, 1.7 equiv.) at 0 °C. The reaction mixture was then allowed to warm to room temperature and stirred for 1 h. Upon completion, the reaction mixture was quenched with 20 mL of ice water and was extracted with dichloromethane (3 x 20 mL). The combined organic extracts were dried over Na_2SO_4 and concentrated under reduced pressure. Finally, the crude residue was purified by column chromatography (silica gel; hexane:ethyl acetate, 95:5) to afford **13c** as a white solid. **Isolated Yield:** 280 mg (97%). **^{1}H NMR** (500 MHz, Chloroform-*d*) δ (ppm): 7.26 (br s, 4H), 6.39 (t, *J* = 15.3 Hz, 2H), 6.27 – 6.13 (m, 2H), 3.69 (s, 3H), 2.59 – 2.45 (m, 4H), 1.88 (d, *J* = 6.5 Hz, 3H). **^{13}C{^{1}H} NMR** (126 MHz, Chloroform-*d*) δ (ppm): 173.6, 137.0, 136.0, 130.9, 130.9, 128.0, 126.3, 126.1, 125.6, 51.7, 34.0, 28.4, 18.7. **HRMS (ESI)**: m/z calcd for $C_{15}H_{19}O_2$ [M+H]: 231.1385; found: 231.1382.

Step 5:[17] To a stirring suspension of $LiAlH_4$ (155 mg, 4.1 mmol, 3.5 equiv.) in anhydrous THF (10 mL) was dropwise added a THF (6 mL) solution of ester **13c** (270 mg, 1.17 mmol, 1.0 equiv.) at -

20 °C under N_2 atmosphere. The reaction mixture was then allowed to warm slowly to room temperature and stirred for 14 h. Upon completion, the reaction mixture was carefully quenched with methanol at 0 °C. Hereafter, a solution of potassium sodium tartrate (20 mL) was added, and the mixture was stirred vigorously for 2 h until the solution was clear. Afterwards, the reaction mixture was extracted with ethyl acetate (3 x 30 mL). The combined organic phases were washed with brine, dried over Na_2SO_4 and concentrated under reduced pressure. Finally, the crude residue was purified by column chromatography (silica gel; hexane:ethyl acetate, 90:10) to afford the alcohol **13** as a white solid. **Isolated Yield:** 205 mg (87%).

^{1}H NMR (400 MHz, Chloroform-*d*) δ (ppm): 7.30 – 7.26 (m, 4H, 7/7', 8/8'-C*H*), 6.43 – 6.37 (m, 2H, 5, 10-C*H*), 6.30 – 6.16 (m, 2H, 4, 11-C*H*), 3.73 (t, *J* = 6.4 Hz, 2H, 1-CH_2), 2.35 – 2.30 (m, 2H, 3-CH_2), 1.90 (dd, *J* = 6.5, 1.6 Hz, 3H, 12-CH_3), 1.81 – 1.74 (m, 2H, 2-CH_2), 1.38 (brs, 1H, O*H*).

^{13}C NMR (101 MHz, Chloroform-*d*) δ (ppm): 136.7 (9-*C*), 136.1 (6-*C*), 130.7 (10-*C*), 130.1 (5-*C*), 129.5 (4-*C*), 126.1 (7/7' or 8/8'-C), 125.9, (7/7' or 8/8'-C), 125.3 (11-*C*), 62.4 (1-*C*), 32.3 (2-*C*), 29.3 (3-*C*), 18.5 (12-*C*).

TLC: R_f = 0.4 (Hexane:EtOAc 4:1)

HRMS (ASAP): m/z calcd for $C_{14}H_{17}$ [M-OH]: 185.1330; found: 185.1329.

Compound 16

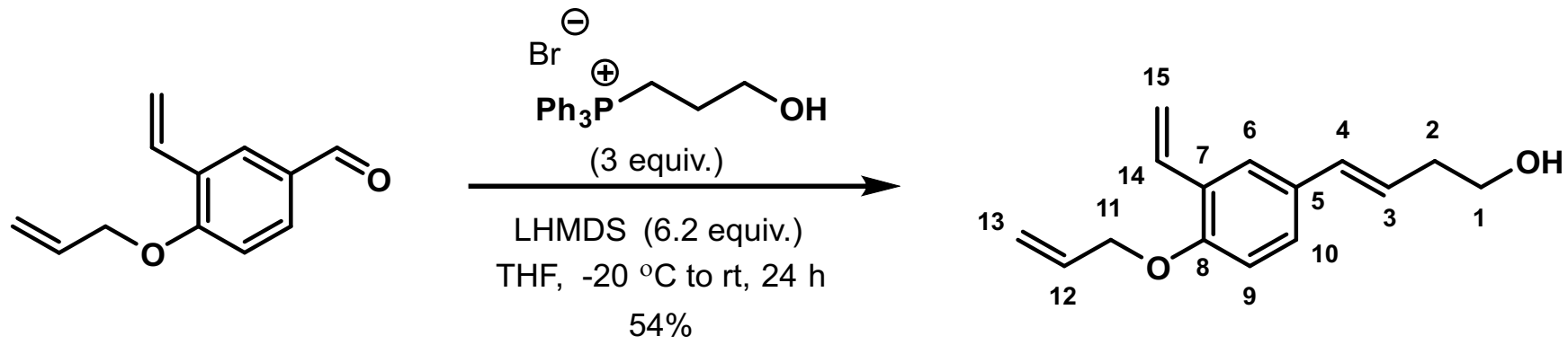

Diene aldehyde was synthesized according to literature procedure.[18]

^{1}H NMR (300 MHz, Chloroform-*d*) δ (ppm): δ 9.92 (s, 1H), 8.03 (d, *J* = 2.0 Hz, 1H), 7.77 (dd, *J* = 8.5, 2.1 Hz, 1H), 7.09 (dd, *J* = 17.7, 11.2 Hz, 1H), 6.99 (d, *J* = 8.5 Hz, 1H), 6.15 – 6.03 (m, 1H), 5.87 (dd, *J* = 17.7, 1.1 Hz, 1H), 5.49 – 5.33 (m, 3H), 4.69 – 4.67 (m, 2H). The data are consistent with the reported literature values.[18]

A solution of lithium bis(trimethylsilyl)amide in tetrahydrofuran (12.4 mL, 1.0 M, 12.4 mmol, 6.2 equiv.) was added dropwise at −20 °C to a suspension of (3-hydroxypropyl)triphenylphosphonium bromide (2.4 g, 6.0 mmol, 3.0 equiv.) in dry THF (20 mL). The mixture was stirred at the same temperature for 45 min, after which a solution of the aldehyde (376 mg, 2.0 mmol, 1.0 equiv.) in THF (5 mL) was added dropwise. The reaction mixture was allowed to warm gradually to room temperature and stirred for 24 h. The reaction was then quenched with water and extracted with ethyl acetate. The combined organic layers were washed with water, dried over Na_2SO_4, filtered, and concentrated under reduced pressure. Purification of the crude residue by silica gel column chromatography afforded the desired product **16** (250 mg, 54% yield) as colorless oil.

^{1}H NMR (300 MHz, Chloroform-*d*) δ (ppm): 7.50 (d, *J* = 2.3 Hz, 1H, 6-C*H*), 7.23 (dd, *J* = 8.4, 2.3 Hz, 1H, 10-C*H*), 7.09 (dd, *J* = 17.8, 11.2 Hz, 1H, 14-C*H*), 6.81 (d, *J* = 8.5 Hz, 1H, 9-C*H*), 6.46 (d, *J* = 15.9 Hz, 1H, 4-C*H*), 6.16 – 6.02 (m, 2H, 3-C*H*, 12-C*H*), 5.79 (dd, *J* = 17.8, 1.6 Hz, 1H, 15-CH_aH$_b$), 5.47 – 5.40 (m, 1H, 13-CH_aH$_b$), 5.32 – 5.28 (m, 2H, 13-CH$_a$$H_b$, 15-CH$_a$$H_b$), 4.58 (dt, *J* = 5.1, 1.6 Hz, 2H, 11-CH_2), 3.76 (t, *J* = 6.3 Hz, 2H, 1-CH_2), 2.52 – 2.45 (m, 2H, 2-CH_2), 1.69 (brs, 1H, O*H*).

^{13}C {^{1}H} NMR (75 MHz, Chloroform-*d*) δ (ppm): 155.2 (8-*C*), 133.2 (12-*C*), 132.2 (4-*C*), 131.6 (14-*C*), 130.1 (5-*C*), 127.0 (7-*C*), 126.5 (10-*C*), 124.4 (3-*C*), 124.2 (6-*C*), 117.4 (13-*C*), 114.6 (15-*C*), 112.4 (9-*C*), 69.2 (11-*C*), 62.1 (1-*C*), 36.4 (2-*C*).

TLC: R_f = 0.3 (Hexane:EtOAc 3:1)

HRMS (ASAP): m/z calcd for $C_{15}H_{19}O_2$ $[M+H]^+$: 231.1385; found: 231.1384.

Compound 18

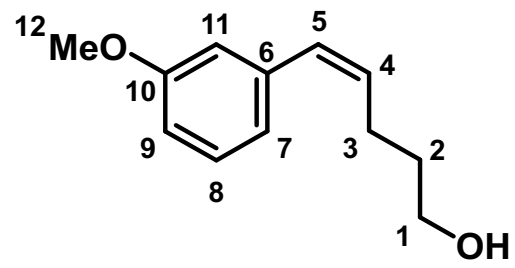

Following the general procedure, a Sonogashira coupling of 3-bromoanisole (1.0 g, 5.3 mmol, 1 equiv.) was carried out with 4-pentynol (0.5 mL, 5.3 mmol, 1 equiv.) at 70 °C, affording the desired coupling product in 89% yield (900 mg). The subsequent step was conducted on a 380 mg (2 mmol) scale to afford the corresponding cis-alkene **18** (330 mg, 86% yield) as pale yellow oil.

^{1}H NMR (500 MHz, Chloroform-*d*) δ (ppm): 7.27 (t, *J* = 7.9 Hz, 1H, 8-C*H*), 6.90 (d, *J* = 7.6 Hz, 1H, 7-C*H*), 6.84 (s, 1H, 11-C*H*), 6.80 (dd, *J* = 8.2, 2.4 Hz, 1H, 9-C*H*), 6.45 (d, *J* = 11.6 Hz, 1H, 5-C*H*), 5.69 (dt, *J* = 11.6, 7.3 Hz, 1H, 4-C*H*), 3.83 (s, 3H, 12-CH_3), 3.69 (t, *J* = 6.5 Hz, 2H, 1-CH_2), 2.45 (qd, *J* = 7.4, 1.8 Hz, 2H, 3-CH_2), 1.77 – 1.72 (m, 2H, 2-CH_2).

^{13}C NMR (126 MHz, Chloroform-*d*) δ (ppm): 159.4 (10-*C*), 138.9 (6-*C*), 132.3 (4-*C*), 129.4 (5-*C*), 129.1 (8-*C*), 121.3 (7-*C*), 114.3 (11-*C*), 112.1 (9-*C*), 62.4 (1-*C*), 55.2 (12-*C*), 32.8 (2-*C*), 24.9 (3-*C*).

TLC: R_f = 0.4 (Hexane:EtOAc 4:1)

HRMS (ESI): m/z calcd for $C_{12}H_{15}O$ $[M-OH]^+$: 175.1123; found: 175.1131.

Compound 20

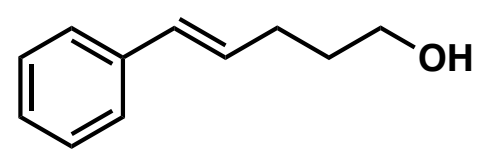

Compound **20** was synthesized according to literature procedure.[19]

^{1}H NMR (300 MHz, Chloroform-*d*) δ (ppm): 7.39 – 7.28 (m, 4H), 7.25 – 7.20 (m, 1H), 6.45 (d, *J* = 15.9 Hz, 1H), 6.26 (dt, *J* = 15.8, 6.8 Hz, 1H), 3.73 (t, *J* = 6.5 Hz, 2H), 2.38 – 2.30 (m, 2H), 1.83 – 1.73 (m, 2H), 1.60 (brs, 1H). The data are consistent with the reported literature values.[19]

Procedure for the borylene insertion into C_{sp3}-H of ^{Cyh}CAAC

Compound 3a

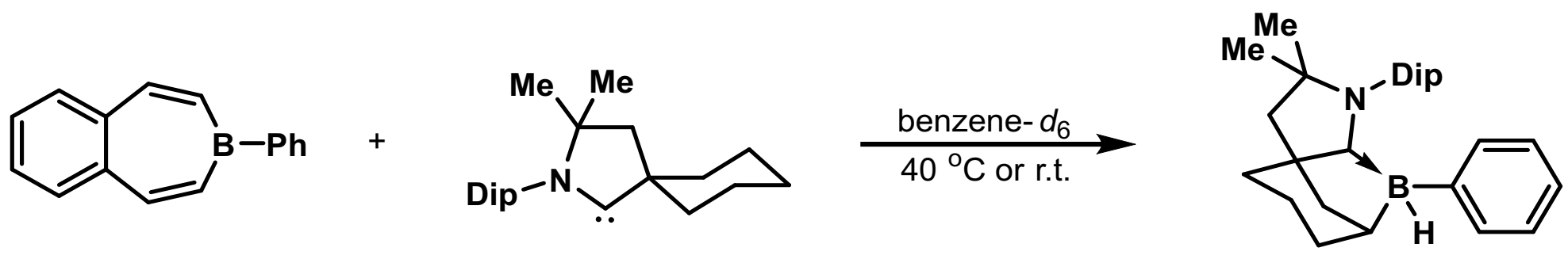

3a, 60%

In a nitrogen-filled glovebox, an oven-dried J. Young NMR tube was charged with phenyl borepin **3** (21.6 mg, 0.10 mmol, 1.0 equiv.) and ^{Cyh}CAAC (32.5 mg, 0.10 mmol, 1.0 equiv.) in benzene-d_6. The NMR tube was sealed, removed from the glovebox, and either heated in a preheated oil bath at 40 °C for 18 h or left at room temperature for 3 days. The solvent was then removed under reduced pressure, and the crude residue was extracted with pentane (3 × 1.5 mL). Removal of the volatile components under reduced pressure afforded product **3a** as a yellow oil in 60% NMR yield (using 1,3,5-trimethoxybenzene as internal standard). Single crystals suitable for X-ray diffraction were obtained by slow evaporation of a saturated toluene solution of **3a** at room temperature.

^{1}H NMR (500 MHz, Benzene-d_6) δ (ppm): 7.24 (t, J = 7.7 Hz, 1H, Ar-C*H*), 7.09 (m, 5H, Ar-C*H*), 6.84 (d, J = 6.8 Hz, 2H, Ar-C*H*), 2.95 (sept, J = 6.5 Hz, 1H, ^{i}Pr-C*H*), 2.79 (sept, J = 6.7 Hz, 1H, *i*Pr-C*H*), 2.55-2.53 (m, 2H), 2.03 - 2.01 (br. m, 1H, Cy-C*H*), 1.86 (m, 3H, Cy-C*H*), 1.78 – 1.47 (m, 6H, Cy-CH_2, 4-CH_2), 1.34 (d, J = 6.4 Hz, 3H, Dip-CH_3), 1.09 (d, J = 6.6 Hz, 3H), 1.05 (d, J = 7.9 Hz, 3H,), 1.04 (s, 3H), 0.90 (s, 3H, Dip-CH_3), 0.82 (d, J = 6.6 Hz, 3H, iPr-CH_3).

^{13}C{^{1}H} NMR (126 MHz, Benzene-d_6) δ (ppm): 146.92, 146.80, 134.57, 133.87, 129.49, 128.22, 126.56, 125.76, 124.69, 123.88, 83.53, 64.74, 47.93, 47.84, 40.48, 30.31, 29.84, 28.51, 28.49, 28.45 , 26.83, 25.06, 23.97, 23.91, 21.99.

^{11}B NMR (96 MHz, Benzene-d_6) δ (ppm): -10.86 (d, J = 79.1 Hz).

HRMS (ASAP): m/z calcd for $C_{29}H_{39}BN$ $[M-H]^+$: 412.3176; found: 412.3181.

Crystal data: $C_{29}H_{40}BN$, light yellow block, 0.23 x 0.17 x 0.16 mm^3, Monoclinic *P2₁*, a=23.3420(4)Å, b=9.9510(1)Å, c=23.9990(5)Å, α=90°, β=119.075(3)°, γ=90°, from 206720 reflections, Θ range for data collection 2.579° to 30.914°, T=100(2)K, V=4871.94(19)$Å^3$, Z=8, Fw=413.43, Dc=1.127 $Mg·m^{-3}$, μ=0.063 mm^{-1}.

Data collection and processing: Rigaku Synergy R diffractometer equipped with Hypix-Arc 100 detector, MoKα (λ = 0.71073Å), -32≤h≤33, -14≤k≤14, -34≤l≤34, frame scan width = 0.25°, scan speed 1.0° per 6.4 sec, 206720 reflections collected, 28675 independent reflections (R-int =0.0522). The data were processed with $CrysAlis^{PRO}$.

Solution and refinement: Structure solved with SHELXT program. Full matrix least-squares refinement based on F^2 with SHELXL on 1276 parameters with 2 restraints gave final R_1= 0.0497 (based on F^2) for data with I>2σ(I), wR_2= 0.1193 on 28675 reflections, and final R_1= 0.0623 (based on F^2), wR_2= 0.1257 for all data, goodness-of-fit on F^2 = 1.028, largest electron density peak 0.33 $e·Å^{-3}$. Largest hole -0.17 $e·Å^{-3}$.

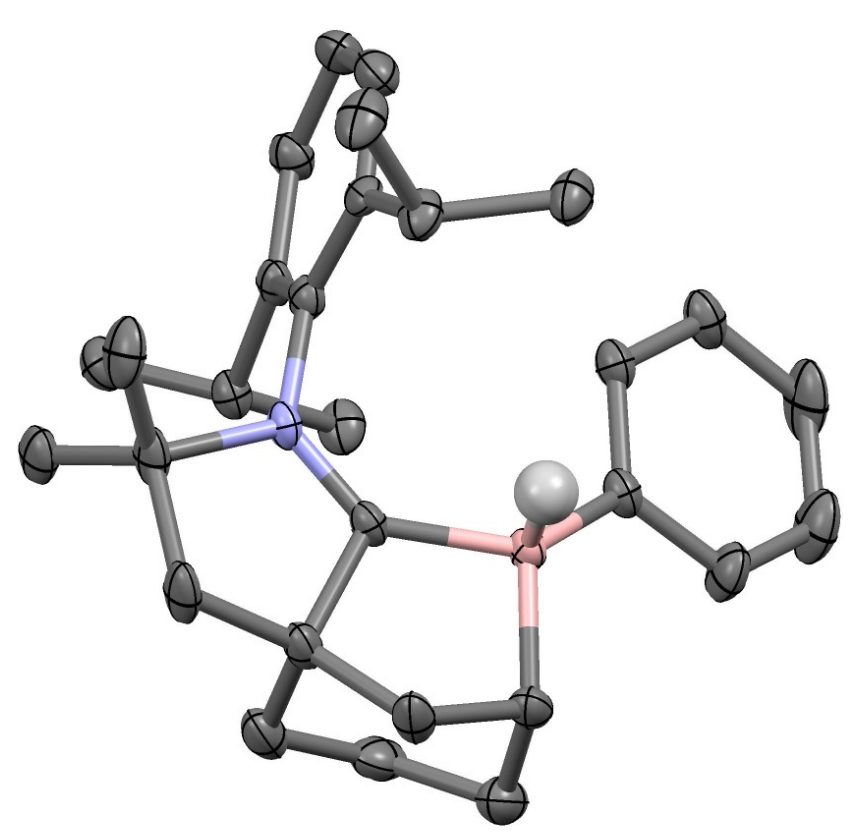

Procedure for the borylene insertion into C_{sp3}-H of ^{Me}CAAC

Compound 3b

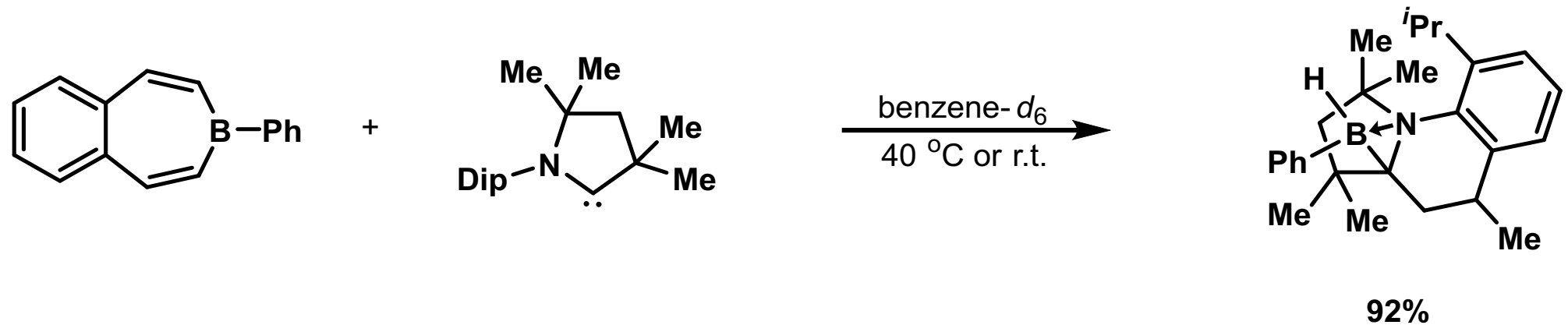

In a nitrogen-filled glovebox, an oven-dried J. Young NMR tube was charged with phenyl borepin **3** (21.6 mg, 0.1 mmol, 1.0 equiv.), ^{Me}CAAC (28.5 mg, 0.1 mmol, 1.0 equiv.), and benzene-d_6 (0.4 mL). The NMR tube was sealed, removed from the glove box, and subsequently heated in a preheated oil bath at 40 °C for 18 h or kept at room temperature for 7 days. Hereafter, the solvent was evaporated under reduced pressure, and the crude residue was extracted with pentane (3 × 1.5 mL). Evaporation of the volatile components under reduced pressure afforded product **3b** as a yellow oil (34.2 mg, 0.092 mmol, 92% yield). Crystals suitable for single-crystal X-ray diffraction were grown by slow evaporation of a saturated solution of **3b** in *n*-pentane. The ^{1}H, ^{13}C, and ^{11}B NMR data are consistent with previously reported data.[20]

^{1}H-NMR (400 MHz, Benzene-d_6) δ (ppm): 7.14 – 7.02 (m, 8H, Ar-C*H*), 3.30 – 3.19 (m, 1H, 10-C*H*), 2.90 (m, 1H, 8-C*H*), 2.33 (d, *J* = 13.3 Hz, 1H, 3-CH_2), 2.00 – 1.80 (m, 2H, 7-CH_2), 1.49 (s, 3H, 1-CH_3), 1.36 (d, *J* = 13.2 Hz, 1H, 3-CH_2), 1.27 (s, 3H, 5-CH_3), 1.21 (d, *J* = 6.6 Hz, 3H, 9-CH_3), 1.19 (s, 3H, 5'-CH_3), 1.12 (d, *J* = 6.6 Hz, 3H, 11-CH_3), 0.80 (s, 3H, 1'-CH_3), 0.27 (d, *J* = 6.5 Hz, 3H, 11'-CH_3).

^{13}C{^{1}H} NMR (101 MHz, Benzene-d_6) δ (ppm): 146.9 (Dip-C_q), 142.1 (Dip-C_q), 136.8 (Dip-C_q), 136.7 (2 x Ph-*C*H), 127.1 (2 x Ph-*C*H), 126.9 (Ph-*C*H), 126.5 (Dip-*C*H), 126.5 (Dip-*C*H), 123.0 (Dip-*C*H), 71.0 (2-C_q), 55.0 (3-CH_2), 40.8 (4-C_q), 32.5 (7-CH_2), 31.1 (8-*C*H), 29.9 (5-CH_3), 28.6 (1-CH_3), 27.8 (10-*C*H), 26.8 (11-CH_3), 26.6 (1'-CH_3), 26.3 (5'-CH_3), 24.6 (11'-CH_3), 19.4 (9-CH_3).

^{11}B NMR (128 MHz, Benzene-d_6) δ (ppm): -7.64.

HRMS (ASAP): m/z calcd for $C_{26}H_{37}BN$ [M+H]$^+$: 374.3019; found: 374.3007.

Crystal data: $C_{26}H_{36}BN$, colorless prism, 0.609 x 0.349 x 0.215 mm^3, Orthorhombic *Pbca*, a=10.42717(9)Å, b=14.2390(1)Å, c=29.6488(3)Å, α=β=γ=90°, from 61039 reflections, 2Θ range for data collection 10.372° to 150.166°, T=100(2)K, V=4402.03(6) Å^3, Z=8, Fw=373.37, Dc=1.127 Mg·m^{-3}, μ=0.468 mm^{-1}.

Data collection and processing: Rigaku Synergy R diffractometer equipped with Hypix-Arc 150 detector, CuKα (λ=1.54184Å), -12≤h≤13 -13≤k≤17, -36≤l≤37, frame scan width = 0.5°, scan speed 1.0° per 0.10 sec, 61039 reflections collected, 4498 independent reflections (R-int =0.0254). The data were processed with CrysAlis^{PRO}.

Solution and refinement: Structure solved with SHELXT program. Full matrix least-squares refinement based on F^2 with SHELXL on 260 parameters with no restraints gave final R_1= 0.0408 (based on F^2) for data with I>2σ(I), wR_2= 0.1057 on 4498 reflections, and final R_1= 0.0428 (based on F^2), wR_2= 0.1076 for all data, goodness-of-fit on F^2 = 1.032; largest electron density peak 0.26 e·Å^{-3}.Largest hole -0.21 e·Å^{-3}.

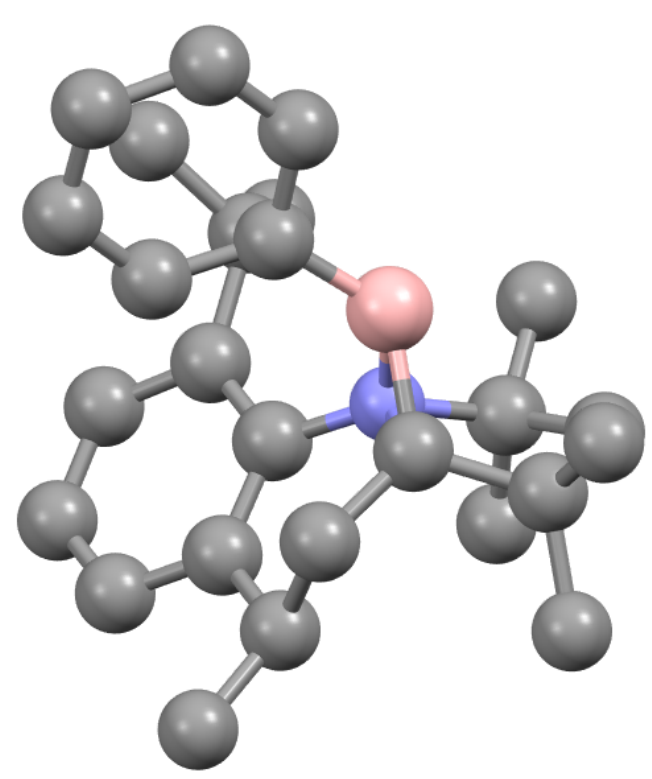

General procedure A: Preparation of alkoxy borepin 6 with tethered alkenes

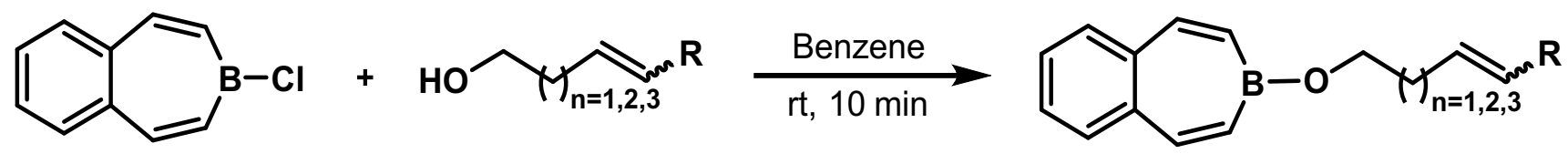

In a nitrogen-filled glovebox, a 5 mL glass vial was charged with borepin **4** (90 mg, 0.51 mmol, 1 equiv.) and dissolved in 1 mL of anhydrous benzene. To this solution, the alcohol **5** (0.51 mmol, 1 equiv.) in benzene (1 mL) was added slowly at room temperature. After 10 minutes, the volatile components were removed under reduced pressure. The residue was washed with cold n-pentane 2 × 3 mL, then dried under reduced pressure, affording the pure borepin-alkenes **6** in 81-97% yield.

General procedure B: Intramolecular [2+1] cycloaddition of borylene with alkene

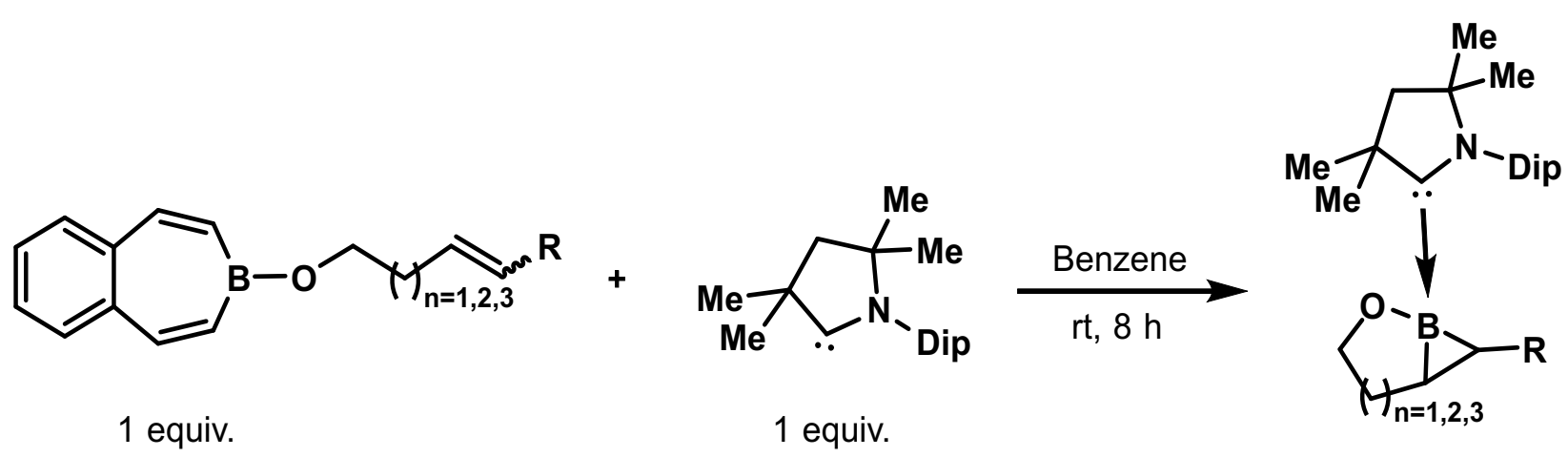

In a nitrogen-filled glovebox, in a 5 mL glass vial, alkoxy borepin **6** (0.1 mmol, 1.0 equiv.) was dissolved in anhydrous benzene (2 mL). A solution of ^{Me}CAAC (28.5 mg, 0.1 mmol, 1.0 equiv.) in benzene (1 mL) was then added slowly to the reaction vessel at room temperature, and the mixture was stirred for 8 hours. After completion, the volatile components were removed under reduced pressure. The resulting residue was dissolved in n-pentane and allowed to evaporate slowly at room temperature or at -30 °C, to afford the product as a yellow solid or yellow crystals. The obtained solid was gently washed with a minimum amount of *n*-pentane (0.5 mL) to obtain the pure borirane product **7**.

Compound 6a

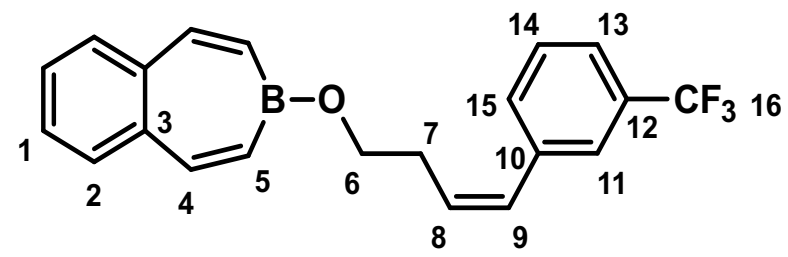

Following **general procedure A** with borepin **4** (90 mg, 0.51 mmol, 1 equiv.) and alcohol **5a** (0.51 mmol, 110 mg, 1 equiv.) afforded 164 mg (0.46 mmol, 91% yield) of compound **6a** as a white solid. Single crystals suitable for X-ray crystallographic analysis were obtained by dissolving the compound in benzene and slowly evaporating the solution at room temperature.

^{1}H NMR (500 MHz, Benzene-d_6) δ (ppm): 7.76 (d, *J* = 14.3 Hz, 2H, 4/4'-C*H*), 7.60 (s, 1H, 11-C*H*), 7.38 – 7.35 (m, 2H, 2/2'-C*H*), 7.24 (d, *J* = 7.8 Hz, 1H, 13-C*H*), 7.20 (d, *J* = 7.8 Hz, 1H, 15-C*H*), 7.14 – 7.10 (m, 2H, 1/1'-C*H*), 6.92 (t, *J* = 7.8 Hz, 1H, 14-C*H*), 6.76 (d, *J* = 14.1 Hz, 2H, 5/5'-C*H*), 6.26 (d, *J* = 11.7 Hz, 1H, 9-C*H*), 5.67 (dt, *J* = 11.7, 7.4 Hz, 1H, 8-C*H*), 3.91 (t, *J* = 6.4 Hz, 2H, 6-CH_2), 2.45 (qd, *J* = 6.6, 1.6 Hz, 2H, 7-CH_2).
^{13}C{^{1}H} NMR (126 MHz, Benzene-d_6) δ (ppm): 152.3 (4/4'-*C*), 138.1 (10-*C*), 137.3 (3/3'-*C*), 134.7 (2/2'-*C*), 133.5 (5/5'-*C*), 131.7 (15-*C*), 130.47 (q, $J_{C\text{-}F}$ = 32.1 Hz, 12-*C*), 130.46 (8-*C*), 129.3 (9-*C*), 128.5 (14-C), 128.1 (1/1'-*C*), 125.4 (q, $J_{C\text{-}F}$ = 3.9 Hz, 11-*C*), 124.6 (q, $J_{C\text{-}F}$ = 272.9 Hz, 16-*C*), 123.2 (q, $J_{C\text{-}F}$ = 3.9 Hz, 13-*C*), 64.3 (6-*C*), 30.7 (7-*C*).
^{19}F {^{1}H} NMR (471 MHz, Benzene-d_6) δ (ppm): -63.27.
^{11}B NMR (160 MHz, Benzene-d_6) δ(ppm): 38.86 (s).
HRMS (ASAP): m/z calcd for $C_{21}H_{19}BF_3O_2$ [M+H]$^+$: 355.1481; found: 355.1481.

Crystal data: $C_{21}H_{18}BF_3O$, colorless plate, 0.230 x 0.117 x 0.035 mm^3, monoclinic *P2₁/c₁*, a=19.7003(5)Å, b=11.2762(2)Å, c=7.8546(2)Å, α=90°, β=97.022(2)°, γ=90°, from 32912 reflections, 2Θ range for data collection 5.516° to 63.14°, T=100(2)K, V=1731.77(7)$Å^3$, Z=4, Fw=354.16, Dc=1.358 $Mg{\cdot}m^{-3}$, μ=0.103 mm^{-1}.

Data collection and processing: Rigaku Synergy R diffractometer equipped with Hypix-Arc 100 detector, MoKα (λ=0.71073Å), -25≤h≤25, -16≤k≤14, -11≤l≤9, frame scan width = 0.25°, scan speed 1.0° per 140.0 sec, 32912 reflections collected, 4979 independent reflections (R-int =0.0390). The data were processed with $CrysAlis^{PRO}$.

Solution and refinement: Structure solved with SHELXT program. Full matrix least-squares refinement based on F^2 with SHELXL on 235 parameters with no restraints gave final R_1= 0.0435 (based on F^2) for data with I>2σ(I), wR_2= 0.1039 on 4979 reflections, and final R_1= 0.0680 (based on F^2), wR_2= 0.1139 for all data, goodness-of-fit on F^2 = 1.024; largest electron density peak 0.35 $e{\cdot}Å^{-3}$.Largest hole -0.23 $e{\cdot}Å^{-3}$.

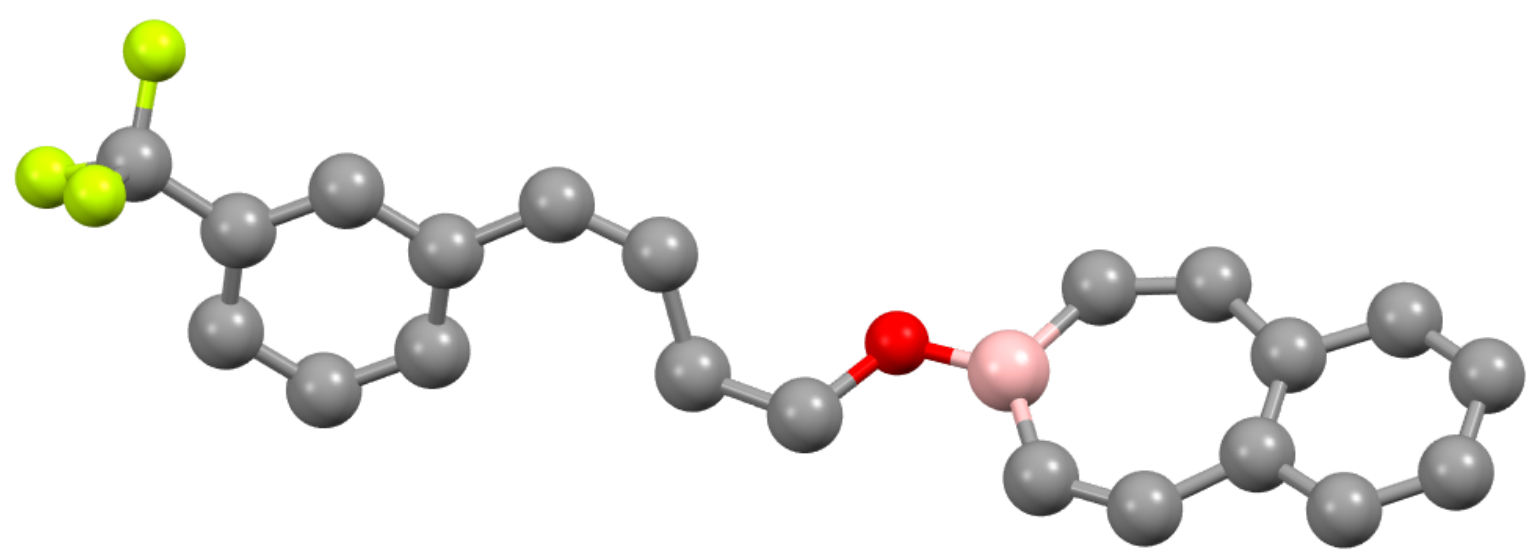

Compound 6b

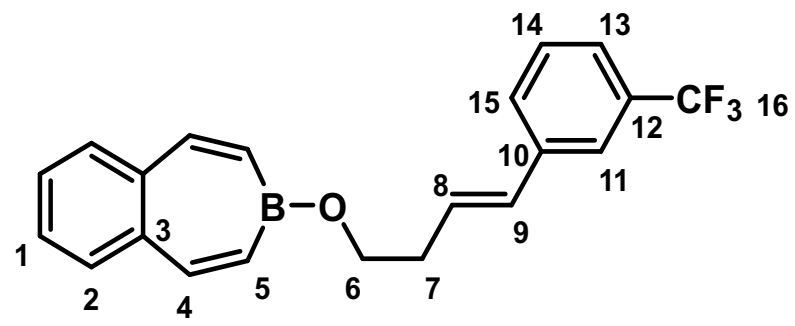

Following **general procedure A** with borepin **4** (90 mg, 0.51 mmol, 1 equiv.) and alcohol **5b** (0.51 mmol, 110 mg, 1 equiv.) afforded 163 mg (0.46 mmol, 90% yield) of compound **6b** as a white solid. Single crystals suitable for X-ray crystallographic analysis were obtained by dissolving the compound in benzene and slowly evaporating the solution at room temperature.

^{1}H NMR (500 MHz, Benzene-d_6) δ (ppm): 7.78 (d, J = 14.2 Hz, 2H, 4/4'-CH), 7.49 (s, 1H, 11-CH), 7.38 – 7.35 (m, 2H, 2/2'-CH), 7.20 (d, J = 7.7 Hz, 1H, 13-CH), 7.14 – 7.10 (m, 2H, 1/1'-CH), 7.07 (d, J = 7.5 Hz, 1H, 15-CH), 6.86 – 6.80 (m, 3H, 5/5',14-CH), 6.13 (d, J = 15.9 Hz, 1H, 9-CH), 6.00 (dt, J = 15.9, 6.9 Hz, 1H, 8-CH), 4.00 (t, J = 6.7 Hz, 2H, 6-CH_2), 2.38 (qd, J = 6.7, 0.8 Hz, 2H, 7-CH_2).

^{13}C{^{1}H} NMR (126 MHz, Benzene-d_6) δ (ppm): 152.3 (4/4'-C), 138.4 (10-C), 137.3 (3/3'-C), 134.7 (2/2'-C), 133.6 (5/5'-C), 130.5 (9-C), 130.5 (q, J_{C-F} = 32.0 Hz, 12-C), 128.9 (15-C), 128.75 (14-C), 128.72 (8-C), 128.1 (1/1'-C), 124.6 (q, J_{C-F} = 272.7 Hz, 16-C), 123.3 (q, J_{C-F} = 3.7 Hz, 13-C), 122.7 (q, J_{C-F} = 3.8 Hz, 11-C), 64.5 (6-C), 35.3 (7-C).

^{19}F {^{1}H} NMR (471 MHz, Benzene-d_6) δ (ppm): -63.34.

^{11}B NMR (160 MHz, Benzene-d_6) δ (ppm): 38.91 (s).

Crystal data: $C_{21}H_{18}BF_3O$, colorless chunk, 0.521 x 0.200 x 0.116 mm^3, monoclinic *P2₁*, a=6.1410(3)Å, b=37.1174(16)Å, c=7.5338(3)Å, α=90°, β=91.296(4)°, γ=90°, from 25957 reflections, 2Θ range for data collection 9.532° to 136.49°, T=100(2)K, V=1716.80(13)$Å^3$, Z=4, Fw=354.16, Dc=1.370 $Mg·m^{-3}$, μ=0.871 mm^{-1}.

Data collection and processing: Rigaku Synergy R diffractometer equipped with Hypix-Arc 150 detector, CuKα (λ=1.54184Å), -7≤h≤7, -44≤k≤44, -9≤l≤9, frame scan width = 0.25°, scan speed 1.0° per 0.12 sec for low resolution and 0.40 for high resolution, 25957 reflections collected, 6268 independent reflections (R-int =0.0597). The data were processed with CrysAlis^{PRO}.

Solution and refinement: Structure solved with SHELXT program. Full matrix least-squares refinement based on F^2 with SHELXL on 469 parameters with 1 restraint gave final R_1= 0.0558 (based on F^2) for data with I>2σ(I), wR_2= 0.1464 on 6268 reflections, and final R_1= 0.0771 (based on F^2), wR_2= 0.1736 for all data; goodness-of-fit on F^2 = 1.087; largest electron density peak 0.24 $e·Å^{-3}$.Largest hole -0.24 $e·Å^{-3}$.

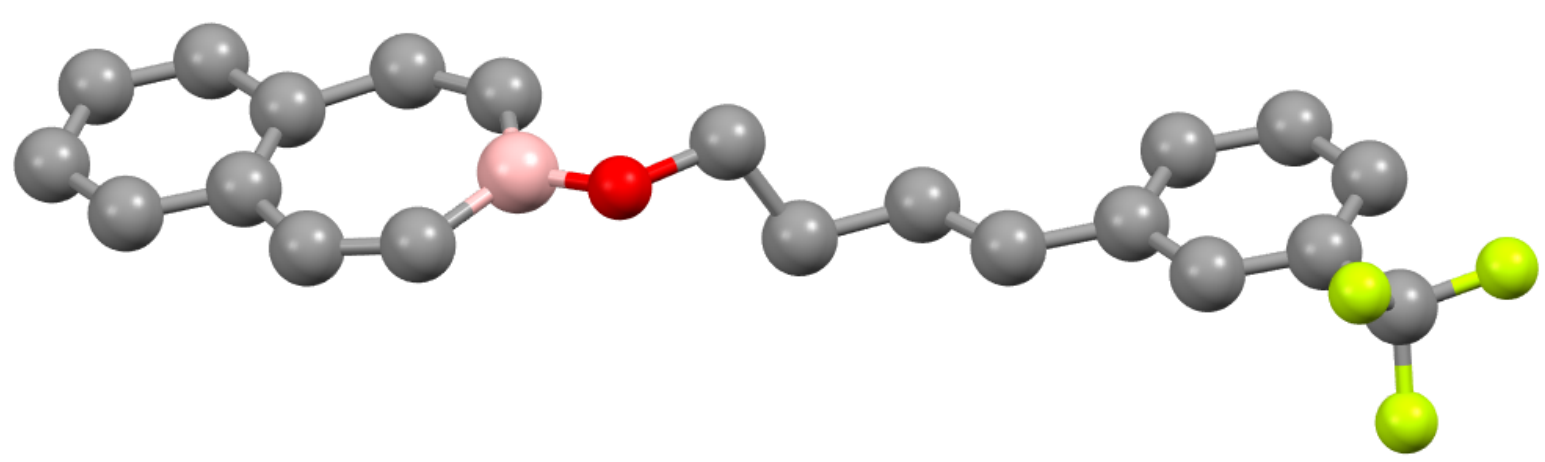

Compound 6c

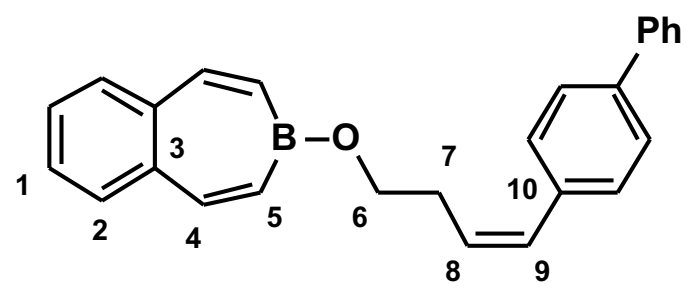

Following **general procedure A** with borepin **4** (90 mg, 0.51 mmol, 1 equiv.) and alcohol **5c** (114 mg, 0.51 mmol, 1 equiv.) afforded 176 mg (0.48 mmol, 95% yield) of compound **6c** as a white solid.

^{1}H NMR (500 MHz, Benzene-d_6) δ (ppm): 7.76 (d, J = 14.3 Hz, 2H, 4/4'-CH), 7.50 – 7.49 (m, 2H, BP-CH), 7.46 (d, J = 8.2 Hz, 2H, BP-CH), 7.37 – 7.33 (m, 4H, BP-CH, 2/2'-CH), 7.23 (t, J = 7.6 Hz, 2H, BP-CH), 7.16 – 7.14 (m, 1H, BP-CH), 7.13 – 7.10 (m, 2H, 1/1'-CH), 6.81 (d, J = 14.1 Hz, 2H, 5/5'-CH), 6.54 (d, J = 11.7 Hz, 1H, 9-CH), 5.76 (dt, J = 11.7, 7.3 Hz, 1H, 8-CH), 4.04 (t, J = 6.7 Hz, 2H, 6-CH_2), 2.72 (qd, J = 6.8, 1.8 Hz, 2H, 7-CH_2).

^{13}C{^{1}H} NMR (126 MHz, Benzene-d_6) δ (ppm): 152.2 (4/4'-C), 140.9 (BP-Cq), 139.6 (BP-Cq), 137.3 (3/3'-C), 136.5 (10-C), 134.7 (2/2'-C), 133.7 (5/5'-C), 130.5 (9-C), 129.3 (BP-C), 128.7 (BP-C), 128.6 (8-C), 128.0 (1/1'-C), 127.1 (BP-C), 127.0 (BP-C), 126.9 (BP-C), 64.8 (6-C), 31.2 (7-C).

^{11}B NMR (160 MHz, Benzene-d_6) δ(ppm): 38.95 (s).

HRMS (ASAP): m/z calcd for $C_{26}H_{24}BO$ [M+H]$^+$: 363.1920; found: 363.1905.

Compound 6d

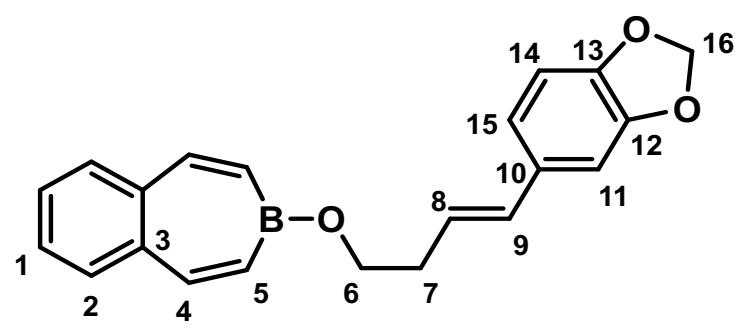

Following **general procedure A** with borepin **4** (90 mg, 0.51 mmol, 1 equiv.) and alcohol **5d** (0.51 mmol, 98 mg, 1 equiv.) afforded 143 mg (0.43 mmol, 85% yield) of compound **6d** as a white solid.

^{1}H NMR (500 MHz, Benzene-d_6) δ (ppm): 7.77 (d, *J* = 14.4 Hz, 2H, 4/4'-C*H*), 7.38 – 7.35 (m, 2H, 2/2'-C*H*), 7.14 – 7.10 (m, 2H, 1/1'-C*H*), 6.89 (s, 1H, 11-C*H*), 6.83 (d, *J* = 14.0 Hz, 2H, 5/5'-C*H*), 6.62 – 6.58 (m, 2H, 14,15-C*H*), 6.27 (d, *J* = 15.8 Hz, 1H, 9-C*H*), 5.96 (dt, *J* = 15.6, 7.1 Hz, 1H, 8-C*H*), 5.29 (s, 2H, 16-CH_2), 4.03 (t, *J* = 6.9 Hz, 2H, 6-CH_2), 2.45(qd, *J* = 6.9 Hz, 0.7 Hz, 2H, 7-CH_2).

^{13}C{^{1}H} NMR (126 MHz, Benzene-d_6) δ (ppm): 152.1 (4/4'-*C*), 148.2 (12-*C*), 147.0 (13-*C*), 137.3 (3/3'-*C*), 134.7 (2/2'-*C*), 133.8 (5/5'-*C*), 132.2 (10-*C*), 131.7 (9-*C*), 128.0 (1/1'-*C*), 124.6 (8-*C*), 120.6 (15-*C*), 108.1 (14-*C*), 105.6 (11-*C*), 100.5 (16-*C*), 65.0 (6-*C*), 35.4 (7-*C*).

^{11}B NMR (160 MHz, Benzene-d_6) δ (ppm): 38.89 (s).

HRMS (ASAP): m/z calcd for $C_{21}H_{20}BO_3$ $[M+H]^+$: 331.1506; found: 331.1516.

Compound 6e

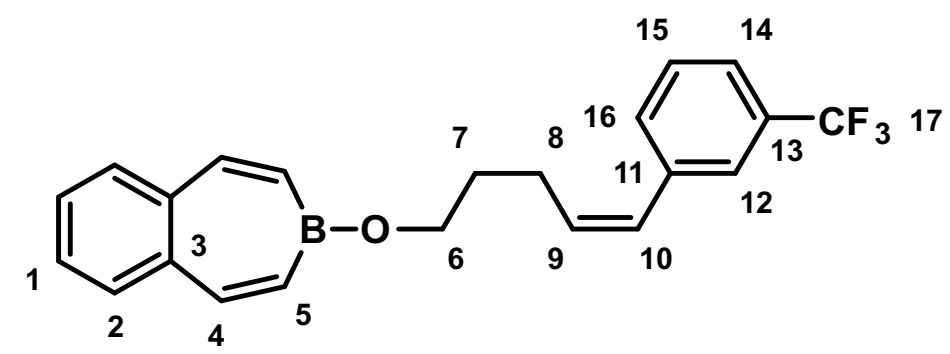

Following **general procedure A** with borepin **4** (90 mg, 0.51 mmol, 1 equiv.) and alcohol **5e** (117 mg, 0.51 mmol, 1.0 equiv.) afforded 182 mg (0.5 mmol, 97% yield) of compound **6e** as a white solid.

^{1}H NMR (500 MHz, Benzene-d_6) δ (ppm): 7.75 (d, J = 14.4 Hz, 2H, 4/4'-CH), 7.50 (s, 1H, 12-CH), 7.39 – 7.35 (m, 2H, 2/2'-CH), 7.21 (d, J = 7.9 Hz, 1H, 14-CH), 7.17 (d, J = 7.9 Hz, 1H, 16-CH), 7.14 – 7.11 (m, 2H, 1/1'-CH), 6.91 (t, J = 7.8 Hz, 1H, 15-CH), 6.74 (d, J = 14.0 Hz, 2H, 5-CH), 6.19 (d, J = 11.7 Hz, 1H, 10-CH), 5.54 (dt, J = 11.6, 7.4 Hz, 1H, 9-CH), 3.87 (t, J = 6.4 Hz, 2H, 6-CH_2), 2.27 (qd, J = 7.4, 1.8 Hz, 2H, 8-CH_2), 1.63 (p, J = 6.7 Hz, 2H, 7-CH_2).

^{13}C{^{1}H} NMR (126 MHz, Benzene-d_6) δ (ppm): 152.1 (4/4'-C), 138.3 (11-C), 137.3 (3/3'-C), 134.7 (2/2'-C), 133.8 (5/5'-C), 133.7 (9-C), 131.7 (16-C), 130.4 (q, $J_{C\text{-}F}$ = 31.9 Hz, 13-C), 128.5 (15-C), 128.0 (1/1'-C), 127.9 (10-C), 125.4 (q, $J_{C\text{-}F}$ = 3.7 Hz, 12-C), 124.6 (q, $J_{C\text{-}F}$ = 272.8 Hz, 17-C), 123.0 (q, $J_{C\text{-}F}$ = 3.7 Hz, 14-C), 64.3 (6-C), 31.5 (7-C), 24.8 (8-C).

^{19}F {^{1}H} NMR (471 MHz, Benzene-d_6) δ (ppm): -63.31.

^{11}B NMR (160 MHz, Benzene-d_6) δ (ppm): 38.80 (s).

HRMS (ASAP): m/z calcd for $C_{22}H_{21}BF_3O$ [M+H]$^+$: 369.1638; found: 369.1638.

Compound 6f

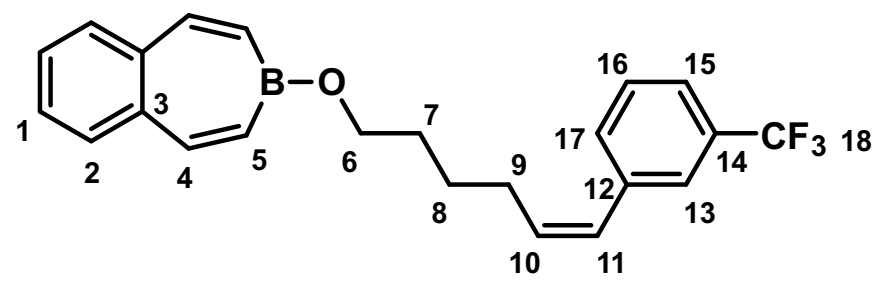

Following **general procedure A** with borepin **4** (90 mg, 0.51 mmol, 1 equiv.) and alcohol **5f** (125 mg, 0.51 mmol, 1 equiv.) afforded 185 mg (0.48 mmol, 95% yield) of compound **6f** as a white solid.

^{1}**H NMR** (500 MHz, Benzene-d_6) δ (ppm): δ 7.78 (d, J = 14.2 Hz, 2H, 4/4'-CH), 7.53 (s, 1H, 13-CH), 7.40 – 7.37 (m, 2H, 2/2'-CH), 7.24 (d, J = 7.8 Hz, 1H, 15-CH), 7.15 – 7.12 (m, 3H, 1/1'-CH, 17-CH), 6.94 (t, J = 7.8 Hz, 1H, 16-CH), 6.79 (d, J = 13.9 Hz, 2H, 5/5'-CH), 6.19 (d, J = 11.7 Hz, 1H, 11-CH), 5.53 (dt, J = 11.6, 7.3 Hz, 1H, 10-CH), 3.90 (t, J = 6.3 Hz, 2H, 6-CH_2), 2.13 (q, J = 7.0 Hz, 2H, 9-CH_2), 1.55 – 1.50 (m, 2H, 7-CH_2), 1.44 – 1.38 (m, 2H, 8-CH_2).

^{13}**C{^{1}H} NMR** (126 MHz, Benzene-d_6) δ (ppm): 152.1 (4/4'-C), 138.4 (12-C), 137.3 (3/3'-C), 134.7 (2/2'-C), 134.3 (10-C), 133.8 (5/5'-C), 131.7 (17-C), 130.4 (q, $J_{C\text{-}F}$ = 31.9 Hz, 14-C), 128.5 (16-C), 128.0 (1/1'-C), 127.6 (11-C), 125.4 (q, $J_{C\text{-}F}$ = 3.8 Hz, 13-C), 124.5 (q, $J_{C\text{-}F}$ = 272.9 Hz, 18-C), 123.0 (q, $J_{C\text{-}F}$ = 3.9 Hz, 15-C), 64.8 (6-C), 31.0 (7-C), 28.0 (9-C), 26.0 (8-C).

^{19}**F {^{1}H} NMR** (471 MHz, Benzene-d_6) δ (ppm): -62.32.

^{11}**B NMR** (160 MHz, Benzene-d_6) δ (ppm): 38.83 (s).

HRMS (ASAP): m/z calcd $C_{23}H_{23}BF_3O[M+H]^+$: 383.1794, found: 383.1783.

Compound 6g

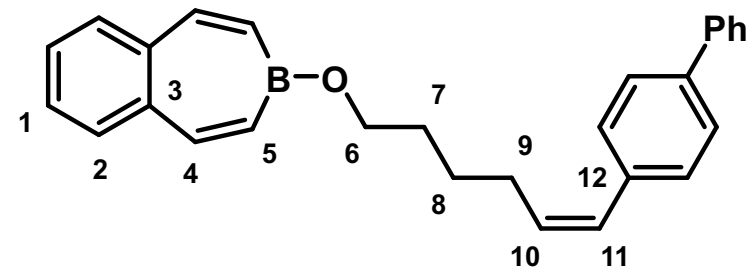

Following **general procedure A** with borepin **4** (90 mg, 0.51 mmol, 1 equiv.) and alcohol **5g** (129 mg, 0.51 mmol, 1 equiv.) afforded 185 mg (0.47 mmol, 93% yield) of compound **6g** as a white solid.

^{1}H NMR (500 MHz, Benzene-d_6) δ (ppm): δ 7.75 (d, J = 14.2 Hz, 2H, 4/4'-C*H*), 7.49 – 7.46 (m, 4H, BP-C*H*), 7.37 – 7.34 (m, 2H, 2/2'-C*H*), 7.31 (d, J = 8.2 Hz, 2H, BP-C*H*), 7.21 (t, J = 7.6 Hz, 2H, BP-C*H*), 7.14 – 7.09 (m, 3H, BP-C*H*, 1/1'-C*H*), 6.80 (d, J = 14.0 Hz, 2H, 5/5'-C*H*), 6.46 (d, J = 11.6 Hz, 1H, 11-C*H*), 5.61 (dt, J = 11.7, 7.3 Hz, 1H, 10-C*H*), 3.93 (t, J = 6.3 Hz, 2H, 6-CH_2), 2.34 (qd, J = 7.5, 1.6 Hz, 2H, 9-CH_2), 1.64 – 1.58 (m, 2H, 7-CH_2), 1.54 – 1.48 (m, 2H, 8-CH_2).
^{13}C{^{1}H} NMR (126 MHz, Benzene-d_6) δ (ppm): δ 152.1 (4/4'-*C*), 141.0 (BP-*Cq*), 139.4 (BP-*Cq*), 137.4 (3/3'-*C*), 136.8 (12-*C*), 134.7 (2/2'-*C*), 133.9 (5/5'-*C*), 132.8 (10-*C*), 129.3 (BP-*C*), 128.9 (11-*C*), 128.7 (BP-*C*), 128.0 (1/1'-*C*), 127.0 (BP-*C*), 127.0 (BP-*C*), 126.9 (BP-*C*), 64.8 (6-*C*), 31.1 (7-*C*), 28.3 (9-*C*), 26.2 (8-*C*).
^{11}B NMR (160 MHz, Benzene-d_6) δ (ppm): 38.81 (s).
HRMS (ASAP): m/z calcd $C_{28}H_{28}BO[M+H]^+$: 391.2223, found: 391.2215

Compound 6h

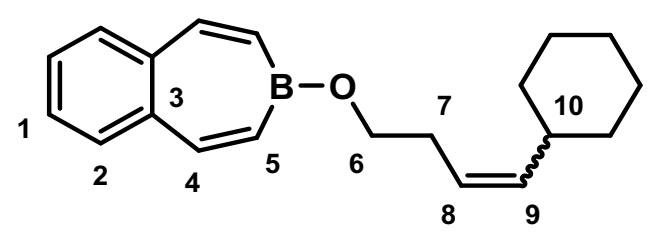

Following **general procedure A** with borepin **4** (90 mg, 0.51 mmol, 1.0 equiv.) and alcohol **5h** (0.51 mmol, 79 mg, 1.0 equiv.) afforded 120 mg (0.41 mmol, 81% yield) of compound **6h** as a colorless liquid.

Note: The borepin was obtained as a mixture of *cis*- and *trans*-olefin isomers in an approximate 2:1 ratio. In the ^{1}H NMR spectrum, the signals of the major and minor isomers overlap, whereas the ^{13}C NMR spectrum exhibits distinct sets of resonances for each isomer.

^{1}H NMR (400 MHz, Benzene-d_6) δ (ppm): 7.75 (d, *J* = 14.1 Hz, 2H, 4/4'-C*H*), 7.37 – 7.33 (m, 2H, 2/2'-C*H*), 7.12 – 7.08 (m, 2H, 1/1'-C*H*), 6.81 (d, *J* = 14.1 Hz, 2H, 5/5'-C*H*), 5.46 – 5.35 (m, 2H, 8, 9-C*H*), 4.06 – 3.99 (m, 2H, 6-CH_2), 2.47 – 2.36 (m, 2H, 7-CH_2), 2.36 – 2.28 and 1.89 – 1.81 (m, 1H, 10-C*H*), 1.69 – 1.52 (m, 5H, CyHex-CH_2), 1.24 – 0.97 (m, 5H, CyHex-CH_2),
^{13}C{^{1}H} NMR (101 MHz, Benzene-d_6) δ (ppm) **(Major isomer)**: 152.2 (4/4'-*C*), 138.8 (9-C), 137.3 (3/3'-*C*), 134.7 (2/2'-*C*), 133.9 (5/5'-*C*), 127.9 (1/1'-C), 123.6 (8-C), 65.3 (6-*C*), 40.7 (10-*C*), 35.3 (7-*C*), 33.0 (CyHex-*C*), 26.1 (2*CyHex-*C*), 26.0 (2*CyHex-*C*)
(Minor isomer): 152.2 (4/4'-*C*), 138.1 (9-C*H*), 137.4 (3/3'-*C*), 134.7 (2/2'-*C*), 133.9 (5/5'-*C*), 128.0 (1/1'-C*H*), 123.5 (8-C*H*), 65.1 (6-*C*), 36.4 (10-*C*), 30.2 (7-*C*), 33.3 (CyHex-*C*), 26.0 (2*CyHex-*C*), 25.9 (2*CyHex-*C*)
^{11}B NMR (128 MHz, Benzene-d_6) δ (ppm): 38.88 (s).
Physical State: colorless liquid.
HRMS (ASAP): m/z calcd for $C_{20}H_{26}BO$ $[M+H]^+$: 293.2077; found: 293.2087.

Compound 6i

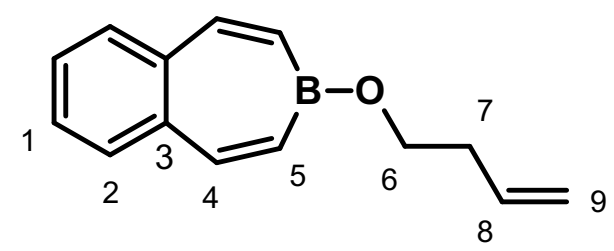

Following **general procedure A** with borepin **4** (90 mg, 0.51 mmol, 1.0 equiv.) and 3-buten-1-ol (0.51 mmol, 37.2 mg, 1.0 equiv.) afforded 103 mg (0.46 mmol, 95% yield) of compound **6i** as a colorless liquid.

^{1}H NMR (500 MHz, Benzene-*d*$_6$) δ (ppm): 7.75 (d, *J* = 14.4 Hz, 2H, 4/4'-C*H*), 7.38 – 7.36 (m, 2H, 2/2'-C*H*), 7.16 – 7.10 (m, 2H, 1/1'-C*H*), 6.79 (d, *J* = 14.3 Hz, 2H, 5/5'-C*H*), 5.85 – 5.77 (m, 1H, 8-C*H*), 5.07 – 5.04 (m, 2H, 9-CH_2), 3.98 (t, *J* = 6.9 Hz, 2H, 6-CH_2), 2.34 (q, *J* = 6.9 Hz, 2H, 7-CH_2).
^{13}C {^{1}H}-NMR (126 MHz, Benzene-*d*$_6$) δ (ppm): 152.2 (4/4'-*C*), 137.4 (3/3'-*C*), 134.9 (8-*C*), 134.7 (2/2'-*C*), 133.8 (5/5'-*C*), 128.1 (1/1'-*C*), 116.5 (9-*C*), 64.7 (6-*C*), 36.2 (7-*C*).
^{11}B NMR (160 MHz, Benzene-*d*$_6$) δ (ppm): 38.81.
HRMS (ASAP): m/z calcd for $C_{14}H_{16}BO$ $[M+H]^+$: 211.1294; found: 211.1288.

Compound 7a

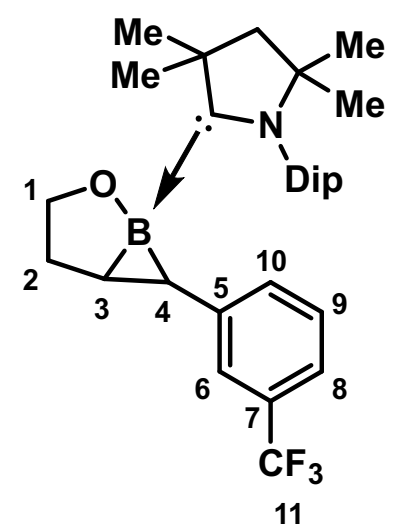

Following **general procedure B** with borepin-alkene **6a** (36 mg, 0.11 mmol, 1.0 equiv.) and ^{Me}CAAC (28.5 mg, 0.11 mmol, 1.0 equiv.) afforded 39 mg (0.076 mmol, 76% yield) of compound **7a** as a yellow solid. Single crystals suitable for X-ray crystallographic analysis were obtained by dissolving the compound in benzene and slowly evaporating the solution at room temperature.

Alternatively, following **general procedure B** with borepin-alkene **6b** (36 mg, 0.11 mmol, 1.0 equiv.) and ^{Me}CAAC (28.5 mg, 0.11 mmol, 1.0 equiv.) afforded 40.4 mg (0.079 mmol, 79% yield) of compound **7a** as a yellow solid. Single crystals suitable for X-ray crystallographic analysis were obtained by dissolving the compound in benzene and slowly evaporating the solution at room temperature.

^{1}H NMR (500 MHz, Benzene-d_6) δ (ppm): 7.91 (s, 1H, 6-C*H*), 7.56 (d, *J* = 7.6 Hz, 1H, 10-C*H*), 7.30 (d, *J* = 7.7 Hz, 1H, 8-C*H*), 7.11 – 7.05 (m, 2H, 9-C*H*, Dip-C*H*), 7.00 (ddd, *J* = 16.9, 7.7, 1.5 Hz, 2H, Dip-C*H*), 3.50 (t, *J* = 8.5 Hz, 1H, 1-CH_aH$_b$), 2.93 (sept, *J* = 6.7 Hz, 1H, *i*Pr-C*H*), 2.83 (q, *J* = 9.0 Hz, 1H, 1-CH$_a$$H_b$), 2.73 (sept, *J* = 6.7 Hz, 1H, *i*Pr-C*H*), 2.50 (d, *J* = 9.9 Hz, 1H, 4-C*H*), 2.28 (dd, *J* = 9.8, 5.4 Hz, 1H, 3-C*H*), 2.12 – 2.05 – 1.97 (m, 2H, 2-CH_2), 1.56 – 1.46 (m, 8H, CH_2^{CAAC}, C$_q$CH_3^{CAAC}, *i*Pr-CH_3), 1.28 – 1.27 (m, 6H, C$_q$CH_3^{CAAC}, *i*Pr-CH_3), 1.22 (d, *J* = 6.7 Hz, 3H, *i*Pr-CH_3), 1.15 (d, *J* = 6.7 Hz, 3H, *i*Pr-CH_3), 1.01 (s, 3H, C$_q$CH_3^{CAAC}), 0.81 (s, 3H, C$_q$CH_3^{CAAC}).
^{13}C{^{1}H} NMR (126 MHz, Benzene-d_6) δ (ppm): 224.3 (carbene-*C*q, identified by HMBC), 146.0 (5-*C*), 145.5 (Dip-*C*q), 145.3 (Dip-*C*q), 135.2 (Dip-*C*q), 133.9 (10-*C*), 129.8 (q, J_{C-F} = 31.2 Hz, 7-*C*), 128.4 (Dip-*C*), 127.7 (q, J_{C-F} = 4.0 Hz, 6-*C*), 127.7 (9-*C*), 125.4 (q, *J* = 255.6 Hz, 11-*C*), 124.3 (Dip-*C*), 123.6 (Dip-*C*), 120.7 (q, J_{C-F} = 4.0 Hz, 8-*C*), 73.0 (C_q(CH$_3$)$_2^{CAAC}$), 67.6 (1-*C*), 53.5 (*C*H$_2^{CAAC}$), 49.5 (C_q(CH$_3$)$_2^{CAAC}$), 45.8 (4-*C*), 40.8 (3-*C*), 30.6 (C$_q$(*C*H$_3$)$_2^{CAAC}$), 29.69 (C$_q$(*C*H$_3$)$_2^{CAAC}$), 29.62 (2-*C*), 29.2 (*i*Pr-*C*H), 28.9 (*i*Pr-*C*H), 28.3 (C$_q$(*C*H$_3$)$_2^{CAAC}$), 28.1 (C$_q$(*C*H$_3$)$_2^{CAAC}$), 25.4 (*i*Pr-*C*H$_3$), 25.0 (*i*Pr-*C*H$_3$), 24.4 (*i*Pr-*C*H$_3$), 23.6 (*i*Pr-*C*H$_3$).

^{19}F{^{1}H} NMR (471 MHz, Benzene-d_6) δ (ppm): -62.75.

^{11}B NMR (160 MHz, Benzene-d_6) δ (ppm): -6.39 (s).

HRMS (ESI): m/z calcd for $C_{31}H_{42}BF_3NO$ $[M+H]^+$: 512.3312; found: 512.3317.

From 6a:

Crystal data: $C_{31}H_{41}BF_3NO$, yellow chunk, 0.22 x 0.13 x 0.07 mm^3, triclinic *P-1*, a=9.6329(2)Å, b=10.2695(2)Å, c=28.4286(7)Å, α=94.763(2)°, β=90.654(2)°, γ=90.687(2)°, from 10240 reflections, 2Θ range for data collection 5.084° to 50.7°, T=100(2)K, V=2802.17(11)Å^3, Z=4, Fw=511.46, Dc=1.212 Mg·m^{-3}, μ=0.085 mm^{-1}.

Data collection and processing: Rigaku Synergy R diffractometer equipped with Hypix-Arc 100 detector, MoKα (λ=0.71073Å), -11≤h≤11, -12≤k≤12, 0≤l≤34, frame scan width = 0.25°, scan speed 1.0° per 20 sec, 10240 reflections collected, 10240 independent reflections. The data were processed with CrysAlis^{PRO}.

Solution and refinement: Structure solved with SHELXT program. Full matrix least-squares refinement based on F^2 with SHELXL on 697 parameters with no restraints gave final R_1= 0.0597 (based on F^2) for data with I>2σ(I), wR_2= 0.1752 on 10240 reflections, and final R_1= 0.0713 (based on F^2), wR_2= 0.1834 for all data; goodness-of-fit on F^2 = 1.075; largest electron density peak 0.54 e·Å^{-3}.Largest hole -0.34 e·Å^{-3}.

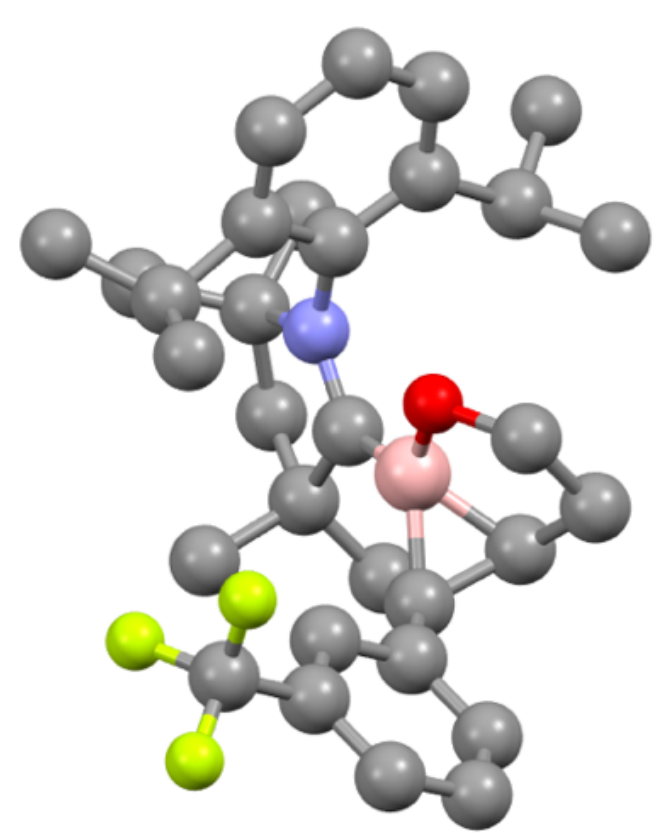

From 6b:

Crystal data: $C_{31}H_{41}BF_3NO$, yellow chunk, 0.277 x 0.205 x 0.101 mm^3, triclinic *P-1*, a=9.62850(10)Å, b=10.26480(10)Å, c=28.3885(4)Å, α=94.8400(10)°, β=90.7540(10)°, γ=90.9040(10)°, from 58537 reflections, 2Θ range for data collection 8.646° to 136.496°, T=100(2)K, V=2795.12(6)$Å^3$, Z=4, Fw=511.46, Dc=1.215 $Mg·m^{-3}$, μ=0.691 mm^{-1}.

Data collection and processing: Rigaku Synergy R diffractometer equipped with Hypix-Arc 150 detector, CuKα (λ=1.54184Å), -11≤h≤11, -12≤k≤12, -32≤l≤34, frame scan width = 0.5°, scan speed 1.0° per 0.1 s, 58537 reflections collected, 10222 independent reflections (R-int =0.0287). The data were processed with $CrysAlis^{PRO}$.

Solution and refinement: Structure solved with SHELXT program. Full matrix least-squares refinement based on F^2 with SHELXL on 696 parameters with no restraints gave final R_1= 0.0605 (based on F^2) for data with I>2σ(I), wR_2= 0.1754 on 10222 reflections, and final R_1= 0.0664 (based on F^2), wR_2= 0.1795 for all data; goodness-of-fit on F^2 = 1.068; largest electron density peak 0.45 $e·Å^{-3}$.Largest hole -0.40 $e·Å^{-3}$.

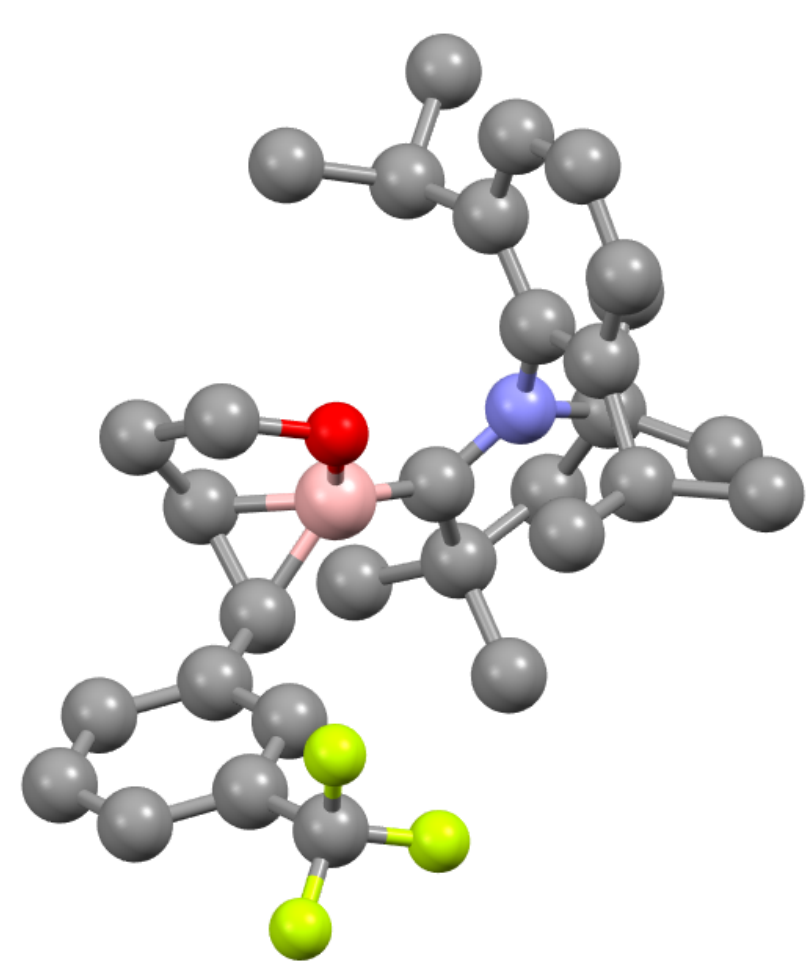

Compound 7c

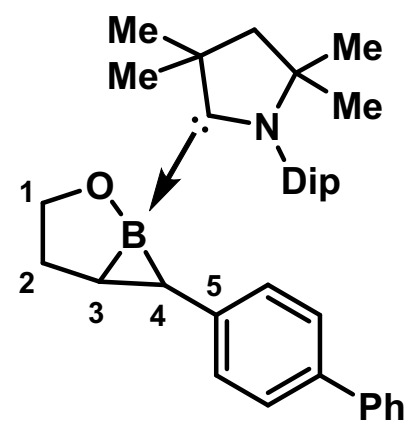

Following **general procedure B** with borepin-alkene **6c** (36 mg, 0.1 mmol, 1.0 equiv.) and ^{Me}CAAC (28.5 mg, 0.11 mmol, 1.0 equiv.) afforded 37 mg (0.071 mmol, 71% yield) of compound **7c** as a yellow solid.

^{1}H NMR (500 MHz, Benzene-d_6) δ (ppm): 7.64 – 7.63 (m, 2H, BP-C*H*), 7.60 – 7.56 (m, 4H, BP-C*H*), 7.22 (t, *J* = 7.6 Hz, 2H, BP-C*H*), 7.14 – 7.10 (m, 2H, Dip-C*H*, BP-C*H*), 7.07 (d, *J* = 7.7 Hz, 1H, Dip-C*H*), 7.02 (d, *J* = 7.6 Hz, 1H, Dip-C*H*), 3.65 – 3.62 (m, 1H, 1-CH_aH$_b$), 3.11 (q, *J* = 8.6 Hz, 1H, 1-CH$_a$$H_b$), 3.01 (sept, *J* = 6.6 Hz, 1H, *i*Pr-C*H*), 2.84 (sept, *J* = 6.7 Hz, 1H, *i*Pr-C*H*), 2.73 (d, *J* = 9.5 Hz, 1H, 4-C*H*), 2.41 – 2.37 (m, 1H, 3-C*H*), 2.23 – 2.13 (m, 2H, 2-CH_2), 1.62 – 1.52 (m, 8H, CH_2^{CAAC}, C$_q$CH_3^{CAAC}, *i*Pr-CH_3), 1.38 (s, 3H, C$_q$CH_3^{CAAC}), 1.34 (d, *J* = 6.6 Hz, 3H, *i*Pr-CH_3), 1.26 (d, *J* = 6.5 Hz, 3H, *i*Pr-CH_3), 1.18 (d, *J* = 6.7 Hz, 3H, *i*Pr-CH_3), 1.04 (s, 3H, C$_q$CH_3^{CAAC}), 0.87 (s, 3H, C$_q$CH_3^{CAAC}).

^{13}C{^{1}H} NMR (126 MHz, Benzene-d_6) δ (ppm): 223.3 (carbene-*C*q, identified by HMBC), 145.7 (Dip-*C*q), 145.6 (Dip-*C*q), 144.1 (5-*C*), 142.0 (BP-*Cq*), 137.0 (BP-*Cq*), 135.5 (Dip-*C*q), 131.2 (BP-*C*), 128.5 (BP-*C*), 128.2 (Dip-*C*), 126.9 (BP-*C*), 126.4 (BP-*C*), 126.3 (BP-*C*), 124.3 (Dip-*C*), 123.7 (Dip-*C*), 72.4 (C_q(CH$_3$)$_2$^{CAAC}), 67.4 (1-*C*), 53.8 (*C*H$_2$^{CAAC}), 49.4 (C_q(CH$_3$)$_2$^{CAAC}), 48.3 (4-*C*), 42.1 (3-*C*), 30.8 (C$_q$(*C*H$_3$)$_2$^{CAAC}), 29.8 (C$_q$(*C*H$_3$)$_2$^{CAAC}, 2-*C*), 29.2 (*i*Pr-*C*H), 28.9 (*i*Pr-*C*H), 28.5 (C$_q$(*C*H$_3$)$_2$^{CAAC}), 28.0 (C$_q$(*C*H$_3$)$_2$^{CAAC}), 25.7 (*i*Pr-*C*H$_3$), 25.0 (*i*Pr-*C*H$_3$), 24.5 (*i*Pr-*C*H$_3$), 23.8 (*i*Pr-*C*H$_3$).

^{11}B NMR (160 MHz, Benzene-d_6) δ (ppm): -5.81 (s).

HRMS (ASAP): m/z calcd for $C_{36}H_{47}BNO$ [M+H]$^+$: 520.3751; found: 520.3739.

Compound 7d

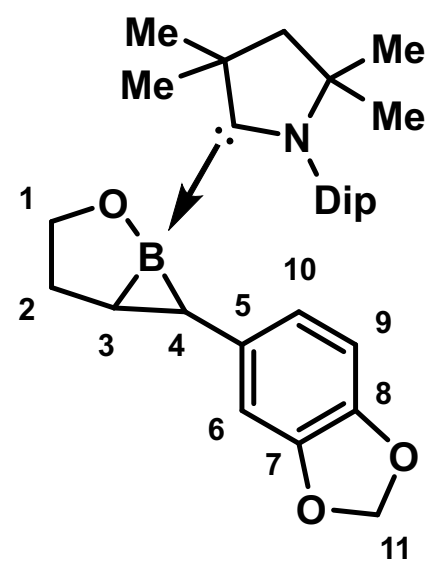

Following **general procedure B** with borepin-alkene **6d** (33 mg, 0.1 mmol, 1.0 equiv.) and ^{Me}CAAC (28.5 mg, 0.1 mmol, 1.0 equiv.) afforded 45 mg (0.085 mmol, 85% yield) of compound **7d** as a yellow solid.

^{1}H NMR (500 MHz, Benzene-d_6) δ (ppm): 7.15 (s, 1H, 6-C*H*), 7.09 (t, *J* = 7.6 Hz, 1H, Dip-C*H*), 7.04 – 6.98 (m, 3H, Dip-C*H*, 10-C*H*), 6.79 (d, *J* = 7.8 Hz, 1H, 9-C*H*), 5.37 (d, *J* = 3.6 Hz, 2H, 11-CH_2), 3.58 – 3.54 (m, 1H, 1-CH_aH$_b$), 3.01 – 2.94 (m, 2H, 1-CH$_a$$H_b$ and *i*Pr-C*H*), 2.78 (sept, *J* = 8.0 Hz, 1H, *i*Pr-C*H*), 2.61 (d, *J* = 9.5 Hz, 1H, 4-C*H*), 2.31 (d, *J* = 7.3 Hz, 1H, 3-C*H*), 2.11 – 2.07 (m, 2H, 2-CH_2), 1.59 – 1.49 (m, 8H, CH_2^{CAAC}, C$_q$CH_3^{CAAC}, *i*Pr-CH_3), 1.31 – 1.29 (m, 6H, C$_q$CH_3^{CAAC}, *i*Pr-CH_3), 1.22 (d, *J* = 6.6 Hz, 3H, *i*Pr-CH_3), 1.15 (d, *J* = 6.7 Hz, 3H, *i*Pr-CH_3), 1.02 (s, 3H, C$_q$CH_3^{CAAC}), 0.82 (s, 3H, C$_q$CH_3^{CAAC}).

^{13}C{^{1}H} NMR (126 MHz, Benzene-d_6) δ (ppm): 223.5 (carbene-*C*q, identified by HMBC), 147.3 (7-*C*), 145.66 (Dip-*C*q), 145.63 (Dip-*C*q), 144.9 (8-*C*), 138.1 (5-*C*), 135.5 (Dip-*C*q), 128.2 (Dip-*C*), 124.2 (Dip-*C*), 123.56 (Dip-*C*/10-*C*), 123.51(Dip-*C*/10-*C*), 111.7 (6-*C*), 107.7 (9-*C*), 100.0 (11-*C*), 72.3 (C_q(CH$_3$)$_2^{CAAC}$), 67.2 (1-*C*), 53.9 (*C*H$_2^{CAAC}$), 49.3 (C_q(CH$_3$)$_2^{CAAC}$), 47.8 (4-*C*), 42.7 (3-*C*), 30.8 (C$_q$(*C*H$_3$)$_2^{CAAC}$), 29.8 (C$_q$(*C*H$_3$)$_2^{CAAC}$), 29.7 (2-*C*), 29.2 (*i*Pr-*C*H), 28.9 (*i*Pr-*C*H), 28.4 (C$_q$(*C*H$_3$)$_2^{CAAC}$), 28.1 (C$_q$(*C*H$_3$)$_2^{CAAC}$), 25.6 (*i*Pr-*C*H$_3$), 25.0 (*i*Pr-*C*H$_3$), 24.5 (*i*Pr-*C*H$_3$), 23.7 (*i*Pr-*C*H$_3$).

^{11}B NMR (160 MHz, Benzene-d_6) δ (ppm): -6.17 (s).

HRMS (ESI): m/z calcd for $C_{31}H_{43}BNO_3$ [M+H]$^+$: 488.3336; found: 488.3349.

Compound 7e

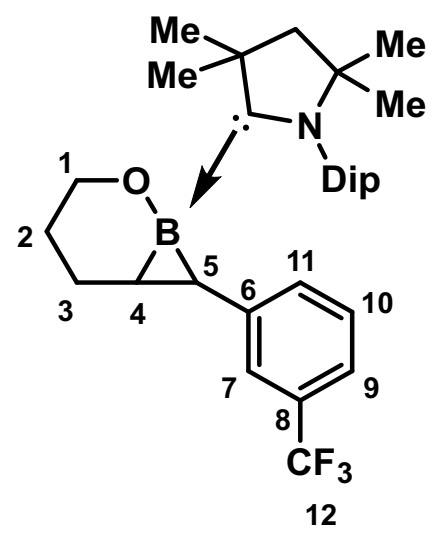

Following **general procedure B** with borepin-alkene **6e** (37 mg, 0.1 mmol, 1.0 equiv.) and ^{Me}CAAC (28.5 mg, 0.1 mmol, 1.0 equiv.) afforded 38 mg (0.072 mmol, 72% yield) of compound **7e** as a yellow solid. Single crystals suitable for X-ray crystallographic analysis were obtained by dissolving the compound in benzene, which was then stored at −30 °C, affording yellow crystals.

^{1}H NMR (500 MHz, Benzene-d_6) δ (ppm): 8.05 (s, 1H, 7-C*H*), 7.68 (d, *J* = 7.7 Hz, 1H, 11-C*H*), 7.34 (d, *J* = 7.7 Hz, 1H, 9-C*H*), 7.13 (t, *J* = 7.7 Hz, 1H, 10-C*H*), 7.09 – 7.03 (m, 2H, Dip-C*H*), 6.98 (dd, *J* = 7.3, 2.0 Hz, 1H, Dip-C*H*), 3.28 (d, *J* = 9.2 Hz, 1H, 1-CH_aH$_b$), 2.89 – 2.77 (m, 2H, *i*Pr-C*H*, 1-CH$_a$$H_b$), 2.68 (sept, *J* = 6.9 Hz, 1H, *i*Pr-C*H*), 2.23 (d, *J* = 11.5 Hz, 1H, 5-C*H*), 2.03 – 1.99 (m, 1H, 3-CH_aH$_b$), 1.86 – 1.78 (m, 1H, 3-CH$_a$$H_b$), 1.65 (s, 3H, C$_qCH_3^{CAAC}$), 1.56 – 1.48 (m, 8H, C$_qCH_3^{CAAC}$, *i*Pr-C$H_3$, C$H_2^{CAAC}$), 1.36 (d, *J* = 6.6 Hz, 3H, *i*Pr-CH_3), 1.16 (d, *J* = 6.6 Hz, 3H, *i*Pr-CH_3), 1.07 (d, *J* = 6.6 Hz, 3H, *i*Pr-CH_3), 0.98 – 0.92 (m, 2H, 2-CH_aH$_b$ 4-C*H*), 0.906 (s, 3H, C$_q$CH_3^{CAAC}), 0.902 (s, 3H, C$_q$CH_3^{CAAC}), 0.80 – 0.72 (m, 1H, 2-CH$_a$$H_b$)

^{13}C{^{1}H} NMR (126 MHz, Benzene-d_6) δ (ppm): 233.6 (carbene-*C*q, identified by HMBC), 146.7 (Dip-*C*q), 146.5 (6-*C*), 144.2 (Dip-*C*q), 136.0 (Dip-*C*q), 135.4 (11-*C*), 129.7 (q, J_{C-F} = 31.1 Hz, 8-*C*), 129.1 (q, J_{C-F} = 3.7 Hz, 7-*C*), 128.5 (Dip-*C*), 127.6 (10-*C*), 125.5 (q, J_{C-F} = 272.6 Hz, 12-*C*), 124.8 (Dip-*C*), 124.6 (Dip-*C*), 120.6 (q, J_{C-F} = 3.9 Hz, 9-*C*), 73.1 (C_q(CH$_3$)$_2^{CAAC}$), 60.6 (1-*C*), 54.0 (*C*H$_2^{CAAC}$), 51.0 (C_q(CH$_3$)$_2^{CAAC}$), 45.3 (5-*C*), 30.3 (4-C), 30.0 (C$_q$(*C*H$_3$)$_2^{CAAC}$), 29.4 (C$_q$(*C*H$_3$)$_2^{CAAC}$), 29.2 (*i*Pr-*C*H), 28.9 (*i*Pr-*C*H), 28.6 (C$_q$(*C*H$_3$)$_2^{CAAC}$), 27.8 (C$_q$(*C*H$_3$)$_2^{CAAC}$), 26.0 (2-*C*), 25.1 (*i*Pr-*C*H$_3$), 24.9 (*i*Pr-*C*H$_3$), 24.8 (*i*Pr-*C*H$_3$), 24.5 (*i*Pr-*C*H$_3$), 22.2 (3-*C*).

^{19}F {^{1}H} NMR (471 MHz, Benzene-d_6) δ (ppm): -62.79.

^{11}B NMR (160 MHz, Benzene-d_6) δ (ppm): -10.01 (s).

HRMS (ASAP): m/z calcd for $C_{32}H_{44}BNOF_3$ [M+H]$^+$: 526.3468; found: 526.3456.

Crystal data: $C_{32}H_{43}BF_3NO$, yellow chunk, 0.44 x 0.37 x 0.25 mm^3, monoclinic $P2_1/n$, a=10.5152(2)Å, b=9.3472(1)Å, c=29.6217(5)Å, α=90°, β=91.439(2)°, γ=90°, from 73257 reflections, 2Θ range for data collection 4.078° to 66.372°, T=100(2)K, V=2910.53(8)Å^3, Z=4, Fw=525.48, Dc=1.199 Mg·m^{-3}, μ=0.084 mm^{-1}.

Data collection and processing: Rigaku Synergy R diffractometer equipped with Hypix-Arc 100 detector, MoKα (λ=0.71073Å), -15≤h≤15, -13≤k≤14, -42≤l≤45, frame scan width = 0.25°, scan speed 1.0° per 1.0 sec, 73257 reflections collected, 10215 independent reflections (R-int =0.0456). The data were processed with CrysAlis^{PRO}.

Solution and refinement: Structure solved with SHELXT program. Full matrix least-squares refinement based on F^2 with SHELXL on 351 parameters with no restraints gave final R_1= 0.0552 (based on F^2) for data with I>2σ(I), wR_2= 0.1484 on 10215 reflections, and final R_1= 0.0754 (based on F^2), wR_2= 0.1641 for all data; goodness-of-fit on F^2 = 1.038; largest electron density peak 0.55 e·Å^{-3}.Largest hole -0.42 e·Å^{-3}.

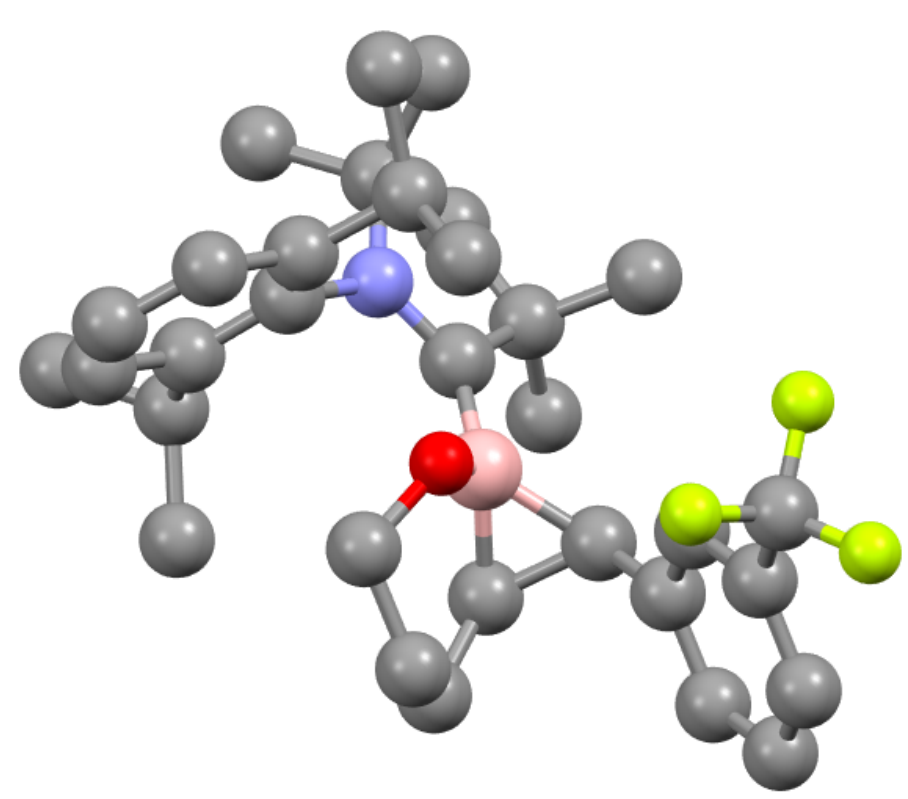

Compound 7f

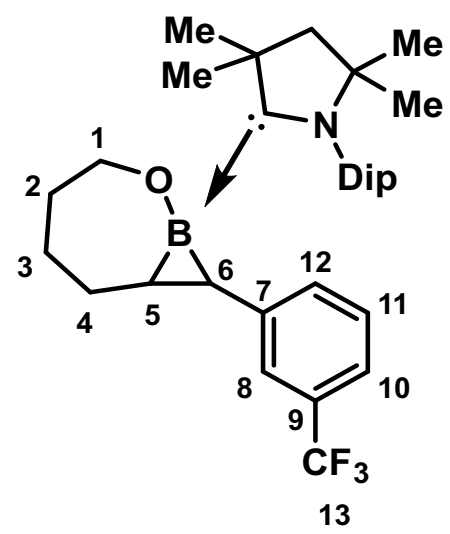

Following **general procedure B** with borepin-alkene **6f** (38 mg, 0.1 mmol, 1.0 equiv.) and ^{Me}CAAC (28.5 mg, 0.1 mmol, 1.0 equiv.) afforded 36.1 mg (0.067 mmol, 67% yield) of compound **7f** as a yellow solid. Single crystals suitable for X-ray crystallographic analysis were obtained by dissolving the compound in benzene, which was then kept at -30 °C.

^{1}H NMR (500 MHz, Benzene-d_6) δ (ppm): 8.27 (s, 1H, 8-C*H*), 7.93 (d, *J* = 7.8 Hz, 1H, 12-C*H*), 7.30 (d, *J* = 7.7 Hz, 1H, 10-C*H*), 7.19 – 7.16 (m, 1H, 11-C*H*), 7.07 – 6.98 (m, 3H, Dip-C*H*), 4.13 (d, *J* = 11.4 Hz, 1H, 1-CH_aH$_b$), 3.74 (t, *J* = 11.4 Hz, 1H, 1-CH$_a$$H_b$), 3.03 (sept, *J* = 6.5 Hz, 1H, *i*Pr-C*H*), 2.77 (sept, *J* = 6.5 Hz, 1H, *i*Pr-C*H*), 2.13 (d, *J* = 11.7 Hz, 1H, 6-C*H*), 2.06 – 1.97 (m, 1H, 2-CH_aH$_b$), 1.85 – 1.66 (m, 4H, 2-CH$_a$$H_b$, 3-$H_aH_b$, 4-C$H_2$), 1.64 (s, 3H, C$_qCH_3$^{CAAC}), 1.51 (d, *J* = 6.7 Hz, 3H, *i*Pr-CH_3), 1.48 – 1.39 (m, 9H, 3-CH$_a$$H_b$, C$H_2$^{CAAC}, C$_qCH_3$^{CAAC}, *i*Pr-C$H_3$), 1.10 – 1.06 (m, 9H, C$_qCH_3$^{CAAC}, *i*Pr-C$H_3$), 0.82 – 0.76 (m, 1H, 5-C*H*), 0.65 (s, 3H, C$_q$CH_3^{CAAC}).

^{13}C{^{1}H} NMR (126 MHz, Benzene-d_6) δ (ppm): 234.3 (carbene-*C*q, identified by HMBC), 147.0 (7-*C*), 146.5 (Dip-*C*q), 144.7 (Dip-*C*q), 135.0 (Dip-*C*q), 132.5 (12-*C*), 129.3 (q, $J_{C\text{-}F}$ = 30.6 Hz, 9-*C*), 128.9 (Dip-*C*), 127.3 (11-*C*), 125.8 (q, $J_{C\text{-}F}$ = 3.9 Hz, 8-*C*) 125.6 (q, $J_{C\text{-}F}$ = 272.6 Hz, 13-*C*), 125.3 (Dip-*C*), 125.0 (Dip-*C*) 119.2 (q, $J_{C\text{-}F}$ = 3.9 Hz, 10-*C*), 73.5 (C_q(CH$_3$)$_2$^{CAAC}), 68.9 (1-*C*), 55.0 (6-C), 53.4 (*C*H$_2$^{CAAC}), 52.5 (C_q(CH$_3$)$_2$^{CAAC}), 35.8 (2-*C*), 34.4 (5-*C*), 30.8 (C$_q$(*C*H$_3$)$_2$^{CAAC}), 30.5 (C$_q$(*C*H$_3$)$_2$^{CAAC}), 30.0 (3-*C*), 29.4 (*i*Pr-*C*H), 28.8 (C$_q$(*C*H$_3$)$_2$^{CAAC}), 27.8 (*i*Pr-*C*H), 26.7 (C$_q$(*C*H$_3$)$_2$^{CAAC}), 26.6 (*i*Pr-*C*H$_3$), 25.9 (*i*Pr-*C*H$_3$), 25.3 (4-*C*), 24.7 (*i*Pr-*C*H$_3$), 24.1 (*i*Pr-*C*H$_3$).

^{19}F {^{1}H} NMR (471 MHz, Benzene-d_6) δ (ppm): -62.81.

^{11}B NMR (160 MHz, Benzene-d_6) δ (ppm): -4.28 (s).

HRMS (ASAP): m/z calcd for $C_{33}H_{46}BNOF_3$ [M+H]$^+$: 540.3635; found: 540.3643.

Crystal data: $C_{33}H_{45}BF_3NO$, yellow irregular, 0.07 x 0.10 x 0.14 mm^3, monoclinic $P2_1/c$ a=9.38323(11)Å, b=9.97177(11)Å, c=31.6139(3)Å, α=90°, β=92.5635(3) °, γ=90° from 32832 reflections, T=100.0(2)K, V=2955.07(6)Å^3, Z=4, Fw=539.51, Dc=1.213 Mg·m^{-3}, μ=0.678 mm^{-1}.

Data collection and processing: Rigaku Synergy R diffractometer equipped with a HyPix-Arc 150° detector, CuKα (λ=1.54184Å), -11≤h≤11, -12≤k≤12, -39≤l≤39, 77940 reflections collected, 6016 independent reflections (R-int =0.0309). The data were processed with CrysAlis^{PRO}.

Solution and refinement: Structure solved with SHELXT program. Full matrix least-squares refinement based on F^2 with SHELXL on 361 parameters with 0 restraints gave final R_1= 0.0423 (based on F^2) for data with I>2σ(I), wR_2= 0.1026 on 6016 reflections, and final R_1= 0.0489 (based on F^2), wR_2= 0.1070 for all data; goodness-of-fit on F^2 = 0.959. Largest electron density peak 0.251 e·Å^{-3}. Largest hole -0.259 e·Å^{-3}.

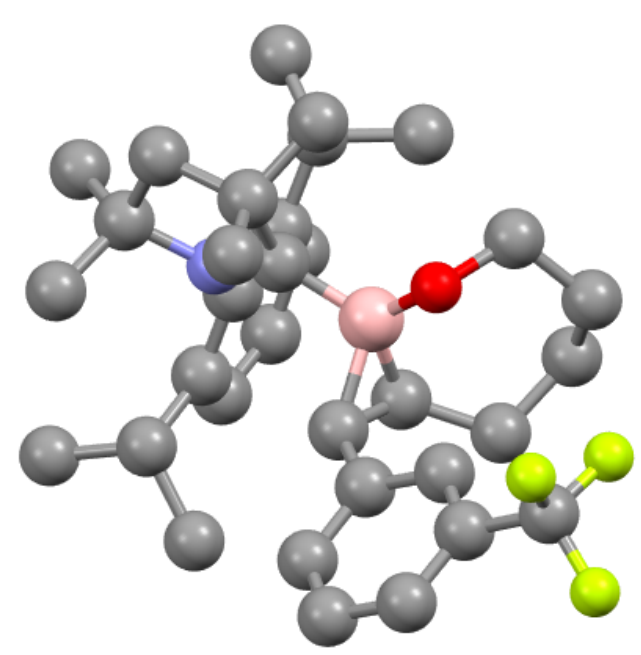

Compound 7g

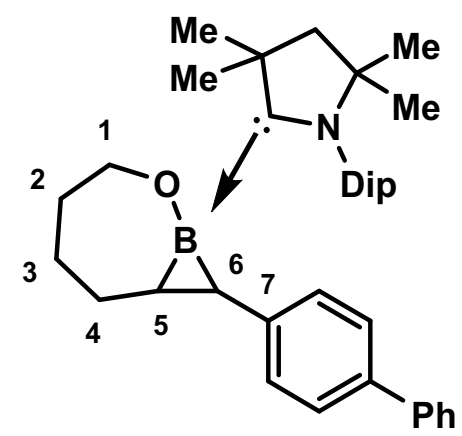

Following **general procedure B** with borepin-alkene **6g** (39 mg, 0.1 mmol, 1.0 equiv.) and ^{Me}CAAC (28.5 mg, 0.1 mmol, 1.0 equiv.) afforded 42.0 mg (0.078 mmol, 78% yield) of compound **7g** as a yellow crystalline solid.

^{1}H NMR (500 MHz, Benzene-d_6) δ (ppm): δ 7.92 (d, J = 8.4 Hz, 2H, BP-CH), 7.66 – 7.61 (m, 4H, BP-CH), 7.21 (t, J = 7.7 Hz, 2H, BP-CH), 7.11 – 7.03 (m, 4H, BP-CH, Dip-CH), 4.19 (d, J = 11.3 Hz, 1H, 1-CH_aH$_b$), 3.82 (t, J = 11.4 Hz, 1H, 1-CH$_a$$H_b$), 3.20 (sept, J = 6.5 Hz, 1H, iPr-CH), 2.86 (sept, J = 6.6 Hz, 1H, iPr-CH), 2.35 (d, J = 11.7 Hz, 1H, 6-CH), 2.09 – 2.02 (m, 2H, 2, 4-CH_aH$_b$), 1.81 – 1.73 (m, 3H, 2, 4-CH$_a$$H_b$, 3-C$H_aH_b$), 1.71 (s, 3H, C$_qCH_3$^{CAAC}), 1.55 – 1.45 (m, 12H, C$_q$CH_3^{CAAC}, 2*iPr-CH_3, CH_2^{CAAC}, 3-CH$_a$$H_b$), 1.18 (s, 3H, C$_qCH_3$^{CAAC}), 1.15 (d, J = 6.5 Hz, 3H, iPr-CH_3), 1.11 (d, J = 6.7 Hz, 3H, iPr-CH_3), 0.97 – 0.91 (m, 1H), 0.69 (s, 3H, C$_q$CH_3^{CAAC}).

^{13}C{^{1}H} NMR (126 MHz, Benzene-d_6) δ (ppm): 231.2 (carbene-Cq, identified by HMBC), 146.8 (Dip-Cq), 145.0 (7-C), 144.9 (Dip-Cq), 142.1 (BP-Cq), 135.6 (Dip-Cq), 135.5 (BP-Cq), 129.9 (BP-C), 128.8 (BP-C), 128.6 (BP-C), 126.7 (BP-C), 126.1 (Dip-C), 125.9 (BP-C), 125.3 (Dip-C), 124.9 (Dip-C), 72.6 (C_q(CH$_3$)$_2$^{CAAC}), 69.0 (1-C), 58.6 (6-C), 54.0 (CH$_2$^{CAAC}), 52.1 (C_q(CH$_3$)$_2$^{CAAC}), 35.9 (2-C), 35.6 (5-C), 31.2 (C$_q$(CH$_3$)$_2$^{CAAC}), 30.6 (C$_q$(CH$_3$)$_2$^{CAAC}), 30.3 (3-C), 29.4 (iPr-CH), 28.8 (C$_q$(CH$_3$)$_2$^{CAAC}), 27.8 (iPr-CH), 26.7 (C$_q$(CH$_3$)$_2$^{CAAC}), 26.5 (iPr-CH$_3$), 26.1 (iPr-CH$_3$), 25.6 (4-C), 24.6 (iPr-CH$_3$), 24.2 (iPr-CH$_3$).

^{11}B NMR (160 MHz, Benzene-d_6) δ (ppm): -2.89 (s).

HRMS (ASAP): m/z calcd for $C_{38}H_{51}BNO$ $[M+H]^+$: 548.4064; found: 548.4088.

Compound 7h

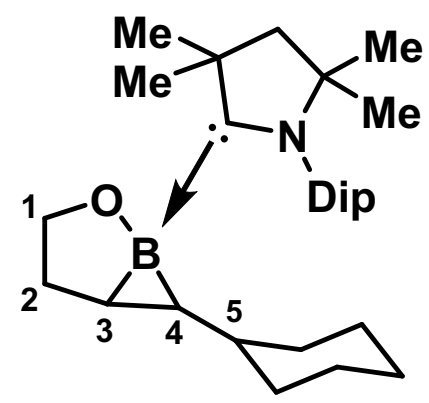

Following **general procedure B** with borepin-alkene **6e** (29 mg, 0.1 mmol, 1.0 equiv.) and ^{Me}CAAC (28.5 mg, 0.1 mmol, 1.0 equiv.) afforded 29.2 mg (0.065 mmol, 65% yield) of compound **7h** as a yellow solid.

^{1}H NMR (500 MHz, Benzene-d_6) δ (ppm): δ 7.17 – 7.14 (m, 1H, Dip-C*H*), 7.11 – 7.09 (m, 1H, Dip-C*H*), 7.04 (dd, *J* = 7.5, 1.5 Hz, 1H, Dip-C*H*), 3.68 (s, 2H, 1-CH_2), 3.01 (sept, *J* = 6.7 Hz, 1H, iPr-C*H*), 2.81 (sept, *J* = 6.7 Hz, 1H, iPr-C*H*), 2.41 (broad s, 1H, 4/3-C*H*), 2.13 – 2.02 (m, 4H, 2-CH_2, CyHex), 1.89 – 1.64 (m, 4H, 3-C*H*, CyHex), 1.64 – 1.44 (m, 10H, 5-C*H*, CH_2^{CAAC}, C_qCH_3^{CAAC}, *i*Pr-CH_3, CyHex), 1.42 – 1.17 (m, 16H, *i*Pr-CH_3, CyHex), 1.10 (s, 3H, C_qCH_3^{CAAC}), 0.85 (s, 3H, C_qCH_3^{CAAC}).

^{13}C{^{1}H}-NMR (126 MHz, Benzene-d_6) δ (ppm): 146.4 (Dip-*C*q), 145.6 (Dip-*C*q), 136.4 (Dip-*C*q), 124.1(2*Dip-*C*), 123.4 (Dip-*C*q), 70.8 ($C_q(CH_3)_2^{CAAC}$), 70.7 (1-*C*), 54.6 (CH_2^{CAAC}), 48.1 ($C_q(CH_3)_2^{CAAC}$), 45.7 (3,4-*C*), 37.2 (5-*C*), 36.2 (CyHex), 35.8 (CyHex), 31.5 ($C_q(CH_3)_2^{CAAC}$), 29.9 ($C_q(CH_3)_2^{CAAC}$), 29.3 (*i*Pr-*C*H), 29.2 (2-*C*), 28.9 (*i*Pr-*C*H), 28.3 ($C_q(CH_3)_2^{CAAC}$), 28.0 ($C_q(CH_3)_2^{CAAC}$), 27.28 (CyHex), 27.27 (CyHex), 27.1 (CyHex), 25.4 (*i*Pr-CH_3), 25.1 (*i*Pr-CH_3), 24.8 (*i*Pr-CH_3), 23.8 (*i*Pr-CH_3).

^{11}B NMR (160 MHz, Benzene-d_6) δ (ppm): -6.24.

HRMS (ASAP): m/z calcd for $C_{30}H_{49}BNO$ $[M+H]^+$: 450.3907; found: 450.3925.

Cyclizative borylation

Compound 8

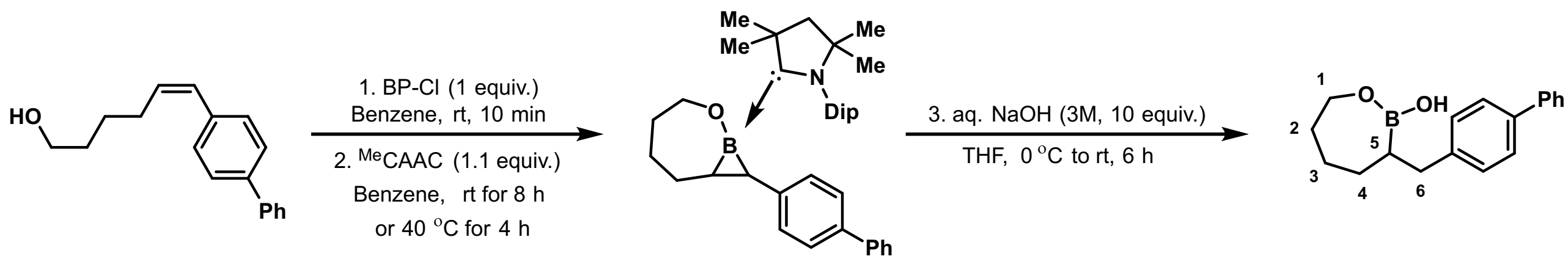

Crude product **7g**

In a nitrogen-filled glovebox, a 4 mL screw-capped glass vial was charged with alcohol **5g** (25.2 mg, 0.1 mmol, 1 equiv.) and dissolved in 0.5 mL of anhydrous benzene. To this solution, borepin **4** (17.4 mg, 0.1 mmol, 1 equiv.) in benzene (0.5 mL) was added dropwise at room temperature. After 10 minutes, the volatile components were removed under reduced pressure. (**borepin formation 6g**)

The crude mixture was then dissolved in 0.5 mL of anhydrous benzene, and a solution of ^{Me}CAAC (31.4 mg, 0.096 mmol, 1.1 equiv.) in benzene (0.5 mL) was added at room temperature. The reaction mixture was stirred at room temperature for 8 h or at 40 °C oil bath for 4 h. Afterward, the solvent was removed under reduced pressure. (**borirane formation 7g**)

The vial was removed from the glovebox, THF (1 mL) was added, and the mixture was cooled to 0 °C in an ice bath. Aqueous NaOH (3 M, 0.3 mL, 10 equiv.) was added, vial was sealed under nitrogen atmosphere and reaction mixture was stirred while warming to room temperature. After 6 h upon completion, reaction mixture was diluted with water and extracted with ethyl acetate (3 x 5 mL). The combined organic layers were washed with water, dried over Na_2SO_4, filtered, and concentrated in vacuo. The crude residue was subjected to column chromatography and 5 mg of alcohol **5g** was recovered along with the desired product. The obtained product was further purified by preparative HPLC to afford the product **8** as a colorless oil. Yield: 11.2 mg (40%, 50% brsm)

^{1}H NMR (500 MHz, Chloroform-*d*) δ (ppm): 7.61 (d, *J* = 7.6 Hz, 2H, BP), 7.54 (d, *J* = 7.8 Hz, 2H, BP), 7.45 (t, *J* = 7.5 Hz, 2H, BP), 7.36 – 7.30 (m, 3H, BP), 4.27 (s, 1H, OH), 4.03 (dd, *J* = 11.9, 5.6 Hz, 1H, 1-CH_aH$_b$), 3.93 (t, *J* = 10.8 Hz, 1H, 1-CH$_a$$H_b$), 3.06 (dd, *J* = 14.1, 5.5 Hz, 1H, 6-CH_aH$_b$), 2.56 (dd, *J* = 14.1, 8.8 Hz, 1H, 6-CH$_a$$H_b$), 1.95 – 1.93 (m, 1H), 1.82 – 1.79 (m, 1H), 1.71 – 1.69 (m, 1H), 1.63 – 1.58 (m, 1H), 1.48 – 1.41 (m, 2H), 1.38 – 1.33 (m, 1H) (2-5, CH_2).

^{13}C {^{1}H} NMR (126 MHz, Chloroform-*d*) δ (ppm): 141.8 (BP-C_q), 141.1 (BP-C_q), 138.5 (BP-C_q), 129.3 (BP-*C*), 128.7 (BP-*C*), 127.0 (BP-*C*), 126.97 (BP-*C*), 126.92 (BP-*C*), 66.4 (1-*C*), 37.1 (6-*C*), 31.4, 29.9, 28.8 (2-4), 27.6 (5-*C,* identified by HSQC).

^{11}B NMR (160 MHz, Chloroform-*d*) δ (ppm): 33.17 (brs).

TLC: R_f = 0.4 (Hexane:EtOAc 3:1)

HRMS (ASAP): m/z calcd for $C_{18}H_{20}BO_2$ [M-H]$^-$: 279.1556; found: 279.1557.

Compound 9

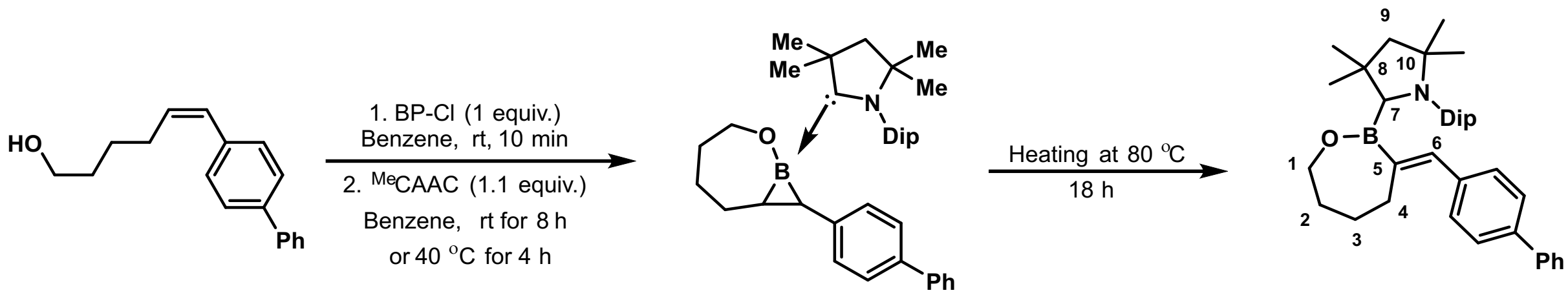

Crude product **7g**

In a nitrogen-filled glovebox, a 4 mL screw-capped glass vial was charged with alcohol **5g** (25.2 mg, 0.1 mmol, 1 equiv.) and dissolved in 0.5 mL of anhydrous benzene. To this solution, borepin **4** (17.4 mg, 0.1 mmol, 1 equiv.) in benzene (0.5 mL) was added dropwise at room temperature. After 10 minutes, the volatile components were removed under reduced pressure. (**borepin formation 6g**)

The crude mixture was then dissolved in 0.5 mL of anhydrous benzene, and a solution of ^{Me}CAAC (31.4 mg, 0.096 mmol, 1.1 equiv.) in benzene (0.5 mL) was added at room temperature. The reaction mixture was stirred at room temperature for 8 h or at 40 °C oil bath for 4 h. (**borirane formation 7g**)

Then, sealed vial was shifted to 80 °C oil bath and kept at this temperature for 18 h. Afterward, the solvent was removed under reduced pressure, and the crude residue was washed with dry cold pentane. The solvent was then removed to obtain the vinyl borane **9**. NMR yield: 55% (using 1,3,5-trimethoxybenzene as internal standard).

^{1}H NMR (500 MHz, Benzene-d_6) δ (ppm): 7.52 – 7.48 (m, 4H, BP-C*H*), 7.34 (d, *J* = 8.1 Hz, 2H, BP-C*H*), 7.28 – 7.19 (m, 4H, BP-C*H,* Dip-C*H*), 7.16 – 7.11 (m, 2H, Dip-C*H*), 7.03 (s, 1H, 6-C*H*), 4.32 (sept, *J* = 6.8 Hz, 1H, *i*Pr-CH^{Dip}), 4.18 (s, 1H, 7-C*H*), 3.58 – 3.49 (m, 2H, *i*Pr-CH^{Dip}, 1-CH_aH$_b$), 3.37 (td, *J* = 11.5, 2.4 Hz, 1H, 1-CH$_a$$H_b$), 2.61 – 2.57 (m, 1H, 4-CH_aH$_b$), 2.11 – 2.06 (m, 1H, 4-CH$_a$$H_b$), 1.86 (d, J_{AB} = 12.3 Hz, 1H, 9-CH_aH$_b$), 1.80 (d, J_{AB} = 12.3 Hz, 1H, 9-CH$_a$$H_b$), 1.61 – 1.56 (m, 1H), 1.45 – 1.41 (m, 10H), 1.37 (s, 3H), 1.34 (d, *J* = 6.7 Hz, 4H), 1.29 – 1.28 (m, 6H), 1.22 – 1.77 (m, 4H) (2,3, *i*Pr-CH_3^{Dip}, 8 and 10-CH_3).

^{13}C {^{1}H} NMR (126 MHz, Benzene-d_6)) δ (ppm): 152.3 (Dip-C_q), 149.5 (Dip-C_q), 145.5 (5-*C*), 141.4 (BP-C_q), 140.9 (BP-C_q), 139.9 (BP-C_q), 139.0 (6-*C*) 137.1 (Dip-C_q), 129.5 (BP-*C*), 128.8 (Dip-*C*), 127.0 (BP-*C*), 126.9 (BP-*C*), 125.6 (BP-*C*), 123.9 (Dip-*C*), 123.7 (Dip-*C*), 67.5 (7-*C*),

66.1 (1-*C*), 62.1 (10-*C*), 59.1 (9-*C*), 40.9 (8-*C*), 30.4, 29.8, 29.4, 29.0, 28.7, 27.9, 26.4, 25.9, 25.5, 25.1, 24.5, 24.2 (2-5, *i*Pr-*C*H_3^{Dip}, 8 and 10-*C*H_3).

^{11}**B NMR** (160 MHz, Benzene-d_6) δ (ppm): 49.19 (brs).

HRMS (ASAP): m/z calcd for $C_{38}H_{51}BNO$ $[M+H]^+$: 548.4064; found: 548.4052.

Compound 10

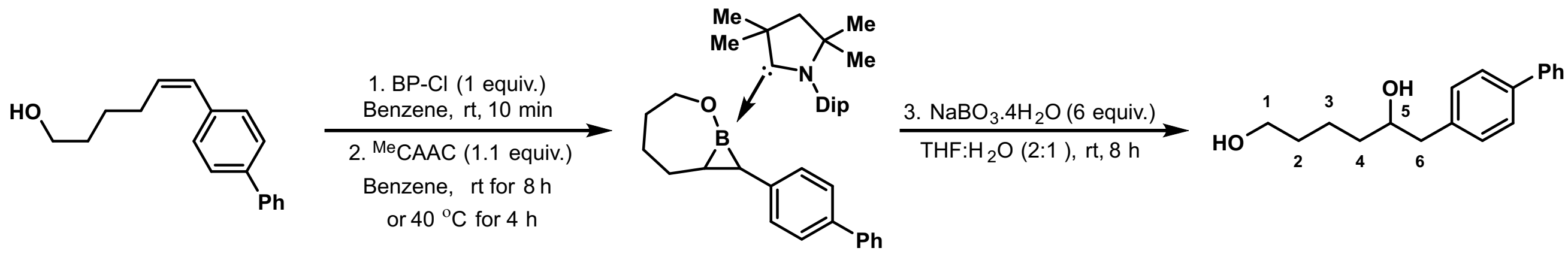

Crude product **7g**

In a nitrogen-filled glovebox, a 4 mL screw-capped glass vial was charged with alcohol **5g** (22 mg, 0.087 mmol, 1 equiv.) and dissolved in 0.5 mL of anhydrous benzene. To this solution, borepin **4** (15.2 mg, 0.087 mmol, 1 equiv.) in benzene (0.5 mL) was added dropwise at room temperature. After 10 minutes, the volatile components were removed under reduced pressure. (**borepin formation 6g**)

The crude mixture **6g** was then dissolved in 0.5 mL of anhydrous benzene, and a solution of ^{Me}CAAC (27.4 mg, 0.096 mmol, 1.1 equiv.) in benzene (0.5 mL) was added at room temperature. The reaction mixture was stirred at room temperature for 8 h or at 40 °C for 4 h. (**borirane formation 7g**)

Afterward, the solvent was removed under reduced pressure, and the vial containing the crude mixture was taken outside the glovebox. The residue was dissolved in degassed THF:H_2O (2:1, 1.5 mL), and $NaBO_3 \cdot 4H_2O$ (80 mg, 0.52 mmol, 6 equiv.) was added. The vial was sealed under nitrogen atmosphere and stirred at room temperature for 8 h. The reaction mixture was then diluted with water and extracted with ethyl acetate (3 x 5 mL). The combined organic layers were washed with water, dried over Na_2SO_4, filtered, and concentrated in vacuo. The crude product was purified by preparative TLC using hexane/ethyl acetate (1:1) as the eluent to afford white solid diol **10** (14 mg) and 2.5 mg of alcohol **5g** was also recovered. Isolated yield: 59% (67% brsm)

^{1}H NMR (500 MHz, Chloroform-*d*) δ (ppm): 7.61 – 7.56 (m, 4H, BP-C*H*), 7.46 (t, *J* = 7.7 Hz, 2H, BP-C*H*), 7.38 – 7.34 (m, 1H, BP-C*H*), 7.31 (d, *J* = 8.1 Hz, 2H, BP-C*H*), 3.92 – 3.88 (m, 1H, 5-C*H*), 3.69 (t, *J* = 5.9 Hz, 2H, 1-CH_2), 2.89 (dd, *J* = 13.6, 4.2 Hz, 1H, 6-C$H_a$$H_b$), 2.73 (dd, *J* = 13.6, 8.4 Hz, 1H, 6-C$H_a$$H_b$), 1.74 (brs, 1H, O-*H*), 1.66 – 1.49 (m, 7H, 2-4 CH_2, O-*H*)

^{13}C {^{1}H} NMR (126 MHz, Chloroform-*d*) δ (ppm): 140.9 (BP-*Cq*), 139.4 (BP-*Cq*), 137.6 (BP-*Cq*), 129.8 (BP-*C*), 128.7 (BP-*C*), 127.3 (BP-*C*), 127.2 (BP-*C*), 127.0 (BP-*C*), 72.5 (5-*C*), 62.7 (1-*C*), 43.7 (6-*C*), 36.4, 32.5, 21.9 (2-4 *C*).

TLC: R_f = 0.6 (Hexane:EtOAc 1:1)

HRMS (ESI): m/z calcd for $C_{18}H_{22}O_2Na$ $[M+Na]^+$: 293.1517; found: 293.1513.

Compound 11

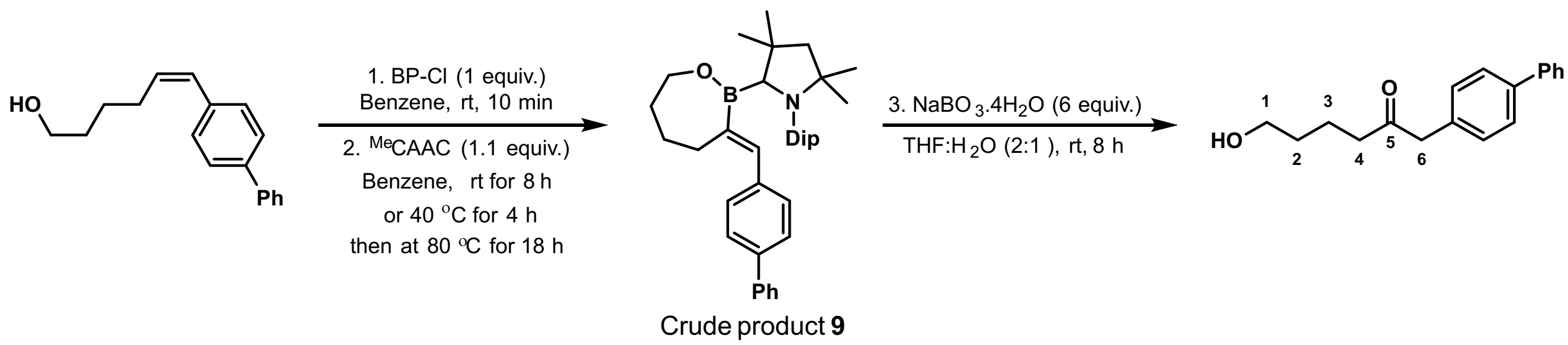

In a nitrogen-filled glovebox, a 4 mL screw-capped glass vial was charged with alcohol **5g** (22 mg, 0.087 mmol, 1 equiv.) and dissolved in 0.5 mL of anhydrous benzene. To this solution, borepin **4** (15.2 mg, 0.087 mmol, 1 equiv.) in benzene (0.5 mL) was added dropwise at room temperature. After 10 minutes, the volatile components were removed under reduced pressure. (**borepin formation 6g**).

The crude mixture was then dissolved in 0.5 mL of anhydrous benzene, and a solution of ^{Me}CAAC (27.4 mg, 0.096 mmol, 1.1 equiv.) in benzene (0.5 mL) was added at room temperature. The reaction mixture was stirred at room temperature for 8 h or at 40 ^{0}C for 4 h (**borirane formation 7g**). Then, reaction was shifted to 80 ^{0}C oil bath and kept at this for 18 h. (**vinyl borane formation 9**)

Afterward, the solvent was removed under reduced pressure, and the vial containing the crude mixture was taken outside the glovebox. The residue was dissolved in degassed THF:H_2O (2:1, 1.5 mL), and $NaBO_3·4H_2O$ (80 mg, 0.52 mmol, 6 equiv.) was added. The vial was sealed under nitrogen atmosphere and stirred at room temperature for 8 h. The reaction mixture was then diluted with water and extracted with ethyl acetate (3 x 5 mL). The combined organic layers were washed with water, dried over Na_2SO_4, filtered, and concentrated in vacuo. The crude product was purified by preparative TLC using hexane/ethyl acetate (3:1) as the eluent to afford the white solid keto alcohol **11** (15 mg) and 3 mg of alcohol **5g** was also recovered. Isolated yield: 64% (74% brsm) obtained as mixtures of ketone and hemiketal (3.3:1).

^{1}H NMR (500 MHz, Chloroform-*d*) δ (ppm): 7.62 – 7.56 (m, 4H, BP), 7.46 (t, *J* = 7.8 Hz, 2H, BP), 7.39 – 7.36 (m, 1H, BP), 7.30 (d, *J* = 7.9 Hz, 2H, BP), 3.76 (s, 2H, 6-C*H*$_2$), 3.61 (t, *J* = 6.3 Hz, 2H, 1-C*H*$_2$), 2.57 (t, *J* = 7.1 Hz, 2H, 4-C*H*$_2$), 1.72 – 1.65 (m, 2H), 1.58 – 1.52 (m, 2H) (2,3-C*H*$_2$).

^{13}C {^{1}H} NMR (126 MHz, Chloroform-*d*) δ (ppm): 208.4 (5-*C*), 140.7 (BP-C_q), 140.0 (BP-C_q),133.2 (BP-C_q), 131.3 (BP-*C*), 129.8 (BP-*C*), 127.4 (BP-*C*), 127.3 (BP-*C*), 127.0 (BP-*C*), 62.3 (1-*C*), 49.7 (6-*C*), 41.6, 32.0, 19.6 (2-4 *C*).

TLC: R_f = 0.4 (Hexane:EtOAc 3:1)

HRMS (ESI): m/z calcd for $C_{18}H_{20}O_2Na$ $[M+Na]^+$: 291.1361; found: 291.1356.

Compound 12

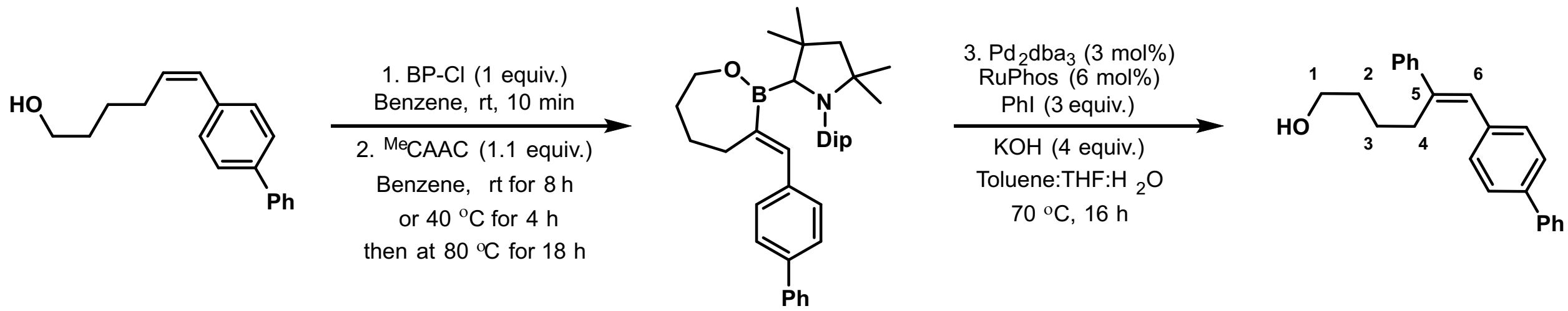

Crude product **9**

In a nitrogen-filled glovebox, a 4 mL glass vial was charged with alcohol **5g** (25.2 mg, 0.1 mmol, 1 equiv.) and dissolved in 0.5 mL of anhydrous benzene. To this solution, borepin **4** (17.4 mg, 0.1 mmol, 1 equiv.) in benzene (0.5 mL) was added dropwise at room temperature. After 10 minutes, the volatile components were removed under reduced pressure. (**borepin formation 6g**)

The crude mixture was then dissolved in 0.5 mL of anhydrous benzene, and a solution of ^{Me}CAAC (31.4 mg, 0.11 mmol, 1.1 equiv.) in benzene (0.5 mL) was added at room temperature. The reaction mixture was stirred at room temperature for 8 h or at 40 °C for 4 h, (**borirane formation 7g**) and then shifted to 80 °C oil bath and kept at this temperature for 18 h. Afterward, the solvent was removed under reduced pressure. (**vinyl borane formation 9**)

Then, separate 4 mL vial was charged with $Pd_2(dba)_3$ (3 mg, 0.003 mmol, 3 mol%), RuPhos (3 mg, 0.006 mmol, 6 mol%) and KOH (22 mg, 0.4 mmol, 4 equiv). Then, THF (0.5 mL) and toluene (1.0 mL) were added and the reaction mixture was stirred at room temperature for 30 min under nitrogen. A solution of vinyl borane **9** in THF (0.5 mL) was added, followed by addition of iodobenzene (61.2 mg, 0.3 mmol, 3.0 equiv). The vial containing the reaction mixture was removed from a glove-box and the degassed water (sparged with N_2 for 1 h, 0.2 mL) was added. The reaction mixture was stirred at 70 °C for 16 h under nitrogen atmosphere. After cooling to room temperature, the reaction was quenched with water and extracted with ethyl acetate (3 x 5 mL). The organic layers were combined, washed with brine, dried over Na_2SO_4 and concentrated under reduced pressure. The crude product was purified by preparative TLC using hexane/ethyl acetate (3:1) as the eluent to afford **12** (14.8 mg, 45%) as a white solid.

^{1}H NMR (300 MHz, Chloroform-*d*) δ (ppm): 7.67 – 7.63 (m, 4H), 7.52 – 7.32 (m, 10H), 6.77 (s, 1H, 6-C*H*), 3.61 (t, *J* = 6.2 Hz, 2H, 1-CH_2), 2.82 (t, *J* = 7.5 Hz, 2H, 4-CH_2), 1.767 – 1.52 (m, 5H, 2,3-CH_2, O*H*).

^{13}C NMR (101 MHz, Chloroform-*d*) δ (ppm): 143.1, 142.9, 140.7 (BP-C_q), 139.3 (BP-C_q), 137.2 (BP-C_q), 129.2, 128.8, 128.4, 128.1, 127.3, 127.0, 126.9, 126.6, 62.6 (1-*C*), 32.6 (2-*C*), 29.9 (4-*C*), 24.9 (3-*C*).

TLC: R_f = 0.35 (Hexane:EtOAc 4:1)

HRMS (ASAP): m/z calcd for $C_{24}H_{24}O$ $[M]^+$: 328.1827; found: 328.1832.

Compound 14

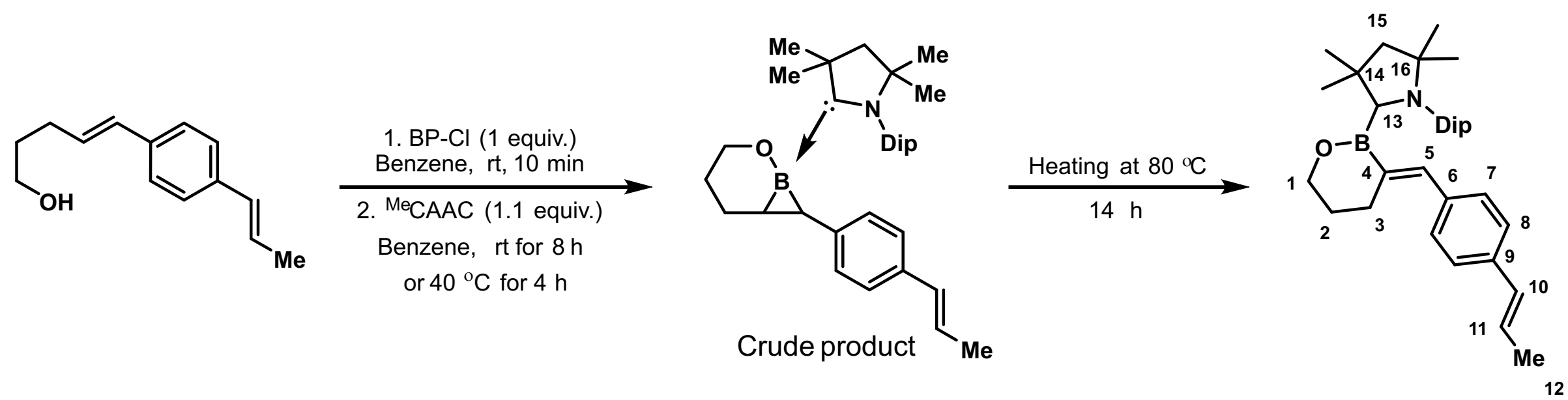

In a nitrogen-filled glovebox, a 4 mL screw-capped glass vial was charged with diene alcohol **13** (20.2 mg, 0.1 mmol, 1 equiv.) and dissolved in 0.5 mL of anhydrous benzene. To this solution, borepin **4** (17.4 mg, 0.1 mmol, 1 equiv.) in benzene (0.5 mL) was added dropwise at room temperature. After 10 minutes, the volatile components were removed under reduced pressure. (**borepin formation**)

The crude mixture was then dissolved in 0.5 mL of anhydrous benzene, and a solution of ^{Me}CAAC (31.4 mg, 0.11 mmol, 1.1 equiv.) in benzene (0.5 mL) was added at room temperature. The reaction mixture was stirred at room temperature for 8 h or at 40 °C for 4 h. (**borirane formation**)

The reaction mixture was shifted to 80 °C oil bath for 14 h. The solvent was then removed under reduced pressure, and the residue was dissolved in pentane (3 mL) and filtered through a syringe filter. Removal of the solvent afforded crude vinyl borane **14**, which was redissolved in pentane (2 mL) and allowed to evaporate slowly at room temperature, yielding pale yellow crystals of **14**. These crystals were used directly for single-crystal X-ray diffraction analysis. NMR yield: 64% (using 1,4-dioxane as internal standard).

^{1}H NMR (500 MHz, Benzene-d_6) δ (ppm): 7.45 (s, 1H, 5-C*H*), 7.30 – 7.24 (m, 5H, 7/7', 8/8'-C*H*, Dip-C*H*), 7.18 – 7.09 (m, 1H, Dip-C*H*), 7.09 (dd, *J* = 7.7, 1.7 Hz, 1H, Dip-C*H*), 6.32 – 6.29 (m, 1H, 10-C*H*), 6.11 – 6.04 (m, 1H, 11-C*H*), 4.43 (sept, *J* = 6.9 Hz, 1H, iPr-CH^{Dip}), 4.36 (s, 1H), 3.74 – 3.70 (m, 1H, 1-CH_aH$_b$), 3.61 (sept, *J* = 12.9, 6.5 Hz, 1H, iPr-CH^{Dip}), 3.43 – 3.38 (m, 1H, 1-CH$_a$$H_b$), 2.30 – 2.19 (m, 2H, 3-CH_2), 1.94 (d, J_{AB} = 12.3 Hz, 1H, 15-CH_aH$_b$), 1.82 (d, J_{AB} = 12.3 Hz, 1H, 15-CH$_a$$H_b$), 1.67 (dd, *J* = 6.6, 1.5 Hz, 3H, 12-CH_3), 1.45 – 1.43 (m, 6H), 1.39 – 1.38 (m, 6H), 1.35 – 1.32 (m, 9H), 1.26 – 1.13 (m, 5H, 2-CH_2)

^{13}**C {^{1}H} NMR** (126 MHz, Benzene-*d*$_6$) δ (ppm): 152.6 (Dip-*Cq*), 149.9 (Dip-*Cq*), 143.6 (5-C), 141.5 (Dip-*Cq*), 138.9 (4-*C*), 137.4 (9-*C*), 136.4 (6-*C*), 130.9 (10-*C*), 130.0 (7/7'-*C*), 125.8 (Dip-*C*), 125.7 (8/8'-*C*, 11-*C*), 124.1 (2*Dip-*C*), 64.6 (1-*C*), 64.3 (13-*C*), 62.7 (16-*C*), 59.4 (15-*C*), 40.9 (14-*C*), 30.7, 30.4, 29.0 (iPr-*C*H^{Dip}), 28.8, 27.6 (iPr-*C*H^{Dip}), 27.4 (3-*C*), 26.8 (2-*C*), 26.3, 26.2, 25.7, 25.1, 24.4, 18.2 (12-*C*).

^{11}**B NMR** (160 MHz, Benzene-*d*$_6$) δ (ppm): 45.73 (brs).

HRMS (ESI): m/z calcd for $C_{34}H_{49}BNO$ [M+H]$^+$: 498.3907; found: 498.3906.

Compound 14:

Crystal data: $C_{34}H_{48}BNO$, yellow chunk, 0.232 x 0.142 x 0.106 mm^3, monoclinic *P2₁/c*, a=19.7983(2)Å, b=13.0228(1)Å, c=11.7125(1)Å, α=90°, β=95.677(1)°, γ=90°, from 101061 reflections, 2Θ range for data collection 8.138° to 149.796°, T=100(2)K, V=3005.01(4)$Å^3$, Z=4, Fw=497.54, Dc=1.100 $Mg{\cdot}m^{-3}$, μ=0.480 mm^{-1}.

Data collection and processing: Rigaku Synergy R diffractometer equipped with Hypix-Arc 150 detector, CuKα (λ=1.54184Å), -24≤h≤24, -16≤k≤14, -14≤l≤14, frame scan width = 0.5°, scan speed 1.0° per 0.10 sec, 101061 reflections collected, 6133 independent reflections (R-int =0.0356). The data were processed with $CrysAlis^{PRO}$.

Solution and refinement: Structure solved with SHELXT program. Full matrix least-squares refinement based on F^2 with SHELXL on 364 parameters with no restraints gave final R_1= 0.0403 (based on F^2) for data with I>2σ(I), wR_2= 0.1023 on 6133 reflections, and final R_1= 0.0446 (based on F^2), wR_2= 0.1057 for all data, goodness-of-fit on F^2 = 1.041 largest electron density peak 0.27 $e{\cdot}Å^{-3}$.Largest hole -0.22 $e{\cdot}Å^{-3}$.

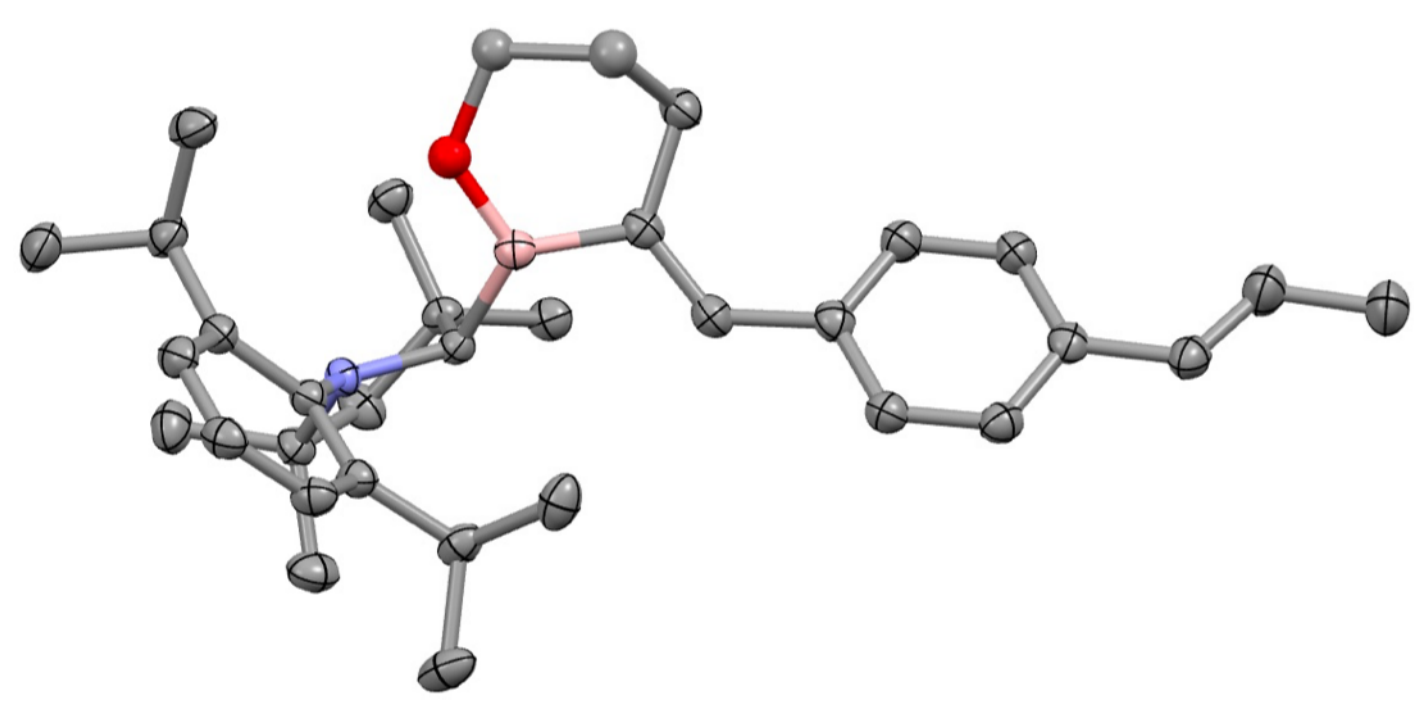

Compound 15

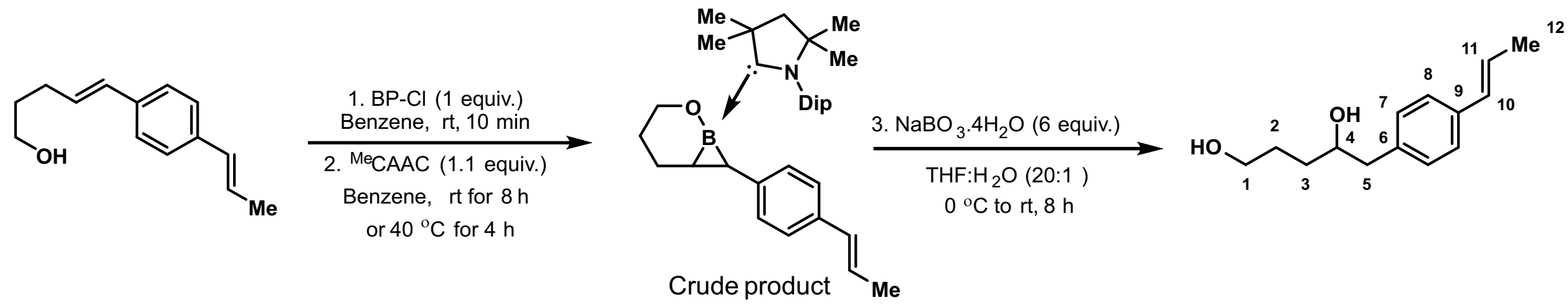

In a nitrogen-filled glovebox, a 4 mL screw-capped glass vial was charged with diene alcohol **13** (20.2 mg, 0.1 mmol, 1 equiv.) and dissolved in 0.5 mL of anhydrous benzene. To this solution, borepin **4** (17.4 mg, 0.1 mmol, 1 equiv.) in benzene (0.5 mL) was added dropwise at room temperature. After 10 minutes, the volatile components were removed under reduced pressure. (**borepin formation**)

The crude mixture was then dissolved in 0.5 mL of anhydrous benzene, and a solution of ^{Me}CAAC (31.4 mg, 0.11 mmol, 1.1 equiv.) in benzene (0.5 mL) was added at room temperature. The reaction mixture was stirred at room temperature for 8 h or at 40 °C for 4 h. Then, solvent was removed was removed under reduced pressure and vial was taken outside of glovebox. (**borirane formation**)

Next, the residue was dissolved in degassed THF (1 mL) and cooled to ice bath. Then, $NaBO_3 \cdot 4H_2O$ (92 mg, 0.6 mmol, 6 equiv.) and degassed water (0.05 mL) was added sequentially at the same temperature. The vial was sealed under nitrogen atmosphere and stirred for 8 h while warming slowly to room temperature. The reaction mixture was then diluted with water and extracted with ethyl acetate (3 x 5 mL). The combined organic layers were washed with water, dried over Na_2SO_4, filtered, and concentrated in vacuo. The crude product was purified by preparative TLC using hexane/ethyl acetate (3:1) as the eluent to afford the white solid diol **15** (12 mg) and 5 mg of alcohol **13** was also recovered. Isolated yield: 54% (73% brsm)

^{1}H NMR (500 MHz, Chloroform-*d*) δ (ppm): 7.29 (d, *J* = 8.1 Hz, 2H), 7.15 (d, *J* = 7.9 Hz, 2H), 6.39 (d, *J* = 15.8, 1H, 10-C*H*), 6.26 – 6.19 (m, 1H, 11-C*H*), 3.88 – 3.83 (m, 1H, 4-C*H*), 3.72 – 3.63 (m, 2H, 1-CH_2), 2.80 (dd, *J* = 13.6, 4.6 Hz, 1H, 5-C$H_a$$H_b$), 2.70 (dd, *J* = 13.5, 8.3 Hz, 1H, 5-C$H_a$$H_b$), 2.42 (brs, 2H, O*H*), 1.89 (dd, *J* = 6.6, 1.3 Hz, 3H, 12-CH_3), 1.76 – 1.69 (m, 3H, 2, 3-CH_2), 1.59 – 1.52 (m, 1H, 2 or 3-CH_2).

^{13}**C NMR** (126 MHz, Chloroform-*d*) δ (ppm): 136.8 (6-*C*), 136.3 (9-*C*), 130.6 (10-*C*), 129.5 (7/7'-*C*), 126.0 (8/8'-*C*, 125.4 (11-*C*), 72.6 (4-*C*), 62.9 (1-*C*), 43.8 (5-*C*), 33.7, 29.2 (2, 3-*C*), 18.4 (12-*C*).

TLC: R_f = 0.5 (Hexane:EtOAc 1:1)

HRMS (ESI): m/z calcd for $C_{14}H_{20}O_2Na$ $[M+H]^+$: 243.1361; found: 243.1354.

Compound 17

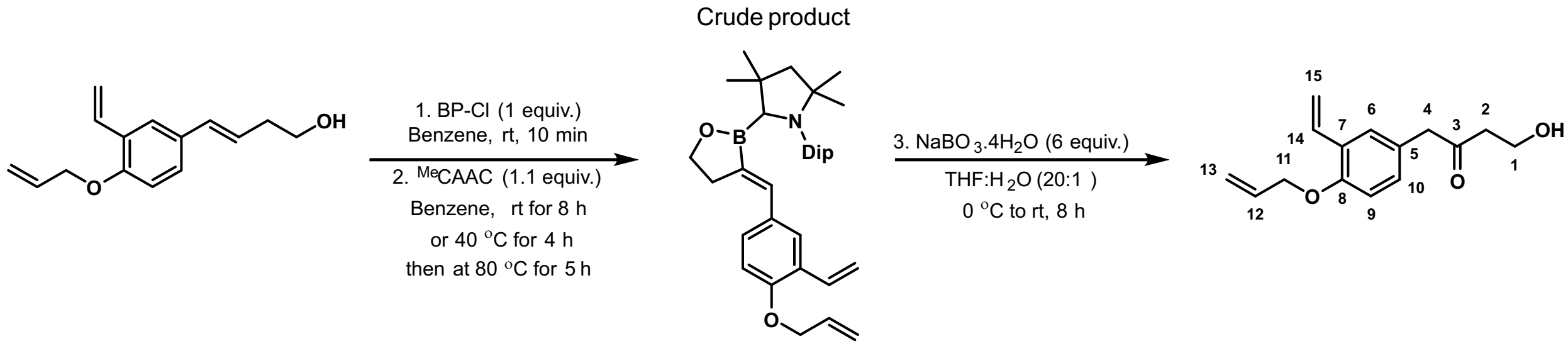

In a nitrogen-filled glovebox, a 4 mL screw-capped glass vial was charged with alcohol **16** (23 mg, 0.1 mmol, 1 equiv.) and dissolved in 0.5 mL of anhydrous benzene. To this solution, borepin **4** (17.4 mg, 0.1 mmol, 1 equiv.) in benzene (0.5 mL) was added dropwise at room temperature. After 10 minutes, the volatile components were removed under reduced pressure. (**borepin formation**)

The crude mixture was then dissolved in 0.5 mL of anhydrous benzene, and a solution of ^{Me}CAAC (32.7 mg, 0.114 mmol, 1.1 equiv.) in benzene (0.5 mL) was added at room temperature. The reaction mixture was stirred at room temperature for 8 h or at 40 °C for 4 h. (**borirane formation**). Then, reaction was shifted to 80 ^{o}C oil bath and kept at this for 5 h. (**vinyl borane formation**)

Next, the residue was dissolved in degassed THF (1 mL) and cooled to ice bath. Then, $NaBO_3 \cdot 4H_2O$ (96 mg, 0.6 mmol, 6 equiv.) and degassed water (0.05 mL) was added sequentially at the same temperature. The vial was sealed and stirred for 8 h while warming slowly to room temperature. The reaction mixture was then diluted with water and extracted with ethyl acetate (3 x 5 mL). The combined organic layers were washed with water, dried over Na_2SO_4, filtered, and concentrated in vacuo. The crude product was purified by preparative TLC using hexane/ethyl acetate (3:1) as the eluent to afford the keto alcohol **17** (9 mg) as colorless oil and 3.5 mg of alcohol **16** was also recovered. NMR yield: 45% (using 4-Nitrobenzaldehyde as internal standard), isolated yield: 36% (42% brsm)

^{1}**H NMR** (500 MHz, Chloroform-*d*) δ (ppm): 7.32 (d, *J* = 2.3 Hz, 1H, 6-C*H*), 7.10 – 7.05 (m, 2H, 14, 10-C*H*), 6.85 (d, *J* = 8.4 Hz, 1H, 9-C*H*), 6.12 – 6.05 (m, 1H, 12-C*H*), 5.76 (d, *J* = 17.7 Hz, 1H, 15-CH_aH$_b$), 5.44 (d, *J* = 17.2 Hz, 1H, 13-CH_aH$_b$), 5.31 – 2.29 (m, 2H, 13-CH$_a$$H_b$,15-CH$_a$$H_b$), 4.57 (d, *J* = 5.1 Hz, 2H, 11-CH_2), 3.85 – 3.82 (m, 2H, 1-CH_2), 3.68 (s, 2H, 4-CH_2), 2.73 (t, *J* = 5.4 Hz, 2H, 2-CH_2), 2.37 (brs, 1H, O*H*).

^{13}**C NMR** (126 MHz, Chloroform-*d*) δ (ppm): 209.5 (3-*C*), 155.0 (8-*C*), 133.2 (12-*C*), 131.3 (14-*C*), 129.6 (10-*C*), 127.5 (6-*C*), 127.4 (7-*C*), 125.9 (5-*C*), 117.4 (13-*C*), 114.8 (15-*C*), 112.7 (9-*C*), 69.2 (11-*C*), 57.9 (1-*C*), 49.8 (4-*C*), 43.5 (2-*C*).

TLC: R_f = 0.3 (Hexane:EtOAc 3:2)

HRMS (ESI): m/z calcd for $C_{15}H_{18}O_3Na$ $[M+Na]^+$: 269.1154; found: 269.1147.

Compound 19

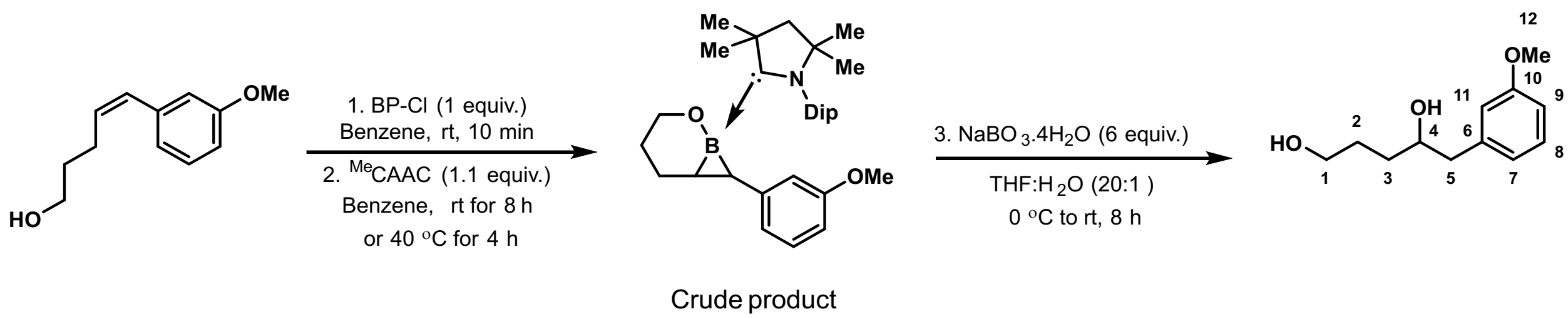

In a nitrogen-filled glovebox, a 4 mL screw-capped glass vial was charged with alcohol **18** (20 mg, 0.104 mmol, 1 equiv.) and dissolved in 0.5 mL of anhydrous benzene. To this solution, borepin **4** (18 mg, 0.104 mmol, 1 equiv.) in benzene (0.5 mL) was added dropwise at room temperature. After 10 minutes, the volatile components were removed under reduced pressure. (**borepin formation**)

The crude mixture was then dissolved in 0.5 mL of anhydrous benzene, and a solution of ^{Me}CAAC (32.7 mg, 0.114 mmol, 1.1 equiv.) in benzene (0.5 mL) was added at room temperature. The reaction mixture was stirred at room temperature for 8 h or at 40 °C for 4 h. Then, solvent was removed was removed under reduced pressure and vial was taken outside of glovebox. (**borirane formation**)

Next, the residue was dissolved in degassed THF (1 mL) and cooled to ice bath. Then, $NaBO_3 \cdot 4H_2O$ (96 mg, 0.62 mmol, 6 equiv.) and degassed water (0.05 mL) was added sequentially at the same temperature. The vial was sealed under nitrogen atmosphere and stirred for 8 h while warming slowly to room temperature. The reaction mixture was then diluted with water and extracted with ethyl acetate (3 x 5 mL). The combined organic layers were washed with water, dried over Na_2SO_4, filtered, and concentrated in vacuo. The crude product was purified by preparative TLC using hexane/ethyl acetate (2:1) as the eluent to afford the diol **19** (14 mg) as colorless oil and 2 mg of alcohol **18** was also recovered. Isolated yield: 64% (71% brsm)

^{1}H NMR (500 MHz, Chloroform-*d*) δ (ppm): 7.25 (t, *J* = 7.8 Hz, 1H, 8-C*H*), 6.83 – 6.78 (m, 3H, 7, 9, 11-C*H*), 3.90 – 3.87 (m, 1H, 4-C*H*), 3.82 (s, 3H, 12-CH_3), 3.73 – 3.64 (m, 2H, 1-CH_2), 2.81 (dd, *J* = 13.5, 4.6 Hz, 1H, 5-CH_aH_b), 2.70 (dd, *J* = 13.5, 8.4 Hz, 1H, 5-CH_aH_b), 2.49 (brs, 1H, O*H*), 1.77 – 1.71 (m, 3H, 2, 3-CH_2), 1.61 – 1.52 (m, 1H, 2, 3-CH_2). The data are consistent with the reported literature values.[21]

^{13}C {^{1}H} NMR (126 MHz, Chloroform-*d*) δ (ppm): 159.8 (10-*C*), 140.0 (6-*C*), 129.6 (8-*C*), 121.7, 115.1, 111.8 (7, 11, 9-*C*), 72.6 (4-*C*), 62.9 (1-*C*), 55.1 (12-*C*), 44.2 (5-*C*), 33.8, 29.2 (2, 3-*C*). The data are consistent with the reported literature values.[21]

Compound 21

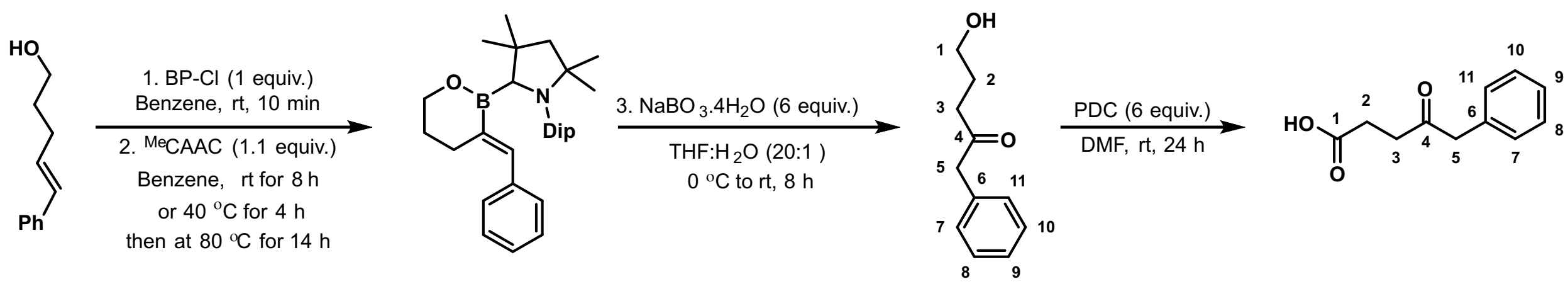

20 Crude product 20a 21

In a nitrogen-filled glovebox, a 4 mL screw-capped glass vial was charged with alcohol **20** (16.2 mg, 0.1 mmol, 1 equiv.) and dissolved in 0.5 mL of anhydrous benzene. To this solution, borepin **4** (17.4 mg, 0.1 mmol, 1 equiv.) in benzene (0.5 mL) was added dropwise at room temperature. After 10 minutes, the volatile components were removed under reduced pressure. (**borepin formation**)

The crude mixture was then dissolved in 0.5 mL of anhydrous benzene, and a solution of ^{Me}CAAC (32.7 mg, 0.114 mmol, 1.1 equiv.) in benzene (0.5 mL) was added at room temperature. The reaction mixture was stirred at room temperature for 8 h or at 40 °C for 4 h. (**borirane formation**). Then, reaction was shifted to 80 ºC oil bath and kept at this temperature for 14 h. (**vinyl borane formation**)

Next, the residue was dissolved in degassed THF (1 mL) and cooled to ice bath. Then, $NaBO_3 \cdot 4H_2O$ (96 mg, 0.6 mmol, 6 equiv.) and degassed water (0.05 mL) was added sequentially at the same temperature. The vial was sealed under nitrogen atmosphere and stirred for 8 h while warming slowly to room temperature. The reaction mixture was then diluted with water and extracted with ethyl acetate (3 x 5 mL). The combined organic layers were washed with water, dried over Na_2SO_4, filtered, and concentrated in vacuo. The crude product was purified by preparative TLC using hexane/ethyl acetate (3:1) as the eluent to afford the keto alcohol **20a** (11 mg) and 3.5 mg of alcohol **20a** was also recovered. Isolated yield: 62% (77% brsm) obtained as colorless liquid and mixtures of ketone and hemiketal (4:1).

^{1}**H NMR** (400 MHz, Chloroform-*d*) δ (ppm): 7.37 – 7.33 (m, 3H, 7-11 C*H*), 7.23 – 7.21 (m, 2H, 7-11 C*H*), 3.73 (s, 2H, 5-CH_2), 3.61 (t, *J* = 6.1 Hz, 2H, 1-CH_2), 2.61 (t, *J* = 6.9 Hz, 2H, 3-CH_2), 1.86 – 1.79 (m, 2H, 2-CH_2), 1.74 (brs, 1H, O*H*). The data are consistent with the reported literature values.[22]

^{13}C {^{1}H} NMR (101 MHz, Chloroform-*d*) δ (ppm): 208.9 (4-*C*), 134.1 (6-*C*), 129.4 (10 or 11-*C*), 128.7 (10 or 11-*C*), 127.1 (9-*C*), 62.1 (1-*C*), 50.2 (5-*C*), 38.7 (3-*C*), 26.4 (2-*C*). The data are consistent with the reported literature values.[22]

A solution of keto alcohol (18 mg, 0.10 mmol, 1 equiv.) and PDC (237 mg, 0.6 mmol, 6 equiv.) in DMF (1 mL) was stirred at room temperature under inert atmosphere for 24 h. The reaction mixture was diluted with Et_2O (2 mL) and filtered through a pad of Celite that was washed thoroughly with additional Et_2O. The filtrate was concentrated in vacuo, and the residue was purified by preparative TLC using hexane/ethyl acetate (1:1) as the eluent to afford the keto acid **21** (13.5 mg, 70%) as colorless liquid.

^{1}H NMR (500 MHz, Chloroform-*d*) δ (ppm): 7.35 (t, J = 7.4 Hz, 2H, 10-C*H*), 7.30 (d, J = 7.1 Hz, 1H, 9-C*H*), 7.23 (d, J = 7.4 Hz, 2H, 11-C*H*), 3.76 (s, 2H, 5-CH_2), 2.77 (t, J = 6.5 Hz, 2H, 3-CH_2), 2.62 (t, J = 6.5 Hz, 2H, 2-CH_2). The data are consistent with the reported literature values.[23]

^{13}C {^{1}H} NMR (126 MHz, Chloroform-*d*) δ (ppm): 206.3 (4-*C*), 178.0 (1-*C*), 133.9 (6-*C*), 129.4 (10 or 11-*C*), 128.8 (10 or 11-*C*), 127.1 (9-*C*), 50.0 (5-*C*), 36.1 (3-*C*), 27.7 (2-*C*). The data are consistent with the reported literature values.[23]

2. Additional Data

2.1. X-ray data collection and structure refinement

Single crystals of **3**, **3a**, **3b**, **6a**, **6b**, **7a** (from 6a or 6b), **7e**, **7f**, **14**, **Int-3'** and **4-DMAP** suitable for X-ray diffraction were coated in Paratone oil (Hampton Research, CA, USA), mounted on a MiTeGen loop, and flash-frozen in liquid nitrogen.

Diffraction data for **3**, **3b**, **6b, 7a** (from 6b)**, 7f** , and **14** were recorded on a Rigaku Synergy R system equipped with a HyPix-Arc 150 detector. Data were measured using Cu-Kα radiation (1.54184 Å) and processed with CrysAlis^{PRO}. (1.171.42.94a, Rigaku OD, 2023). Data for structures **3a**, **6a, 7a,7e, Int-3'** and **4-DMAP** were collected on a Rigaku Synergy-R diffractometer with a HyPix ARC 100º detector, MoKα (λ=0.71073Å) and processed with CrysAlis^{PRO}. (1.171.42.94a, Rigaku OD, 2023). Data collection was performed under LN at 100 K. The structures were solved by direct methods using SHELXT[24]. All non-hydrogen atoms were further refined by SHELXL[25] with anisotropic displacement coefficients. Hydrogen atoms were assigned isotropic displacement coefficients, U(H) = 1.2U(C) or 1.5U (C-methyl), and their coordinates were allowed to ride on their respective carbons. Refinement was carried out with the OLEX-2[26] GUI. Crystallographic data and refinement parameters are summarized in the tables below.

Table 1. X-ray crystallographic data.

Complex	3a	3b	6a	6b
**CCDC No.**	**2551991**	**2551988**	**2551987**	**2551992**
**Formula**	$C_{29}H_{40}BN$	$C_{26}H_{36}BN$	$C_{21}H_{18}BF_3O$	$C_{21}H_{18}BF_3O$
**Formula weight**	413.43	373.37	354.16	354.16
**Crystal system**	monoclinic	orthorhombic	monoclinic	monoclinic
**Space group**	$P\,2_1$	*P bca*	$P\,2_1/c$	$P\,2_1$
**Crystal size**	0.23 x0.17 x0.16	0.61 x0.35 x0.22	0.23 x0.12 x0.04	0.52 x0.20 x0.12
**Crystal color and shape**	Yellow block	Colorless prism	Colorless plate	Colorless irregular
**Temperature (K)**	100	100	100	100
**wavelength (Å)**	0.71073	1.54178	0.71073	1.54178
**a, (Å)**	23.3420(4)	10.4272(1)	19.7003(5)	6.1410(3)
**b, (Å)**	9.9510(1)	14.2390(1)	11.2762(2)	37.1174(16)
**c, (Å)**	23.9990(5)	29.6488(3)	7.8546(2)	7.5338(3)
**α, (°)**	90	90	90	90
**β, (°)**	119.075(3)	90	97.022(2)	91.296(4)
**γ, (°)**	90	90	90	90
**Volume ($Å^3$)**	4871.94(19)	4402.03(6)	1731.77(7)	1716.80(13)
**Z**	8	8	4	4
**ρ_{calcd},(g cm^{-1})**	1.127	1.127	1.358	1.370
**μ, mm^{-1}**	0.063	0.468	0.103	0.871
**F(000)**	1808	1632	736	736
**Limiting indices**	$-32 \le h \le 33$<br>$-14 \le k \le 14$<br>$-34 \le l \le 34$	$-12 \le h \le 13$<br>$-13 \le k \le 17$<br>$-36 \le l \le 37$	$-25 \le h \le 25$<br>$-16 \le k \le 14$<br>$-11 \le l \le 9$	$-7 \le h \le 7$<br>$-44 \le k \le 44$<br>$-9 \le l \le 9$
**Reflections collected**	206720(28675)	61039(4498)	32912(4979)	25957(6268)
**R_{int}**	0.0522	0.0254	0.0390	0.0597
**Theta range for data**	2.579-30.914	5.186-75.083	2.758-31.570	4.766-68.245
**Completeness to θ (%)**	95.5	99.3	85.7	99.7
**data/restraints/parameters**	28675/1/1359	4498/0/260	4979/0/235	6268/1/469
**goodness-of-fit on F^2**	1.028	1.032	1.024	1.087
**Final R_1 and wR_2 indices [*I***	0.0497,0.1193	0.0408,0.1057	0.0435,0.1039	0.0558,0.1464
**R_1 and wR_2 indices (all**	0.0623,0.1257	0.0428,0.1076	0.0680,0.1139	0.0771,0.1736
**Largest diff peak and hole**	0.325, -0.171	0.255, -0.212	0.346, -0.234	0.244, -0.241
**Flack Parameter**	-0.4(9)			-0.11(11)

Continue …

Complex	**7a** (from 6b)	**7a** (from 6a)	**7e**	**7f**
**CCDC No.**	**2551989**	**2551985**	**2551986**	**2551990**
**Formula**	$C_{31} H_{41} B F_3 N O$	$C_{31} H_{41} B F_3 N O$	$C_{32} H_{43} B F_3 N O$	$C_{33} H_{45} B F_3 N O$
**Formula weight**	511.46	373.37	525.48	539.51
**Crystal system**	triclinic	triclinic	monoclinic	monoclinic
**Space group**	*P -1*	*P -1*	$P\ 2_1/n$	$P\ 2_1/c$
**Crystal size**	0.28 x0.21 x0.10	0.22 x0.13 x0.07	0.44 x0.37 x0.25	0.07 x 0.10 x0.14
**Crystal color and shape**	Yellow irregular	Yellow block	Yellow block	Yellow irregular
**Temperature (K)**	100	100	100	100
**wavelength (Å)**	1.54178	0.71073	0.71073	1.54178
**a, (Å)**	9.6285(1)	9.6329(2)	10.5152(2)	9.38323(11)
**b, (Å)**	10.2648(1)	10.2695(2)	9.3472(1)	9.97177(11)
**c, (Å)**	28.3885(4)	28.4286(7)	29.6217(5)	31.6139(3)
**α, (°)**	94.8400(10)	94.763(2)	90	90
**β, (°)**	90.7540(10)	90.654(2)	91.439(2)	92.5635(10)
**γ, (°)**	90.9040(10)	90.687(2)	90	90
**Volume (Å^3)**	2795.12(6)	2802.17(11)	2910.53(8)	2955.07(6)
**Z**	4	4	4	4
**ρ_{calcd},(g cm^{-1})**	1.215	1.212	1.199	1.213
**μ, mm^{-1}**	0.691	0.085	0.084	0.678
**F(000)**	1096	1096	1128	1160
**Limiting indices**	$-11 \leq h \leq 11$<br>$-12 \leq k \leq 12$<br>$-32 \leq l \leq 34$	$-11 \leq h \leq 11$<br>$-12 \leq k \leq 12$<br>$0 \leq l \leq 34$	$-15 \leq h \leq 15$<br>$-13 \leq k \leq 14$<br>$-42 \leq l \leq 45$	$-11 \leq h \leq 11$<br>$-12 \leq k \leq 12$<br>$-39 \leq l \leq 35$
**Reflections collected**	58537(10222)	10240(10240)	73257(10215)	77940(6016)
**R_{int}**	0.0287	?*	0.0456	0.0309
**Theta range for data**	4.323-68.248	2.542-25.350	2.039-33.186	4.650-74.989
**Completeness to θ (%)**	99.8	99.7	91.7	98.6
**data/restraints/parameters**	10222/0/696	10240/0/697	10215/0/351	6016/0/361
**goodness-of-fit on F^2**	1.068	1.075	1.038	0.959
**Final R_1 and wR_2 indices [*I***	0.0605,0.1754	0.0597,0.1752	0.0552,0.1484	0.0423,0.1026
**R_1 and wR_2 indices (all**	0.0664,0.1795	0.0713,0.1834	0.0754,0.1641	0.0489,0.1070
**Largest diff peak and hole**	0.448, -0.399	0.538, -0.337	0.546, -0.416	0.251, -0.259
**Flack Parameter**				

*Twin

Continue …

Complex	14	3	4-DMAP	Int-3`
**CCDC No.**	**2559061**	**2570687**	**2570688**	**2570689**
**Formula**	$C_{34}H_{48}BNO$	$C_{16}H_{13}B$	$C_{42}H_{47}B_2Cl_5N_6$	$C_{33}H_{43}BClN$
**Formula weight**	497.54	216.07	834.72	499.94
**Crystal system**	monoclinic	monoclinic	monoclinic	monoclinic
**Space group**	$P\,2_1/c$	$P\,2_1/c$	$P\,2_1/c$	$P\,2_1/n$
**Crystal size**	0.23 x0.14 x0.11	0.13 x0.06 x0.02	0.52 x0.38 x0.04	0.62 x0.39 x0.14
**Crystal color and shape**	Yellow block	Colorless plate	Colorless prism	Orange chunk
**Temperature (K)**	100	100	100	100
**wavelength (Å)**	1.54178	1.54178	0.71073	0.71073
**a, (Å)**	19.7983(2)	5.8144(2)	10.2985(3)	10.5806(2)
**b, (Å)**	13.0228(1)	7.8342(2)	29.1124(8)	18.1177(2)
**c, (Å)**	11.7125(1)	25.2717(7)	14.6202(3)	14.8033(2)
**α, (°)**	90	90	90	90
**β, (°)**	95.677(1)	96.037(3)	106.941(3)	105.423(2)
**γ, (°)**	90	90	90	90
**Volume (Å^3)**	3005.01(4)	1144.77(6)	4193.1(2)	2735.55(8)
**Z**	4	4	4	4
**ρ_{calcd},(g cm^{-1})**	1.100	1.254	1.322	1.214
**μ, mm^{-1}**	0.480	0.520	0.385	0.162
**F(000)**	1088	456	1744	1080
**Limiting indices**	$-24 \le h \le 24$<br>$-16 \le k \le 14$<br>$-14 \le l \le 14$	**$-6 \le h \le 7$<br>$-9 \le k \le 9$<br>$-31 \le l \le 31$**	**$-13 \le h \le 13$<br>$-36 \le k \le 33$<br>$-18 \le l \le 17$**	**$-16 \le h \le 15$<br>$-27 \le k \le 26$<br>$-20 \le l \le 23$**
**Reflections collected**	101061(6133)	8188(8188)	47141(8893)	70172(9979)
**R_{int}**	0.0356	?*	0.0314	0.0421
**Theta range for data**	4.069-74.989	3.517-75.174	2.496-26.732	2.663-33.873
**Completeness to θ (%)**	99.3	98.0	99.9	90.3
**data/restraints/parameters**	6133/0/364	8188/0/202	8893/0/502	9979/0/331
**goodness-of-fit on F^2**	1.041	1.122	1.041	1.054
**Final R_1 and wR_2 indices [I**	0.0403,0.1023	0.0666,0.1897	0.0387,0.0871	0.0418,0.1150
**R_1 and wR_2 indices (all**	0.0446,0.1057	0.0753,0.2004	0.0522,0.0920	0.0524,0.1226
**Largest diff peak and hole**	0.268, -0.224	0.231, -0.200	0.303, -0.295	0.519,-0.219
**Flack Parameter**				

2.2. Low-temperature NMR experiment

<u>^{Me}CAAC coordination intermediate</u>: Low-temperature NMR experiments were conducted to validate the proposed reaction mechanism and characterize intermediates involved in the transformation. It is proposed that ^{Me}CAAC initially coordinates to the boron center of the alkoxy borepin **6**, driving the formation of a naphthalene borirane intermediate. At room temperature, the latter intermediate facilitates the extrusion of naphthalene and the generation of a borylene, which subsequently reacts with the tethered alkene to give the fused borirane as the final product **7**.

To investigate the initial coordination step, a solution of ^{Me}CAAC (20.2 mg, 0.07 mmol, 1.0 equiv.) in toluene-d_8 (0.2 mL) was prepared in an NMR tube equipped with a rubber septum under an N_2 atmosphere and cooled to −70 °C. A solution of alkoxy borepin **6a** (25 mg, 0.07 mmol, 1.0 equiv.) in toluene-d_8 (0.2 mL) was then added via syringe at −70 °C. The resulting sample was analyzed by NMR spectroscopy at −10 °C. In the ^{1}H NMR spectrum, the α-protons of the **Int-1** appeared at 6.15 ppm (**A**), upfield relative to the free alkoxy borepin **6a** (6.75 ppm), consistent with a reduced ring current due to coordination of the boron center with ^{Me}CAAC (**Figure S1**). In addition, the expected doublet corresponding to the β-protons (**B**) of **Int-1** overlaps with the aromatic region (7.30–7.10 ppm). The ^{11}B NMR spectrum shows a significant shift in the boron resonance from 38 ppm to a sharp signal at −2 ppm, indicating quaternization of the boron center in the initial step of the reaction (**Figure S2**). This observation supports the first-step coordination of ^{Me}CAAC with the alkoxy borepin **6a**. Due to the low temperature, complete coordination was not achieved, as a portion of the ^{Me}CAAC remained frozen in the NMR tube. As a result, unreacted starting material **6a** was also observed in the NMR spectrum.

Upon warming the NMR tube to room temperature for 5 min and reanalyzing the sample, the resonance corresponding to the coordinated species disappeared, and a new signal at −12 ppm appeared in the ^{11}B NMR spectrum, supporting the formation of a naphthalene borirane **Int-3** (**Figure S2-iii**).

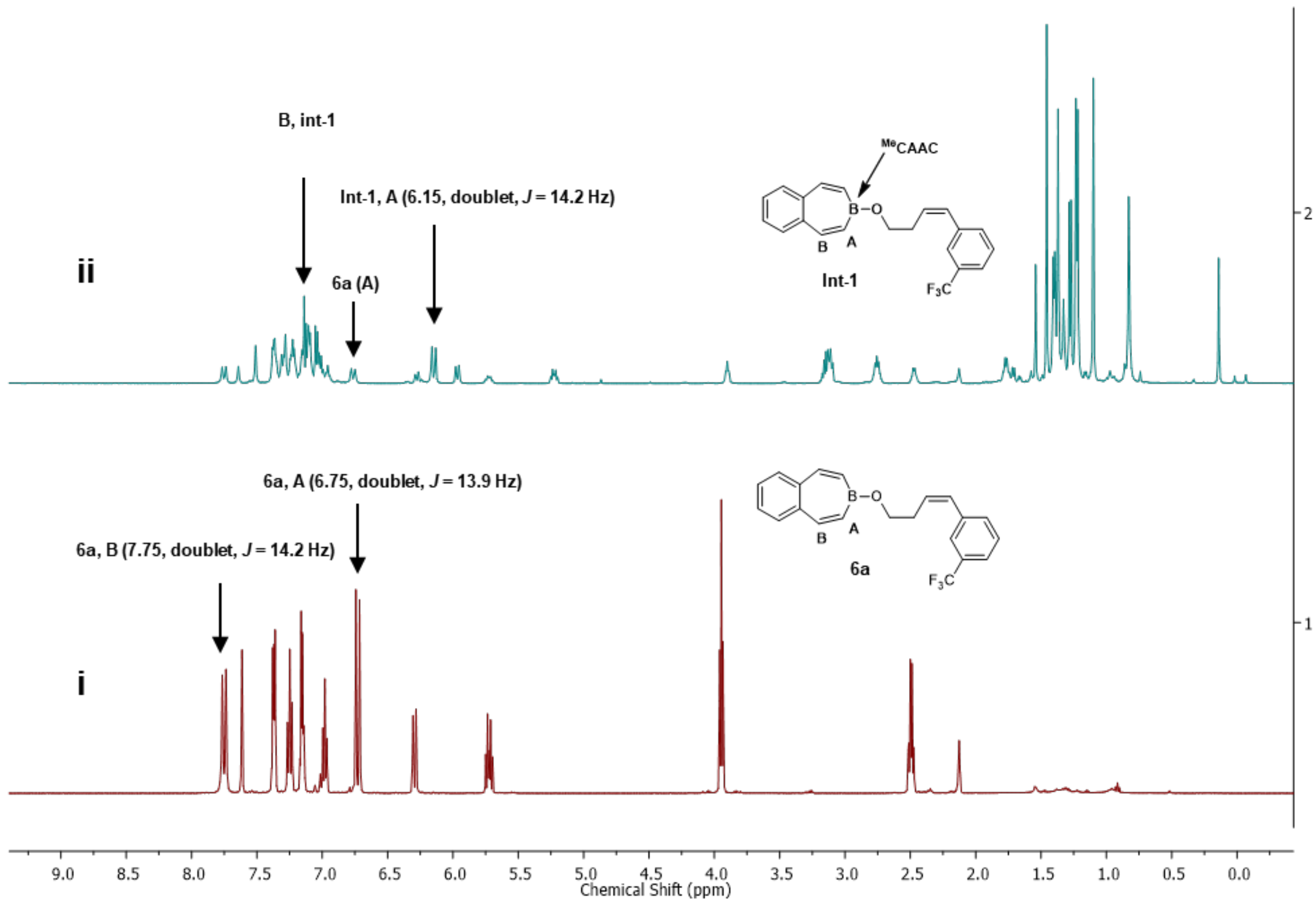

Figure S1: Stacked ^{1}H NMR (500 MHz, 263 K, Toluene-d_8) spectra highlighting the variation in characteristic borepin proton signal upon coordination to ^{Me}CAAC.

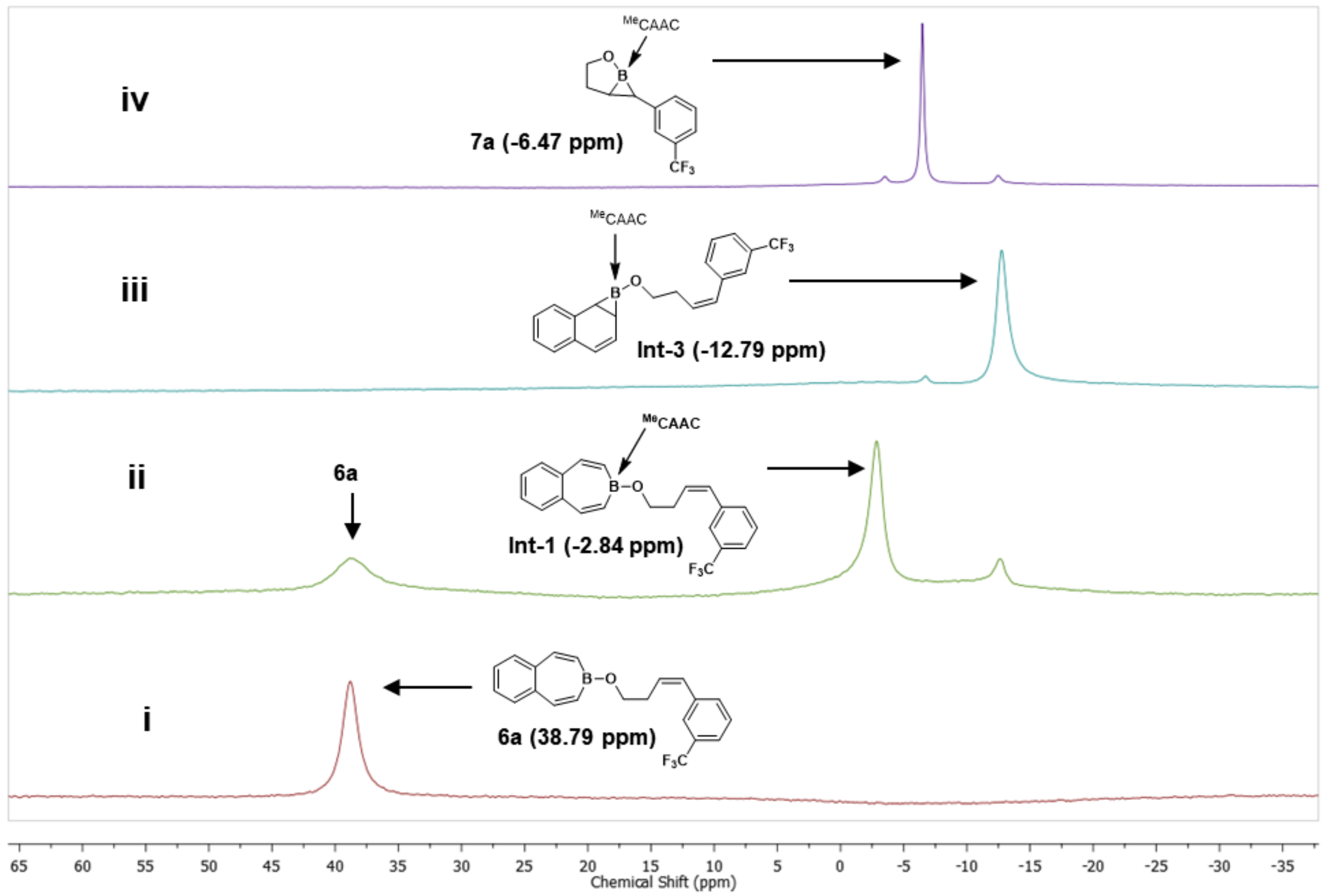

Figure S2: Stacked ^{11}B NMR (500 MHz, toluene-d_8) spectra illustrating the progression from the starting material through intermediates to the final product.

Borirane-naphthalene intermediate: To characterize the structure of **Int-3**, ^{1}H NMR and 2D-COSY NMR were performed at −10 °C. At this temperature, the intermediate was found to be relatively stable and did not transform into the final product (**Figure S3**). Two characteristic sets of signals support this assignment. First, the protons corresponding to 1-C*H* and 2-C*H* of the endocyclic double bond resonate at 6.35 ppm as a doublet and at 6.23–6.21 ppm as a multiplet, respectively. In addition, the characteristic borirane protons 3-C*H* and 4-C*H* appear as a multiplet at 1.90–1.79 ppm with an integration corresponding to two protons (**Figure S3**). Further support for this structural assignment was obtained from 2D-COSY analysis, which revealed a strong correlation between the endocyclic olefinic protons 1-C*H* and 2-C*H*. In addition, 2-C*H* also exhibited a correlation with the borirane proton 3-C*H* in the 2D COSY spectrum (**Figures S4** and **S5**). The signals corresponding to the aromatic protons of the naphthalene backbone and the aryl substituents (^{Me}CAAC and CF_3-aryl) were also consistent with the proposed structure. The methylene (11-CH_2 and 12-CH_2) and methine protons of the isopropyl group of ^{Me}CAAC appear in the 3.46–2.12 ppm region. The olefinic protons are observed as a doublet at 6.25 ppm (14-C*H*) and a doublet of triplets at 5.74 ppm (13-C*H*). All other resonances attributed to the ^{Me}CAAC fragment are consistent with the proposed structure.

In the 2D COSY spectrum, proton 13-CH shows correlations with the adjacent olefinic proton 14-C*H* and methylene proton 12-CH_2. In addition, the methylene protons 11-CH_2 and 12-CH_2 exhibit a clear correlation, providing strong support for the proposed structure of the intermediate. The ^{1}H NMR data and 2D COSY analysis for **Int-3** are illustrated in **Figures S3-S5**.

^{1}H NMR (500 MHz, 263 K, Toluene-d_8) δ (ppm) of **Int 1**: 7.55 (d, J = 7.6 Hz, 1H), 7.51 (s, 1H, 16-C*H*), 7.26 (d, J = 7.6 Hz, 1H), 7.15 (t, J = 7.7 Hz, 1H), 7.07 (d, J = 7.0 Hz, 1H), 7.04 – 6.95 (m, 5H), 6.88 (d, J = 6.8 Hz, 1H), **6.35 (d, J = 9.2 Hz, 1H, 1-C*H*), 6.25 (d, J = 11.8 Hz, 1H, 14-C*H*), 6.23 – 6.21 (m, 1H, 2-C*H*), 5.74 (dt, J = 11.7, 7.6 Hz, 1H, 13-C*H*),** 3.46 (dd, J = 13.5, 7.4 Hz, 1H, 11-CH_aH$_b$), 3.06 (brs, 1H, 11-CH$_a$$H_b$), 2.83 (sept, J = 6.5 Hz, 1H, *i*Pr-C*H*), 2.75 (sept, J = 6.4 Hz, 1H, *i*Pr-C*H*), 2.35 – 2.28 (m, 1H, 12-CH_aH$_b$), 2.16 – 2.12 (m, 1H, 12-CH$_a$$H_b$), **1.90 – 1.79 (m, 2H, 3,4-C*H*),** 1.72 (s, 3H, C$_q$CH_3^{CAAC}), 1.70 (s, 3H, C$_q$CH_3^{CAAC}), 1.57 – 1.53 (m, 2H, CH_2^{CAAC}), 1.39 (d, J = 6.2 Hz, 3H, *i*Pr-CH_3), 1.21 (d, J = 6.2 Hz, 3H, *i*Pr-CH_3), 1.15 (d, J = 6.6 Hz, 3H, *i*Pr-CH_3), 1.11 (d, J = 6.5 Hz, 3H, *i*Pr-CH_3), 0.97 (s, 3H, C$_q$CH_3^{CAAC}), 0.85 (s, 3H, C$_q$CH_3^{CAAC}).

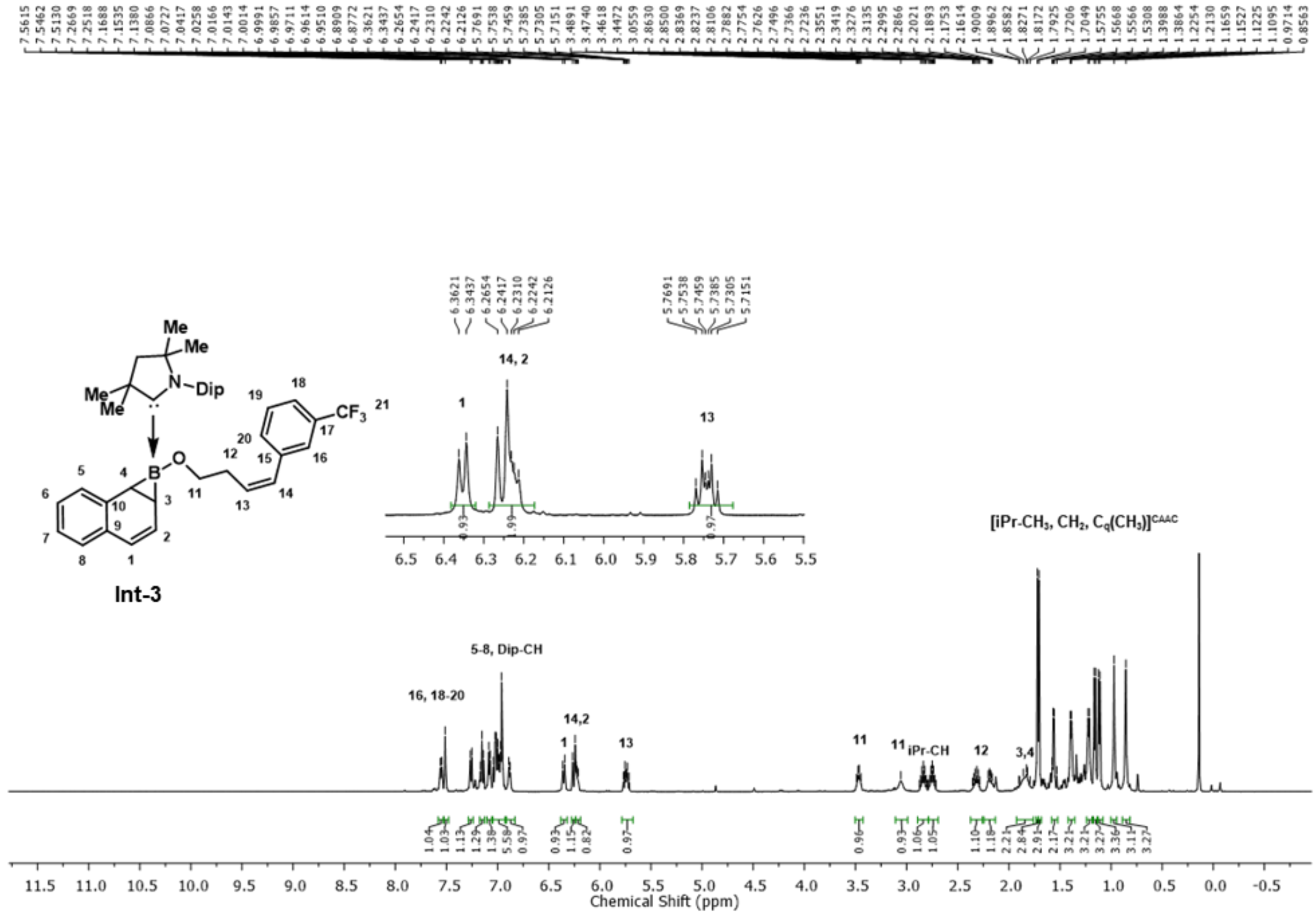

Figure S3: ^{1}H NMR (Toluene-d_8, 263 K, 500 MHz) of **int-3**.

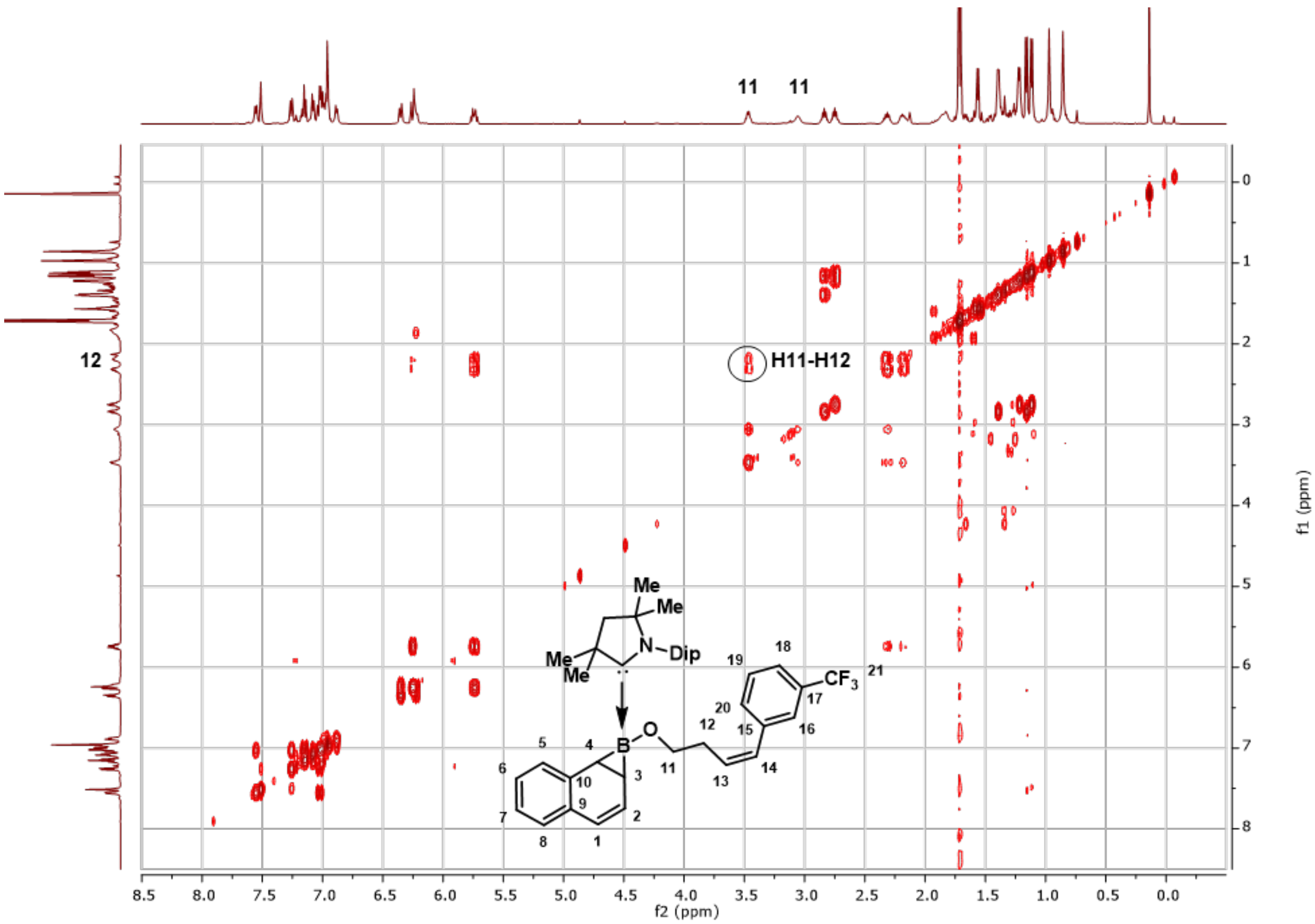

Figure S4: Full spectrum of ^{1}H -^{1}H 2D COSY NMR (Toluene-d_8, 263 K, 500 MHz) of **int-3**.

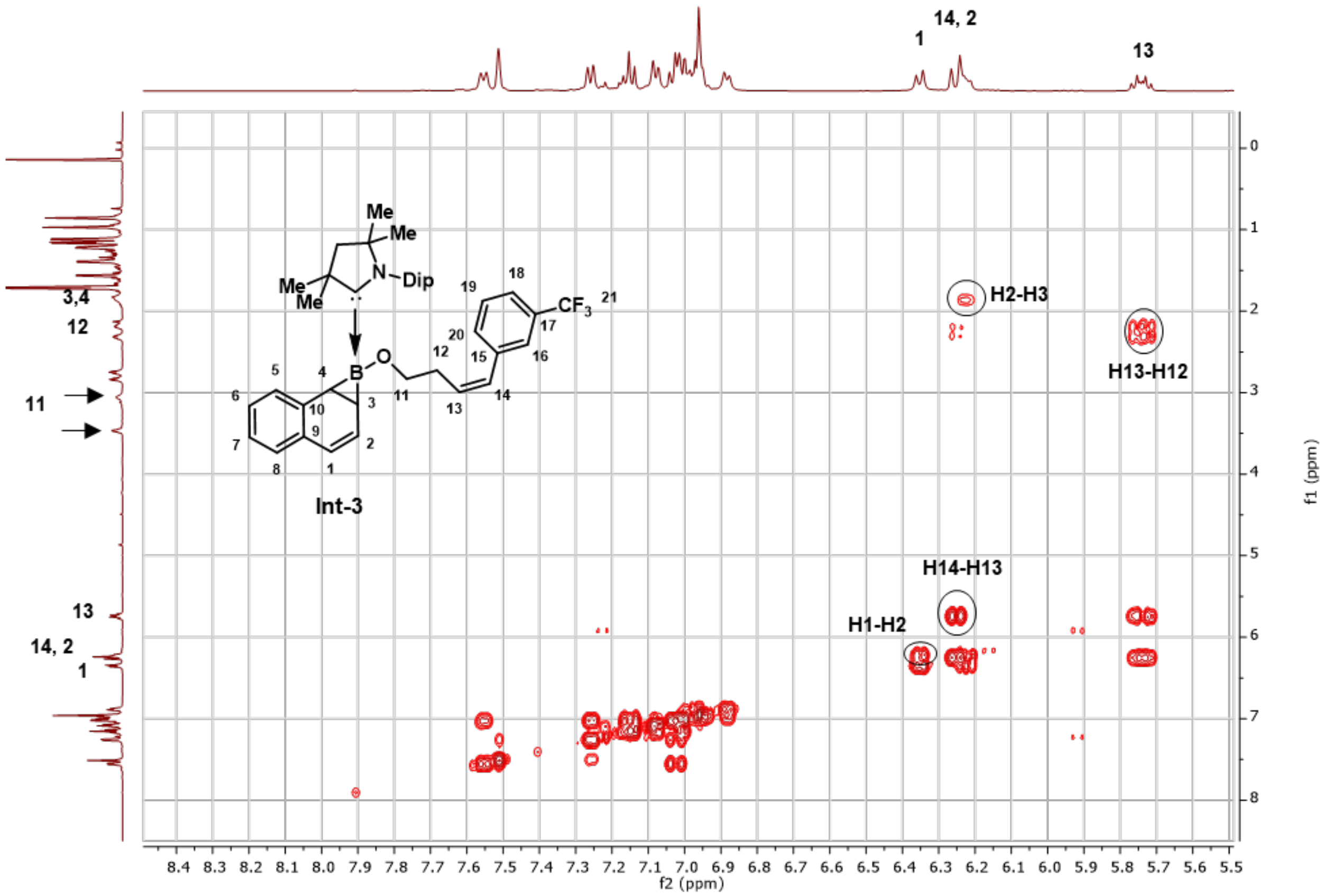

Figure S5: Zoomed in spectra of ^{1}H -^{1}H 2D COSY NMR (Toluene-d_8, 263 K, 500 MHz) of **int-3**.

2.3. Stability test of compounds 3 and 6g

The compound was stored on the bench in an open vial exposed to air. ^{1}H and ^{11}B NMR spectra were recorded at different time intervals over several days to assess their stability. Compound **3** was found to be stable under these conditions, showing only minor decomposition over the observed period (**Figures S6** and **S7**). However, compound **6g** remained unchanged and showed no decomposition (**Figures S8** and **S9**).

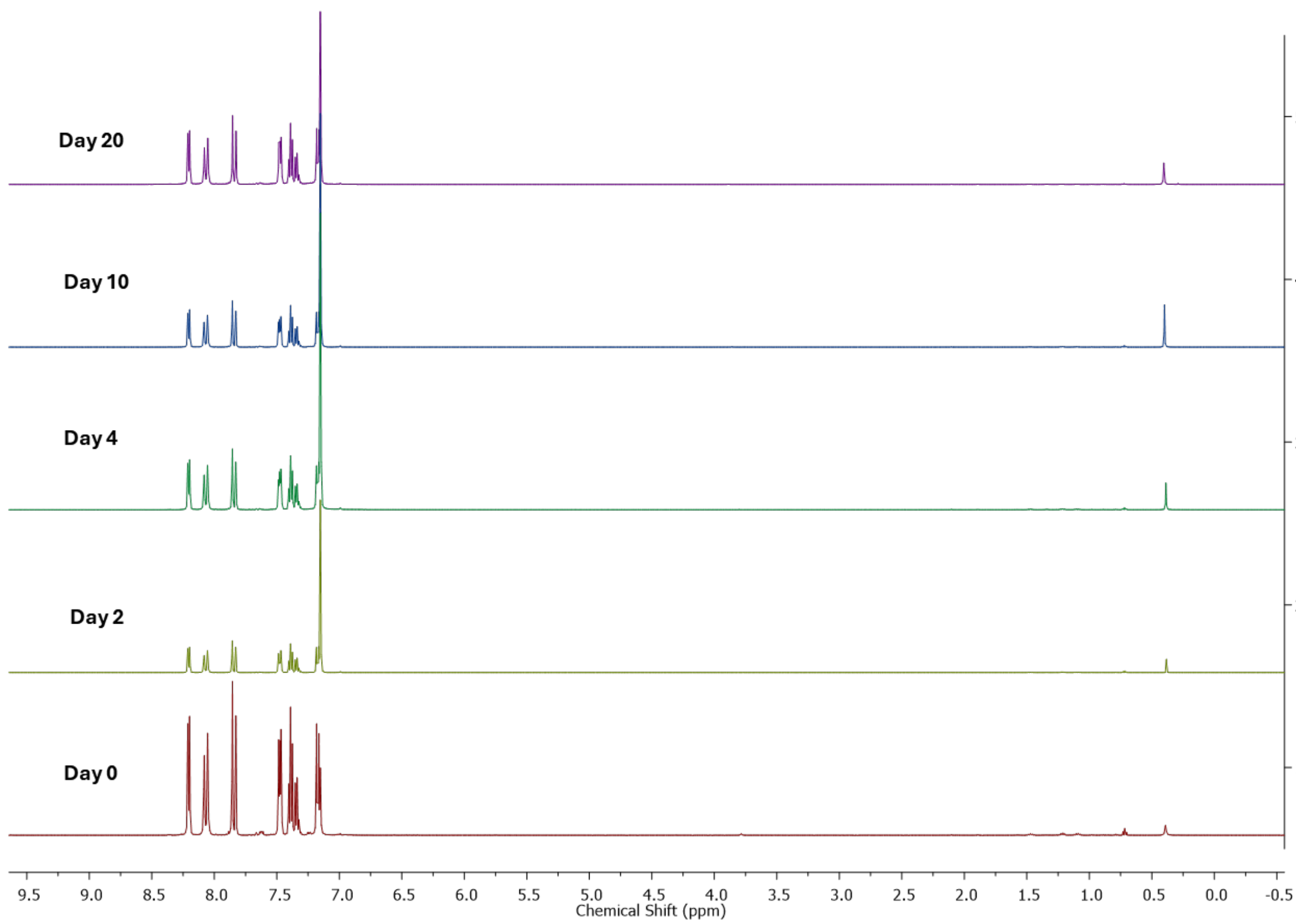

Figure S6: Stacked ^{1}H NMR spectra (500 MHz, Benzene-d_6) of compound **3** recorded over several days.

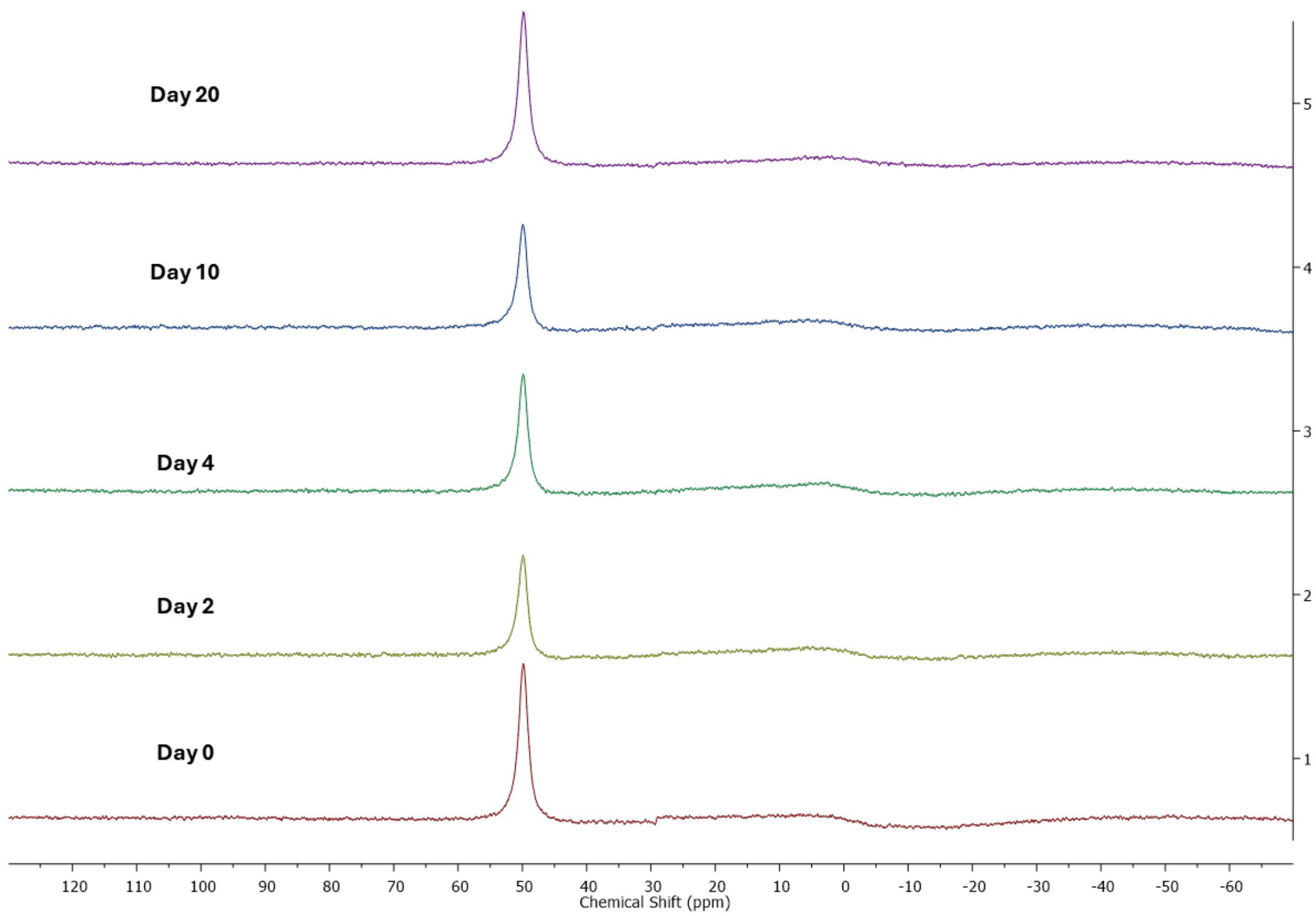

Figure S7: Stacked ^{11}B NMR spectra (160 MHz, Benzene-d_6) of compound **3** recorded over several days.

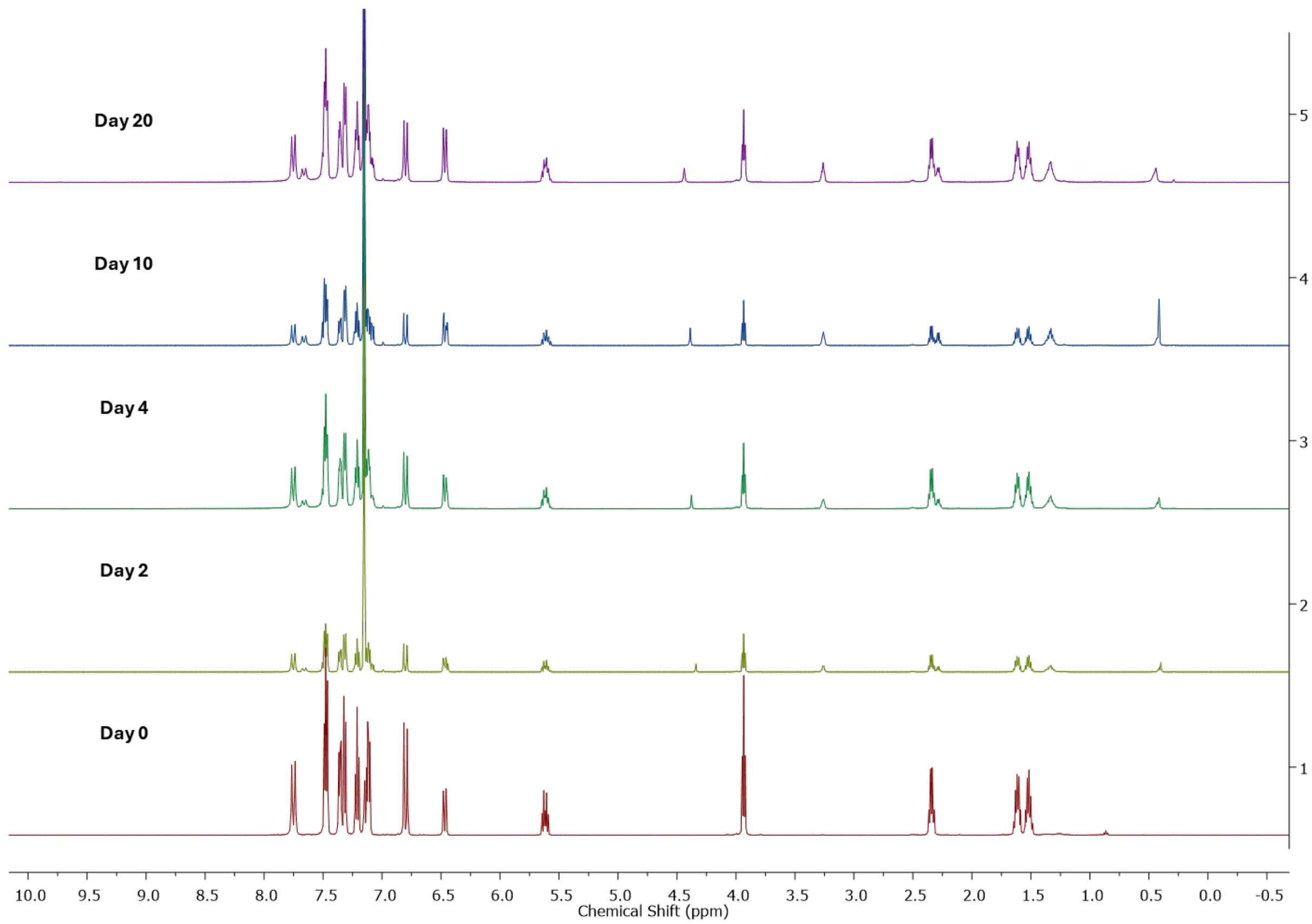

Figure S8: Stacked ^{1}H NMR spectra (500 MHz, Benzene-d_6) of compound **6g** recorded over several days.

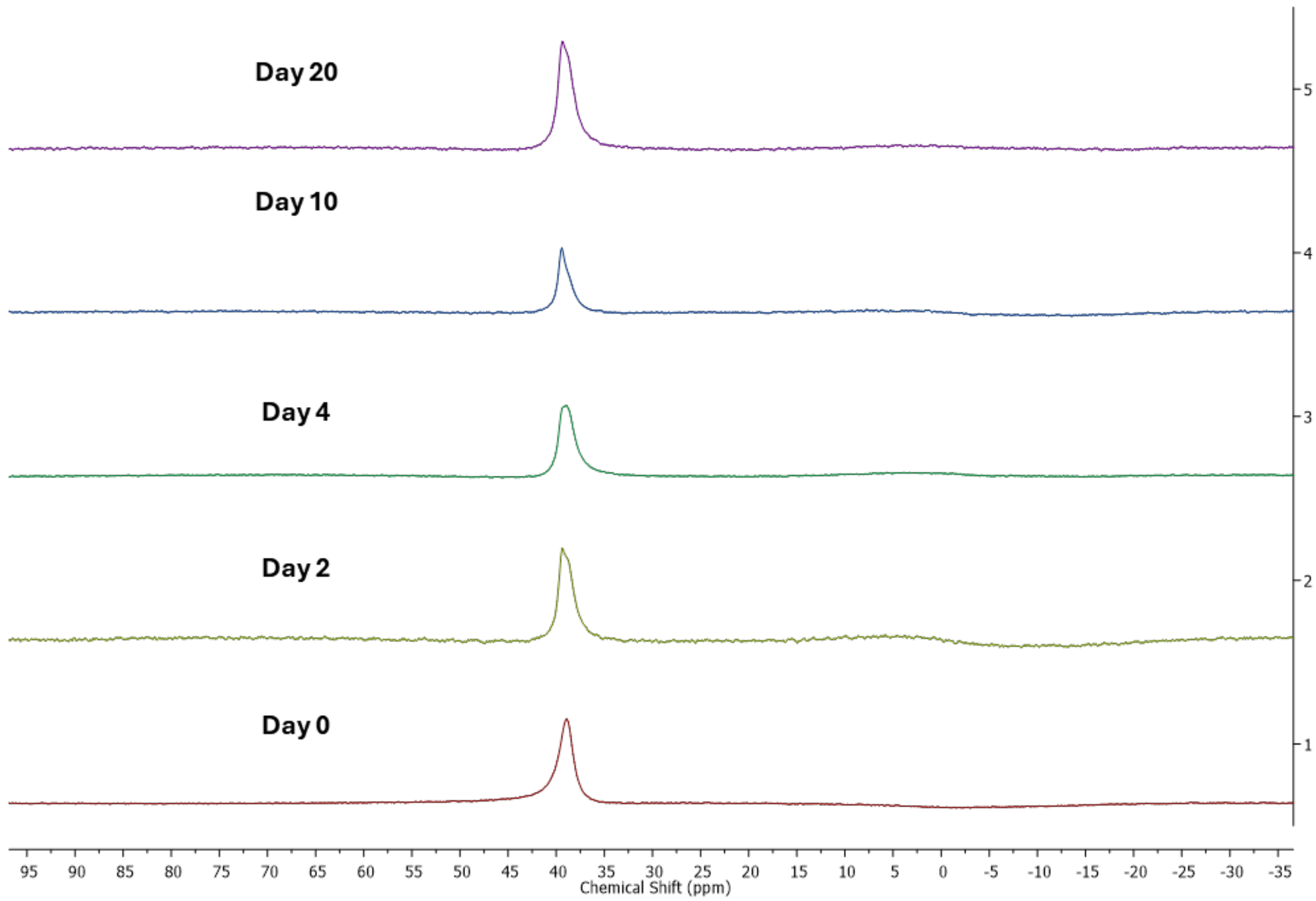

Figure S9: Stacked ^{11}B NMR spectra (160 MHz, Benzene-d_6) of compound **6g** recorded over several days.

2.4. X-ray structure plane analysis of compound 3 and 4-DMAP adduct

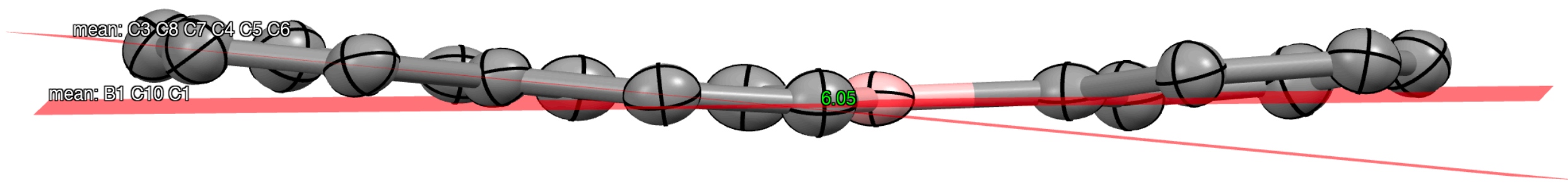

Figure S10: Planes between the two fused rings in the X-ray structure of borepin **3**.

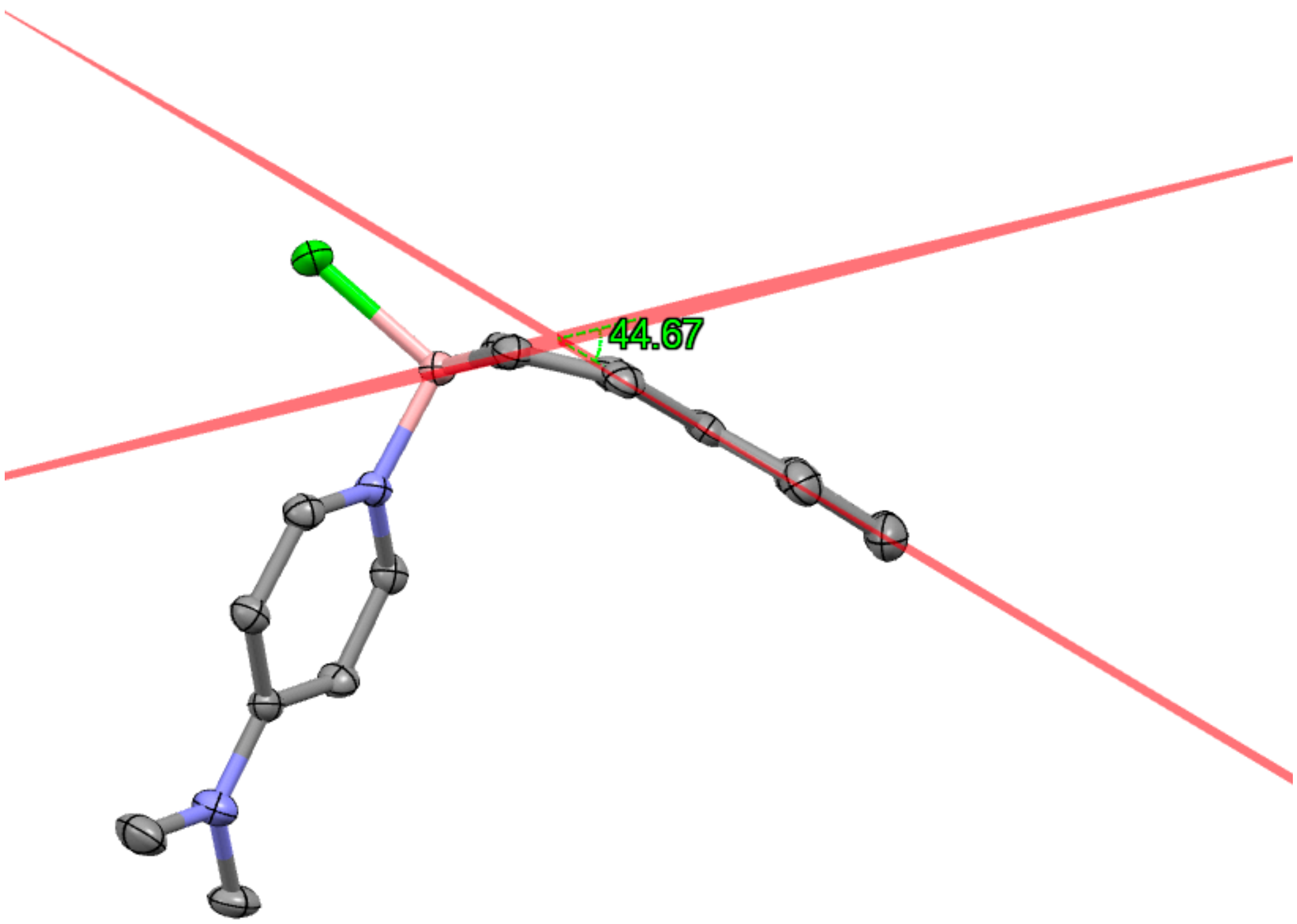

Figure S11: Planes between the two fused rings in the X-ray structure of borepin **4**-DMAP.

2.5 Procedure for a formation of Int-3'

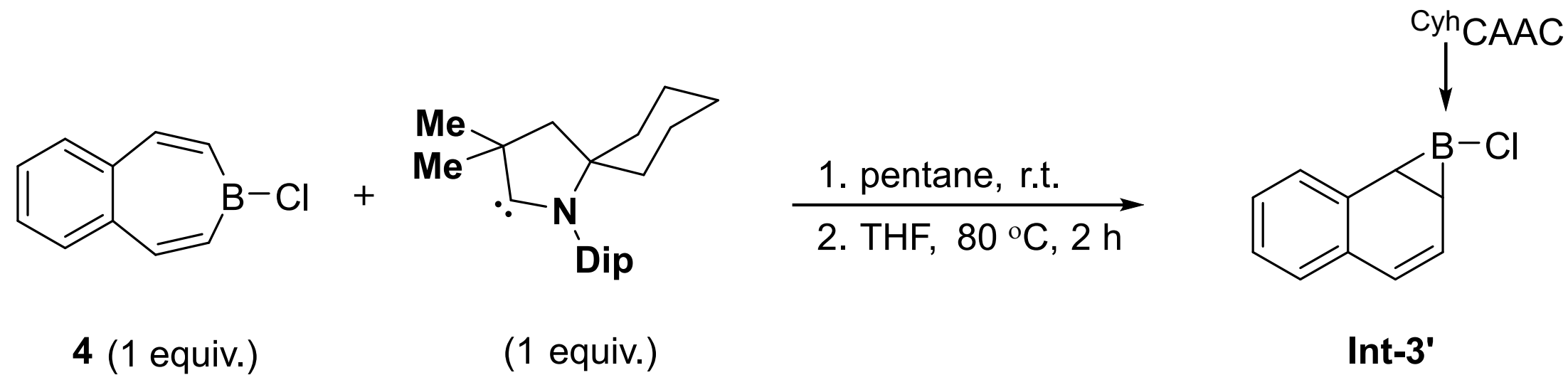

In a nitrogen-filled glovebox, an oven-dried 4 mL glass vial was charged with BP-Cl **4** (25 mg, 0.14 mmol, 1.0 equiv.) and ^{Cyh}CAAC (46.7 mg, 0.14 mmol, 1.0 equiv.) in anhydrous pentane (1 mL). The immediate formation of a white precipitate was observed. The pentane was decanted, and the resulting solid was washed with pentane and dried under reduced pressure. The obtained solid was then dissolved in THF (0.4 mL), transferred to a J. Young NMR tube, which was sealed inside the glovebox, removed from the glovebox, and heated in a preheated oil bath at 80 °C for 2 h. After cooling to room temperature, the solvent was removed under reduced pressure. Single crystals suitable for X-ray diffraction were obtained by slow evaporation of a DCM/pentane (1:1, 1 mL) solution of the crude mixture at room temperature.

Crystal data: $C_{33}H_{43}BClN$, Orange chunk, 0.62 x 0.39 x 0.14 mm^3, Monoclinic *P2₁/n*, a=10.5806(2)Å, b=18.1177(2)Å, c=14.8033(2)Å, α=90°, β=105.423(2)°, γ=90°, from 70172 reflections, Θ range for data collection 2.663° to 33.873°, T=100(2)K, V=2735.55(8)Å^3, Z=4, Fw=499.94, Dc=1.214 Mg·m^{-3}, μ=0.162 mm^{-1}.

Data collection and processing: Rigaku Synergy R diffractometer equipped with Hypix-Arc 100 detector, MoKα (λ=0.71073Å), -16≤h≤15, -27≤k≤26, -20≤l≤23, frame scan width = 0.25°, scan speed 1.0° per 1.2 sec, 70172 reflections collected, 9979 independent reflections (R-int =0.0421). The data were processed with CrysAlis^{PRO}.

Solution and refinement: Structure solved with SHELXT program. Full matrix least-squares refinement based on F^2 with SHELXL on 331 parameters with no restraints gave final R_1= 0.0418 (based on F^2) for data with I>2σ(I), wR_2= 0.1150 on 9979 reflections, and final R_1= 0.0524 (based

on F^2), wR_2= 0.1226 for all data, goodness-of-fit on F^2 = 1.054 largest electron density peak 0.519 $e\cdot Å^{-3}$. Largest hole -0.219 $e\cdot Å^{-3}$.

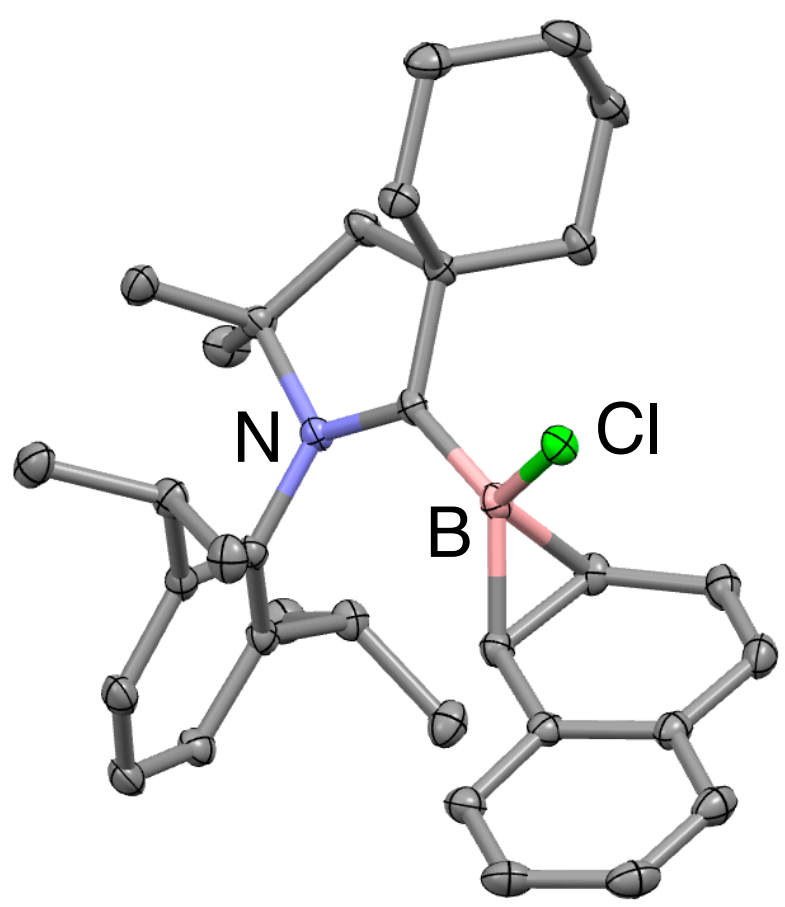

3. Computational Details

3.1. Geometry Optimizations

All quantum chemical calculations were performed with ORCA 6.1.1.[27] Geometry optimizations of all minima and transition state structures were carried out using density functional theory at the ωB97X-D3/cc-pVTZ level of theory with VeryTightOpt convergence criteria.[28-30] For each calculation, the DefGrid3 integration grid was employed with VeryTightSCF criteria. Solvent effects were included implicitly throughout using the conductor-like polarizable continuum model (CPCM) with toluene as the solvent.[31]

Initial transition state guesses connecting the different intermediates were generated using the nudged elastic band method as implemented in ORCA.[32,33] These preliminary transition state searches were performed at the ωB97X-D3/def2-SVP level of theory using the TIGHT-NEB-TS option.[32-34] The resulting transition state candidate geometries were then reoptimized at the higher ωB97X-D3/cc-pVTZ level of theory using the calculation settings described above.

For transition state optimizations, the Hessian was calculated before the optimization process and and recalculated every 25 steps. Analytical harmonic vibrational frequency calculations were performed to characterize all optimized stationary points. Minima were confirmed by the absence of imaginary vibrational frequencies, whereas transition states were confirmed by the presence of a single imaginary frequency corresponding to the intended reaction coordinate. An open-shell singlet electronic variant of **TS-3** was obtained using broken-symmetry DFT as implemented in Orca. To this end, the optimized closed-shell cartesian coordinates of **TS-3** were re-optimized on the triplet surface at the UKS-ωB97X-D3/def2-SVP level. The triplet transition state was then used to as an initial guess for the broken-symmetry calculation at the same level of theory by setting the target spin of the system to a singlet and flipping the spin on the boron atom. Finally, the open-shell singlet calculated with the def2-SVP basis was re-optimized at the UKS-ωB97X-D3/cc-pVTZ level.

Intrinsic reaction coordinate (IRC) calculations were performed from the optimized transition state structures to verify their connectivity to the corresponding reactant and product minima.[35] IRC calculations used the same electronic structure settings as the optimization process. Each IRC calculation was propagated for 75 steps in both the forward and reverse directions from the transition state structure.

3.2 Thermochemistry and Free Energy Calculations

The free energies of the reactants, intermediates, transition states, and products at 300 K were determined from their electronic energies and vibrational frequencies obtained using normal mode analysis. The potential energy was extrapolated to the complete basis set limit using single point energies computed with the cc-pVXZ (X = D, T, and Q) basis set series.[30,36] The extrapolation was performed using a three-point fit of the form:

$$E_X = E_{CBS} + A\,X^{-\alpha}$$

where α = 3.0. Harmonic vibrational frequency calculations were performed at the same level of theory as the final geometry optimizations, ωB97X-D3/cc-pVTZ with CPCM toluene solvation. These frequency calculations were used to confirm the character of each stationary point and to compute zero-point energies, vibrational enthalpy corrections, and vibrational entropy corrections. Because of the importance of the relative stability of the two isomers of **7a**, the ωB97M-V functional was used to calculate the change in their free energy at 300 K, as a comparison. This functional was used to optimize each isomer of **7a**, calculate the vibrational frequencies and perform a CBS extrapolation of the potential using the same computational settings outlined above.

Translational and rotational contributions to the free energy were evaluated using the rigid-rotor expressions at a 1 atm standard state. Vibrational zero-point energies and vibrational enthalpy corrections were computed from the non-imaginary vibrational frequencies. Low frequency vibrational entropies were treated using the Grimme quasi rigid-rotor harmonic oscillator correction.[37] In this treatment, low frequency modes are interpolated between harmonic oscillator and free rotor entropy limits. A frequency cutoff of 100 cm^{-1}, damping exponent of 4, and average moment of inertia parameter of 1.0×10^{-44} kg m^2 were used. For naphthalene, a rotational symmetry number of σ = 4 was included in the rotational partition function.

Finally, because the molecular thermochemical quantities were initially evaluated using a 1 atm translational standard state, reaction free energies were converted to a 1 M solution standard state using[38]:

$$\Delta G^{\circ}(1\,M) = \Delta G^{\circ}(1\,atm) + \Delta nRT\,ln(C^{\circ}RT/p^{\circ})$$

where Δn is the change in moles between products and reactants, C° = 1 mol L^{-1} = 1000 mol m^{-3}, and p° = 1 atm. This correction was applied automatically for each elementary step based on the number of molecular species in the corresponding product and reactant definitions. At 300 K and

1 atm, this correction is approximately 1.89 kcal mol⁻¹ per unit change in molecularity. Thus, association steps with $\Delta n = -1$ are shifted by approximately −1.89 kcal mol⁻¹, dissociation steps with $\Delta n = +1$ are shifted by approximately +1.89 kcal mol⁻¹, and isomolecular steps with $\Delta n = 0$ are unchanged.

3.3. Energy profile for alternative borylene formation from Int-2

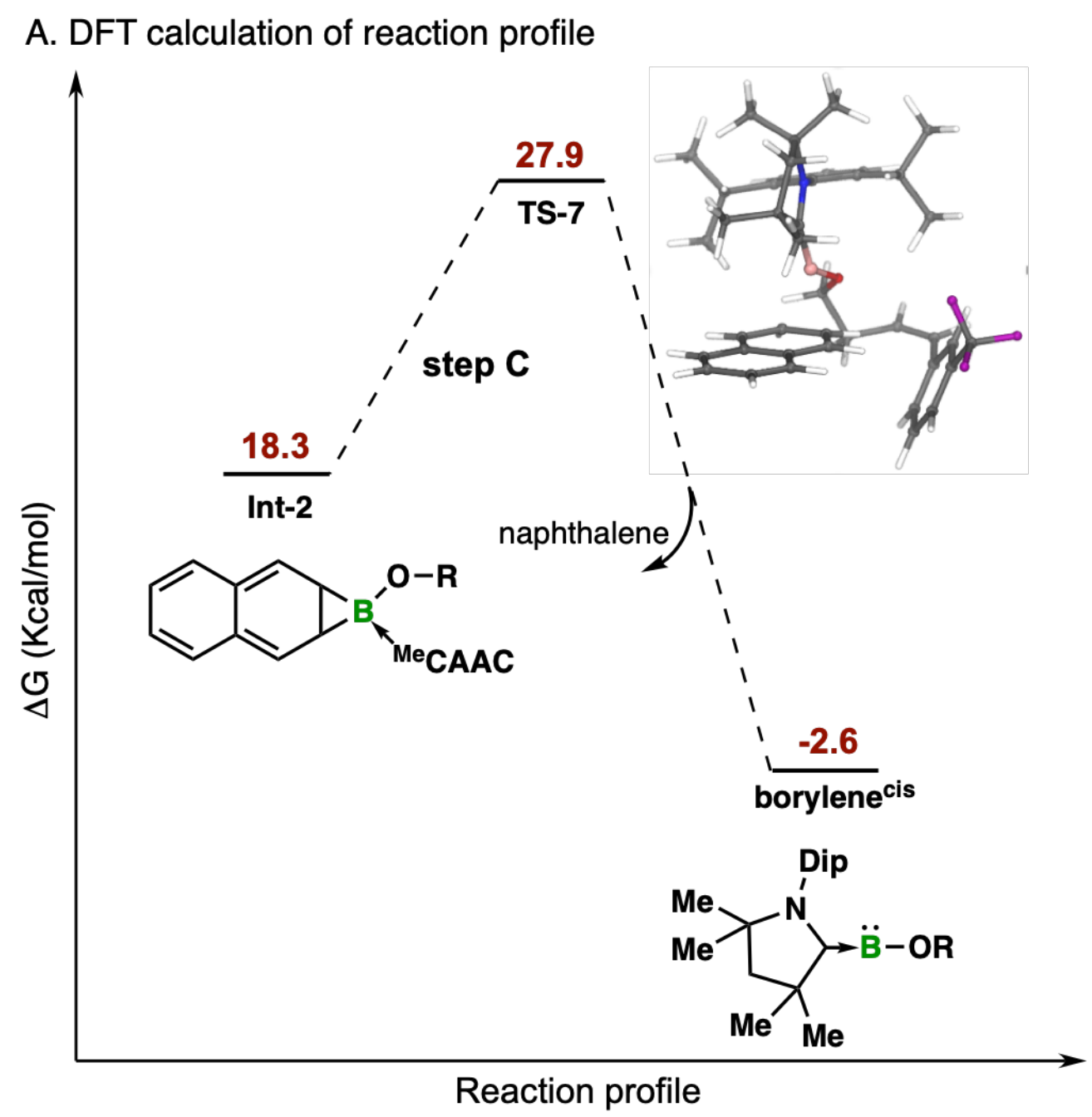

Figure S12: Energy profile for direct borylene formation from **Int-2**.

Table S2: Free energies of intermediates and transition-states:

	cis	***cis*-alternative**	***trans***
Reactants	0	N/A	-1.83
TS1	8.4	N/A	7.85
Adduct	-7.08	N/A	-9.36
TS2	23.28	25.05	21.98
Borirane Intermediate	18.27	20.29	20.45
TS3	25.35	27.98	24.53
TS3 (Open-Shell Singlet)	13.61	N/A	N/A
Borirane	-2.69	-0.87	-1.84
TS4	17.56	20.13	18.82
Borylene (singlet) + Naphthalene	-2.63	N/A	-4.98
Borylene (triplet) + Naphthalene	10.66	N/A	13.83
TS5	8.15	N/A	10.04
borirane	-34.37	N/A	-33.52
TS6	-0.12	N/A	-0.12
TS7	27.86	30.25	27.73

	Cis	***cis-alternative***	***trans***
Borepin	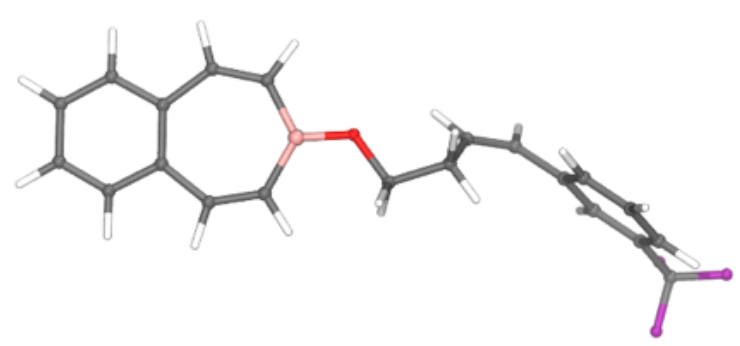		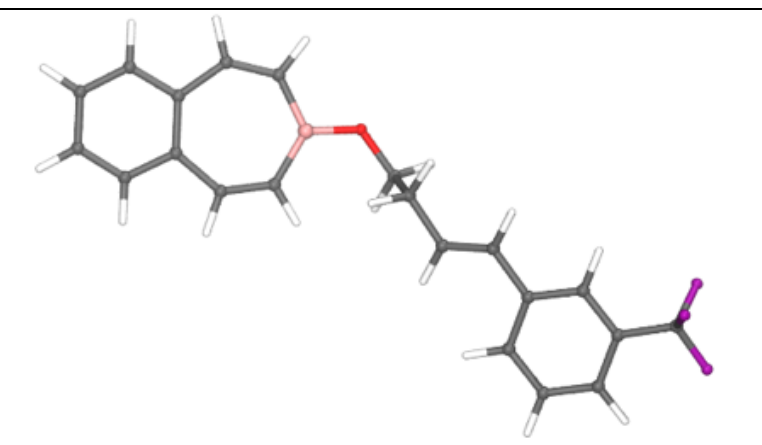
TS1	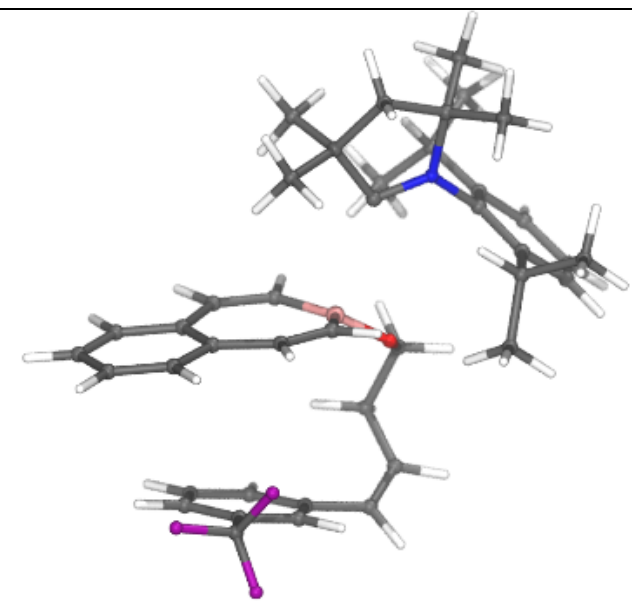		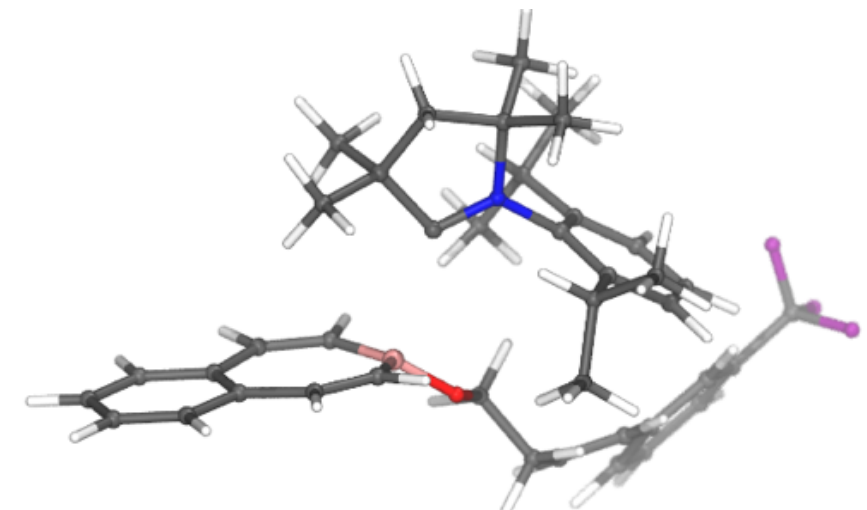
Int-1	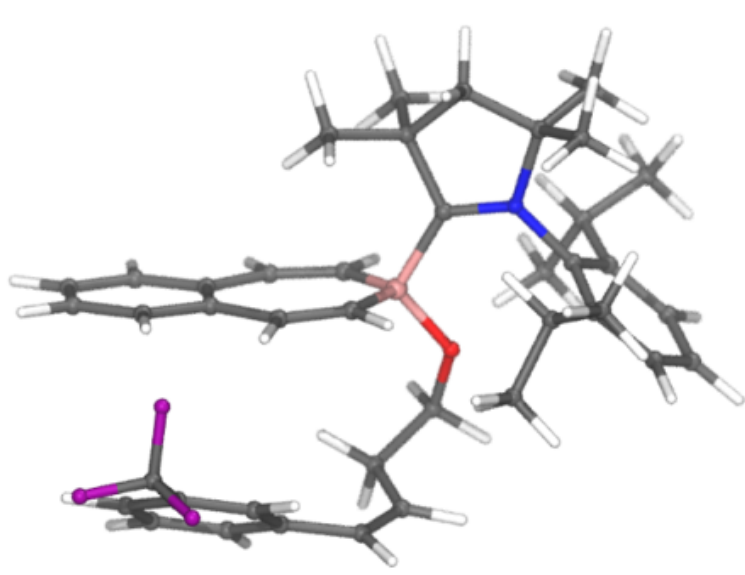		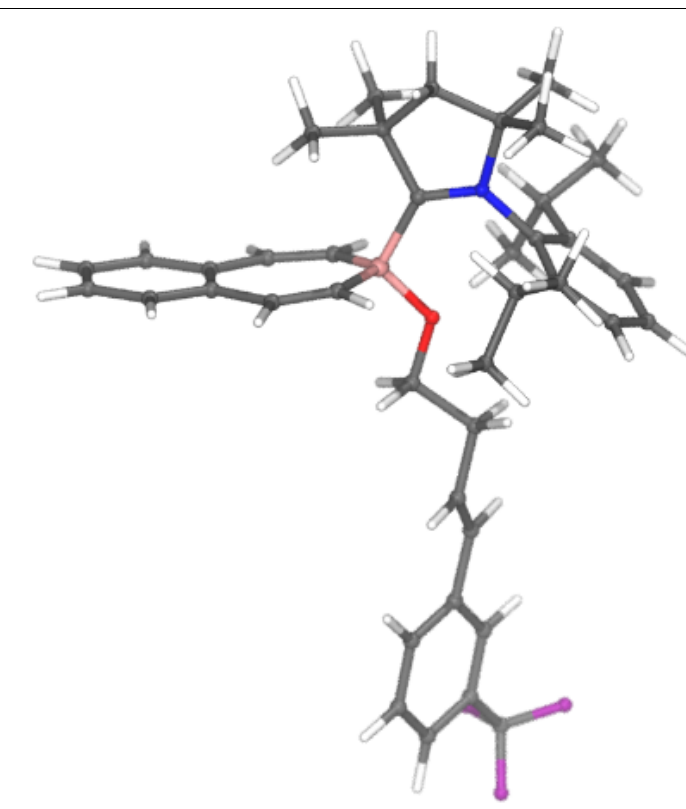

TS2	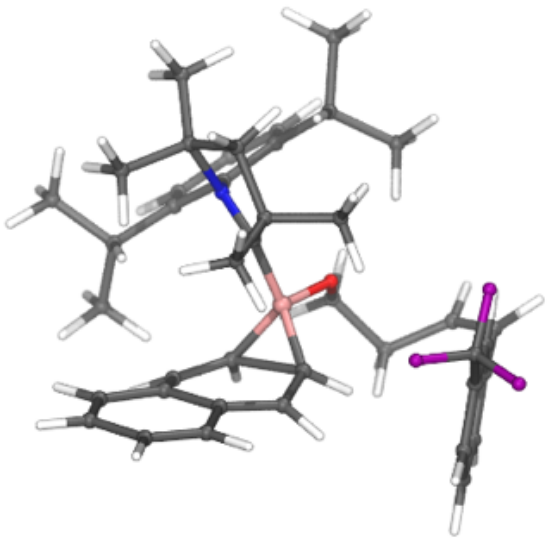	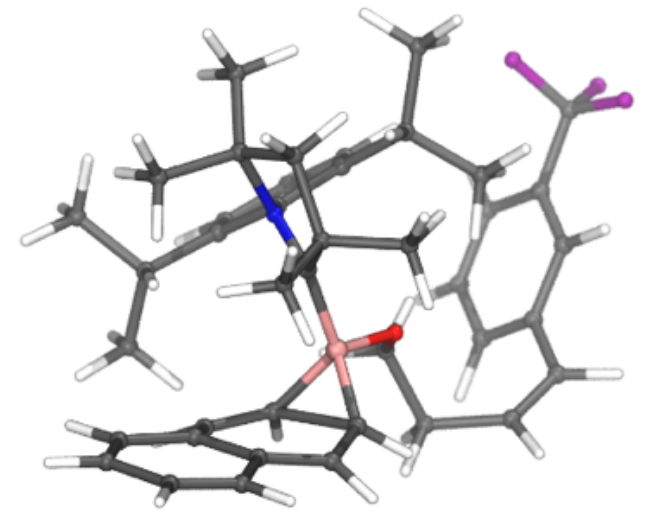	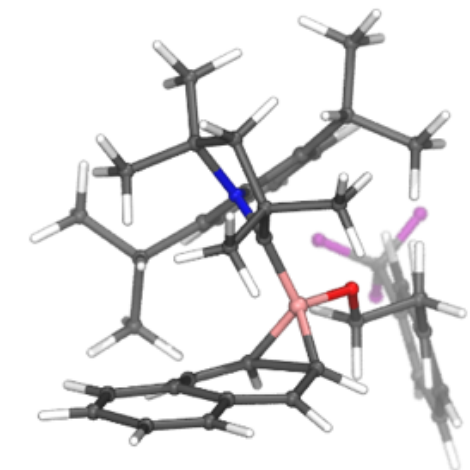
Int-2	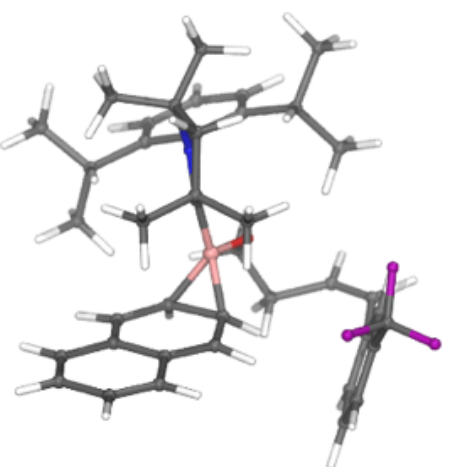	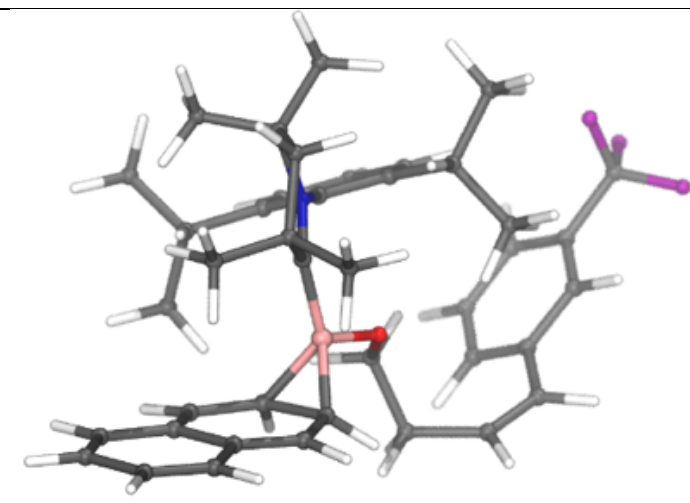	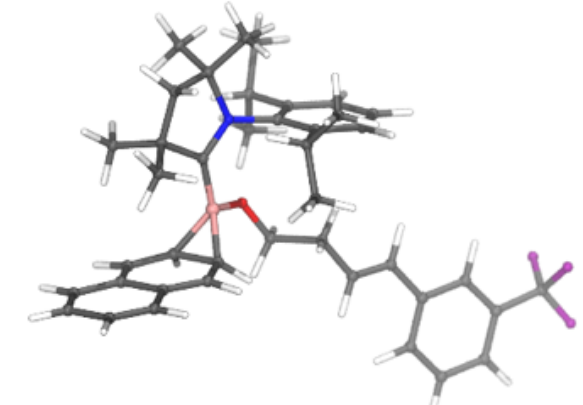
TS3	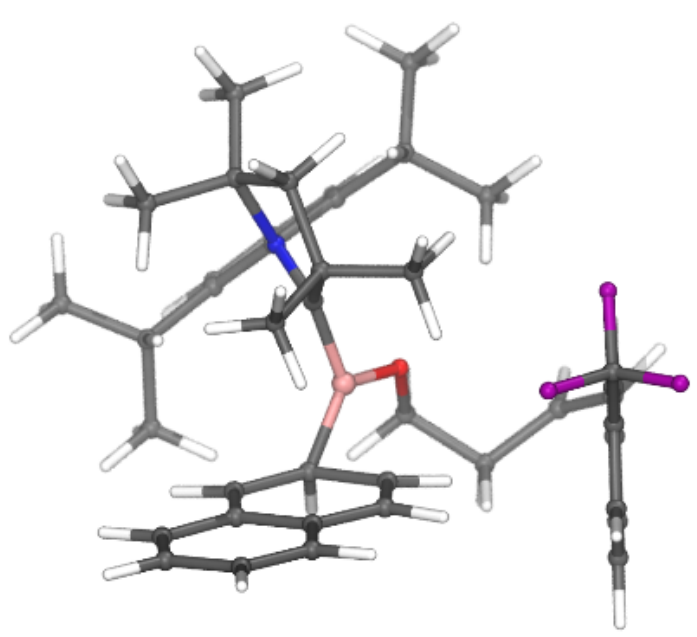	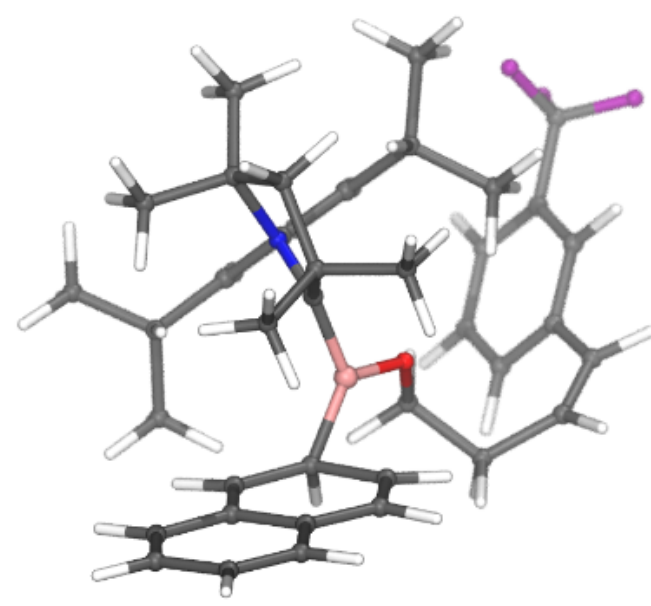	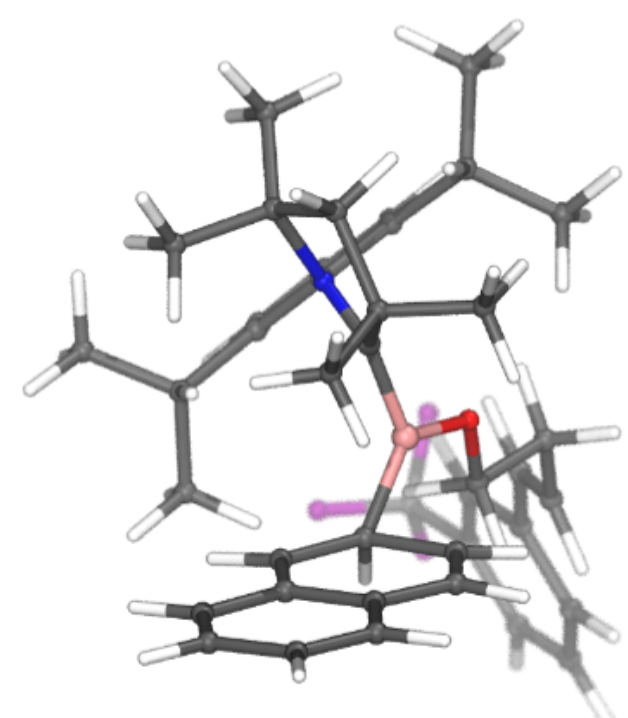
TS3 OSS	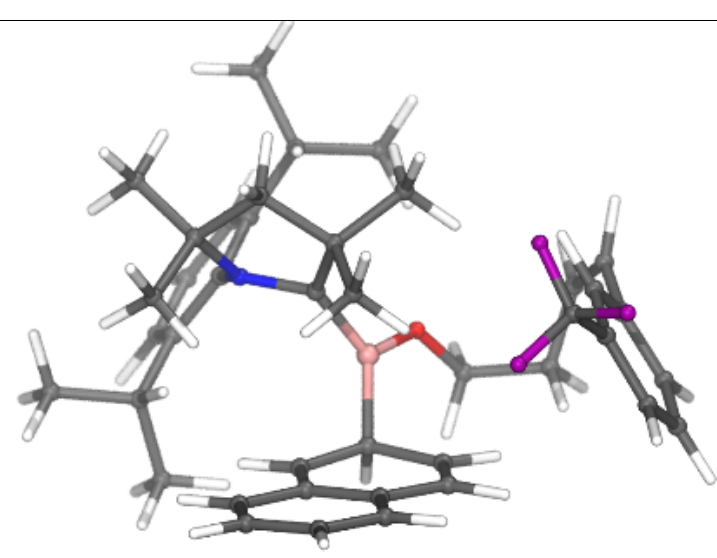		
Int-3	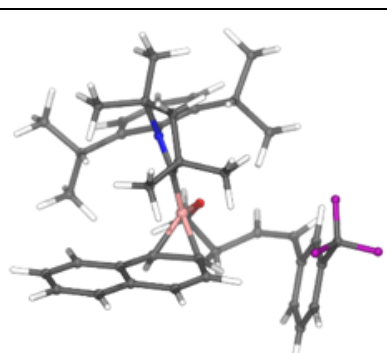	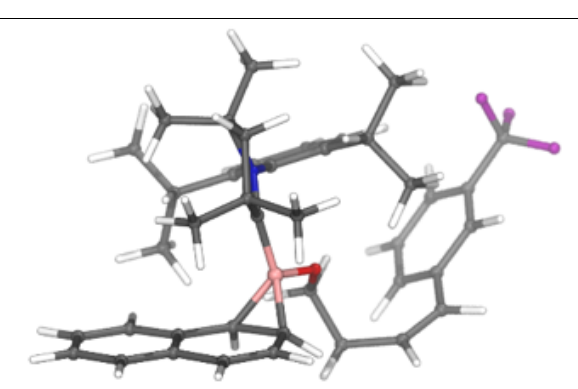	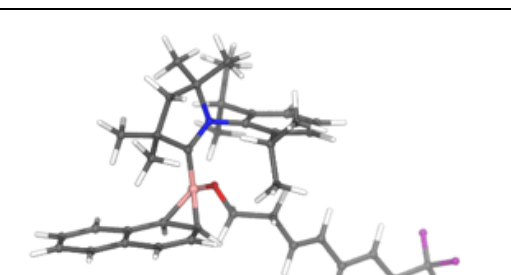

TS4	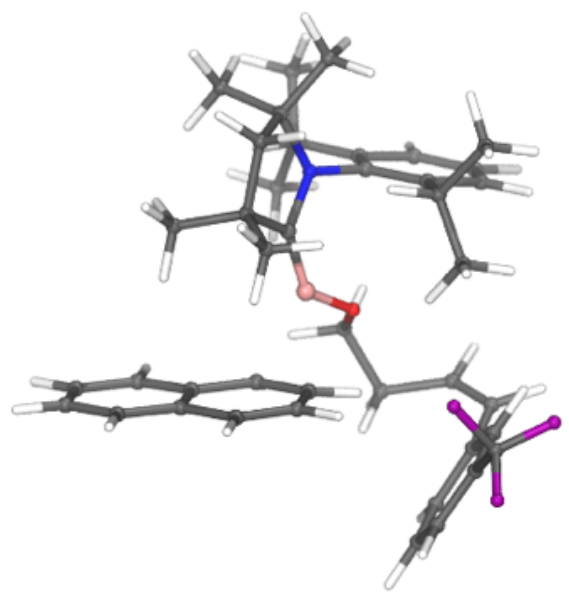	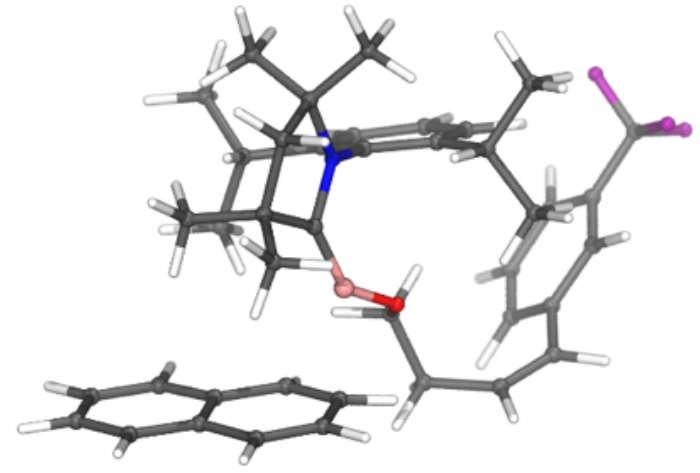	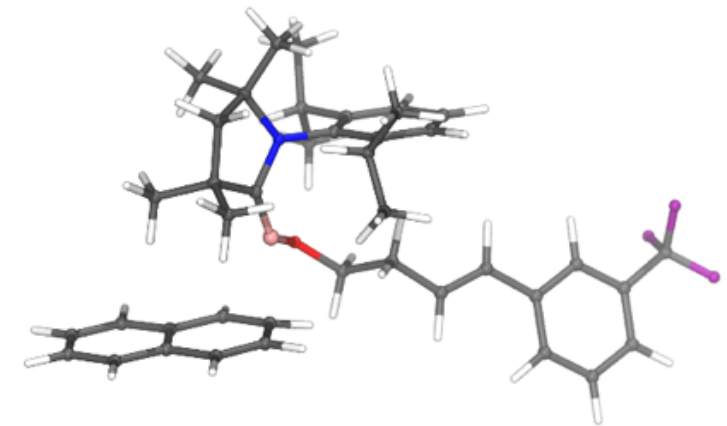
Borylene	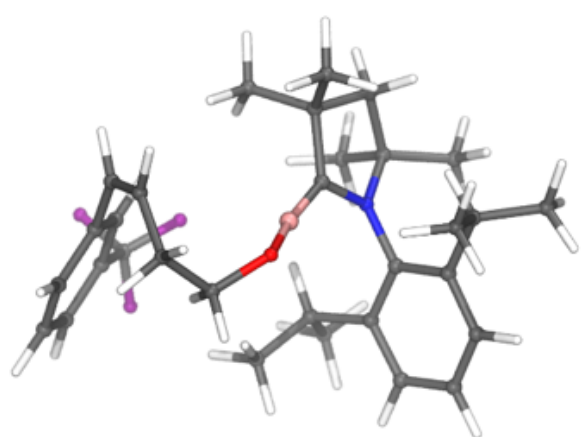		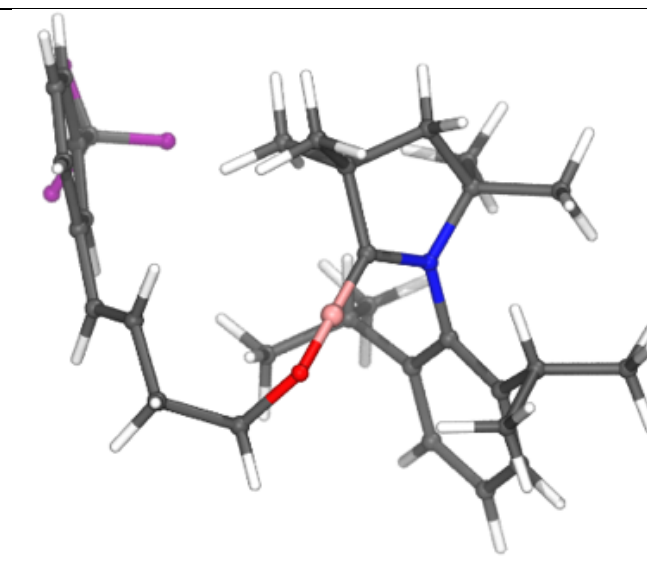
Borylene (triplet)	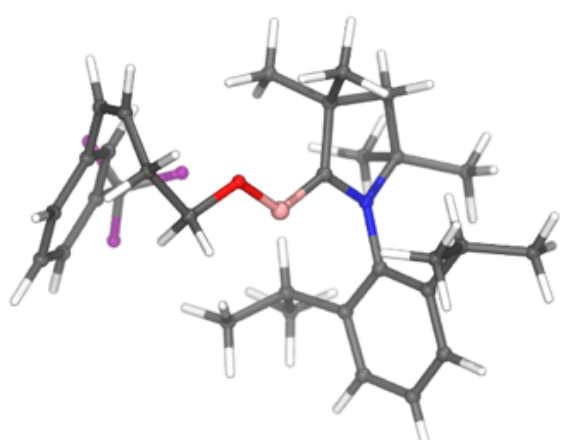		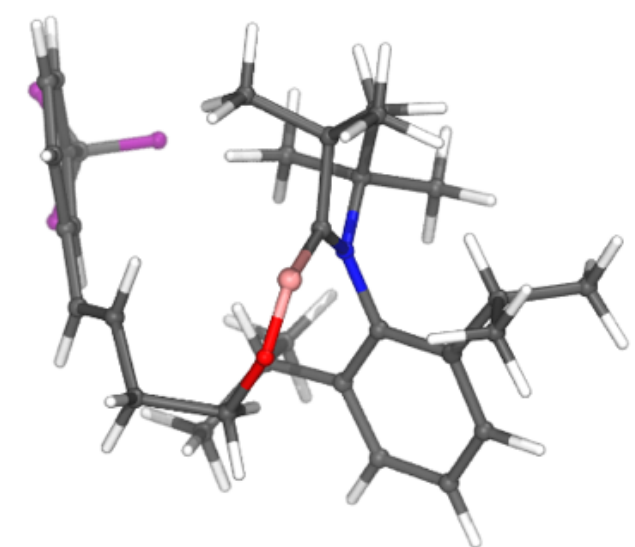

TS5 and TS5’	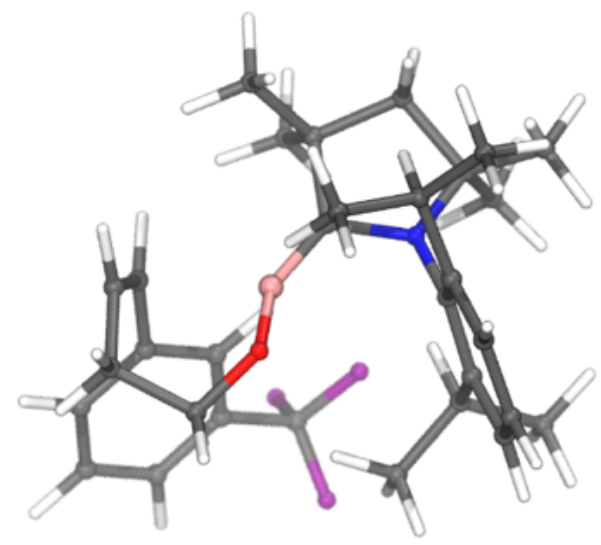		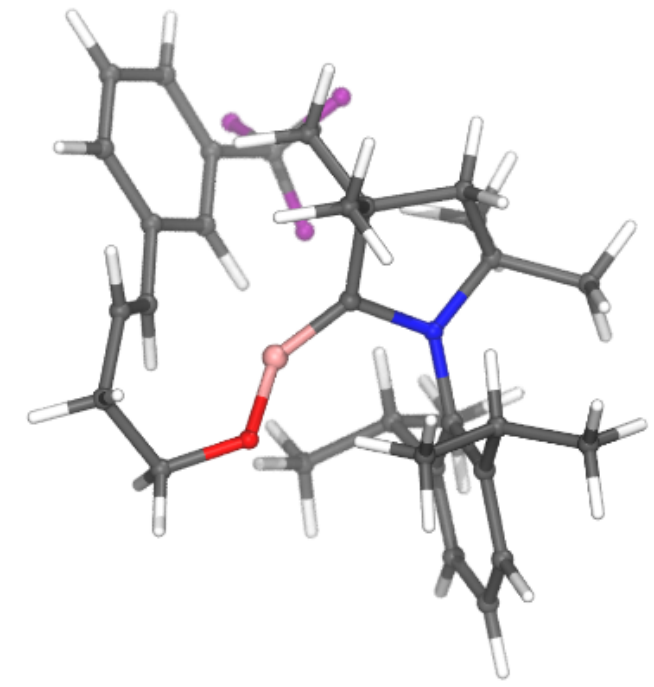
7a and 7a’	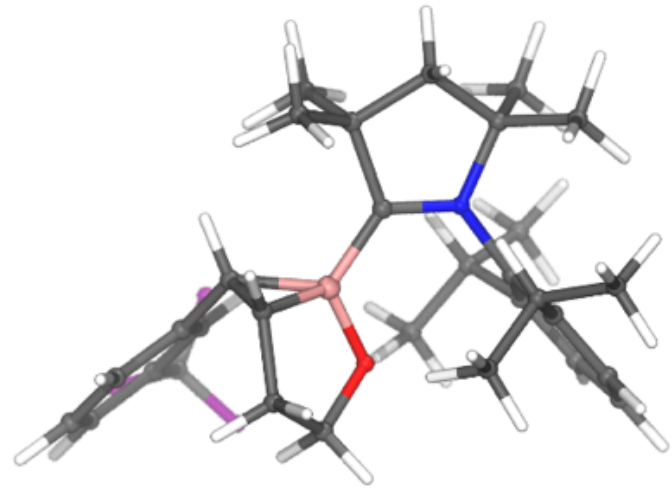		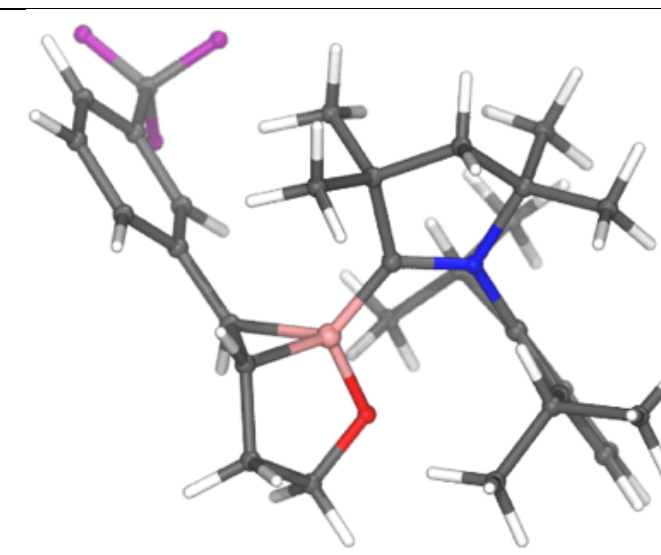
TS6	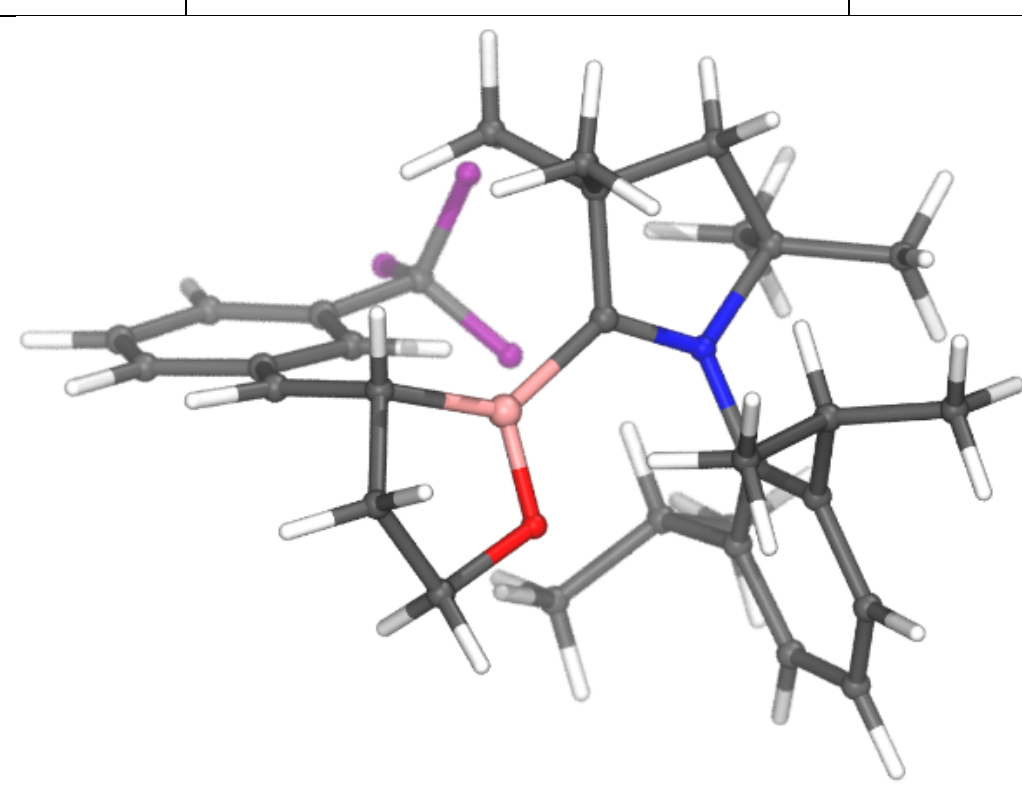		
TS7	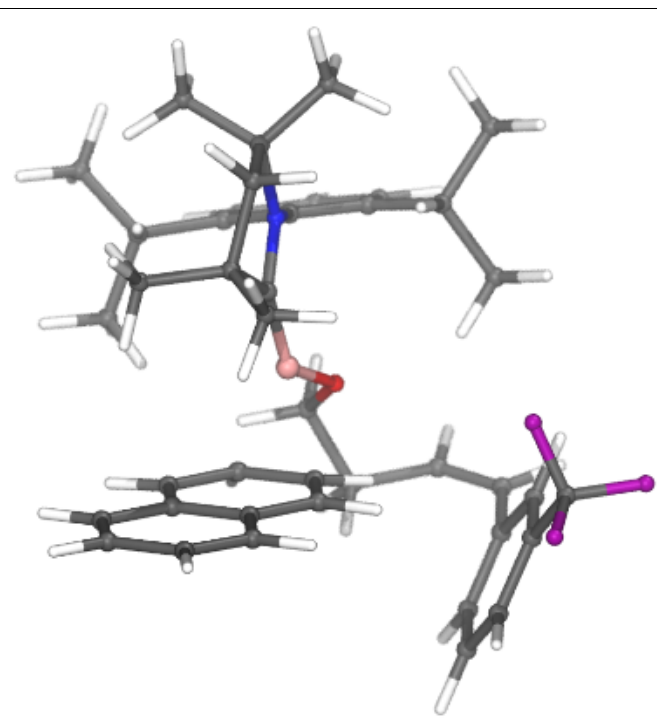	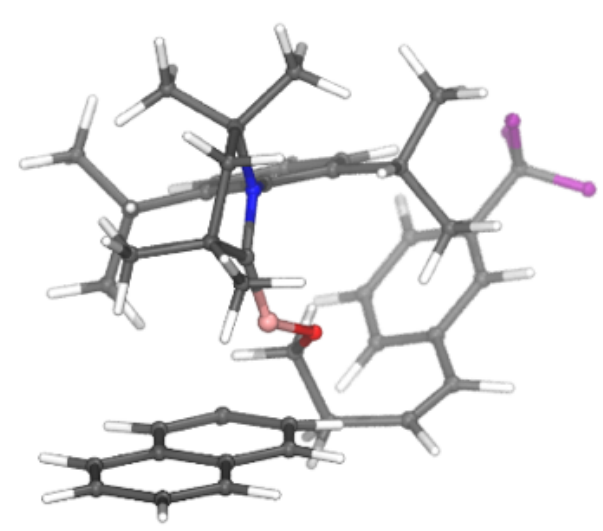	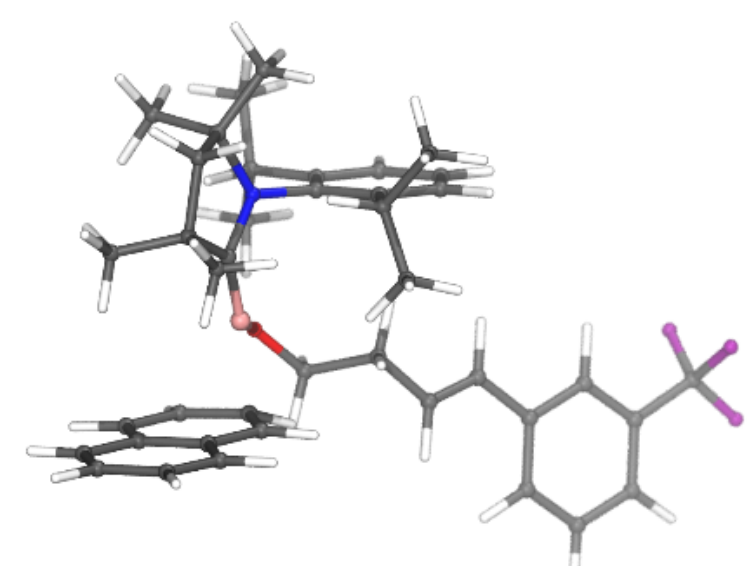

4. Notes

4.1 Evaluation of different Lewis bases

During our evaluation of various Lewis bases for promoting borylene formation, we found that, unlike CAAC, other Lewis bases, including N-heterocyclic carbenes (NHCs), 4-dimethylaminopyridine (DMAP), and phosphines, were ineffective in generating the corresponding borylenes. Specifically, the reactions of compound **6a** with ^{Dip}NHC and DMAP afforded only the corresponding Lewis base-coordinated adducts at room temperature. Attempts to promote borylene formation by conducting these reactions at elevated temperatures (80–110 °C) were also unsuccessful. In contrast, no coordination was detected with phosphines, and the borepin precursor remained unreacted under the reaction conditions.

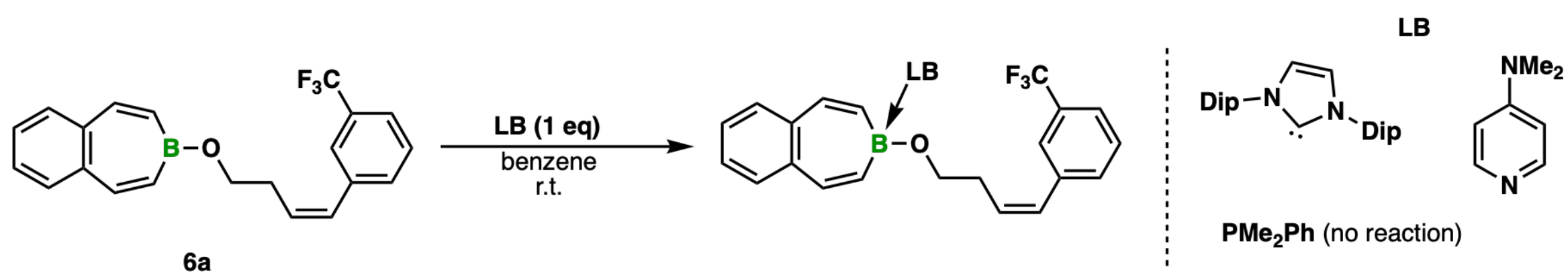

4.2 Cycloaddition reaction with terminal alkenes

The reaction afforded the corresponding borirane derivative, indicating that the transformation is also feasible with terminal alkene.. This was confirmed using ^{1}H and ^{11}B NMR and HRMS spectrum of the reaction mixture, see spectra below. In this case, the ^{11}B NMR, as well as the ^{1}H NMR, is similar to other reported derivatives in this study.

However, repeated attempts to isolate this borirane product in pure form were unsuccessful due to its decomposition with time.

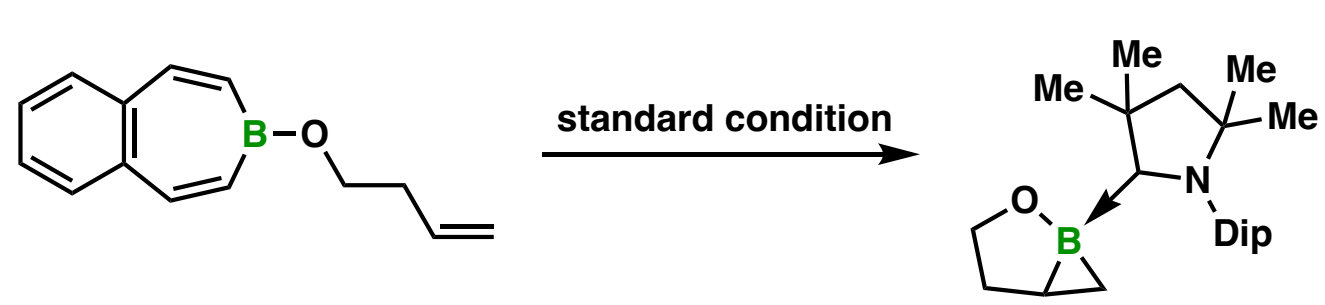

^{11}B NMR (160 MHz, Benzene-d_6) reaction mixture.

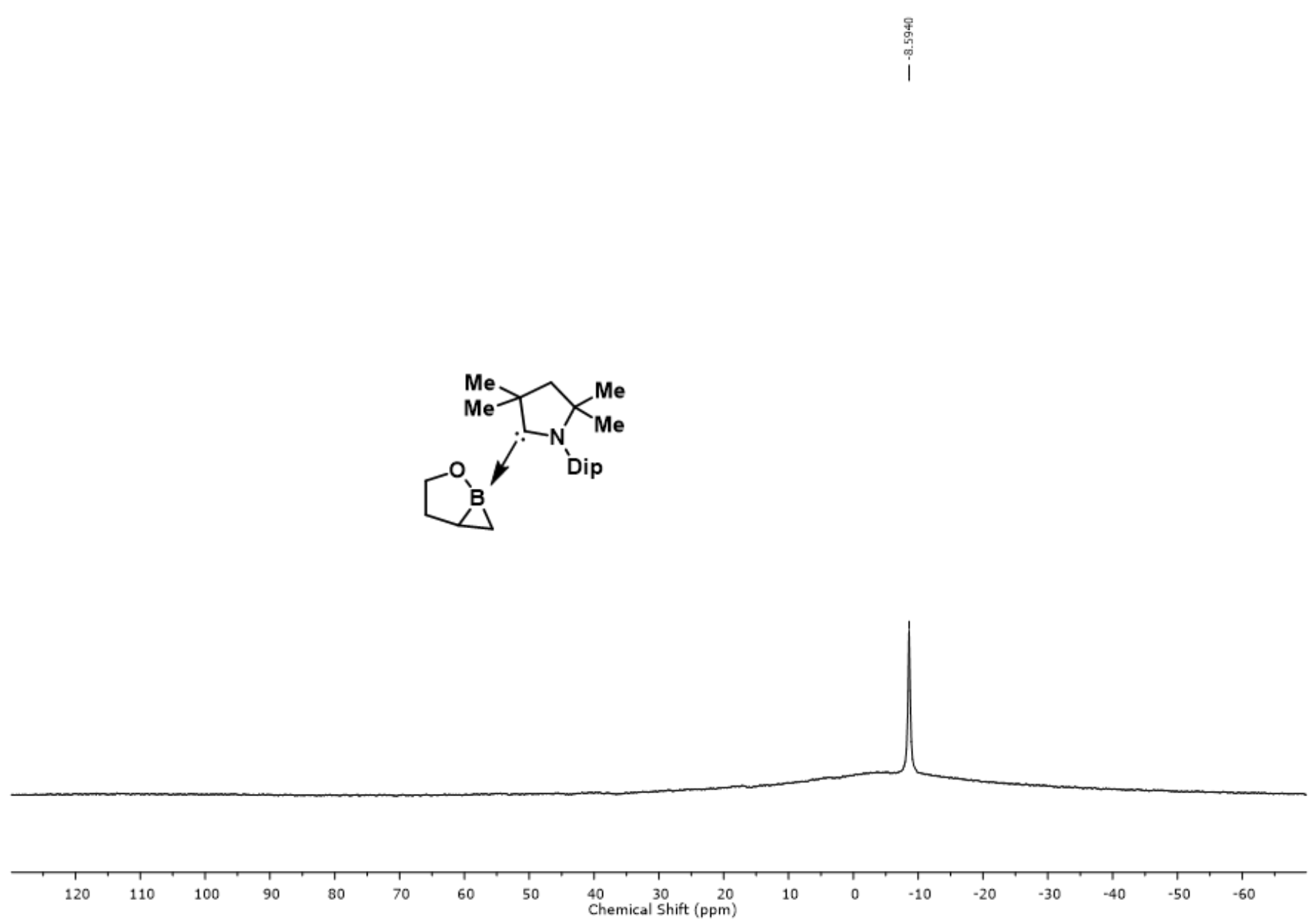

^{1}H NMR (500 MHz, Benzene-d_6) reaction mixture

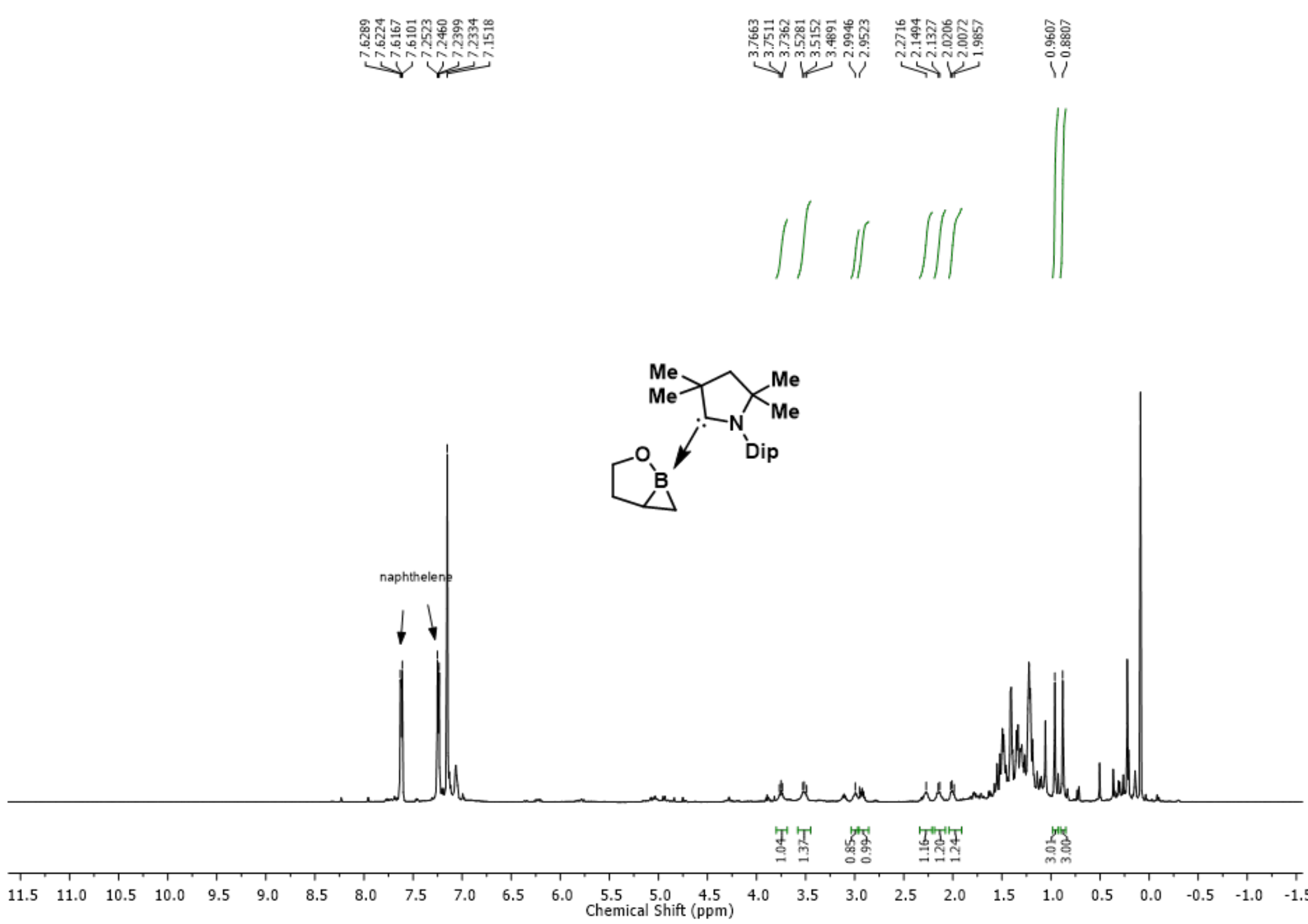

HRMS (ESI) m/z calcd for $C_{24}H_{39}BNO$ [M+H]$^{+}$: 368.3125; found: 368.3134.

Elemental Composition Report — Page 1

Single Mass Analysis
Tolerance = 5.0 PPM / DBE: min = -1.5, max = 500.0
Element prediction: Off
Number of isotope peaks used for i-FIT = 3

Monoisotopic Mass, Even Electron Ions
143 formula(e) evaluated with 1 results within limits (up to 50 closest results for each mass)
Elements Used:
C: 24-24 H: 10-60 B: 0-2 N: 0-8 O: 0-5

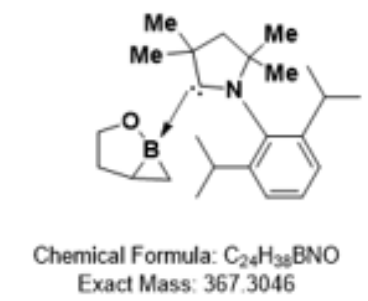

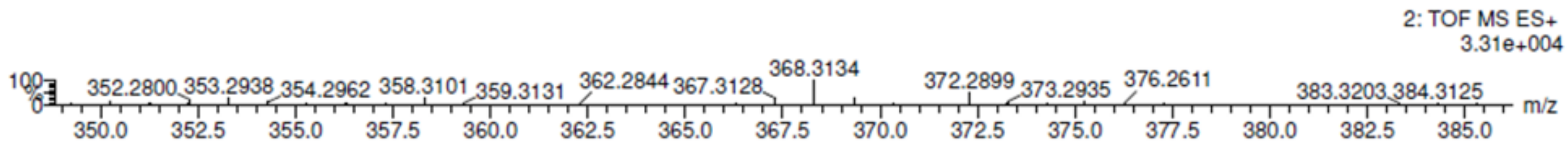

Mass	Calc. Mass	mDa	PPM	DBE	i-FIT	Norm	Conf(%)	Formula
Minimum:				-1.5				
Maximum:		5.0	5.0	500.0				
368.3134	368.3125	0.9	2.4	6.5	487.3	n/a	n/a	C24 H39 B N O

4.3 Proposed mechanism

For the conversion of **7g** to **8**:

In the presence of hydroxide base, borirane **7g** is converted to product **8** via hydroxide-assisted selective cleavage of the exocyclic B–C bond of the borirane, accompanied by the formation of a new B–OH bond. The product **8** was isolated and along with the byproduct **A** which was isolated and characterized. Based on these findings, we proposed two plausible pathways for this transformation.

Proposed pathway 1

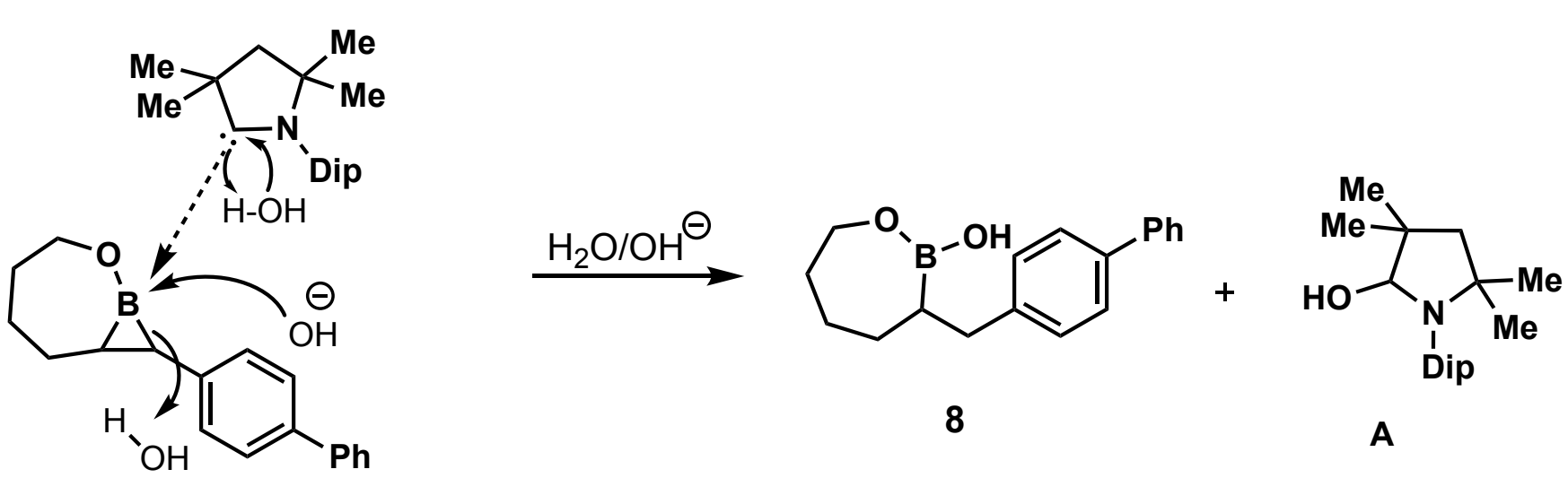

Proposed pathway 2:

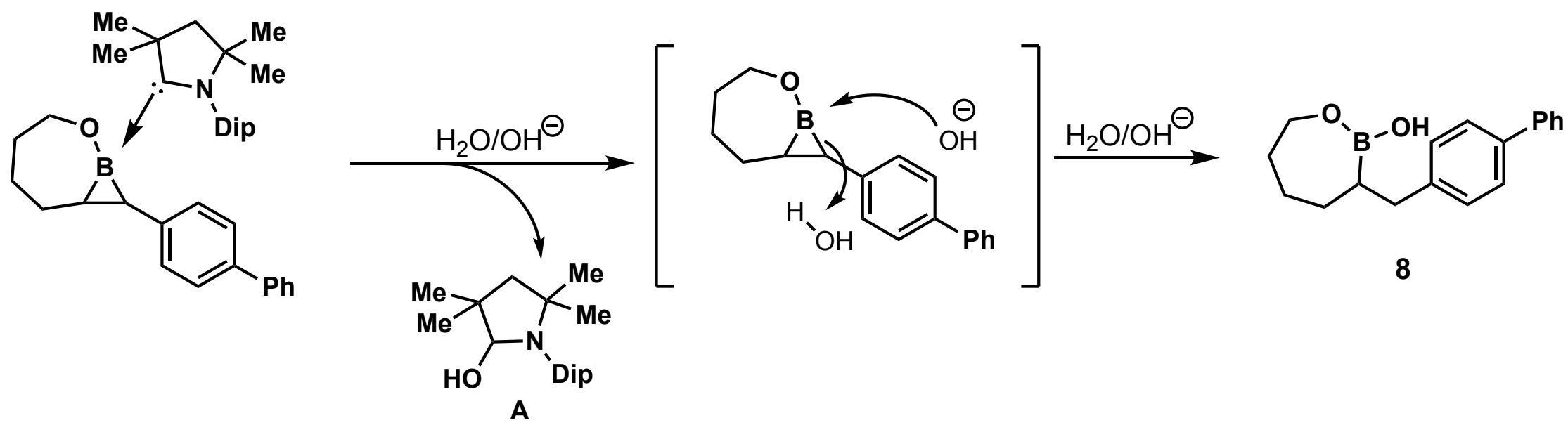

<u>For the conversion of **7g** to **9:**</u>

The stereochemistry for product **9** is proposed based on concerted pathway to form the boron hydride and subsequent hydride transfer to CAAC give the final product (see Scheme 8 and references for mechanism details). Further, stereochemical assignment is based on the solid-state structure of the analogous compound **14**, as determined by single-crystal X-ray diffraction. We believe that the regio-selectivity in this reaction arises from the pathway that minimizes steric hindrance between the bulky CAAC ligand and the aryl substituents. A hydride shift from the exocyclic carbon atom would lead to a structure with significantly higher steric repulsion due to the geminal positioning of the CAAC-bearing boron atom and biphenyl groups.

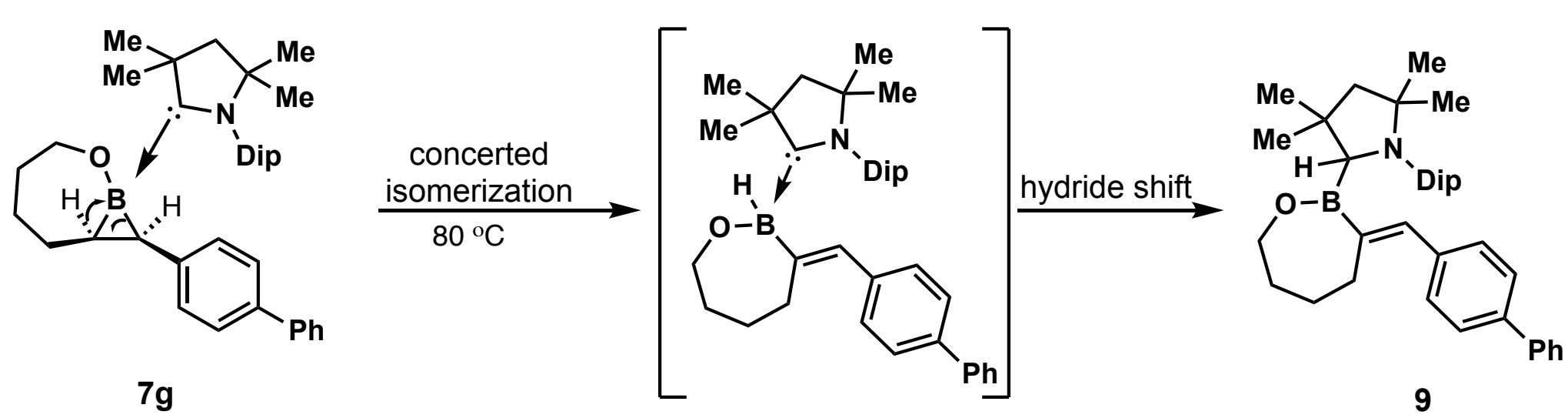

For the conversion of **7g** to **12** is proposed to proceed via vinyl borane intermediate **9**, followed by formation of the coupling product through the standard Suzuki–Miyaura cross-coupling mechanism. The same vinyl borane intermediate was subjected to oxidation using sodium perborate, yielding the ketone **11**. Whereas, oxidation of borirane **7g** provided the 1,5-diol **12**.

4.4 Aromaticity comparision of chloro borepin 4 and 6a

The alkoxy-substituted borepin **6a** exhibits a relatively upfield α-proton resonance (6.76 ppm) compared to the corresponding chloro borepin **4** (δ = 7.23 ppm). This upfield shift for **6a** is consistent with increased π-donation from the alkoxy substituent to the electron-deficient boron center, which reduces the ring current and consequently diminishes the aromatic character of the borepin relative to the B–Cl analogue **(**Figure S13).

^{1}H NMR (500 MHz, Benzene-d_6)

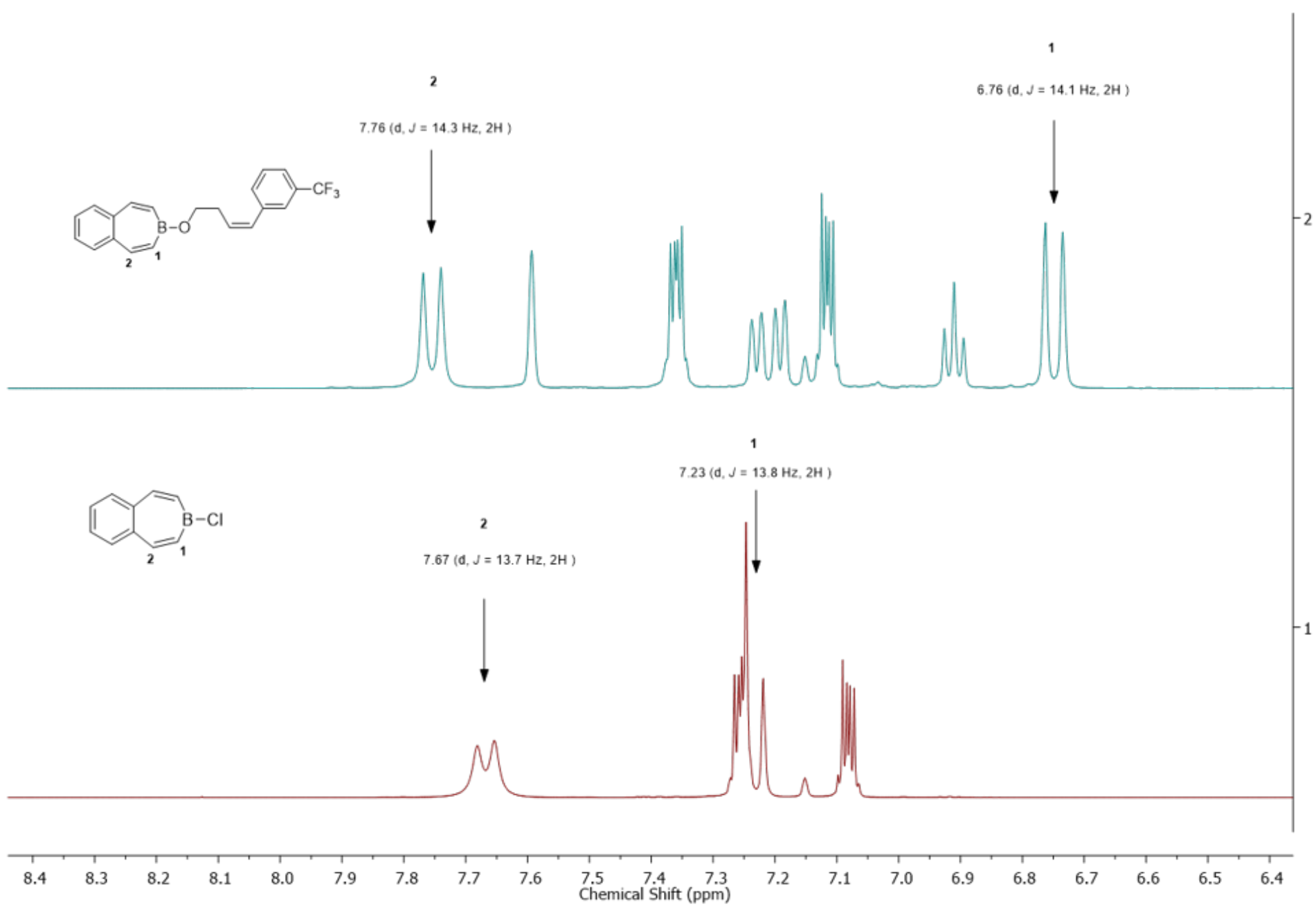

Figure S13. Comparsion of ^{1}H NMR spectra of borepin **4** and alkoxy-substituted borepin **6a**

4.5 Analysis of possible minor isomer

From the analysis of the reaction mixture during the formation of product **7a** from both **6a** and **6b**, we observed a small signal at -3.5 ppm adjacent to the major product signal in the ^{11}B NMR spectrum (**Figure S14a**). Similarly, an additional small signal at -63.36 ppm was observed in the ^{19}F NMR spectrum (**Figure S14b**). These signals persist in the NMR spectra of the major product even after purification by crystallization, indicating that this minor species shares similar physicochemical properties with the main compound. Based on these observations, we propose that these signals correspond to the minor *anti*-isomer of compound **7a**.

To fully confirm this assignment, structurally more informative methods are required. However, no well-resolved signals could be confidently assigned to the minor diastereomer in the ^{1}H NMR spectra; we observed only weak signal patterns in selected regions (**Figure S14c**). Furthermore, the sensitivity of ^{13}C and 2D NMR experiments was insufficient for definitive verification.

As mentioned in the manuscript, the structure of the major product was elucidated by 2D NMR experiments and unambiguously confirmed by X-ray crystallographic analysis. Therefore, the reaction was described as affording predominantly the *syn*-product. The concentration of the minor species is too low to allow its isolation and independent structural confirmation. Nevertheless, the DFT calculations and these weak NMR signals collectively suggest the presence of a minor amount of the alternative diastereomer. Based on the NMR integration, the quantity of the minor isomer is estimated to be 6–8%. This experimental ratio roughly correlates with the calculated energy difference between the two isomers. Using the experimental populations determined from NMR, the free energy difference between the *syn*/*anti* conformers is expected to fall between 1.45 kcal/mol and 1.63 kcal/mol. The ωB97M-V/CBS calculations gave a smaller free energy difference of 1.1 kcal/mol. However, the difference between the calculated and experimental values are well within the expected uncenertanties associated with using a single representative minimum rather than a full conformational ensemble for each isomer, density functional theory and an implicit solvent model to calculate the free energies. This comparison indicates that the observed product ratio is consistent with the calculated thermodynamic preference for the *syn* isomer. Mechanistically, the high barrier for TS-6 from the relaxed product minima suggests that *syn*/*anti* interconversion is unlikely after complete vibrational cooling. Instead, the initially formed borirane products may retain sufficient excess energy to access the *syn*/*anti* interconversion pathway before full relaxation and collisional cooling. As this excess energy is dissipated, the deeper *syn* product well can bias the final trapped distribution toward the experimentally observed *syn*-rich product mixture. In addition to this case, we observed a minor peak in the ^{11}B NMR spectra of a few other borirane derivatives.

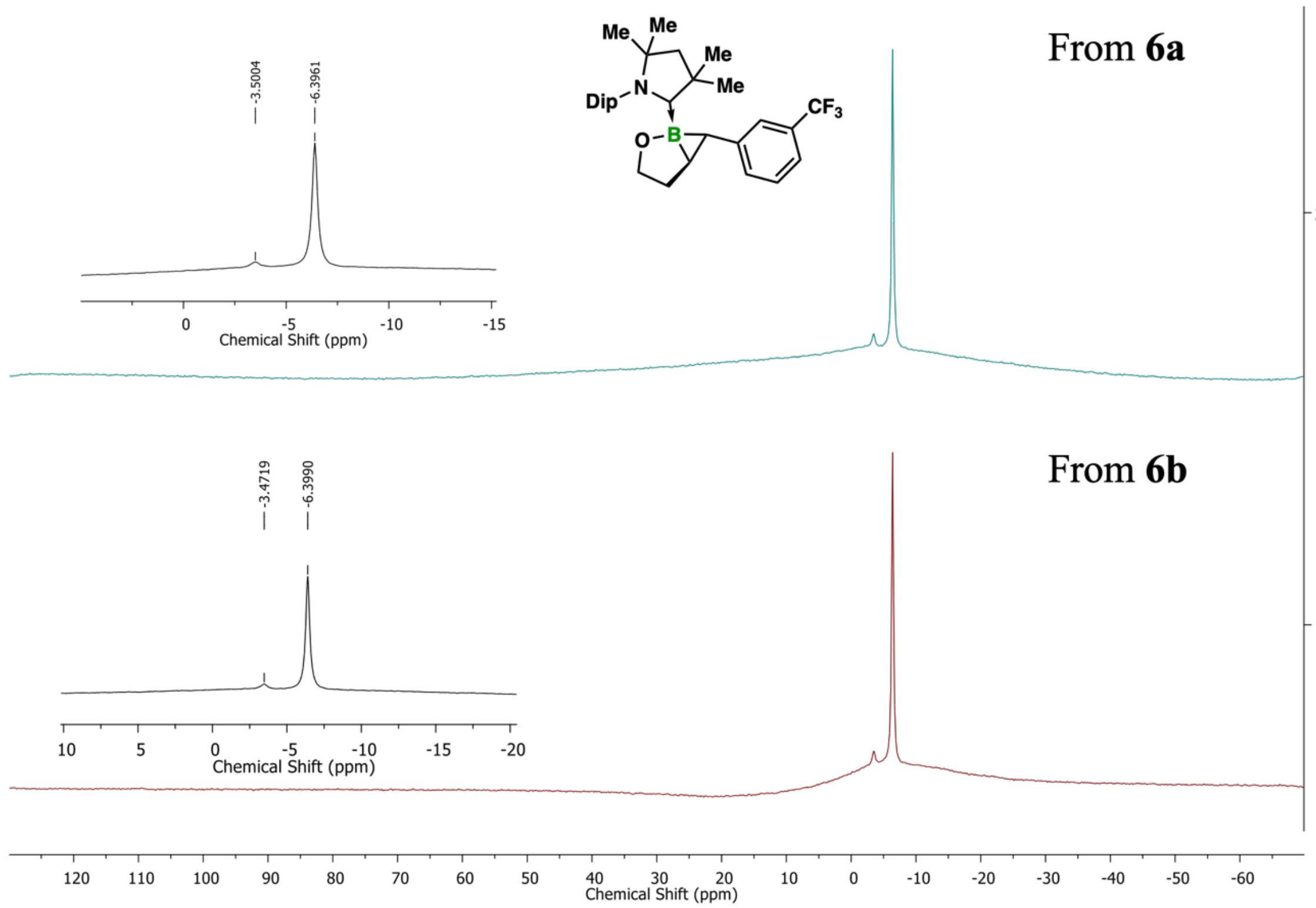

Figure S14a: Stacked ^{11}B NMR (160 MHz, Benzene-d_6) of **7a** from reaction **6a** and **6b**

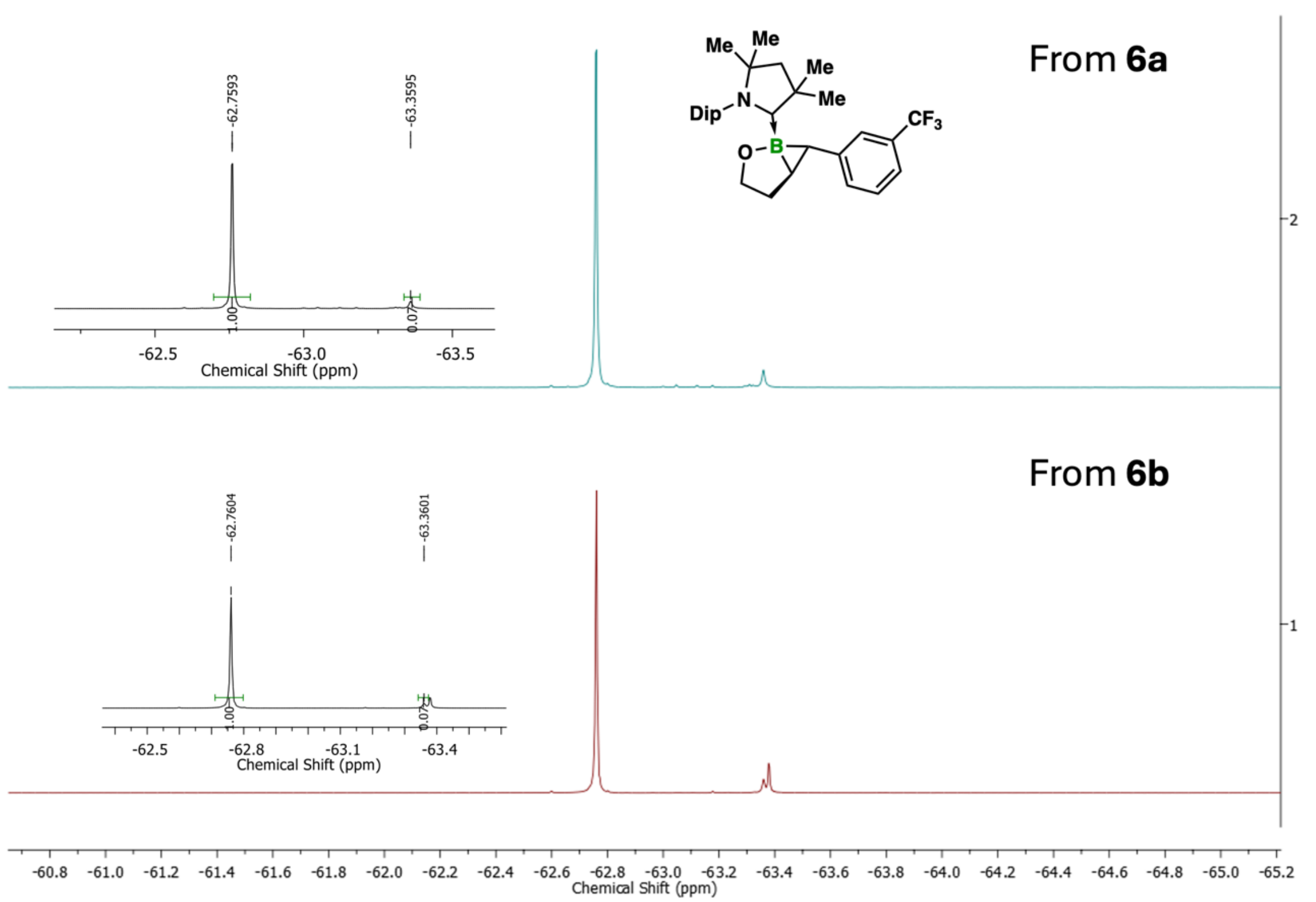

Figure S14b: Stacked ^{19}F NMR (471 MHz, Benzene-d_6) of **7a** from reaction **6a** and **6b**

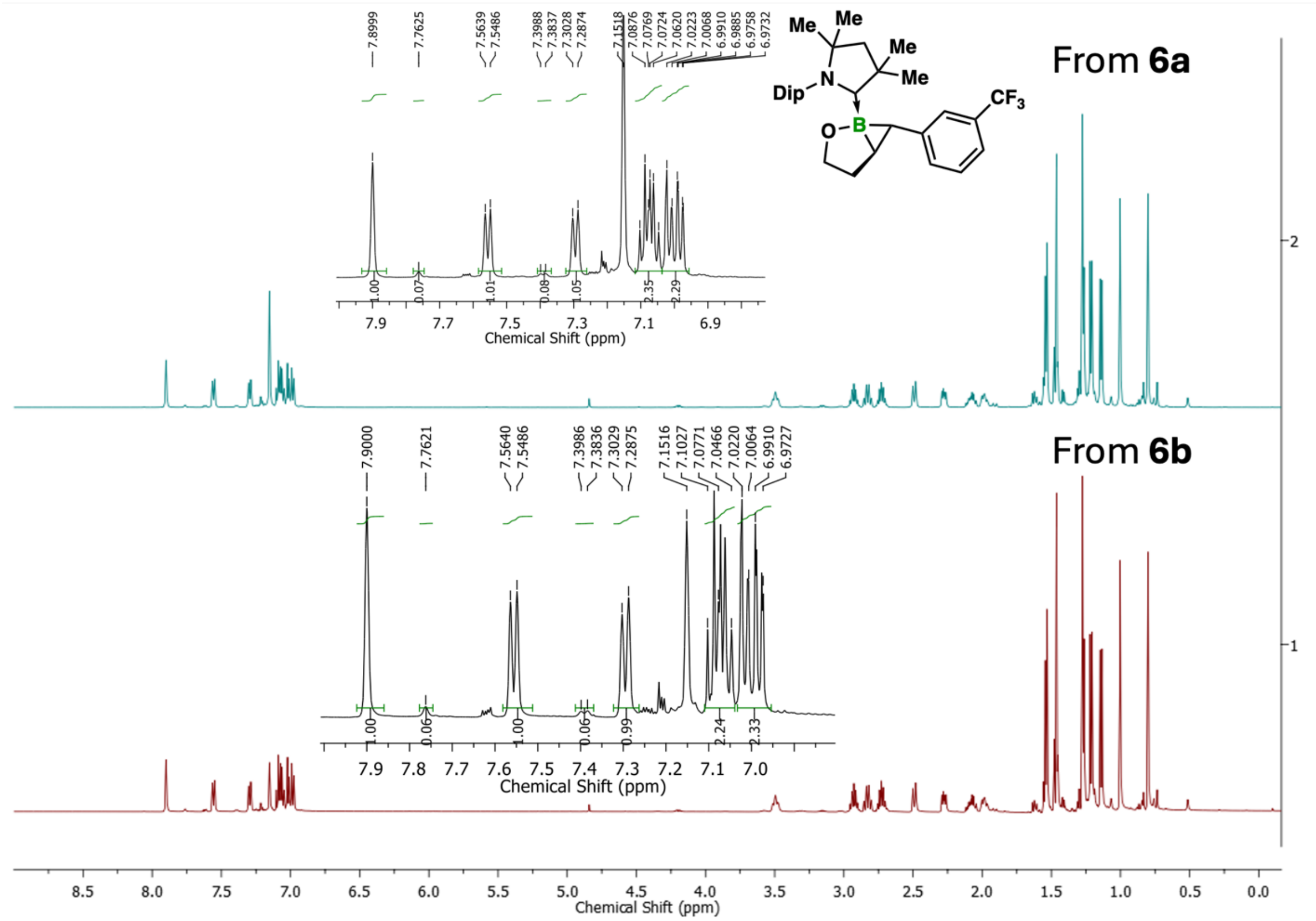

Figure S14c: Stacked ^{1}H NMR (500 MHz, Benzene-d_6) of **7a** from reaction **6a** and **6b**

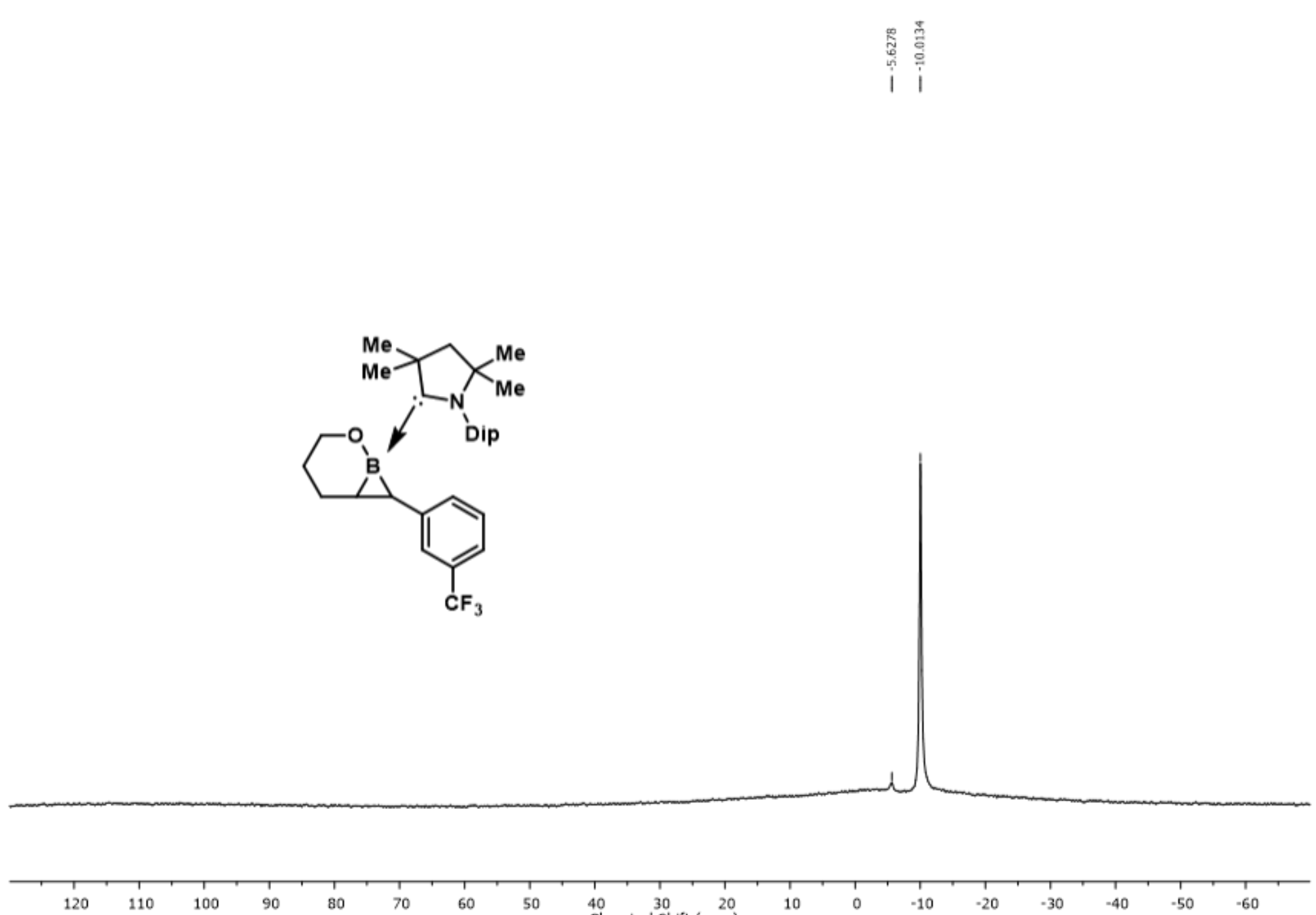

Figure 14d ^{11}B NMR (160 MHz, Benzene-d_6) of **7e** from **6e**

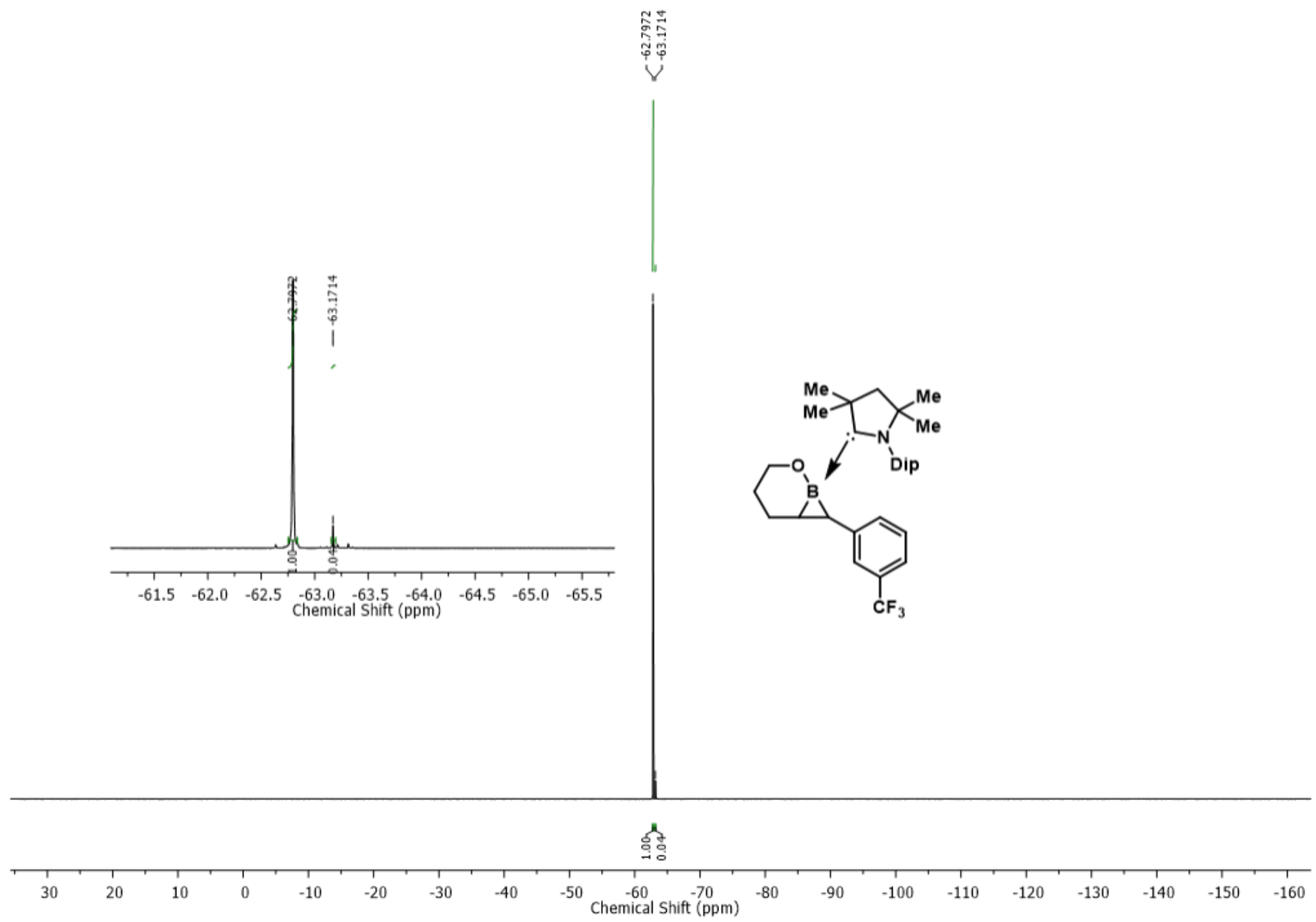

Figure 14e ^{19}F NMR (471 MHz, Benzene-d_6) of **7e** from **6e**

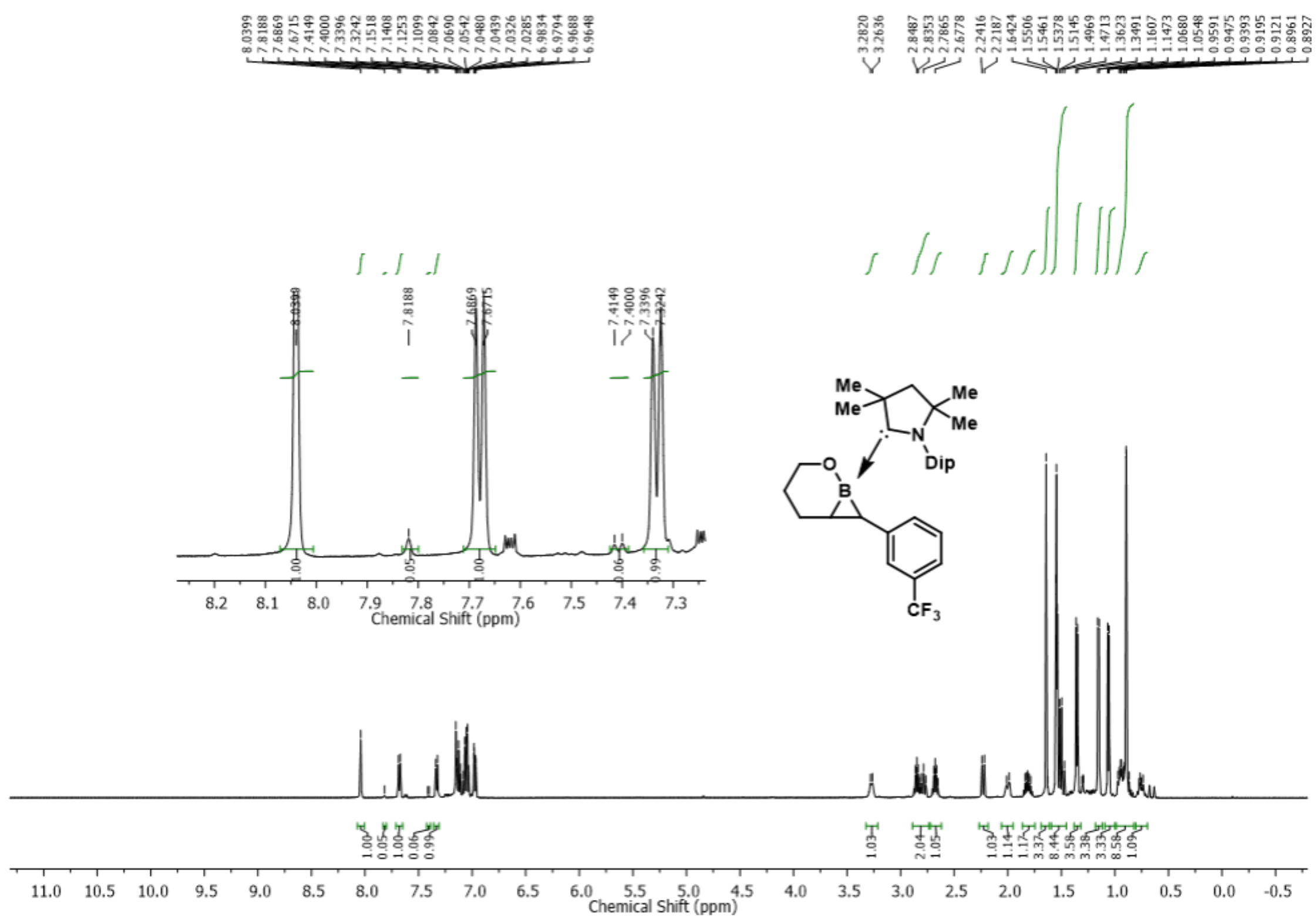

Figure 14f ^{1}H NMR (500 MHz, Benzene-d_6) of **7e** from **6e**

5. NMR and HRMS spectra

^{1}H NMR (300 MHz, Chloroform-*d*) of Compound 1

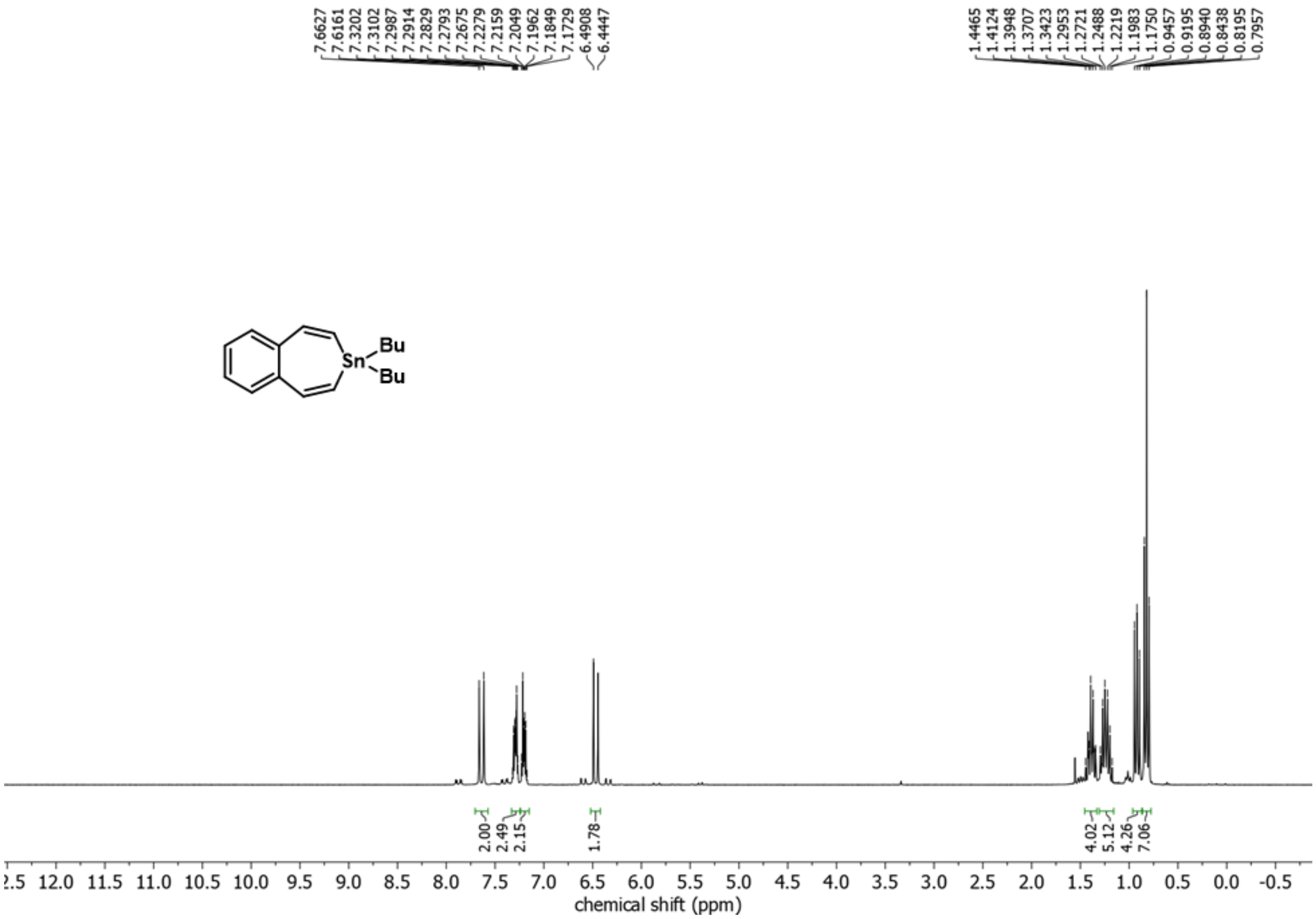

$^{13}C\{^1H\}$ NMR (75 MHz, Chloroform-*d*) of Compound 1

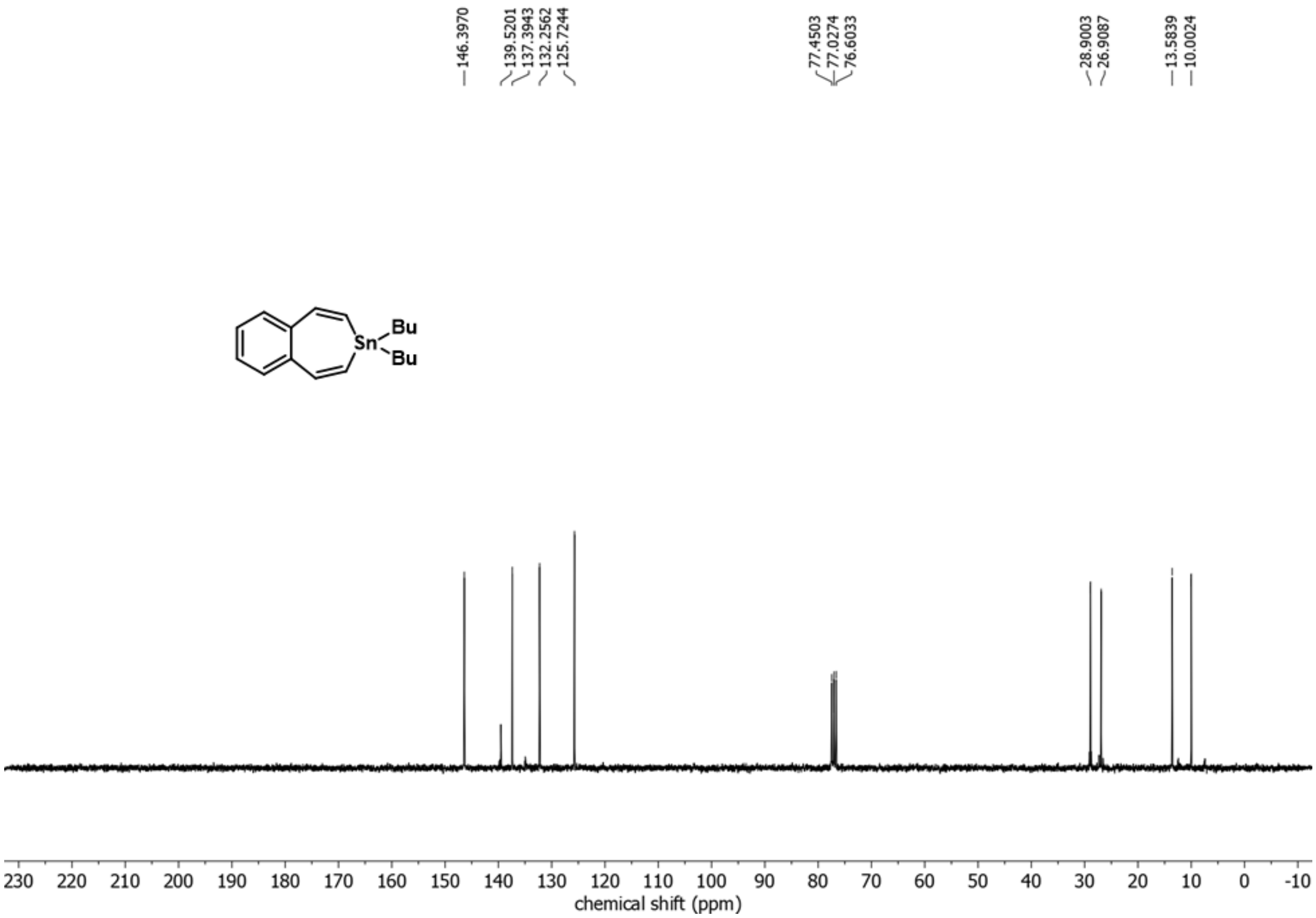

HRMS (ASAP) of Compound 1

Elemental Composition Report

Page 1

Single Mass Analysis
Tolerance = 50.0 PPM / DBE: min = -1.5, max = 500.0
Element prediction: Off
Number of isotope peaks used for i-FIT = 3

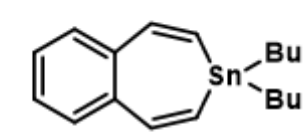

Chemical Formula: $C_{18}H_{26}Sn$
Exact Mass: 362.1057

Monoisotopic Mass, Even Electron Ions
10 formula(e) evaluated with 1 results within limits (up to 50 closest results for each mass)
Elements Used:
C: 18-18 H: 10-30 B: 0-2 Na: 0-1 Sn: 0-1

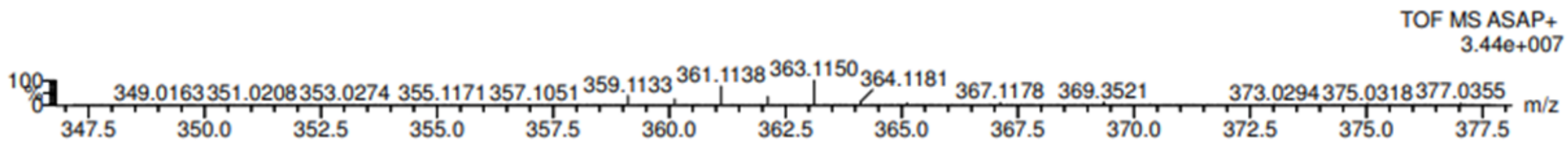

Minimum:				-1.5				
Maximum:		5.0	50.0	500.0				
Mass	Calc. Mass	mDa	PPM	DBE	i-FIT	Norm	Conf(%)	Formula
363.1150	363.1135	1.5	4.1	6.5	1024.3	n/a	n/a	C18 H27 Sn

^{1}H NMR (300 MHz, Chloroform-*d*) of Compound 2

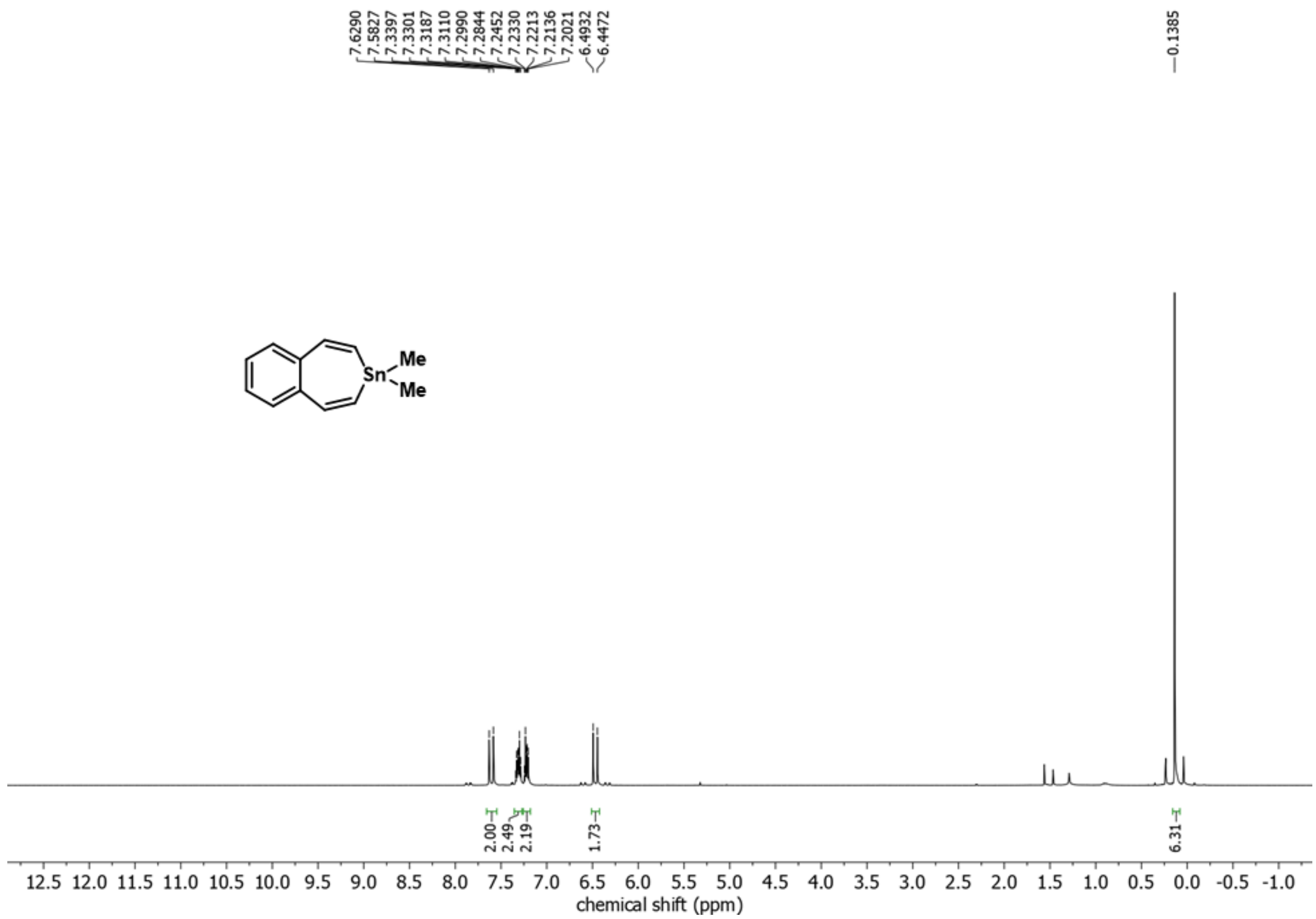

$^{13}C\{^{1}H\}$ NMR (75 MHz, Chloroform-*d*) of Compound 2

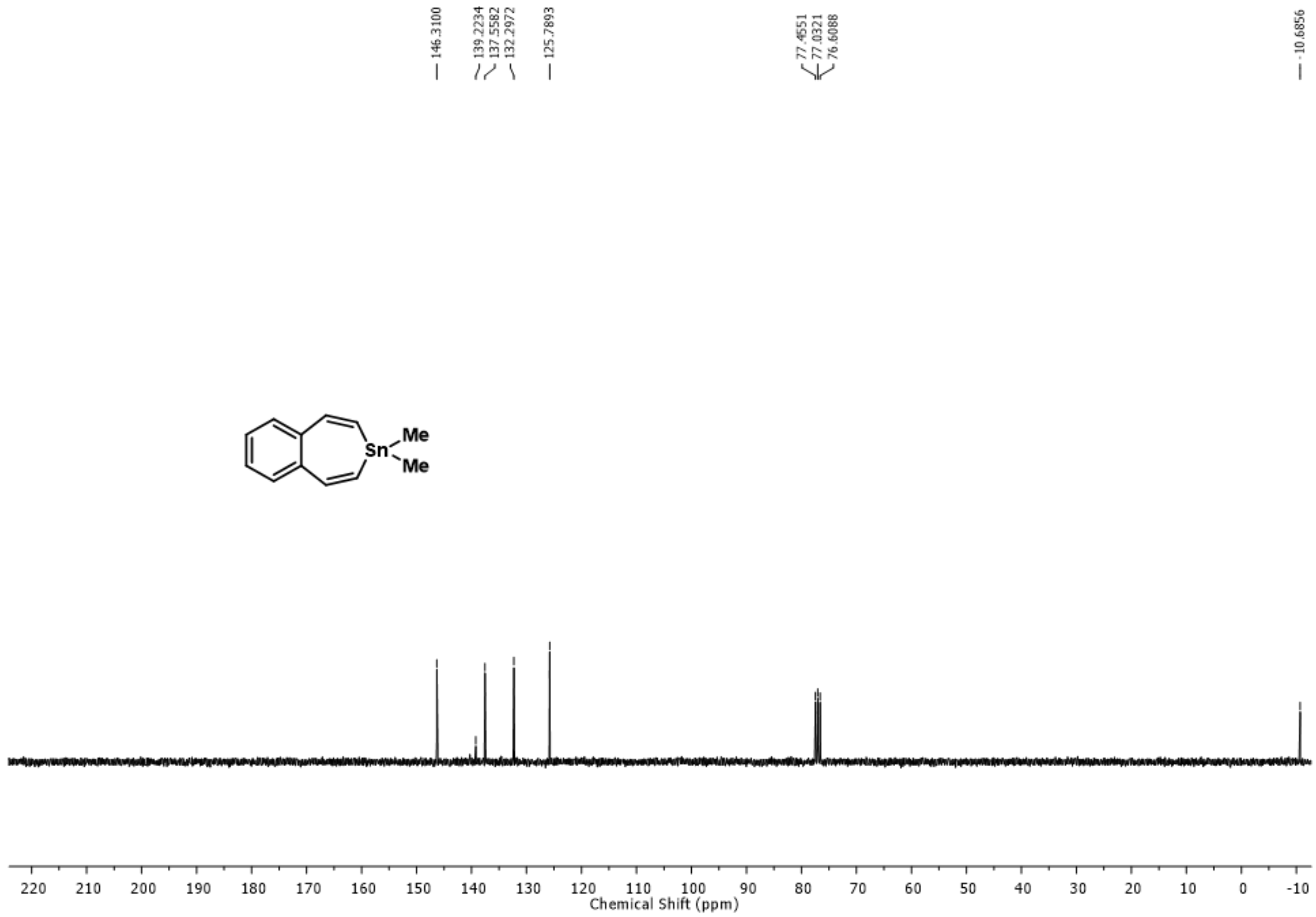

HRMS (ESI) of Compound 2

Elemental Composition Report **Page 1**

Single Mass Analysis
Tolerance = 5.0 PPM / DBE: min = -1.5, max = 500.0
Element prediction: Off
Number of isotope peaks used for i-FIT = 3

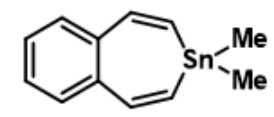

Chemical Formula: $C_{12}H_{14}Sn$
Exact Mass: 278.0118

Monoisotopic Mass, Even Electron Ions
4 formula(e) evaluated with 1 results within limits (up to 50 closest results for each mass)
Elements Used:
C: 12-15 H: 10-23 Na: 0-1 Sn: 0-1

1: TOF MS ES+
3.96e+005

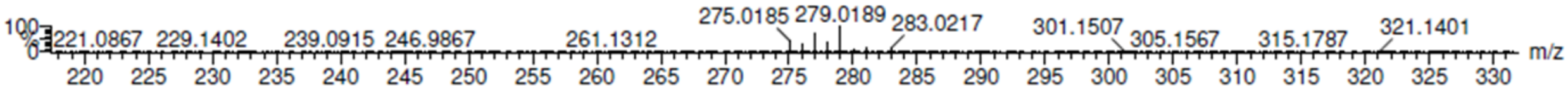

Minimum:				-1.5				
Maximum:		5.0	5.0	500.0				
Mass	Calc. Mass	mDa	PPM	DBE	i-FIT	Norm	Conf(%)	Formula
279.0189	279.0196	-0.7	-2.5	6.5	276.6	n/a	n/a	C12 H15 Sn

^{1}H NMR (400 MHz, Benzene-d_6) of Compound 3

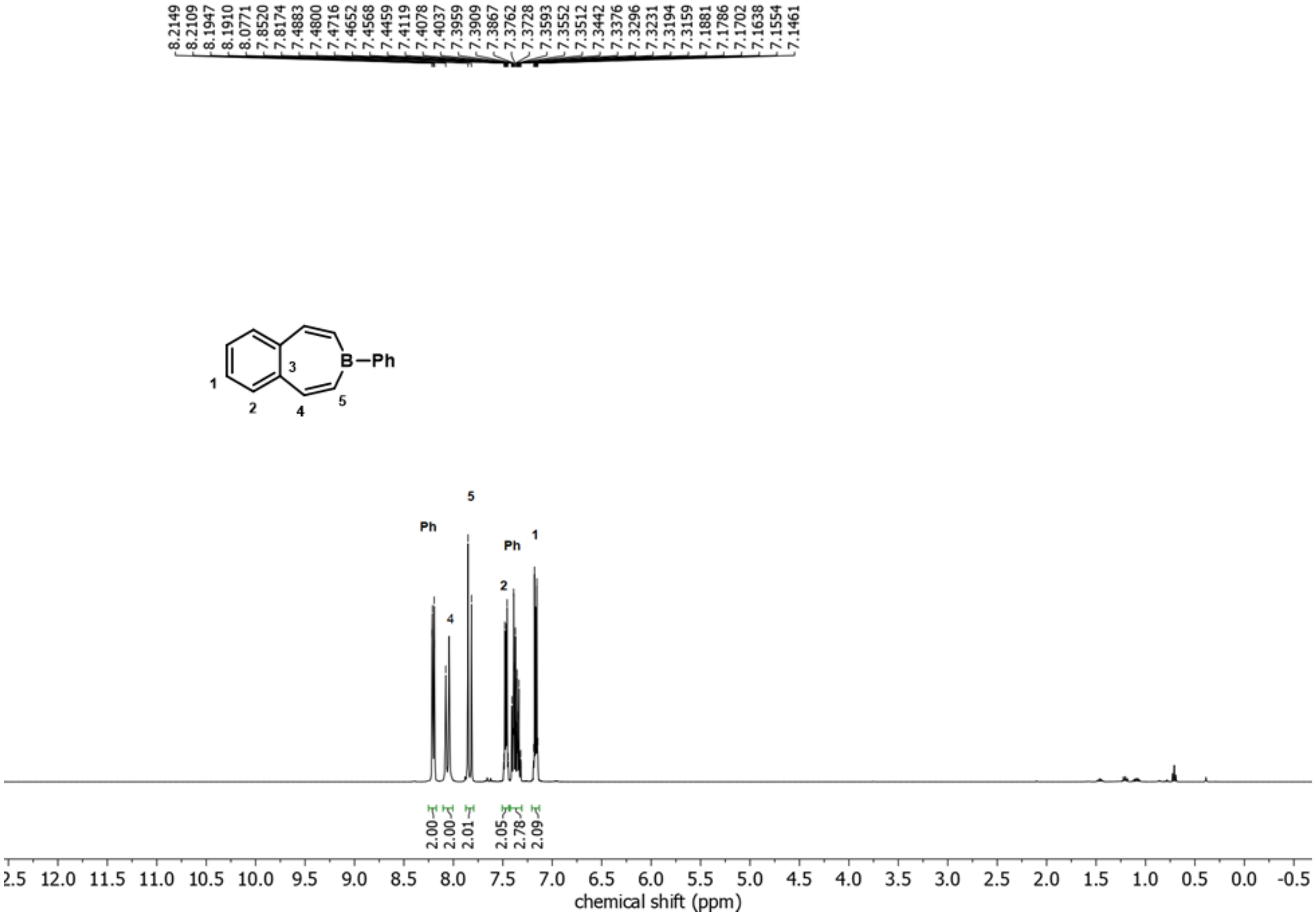

$^{13}C\{^1H\}$ NMR (101 MHz, Benzene-d_6) of Compound 3

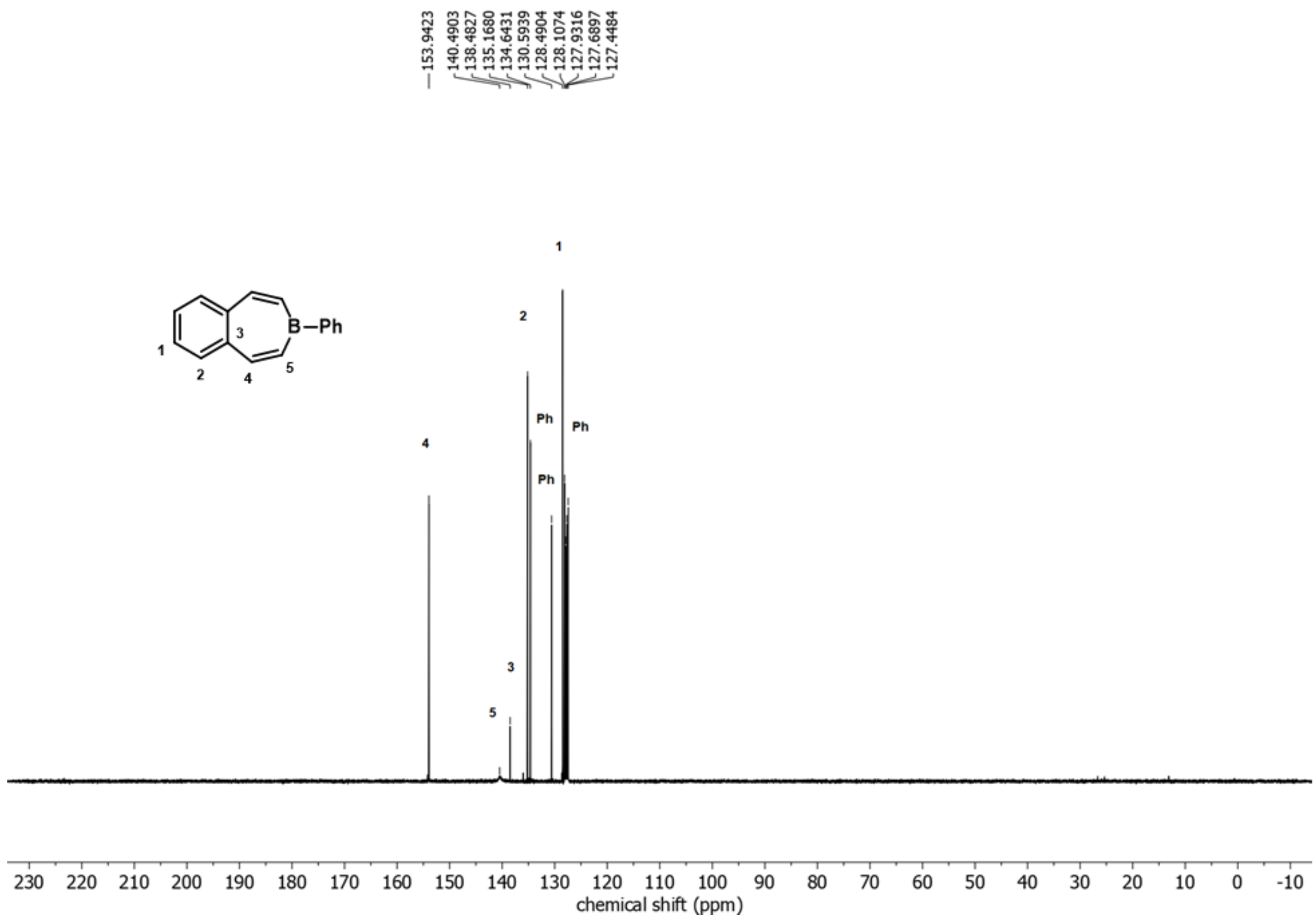

^{11}B NMR (128 MHz, Benzene-d_6) of Compound 3

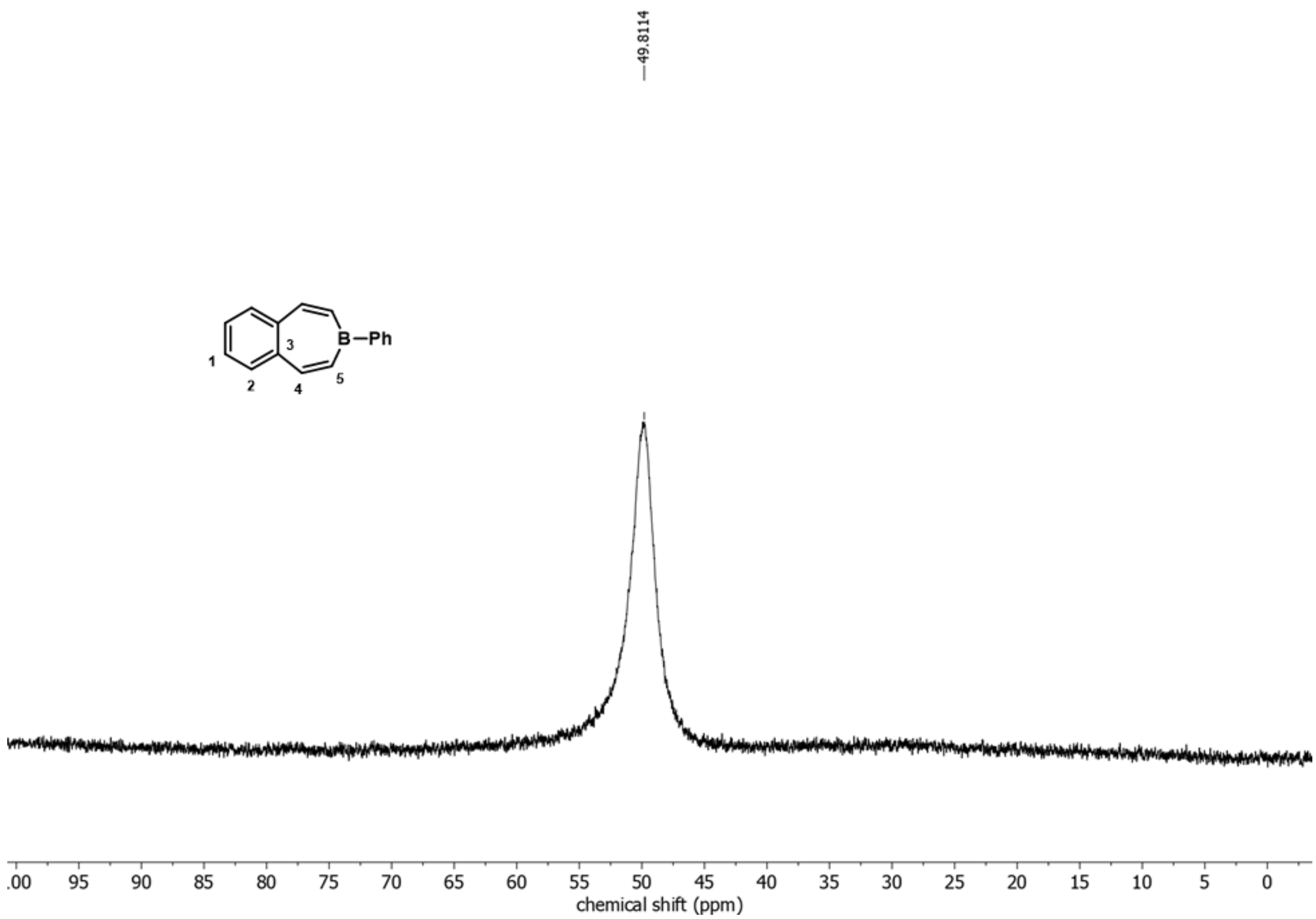

HRMS (ASAP) of Compound 3

Elemental Composition Report **Page 1**

Single Mass Analysis
Tolerance = 50.0 PPM / DBE: min = -1.5, max = 500.0
Element prediction: Off
Number of isotope peaks used for i-FIT = 3

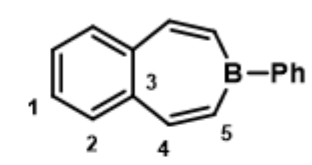

Chemical Formula: $C_{16}H_{13}B$
Exact Mass: 216.1110

Monoisotopic Mass, Even Electron Ions
3 formula(e) evaluated with 1 results within limits (up to 50 closest results for each mass)
Elements Used:
C: 16-16 H: 10-20 B: 0-2 Na: 0-1
DCM

TOF MS ASAP+
2.72e+007

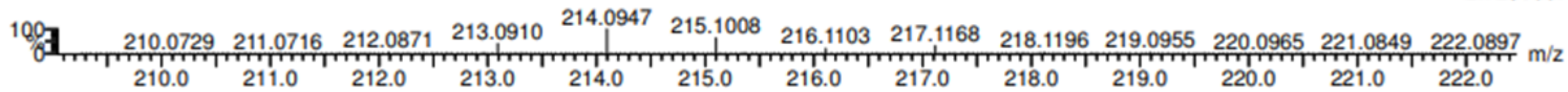

Minimum:				-1.5				
Maximum:		5.0	50.0	500.0				
Mass	Calc. Mass	mDa	PPM	DBE	i-FIT	Norm	Conf(%)	Formula
217.1168	217.1189	-2.1	-9.7	10.5	782.7	n/a	n/a	C16 H14 B

^{1}H NMR (500 MHz, Benzene-d_6) of Compound 4

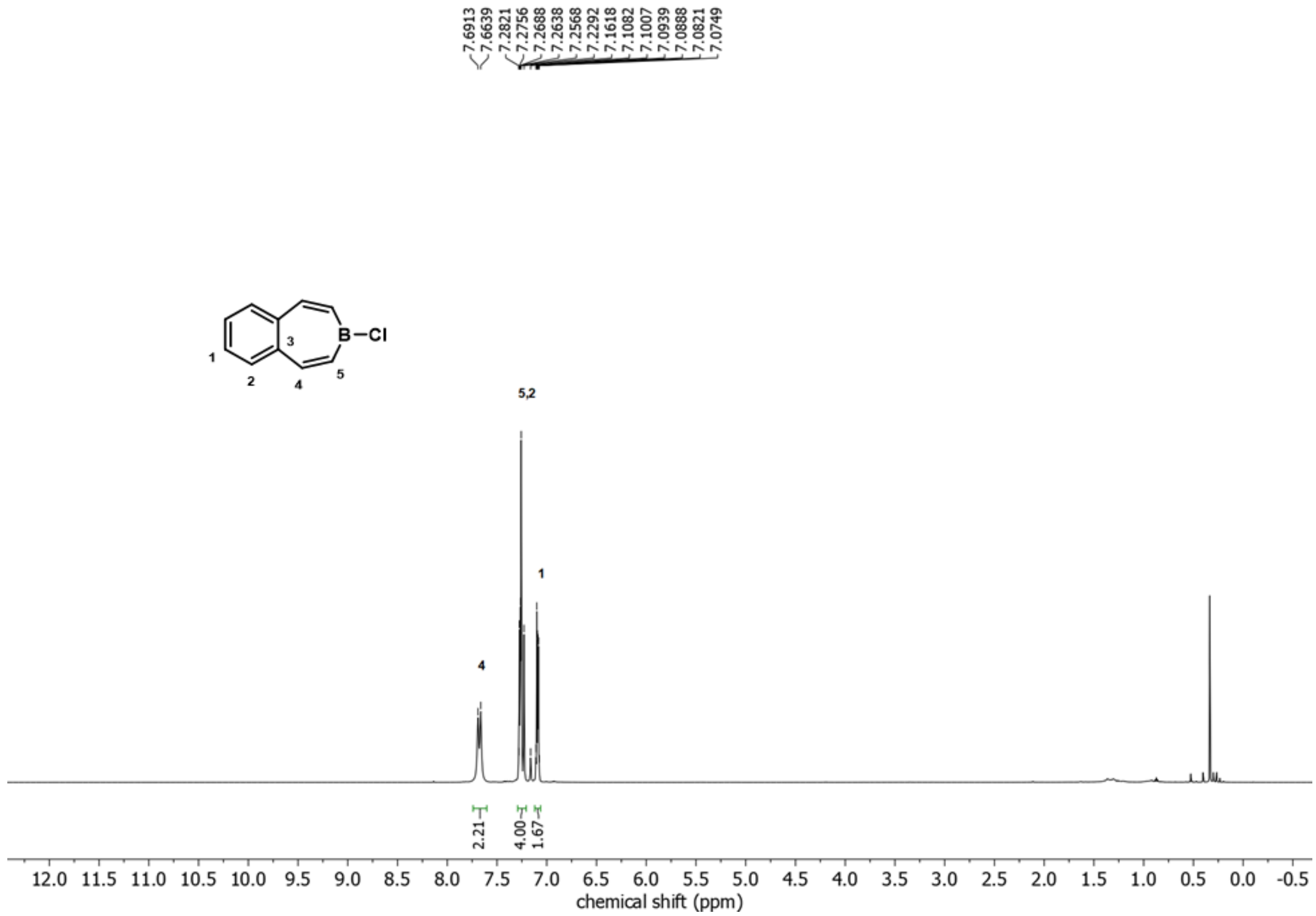

$^{13}C\{^{1}H\}$ NMR (126 MHz, Benzene-d_6) of Compound 4

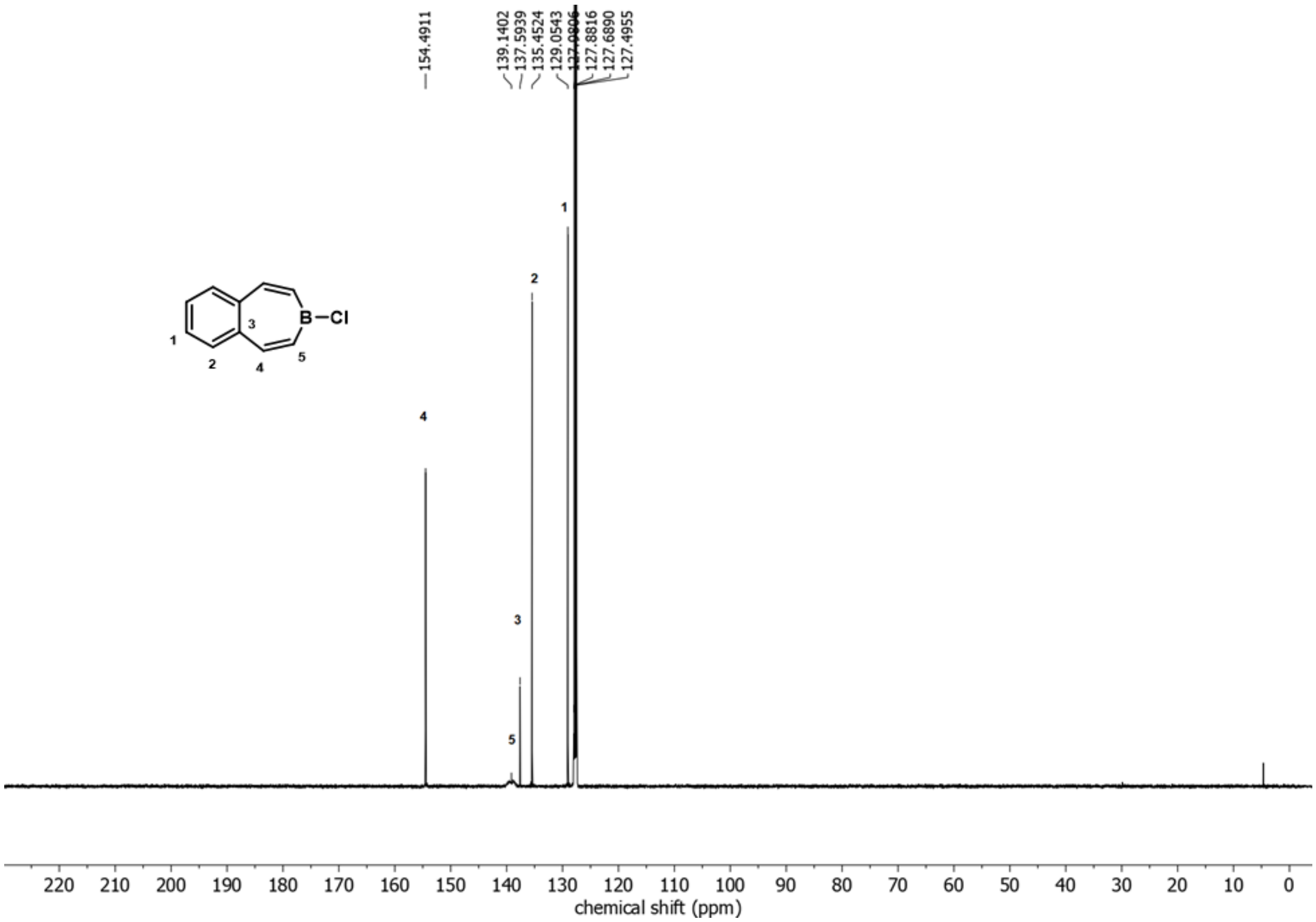

^{11}B NMR (160 MHz, Benzene-d_6) of Compound 4

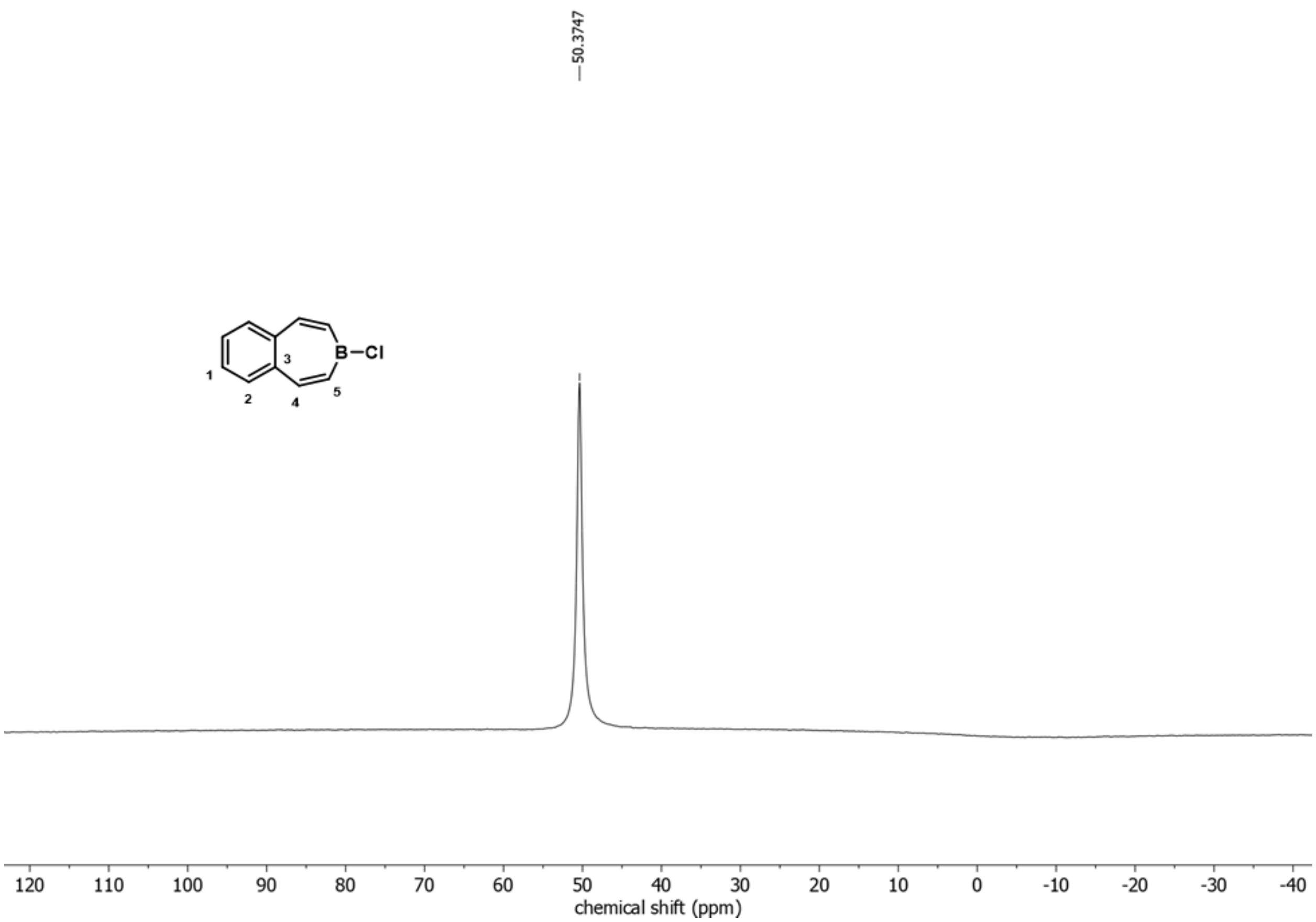

^{1}H NMR (500 MHz, Benzene-d_6) of Compound 3a

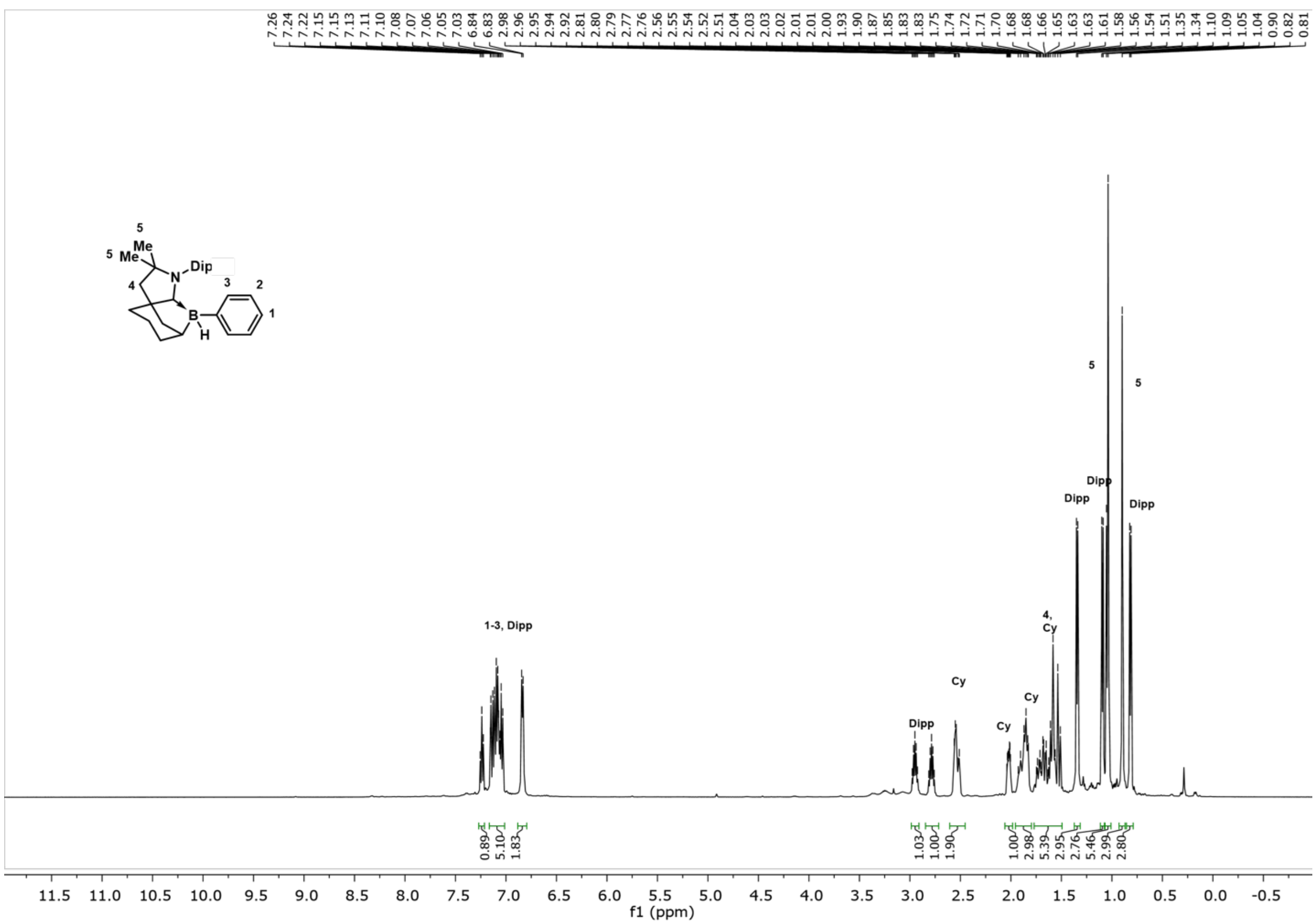

$^{13}C\{^1H\}$ NMR (126 MHz, Benzene-d_6) of Compound 3a

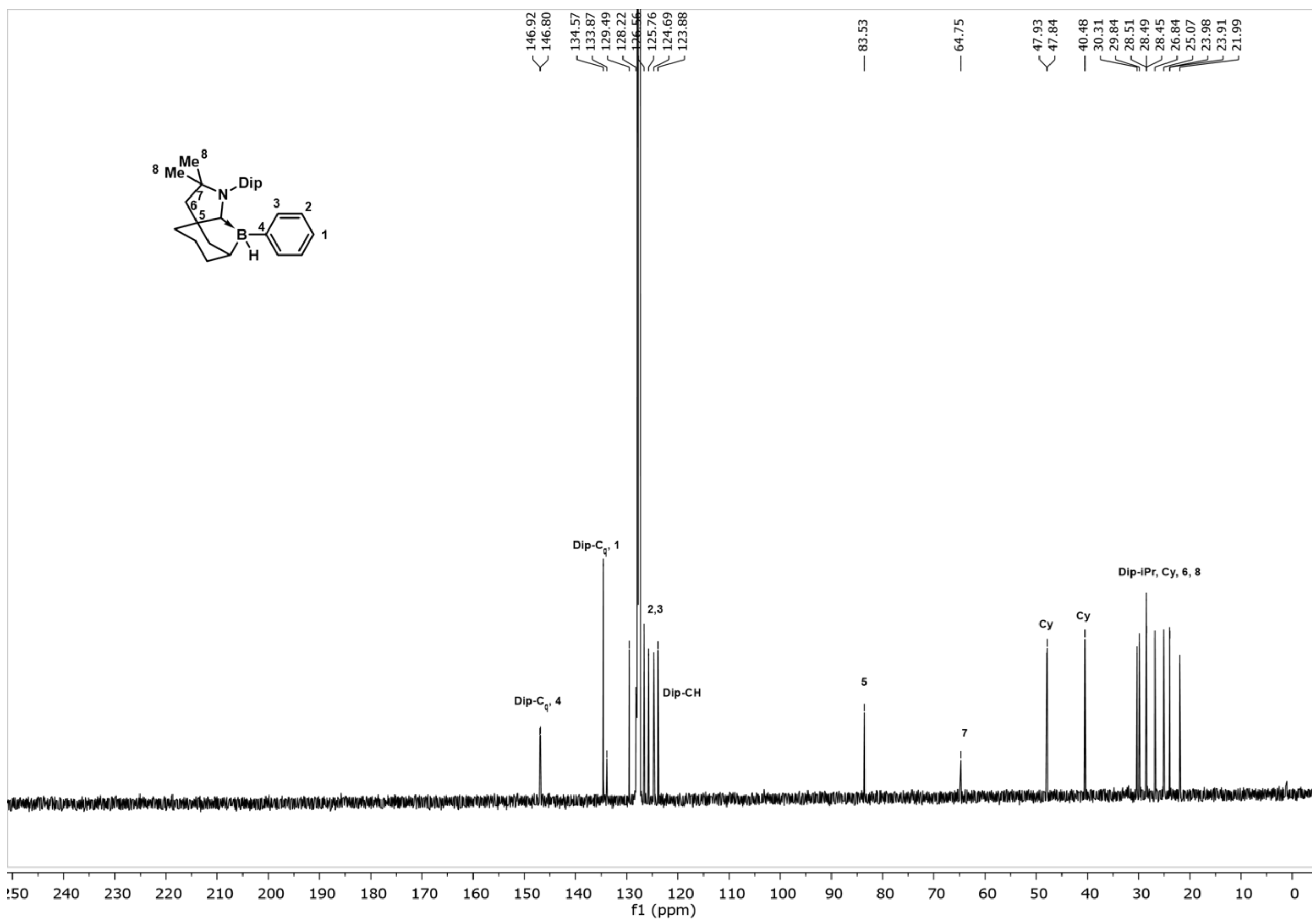

^{11}B NMR (160 MHz, Benzene-d_6) of Compound 3a

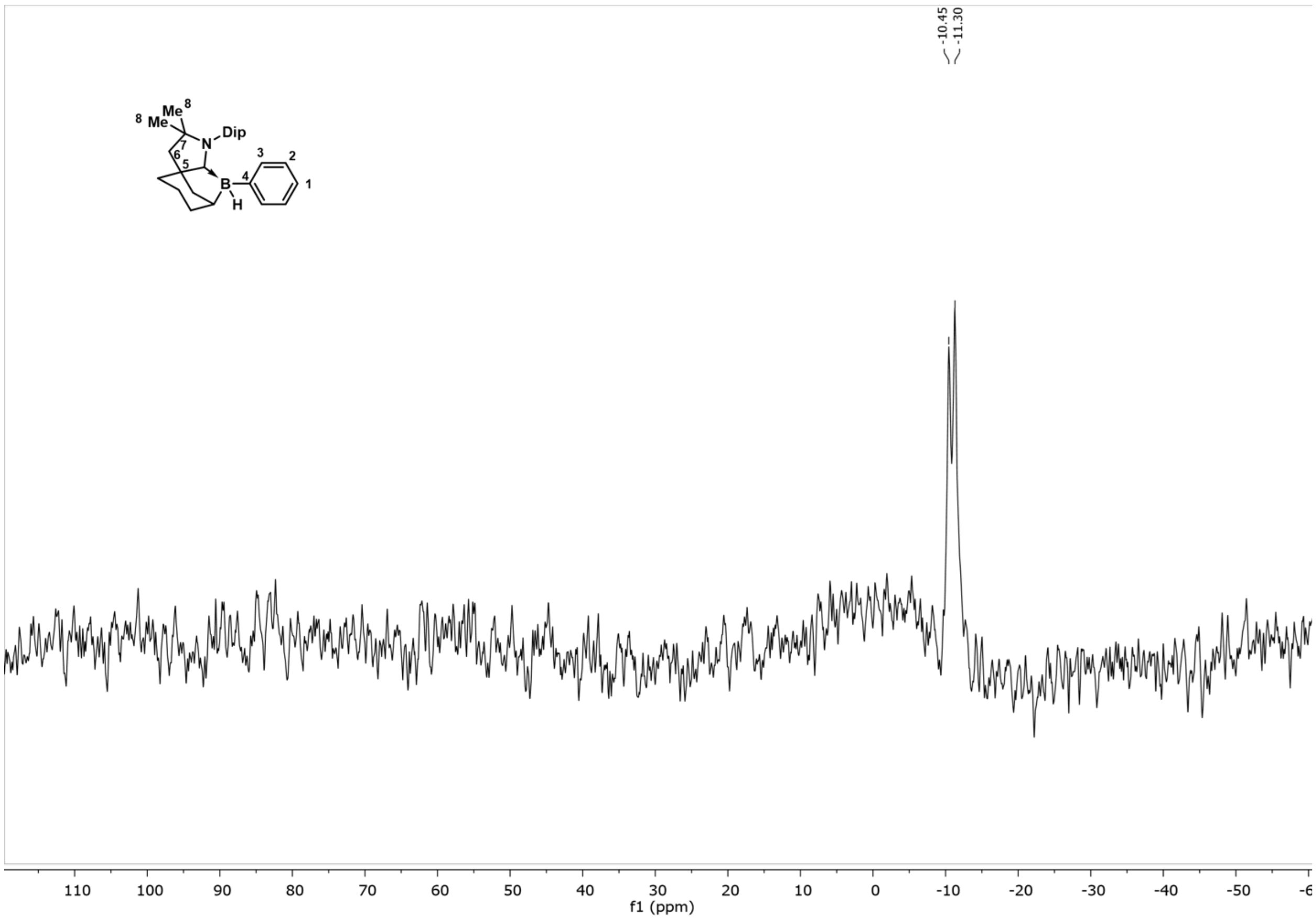

HRMS (ASAP) of Compound 3a

Elemental Composition Report **Page 1**

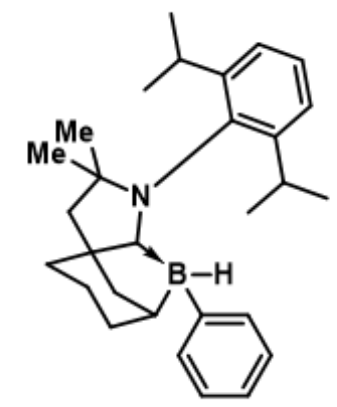

Chemical Formula: $C_{29}H_{40}BN$
Exact Mass: 413.3248

Single Mass Analysis
Tolerance = 50.0 PPM / DBE: min = -1.5, max = 500.0
Element prediction: Off
Number of isotope peaks used for i-FIT = 3

Monoisotopic Mass, Even Electron Ions
5 formula(e) evaluated with 1 results within limits (up to 50 closest results for each mass)
Elements Used:
C: 29-29 H: 30-40 B: 0-2 N: 1-3 Na: 0-1
DCM

TOF MS ASAP+
1.24e+007

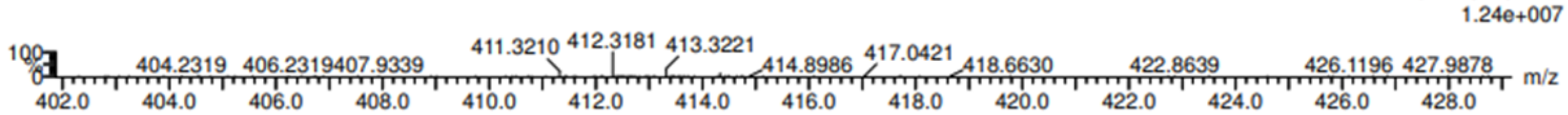

Minimum:				-1.5				
Maximum:		5.0	50.0	500.0				
Mass	Calc. Mass	mDa	PPM	DBE	i-FIT	Norm	Conf(%)	Formula
412.3181	412.3176	0.5	1.2	11.5	358.9	n/a	n/a	C29 H39 B N

^{1}H NMR (400 MHz, Benzene-d_6) of Compound 3b

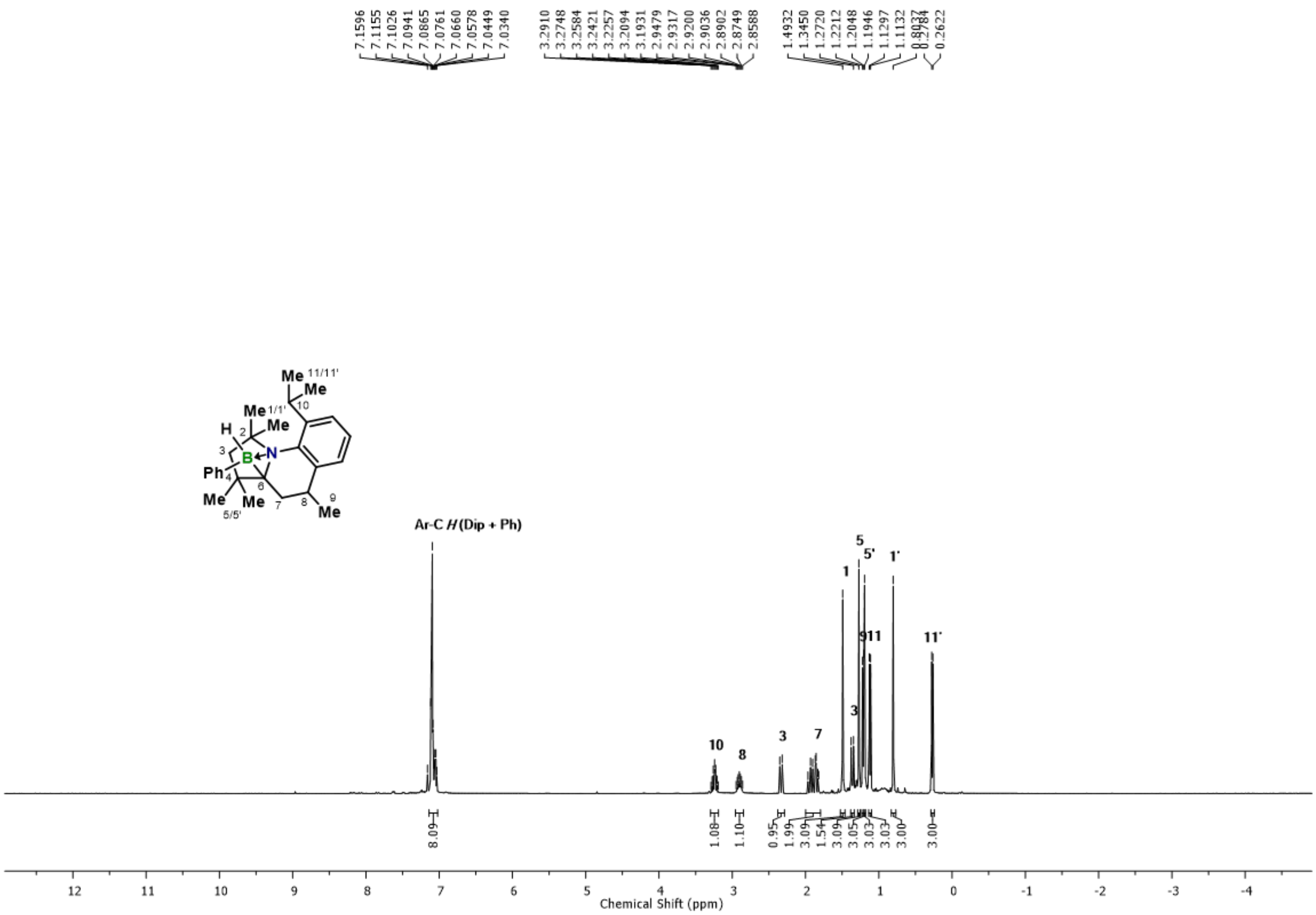

$^{13}C\{^1H\}$ NMR (101 MHz, Benzene-d_6) of Compound 3b

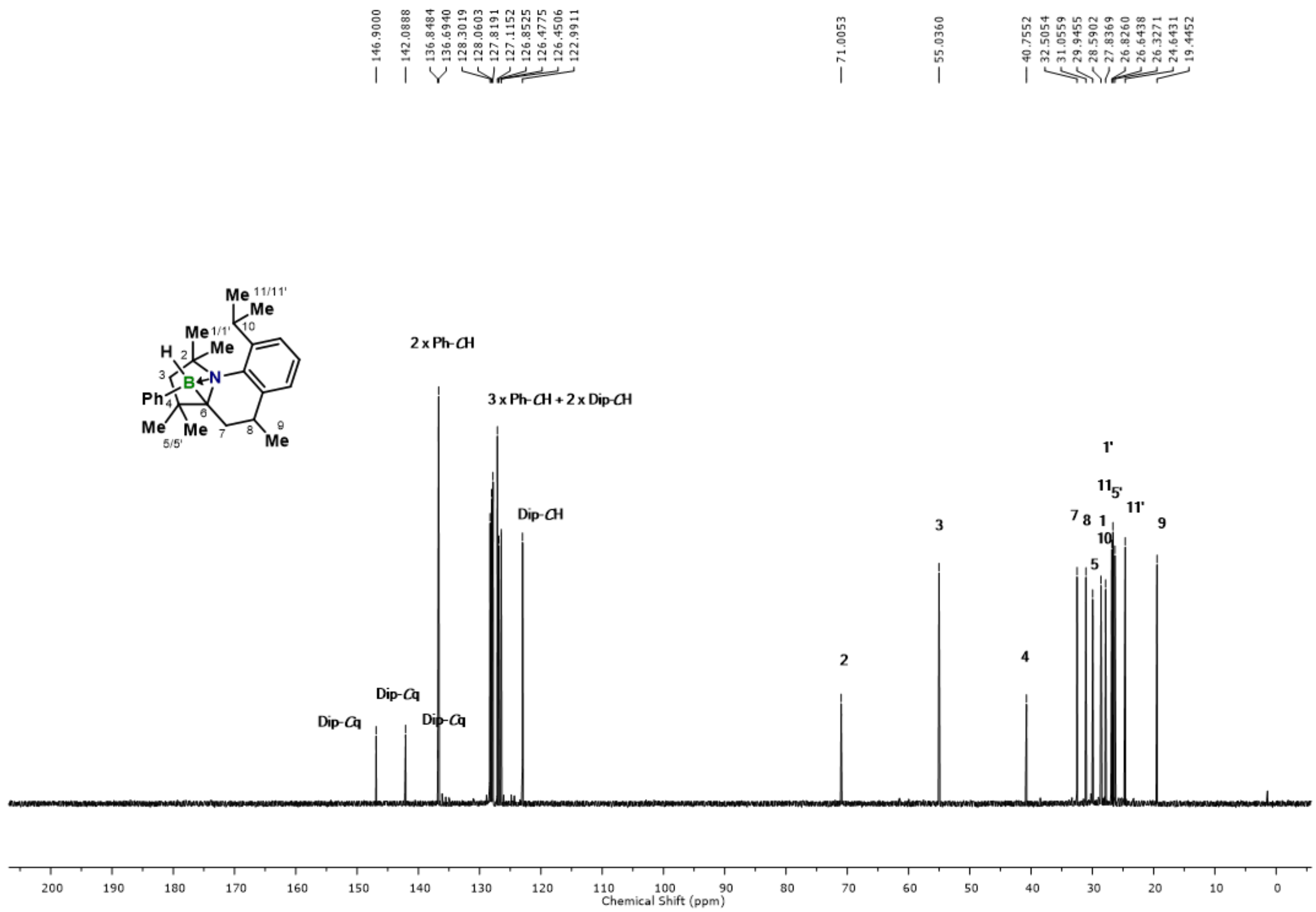

^{11}B NMR (128 MHz, Benzene-d_6) of Compound 3b

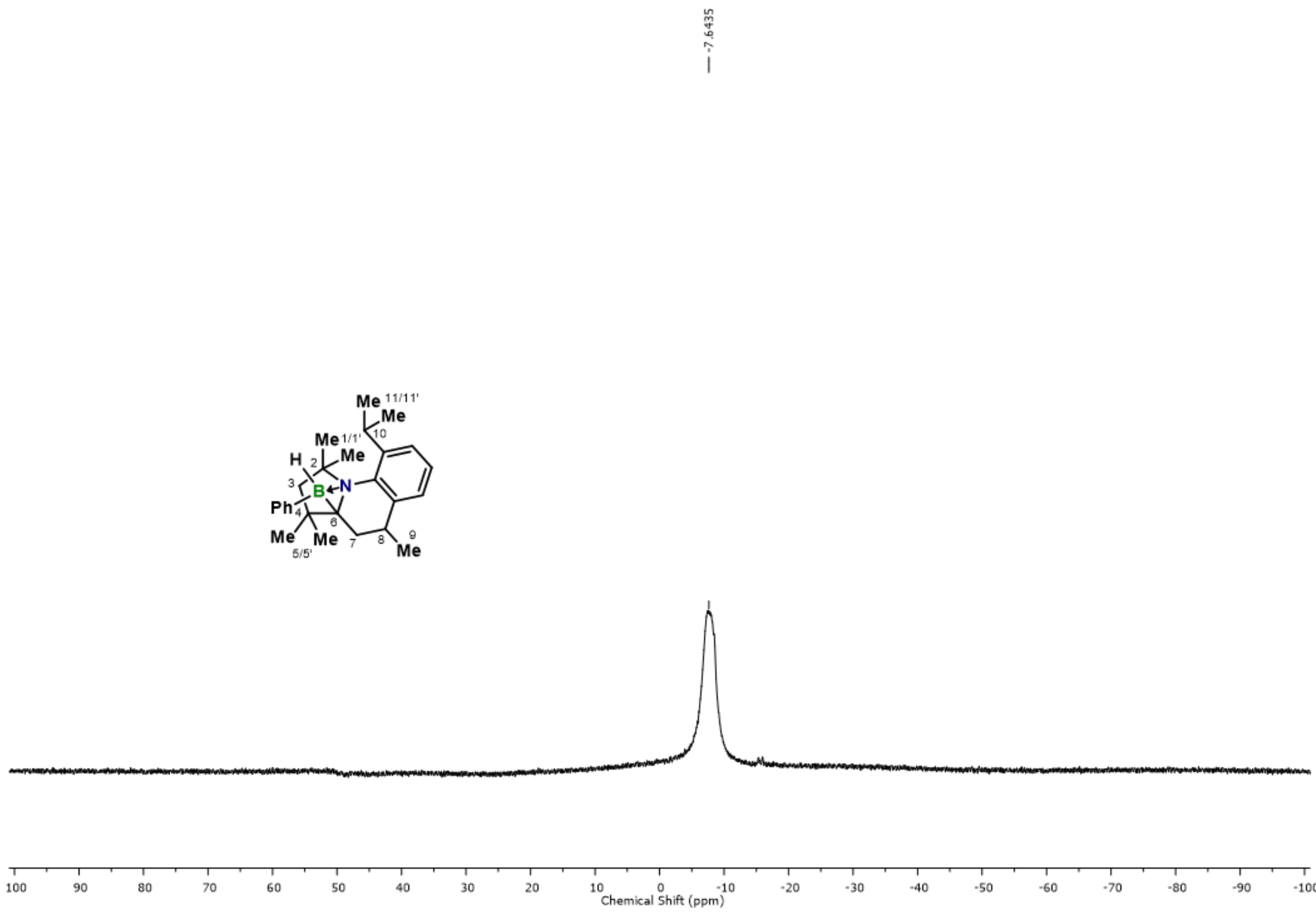

HRMS (ASAP) of Compound 3b

Elemental Composition Report **Page 1**

Single Mass Analysis
Tolerance = 5.0 PPM / DBE: min = -1.5, max = 500.0
Element prediction: Off
Number of isotope peaks used for i-FIT = 3

Monoisotopic Mass, Even Electron Ions
1 formula(e) evaluated with 1 results within limits (up to 50 closest results for each mass)
Elements Used:
C: 26-26 H: 30-40 B: 0-2 N: 1-1 Na: 0-1
pentane

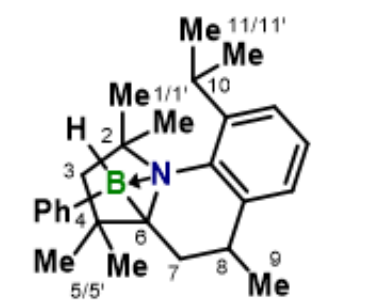

Chemical Formula: $C_{26}H_{36}BN$
Exact Mass: 373.2941

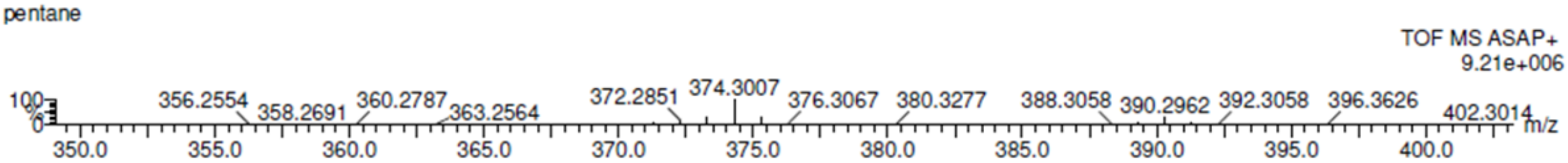

Minimum:				-1.5				
Maximum:		5.0	5.0	500.0				
Mass	Calc. Mass	mDa	PPM	DBE	i-FIT	Norm	Conf(%)	Formula
374.3007	374.3019	-1.2	-3.2	9.5	638.7	n/a	n/a	C26 H37 B N

^{1}H NMR (500 MHz, Chloroform-*d*) of Compound 5a

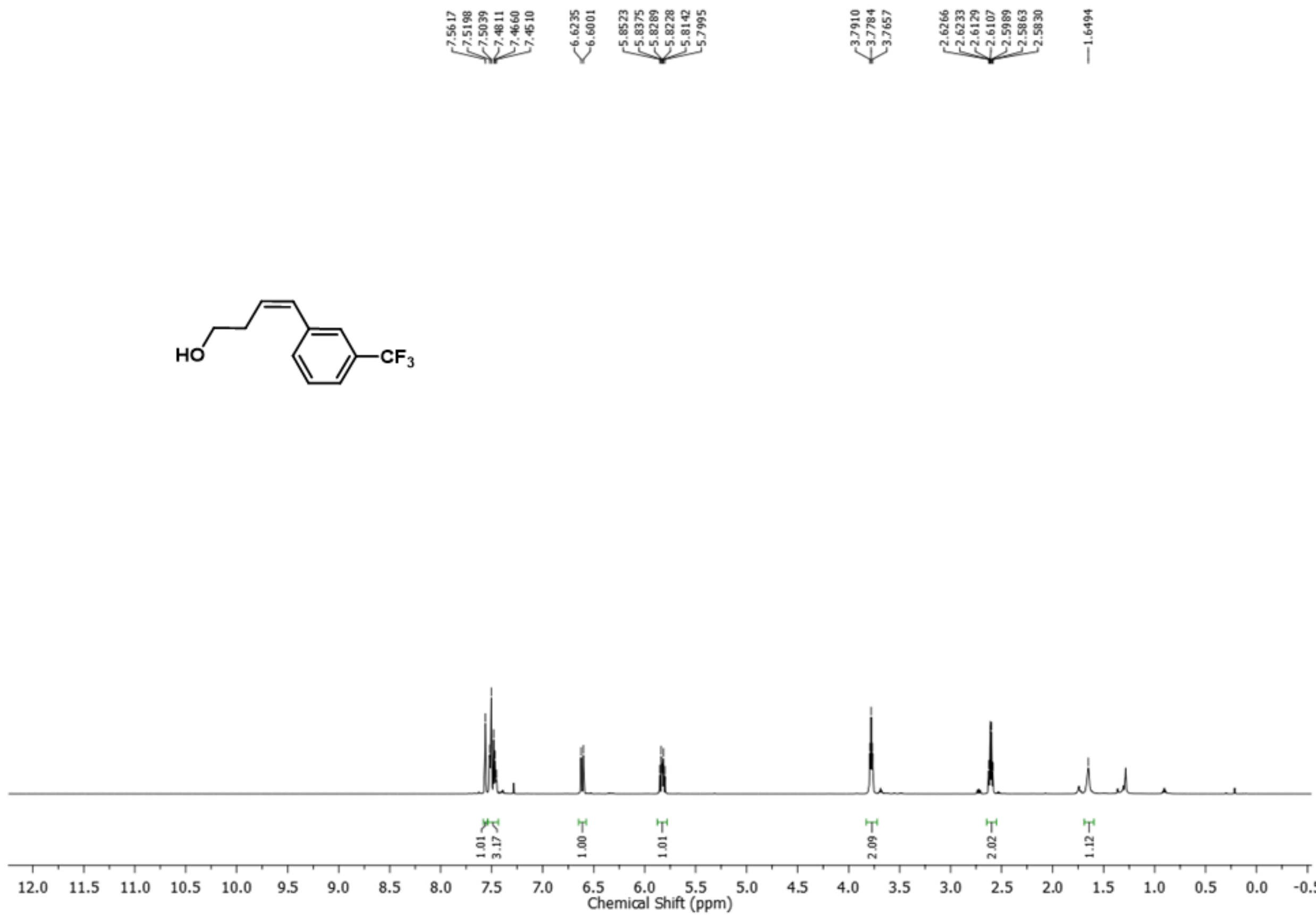

$^{13}C\{^{1}H\}$ NMR (126 MHz, Chloroform-*d*) of Compound 5a

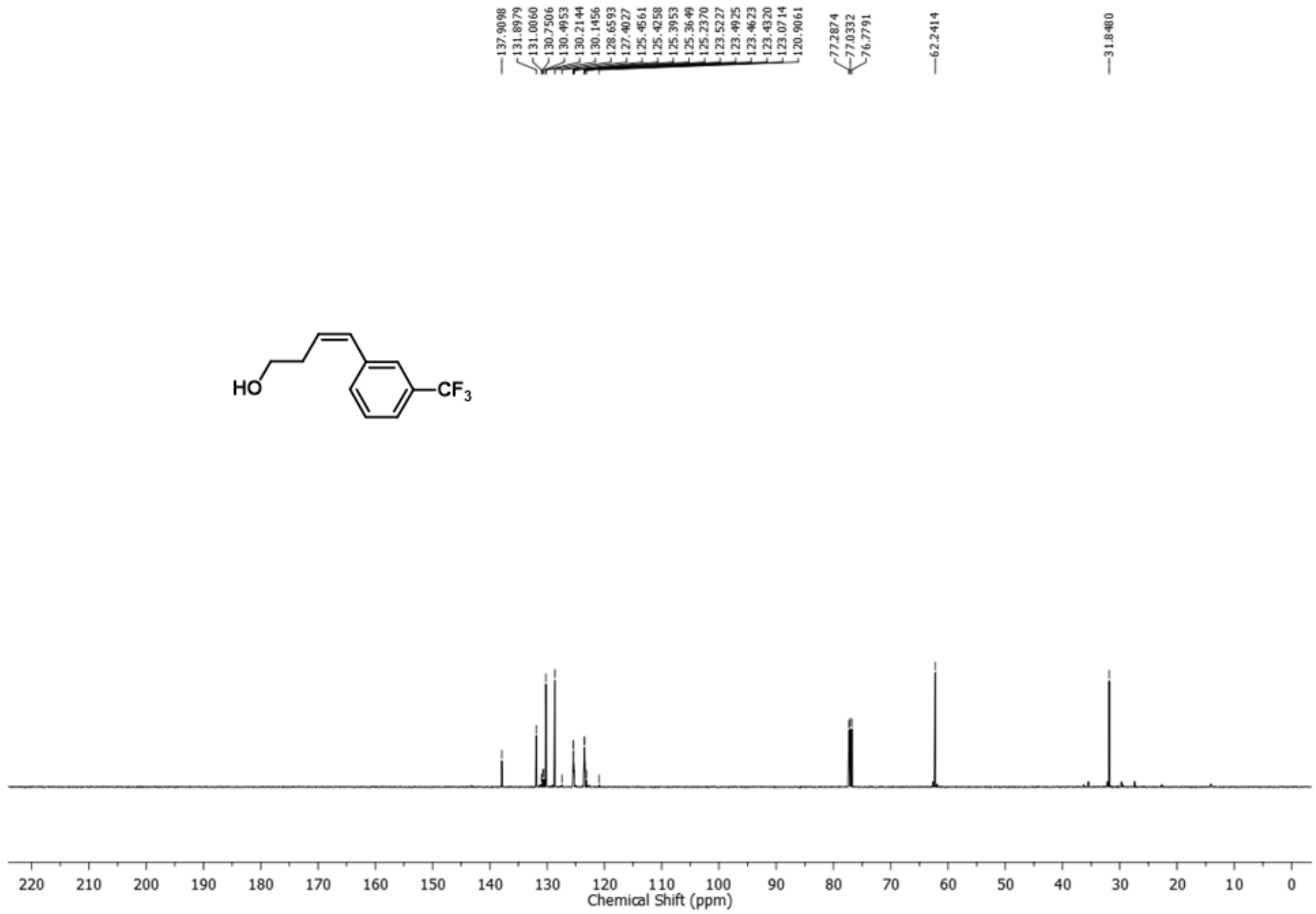

^{19}F NMR (471 MHz, Chloroform-*d*) of Compound 5a

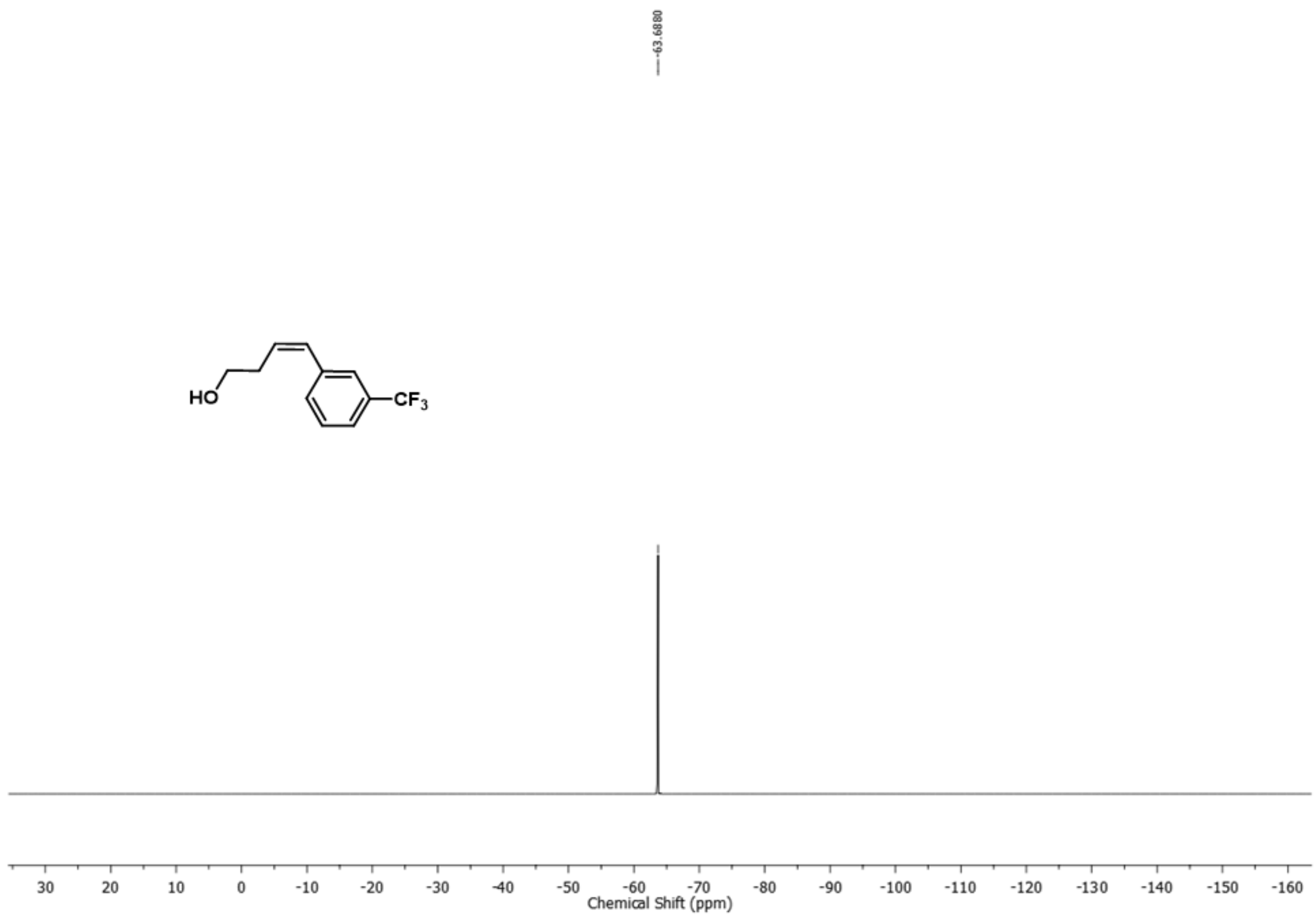

HRMS (ESI) of Compound 5a

Elemental Composition Report — **Page 1**

Single Mass Analysis
Tolerance = 5.0 PPM / DBE: min = -1.5, max = 500.0
Element prediction: Off
Number of isotope peaks used for i-FIT = 3

Monoisotopic Mass, Even Electron Ions
8 formula(e) evaluated with 1 results within limits (up to 50 closest results for each mass)
Elements Used:
C: 11-12 H: 8-20 Na: 0-1 F: 3-6

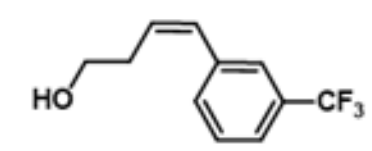

Chemical Formula: $C_{11}H_{11}F_3O$
Exact Mass: 216.0762

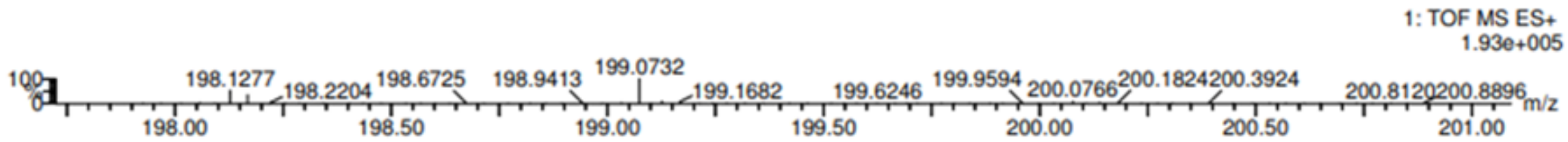

Minimum:				-1.5				
Maximum:		5.0	5.0	500.0				
Mass	Calc. Mass	mDa	PPM	DBE	i-FIT	Norm	Conf(%)	Formula
199.0732	199.0735	-0.3	-1.5	5.5	494.1	n/a	n/a	C11 H10 F3

^{1}H NMR (500 MHz, Chloroform-*d*) of Compound 5f

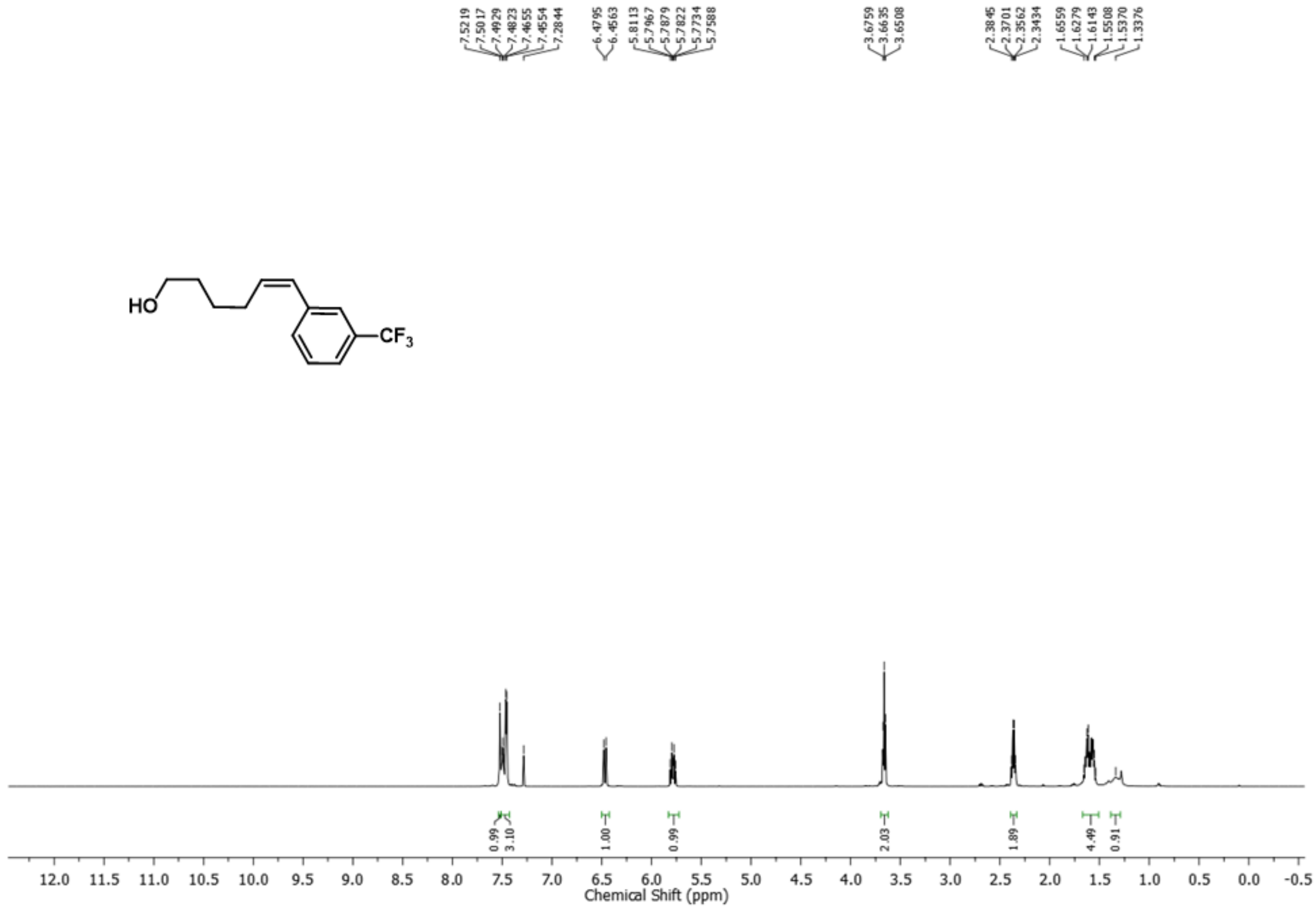

$^{13}C\{^{1}H\}$ NMR (126 MHz, Chloroform-*d*) of Compound 5f

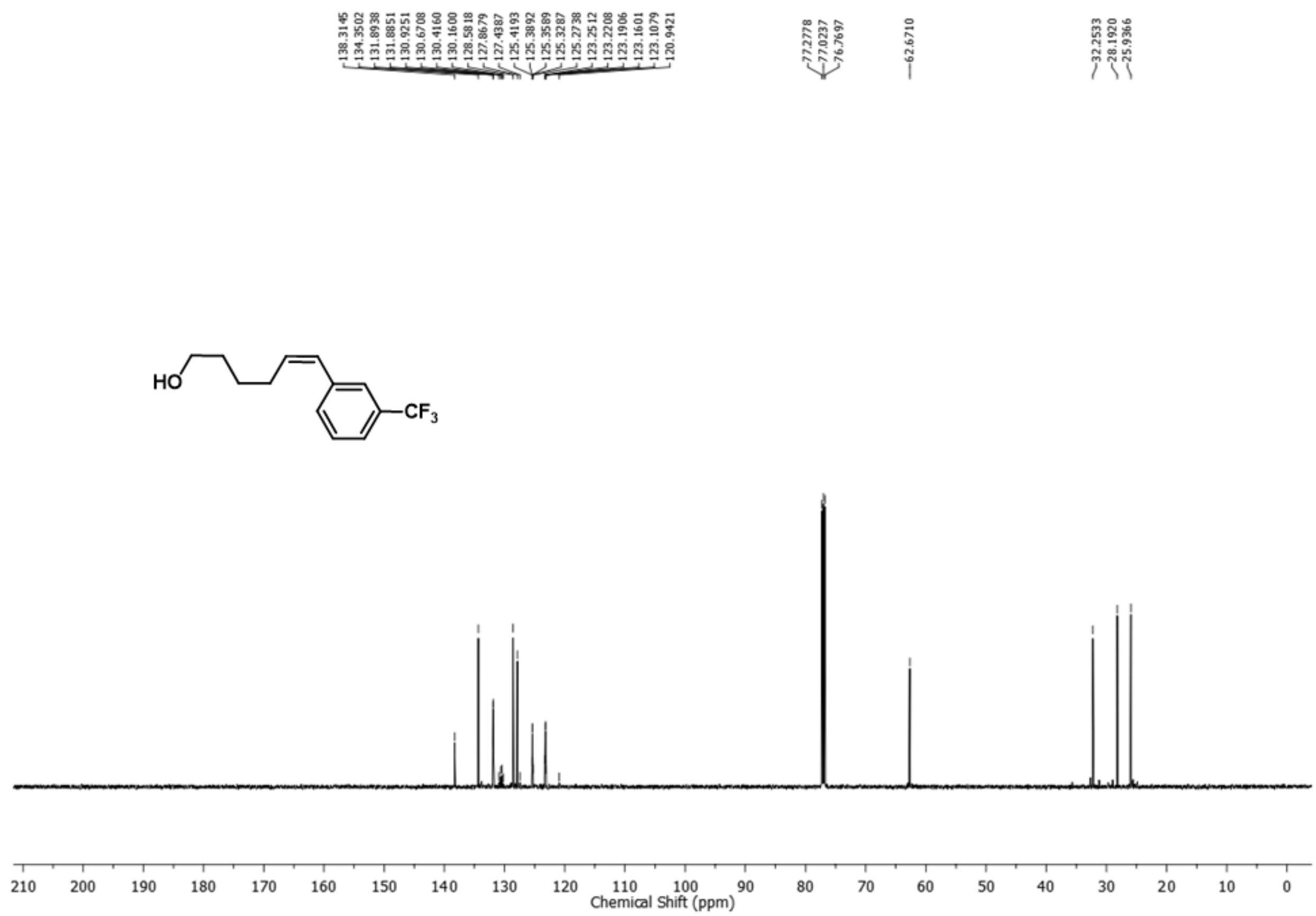

^{19}F NMR (471 MHz, Chloroform-*d*) of Compound 5f

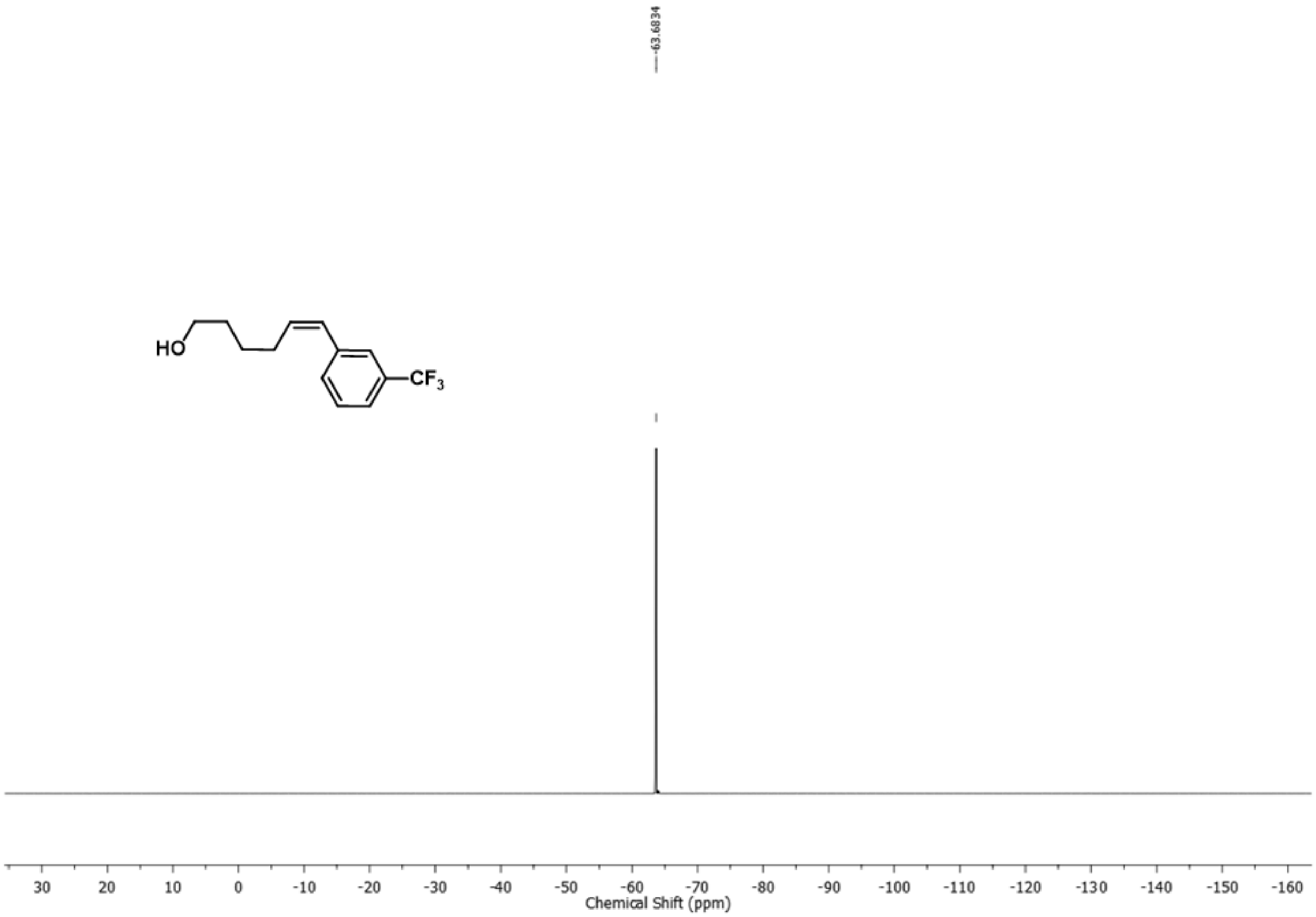

GC/MS(EI) of Compound 5f

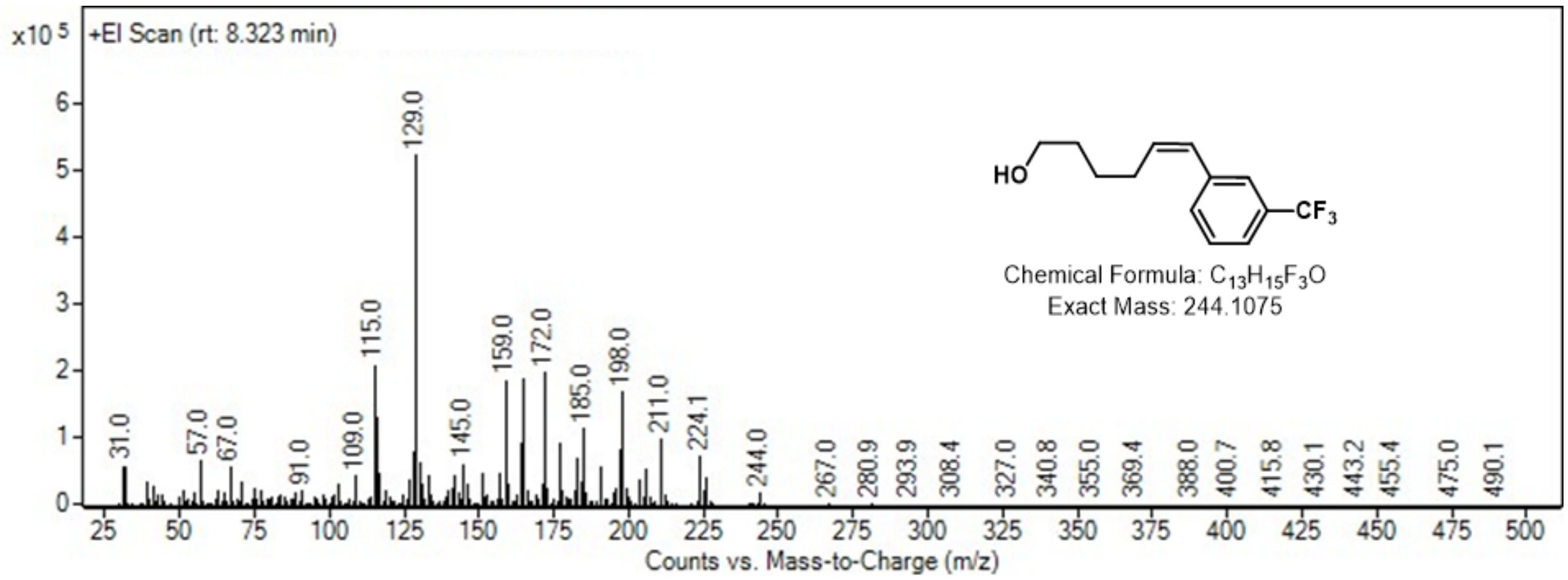

^{1}H NMR (300 MHz, Chloroform-*d*) of Compound 5g

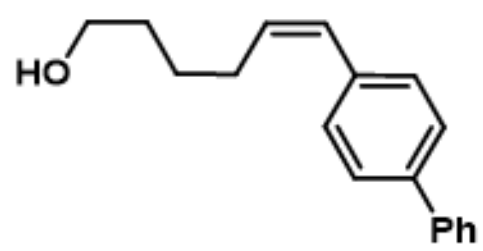

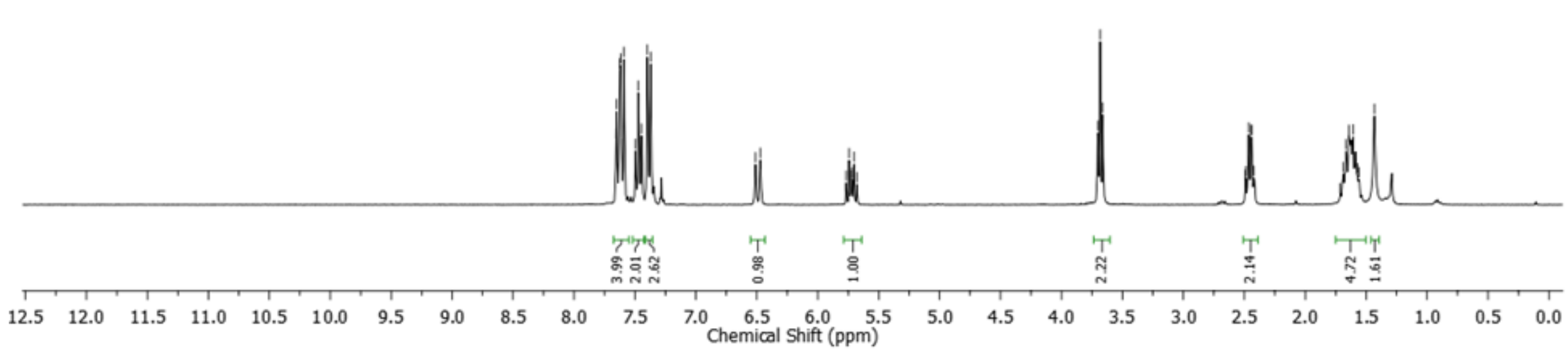

$^{13}C\{^{1}H\}$ NMR (75 MHz, Chloroform-*d*) of Compound 5g

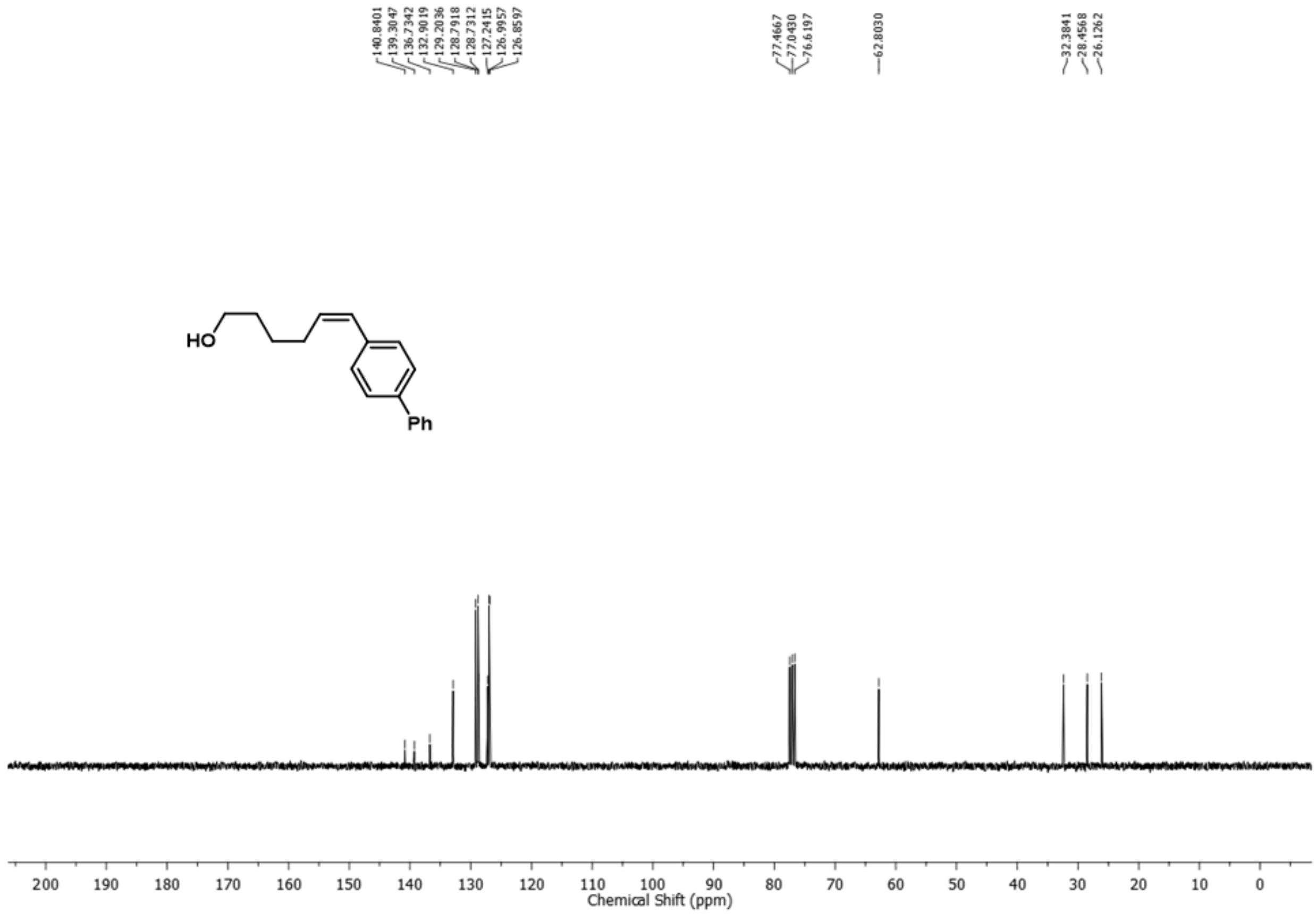

HRMS (ESI) of Compound 5g

Elemental Composition Report

Single Mass Analysis
Tolerance = 5.0 PPM / DBE: min = -1.5, max = 500.0
Element prediction: Off
Number of isotope peaks used for i-FIT = 3

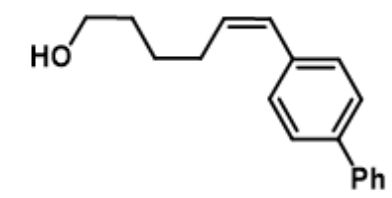

Chemical Formula: $C_{18}H_{20}O$
Exact Mass: 252.1514

Monoisotopic Mass, Even Electron Ions
3 formula(e) evaluated with 1 results within limits (up to 50 closest results for each mass)
Elements Used:
C: 18-18 H: 10-20 B: 0-2 Na: 0-1

1: TOF MS ES+
2.09e+005

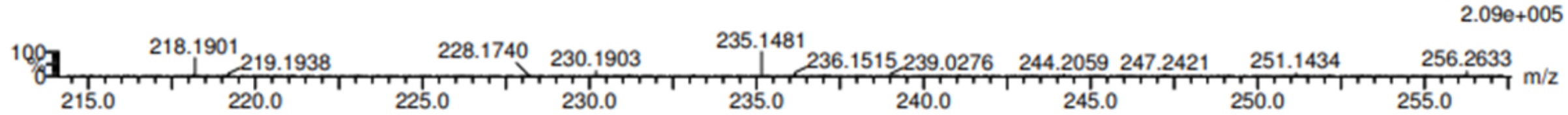

	Mass	Calc. Mass	mDa	PPM	DBE	i-FIT	Norm	Conf(%)	Formula
Minimum:					-1.5				
Maximum:			5.0	5.0	500.0				
	235.1481	235.1487	-0.6	-2.6	9.5	460.8	n/a	n/a	C18 H19

^{1}H NMR (400 MHz, Chloroform-*d*) of Compound 13

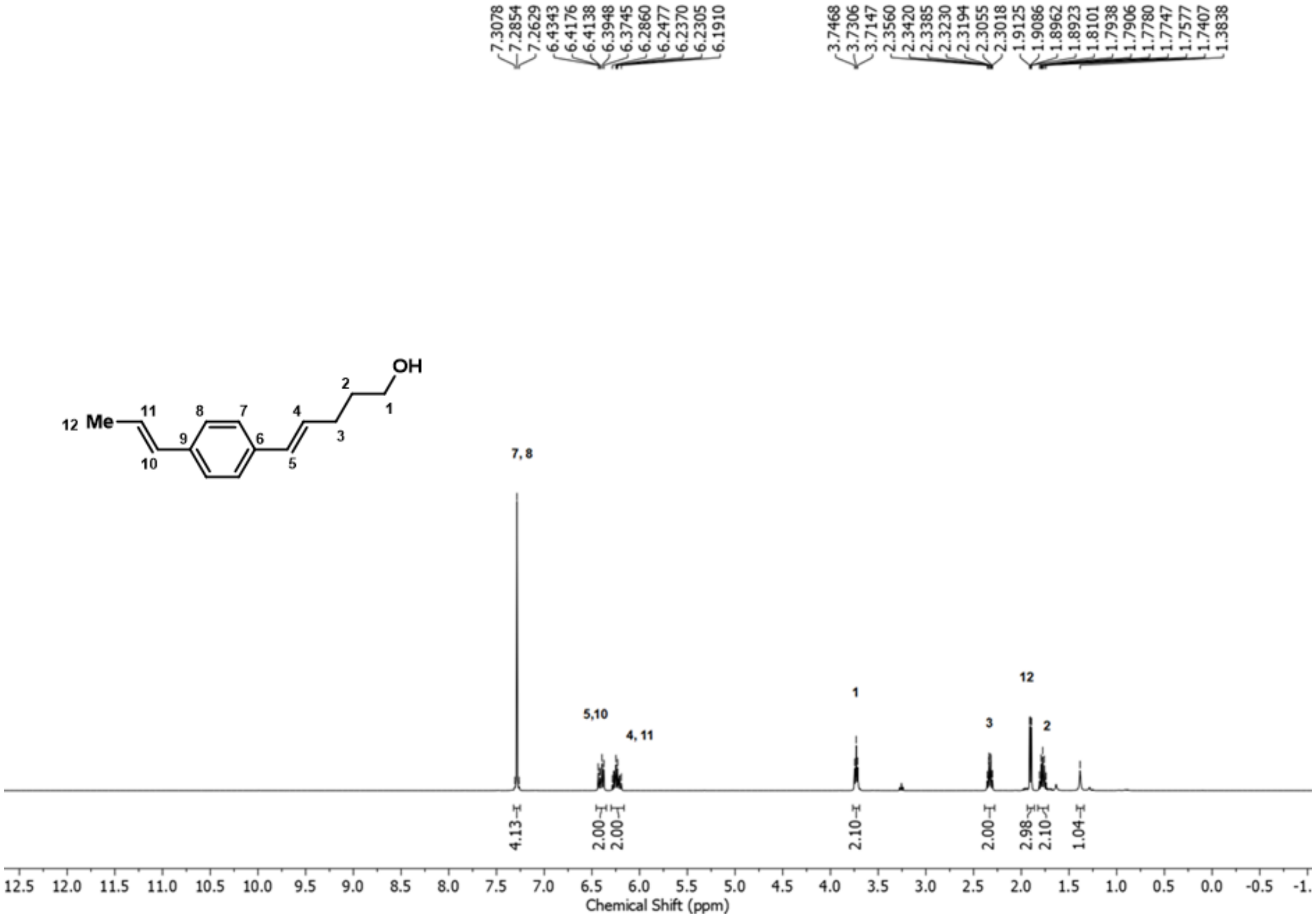

^{13}C NMR (101 MHz, Chloroform-*d*) of Compound 13

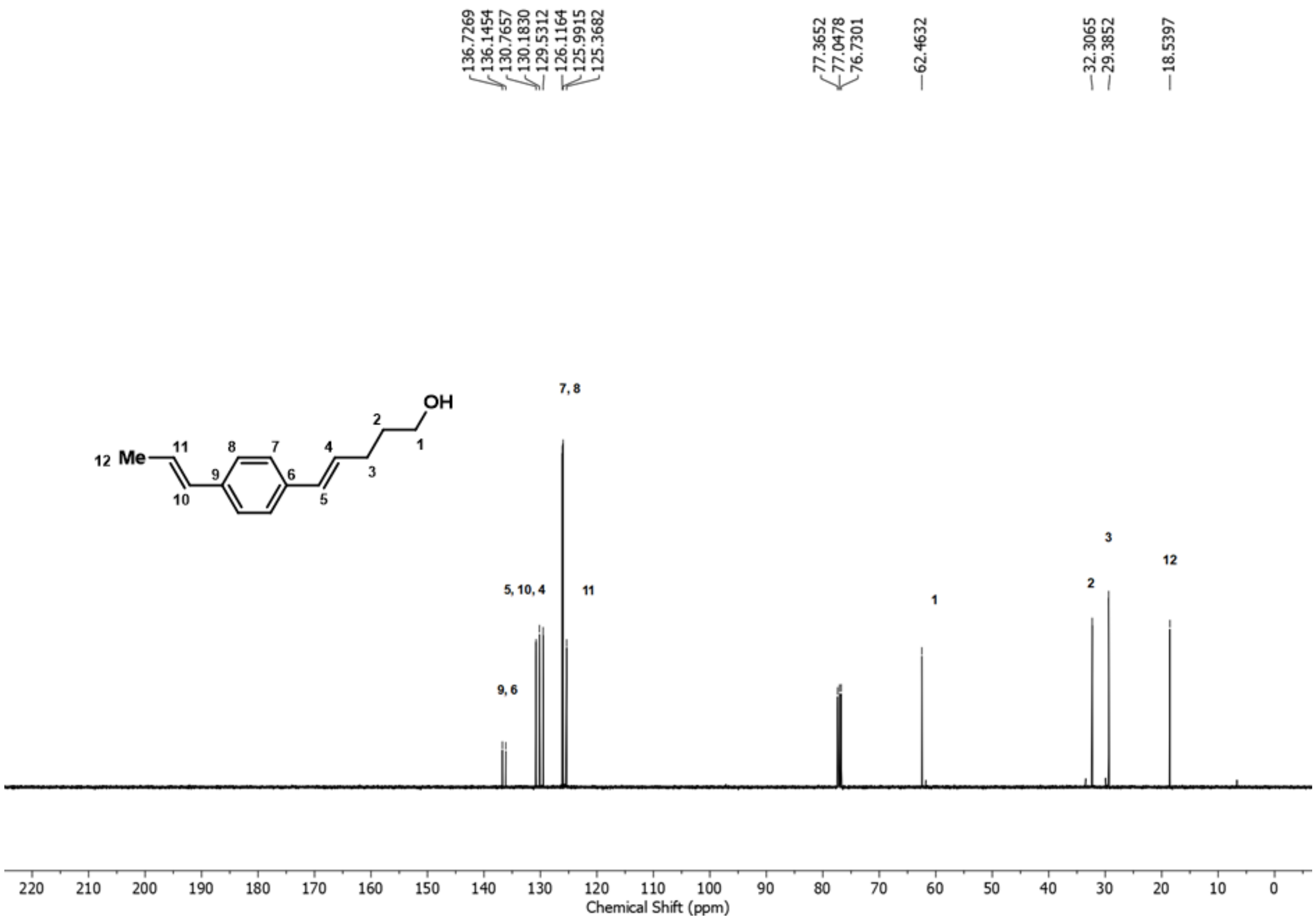

HRMS (ASAP) of Compound 13

Elemental Composition Report **Page 1**

Single Mass Analysis
Tolerance = 5.0 PPM / DBE: min = -1.5, max = 500.0
Element prediction: Off
Number of isotope peaks used for i-FIT = 3

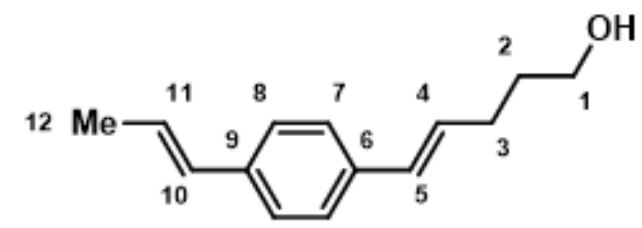

Chemical Formula: $C_{14}H_{18}O$
Exact Mass: 202.1358

Monoisotopic Mass, Even Electron Ions
4 formula(e) evaluated with 1 results within limits (up to 50 closest results for each mass)
Elements Used:
C: 14-20 H: 1-20 Na: 0-1 Sn: 0-1

DCM
KD-265 70 (1.201) Cm (70:78-143:154)

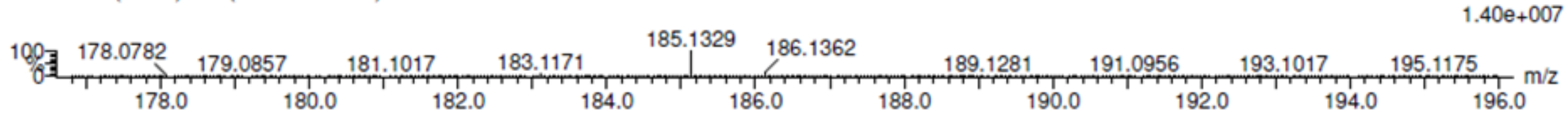

Minimum:				-1.5				
Maximum:		5.0	5.0	500.0				
Mass	Calc. Mass	mDa	PPM	DBE	i-FIT	Norm	Conf(%)	Formula
185.1329	185.1330	-0.1	-0.5	6.5	671.7	n/a	n/a	C14 H17

1H NMR (300 MHz, Chloroform-*d*) of Compound 16

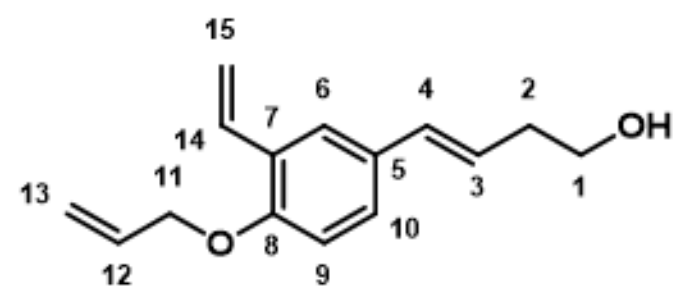

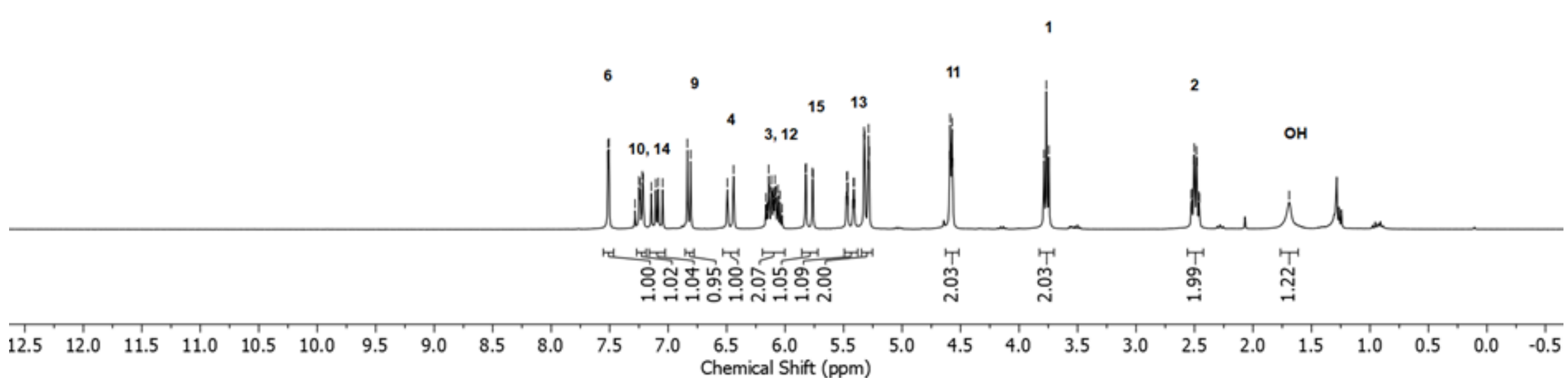

^{13}C NMR (75 MHz, Chloroform-*d*) of Compound 16

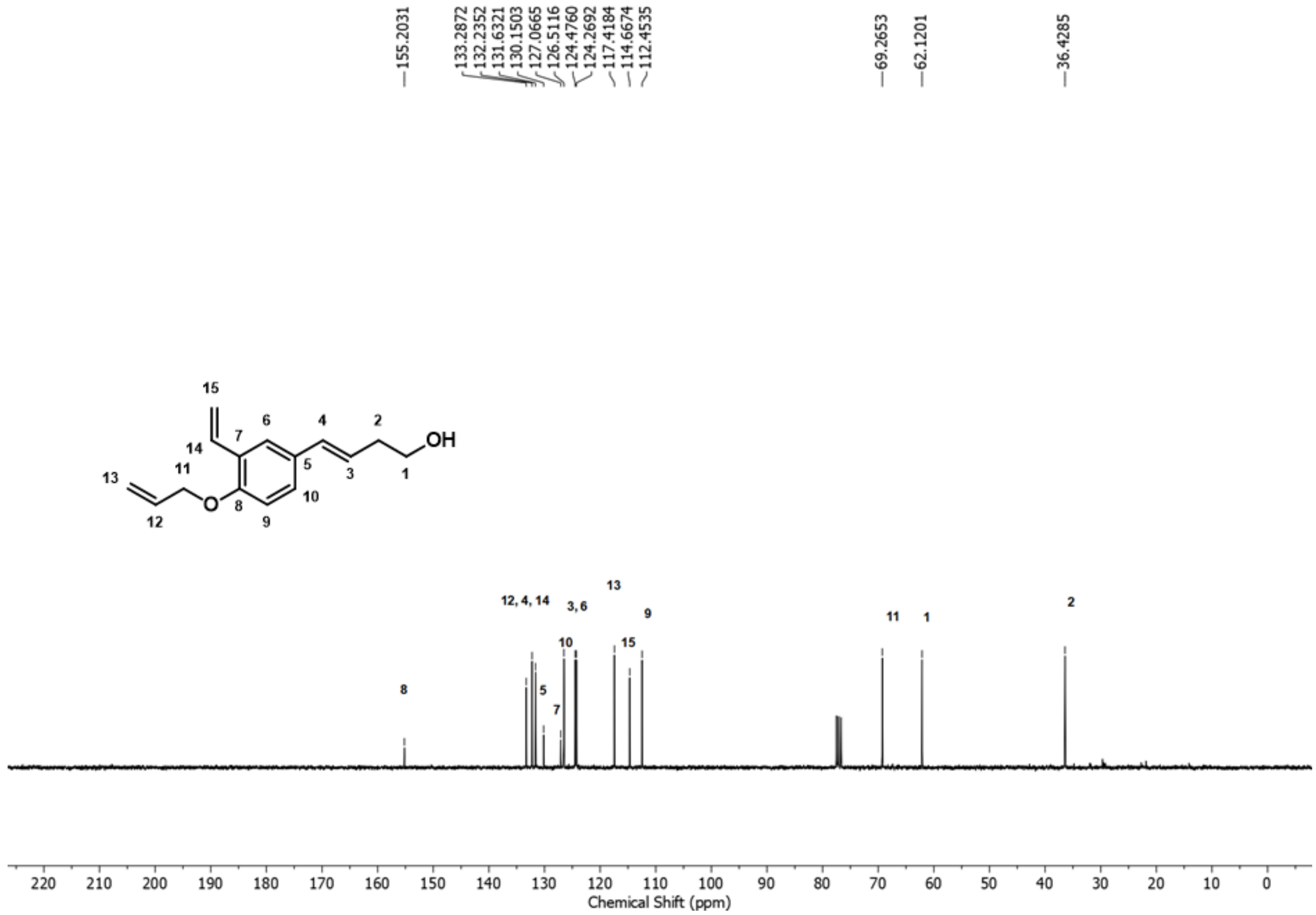

HRMS (ASAP) of Compound 16

Elemental Composition Report **Page 1**

Single Mass Analysis
Tolerance = 50.0 PPM / DBE: min = -1.5, max = 500.0
Element prediction: Off
Number of isotope peaks used for i-FIT = 3

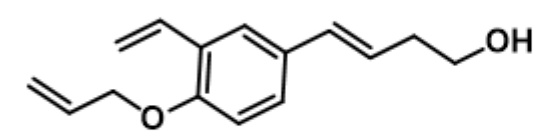

Chemical Formula: $C_{15}H_{18}O_2$
Exact Mass: 230.1307

Monoisotopic Mass, Even Electron Ions
19 formula(e) evaluated with 1 results within limits (up to 50 closest results for each mass)
Elements Used:
C: 15-15 H: 8-20 B: 0-2 O: 1-2 Na: 0-1 Sn: 0-1

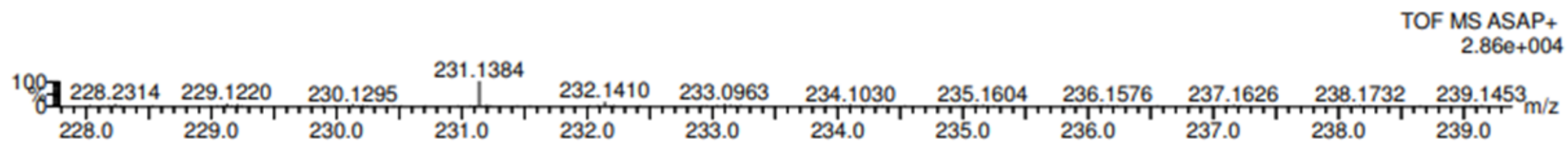

Minimum:				-1.5				
Maximum:		5.0	50.0	500.0				
Mass	Calc. Mass	mDa	PPM	DBE	i-FIT	Norm	Conf(%)	Formula
231.1384	231.1385	-0.1	-0.4	6.5	159.0	n/a	n/a	C15 H19 O2

^{1}H NMR (500 MHz, Chloroform-*d*) of Compound 18

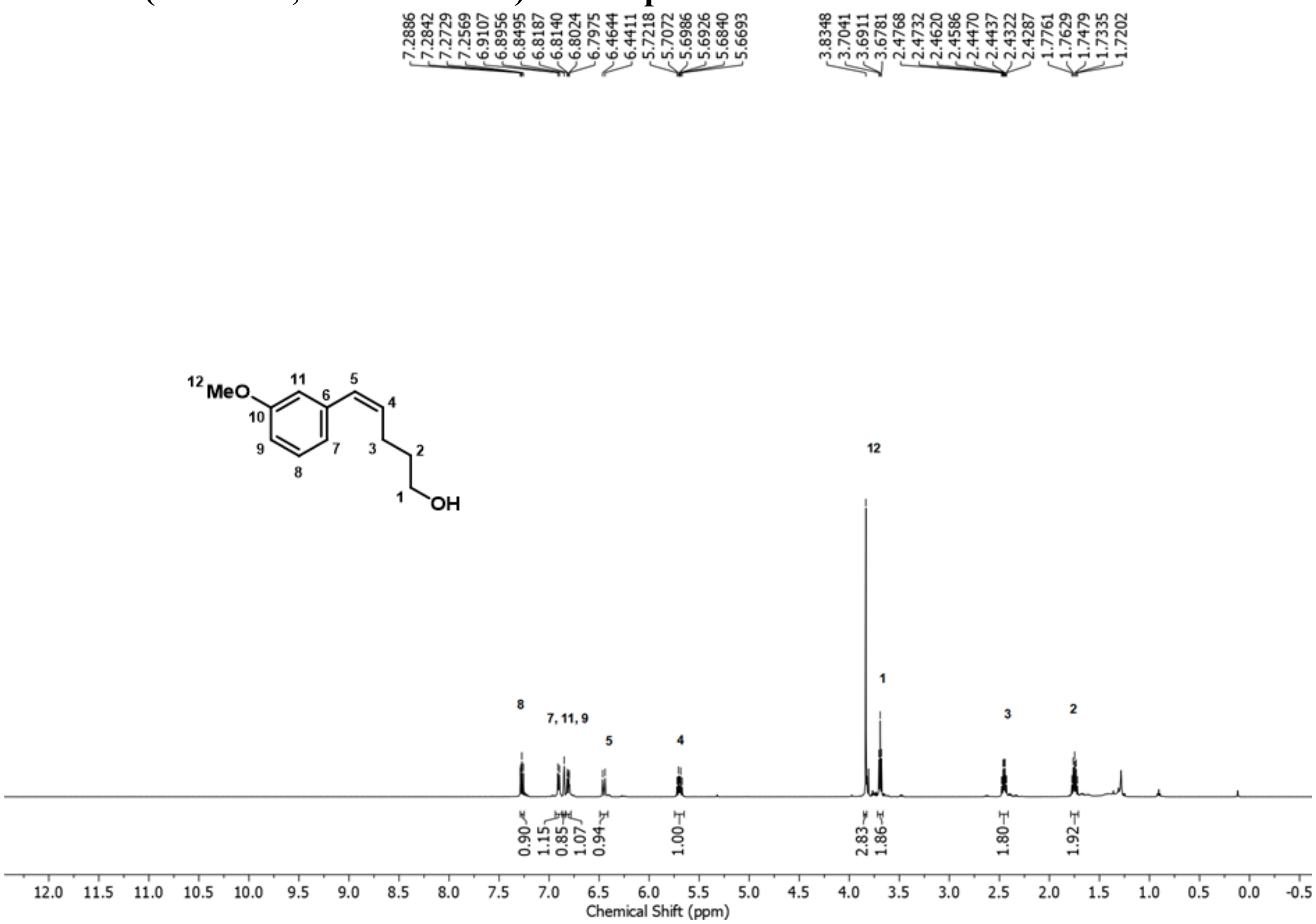

^{13}C NMR (126 MHz, Chloroform-*d*) of Compound 18

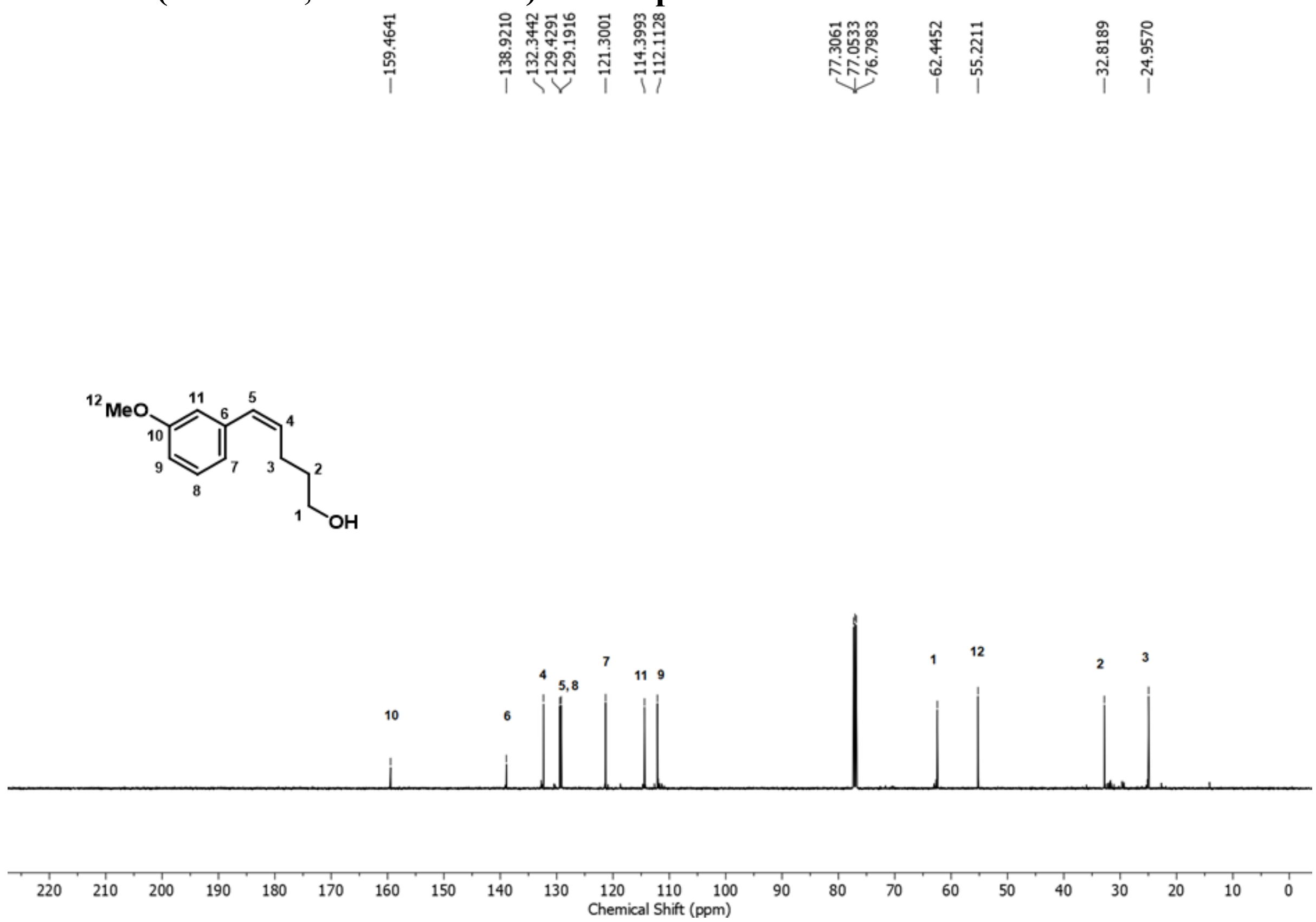

HRMS (ESI) of Compound 18

Elemental Composition Report

Page 1

Single Mass Analysis
Tolerance = 5.0 PPM / DBE: min = -1.5, max = 500.0
Element prediction: Off
Number of isotope peaks used for i-FIT = 3

Monoisotopic Mass, Even Electron Ions
2 formula(e) evaluated with 1 results within limits (up to 50 closest results for each mass)
Elements Used:
C: 12-12 H: 10-20 O: 1-1 Na: 0-1

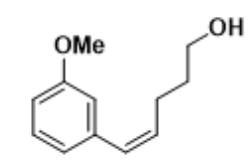

Chemical Formula: $C_{12}H_{16}O_2$
Exact Mass: 192.1150

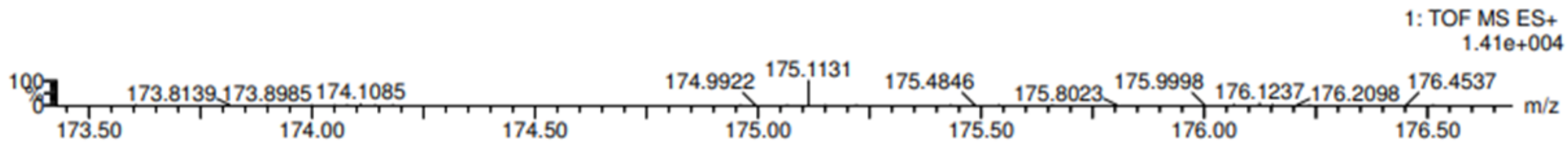

Minimum:				-1.5				
Maximum:		5.0	5.0	500.0				
Mass	Calc. Mass	mDa	PPM	DBE	i-FIT	Norm	Conf(%)	Formula
175.1131	175.1123	0.8	4.6	5.5	128.1	n/a	n/a	C12 H15 O

^{1}H NMR (500 MHz, Benzene-d_6) of Compound 6a

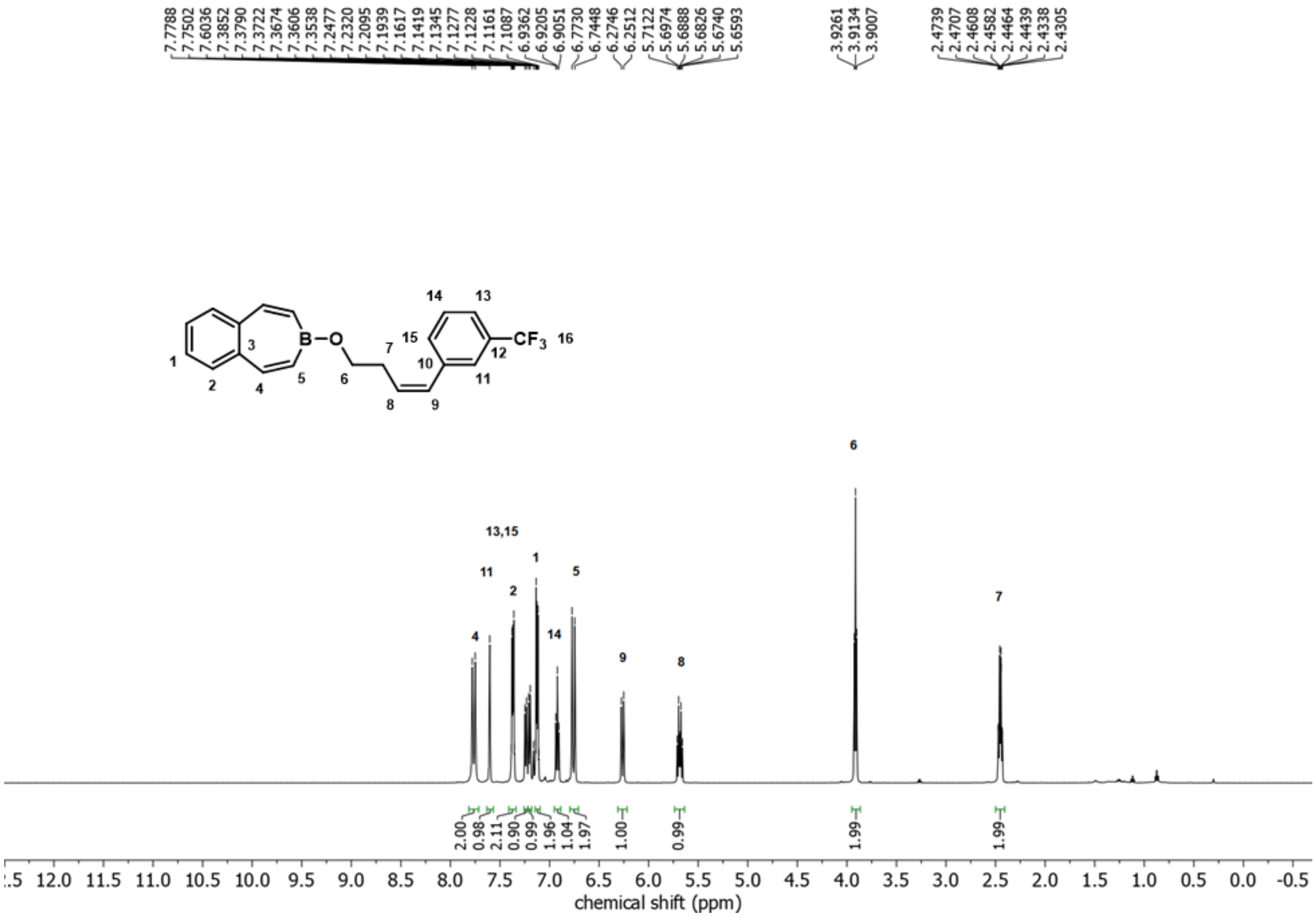

$^{13}C\{^{1}H\}$ NMR (126 MHz, Benzene-d_6) of Compound 6a

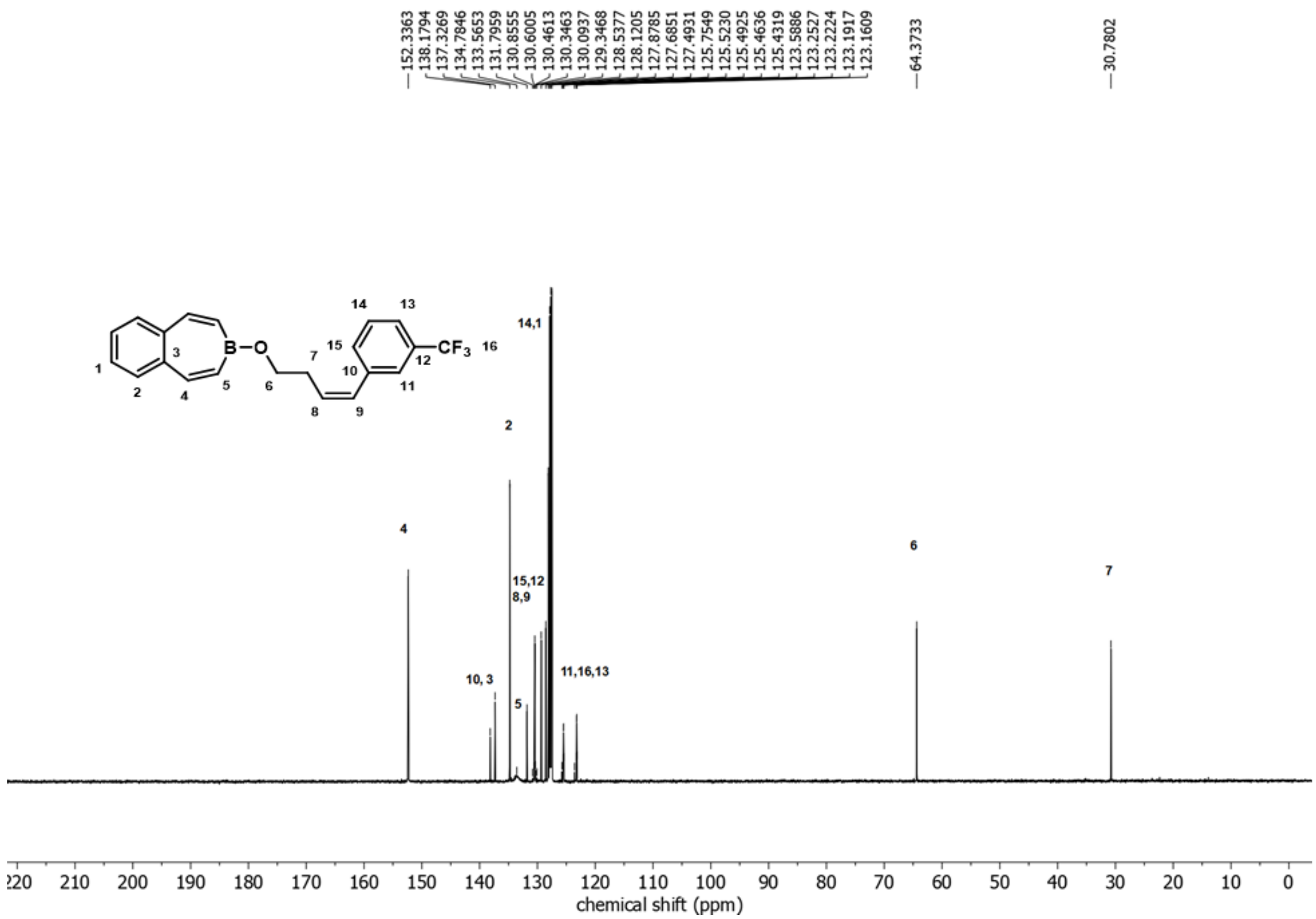

^{19}F NMR (471 MHz, Benzene-d_6) of Compound 6a

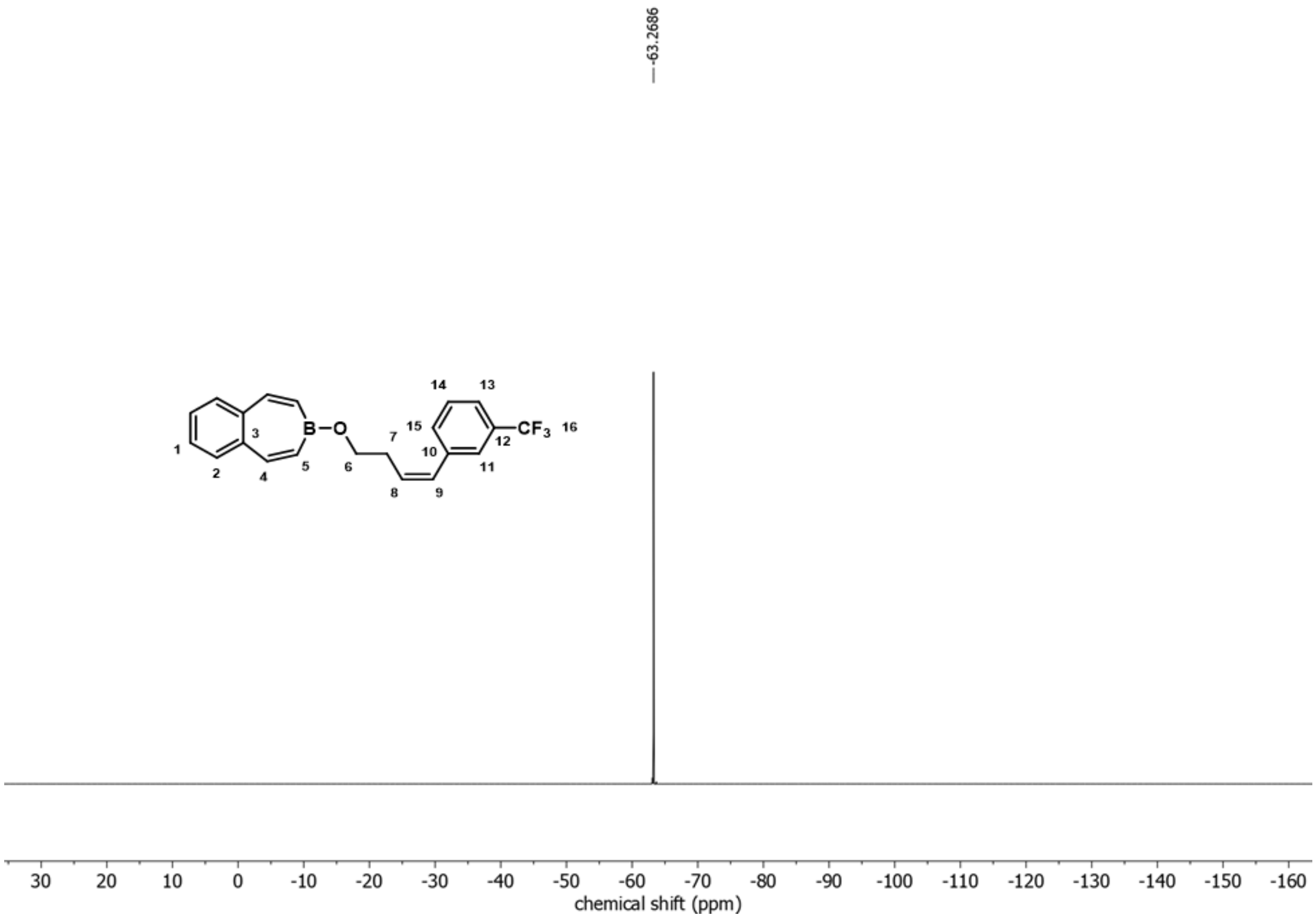

^{11}B NMR (160 MHz, Benzene-d_6) of Compound 6a

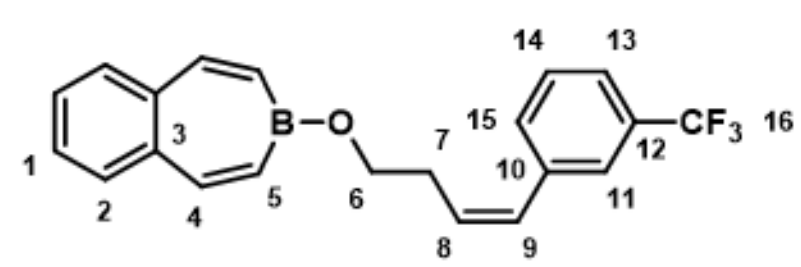

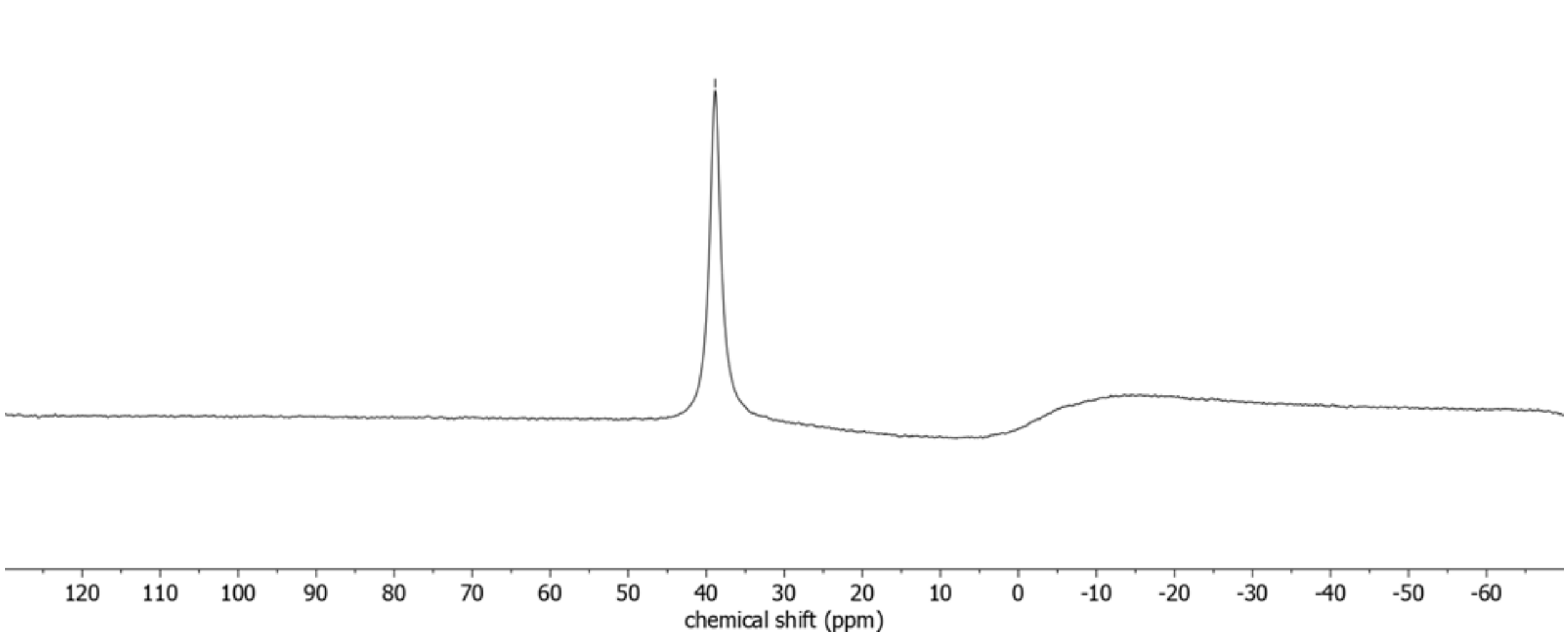

^{1}H -^{1}H COSY (500 MHz, Benzene-d_6) of Compound 6a

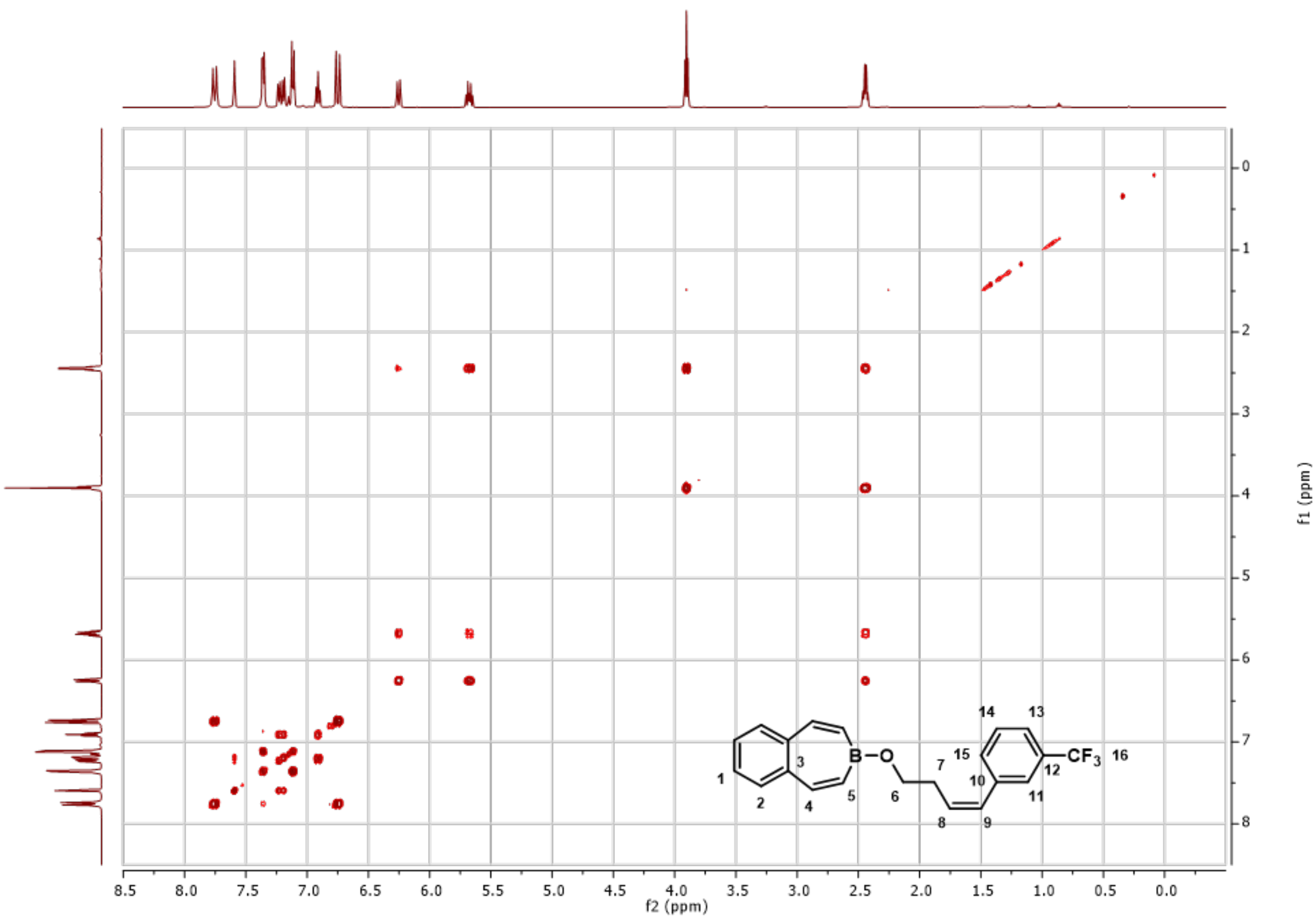

^{1}H -^{13}C HSQC (500 MHz, Benzene-d_6)) of Compound 6a

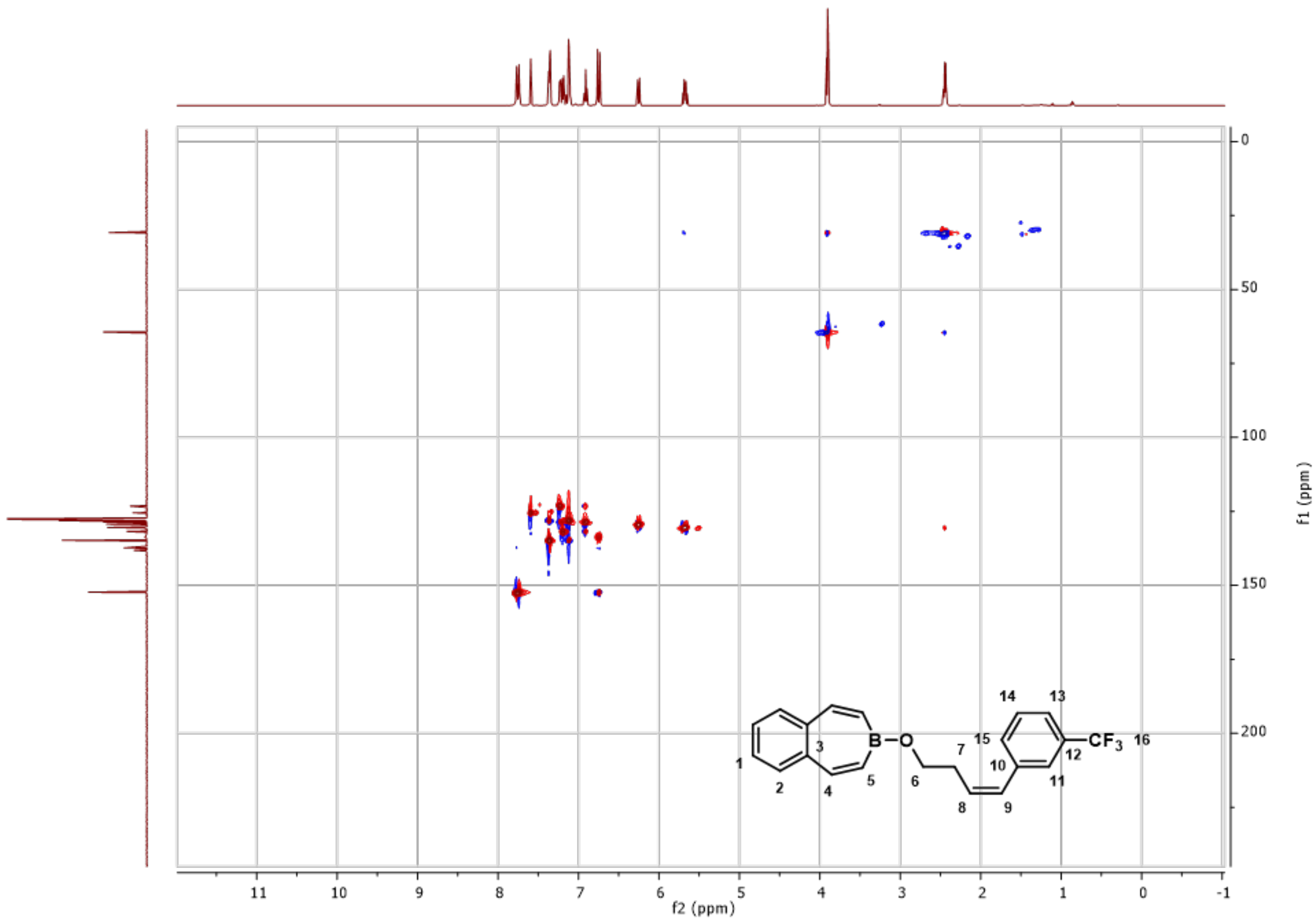

^{1}H -^{13}C HMBC (500 MHz, Benzene-d_6) of Compound 6a

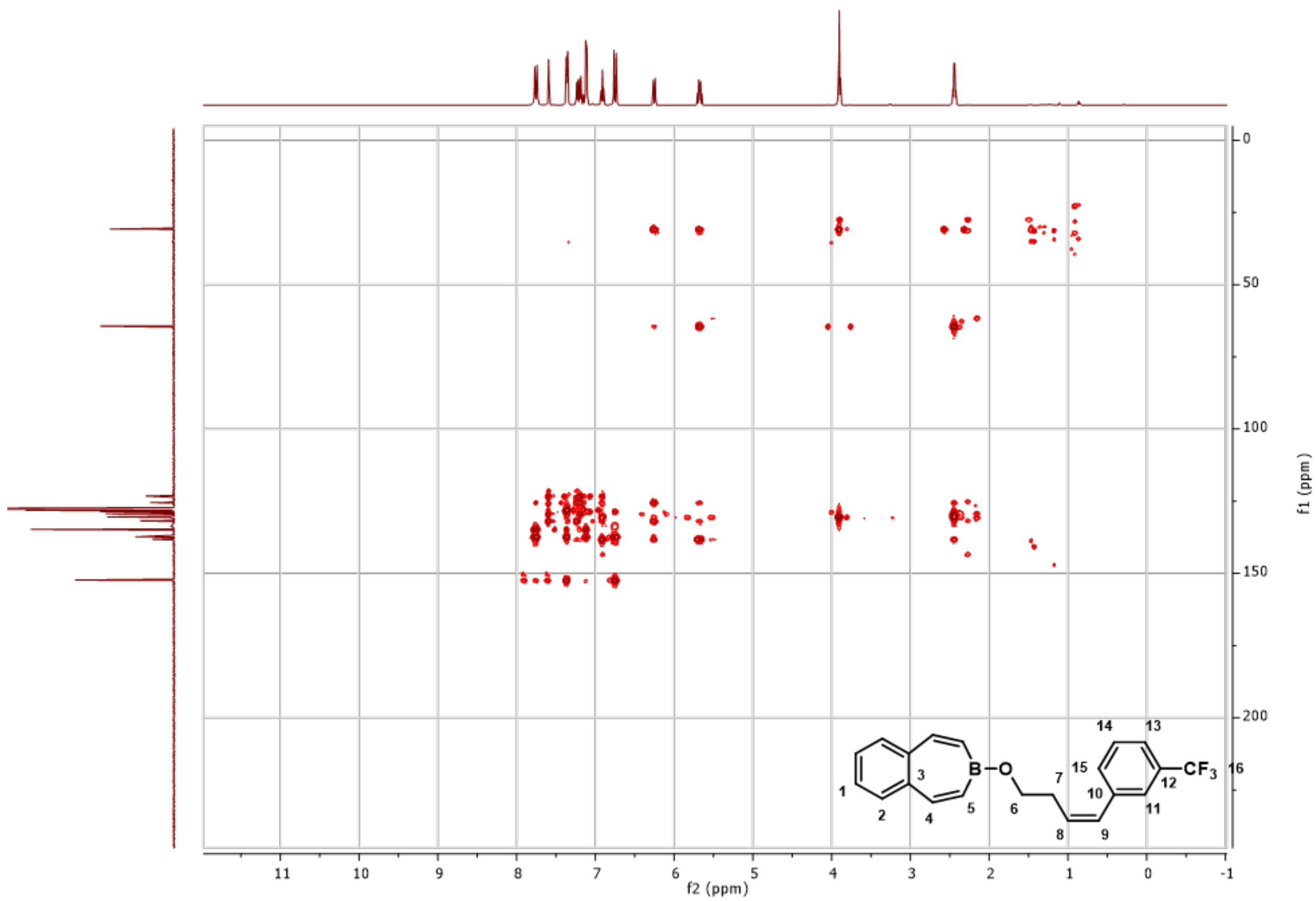

HRMS (ASAP) of Compound 6a

Elemental Composition Report **Page 1**

Single Mass Analysis
Tolerance = 5.0 PPM / DBE: min = -1.5, max = 500.0
Element prediction: Off

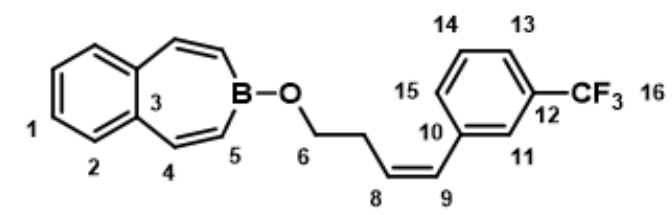

Chemical Formula: $C_{21}H_{18}BF_3O$
Exact Mass: 354.1403

Monoisotopic Mass, Even Electron Ions
37 formula(e) evaluated with 1 results within limits (up to 50 closest results for each mass)
Elements Used:
C: 21-21 H: 7-25 B: 0-1 O: 0-3 F: 0-6
DCM

TOF MS ASAP+
8.31e+007

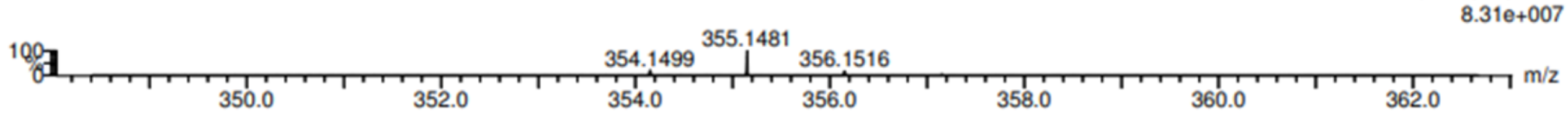

Minimum:				-1.5	
Maximum:		5.0	5.0	500.0	
Mass	Calc. Mass	mDa	PPM	DBE	Formula
355.1481	355.1481	0.0	0.0	11.5	C21 H19 B O F3

^{1}H NMR (500 MHz, Benzene-d_6) of Compound 6b

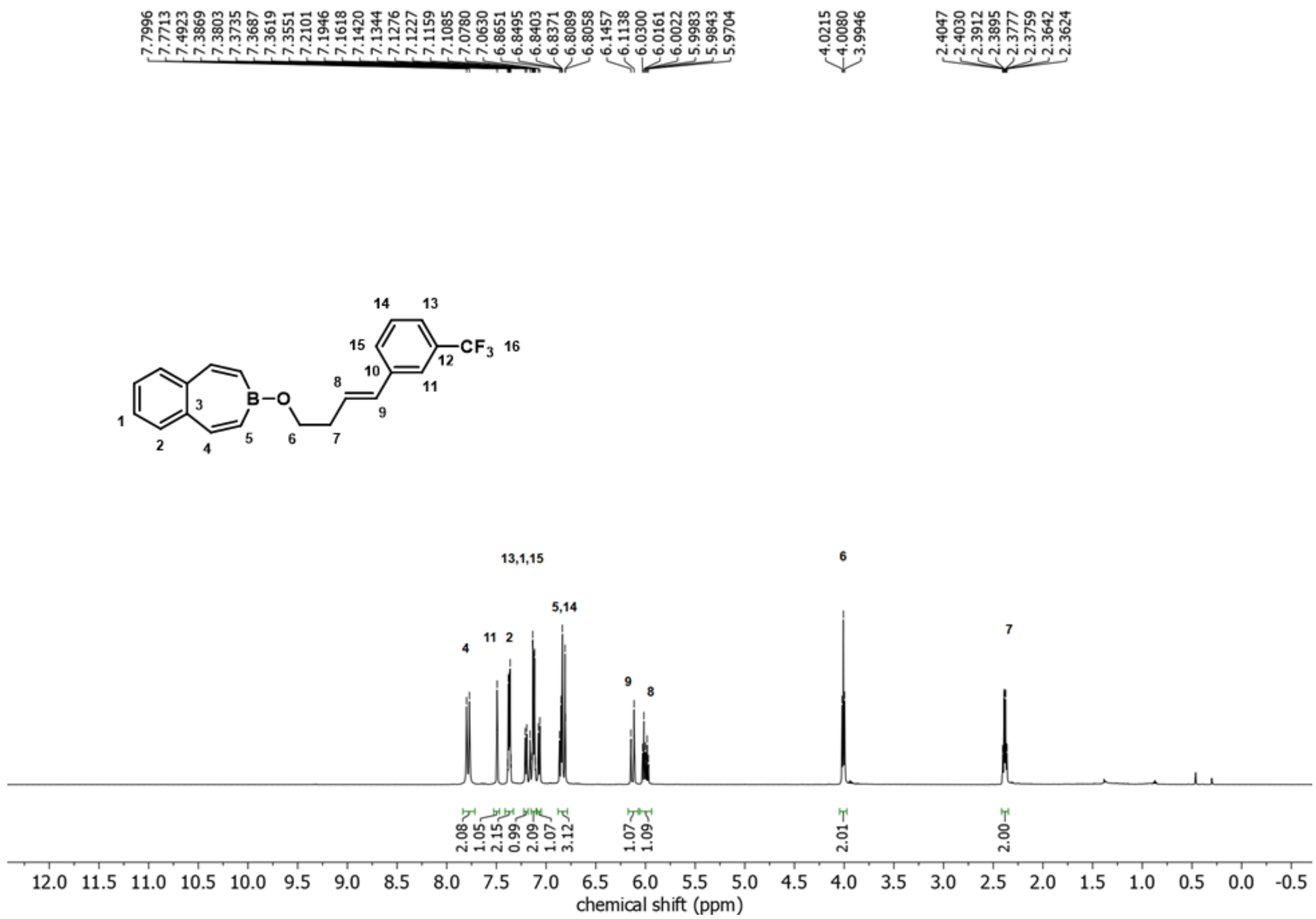

$^{13}C\{^{1}H\}$ NMR (126 MHz, Benzene-d_6) of Compound 6b

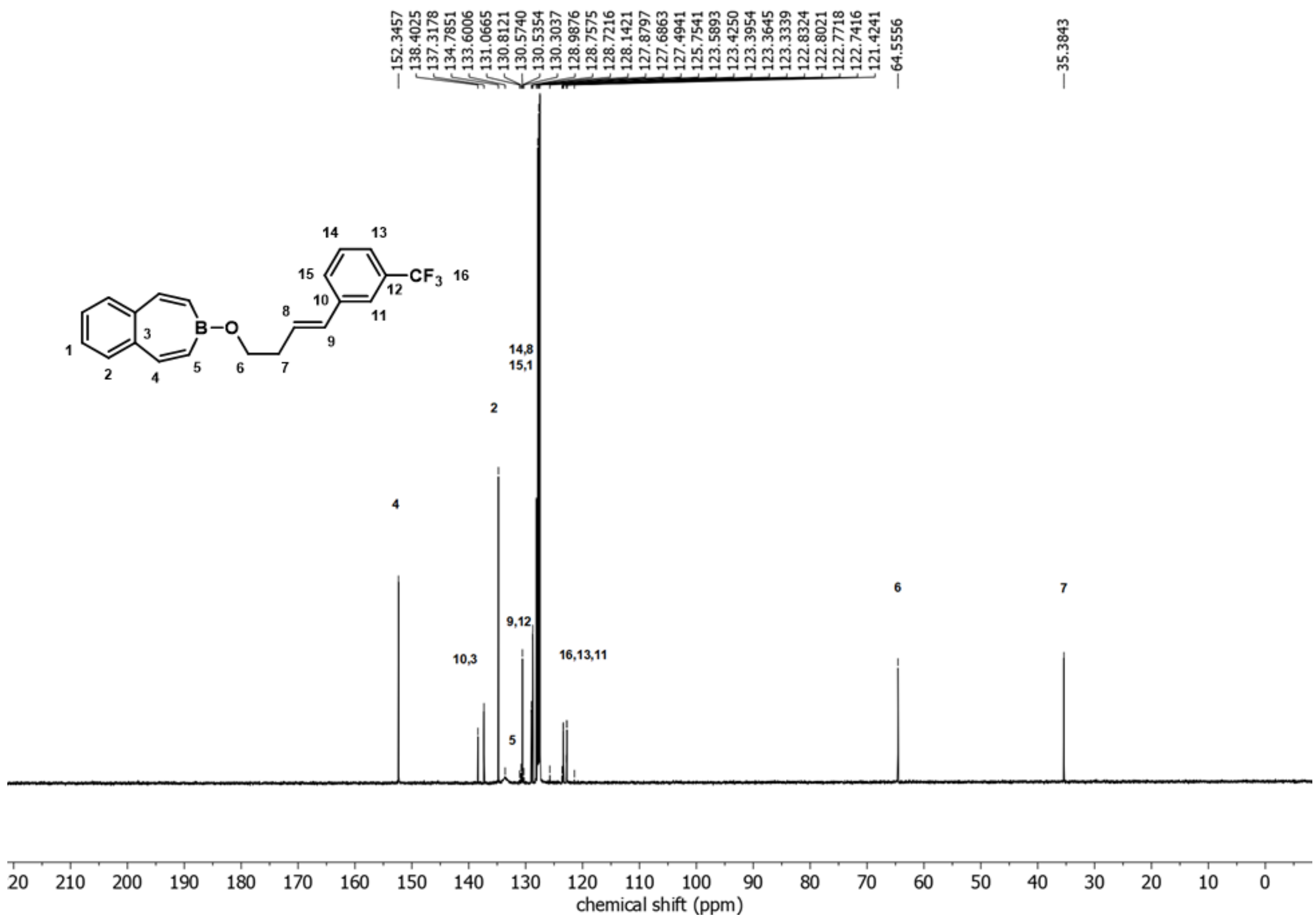

^{19}F NMR (471 MHz, Benzene-d_6) of Compound 6b

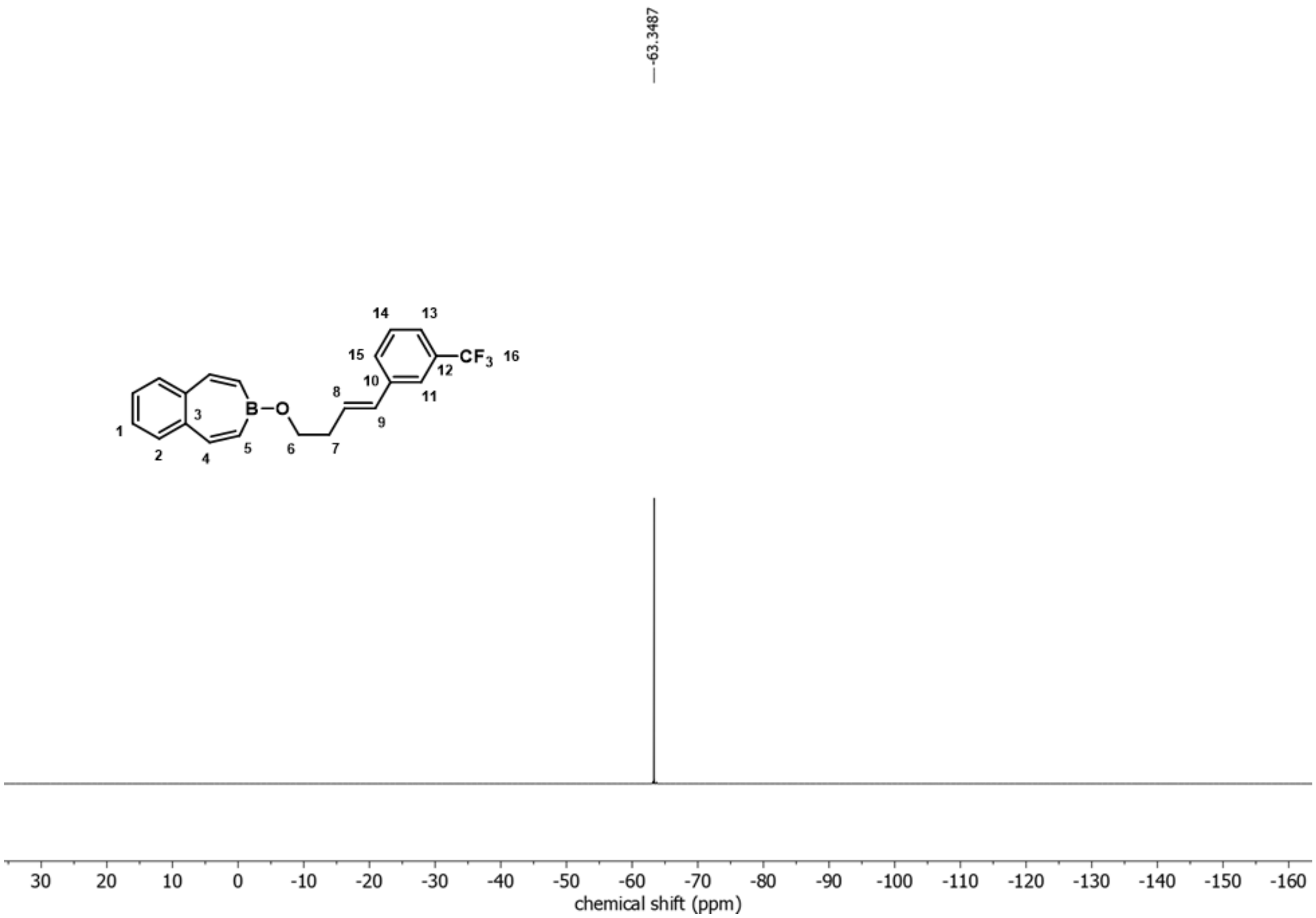

^{11}B NMR (160 MHz, Benzene-d_6) of Compound 6b

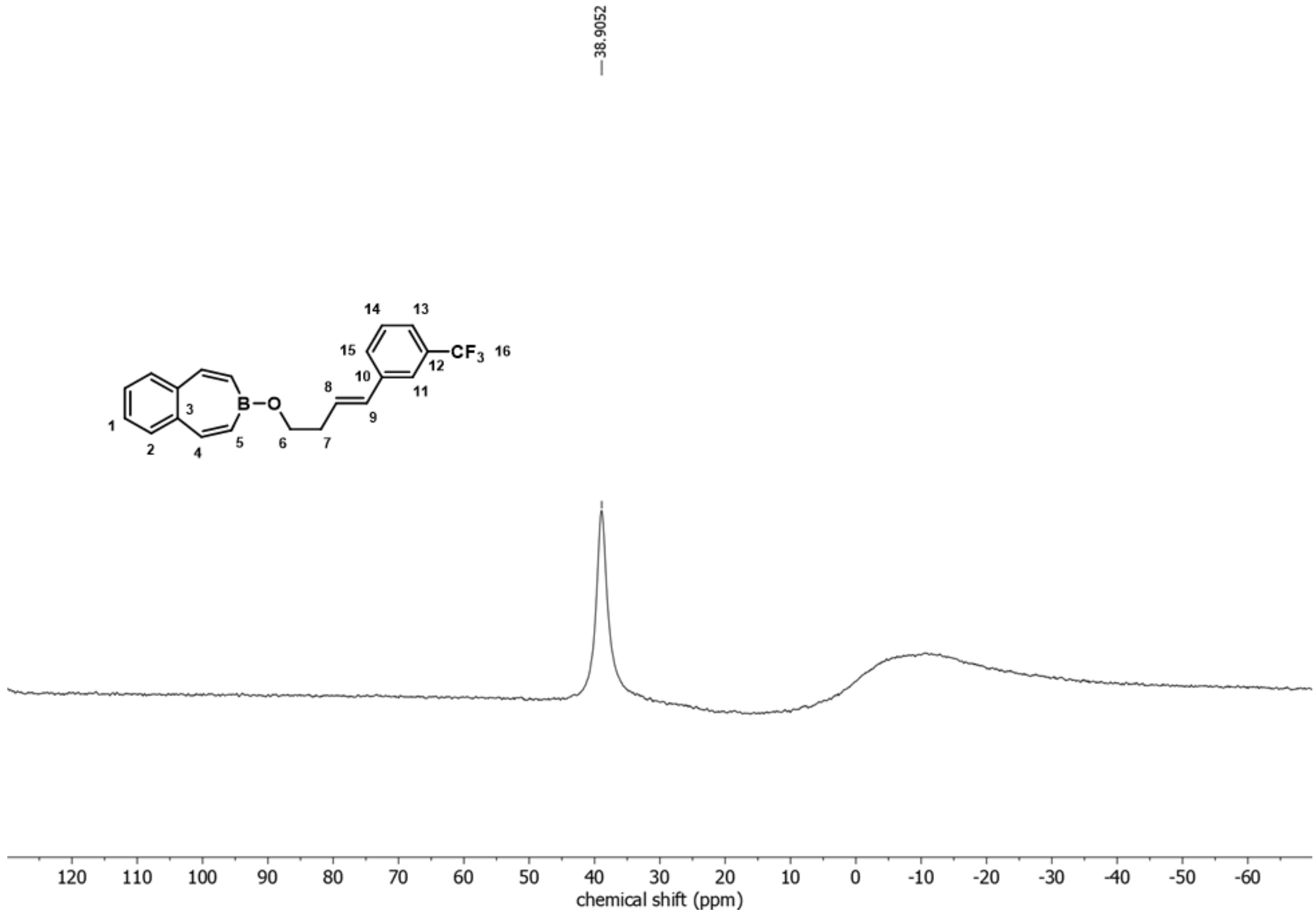

^{1}H -^{1}H COSY (500 MHz, Benzene-d_6) of Compound 6b

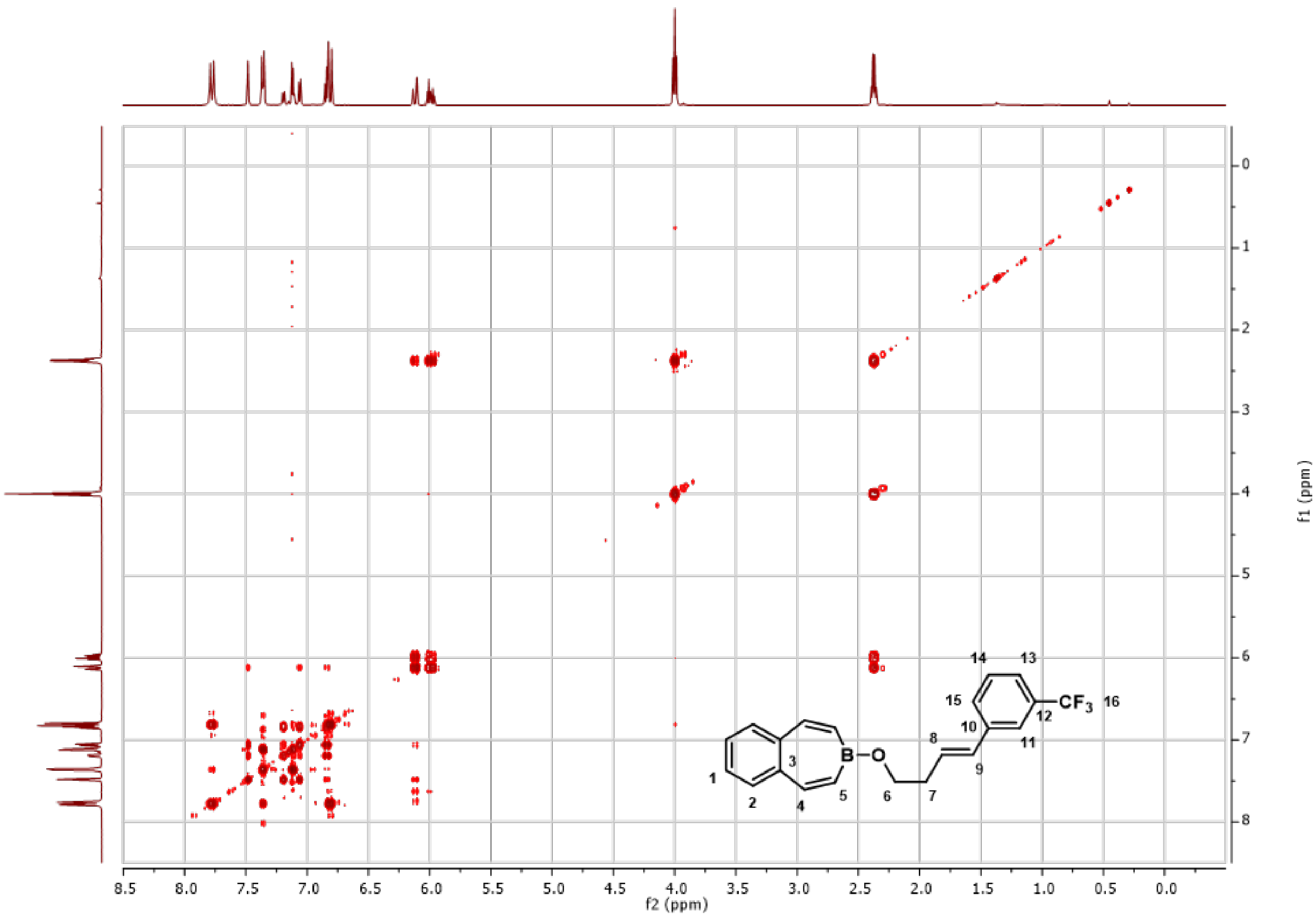

^{1}H -^{13}C HSQC (500 MHz, Benzene-d_6) of Compound 6b

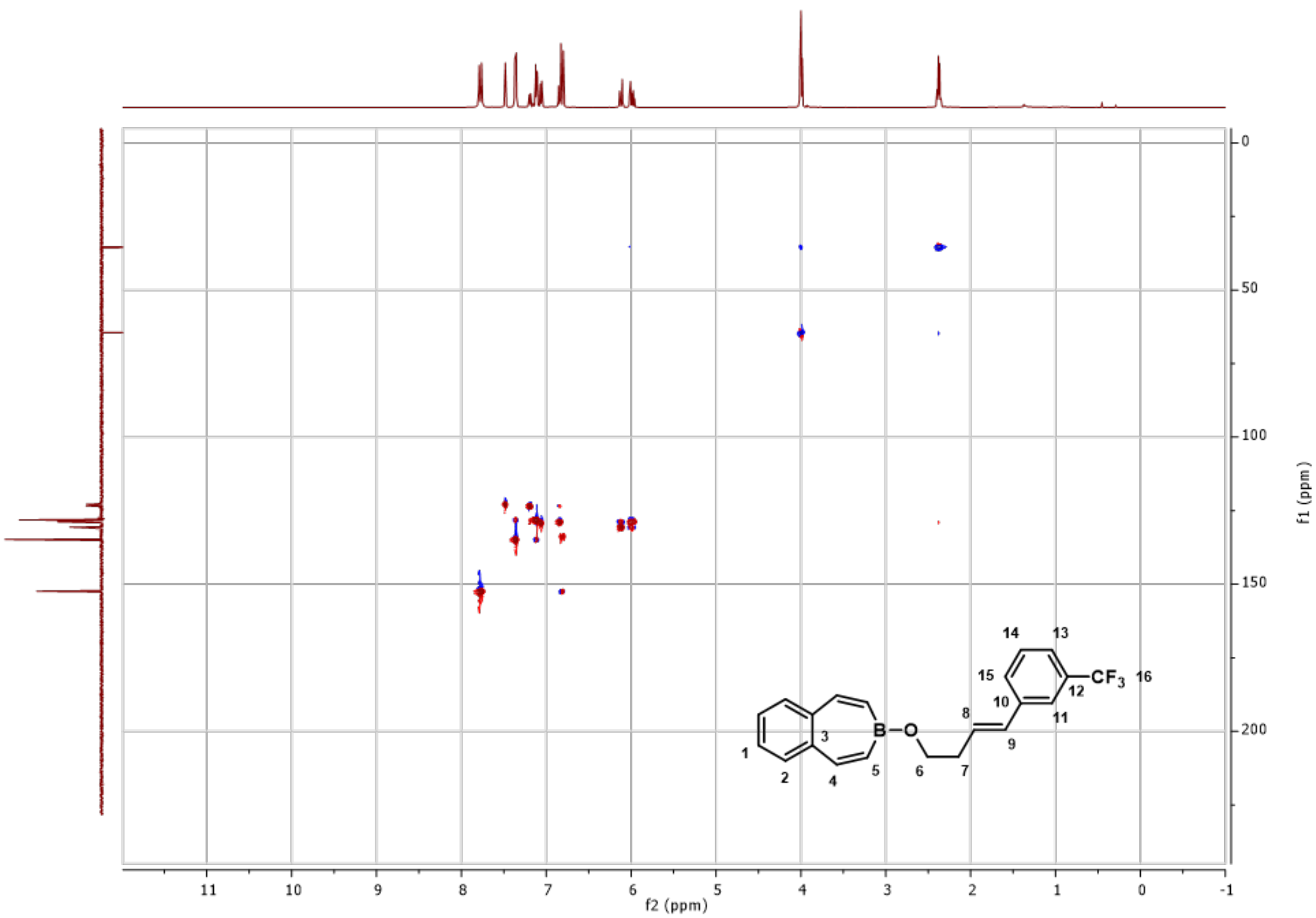

^{1}H -^{13}C HMBC (500 MHz, Benzene-d_6) of Compound 6b

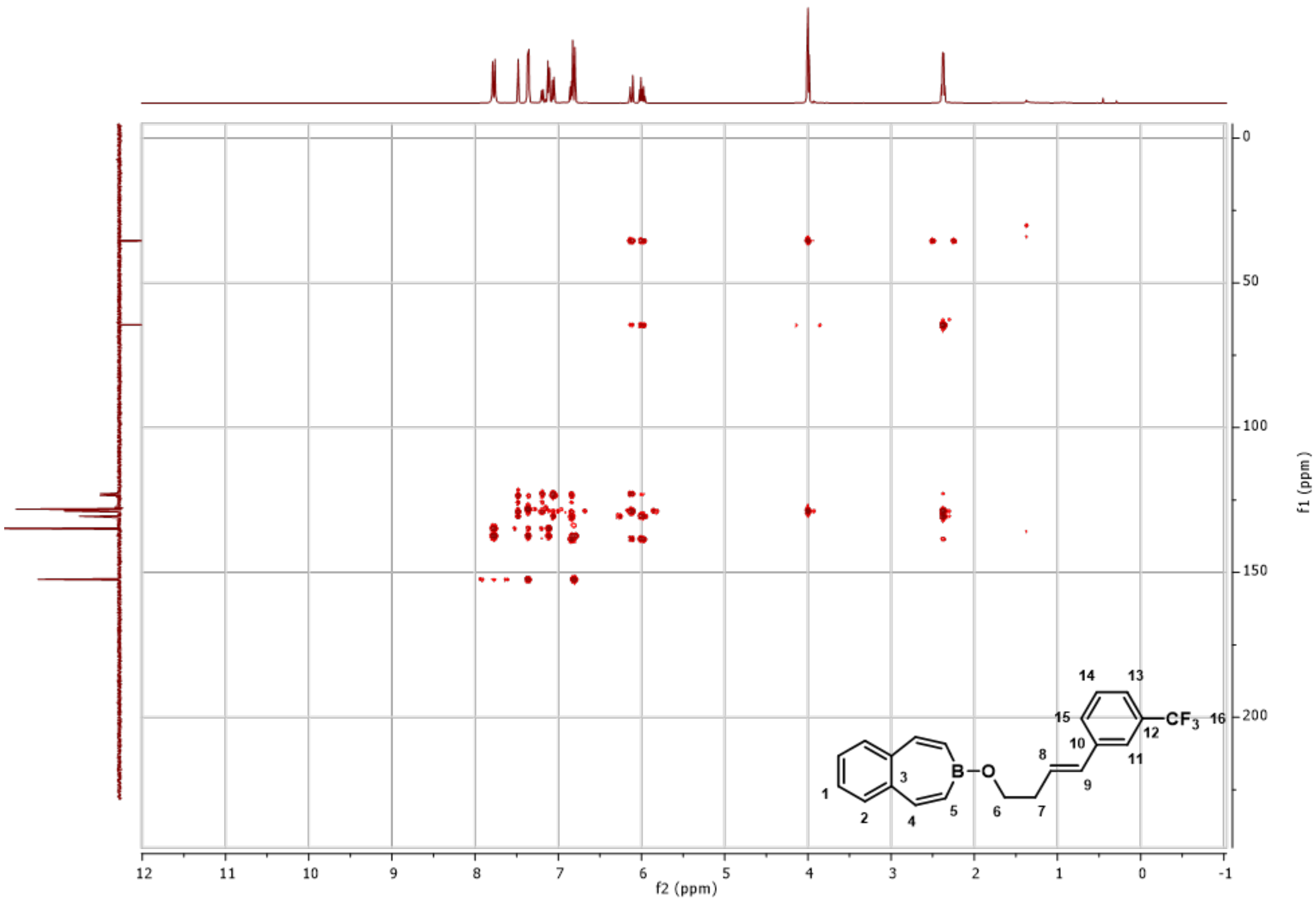

^{1}H NMR (500 MHz, Benzene-d_6) of Compound 6c

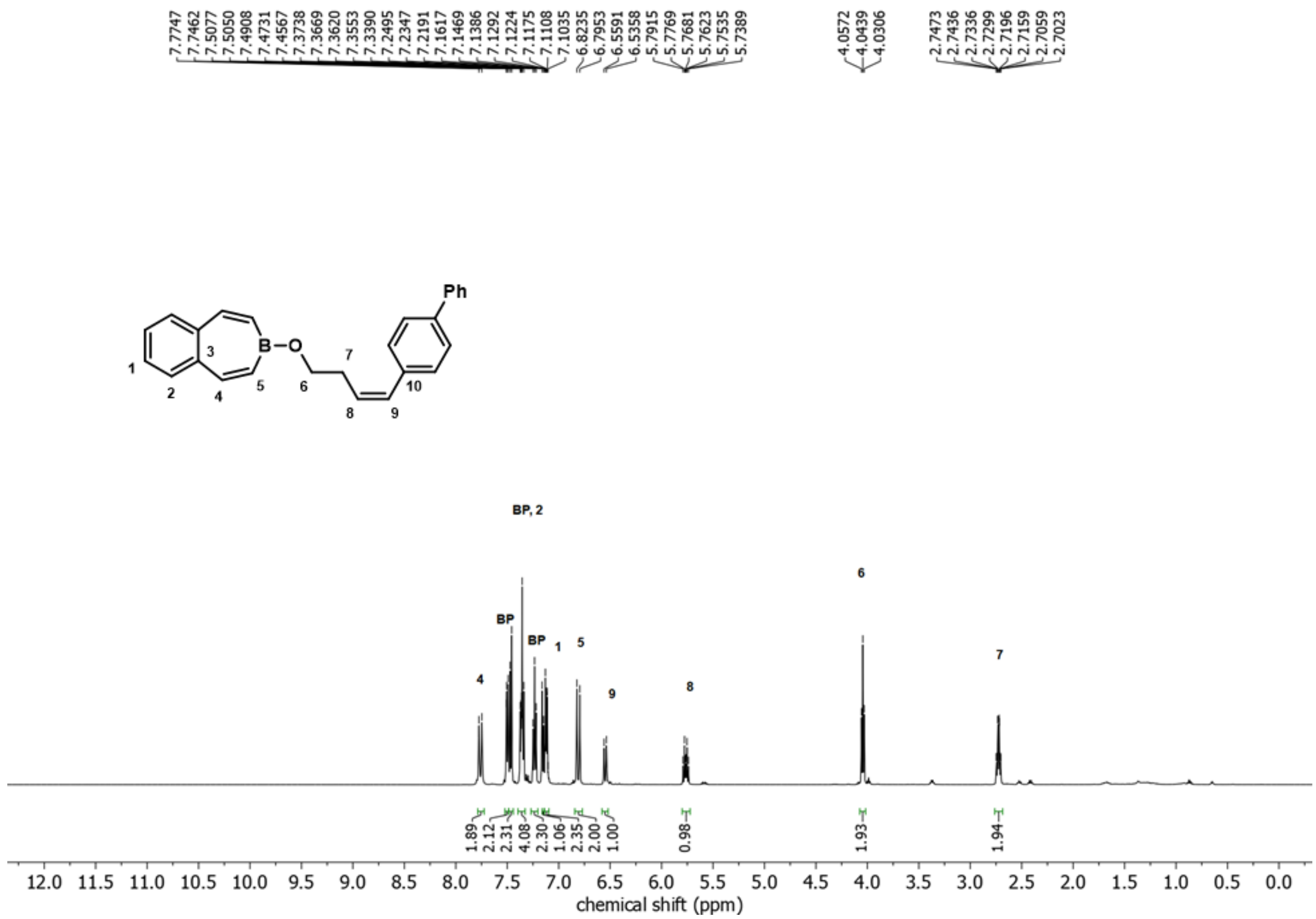

$^{13}C\{^{1}H\}$ NMR (126 MHz, Benzene-d_6) of Compound 6c

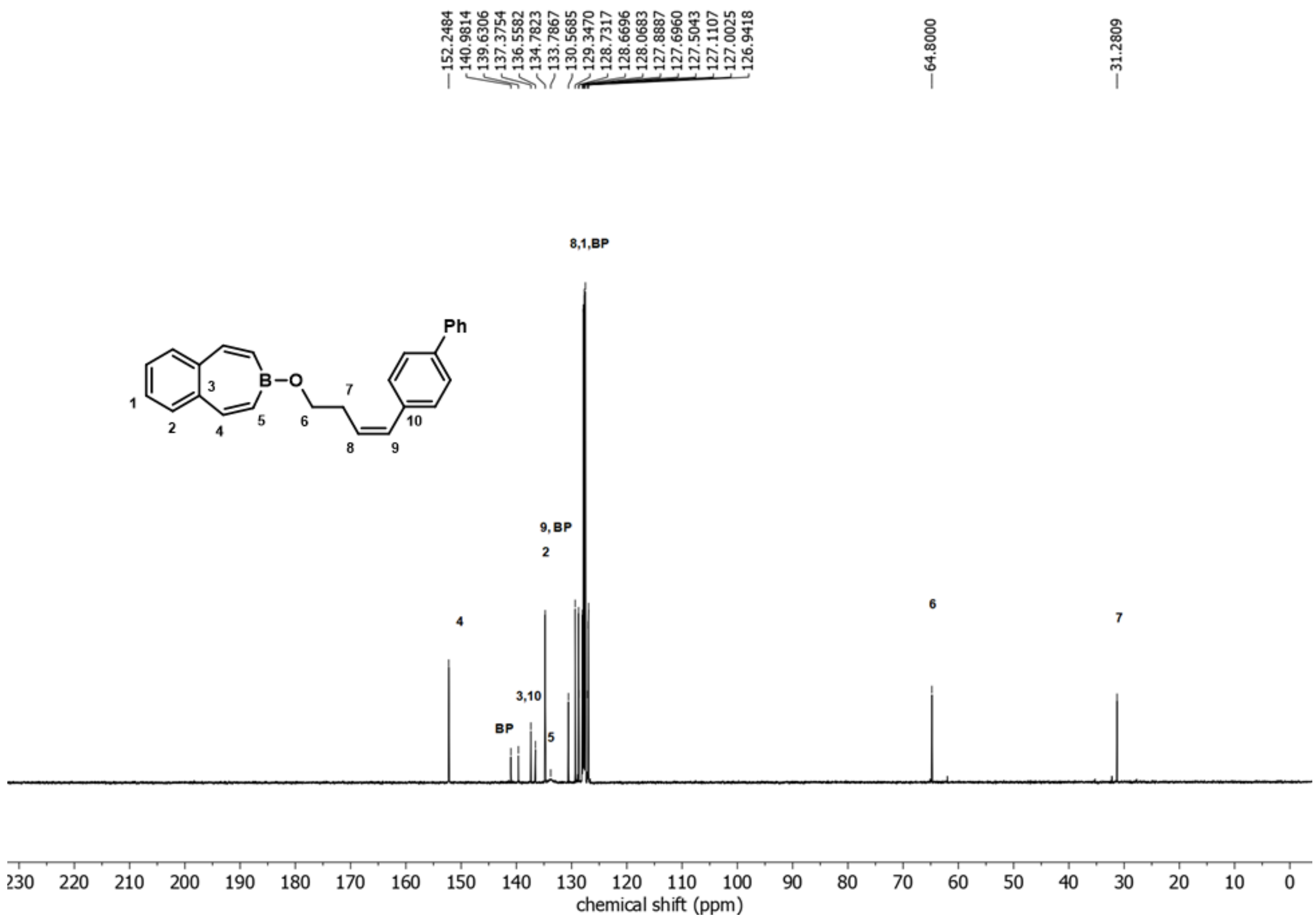

^{11}B NMR (160 MHz, Benzene-d_6) of Compound 6c

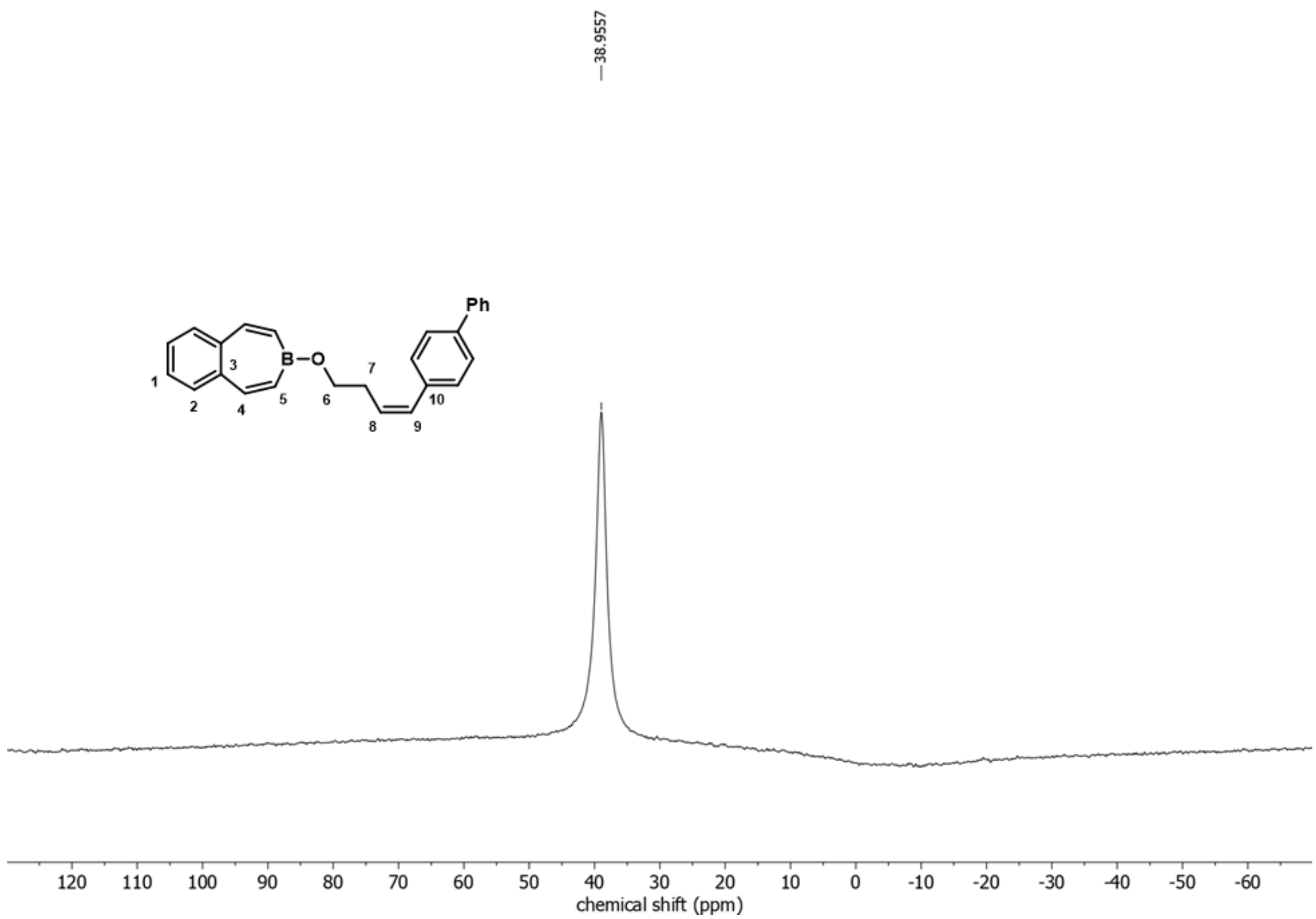

^{1}H -^{1}H COSY (500 MHz, Benzene-d_6) of Compound 6c

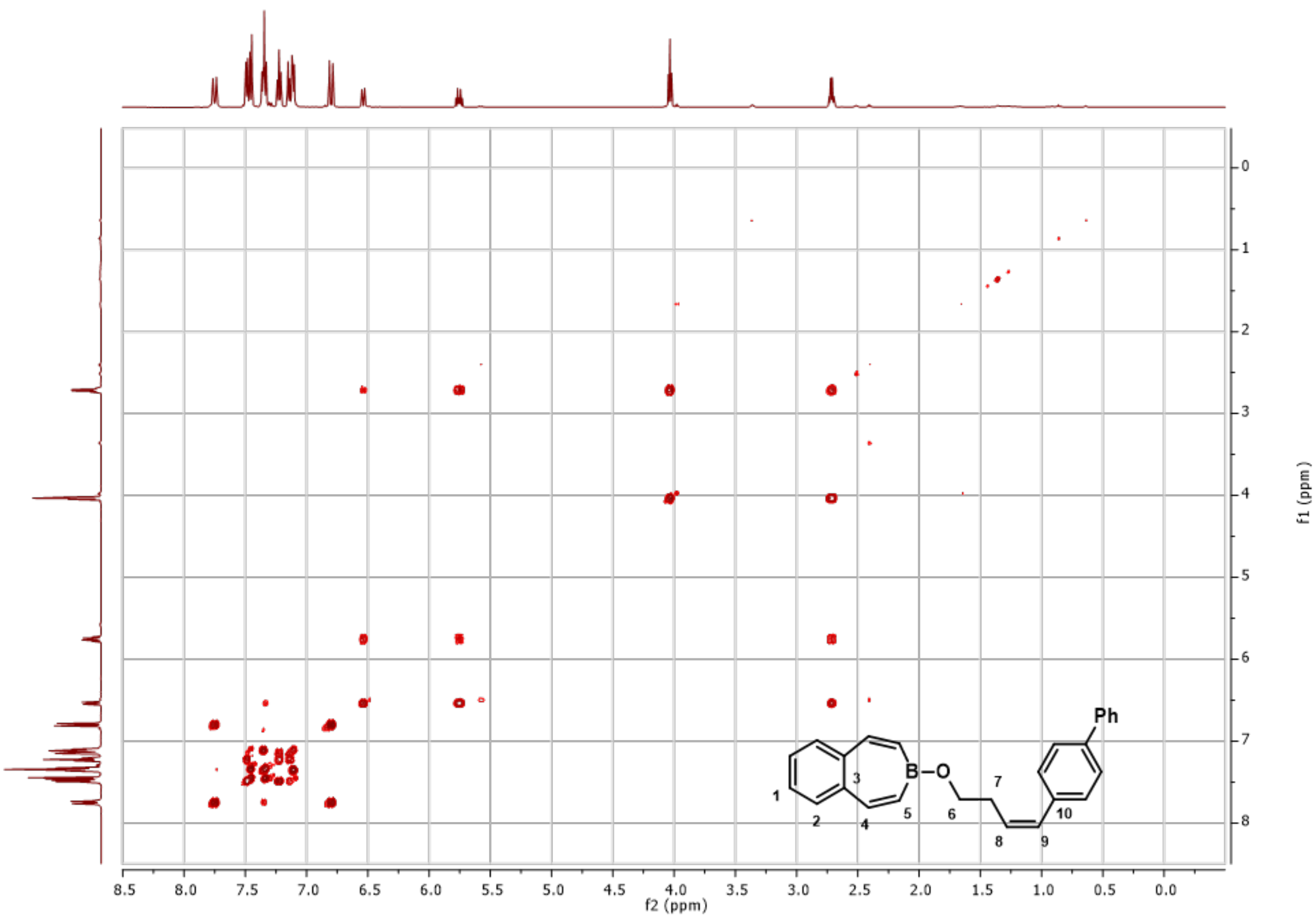

^1H -^{13}C HSQC (500 MHz, Benzene-d_6) of Compound 6c

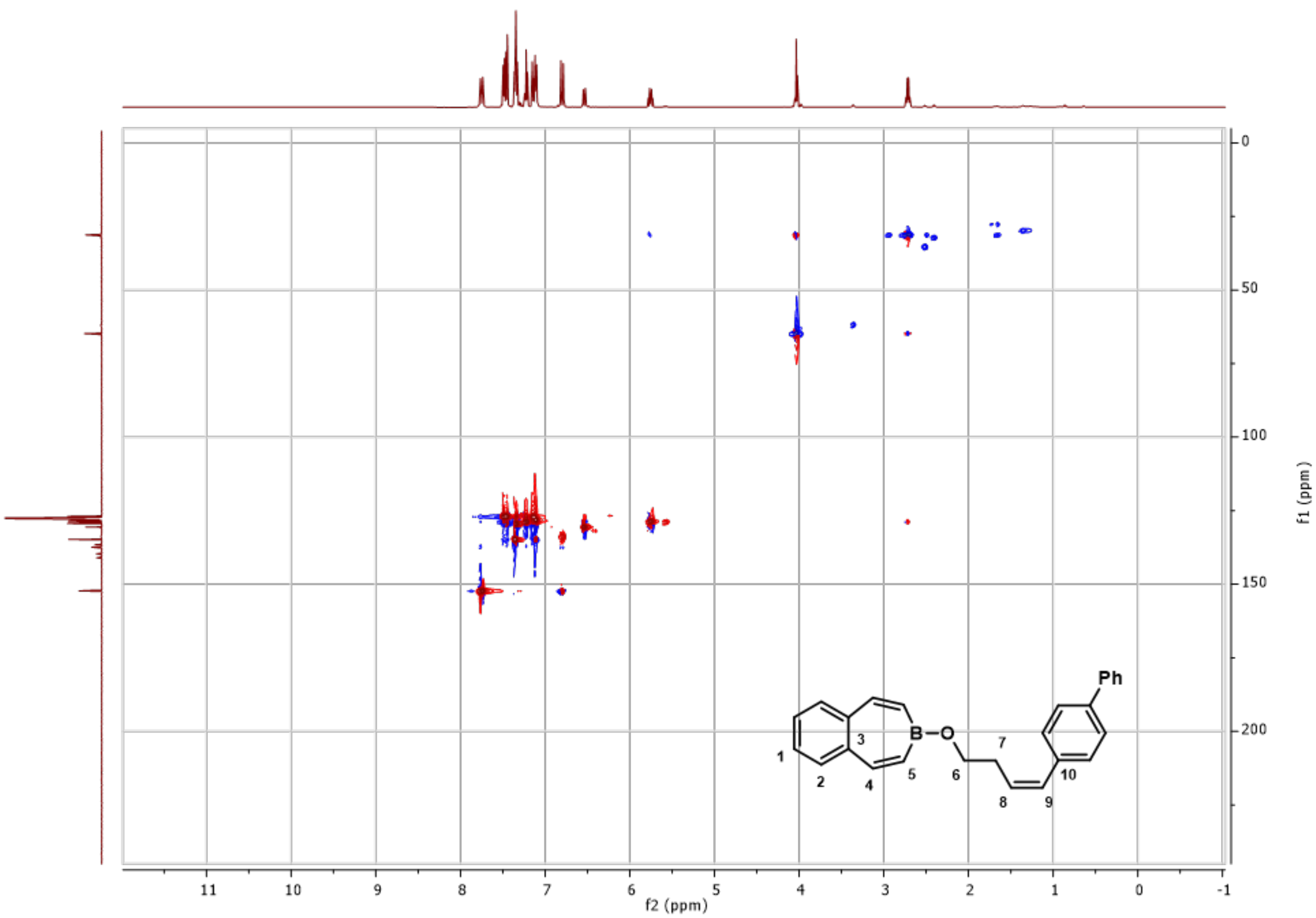

^{1}H -^{13}C HMBC (500 MHz, Benzene-d_6) of Compound 6c

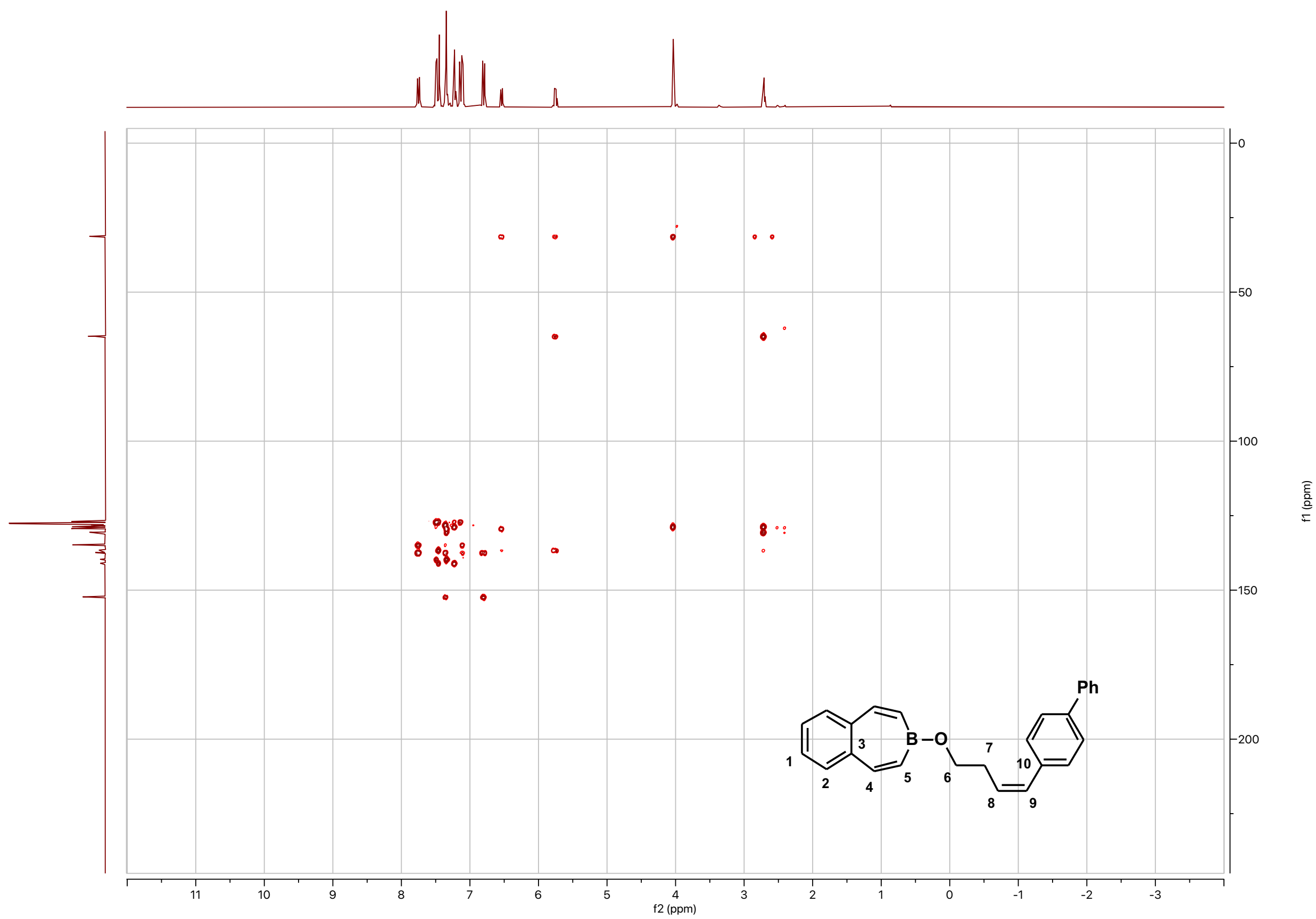

HRMS (ASAP) of Compound 6c

Elemental Composition Report **Page 1**

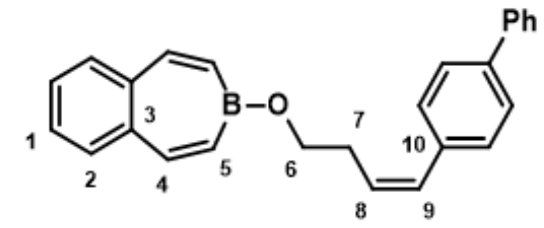

Chemical Formula: $C_{26}H_{23}BO$
Exact Mass: 362.1842

Single Mass Analysis
Tolerance = 5.0 PPM / DBE: min = -1.5, max = 500.0
Element prediction: Off
Number of isotope peaks used for i-FIT = 3

Monoisotopic Mass, Even Electron Ions
9 formula(e) evaluated with 1 results within limits (up to 50 closest results for each mass)
Elements Used:
C: 26-26 H: 20-30 B: 0-2 O: 1-3 Na: 0-1
DCM

TOF MS ASAP+
1.24e+007

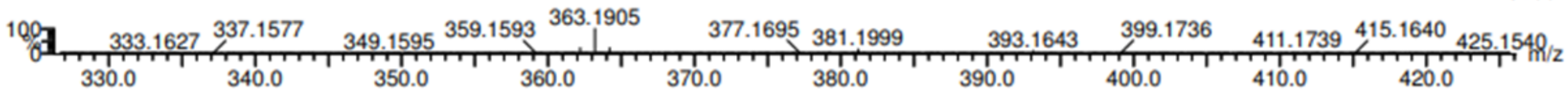

Mass	Calc. Mass	mDa	PPM	DBE	i-FIT	Norm	Conf(%)	Formula
Minimum:				-1.5				
Maximum:		5.0	5.0	500.0				
363.1905	363.1920	-1.5	-4.1	15.5	772.3	n/a	n/a	C26 H24 B O

^{1}H NMR (500 MHz, Benzene-d_6) of Compound 6d

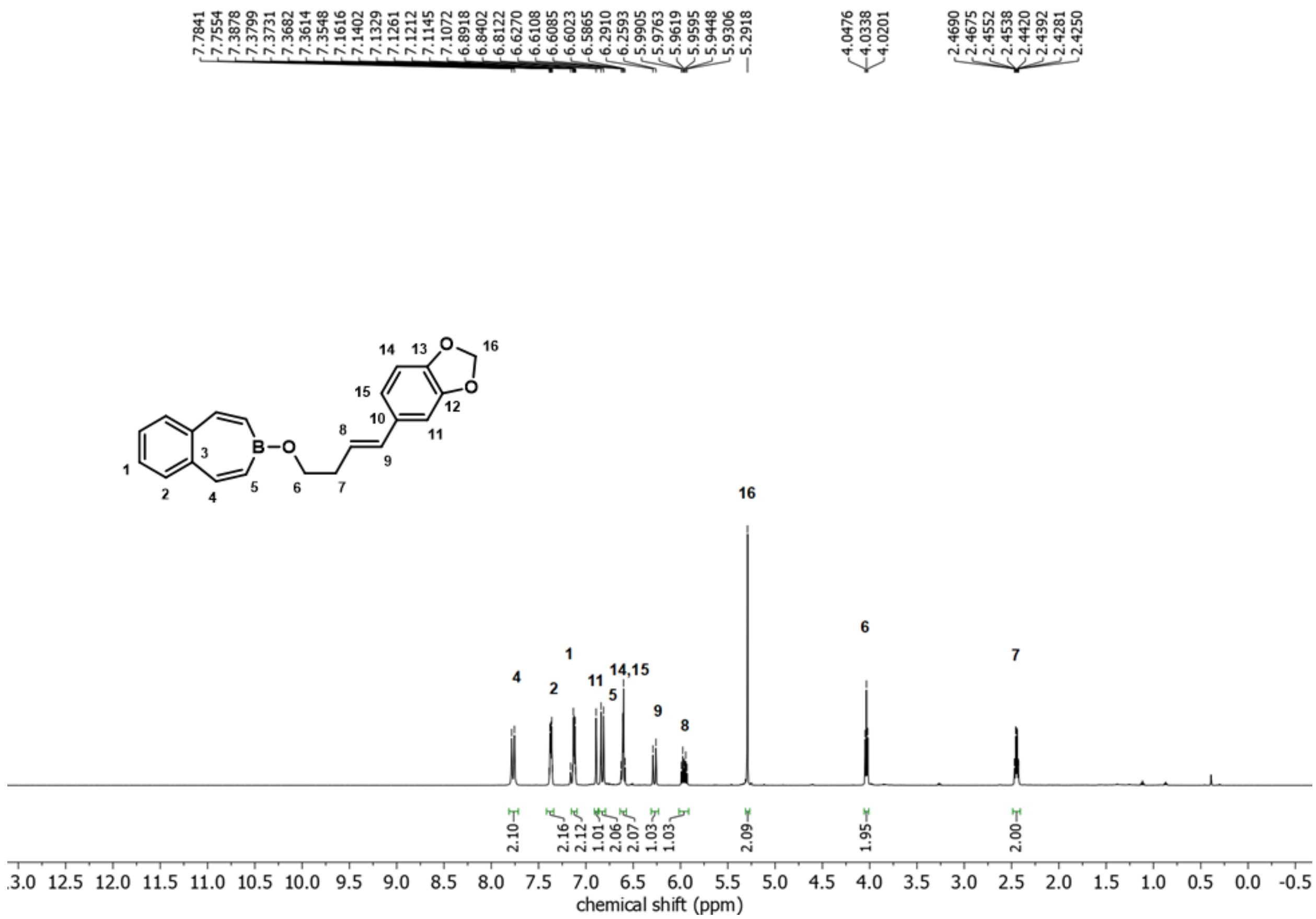

$^{13}C\{^1H\}$ NMR (126 MHz, Benzene-d_6) of Compound 6d

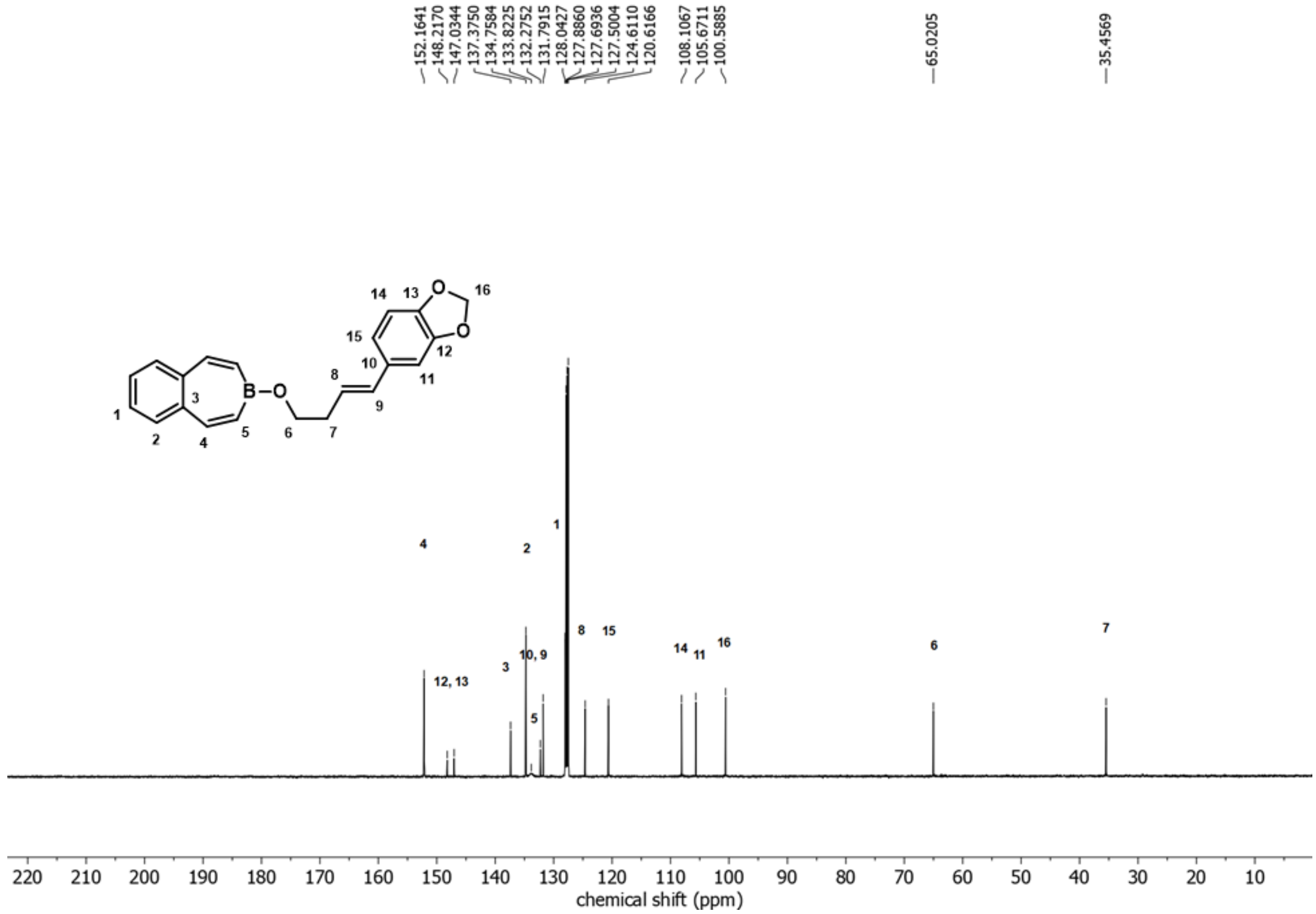

^{11}B NMR (160 MHz, Benzene-d_6) of Compound 6d

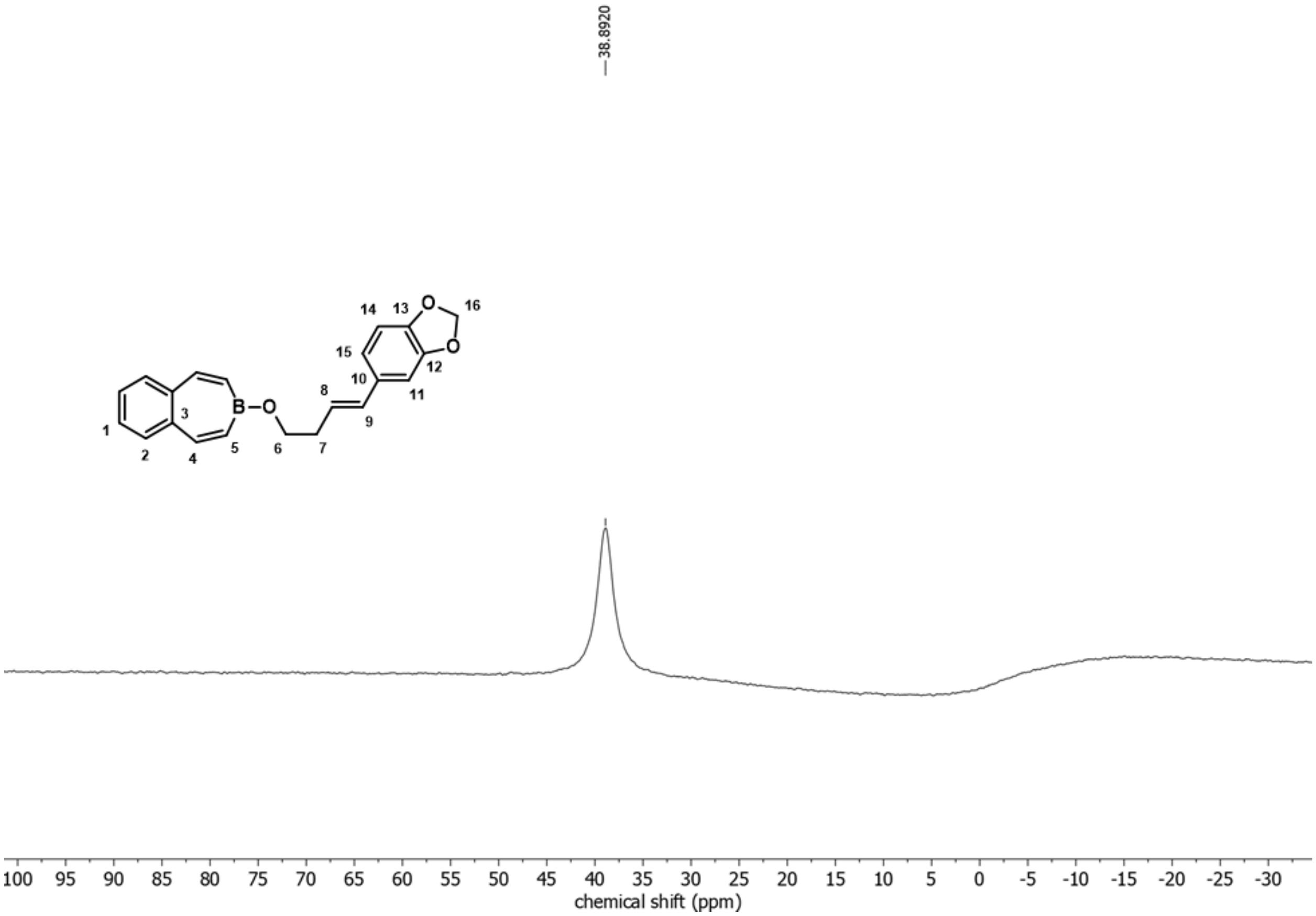

^{1}H -^{1}H COSY (500 MHz, Benzene-d_6) of Compound 6d

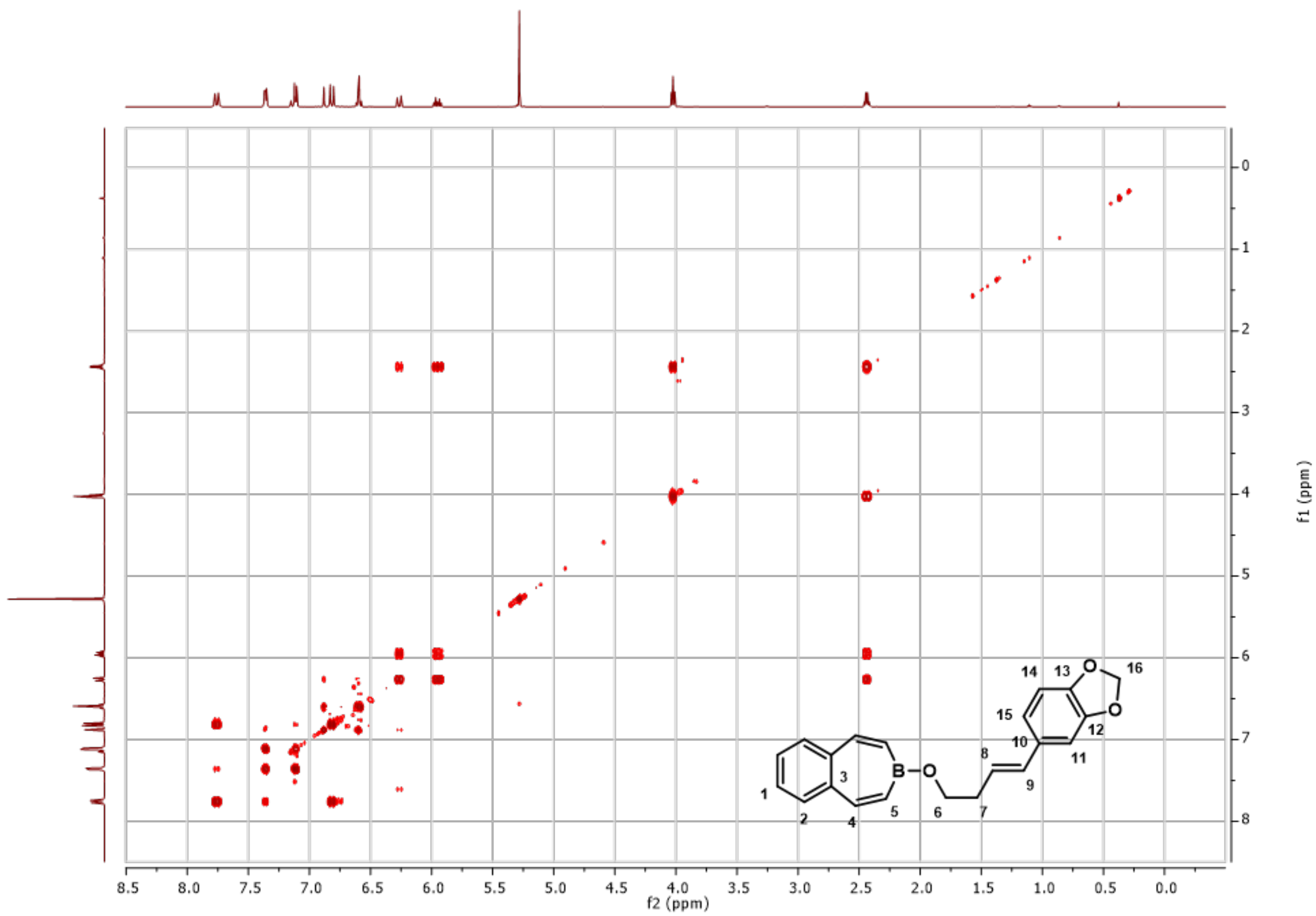

^{1}H -^{13}C HSQC (500 MHz, Benzene-d_6) of Compound 6d

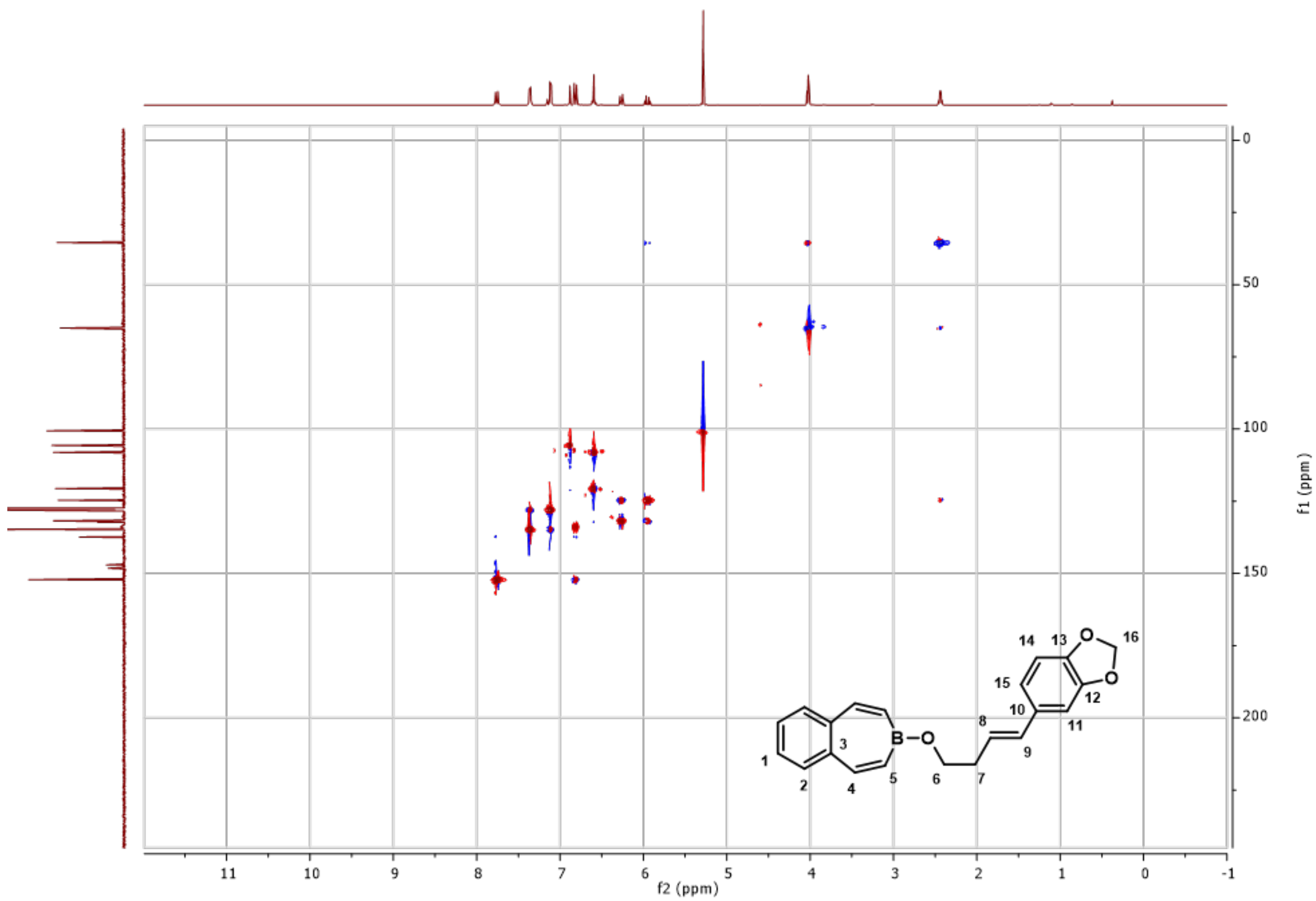

^{1}H -^{13}C HMBC (500 MHz, Benzene-d_6) of Compound 6d

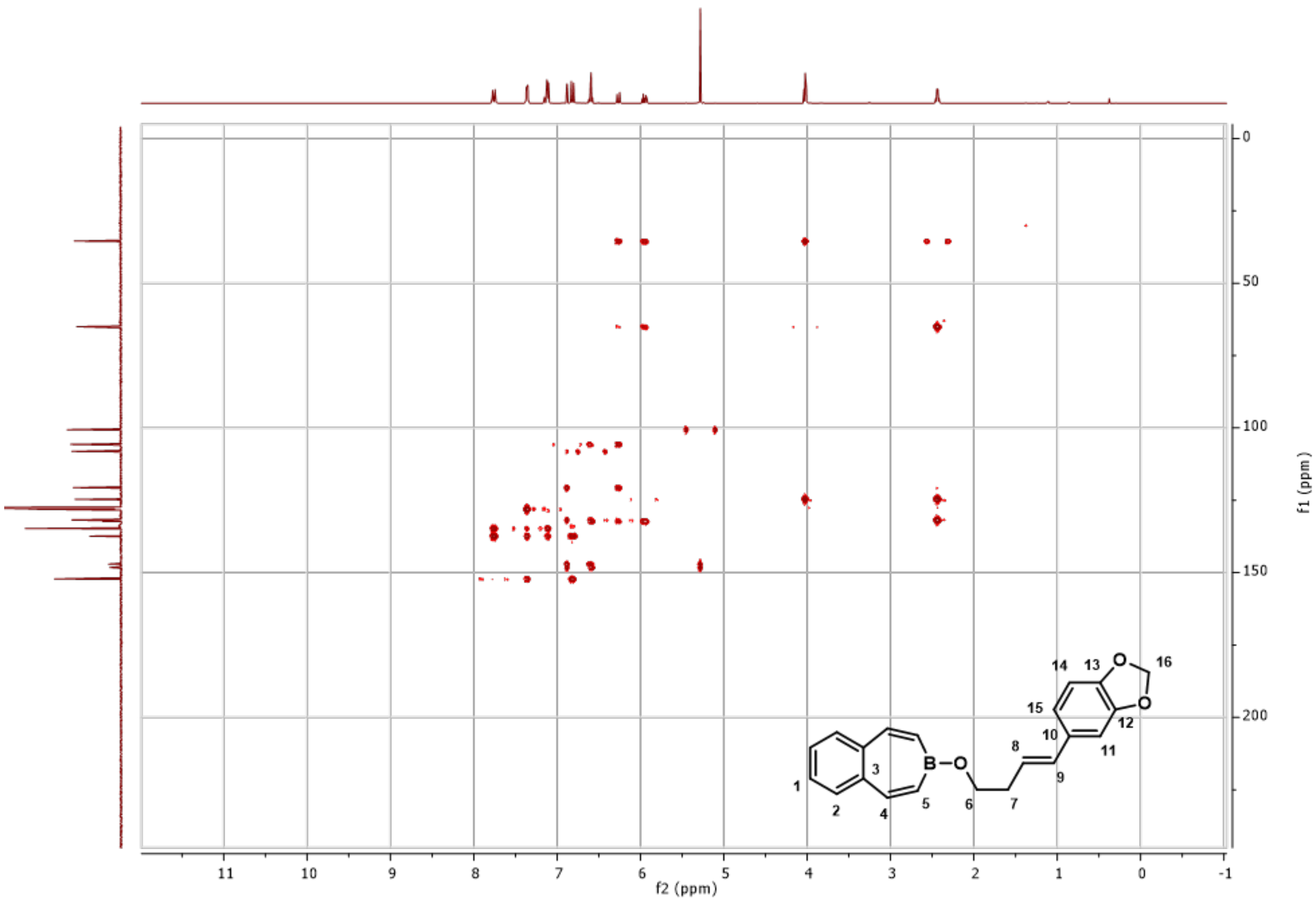

HRMS (ASAP) of Compound 6d

Elemental Composition Report **Page 1**

Single Mass Analysis
Tolerance = 5.0 PPM / DBE: min = -1.5, max = 500.0
Element prediction: Off

Monoisotopic Mass, Even Electron Ions
36 formula(e) evaluated with 1 results within limits (up to 50 closest results for each mass)
Elements Used:
C: 20-21 H: 10-25 B: 0-1 N: 0-6 O: 0-3
DCM

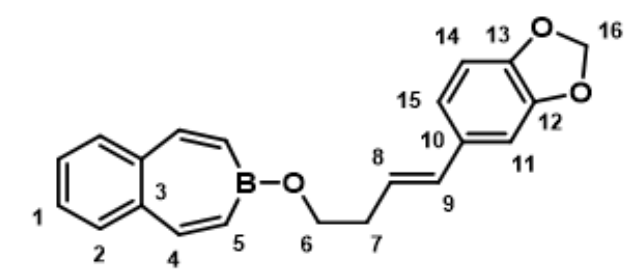

Chemical Formula: $C_{21}H_{19}BO_3$
Exact Mass: 330.1427

TOF MS ASAP+
2.53e+007

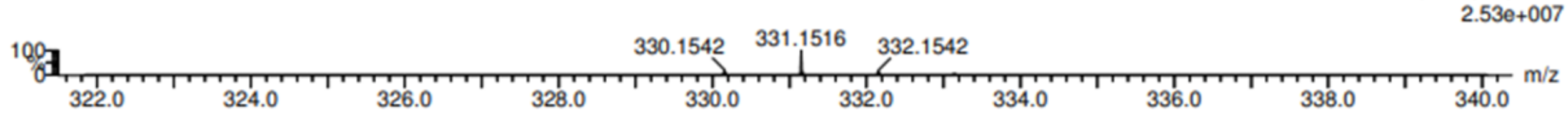

Minimum:				-1.5	
Maximum:		5.0	5.0	500.0	
Mass	Calc. Mass	mDa	PPM	DBE	Formula
331.1516	331.1506	1.0	3.0	12.5	C21 H20 B O3

^{1}H NMR (500 MHz, Benzene-d_6) of Compound 6e

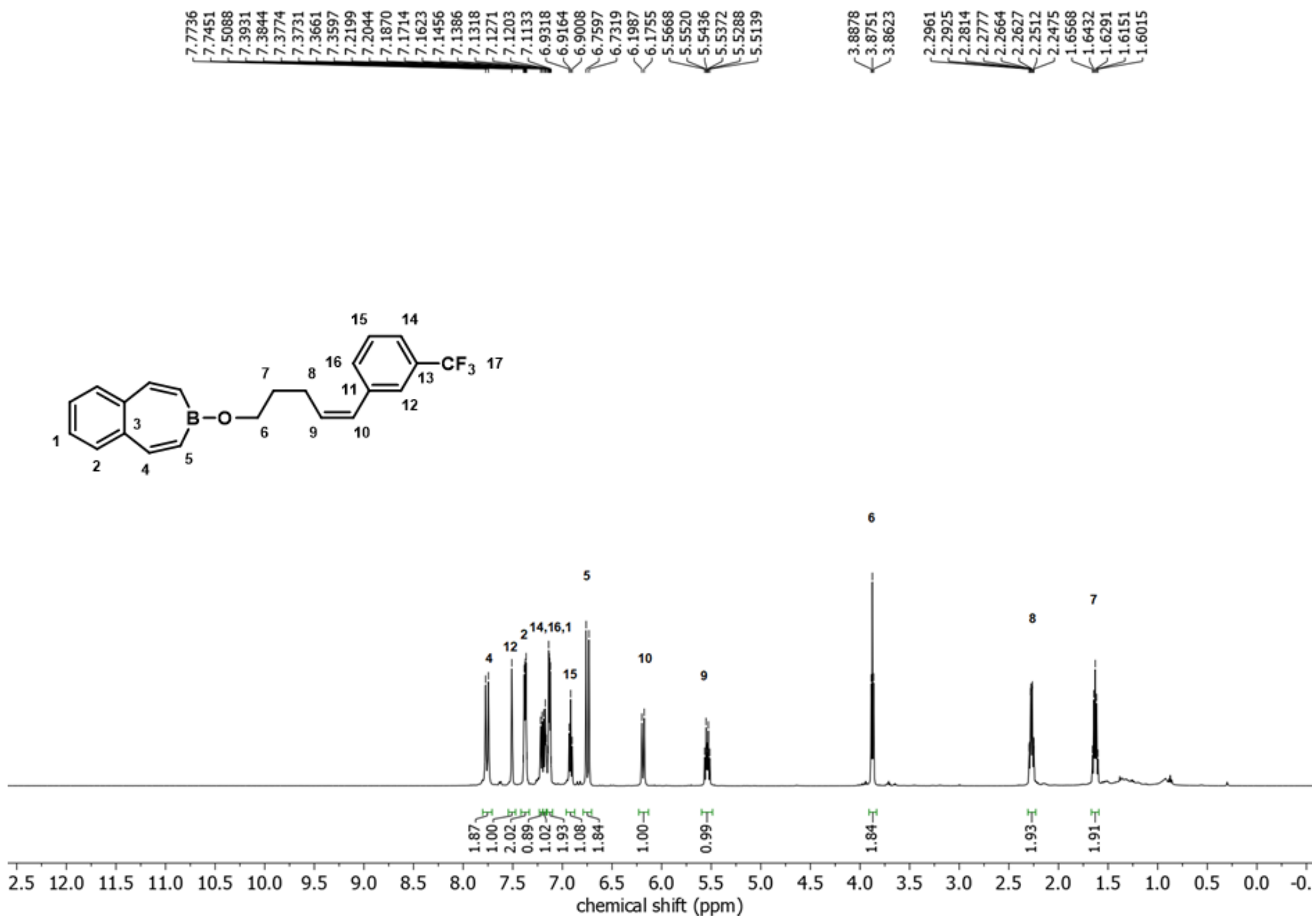

$^{13}C\{^1H\}$ **NMR (126 MHz, Benzene-d_6) of Compound 6e**

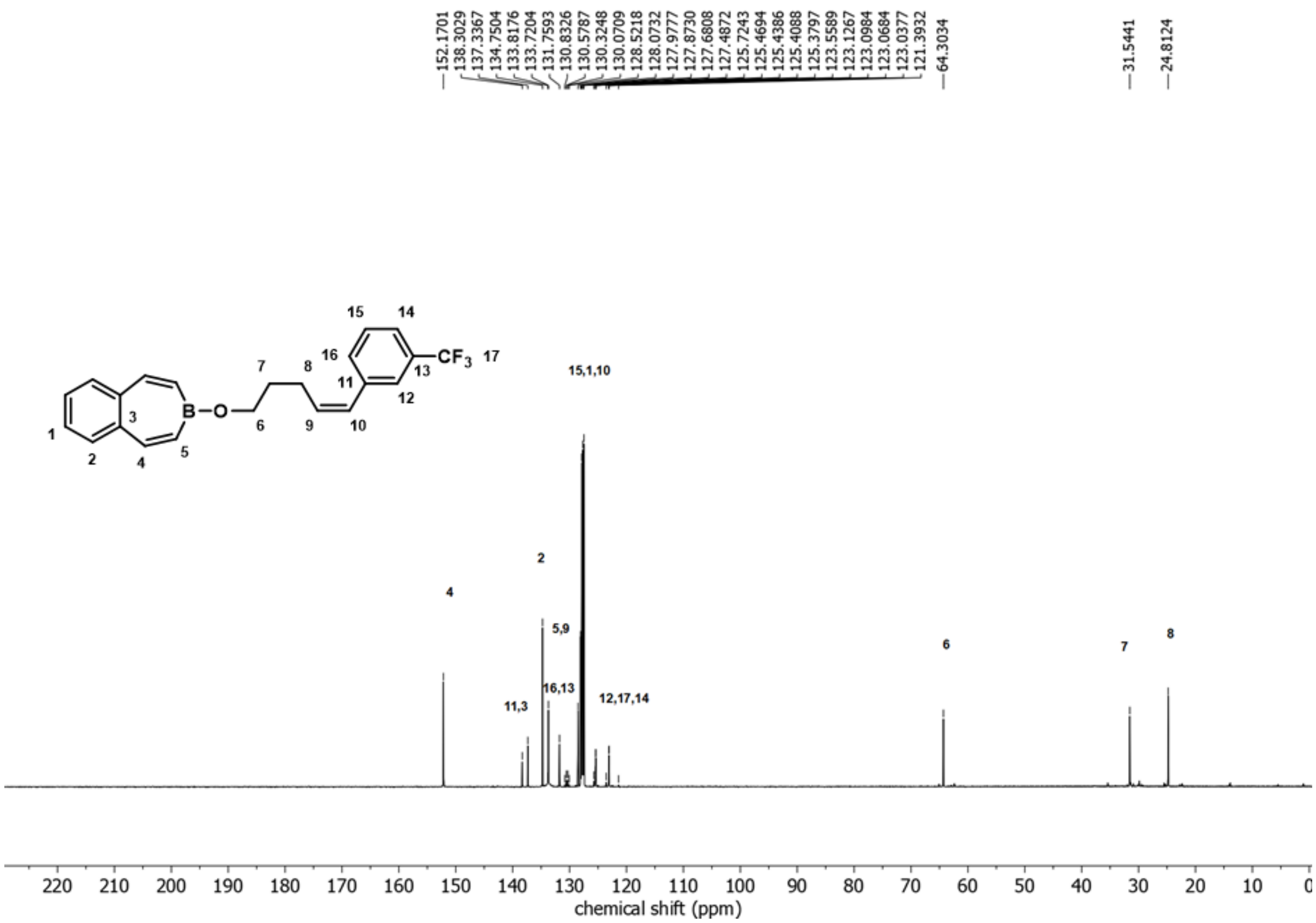

^{19}F NMR (471 MHz, Benzene-d_6) of Compound 6e

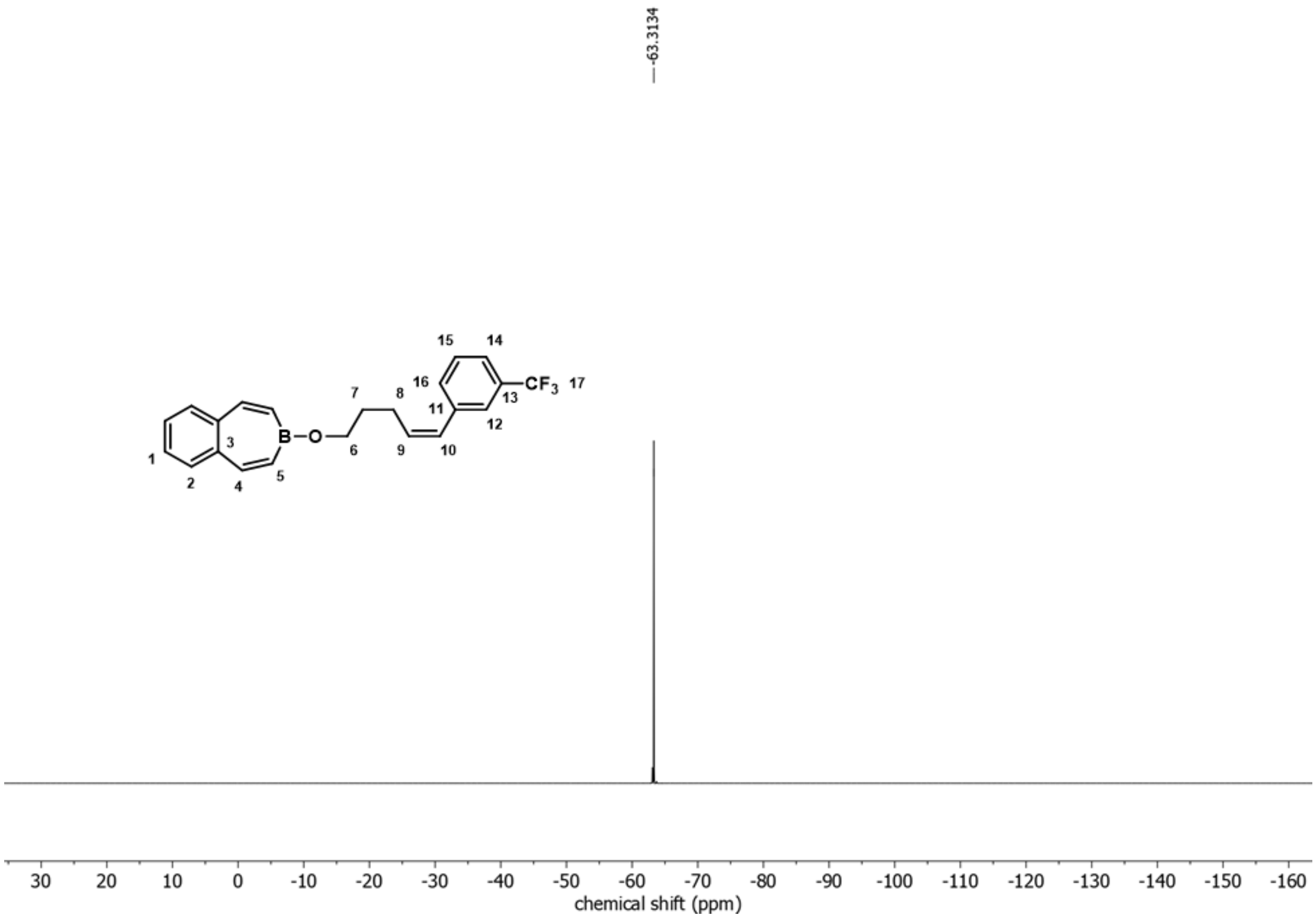

^{11}B NMR (160 MHz, Benzene-d_6) of Compound 6e

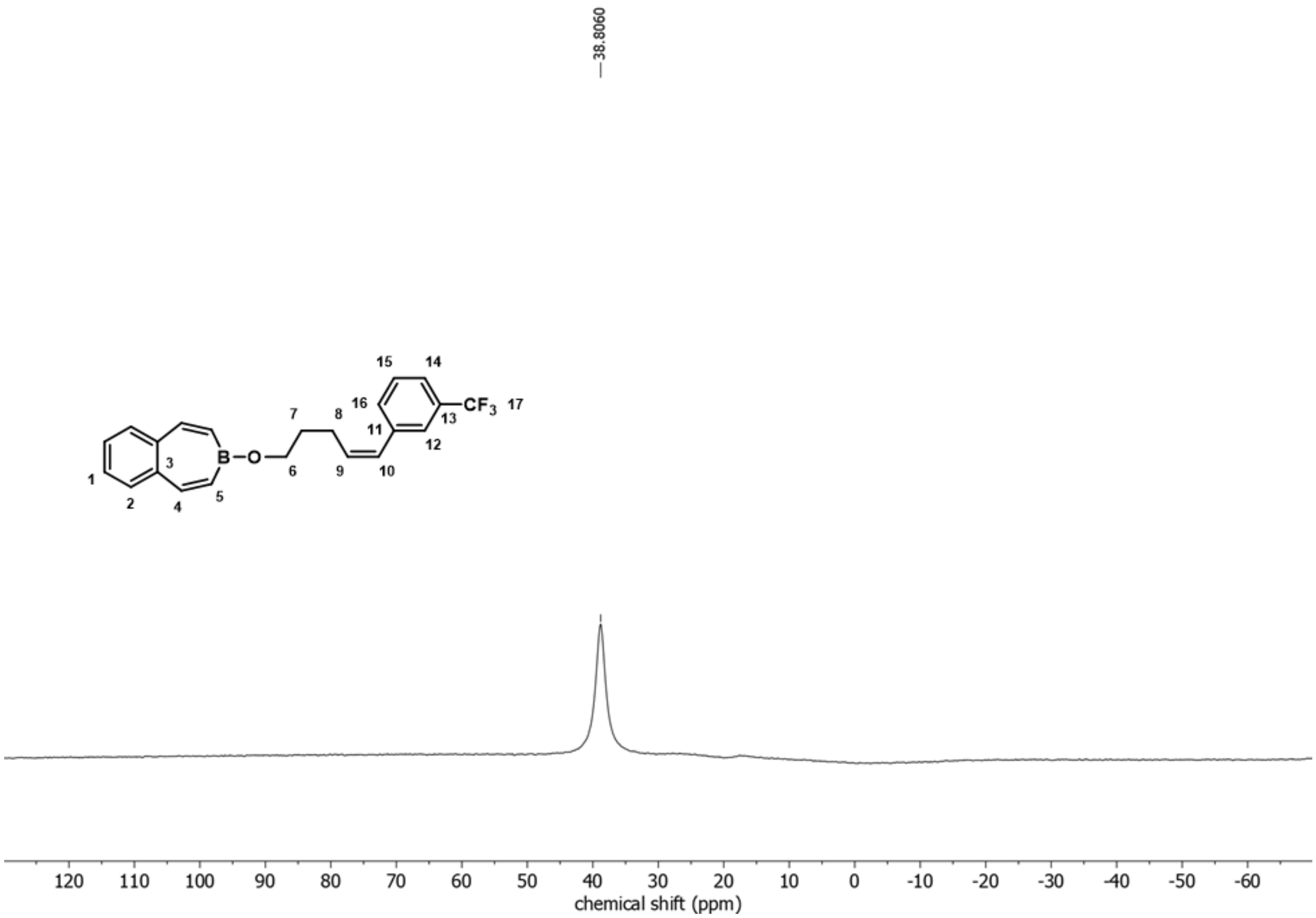

^1H -^1H COSY (500 MHz, Benzene-d_6) of Compound 6e

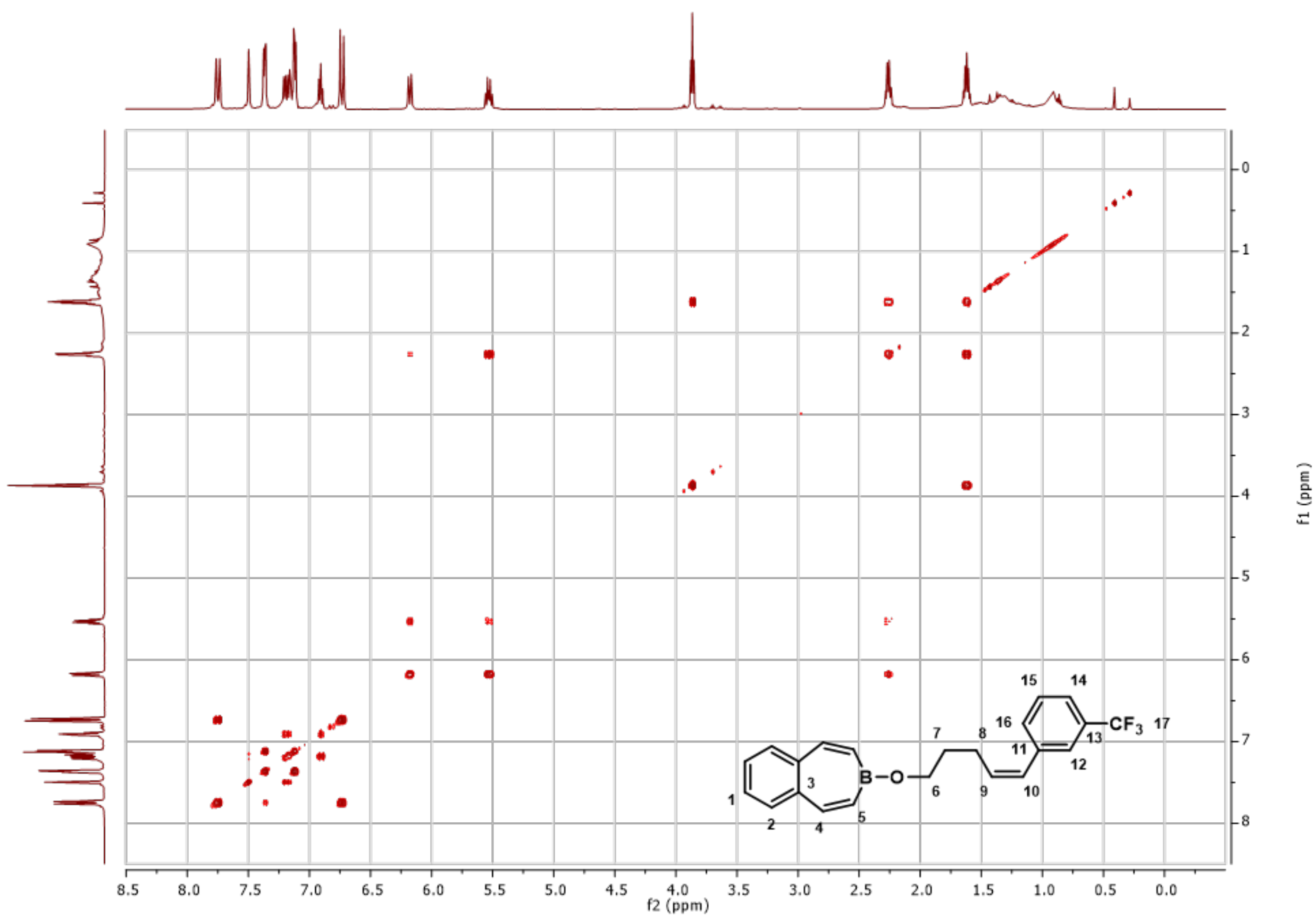

^{1}H -^{13}C HSQC (500 MHz, Benzene-d_6) of Compound 6e

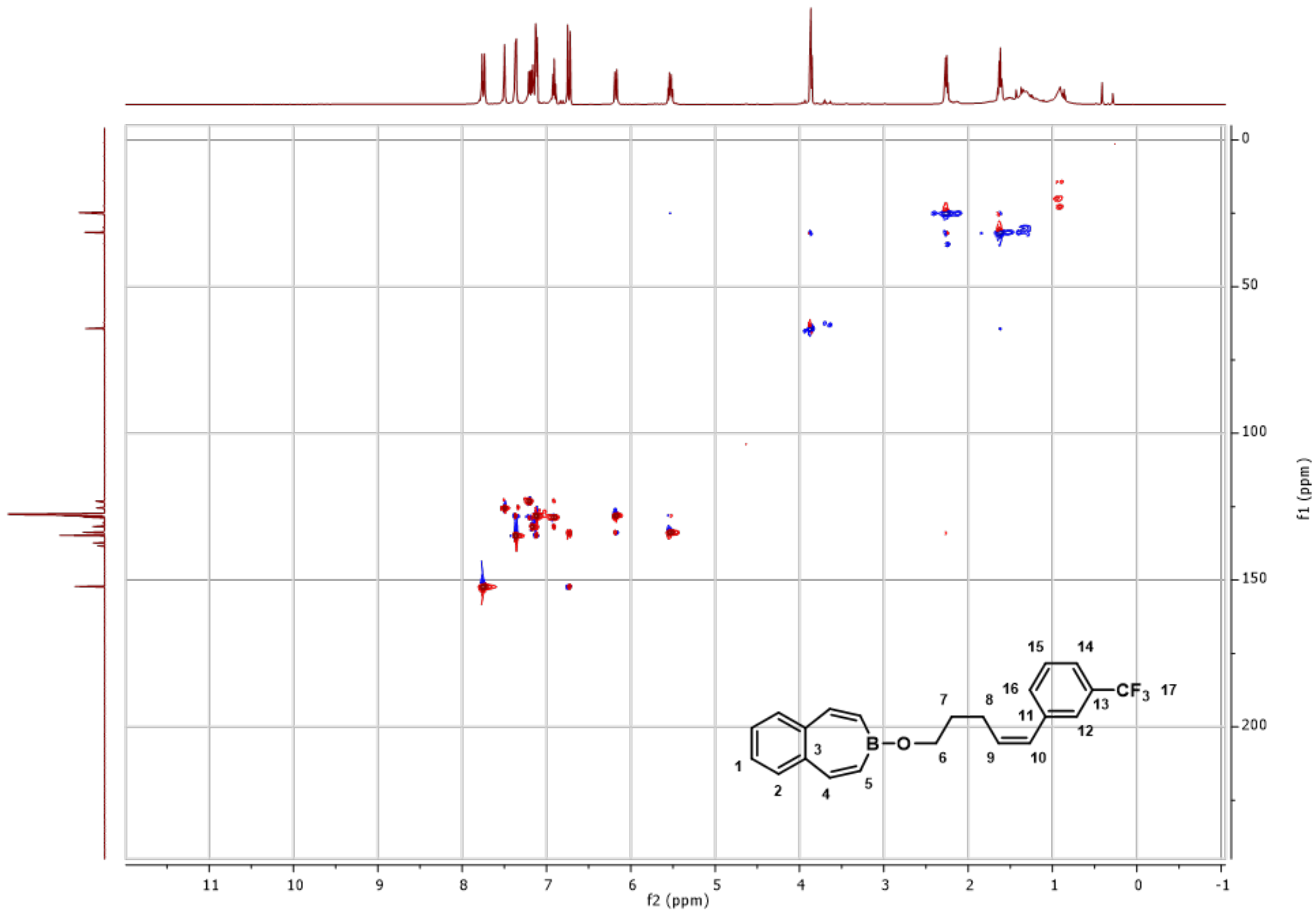

^1H -^{13}C HMBC (500 MHz, Benzene-d_6) of Compound 6e

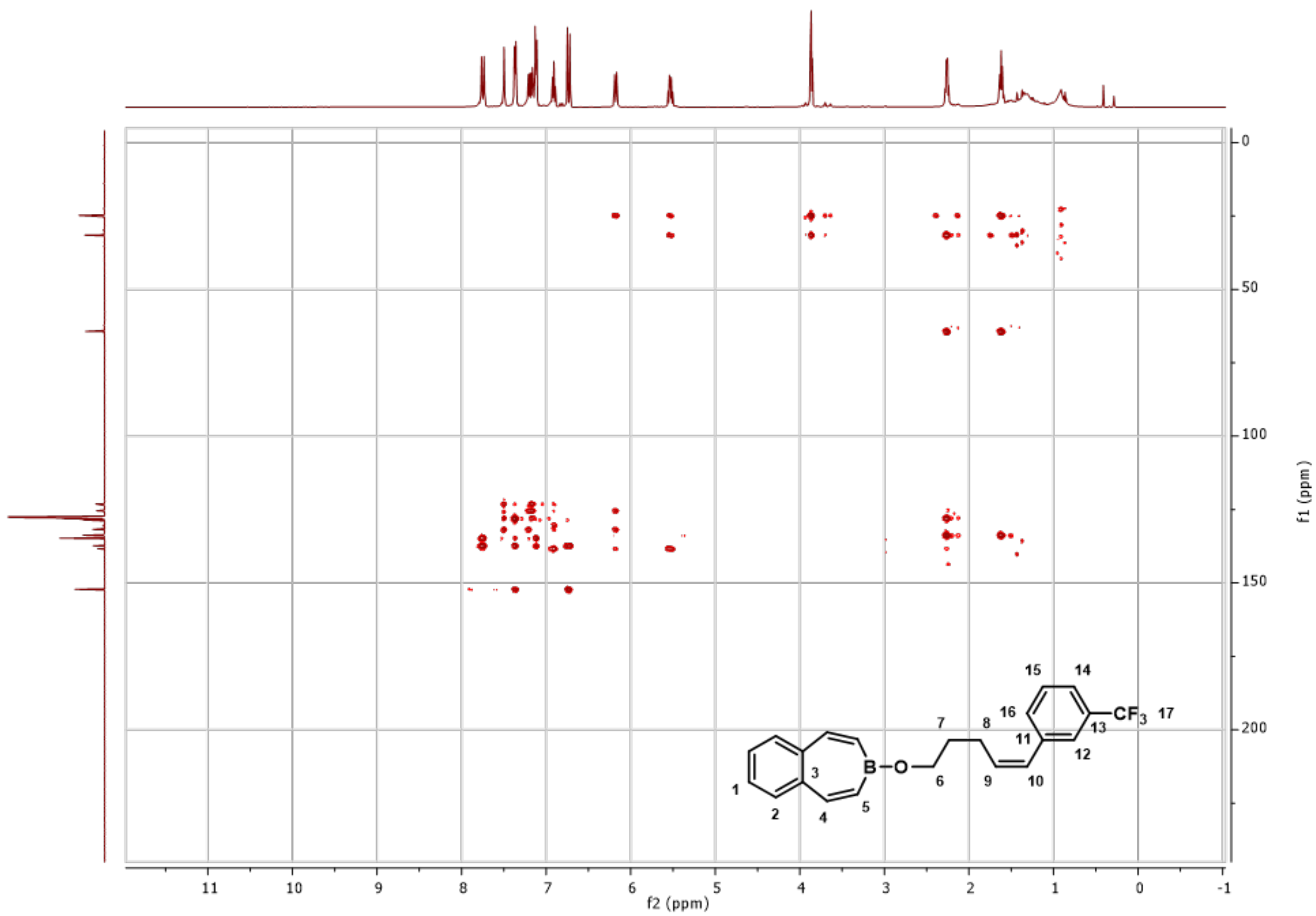

HRMS (ASAP) of Compound 6e

Elemental Composition Report

Page 1

Single Mass Analysis
Tolerance = 50.0 PPM / DBE: min = -1.5, max = 500.0
Element prediction: Off
Number of isotope peaks used for i-FIT = 3

Monoisotopic Mass, Even Electron Ions
19 formula(e) evaluated with 1 results within limits (up to 50 closest results for each mass)
Elements Used:
C: 22-22 H: 10-60 B: 0-2 O: 1-1 F: 0-6
DCM

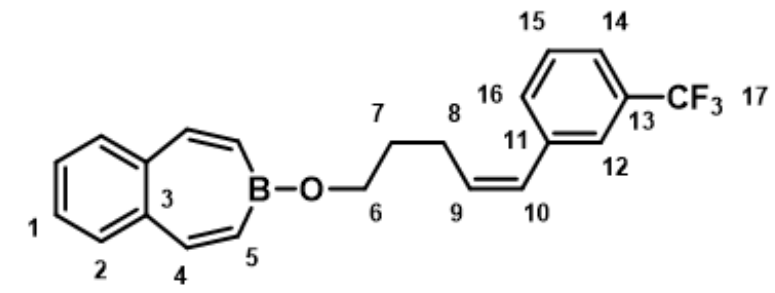

Chemical Formula: $C_{22}H_{20}BF_3O$
Exact Mass: 368.1559

TOF MS ASAP+
3.08e+008

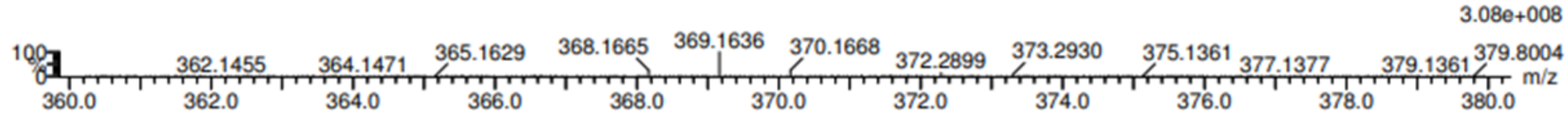

Minimum:				-1.5				
Maximum:		5.0	50.0	500.0				
Mass	Calc. Mass	mDa	PPM	DBE	i-FIT	Norm	Conf(%)	Formula
369.1636	369.1638	-0.2	-0.5	11.5	997.7	n/a	n/a	C22 H21 B O F3

^{1}H NMR (500 MHz, Benzene-d_6) of Compound 6f

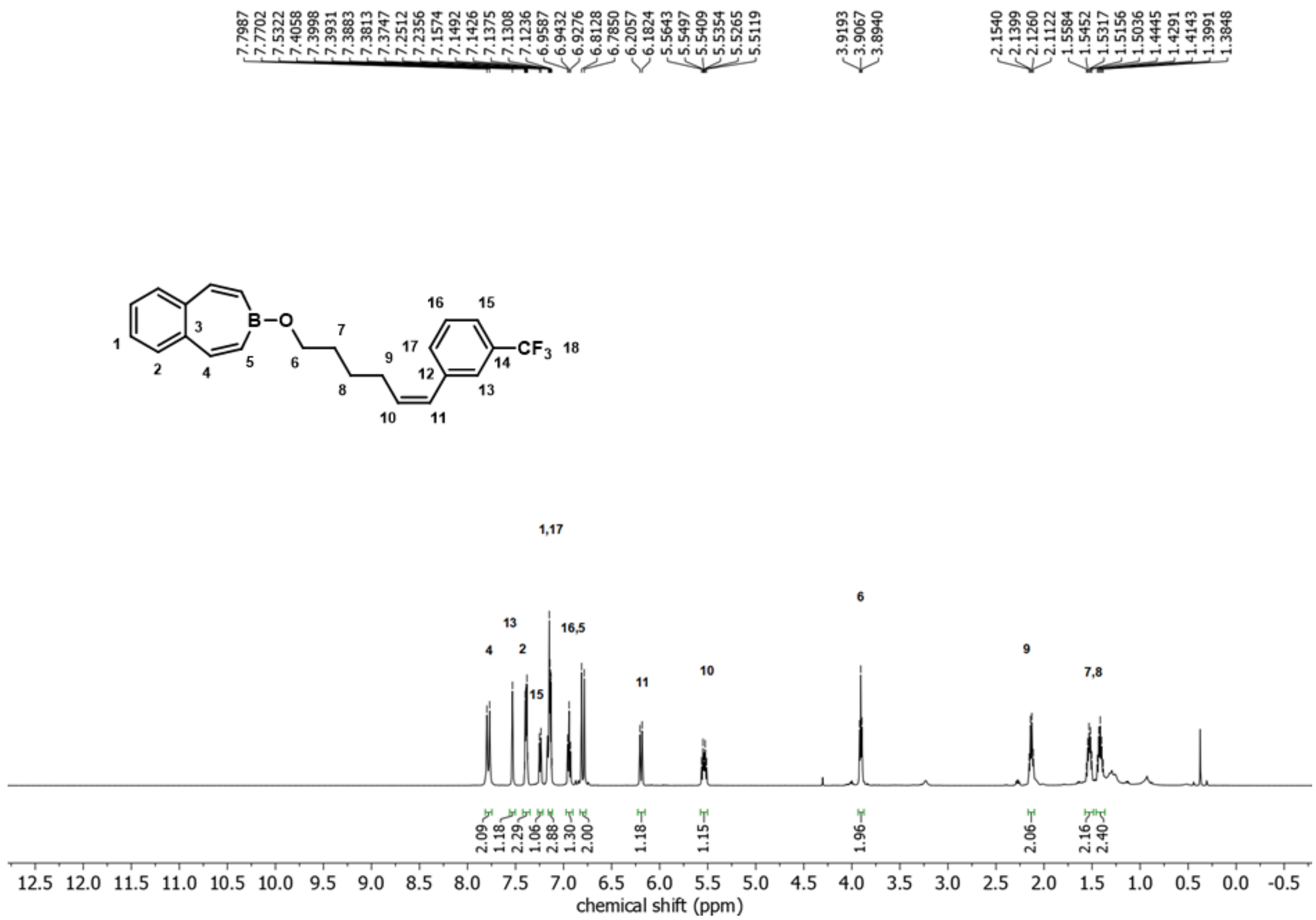

$^{13}C\{^1H\}$ NMR (126 MHz, Benzene-d_6) of Compound 6f

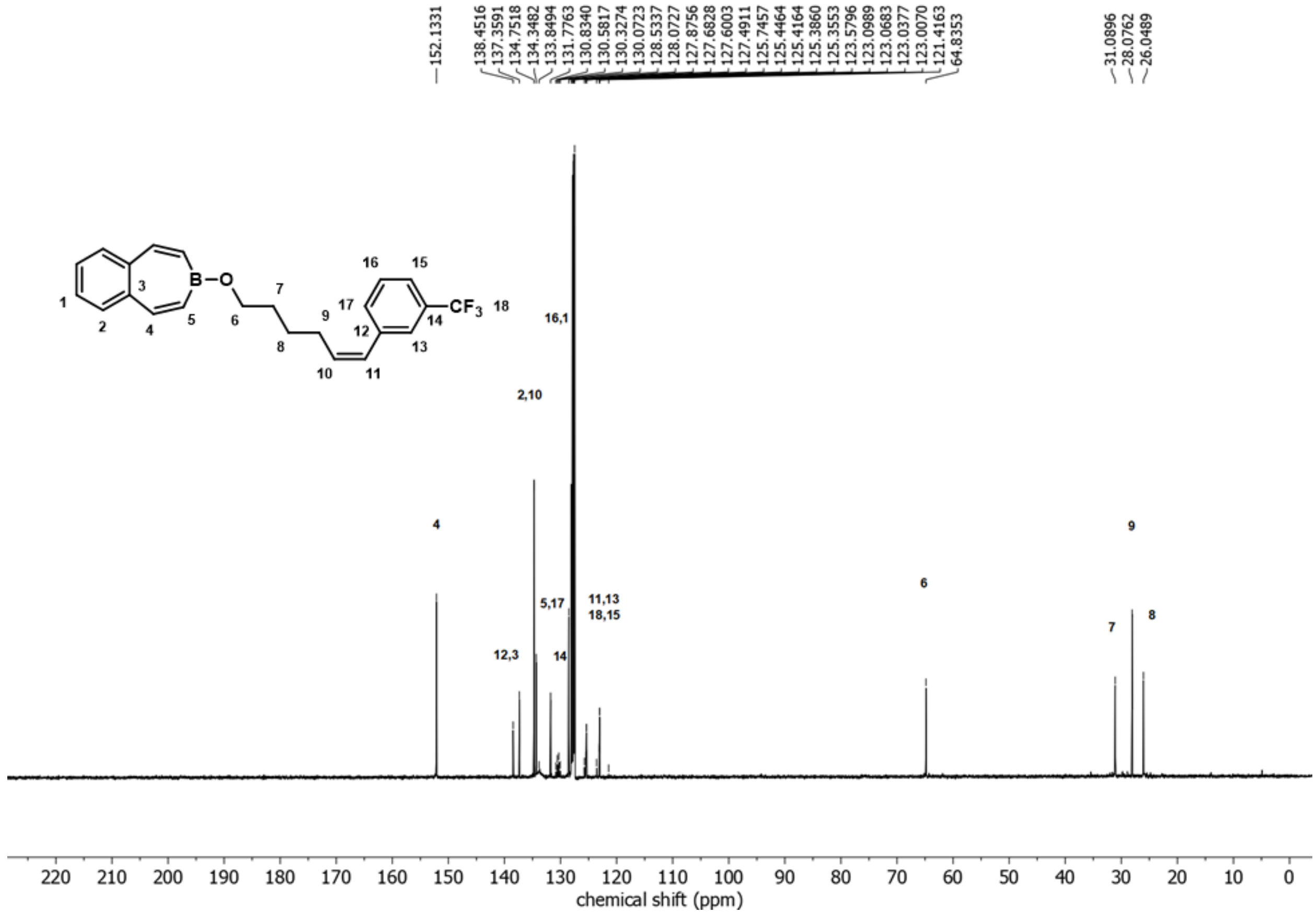

^{19}F NMR (471 MHz, Benzene-d_6) of Compound 6f

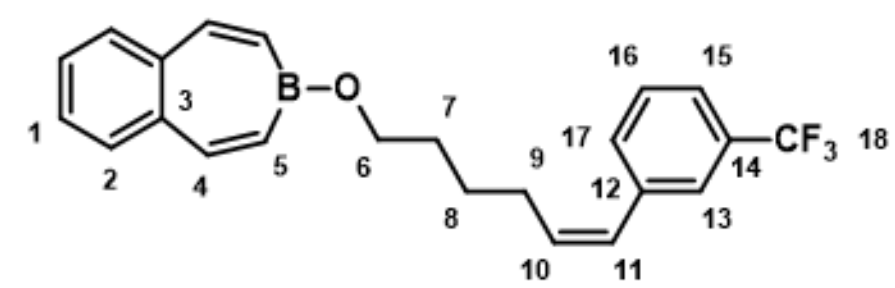

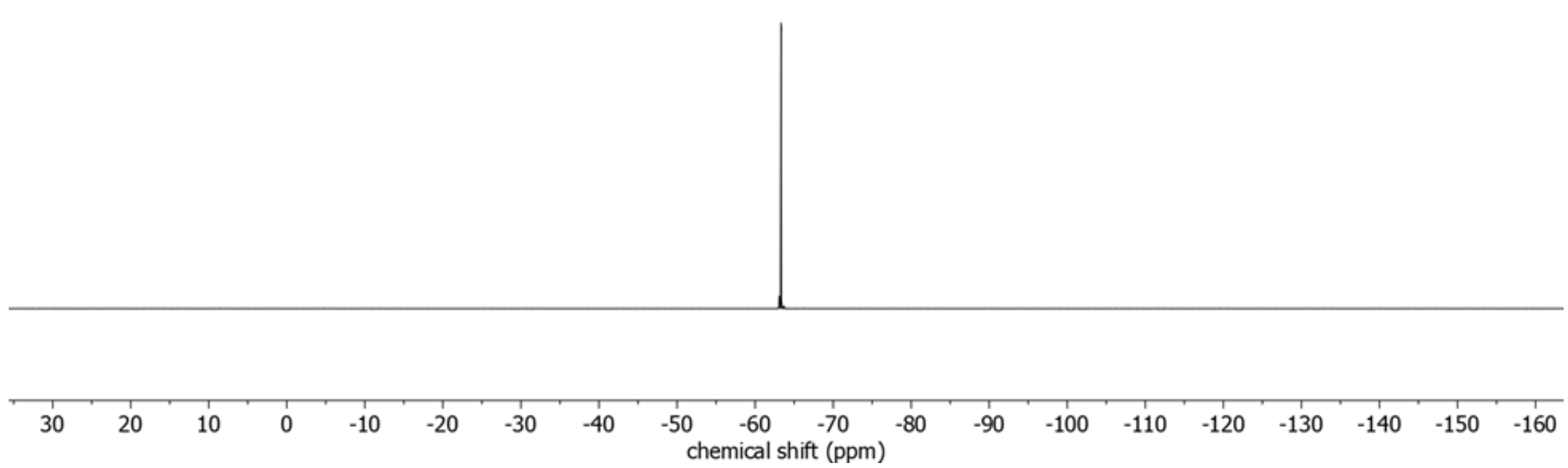

^{11}B NMR (160 MHz, Benzene-d_6) of Compound 6f

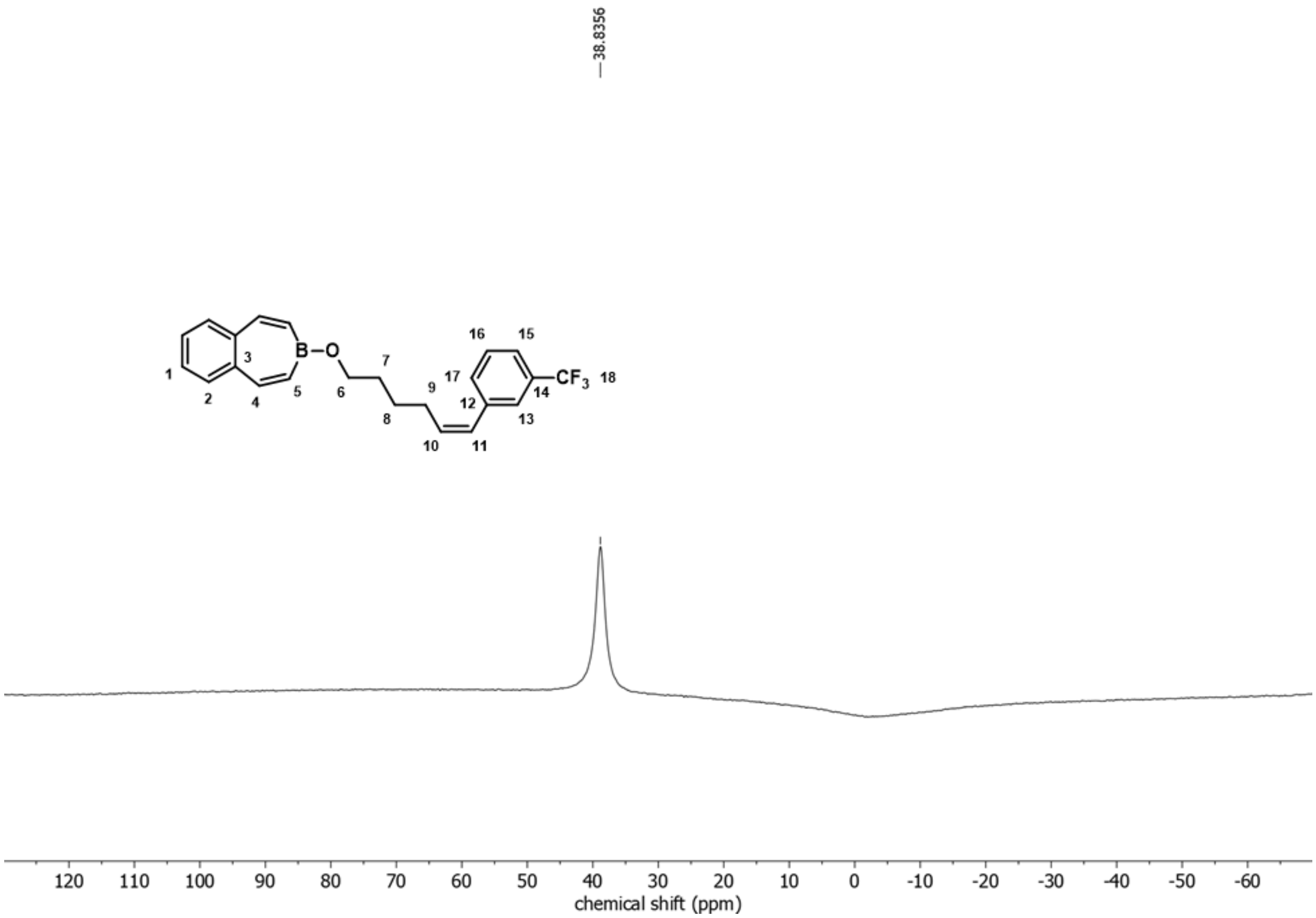

^{1}H -^{1}H COSY (500 MHz, Benzene-d_6) of Compound 6f

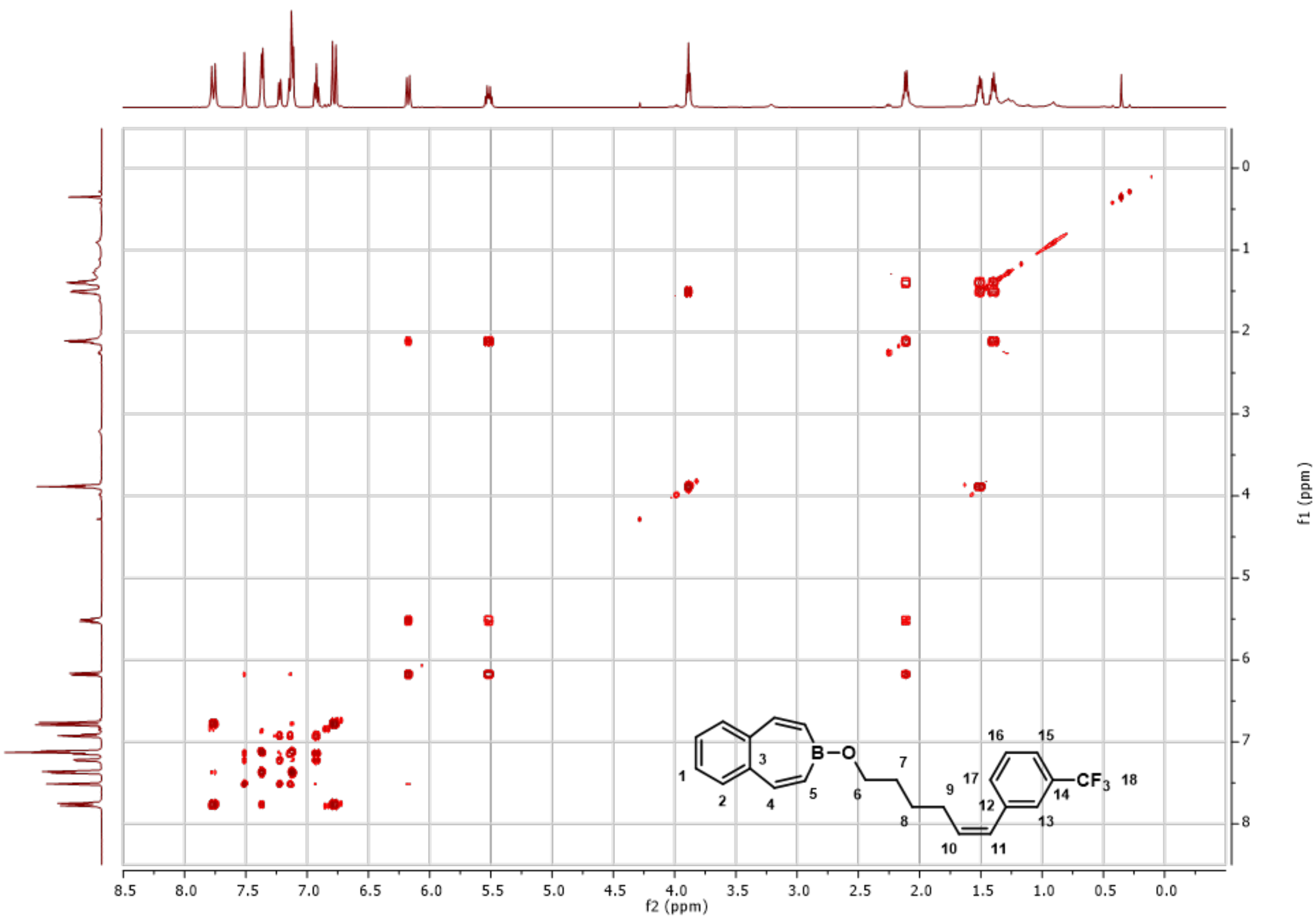

^{1}H -^{13}C HSQC (500 MHz, Benzene-d_6) of Compound 6f

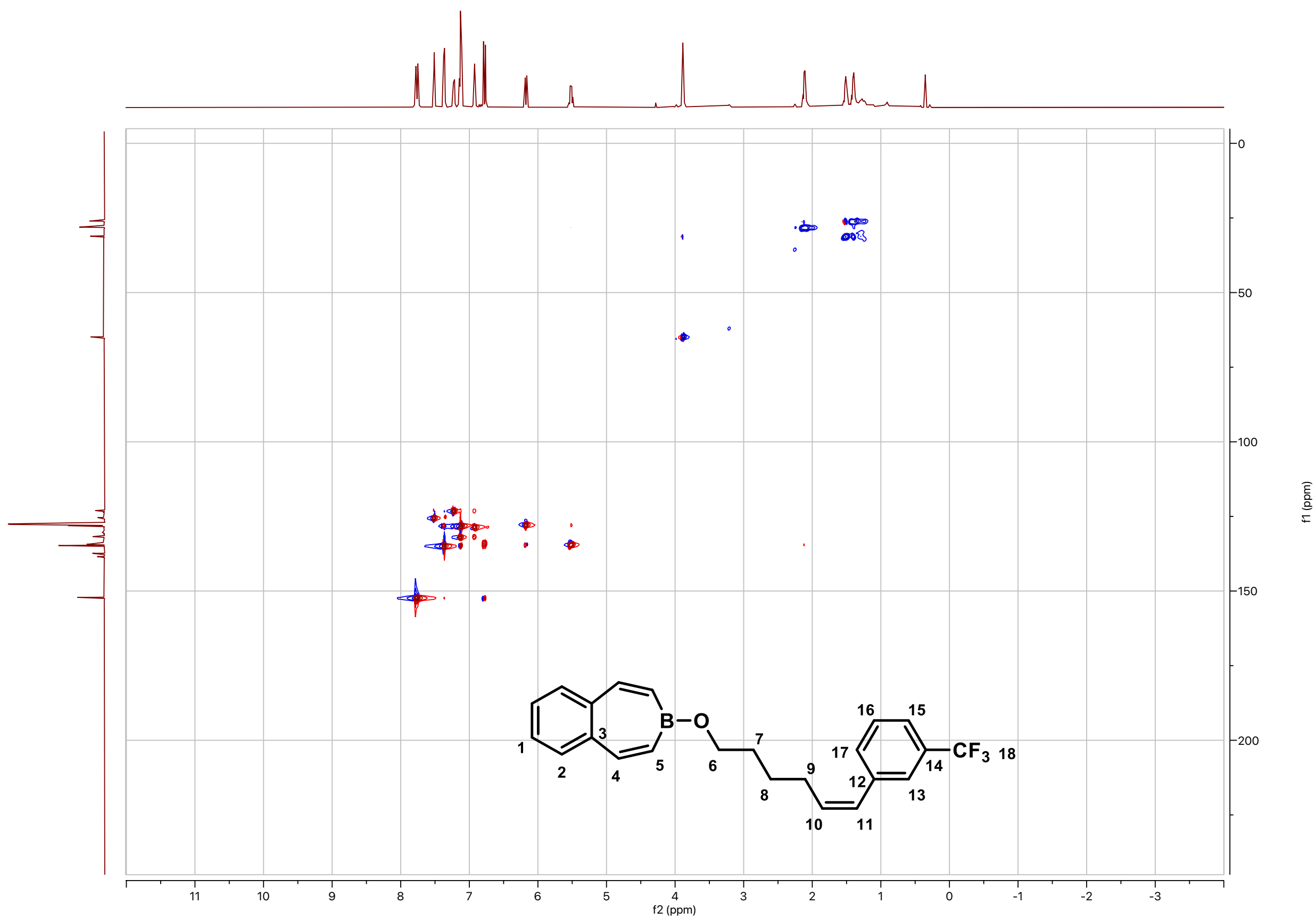

^{1}H -^{13}C HMBC (500 MHz, Benzene-d_6) of Compound 6f

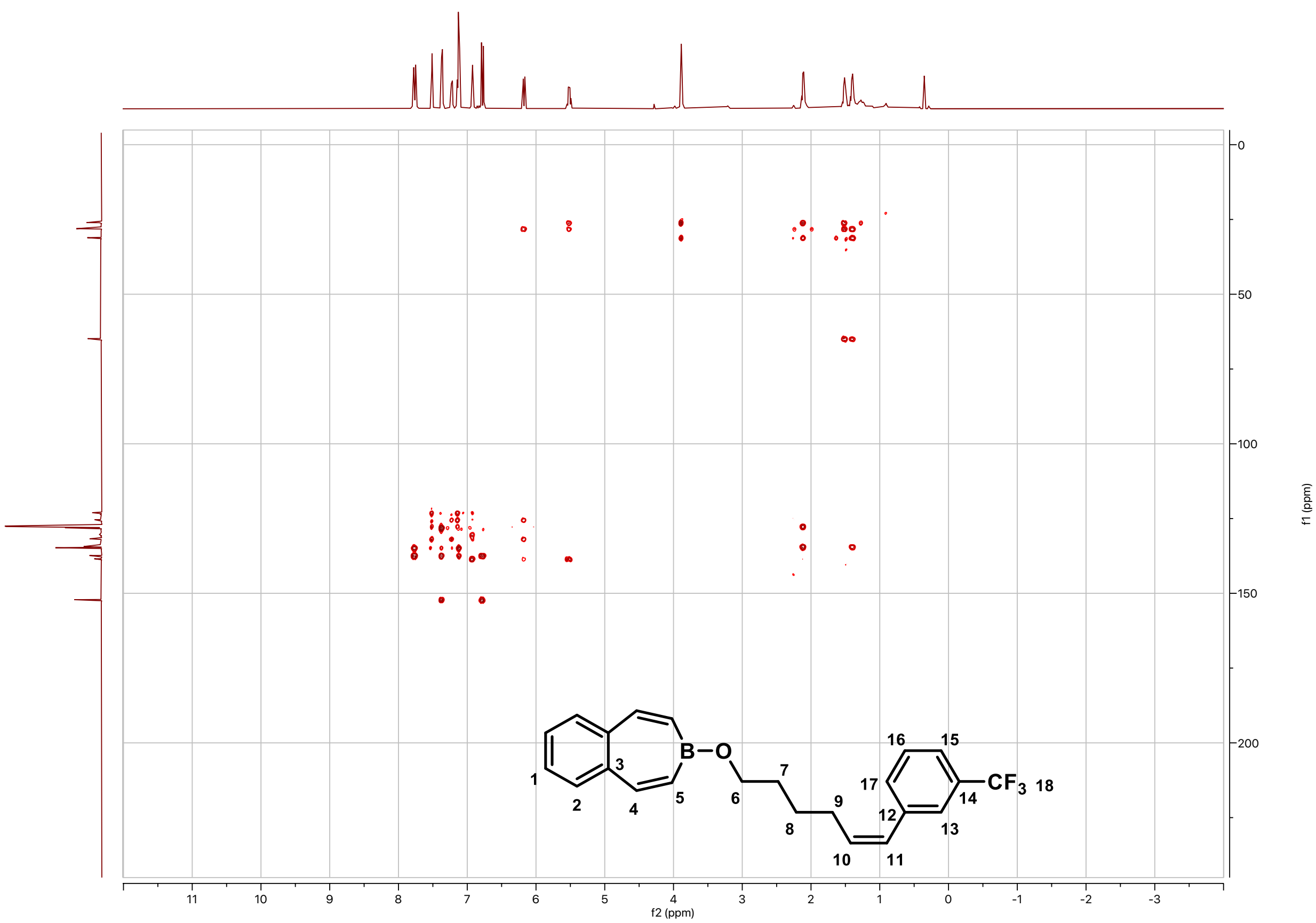

HRMS (ASAP) of Compound 6f

Elemental Composition Report **Page 1**

Single Mass Analysis
Tolerance = 5.0 PPM / DBE: min = -1.5, max = 500.0
Element prediction: Off
Number of isotope peaks used for i-FIT = 3

Monoisotopic Mass, Even Electron Ions
4 formula(e) evaluated with 1 results within limits (up to 50 closest results for each mass)
Elements Used:
C: 23-32 H: 20-50 B: 0-2 O: 1-1 F: 3-3 Na: 0-1
DCM

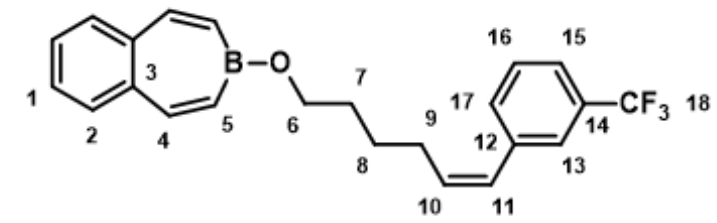

Chemical Formula: $C_{23}H_{22}BF_3O$
Exact Mass: 382.1716

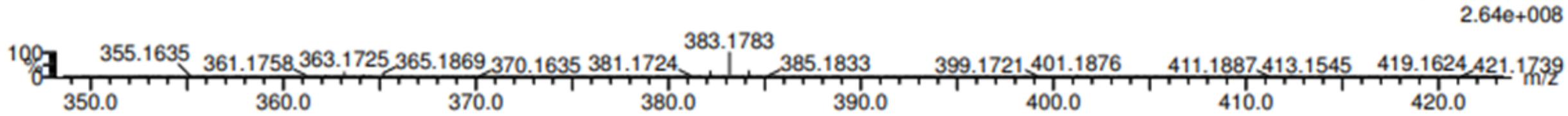

Minimum:				-1.5				
Maximum:		5.0	5.0	500.0				
Mass	Calc. Mass	mDa	PPM	DBE	i-FIT	Norm	Conf(%)	Formula
383.1783	383.1794	-1.1	-2.9	11.5	1056.3	n/a	n/a	C23 H23 B O F3

^{1}H NMR (500 MHz, Benzene-d_6) of Compound 6g

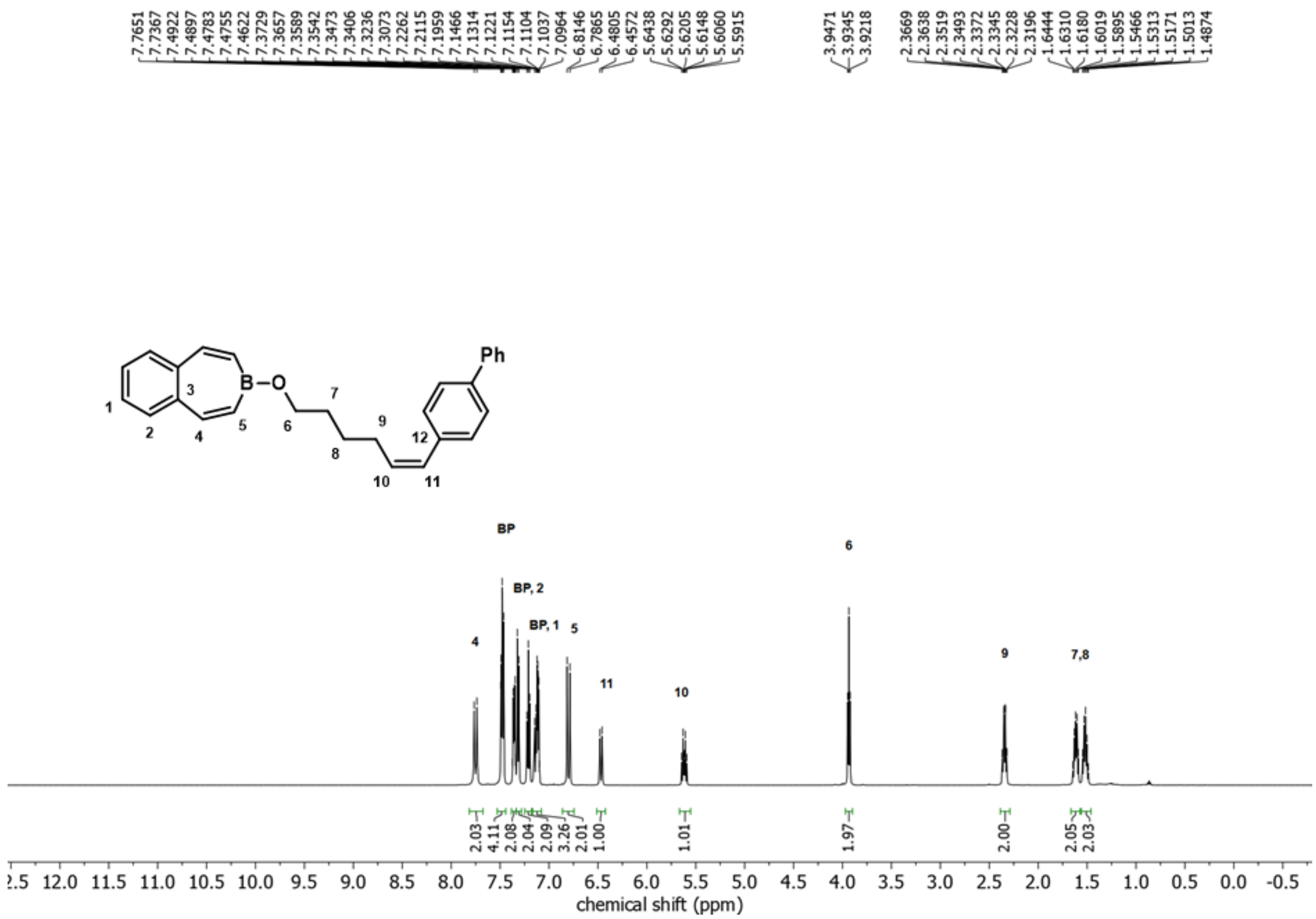

$^{13}C\{^{1}H\}$ NMR (126 MHz, Benzene-d_6) of Compound 6g

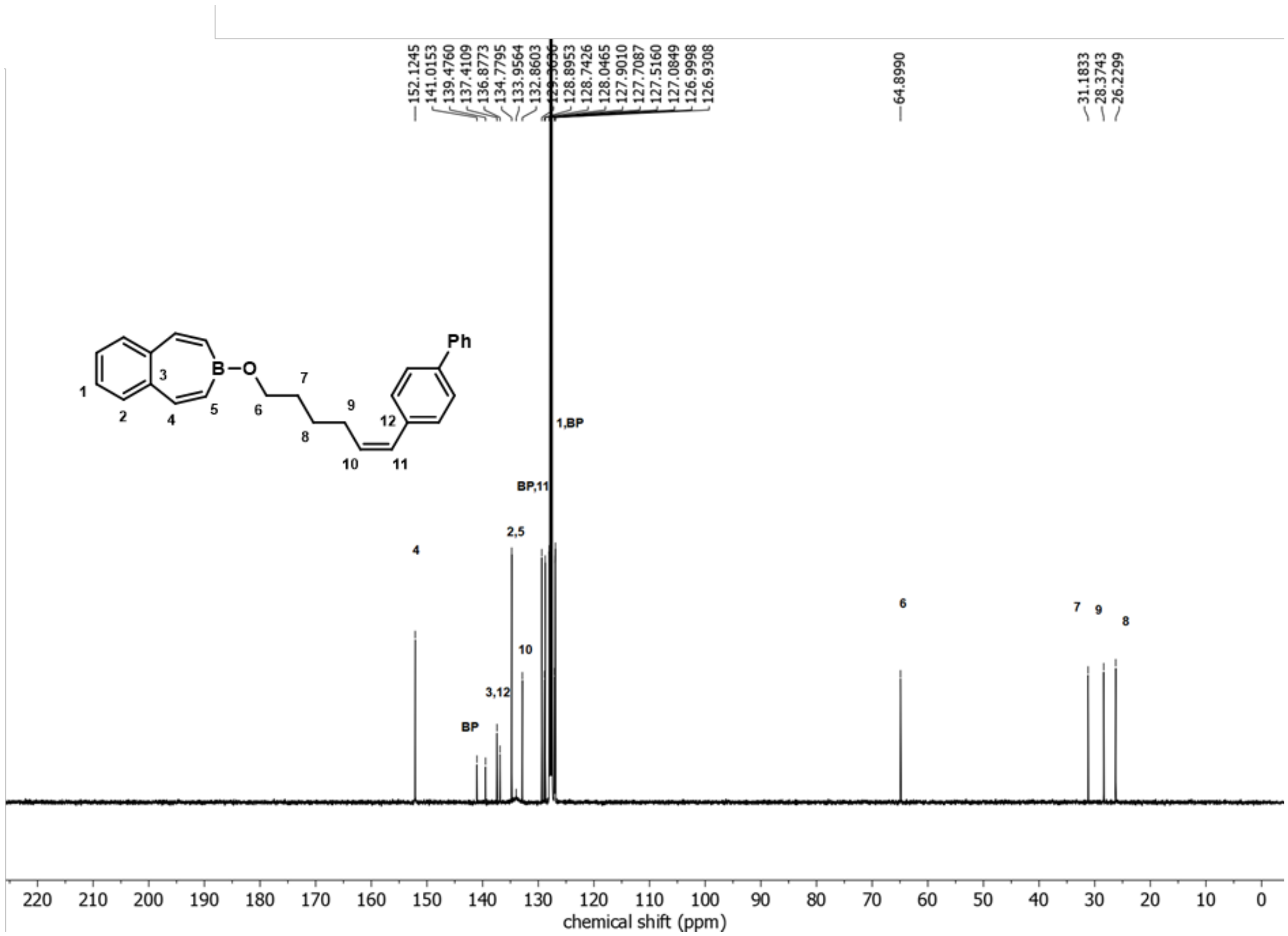

^{11}B NMR (160 MHz, Benzene-d_6) of Compound 6g

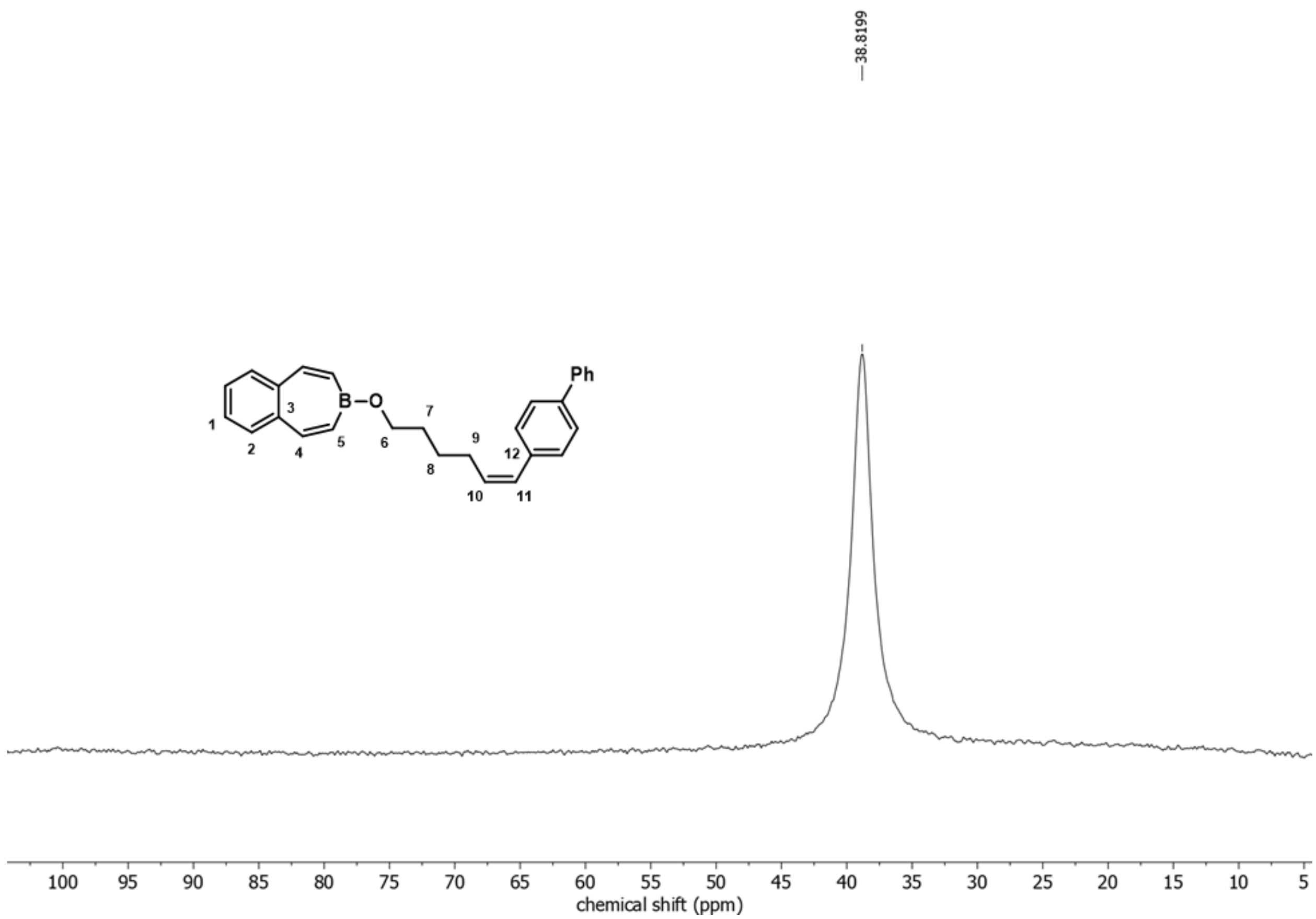

^{1}H -^{1}H COSY (400 MHz, Benzene-d_6) of Compound 6g

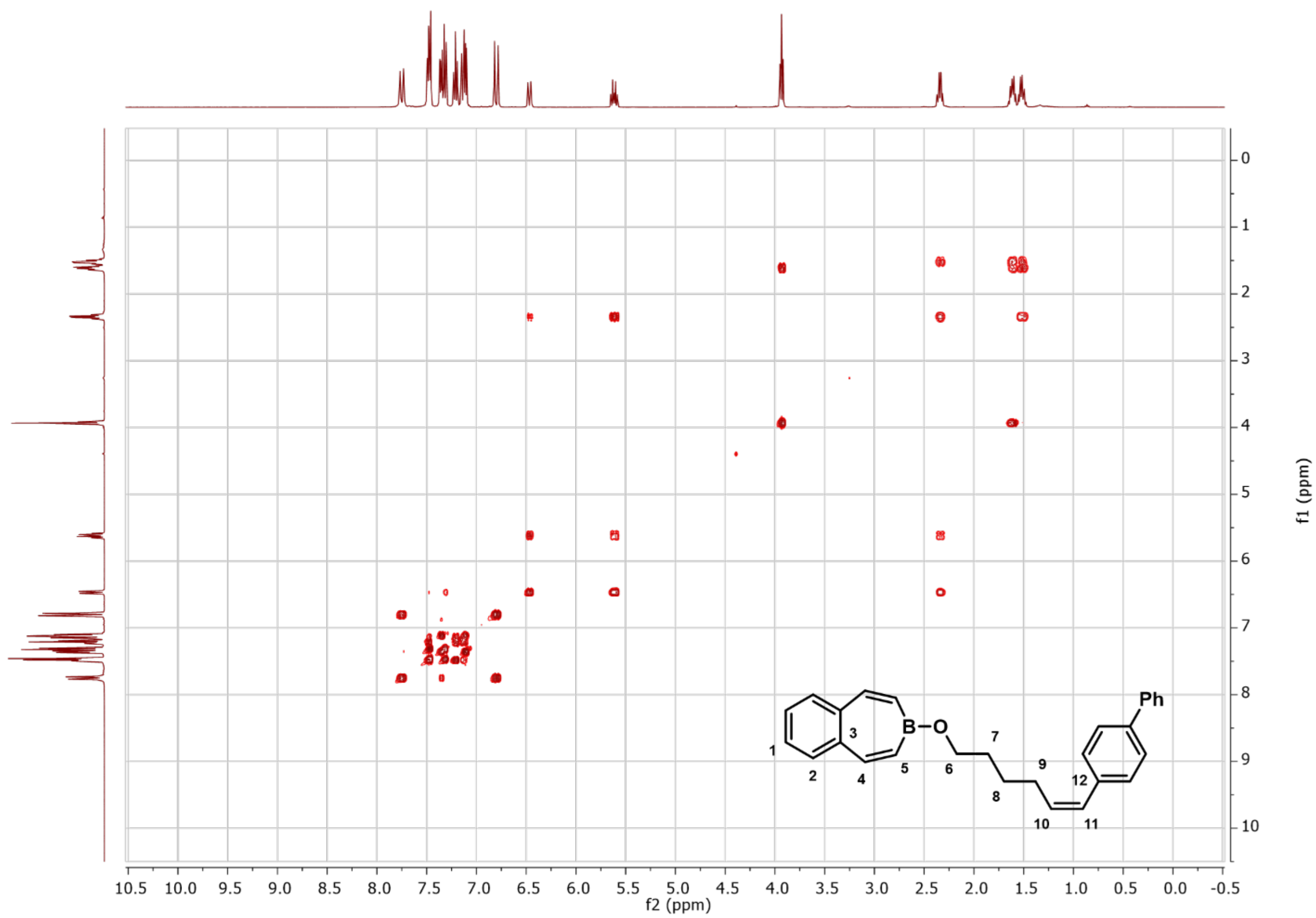

^{1}H -^{13}C HSQC (400 MHz, Benzene-d_6) of Compound 6g

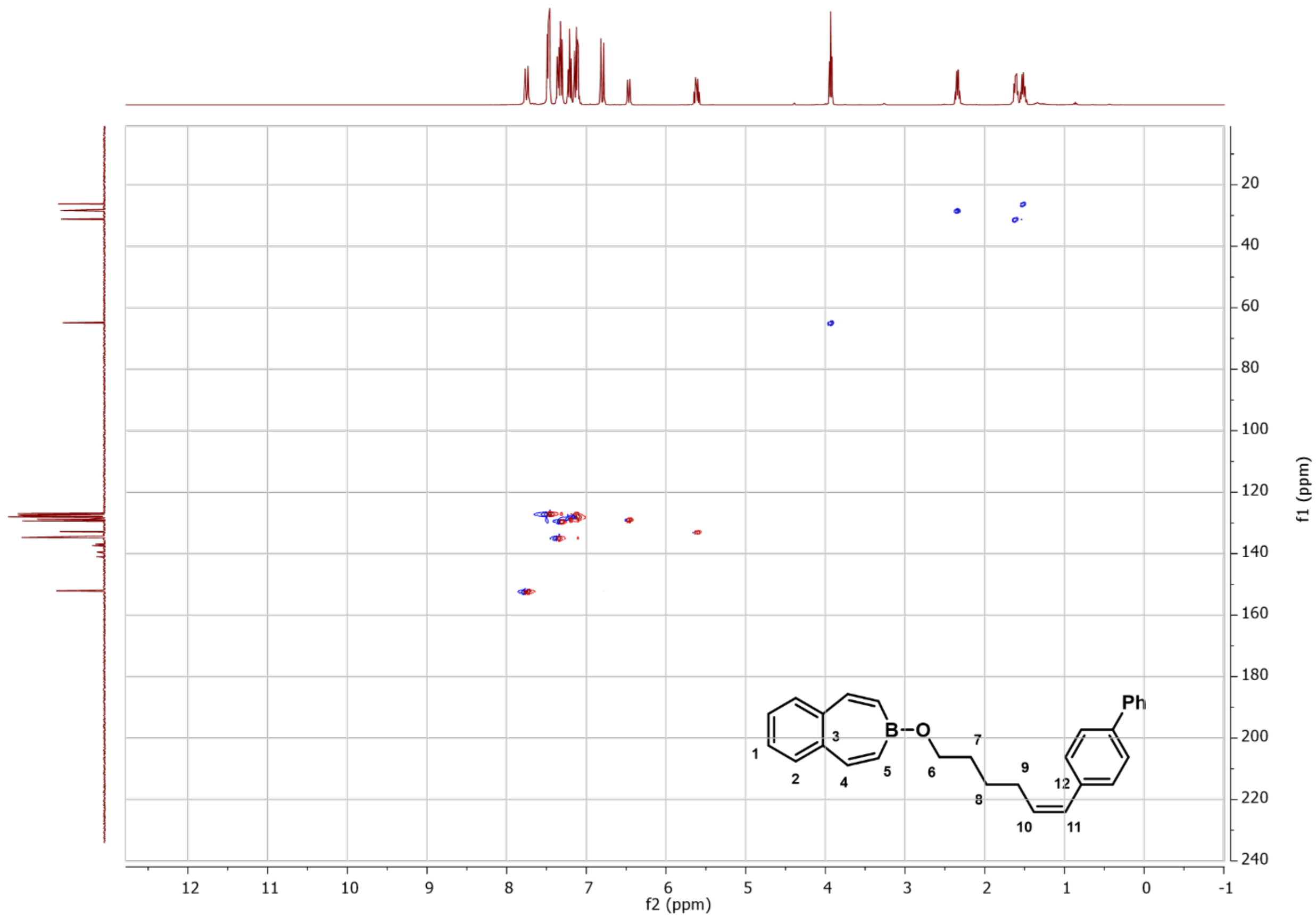

^{1}H -^{13}C HMBC (400 MHz, Benzene-d_6) of Compound 6g

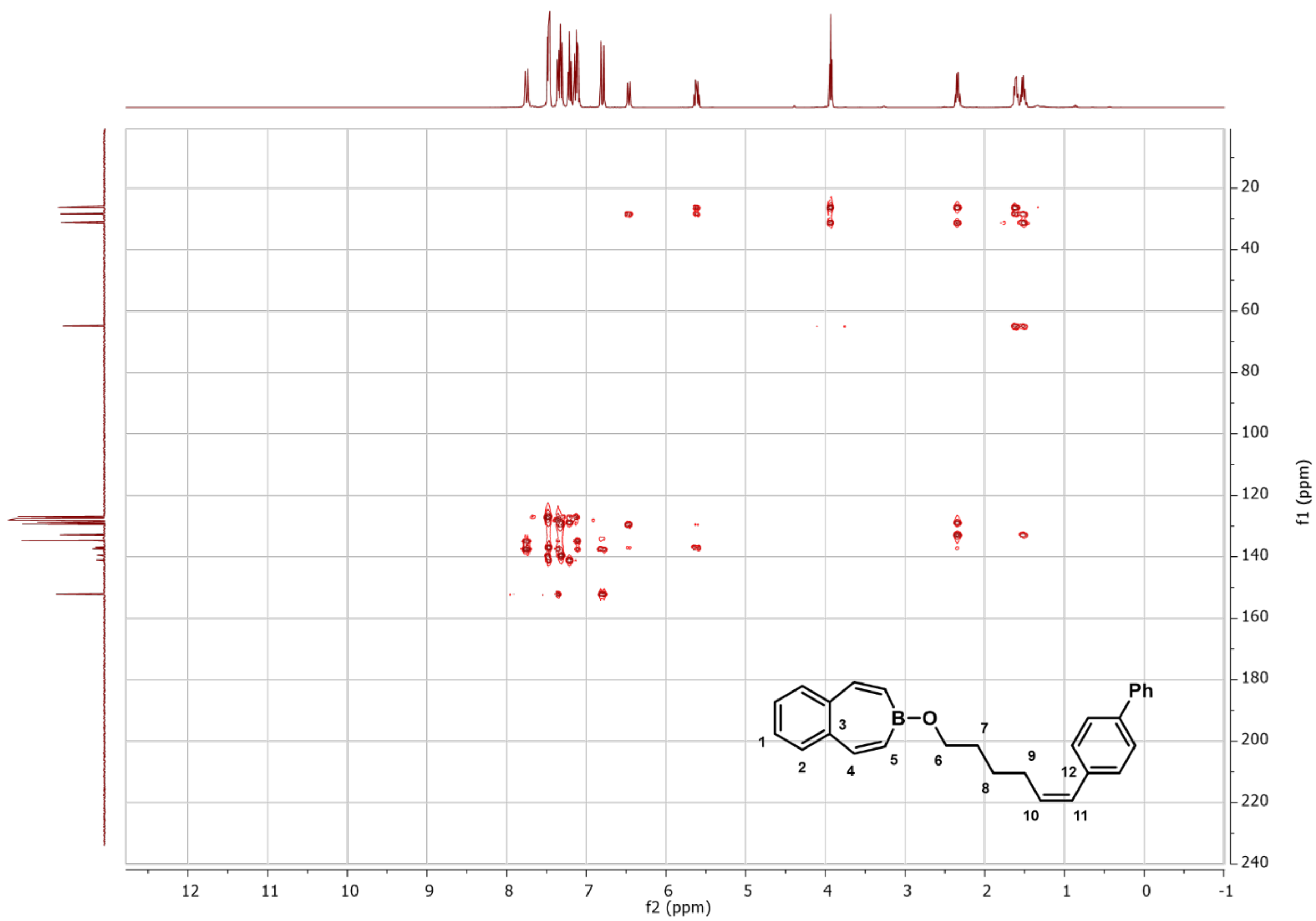

HRMS (ASAP) of Compound 6g

Elemental Composition Report **Page 1**

Single Mass Analysis
Tolerance = 50.0 PPM / DBE: min = -1.5, max = 500.0
Element prediction: Off
Number of isotope peaks used for i-FIT = 3

Monoisotopic Mass, Even Electron Ions
2 formula(e) evaluated with 1 results within limits (up to 50 closest results for each mass)
Elements Used:
C: 28-28 H: 27-50 B: 0-2 O: 1-1 Na: 0-1
DCM

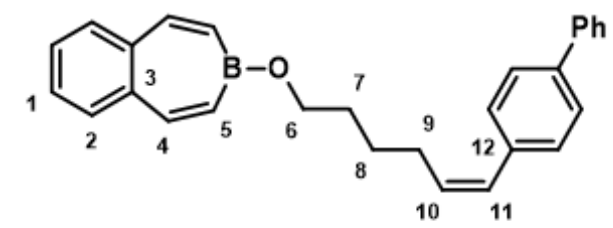

Chemical Formula: $C_{28}H_{27}BO$
Exact Mass: 390.2155

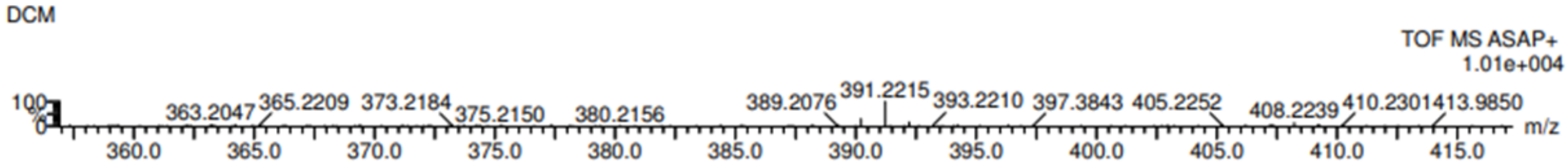

Minimum:				-1.5				
Maximum:		5.0	50.0	500.0				
Mass	Calc. Mass	mDa	PPM	DBE	i-FIT	Norm	Conf(%)	Formula
391.2215	391.2233	-1.8	-4.6	15.5	66.1	n/a	n/a	C28 H28 B O

^{1}H NMR (400 MHz, Benzene-d_6) of Compound 6h

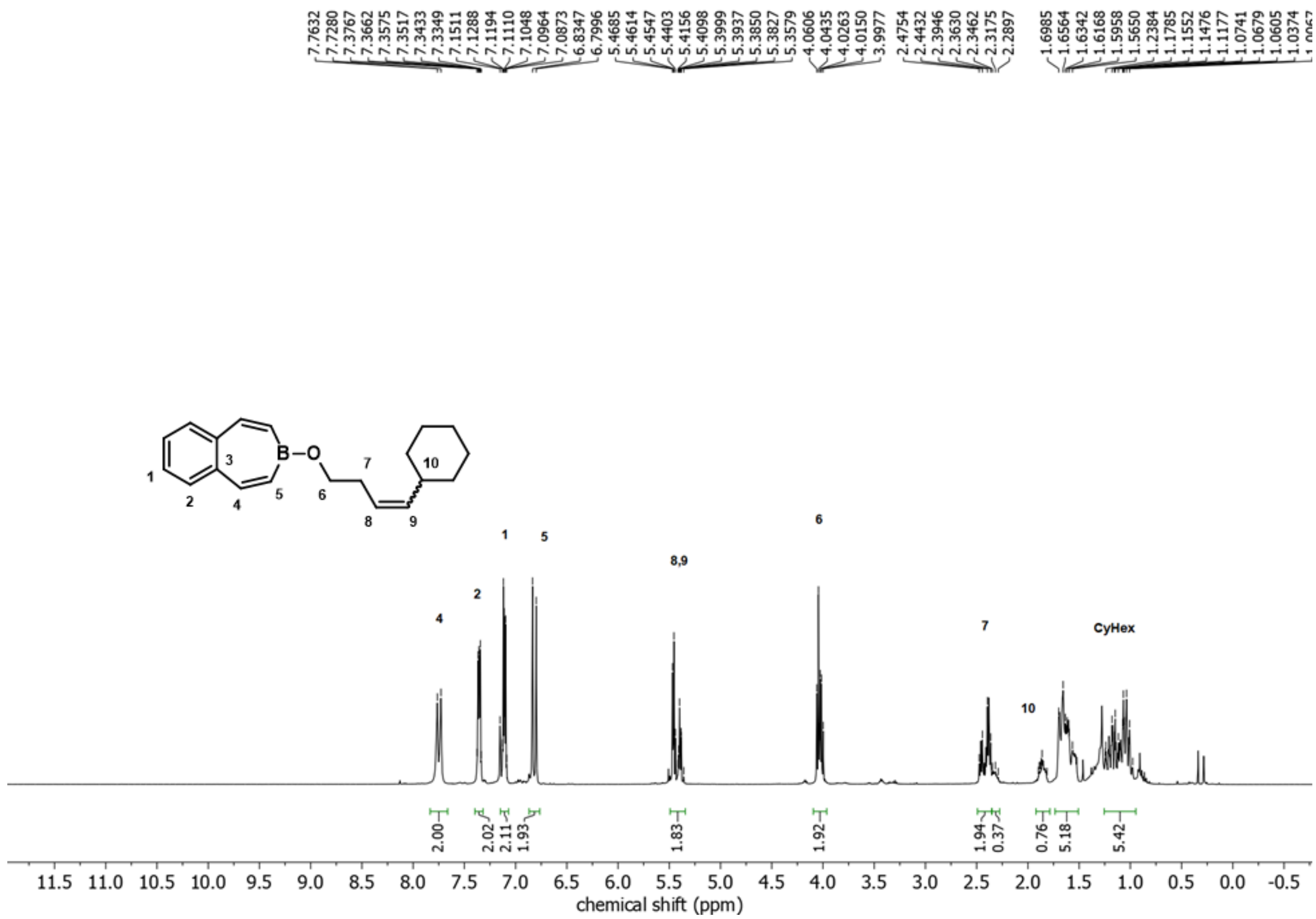

$^{13}C\{^{1}H\}$ NMR (101 MHz, Benzene-d_6) of Compound 6h

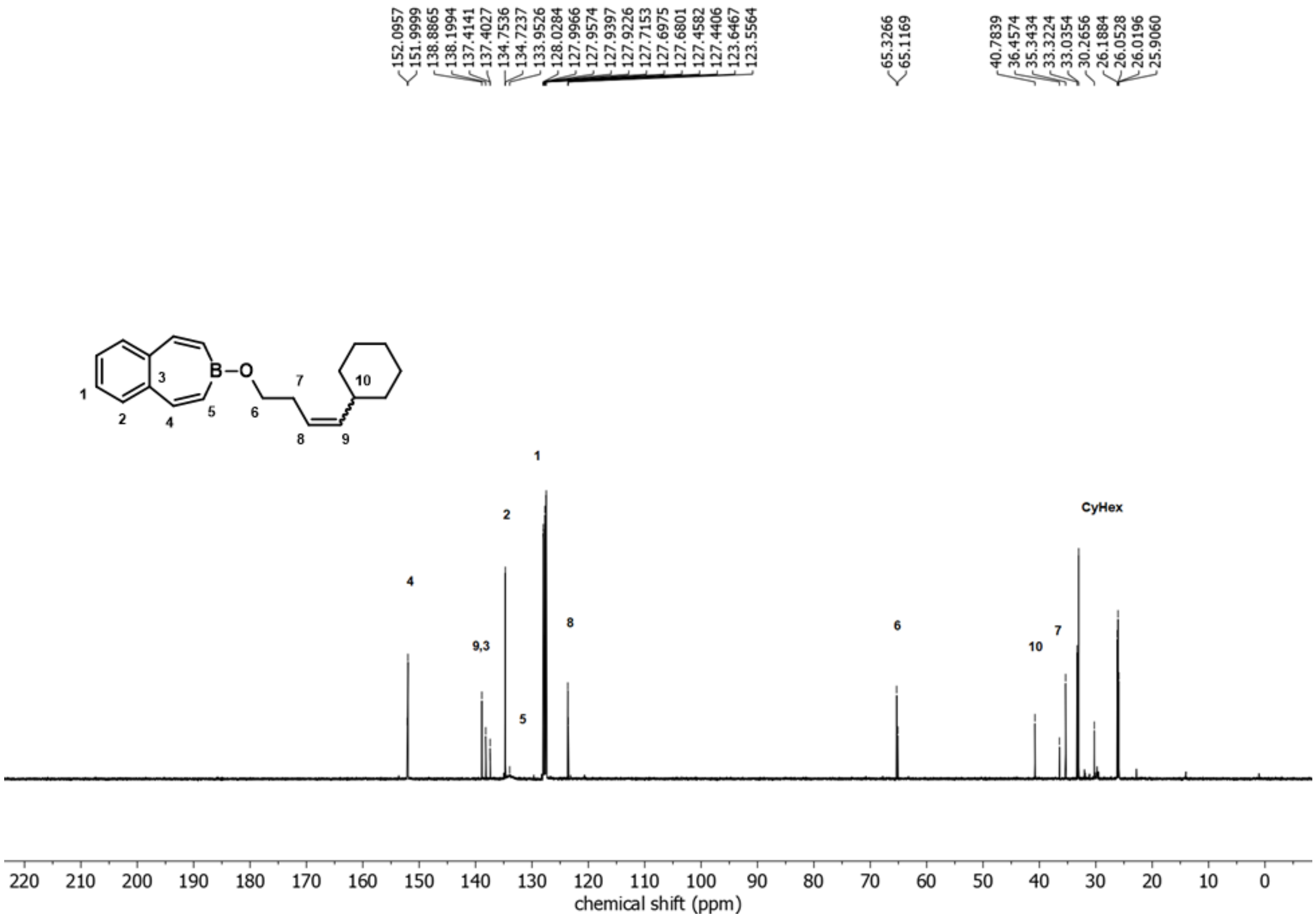

^{11}B NMR (128 MHz, Benzene-d_6) of Compound 6h

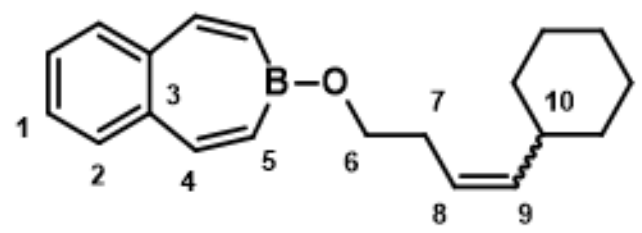

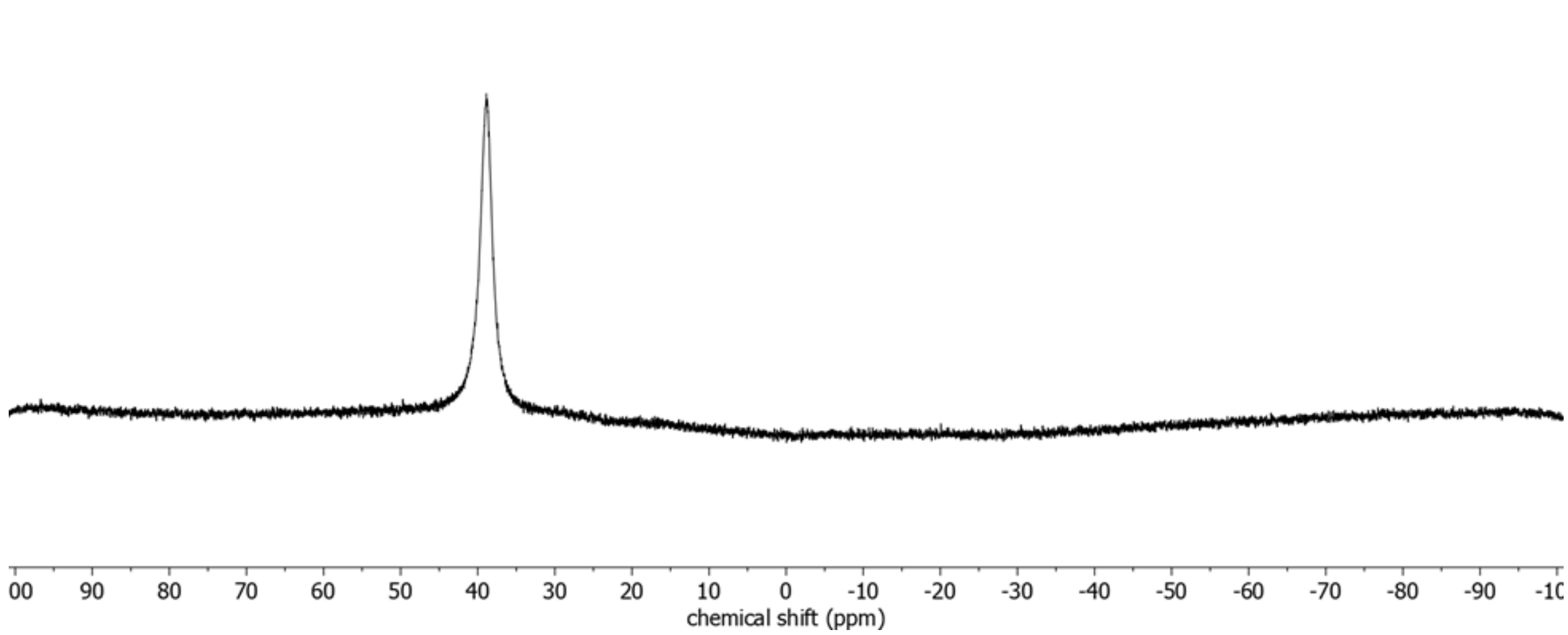

^{1}H -^{1}H COSY (400 MHz, Benzene-d_6) of Compound 6h

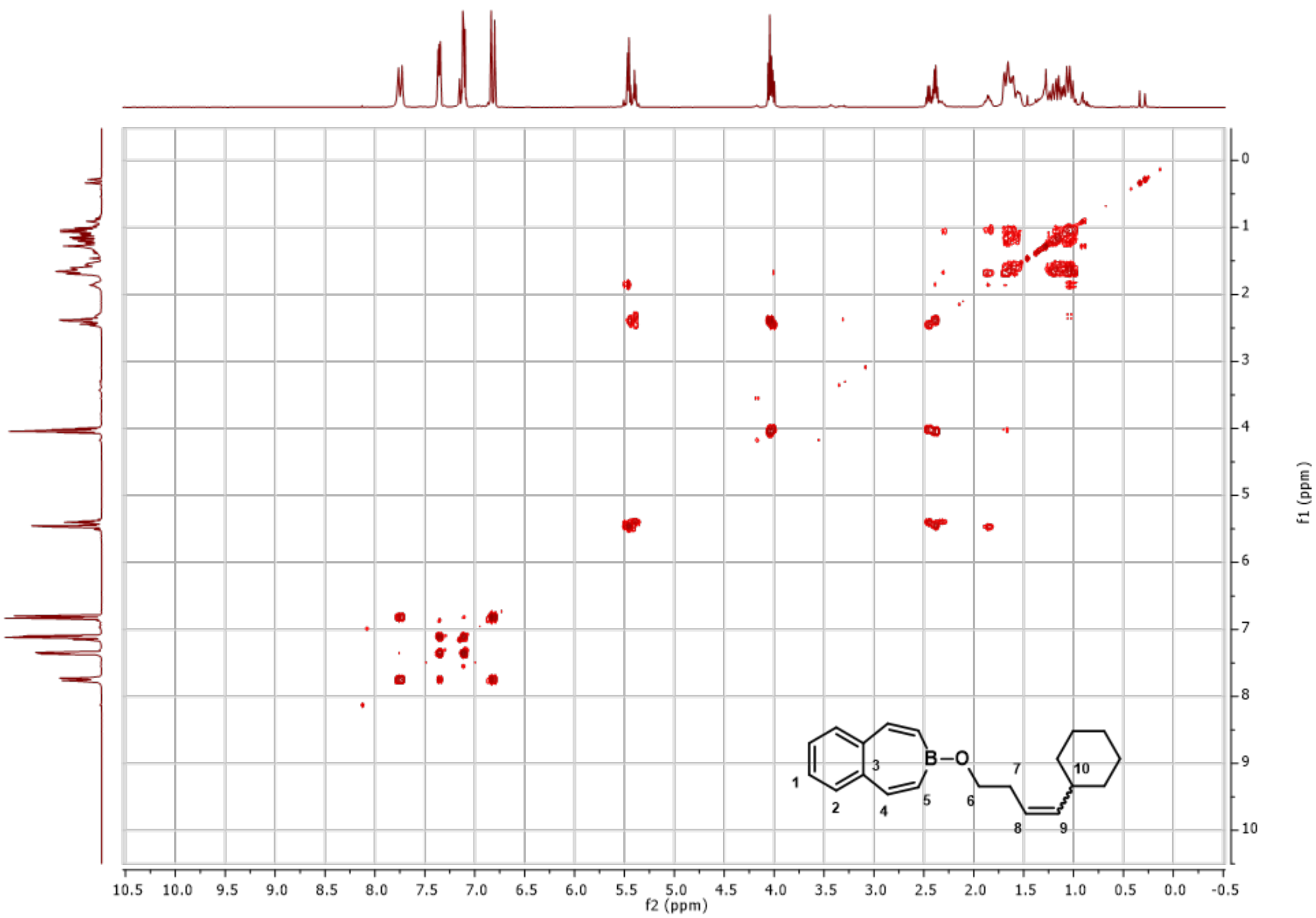

^{1}H -^{13}C HSQC (400 MHz, Benzene-d_6) of Compound 6h

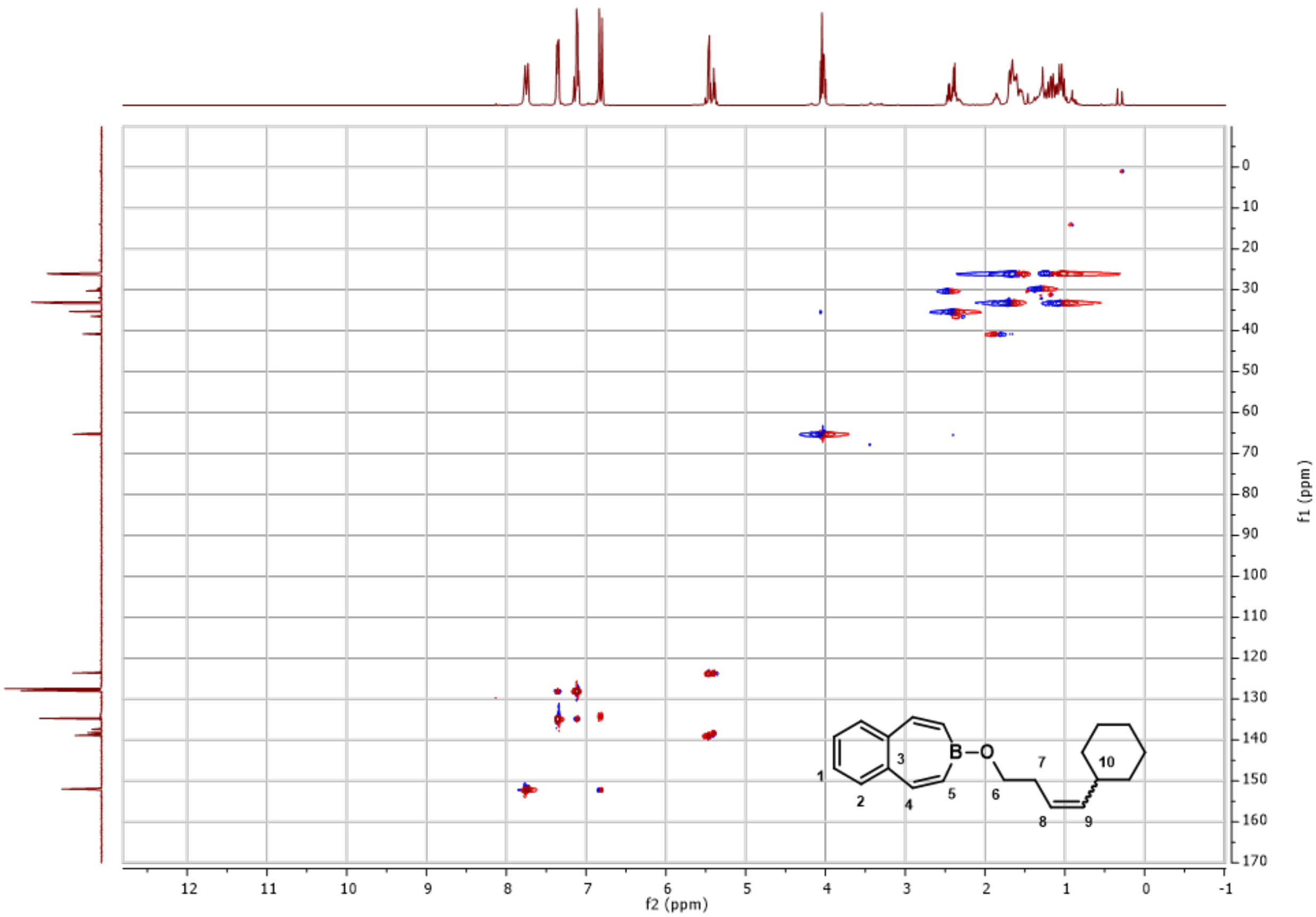

^{1}H -^{13}C HMBC (400 MHz, Benzene-d_6) of Compound 6h

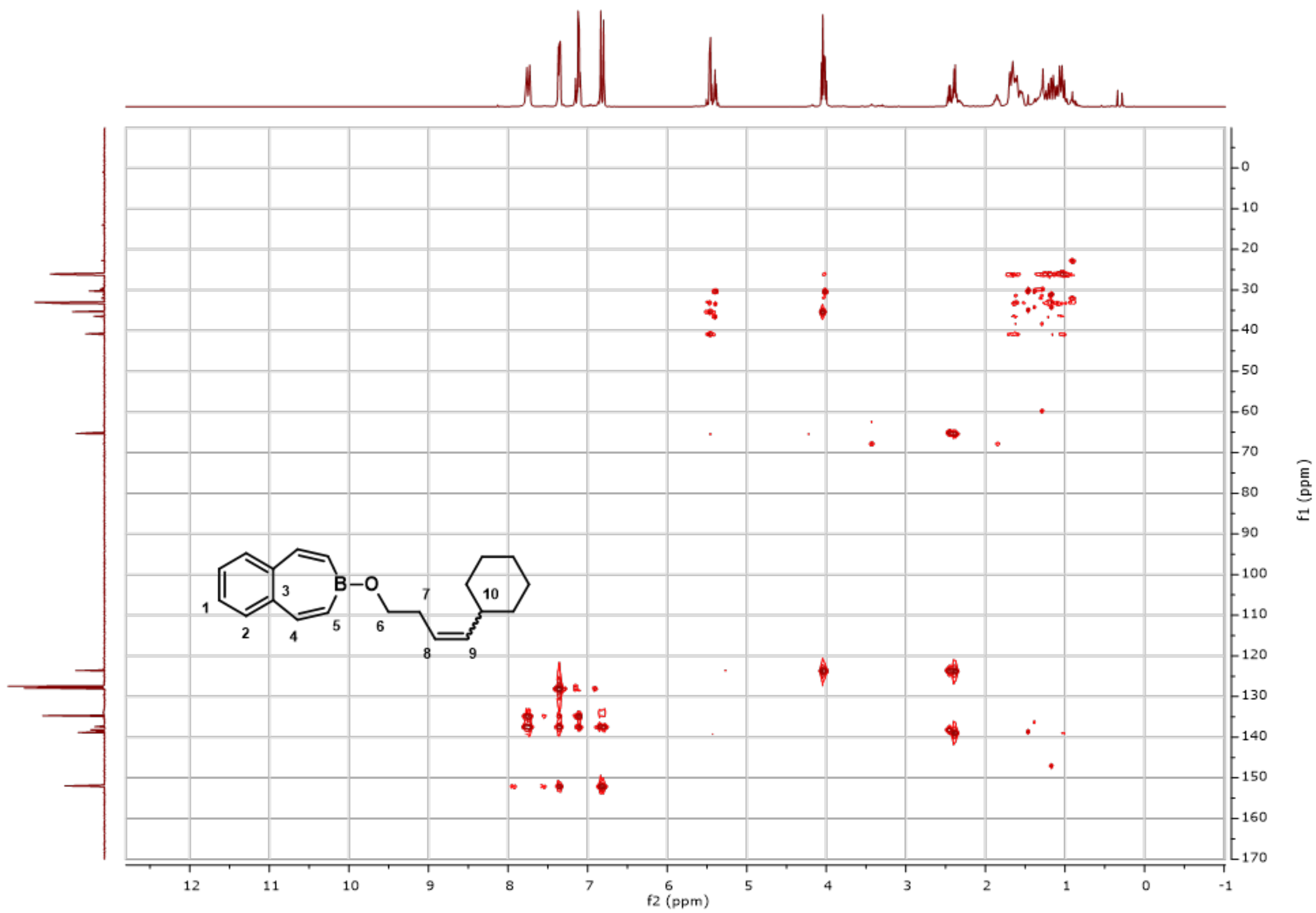

HRMS (ASAP) of Compound 6h

Elemental Composition Report | **Page 1**

Single Mass Analysis
Tolerance = 5.0 PPM / DBE: min = -1.5, max = 500.0
Element prediction: Off
Number of isotope peaks used for i-FIT = 3

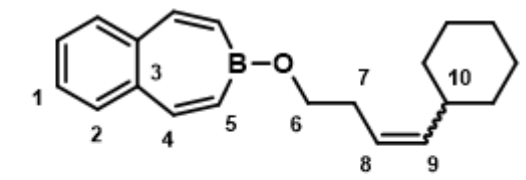

Chemical Formula: $C_{20}H_{25}BO$
Exact Mass: 292.1998

Monoisotopic Mass, Even Electron Ions
3 formula(e) evaluated with 1 results within limits (up to 50 closest results for each mass)
Elements Used:
C: 20-20 H: 20-45 B: 0-2 O: 1-1 Na: 0-1
DCM

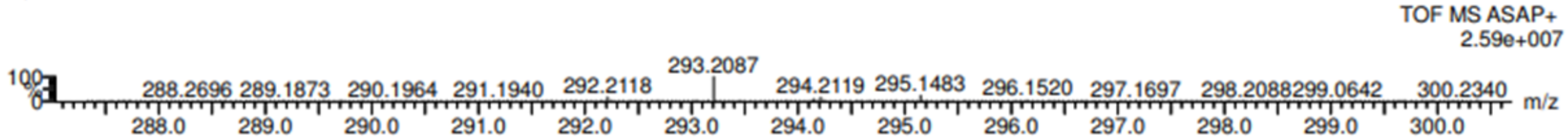

Mass	Calc. Mass	mDa	PPM	DBE	i-FIT	Norm	Conf(%)	Formula
Minimum:				-1.5				
Maximum:		5.0	5.0	500.0				
293.2087	293.2077	1.0	3.4	8.5	1037.9	n/a	n/a	C20 H26 B O

^{1}H NMR (500 MHz, Benzene-d_6) of Compound 6i

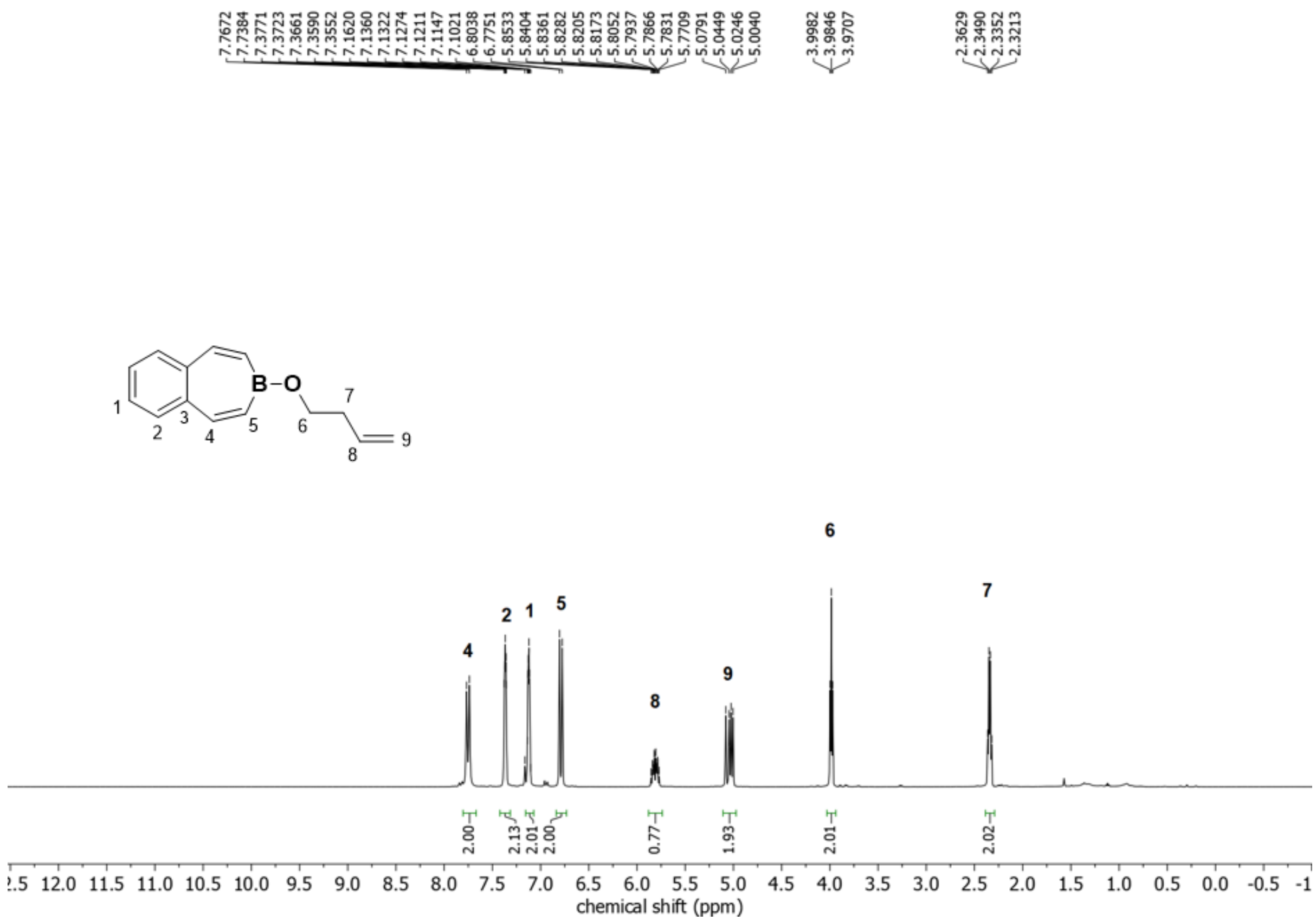

$^{13}C\{^1H\}$ NMR (126 MHz, Benzene-d_6) of Compound 6i

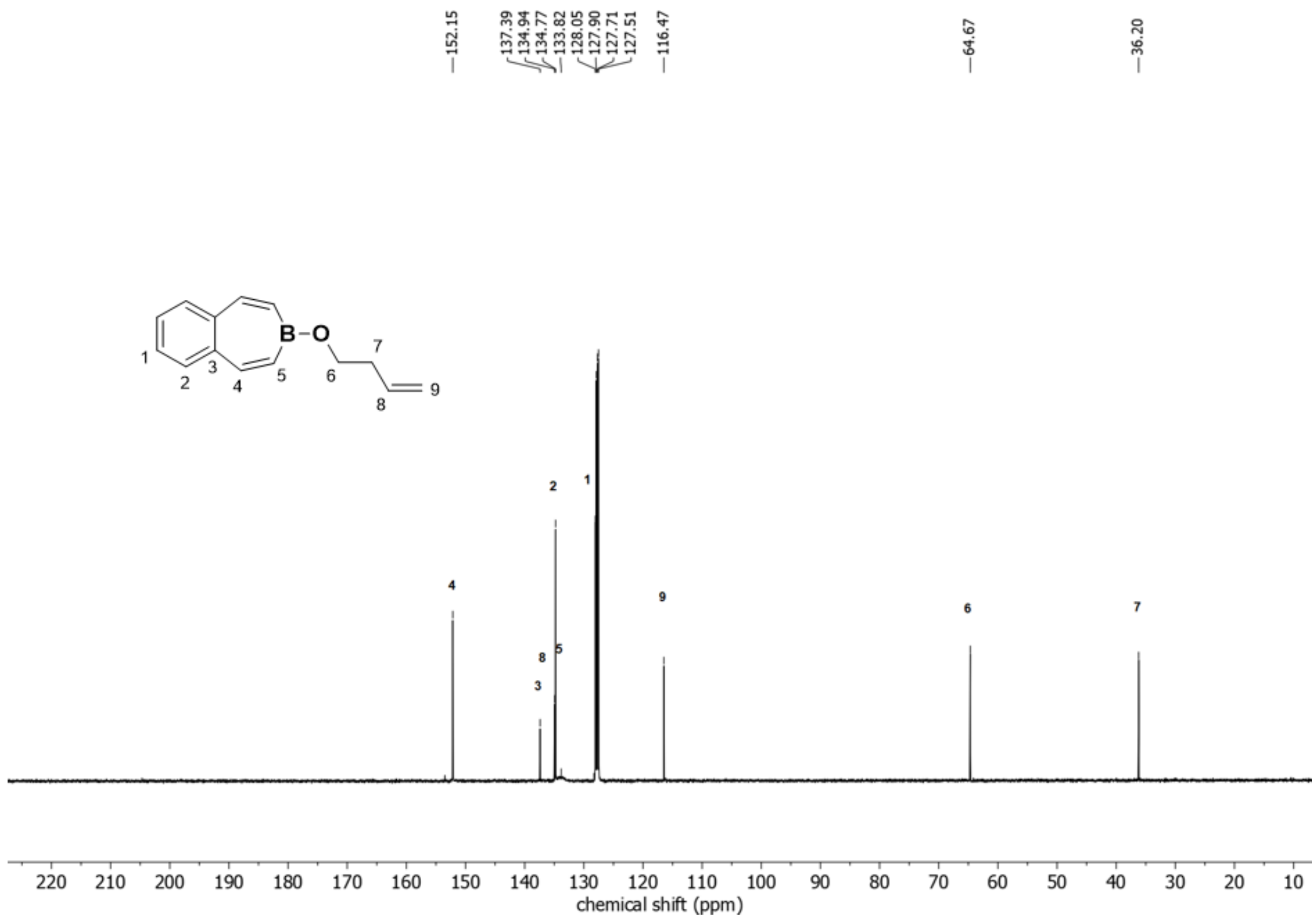

^{11}B NMR (160 MHz, Benzene-d_6) of Compound 6i

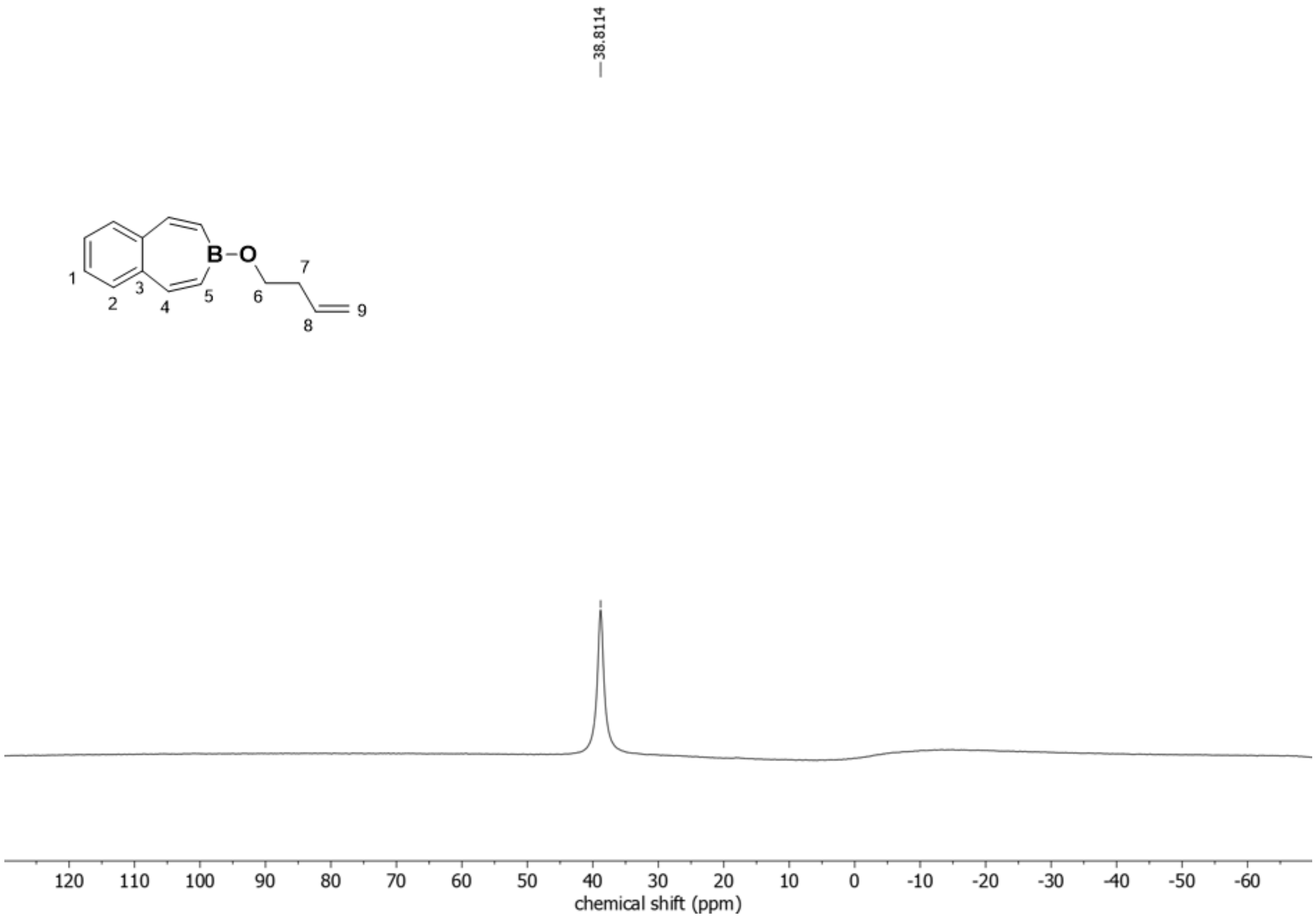

HRMS (ASAP) of Compound 6i

Elemental Composition Report — **Page 1**

Single Mass Analysis
Tolerance = 5.0 PPM / DBE: min = -1.5, max = 500.0
Element prediction: Off
Number of isotope peaks used for i-FIT = 3

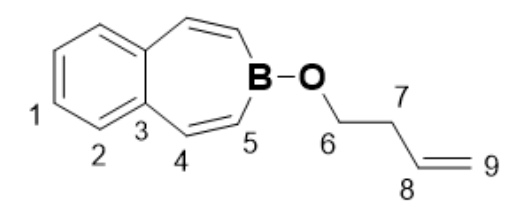

Chemical Formula: $C_{14}H_{15}BO$
Exact Mass: 210.1216

Monoisotopic Mass, Even Electron Ions
49 formula(e) evaluated with 1 results within limits (up to 50 closest results for each mass)
Elements Used:
C: 14-21 H: 10-25 B: 0-1 N: 0-6 O: 0-3

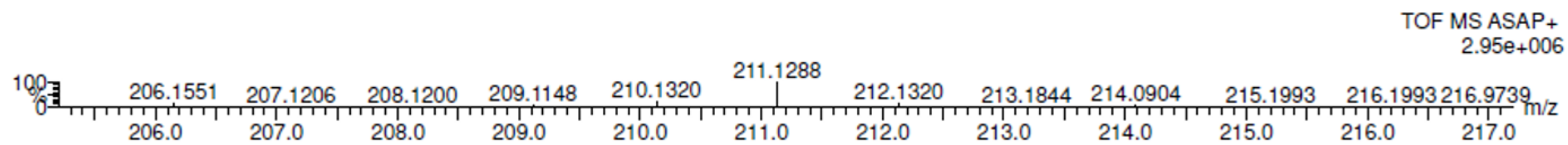

Minimum:				-1.5				
Maximum:		5.0	5.0	500.0				
Mass	Calc. Mass	mDa	PPM	DBE	i-FIT	Norm	Conf(%)	Formula
211.1288	211.1294	-0.6	-2.8	7.5	823.6	n/a	n/a	C14 H16 B O

^{1}H NMR (500 MHz, Benzene-d_6) of Compound 7a

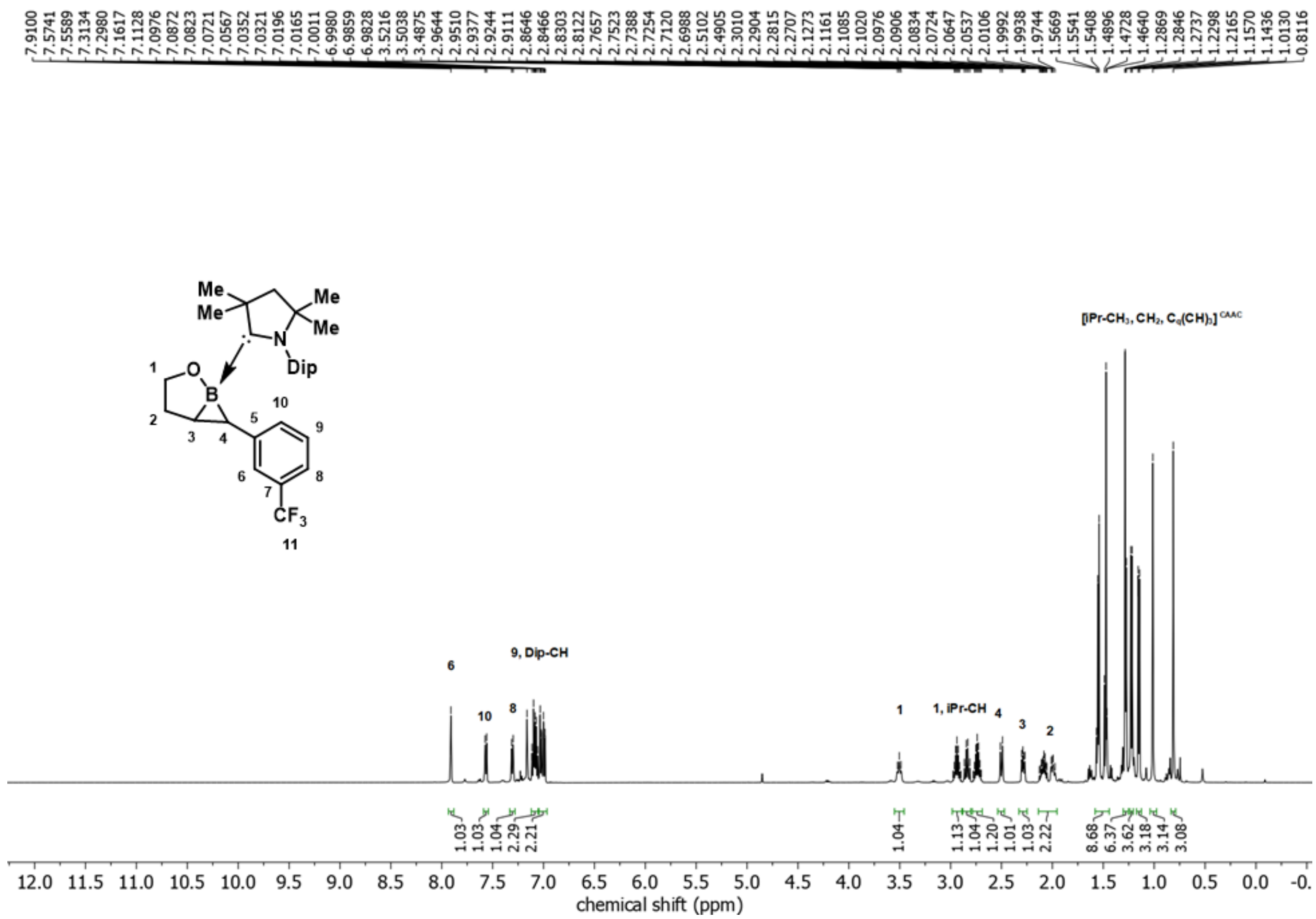

$^{13}C\{^1H\}$ NMR (126 MHz, Benzene-d_6) of Compound 7a

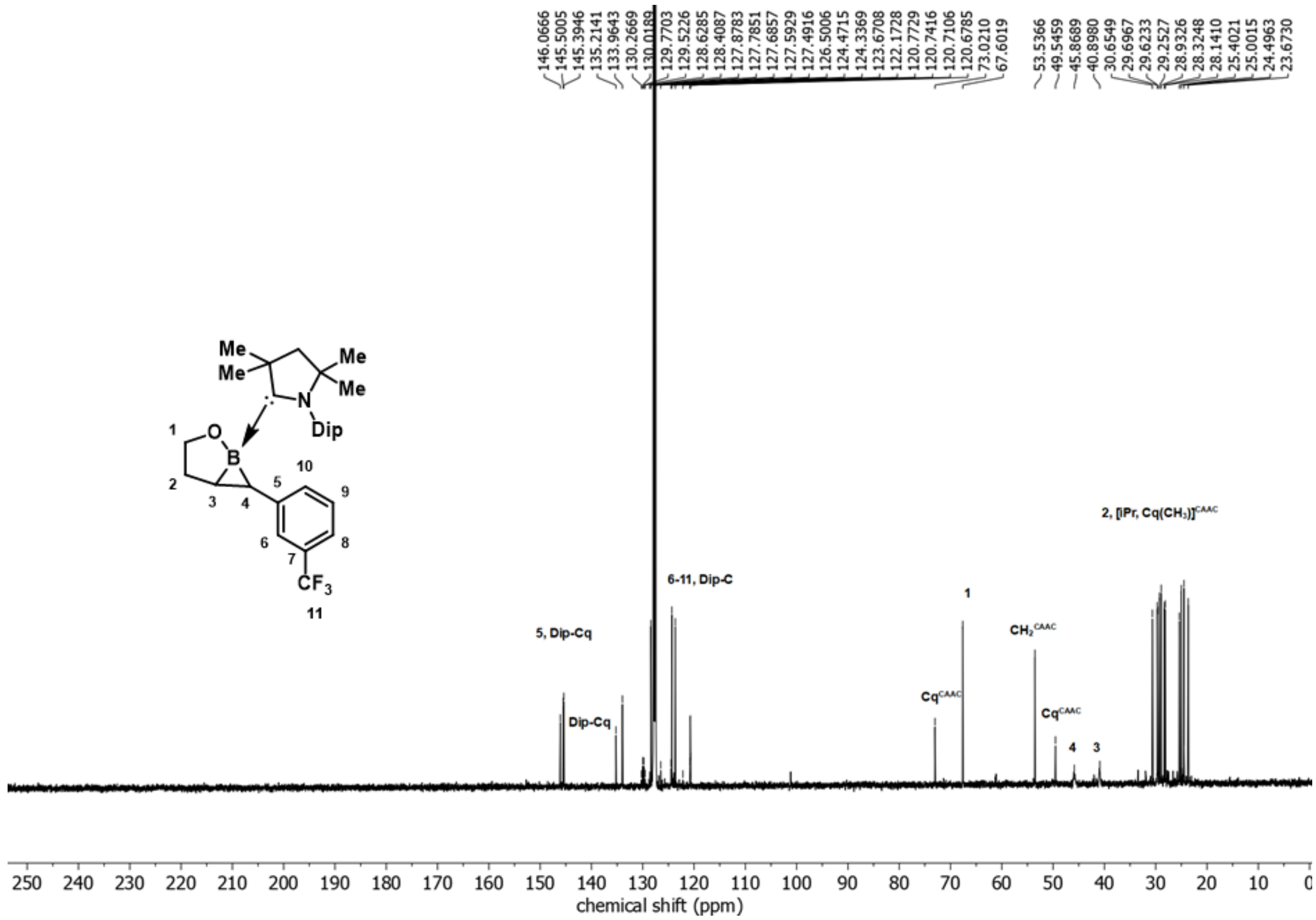

^{19}F NMR (471 MHz, Benzene-*d*$_6$) of Compound 7a

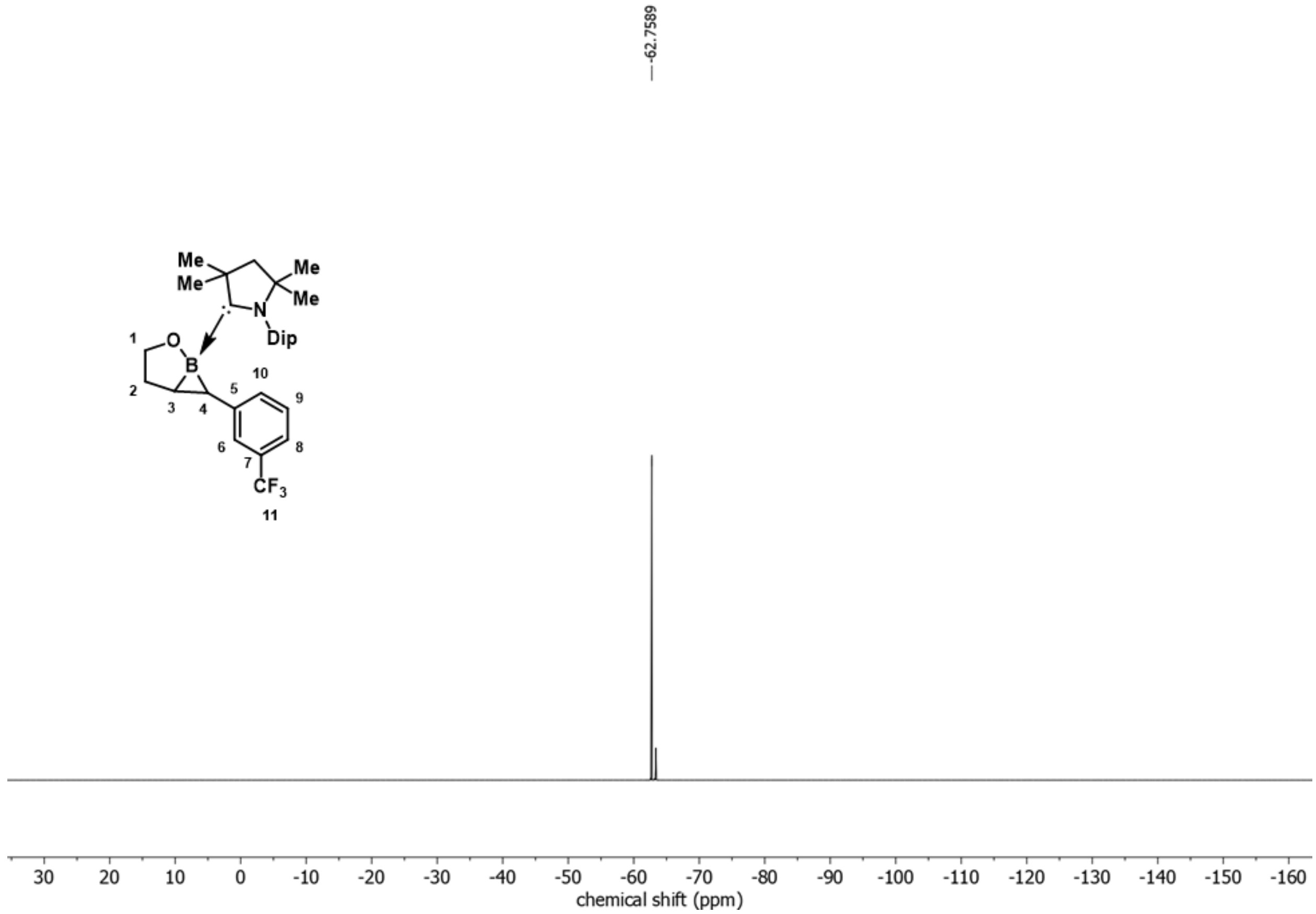

^{11}B NMR (160 MHz, Benzene-d_6) of Compound 7a

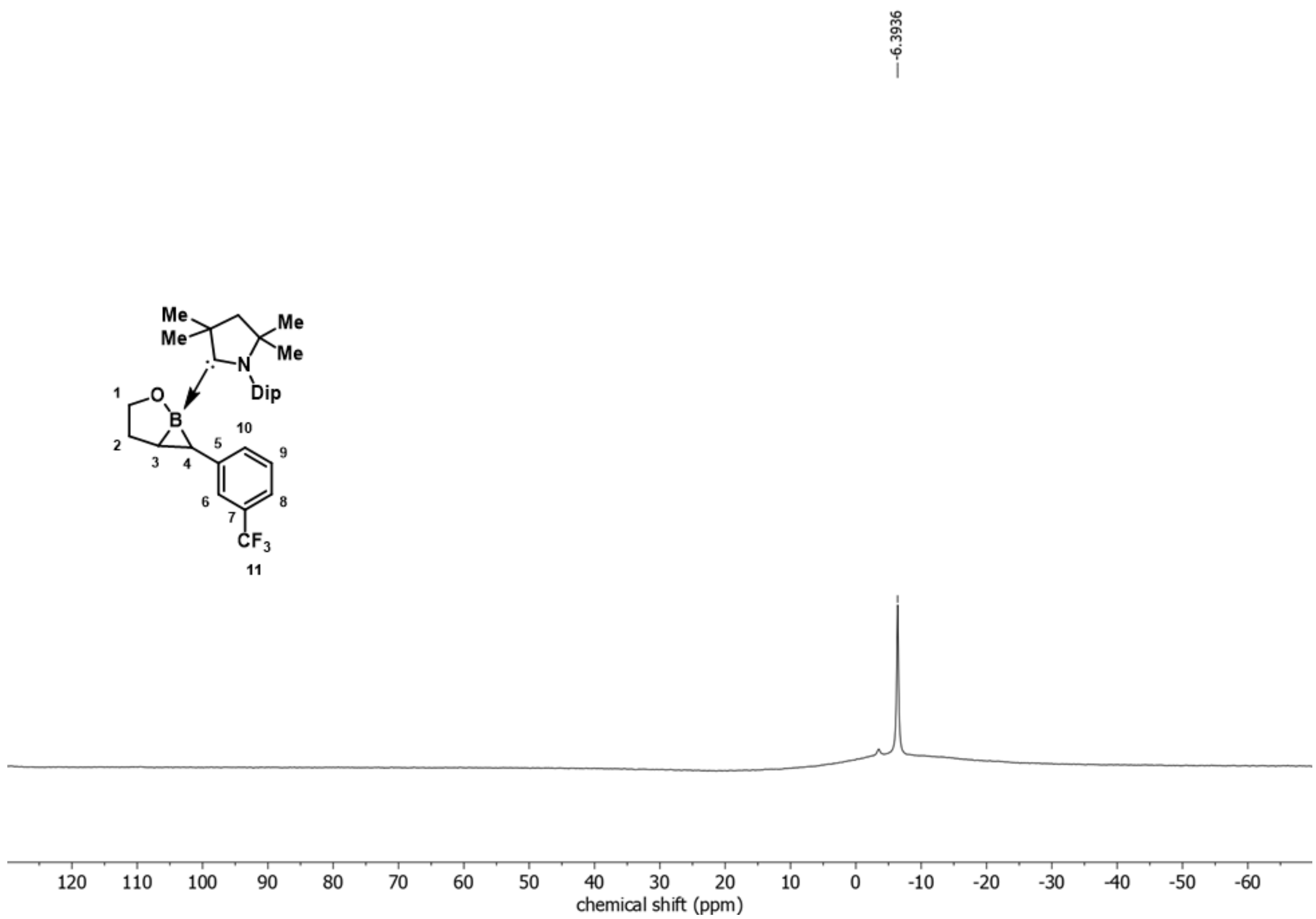

^{1}H -^{1}H COSY (500 MHz, Benzene-d_6) of Compound 7a

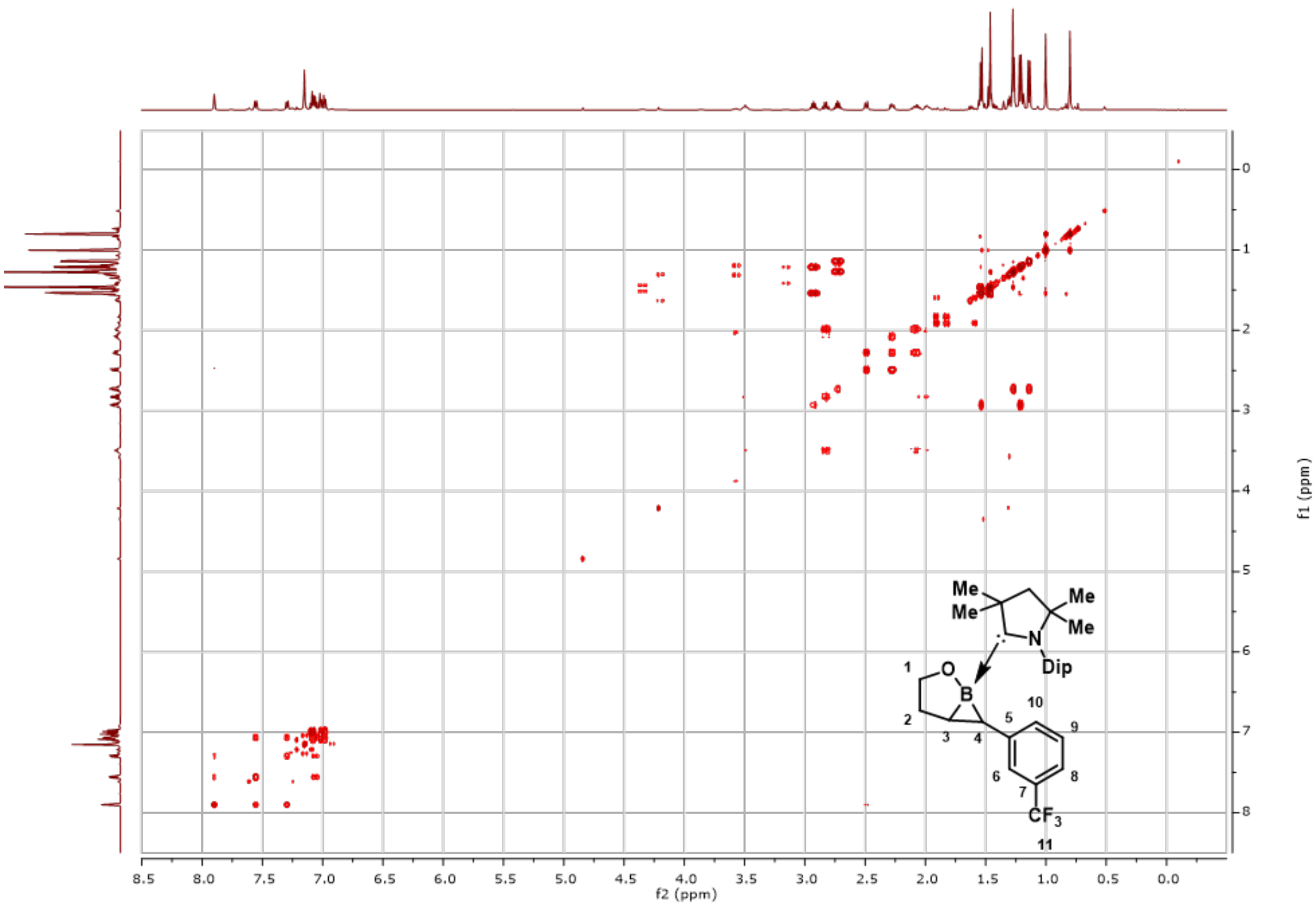

^{1}H -^{13}C HSQC (500 MHz, Benzene-d_6) of Compound 7a

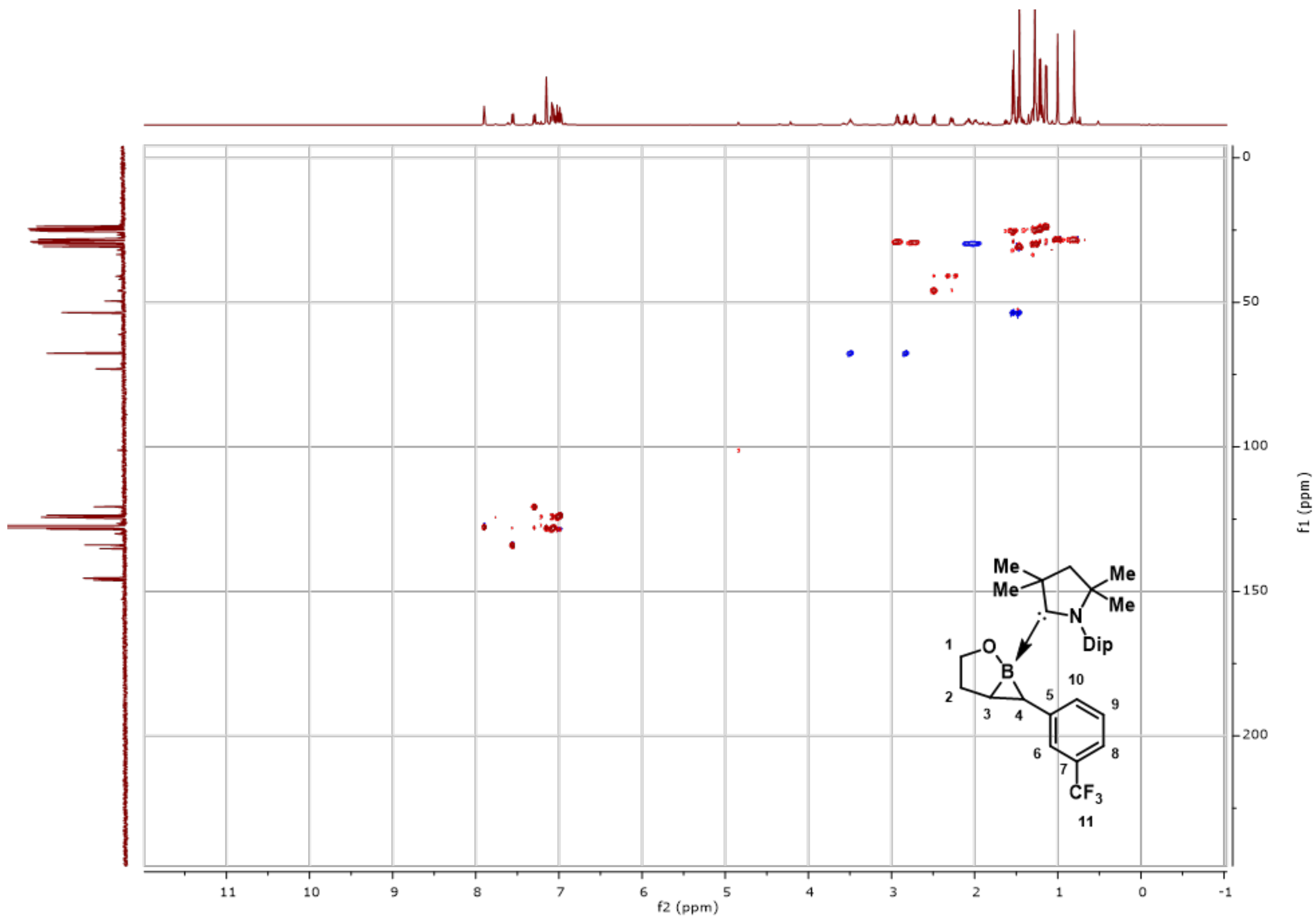

^{1}H -^{13}C HMBC (500 MHz, Benzene-d_6) of Compound 7a

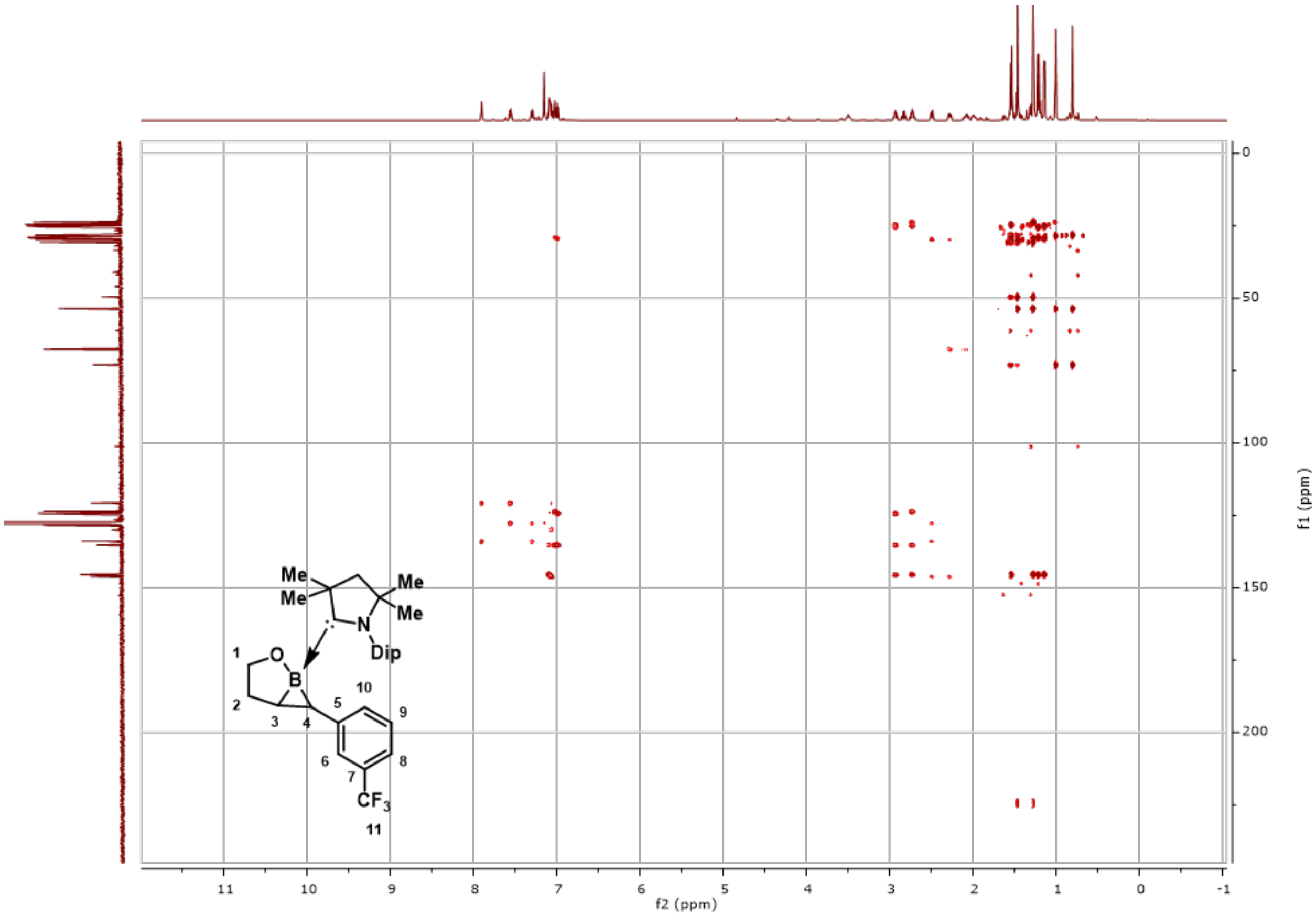

HRMS (ESI) of Compound 7a

Elemental Composition Report — Page 1

Single Mass Analysis
Tolerance = 5.0 PPM / DBE: min = -1.5, max = 500.0
Element prediction: Off
Number of isotope peaks used for i-FIT = 3

Monoisotopic Mass, Even Electron Ions
293 formula(e) evaluated with 1 results within limits (up to 50 closest results for each mass)
Elements Used:
C: 31-31 H: 10-50 B: 0-1 N: 0-6 O: 0-3 F: 0-6

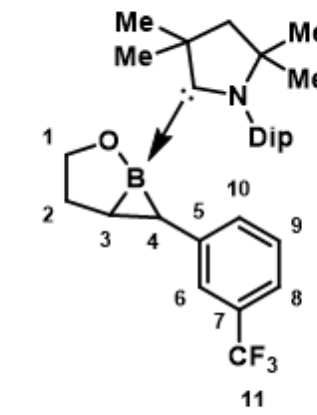

Chemical Formula: $C_{31}H_{41}BF_3NO$
Exact Mass: 511.3233

DCM

1: TOF MS ES+
8.18e+004

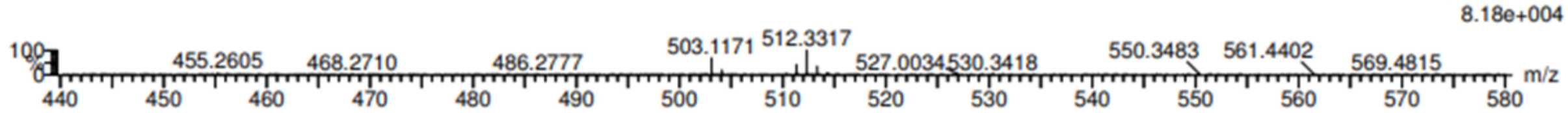

Mass	Calc. Mass	mDa	PPM	DBE	i-FIT	Norm	Conf(%)	Formula
Minimum:				-1.5				
Maximum:		5.0	5.0	500.0				
512.3317	512.3312	0.5	1.0	10.5	320.9	n/a	n/a	C31 H42 B N O F3

^{1}H NMR (500 MHz, Benzene-d_6) of Compound 7c

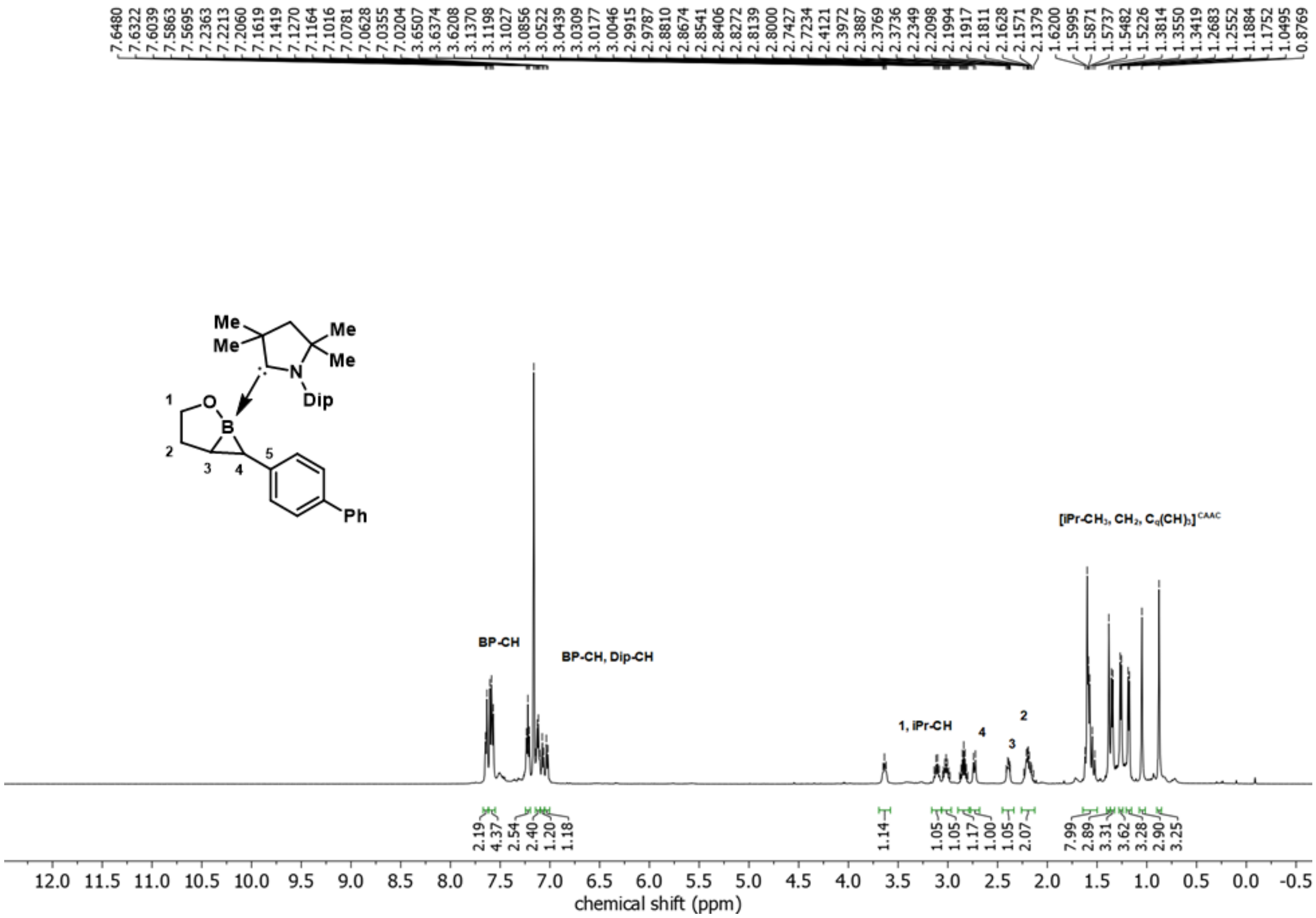

$^{13}C\{^{1}H\}$ NMR (126 MHz, Benzene-d_6) of Compound 7c

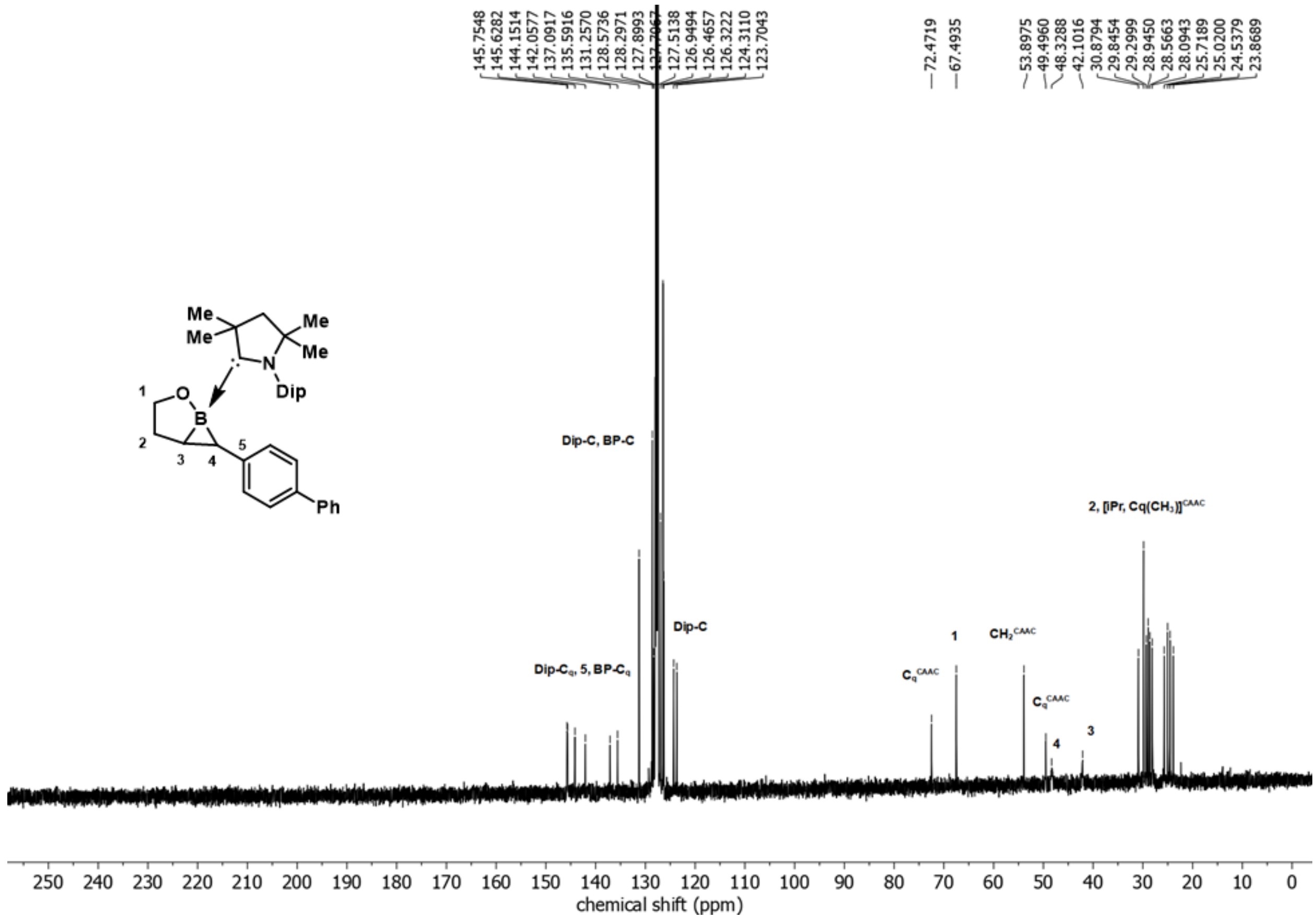

^{11}**B NMR (160 MHz, Benzene-*d*$_6$) of Compound 7c**

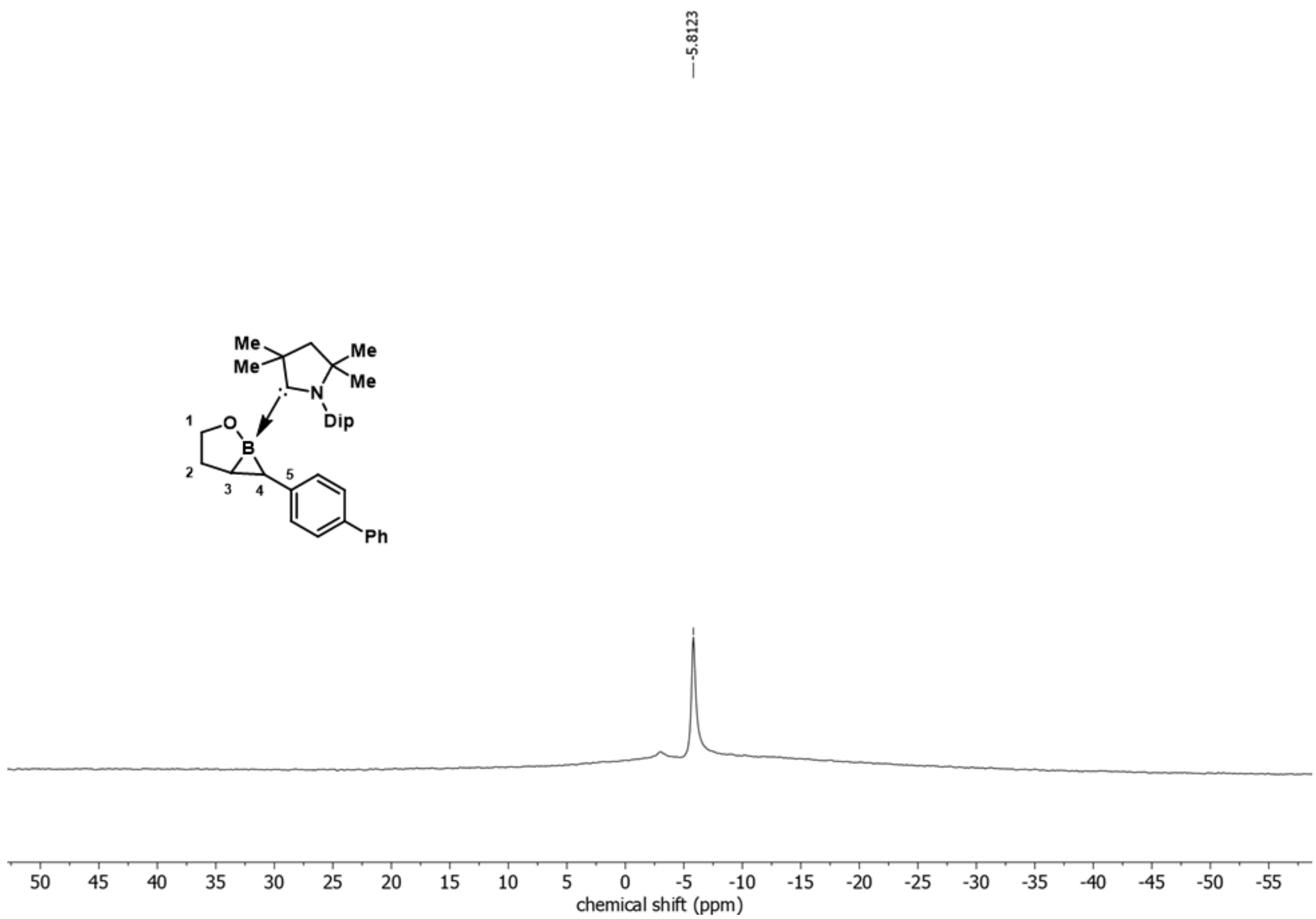

^{1}H -^{1}H COSY (500 MHz, Benzene-d_6) of Compound 7c

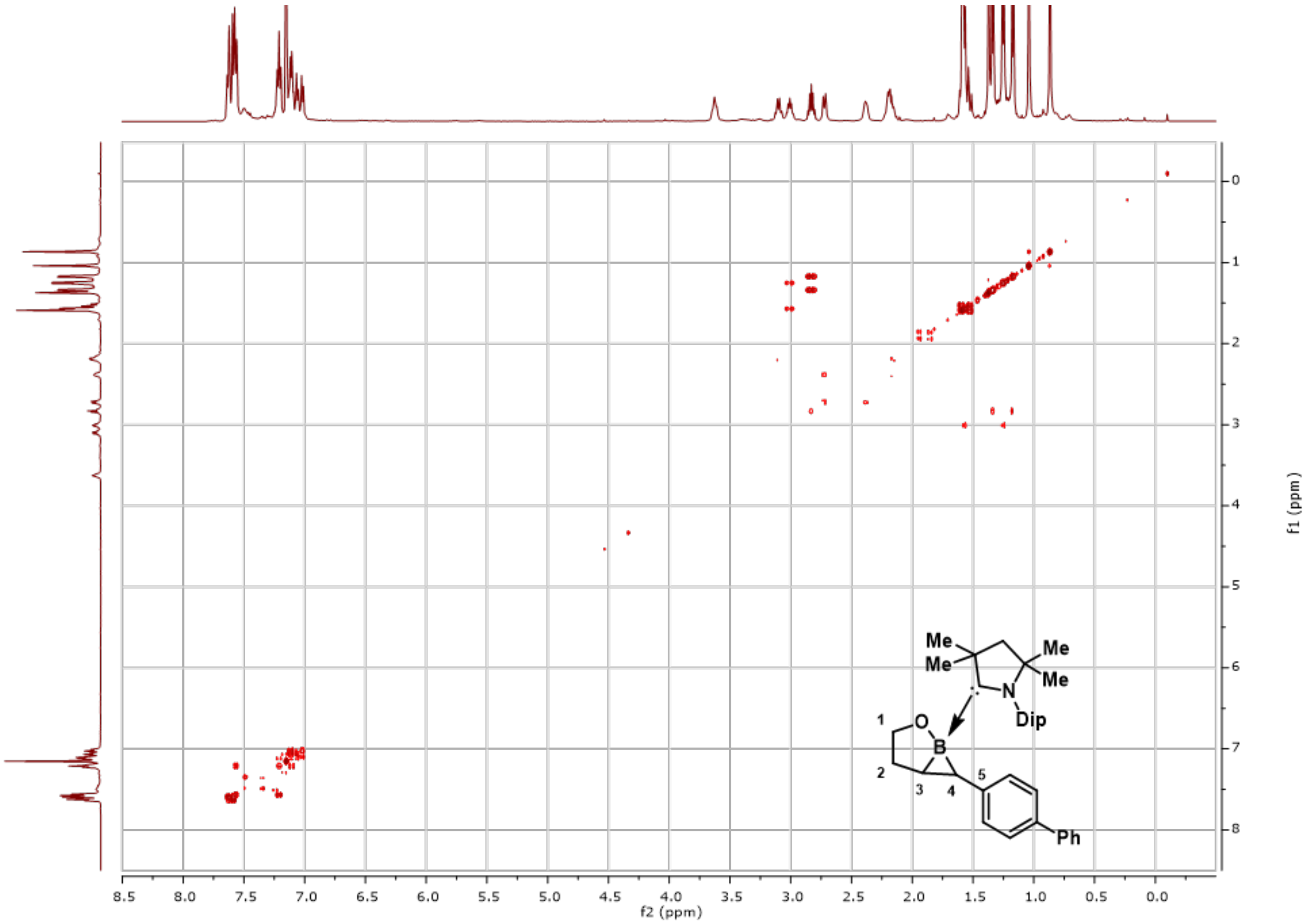

^{1}H -^{13}C HSQC (500 MHz, Benzene-d_6) of Compound 7c

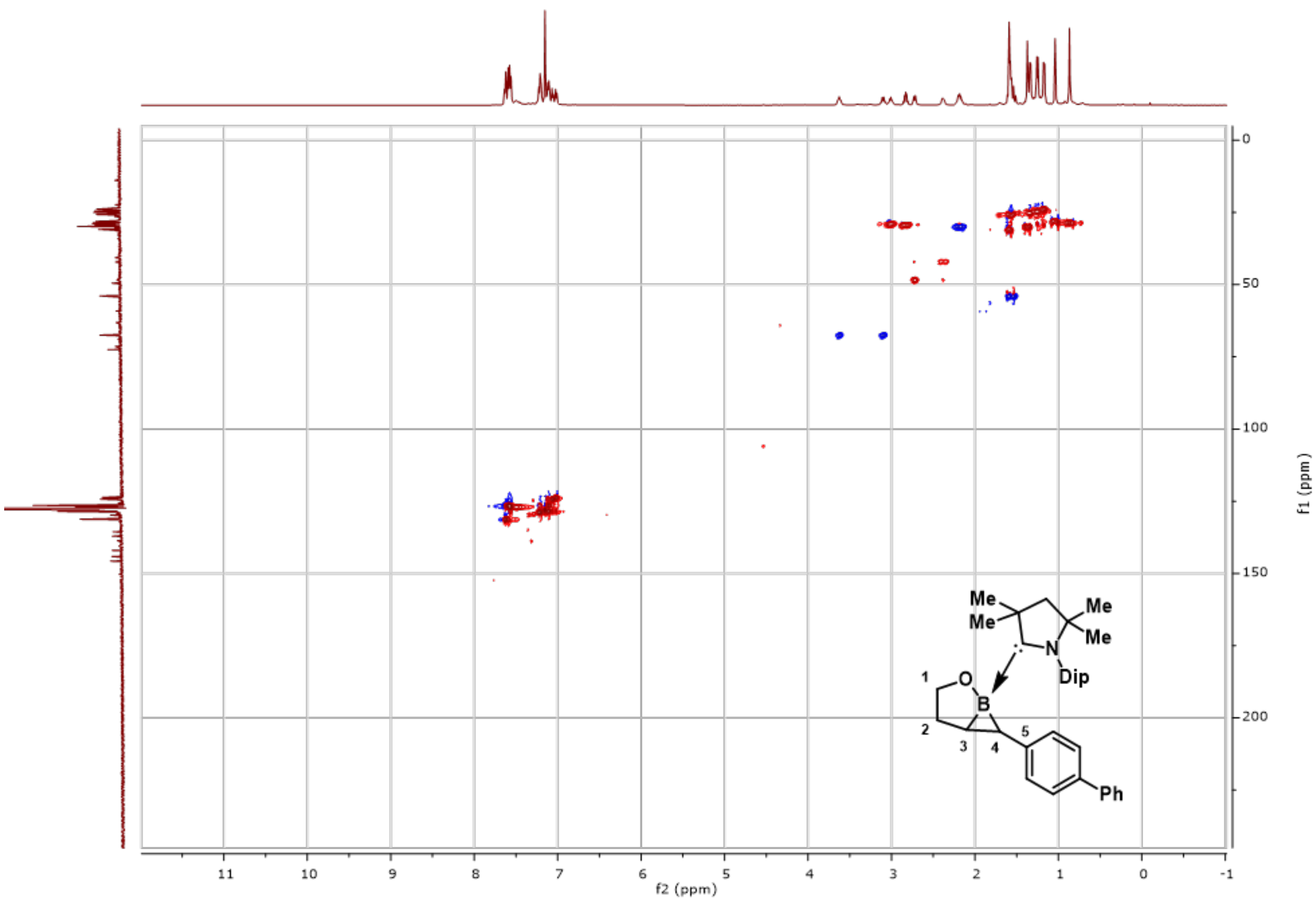

^1H -^{13}C HMBC (500 MHz, Benzene-d_6) of Compound 7c

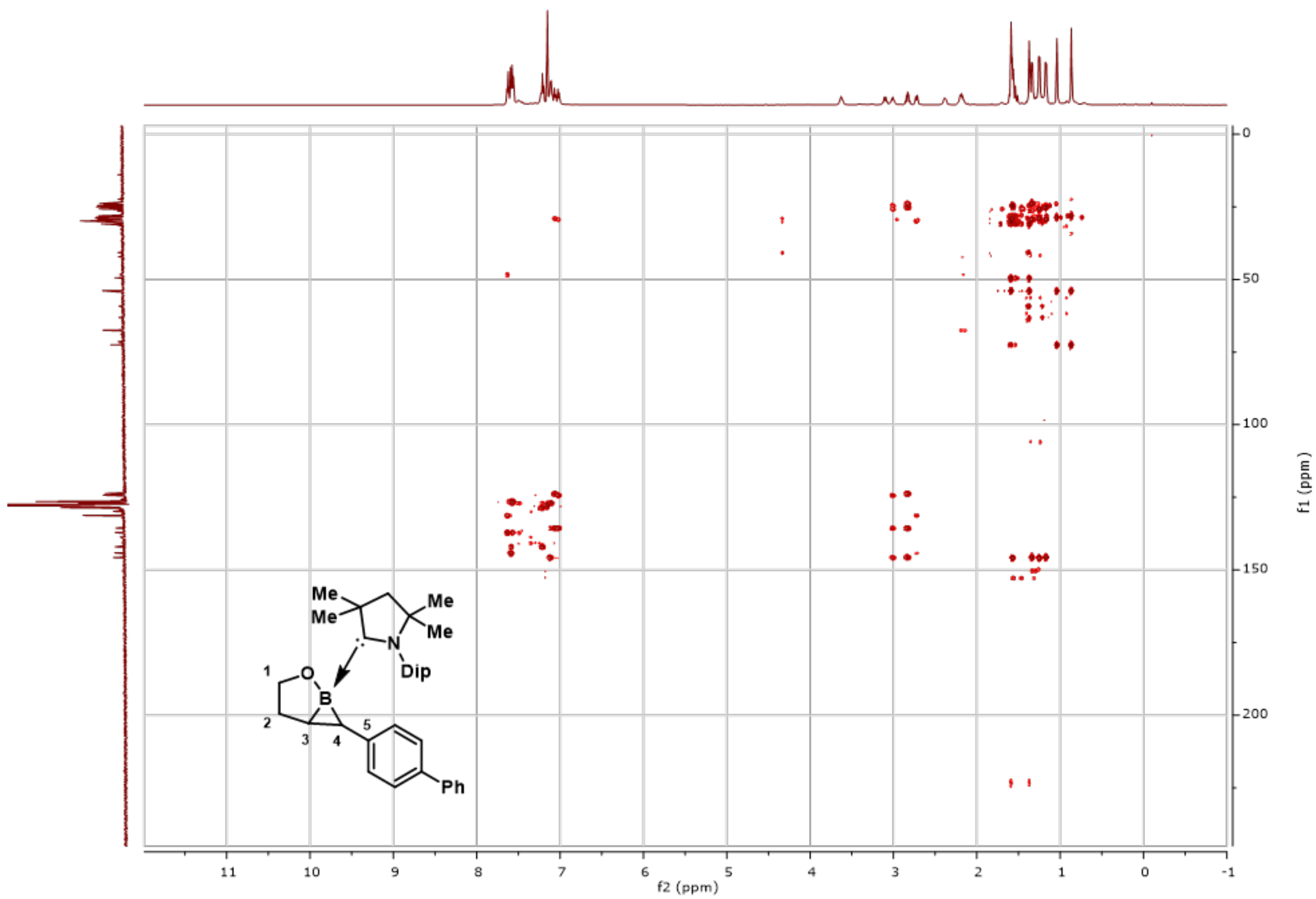

HRMS (ASAP) of Compound 7c

Elemental Composition Report **Page 1**

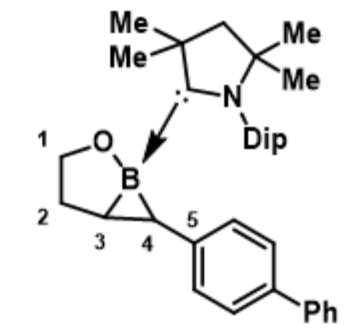

Chemical Formula: $C_{36}H_{46}BNO$
Exact Mass: 519.3672

Single Mass Analysis
Tolerance = 50.0 PPM / DBE: min = -1.5, max = 500.0
Element prediction: Off
Number of isotope peaks used for i-FIT = 3

Monoisotopic Mass, Even Electron Ions
2 formula(e) evaluated with 1 results within limits (up to 50 closest results for each mass)
Elements Used:
C: 36-36 H: 40-50 B: 0-2 N: 1-1 O: 1-2 Na: 0-1
DCM

TOF MS ASAP+
9.74e+006

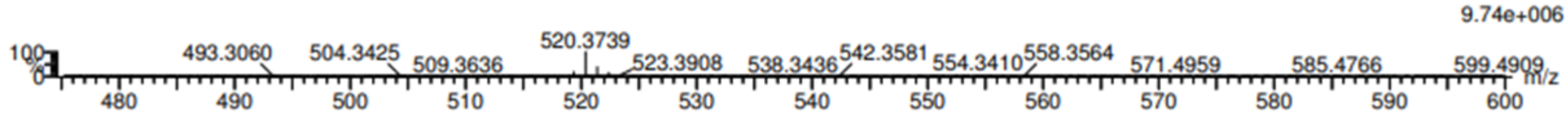

Minimum:				-1.5				
Maximum:		5.0	50.0	500.0				
Mass	Calc. Mass	mDa	PPM	DBE	i-FIT	Norm	Conf(%)	Formula
520.3739	520.3751	-1.2	-2.3	14.5	742.7	n/a	n/a	C36 H47 B N O

^{1}H NMR (500 MHz, Benzene-d_6) of Compound 7d

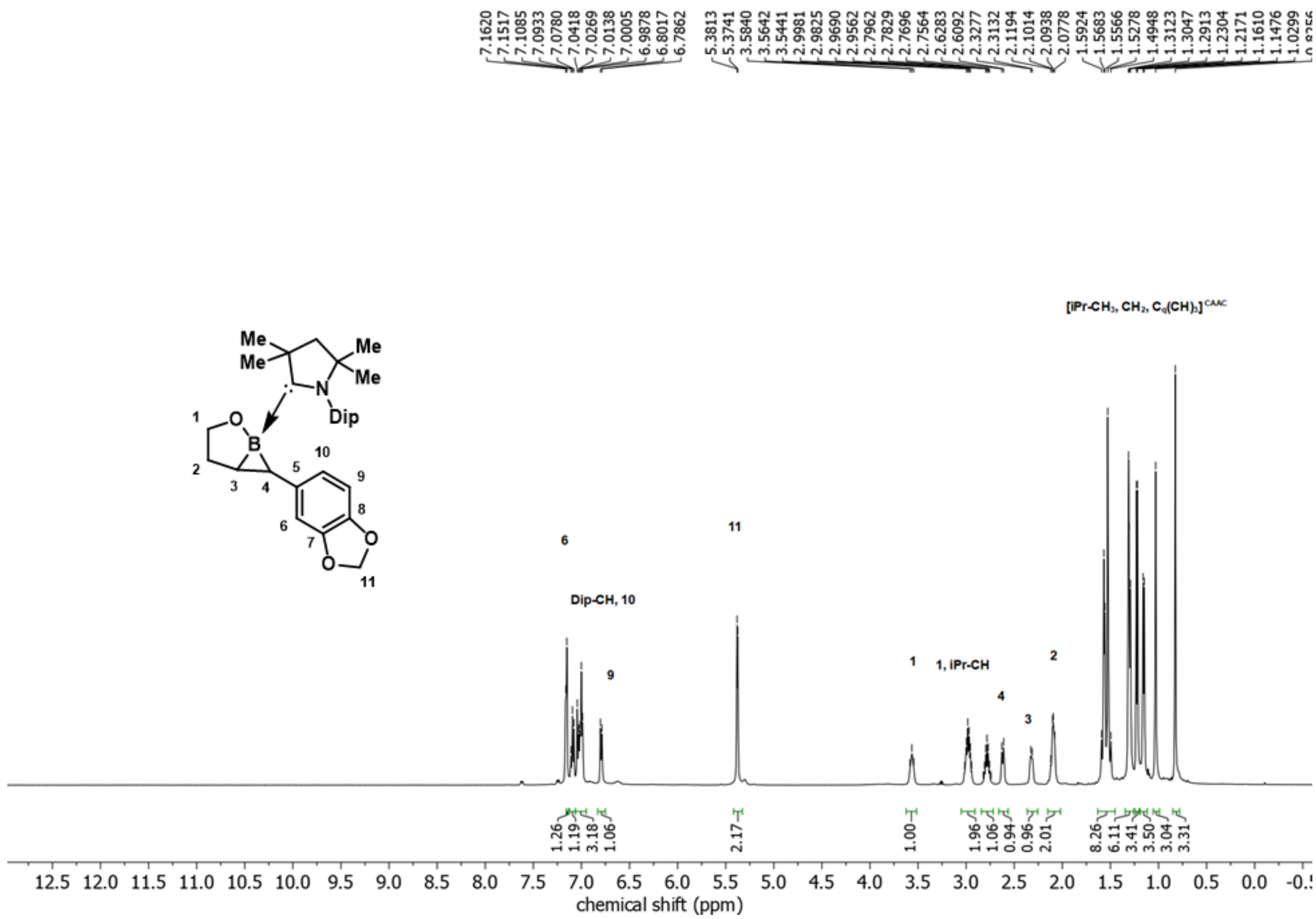

$^{13}C\{^1H\}$ NMR (126 MHz, Benzene-d_6) of Compound 7d

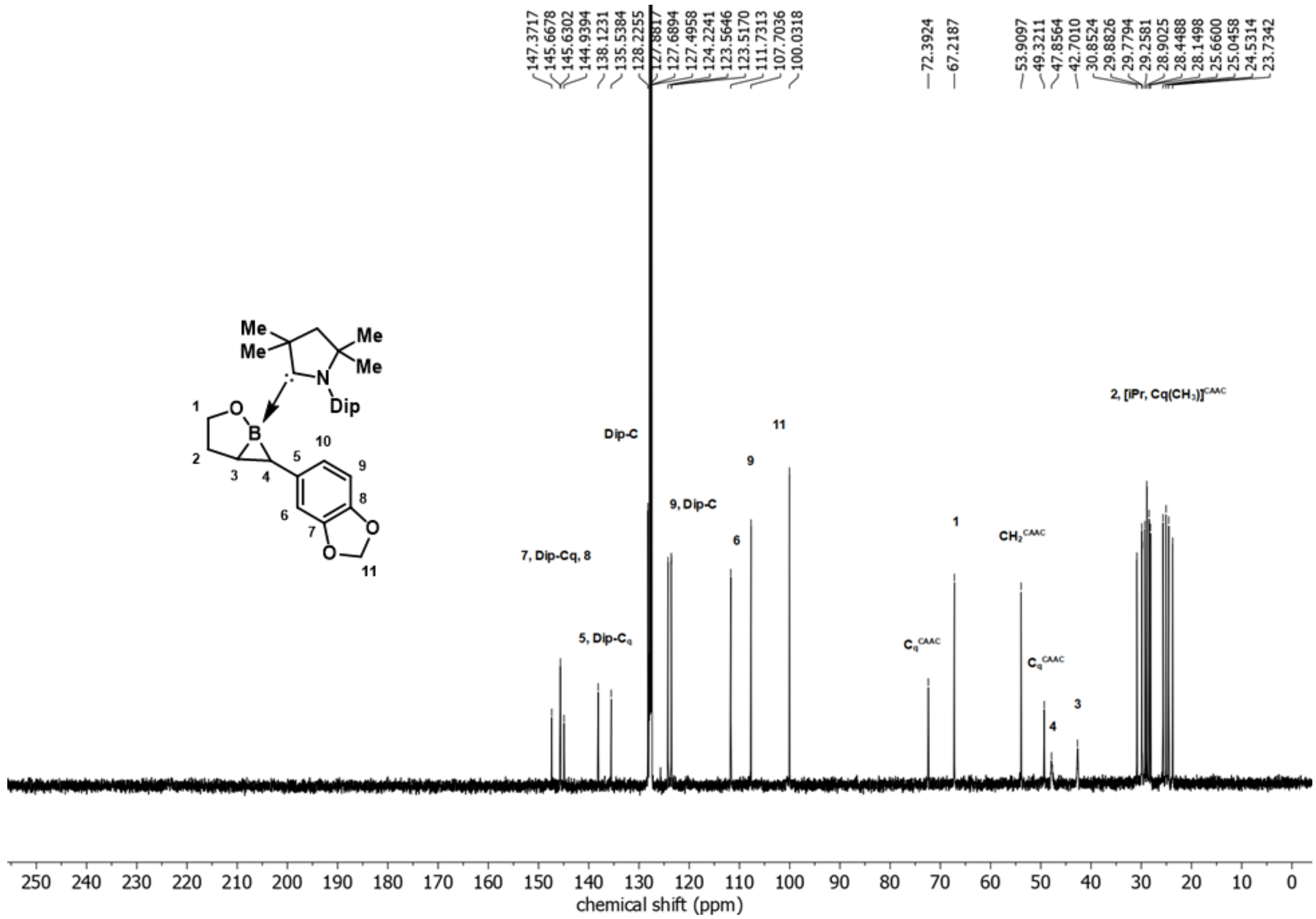

^{11}B NMR (160 MHz, Benzene-d_6) of Compound 7d

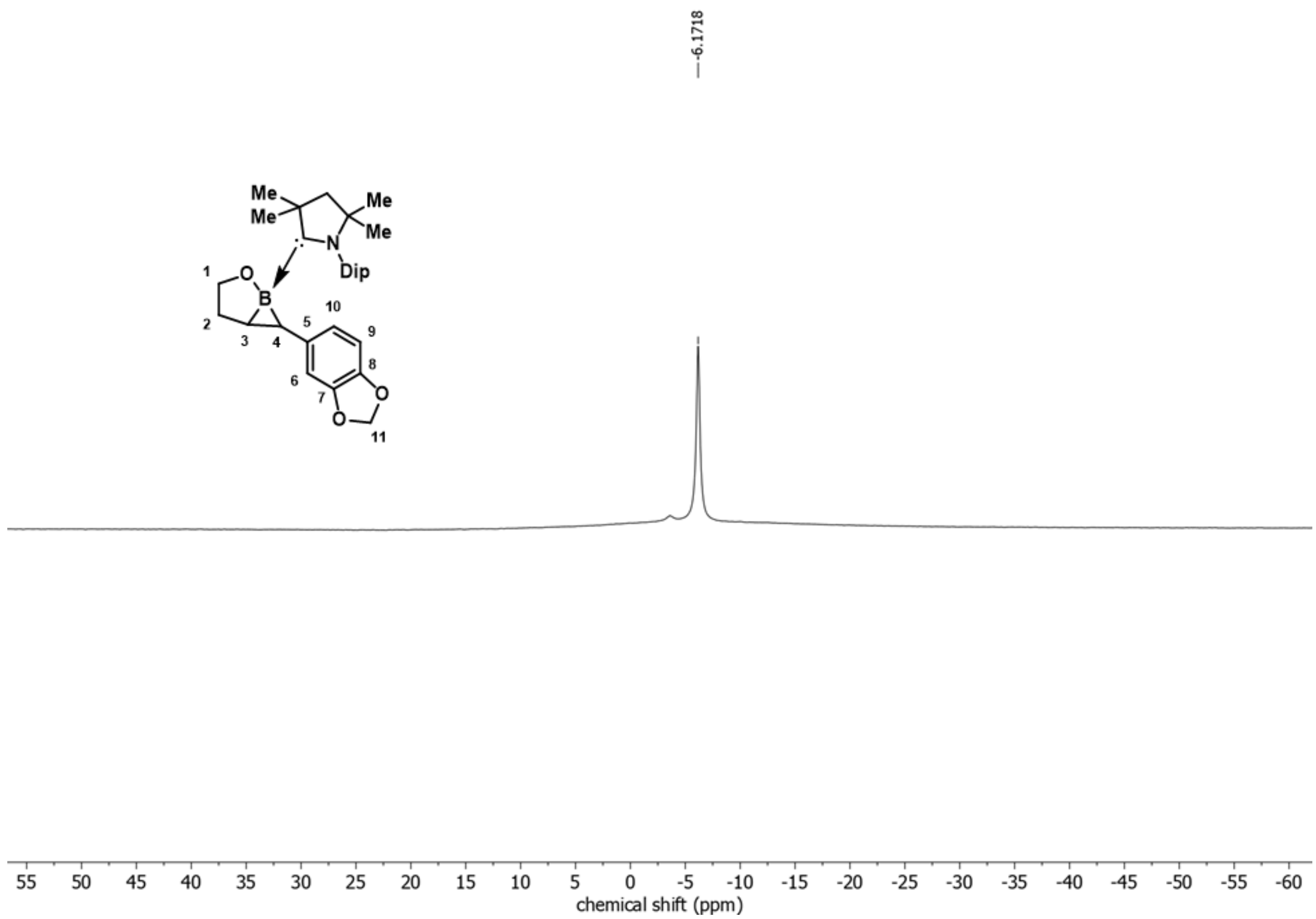

^{1}H -^{1}H COSY (500 MHz, Benzene-d_6) of Compound 7d

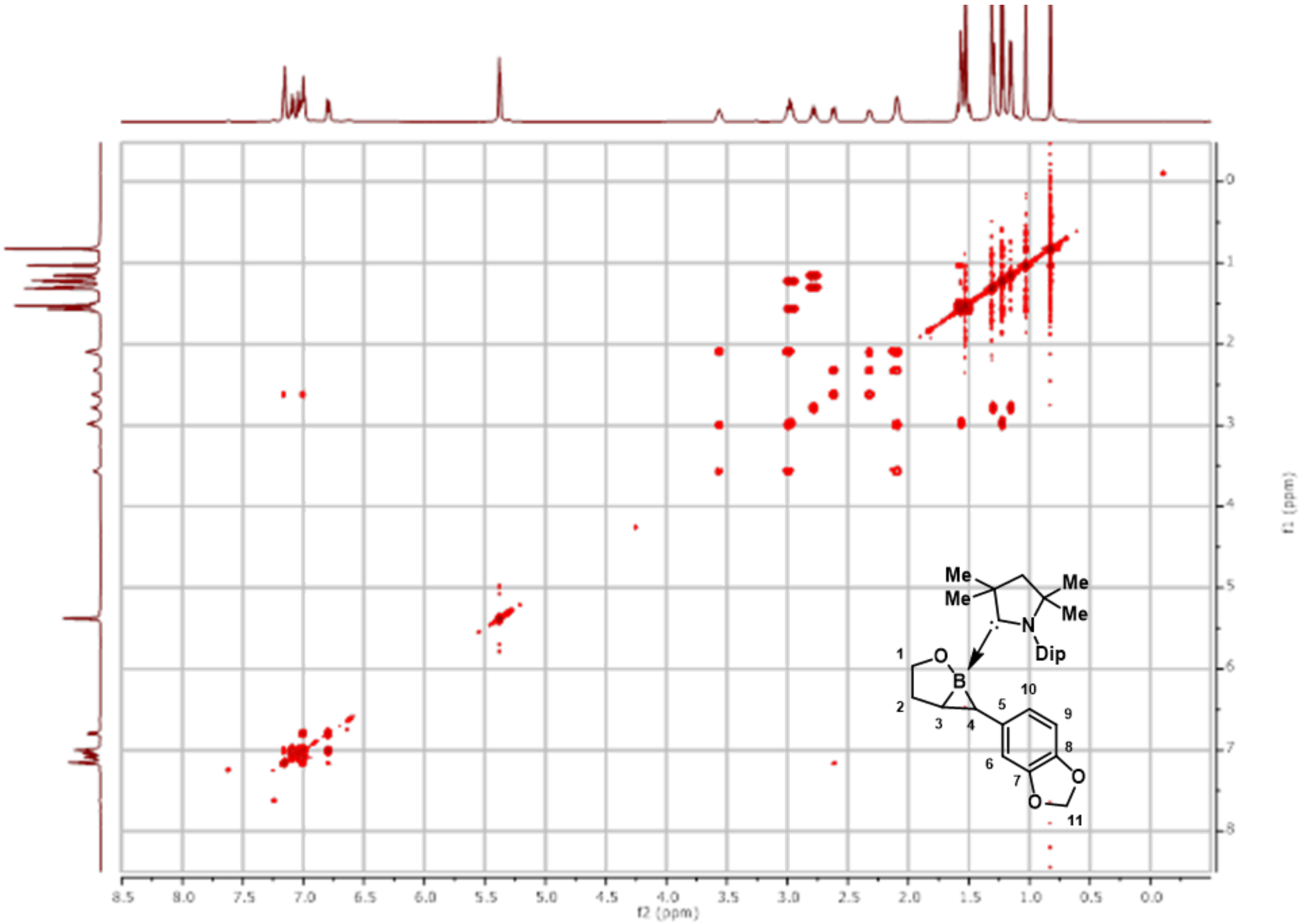

^{1}H -^{13}C HSQC (500 MHz, Benzene-d_6) of Compound 7d

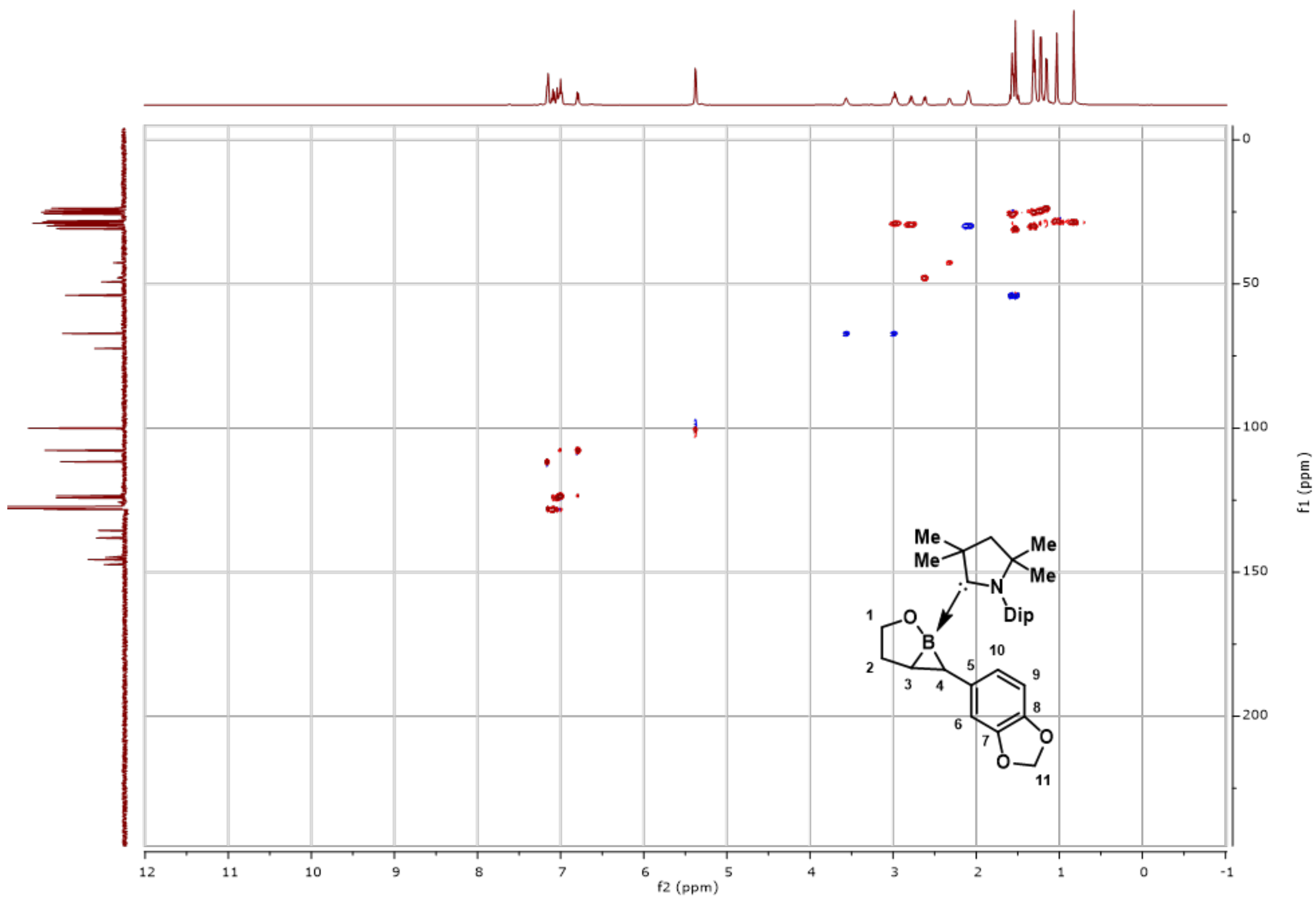

^{1}H -^{13}C HMBC (500 MHz, Benzene-d_6) of Compound 7d

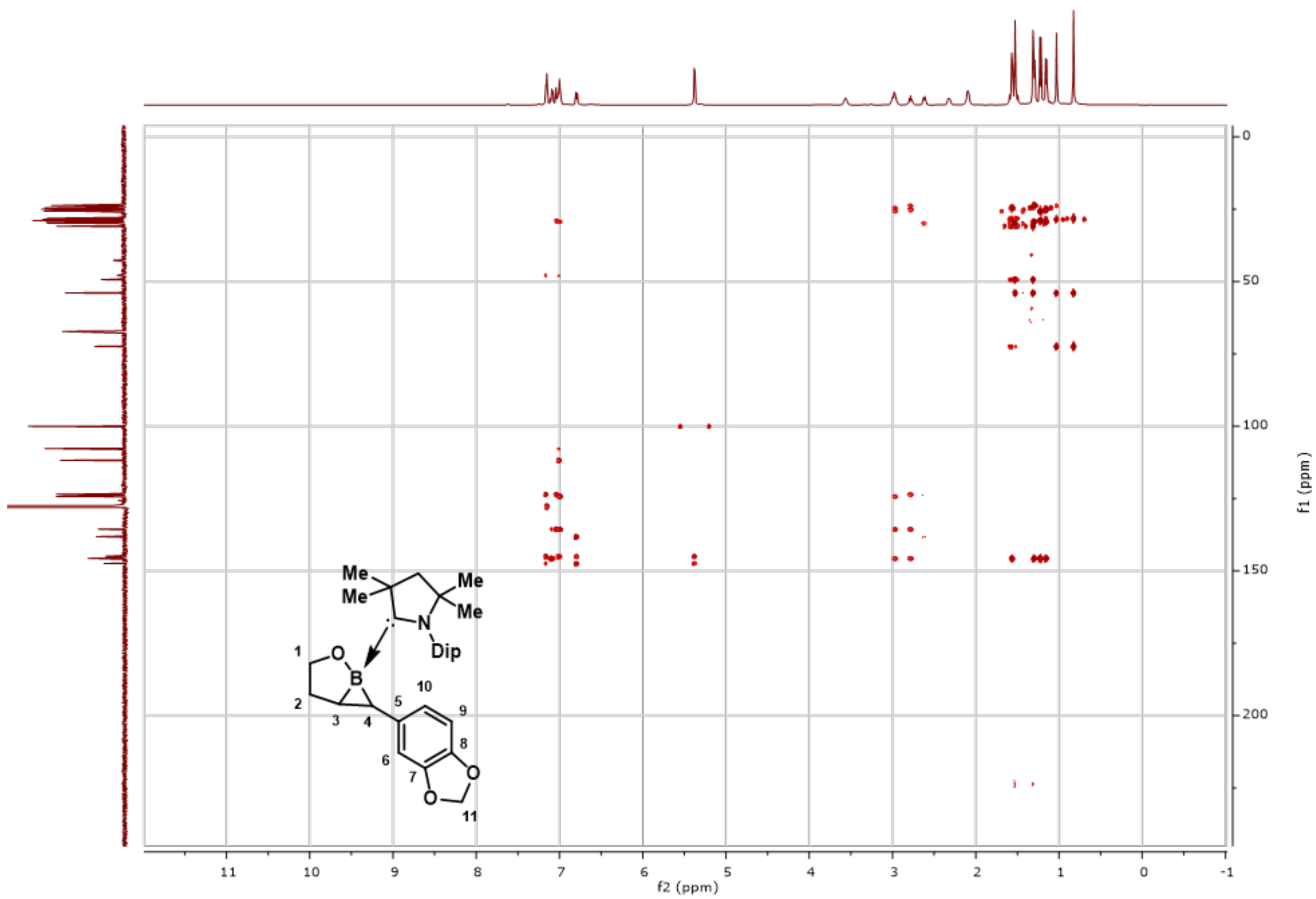

HRMS (ESI) of Compound 7d

Elemental Composition Report

Page 1

Single Mass Analysis
Tolerance = 5.0 PPM / DBE: min = -1.5, max = 500.0
Element prediction: Off
Number of isotope peaks used for i-FIT = 3

Monoisotopic Mass, Even Electron Ions
129 formula(e) evaluated with 1 results within limits (up to 50 closest results for each mass)
Elements Used:
C: 31-31 H: 10-60 B: 0-2 N: 0-8 O: 0-5
DCM

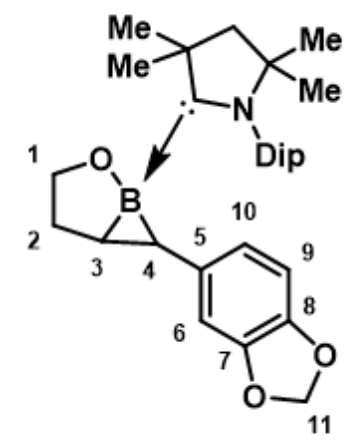

Chemical Formula: $C_{31}H_{42}BNO_3$
Exact Mass: 487.3258

1: TOF MS ES+
1.70e+003

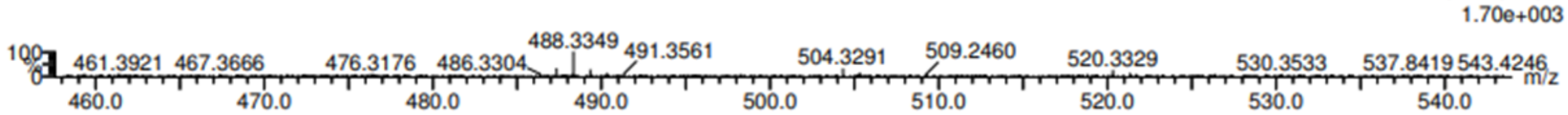

Minimum:				-1.5				
Maximum:		5.0	5.0	500.0				
Mass	Calc. Mass	mDa	PPM	DBE	i-FIT	Norm	Conf(%)	Formula
488.3349	488.3336	1.3	2.7	11.5	153.3	n/a	n/a	C31 H43 B N O3

^{1}H NMR (500 MHz, Benzene-d_6) of Compound 7e

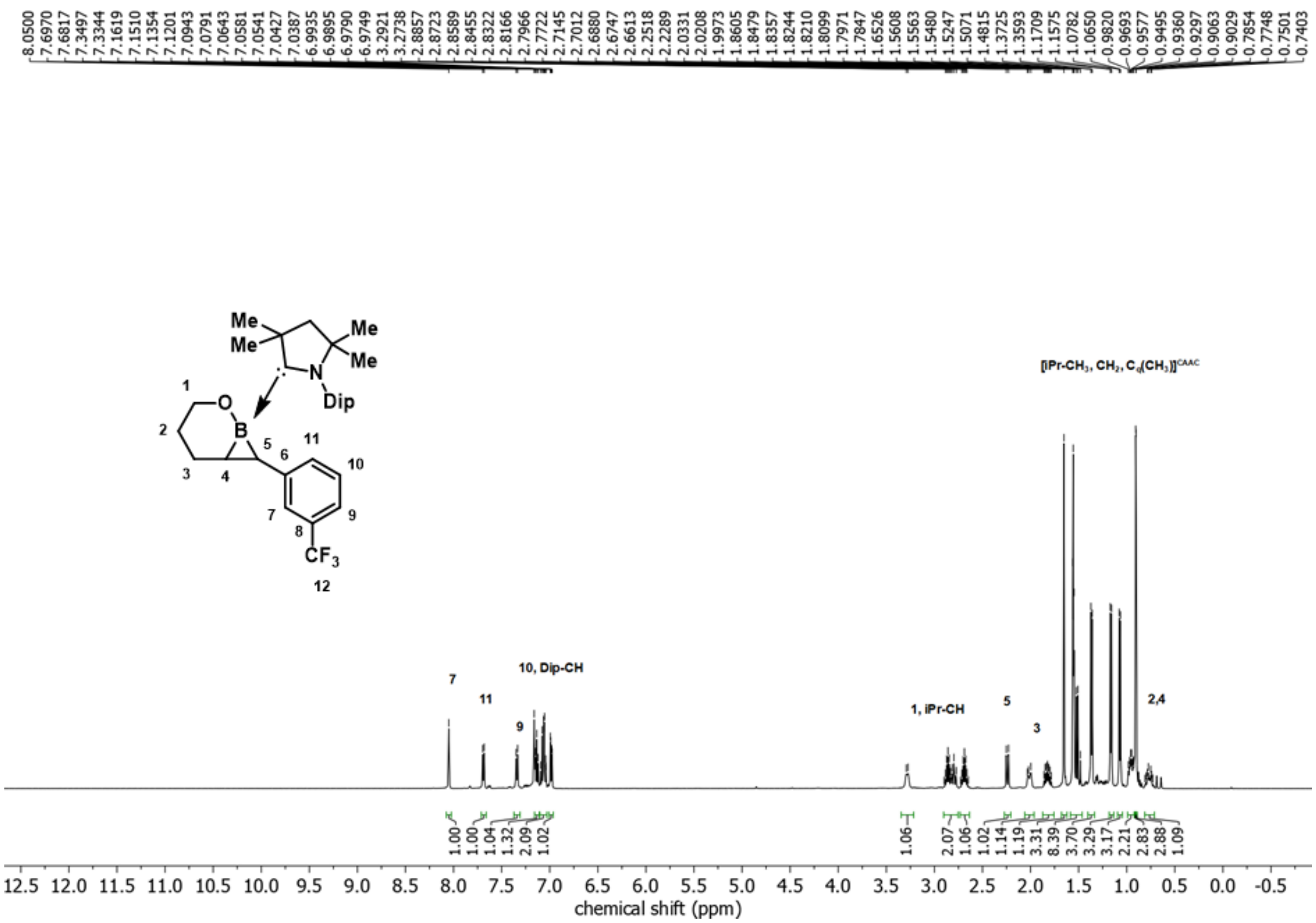

$^{13}C\{^{1}H\}$ NMR (126 MHz, Benzene-d_6) of Compound 7e

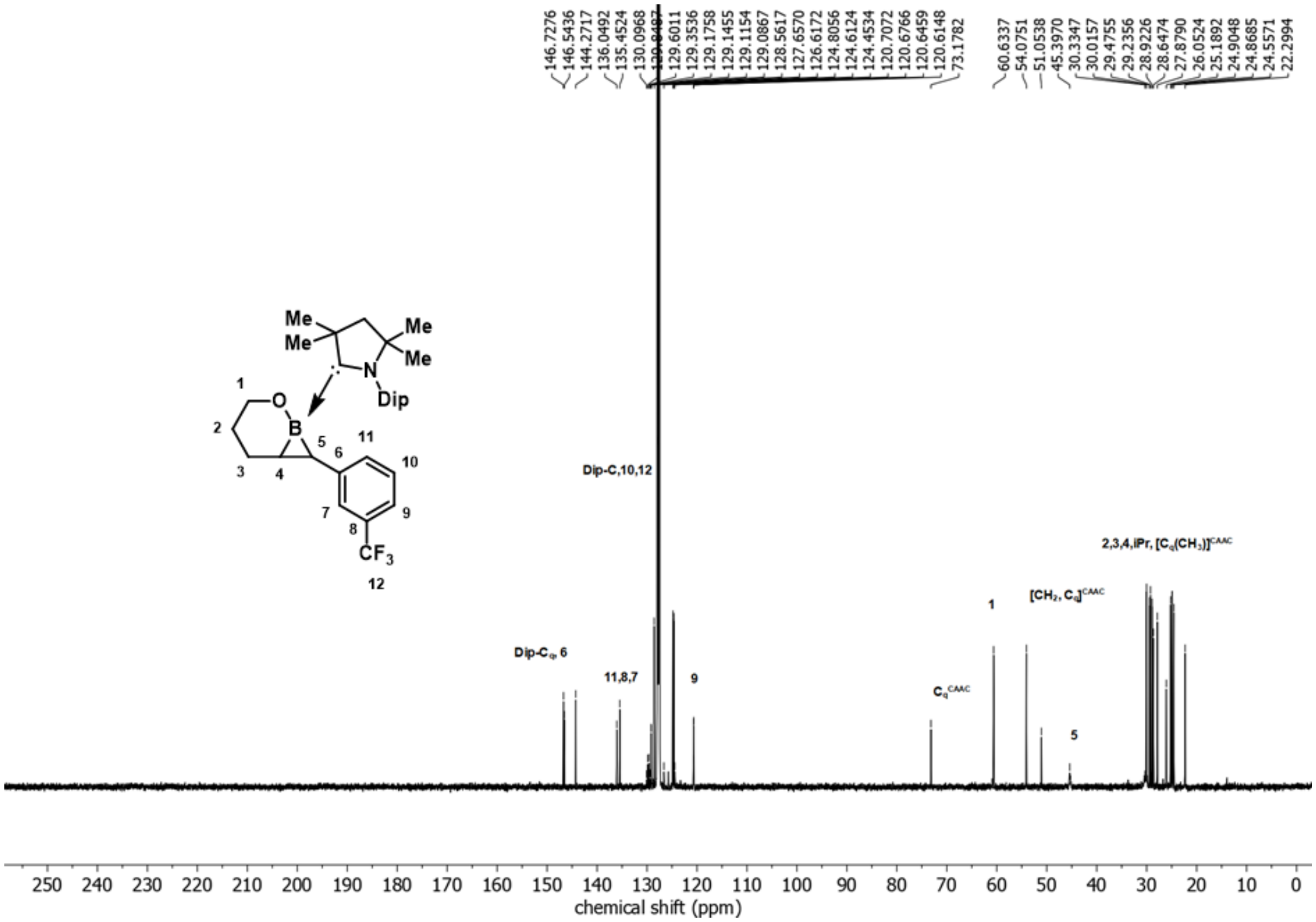

^{19}F NMR (471 MHz, Benzene-d_6) of Compound 7e

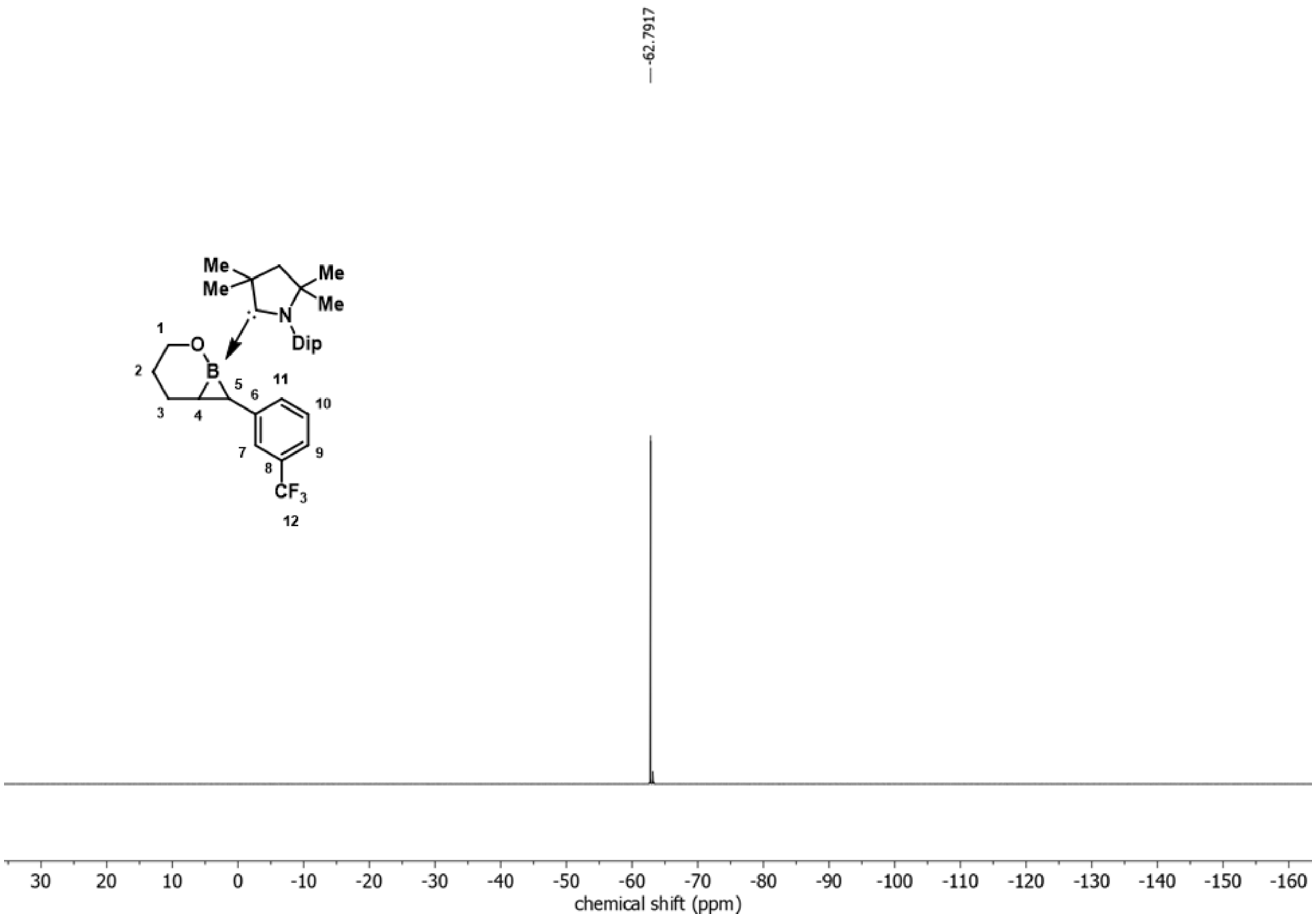

^{11}B NMR (160 MHz, Benzene-*d*$_6$) of Compound 7e

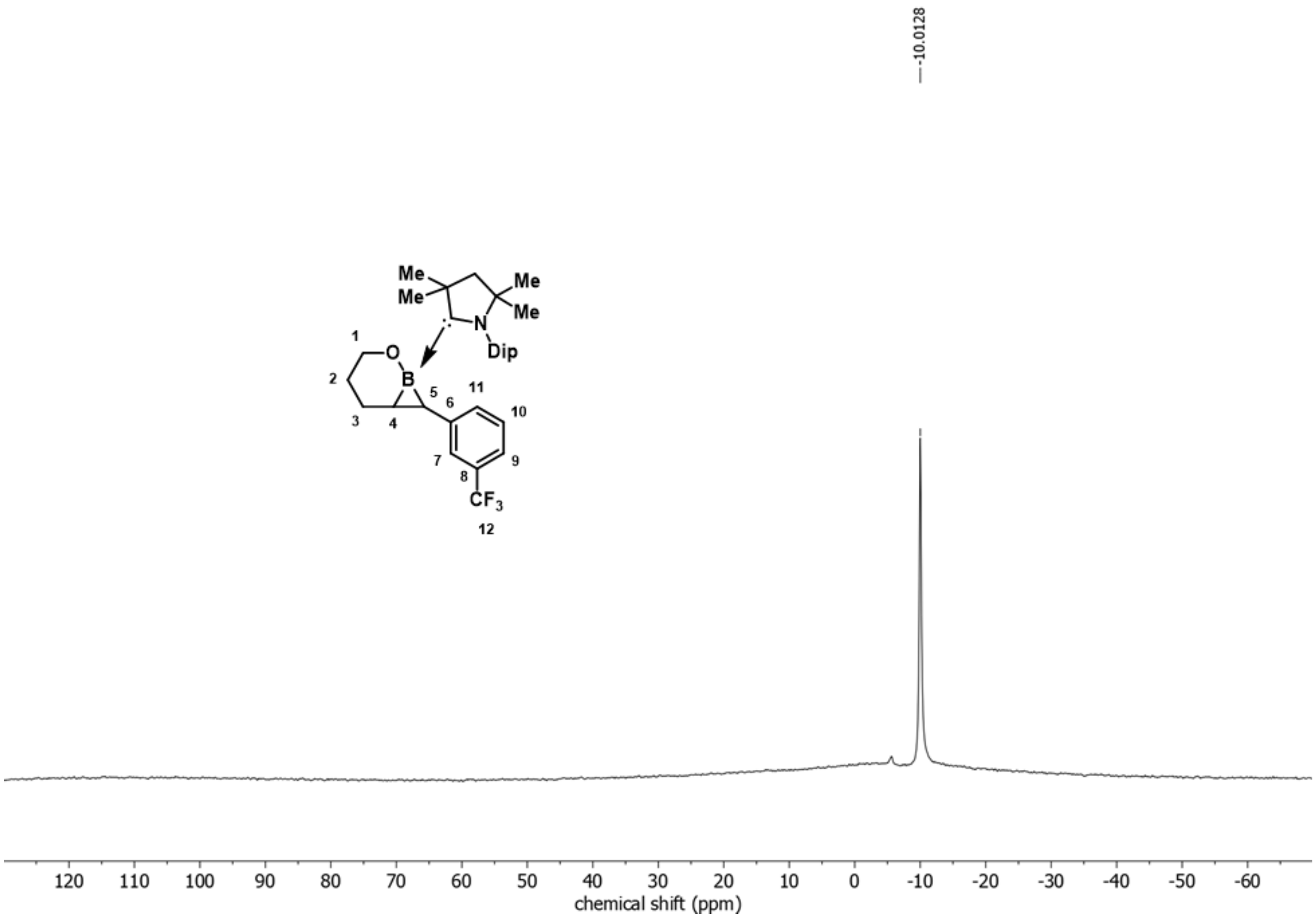

^{1}H -^{1}H COSY (500 MHz, Benzene-d_6) of Compound 7e

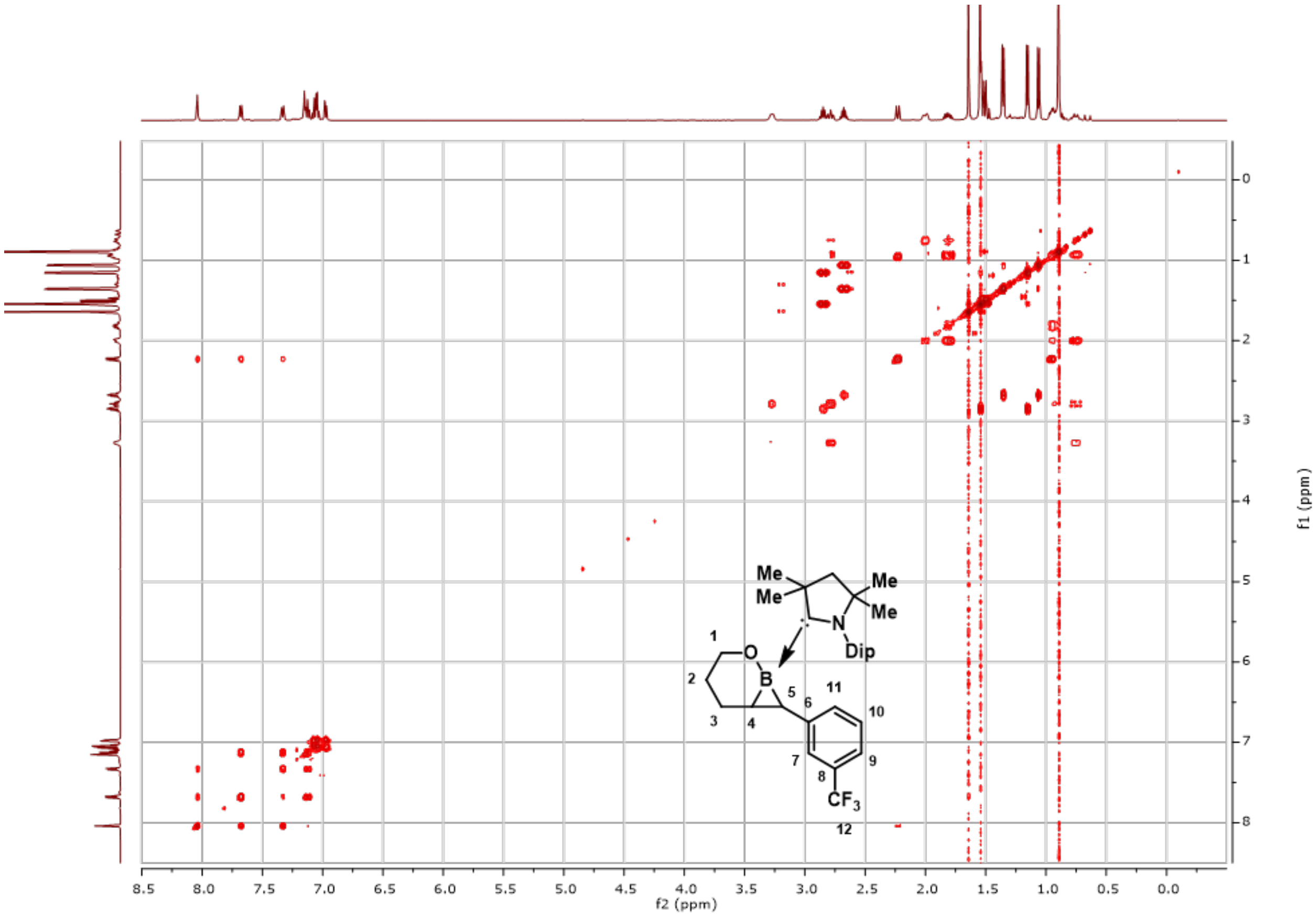

^{1}H -^{13}C HSQC (500 MHz, Benzene-d_6) of Compound 7e

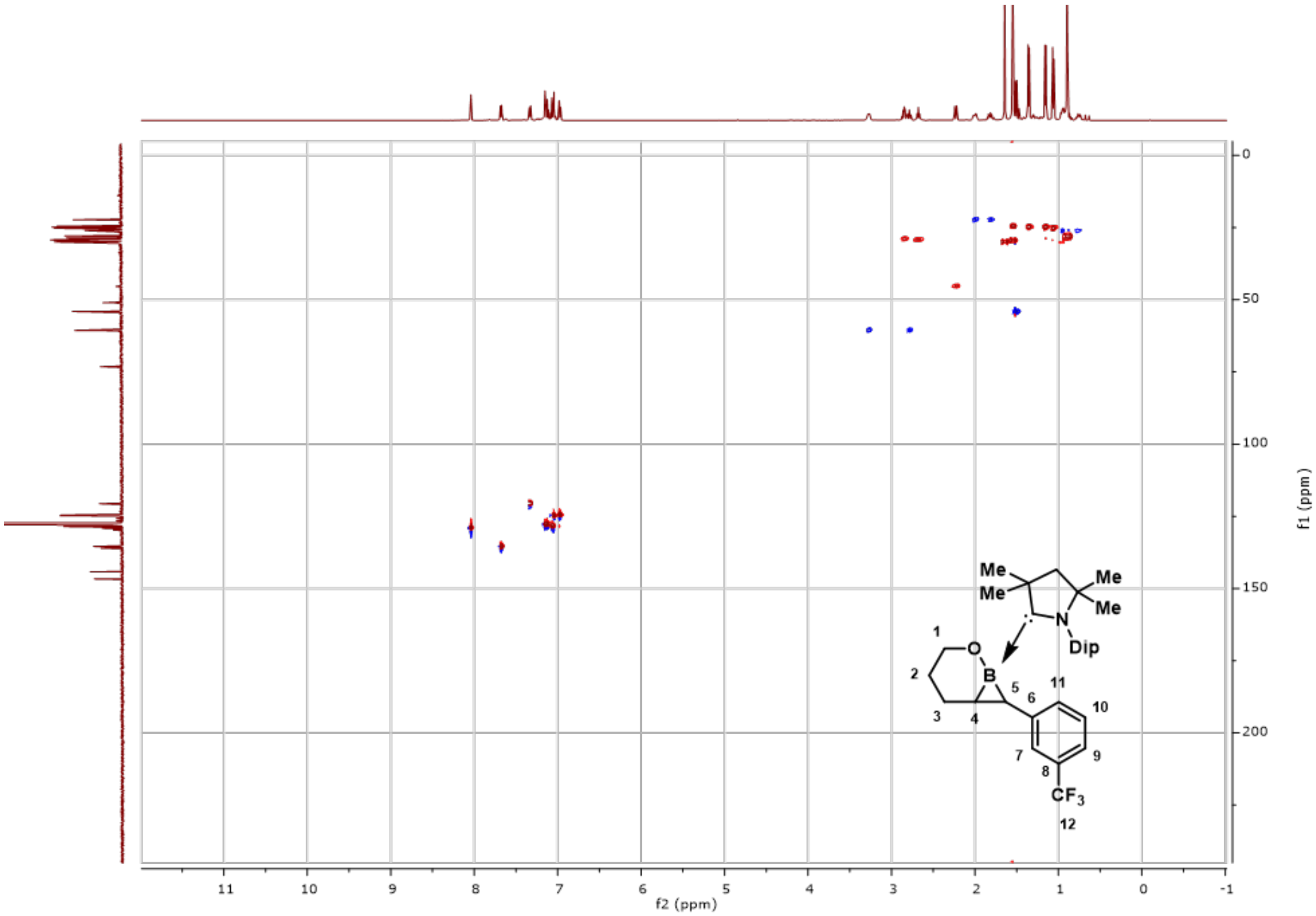

^{1}H -^{13}C HMBC (500 MHz, Benzene-d_6) of Compound 7e

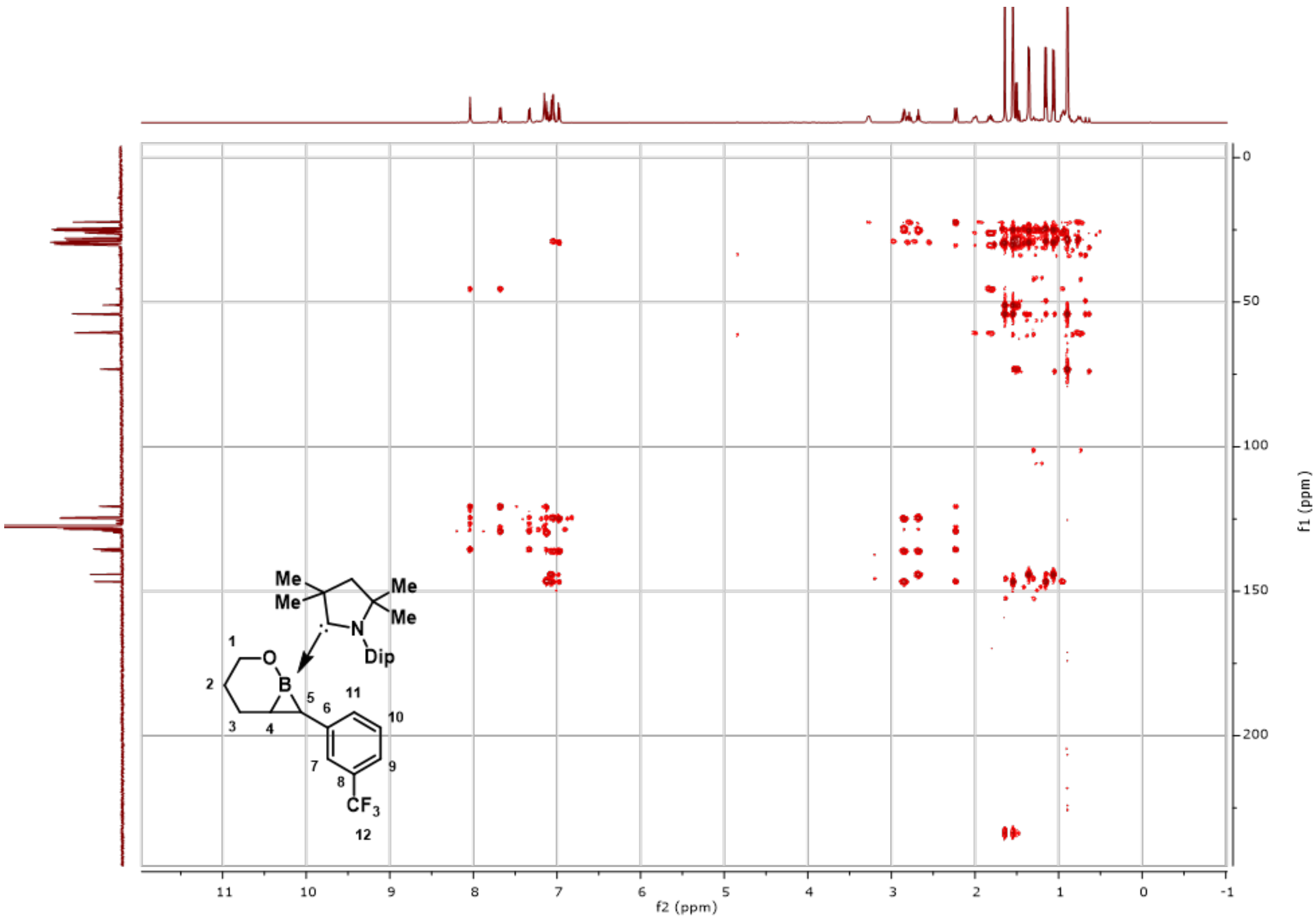

HRMS (ASAP) of Compound 7e

Elemental Composition Report **Page 1**

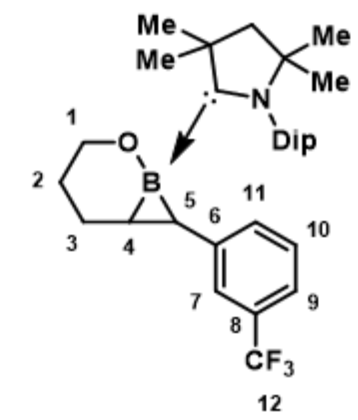

Chemical Formula: $C_{32}H_{43}BF_3NO$
Exact Mass: 525.3390

Single Mass Analysis
Tolerance = 50.0 PPM / DBE: min = -1.5, max = 500.0
Element prediction: Off
Number of isotope peaks used for i-FIT = 3

Monoisotopic Mass, Even Electron Ions
1 formula(e) evaluated with 1 results within limits (up to 50 closest results for each mass)
Elements Used:
C: 32-32 H: 40-50 B: 0-2 N: 1-1 O: 1-1 F: 3-3 Na: 0-1
DCM

TOF MS ASAP+
1.70e+008

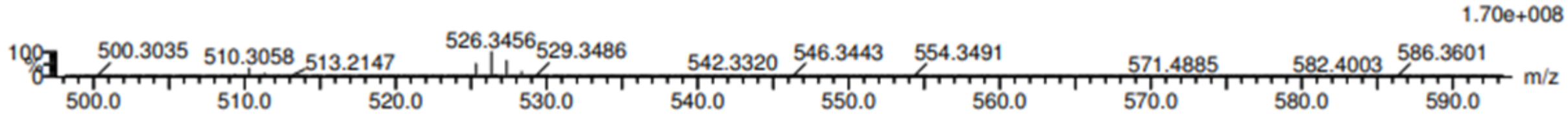

	Mass	Calc. Mass	mDa	PPM	DBE	i-FIT	Norm	Conf(%)	Formula
Minimum:					-1.5				
Maximum:			5.0	50.0	500.0				
	526.3456	526.3468	-1.2	-2.3	10.5	784.1	n/a	n/a	C32 H44 B N O F3

^{1}H NMR (500 MHz, Benzene-d_6) of Compound 7f

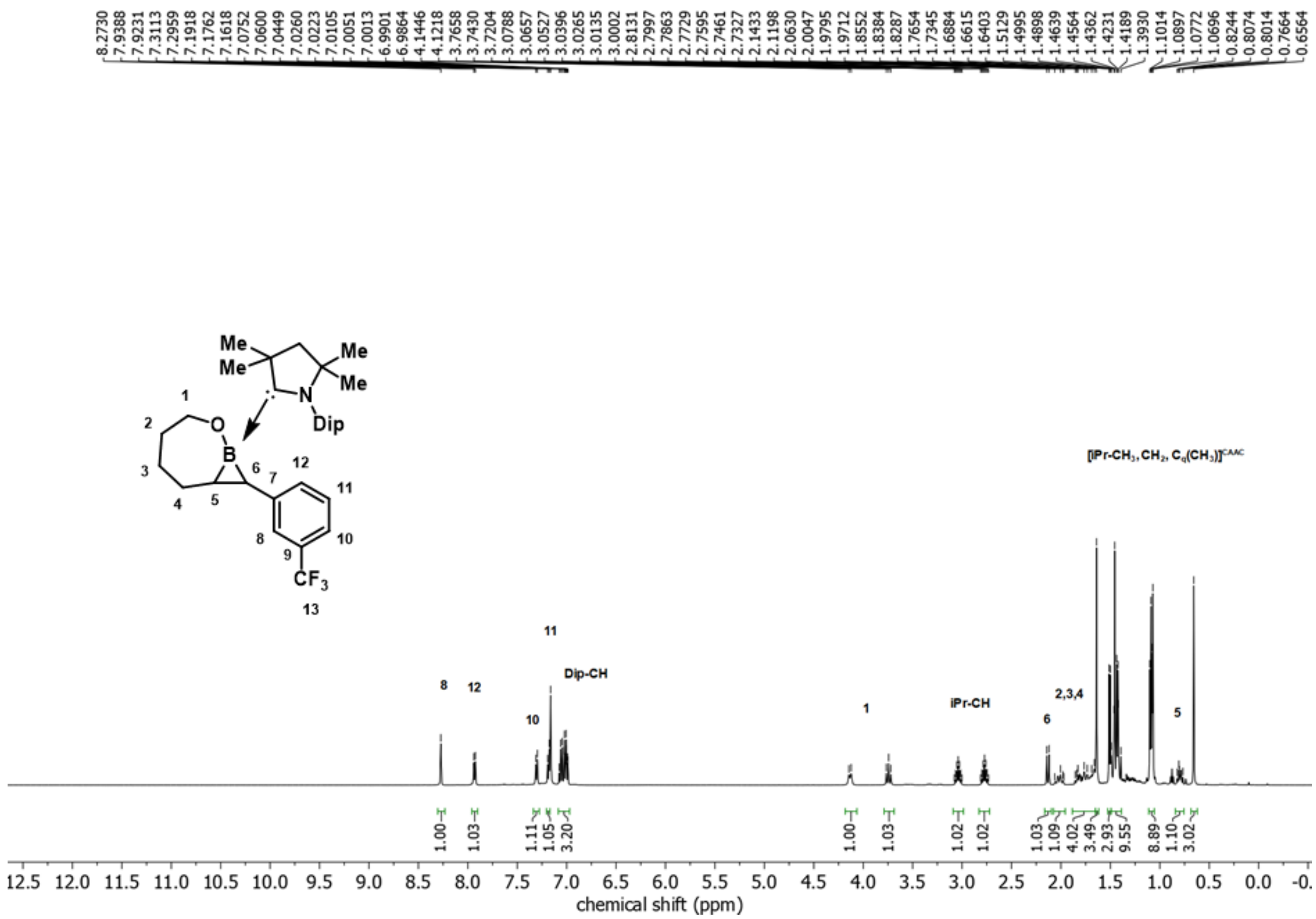

$^{13}C\{^1H\}$ NMR (126 MHz, Benzene-d_6) of Compound 7f

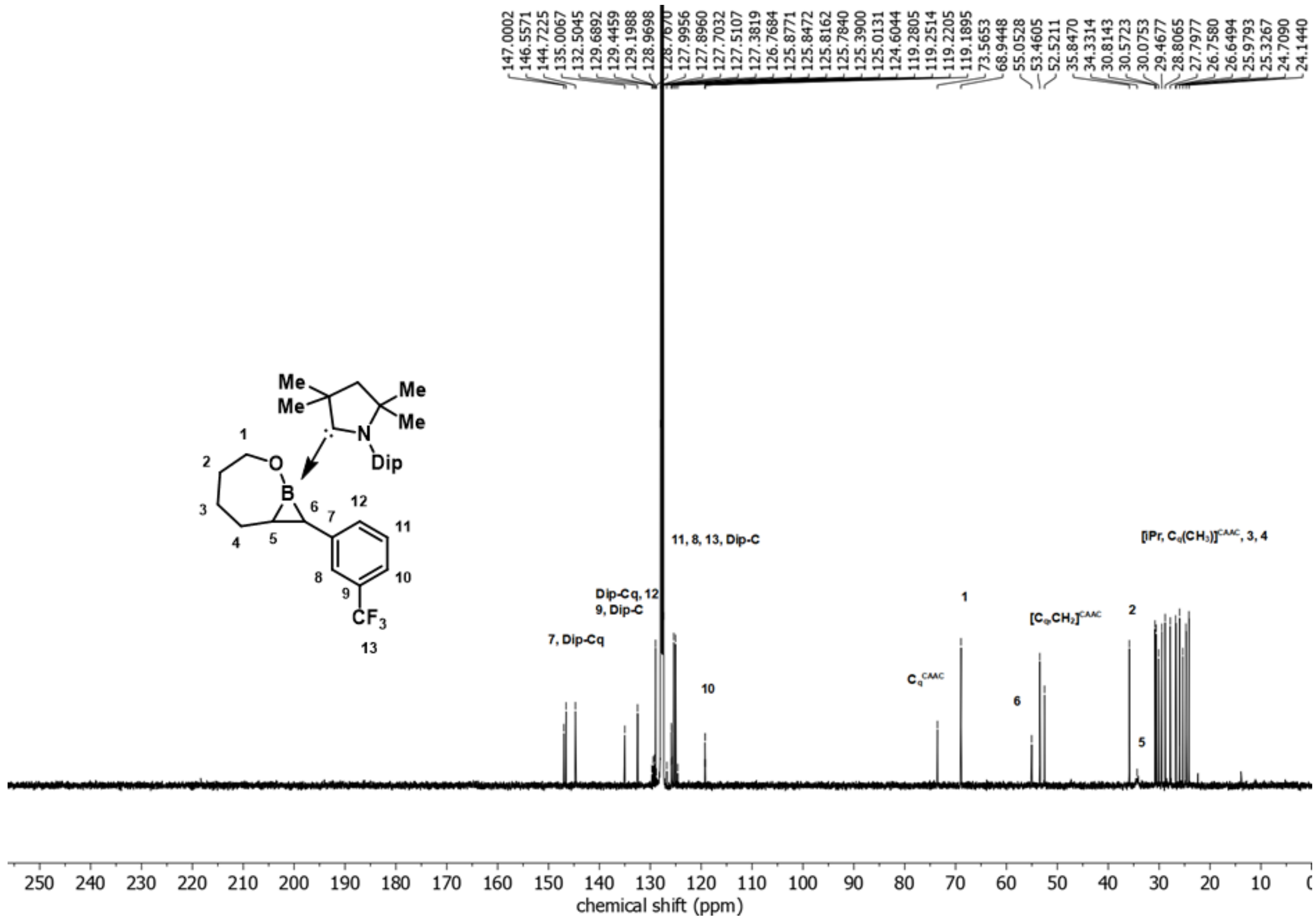

^{19}F NMR (471 MHz, Benzene-d_6) of Compound 7f

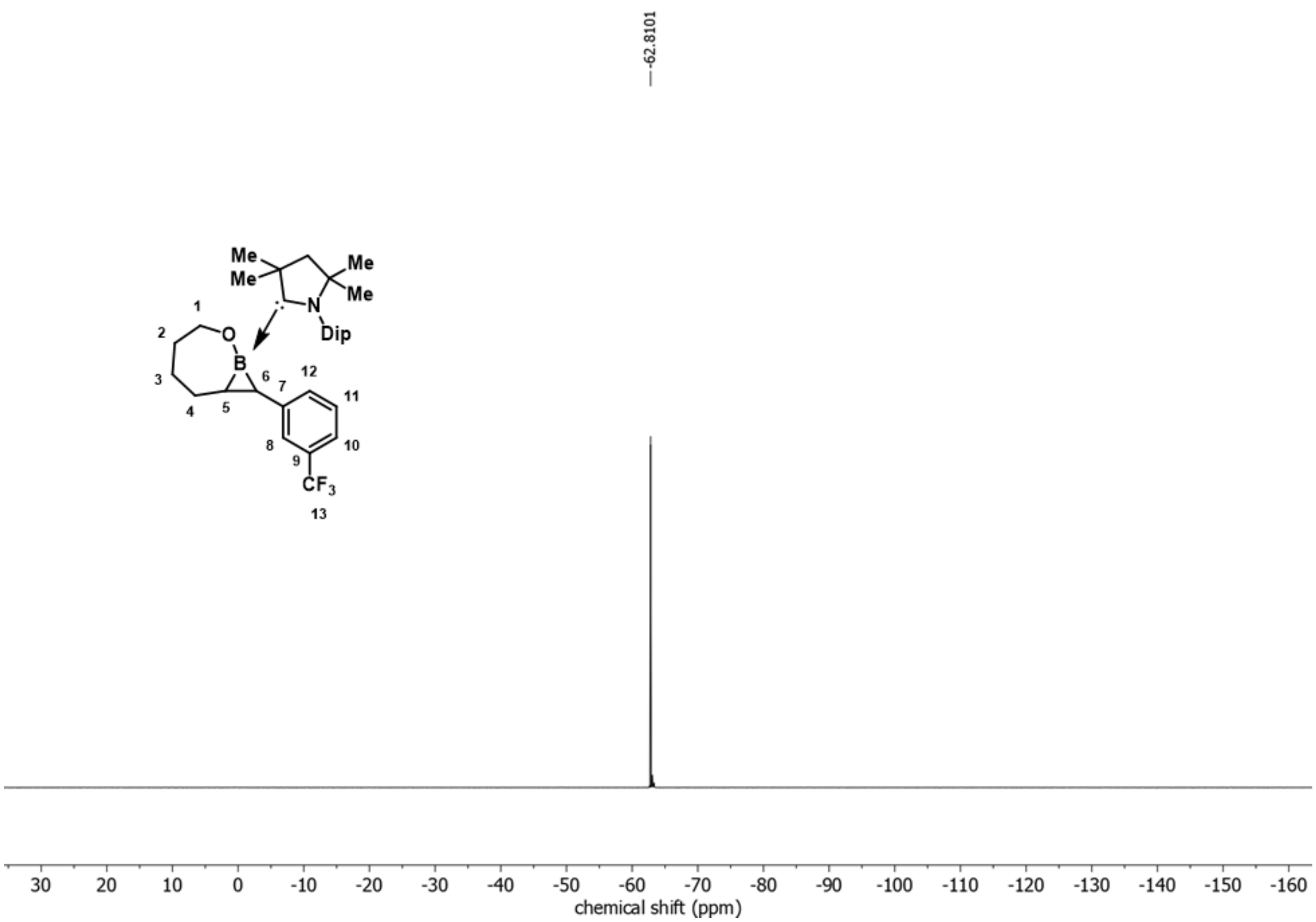

^{11}B NMR (160 MHz, Benzene-d_6) of Compound 7f

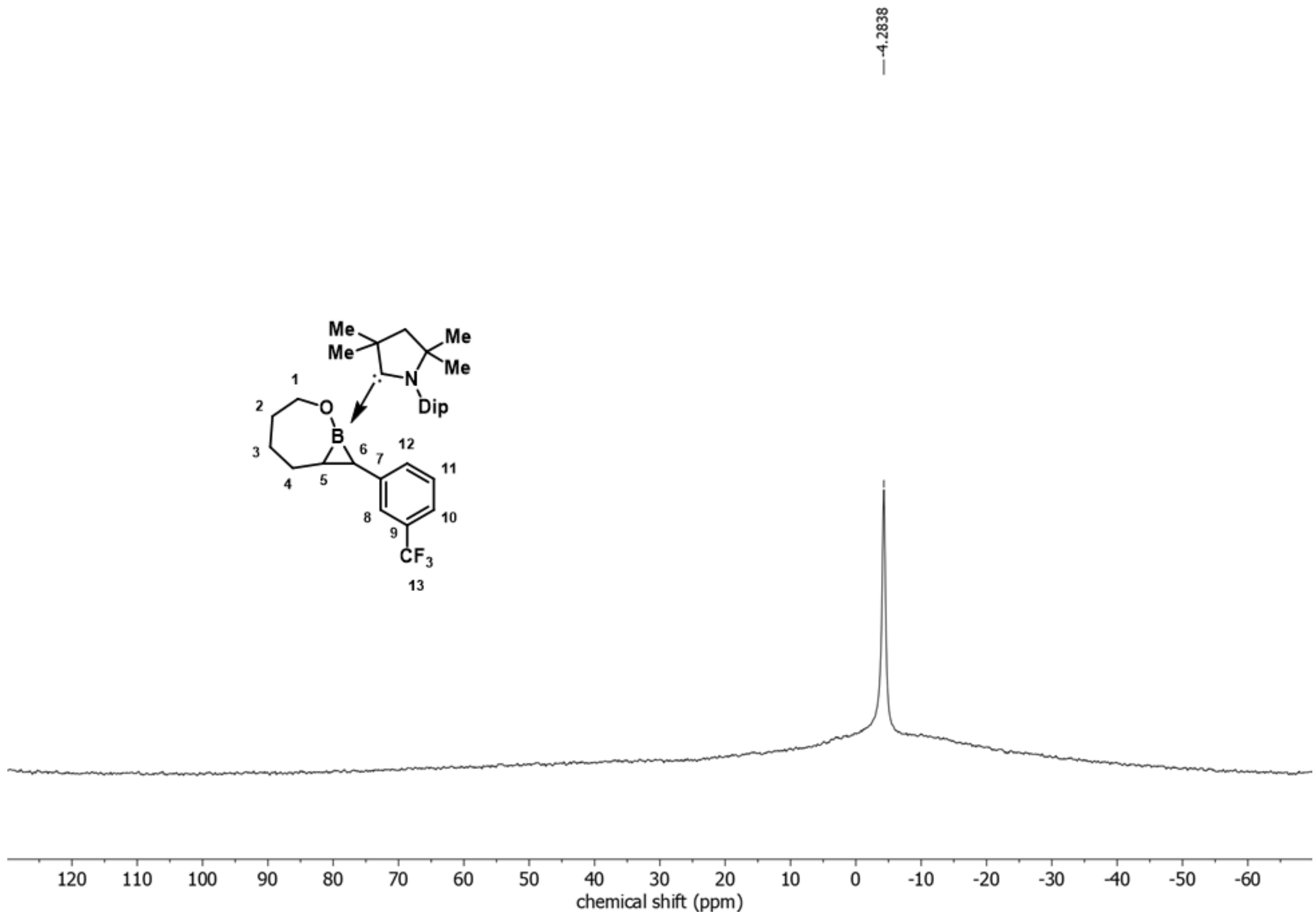

^1H -^1H COSY (500 MHz, Benzene-d_6) of Compound 7f

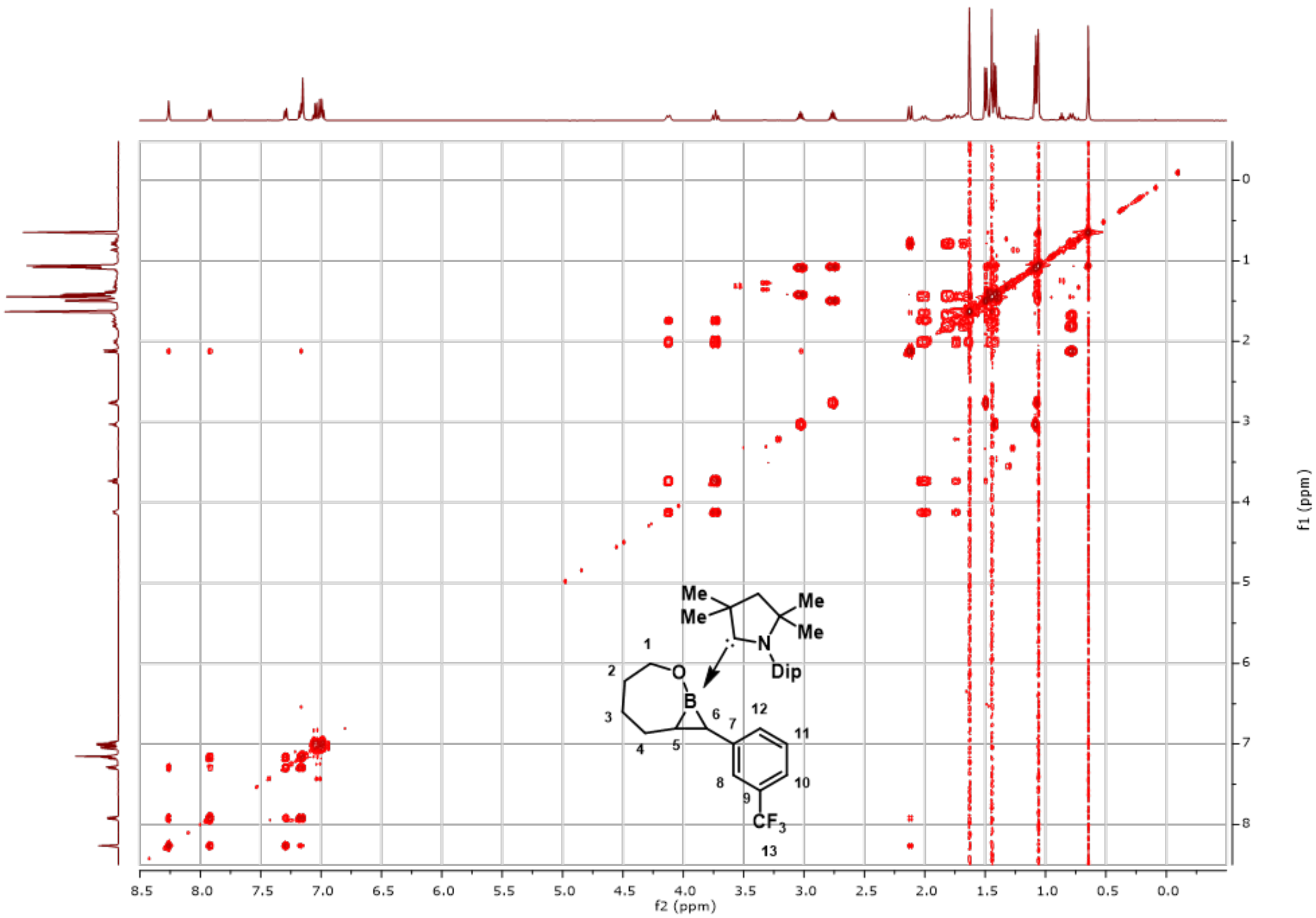

^{1}H -^{13}C HSQC (500 MHz, Benzene-d_6) of Compound 7f

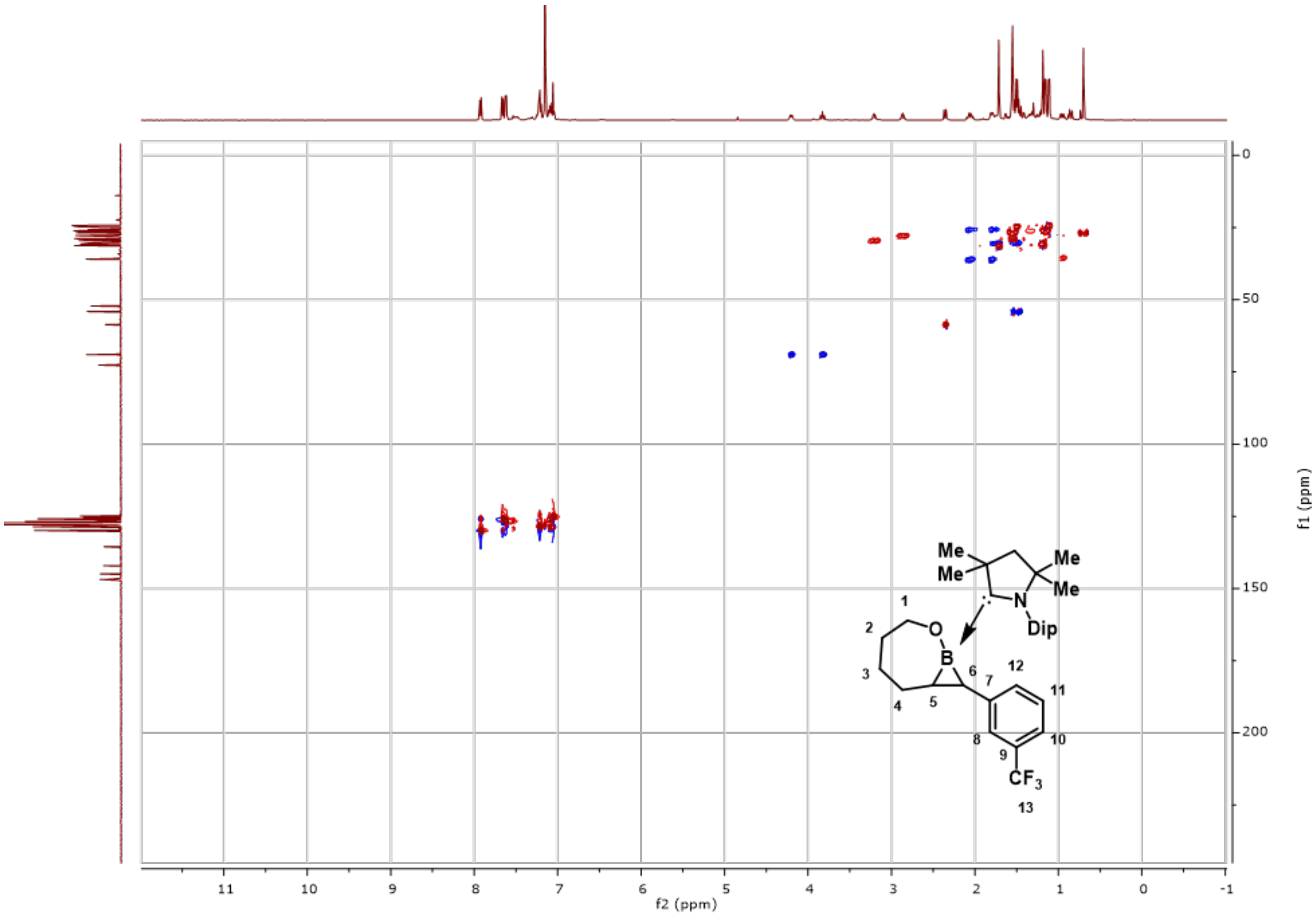

^{1}H -^{13}C HMBC (500 MHz, Benzene-d_6) of Compound 7f

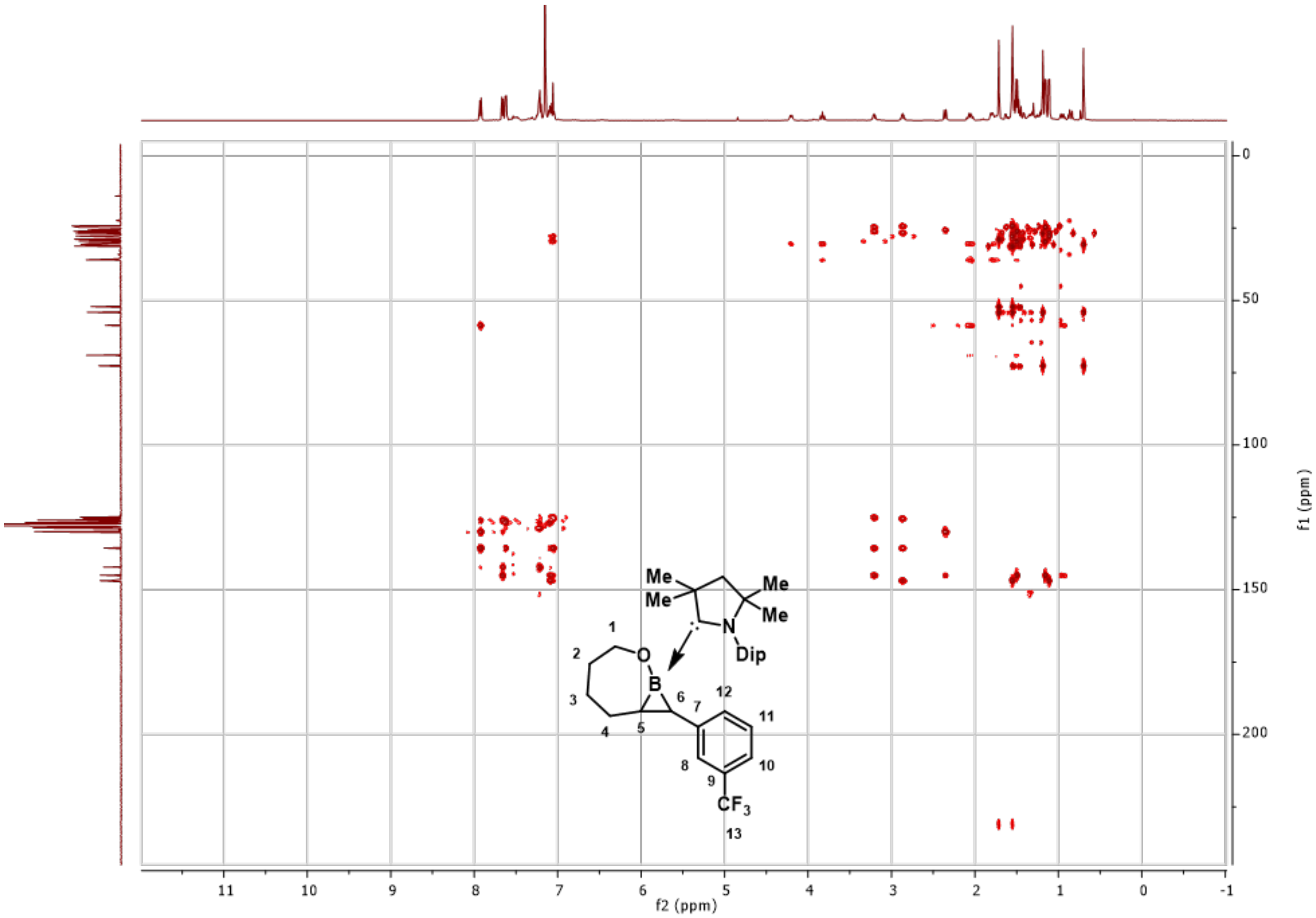

HRMS (ASAP) of Compound 7f

Elemental Composition Report

Page 1

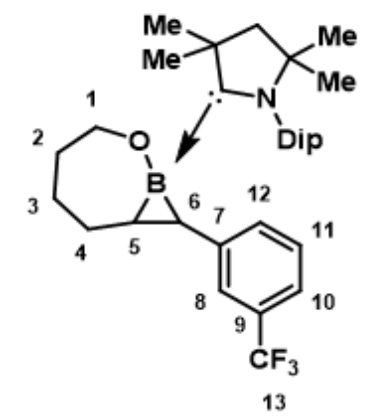

Chemical Formula: $C_{33}H_{45}BF_3NO$
Exact Mass: 539.3546

Single Mass Analysis
Tolerance = 5.0 PPM / DBE: min = -1.5, max = 500.0
Element prediction: Off
Number of isotope peaks used for i-FIT = 3

Monoisotopic Mass, Even Electron Ions
3 formula(e) evaluated with 1 results within limits (up to 50 closest results for each mass)
Elements Used:
C: 33-33 H: 40-50 B: 0-2 N: 1-1 O: 1-1 F: 1-3 Na: 0-1
DCM

TOF MS ASAP+
2.24e+007

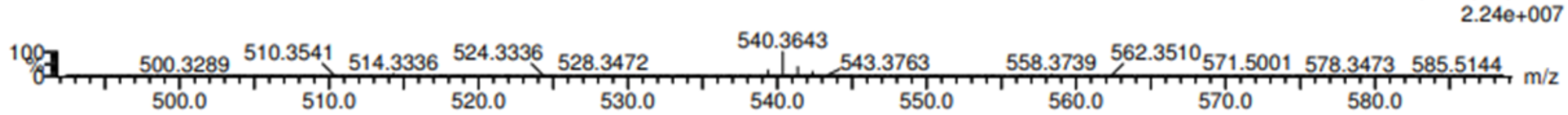

Minimum:				-1.5				
Maximum:		5.0	5.0	500.0				
Mass	Calc. Mass	mDa	PPM	DBE	i-FIT	Norm	Conf(%)	Formula
540.3643	540.3625	1.8	3.3	10.5	848.7	n/a	n/a	C33 H46 B N O F3

^{1}H NMR (500 MHz, Benzene-d_6) of Compound 7g

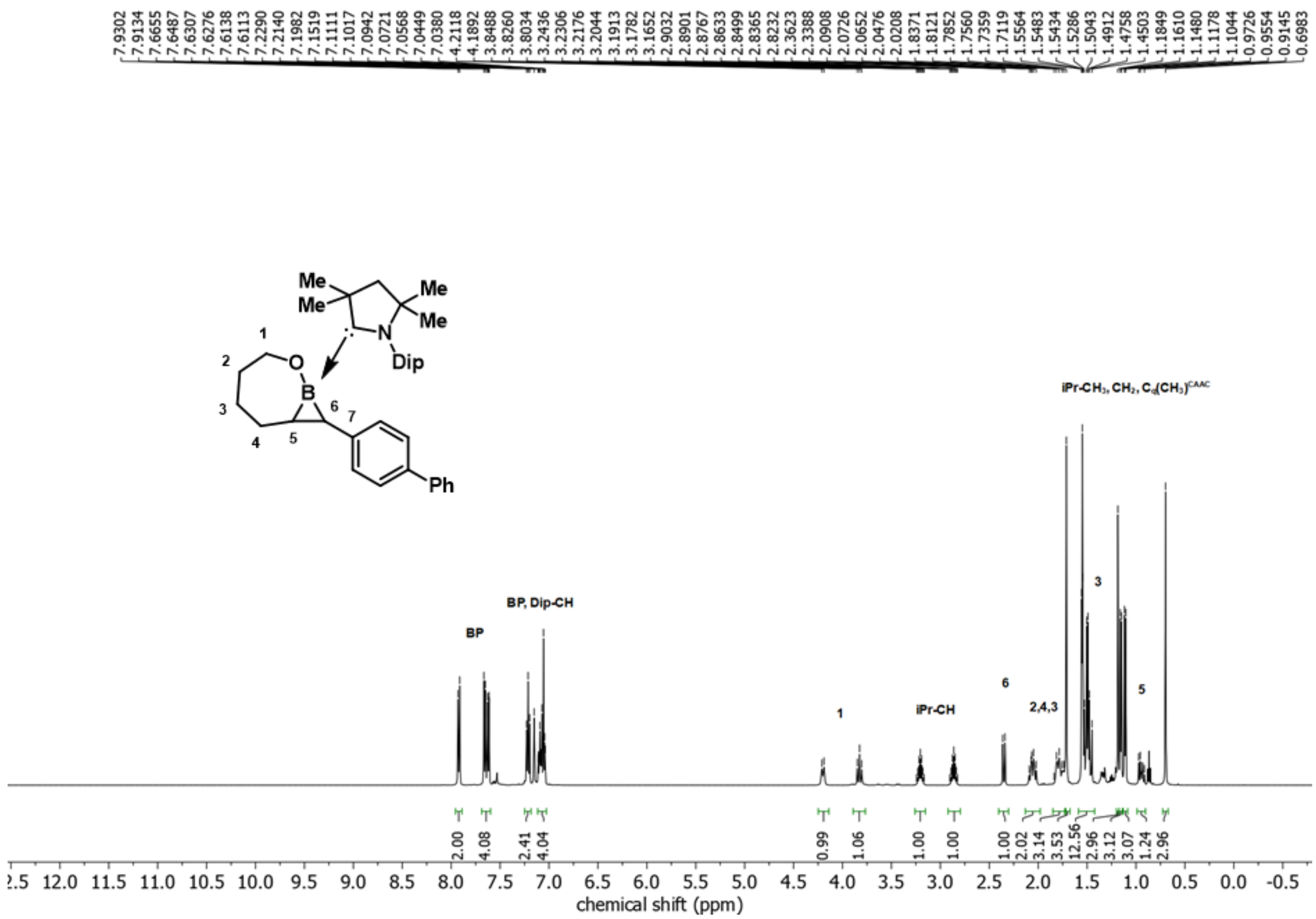

$^{13}C\{^1H\}$ NMR (126 MHz, Benzene-d_6) of Compound 7g

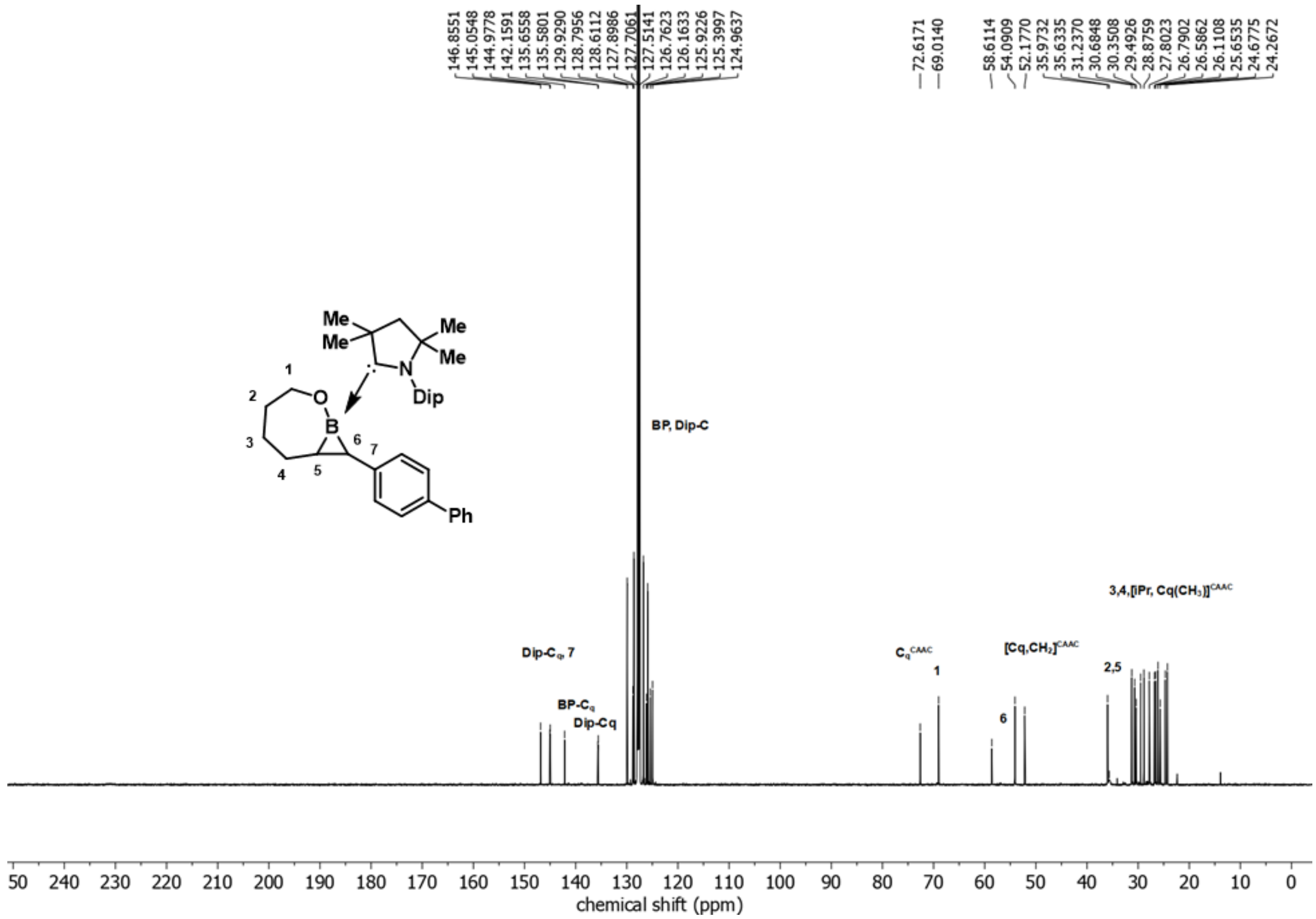

^{11}**B NMR (160 MHz, Benzene-*d*$_6$) of Compound 7g**

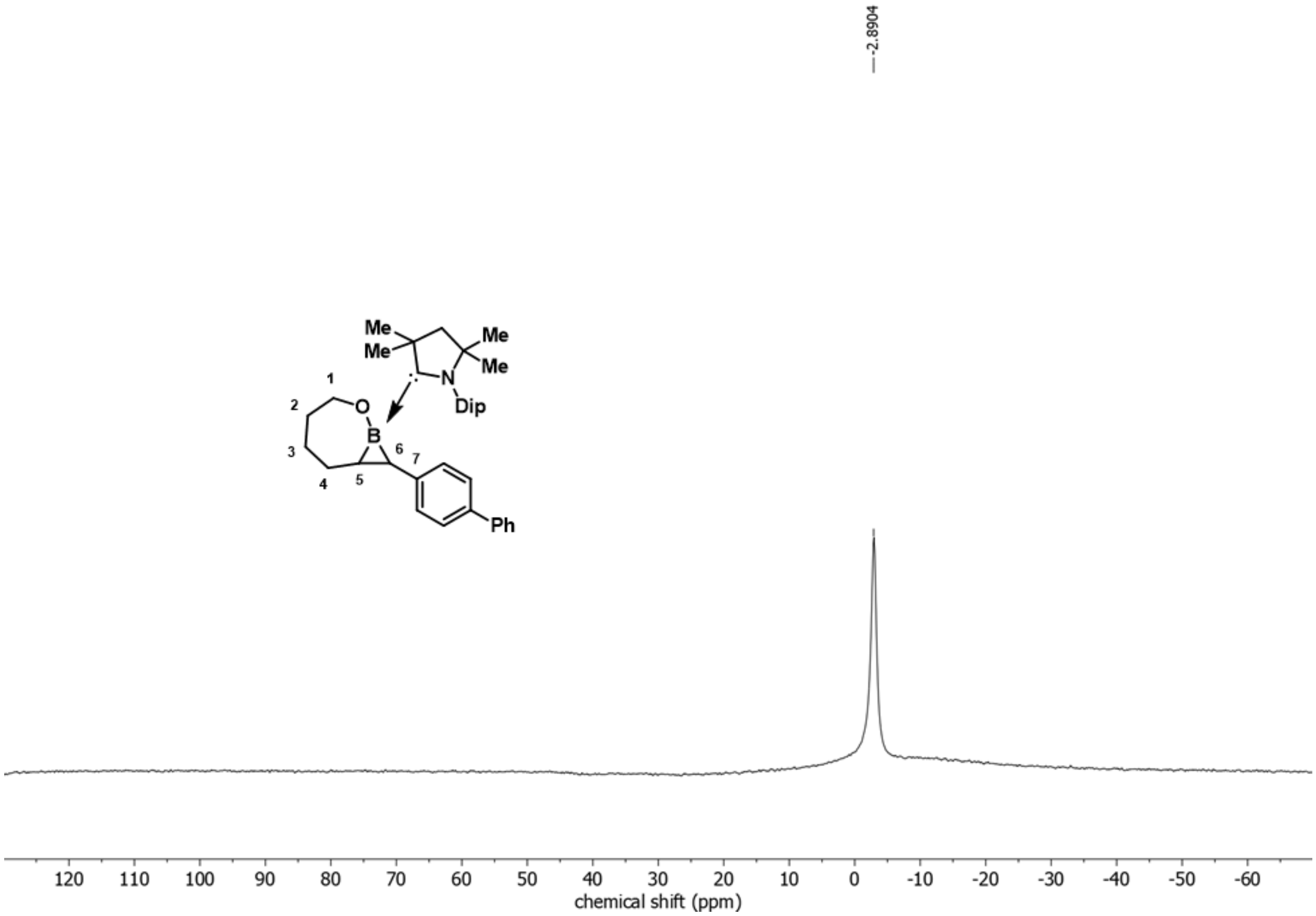

^{1}H -^{1}H COSY (500 MHz, Benzene-d_6) of Compound 7g

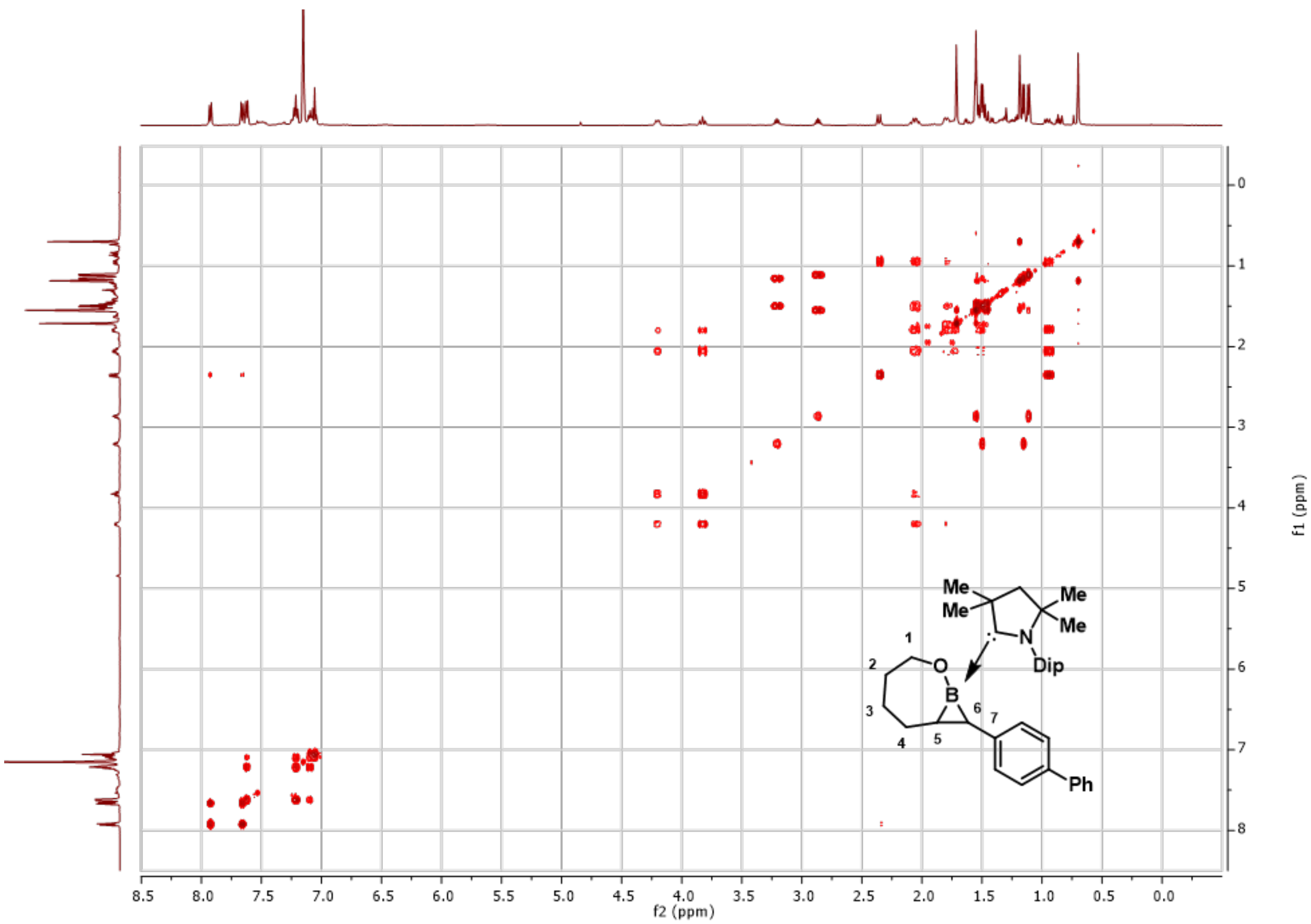

^{1}H -^{13}C HSQC (500 MHz, Benzene-d_6) of Compound 7g

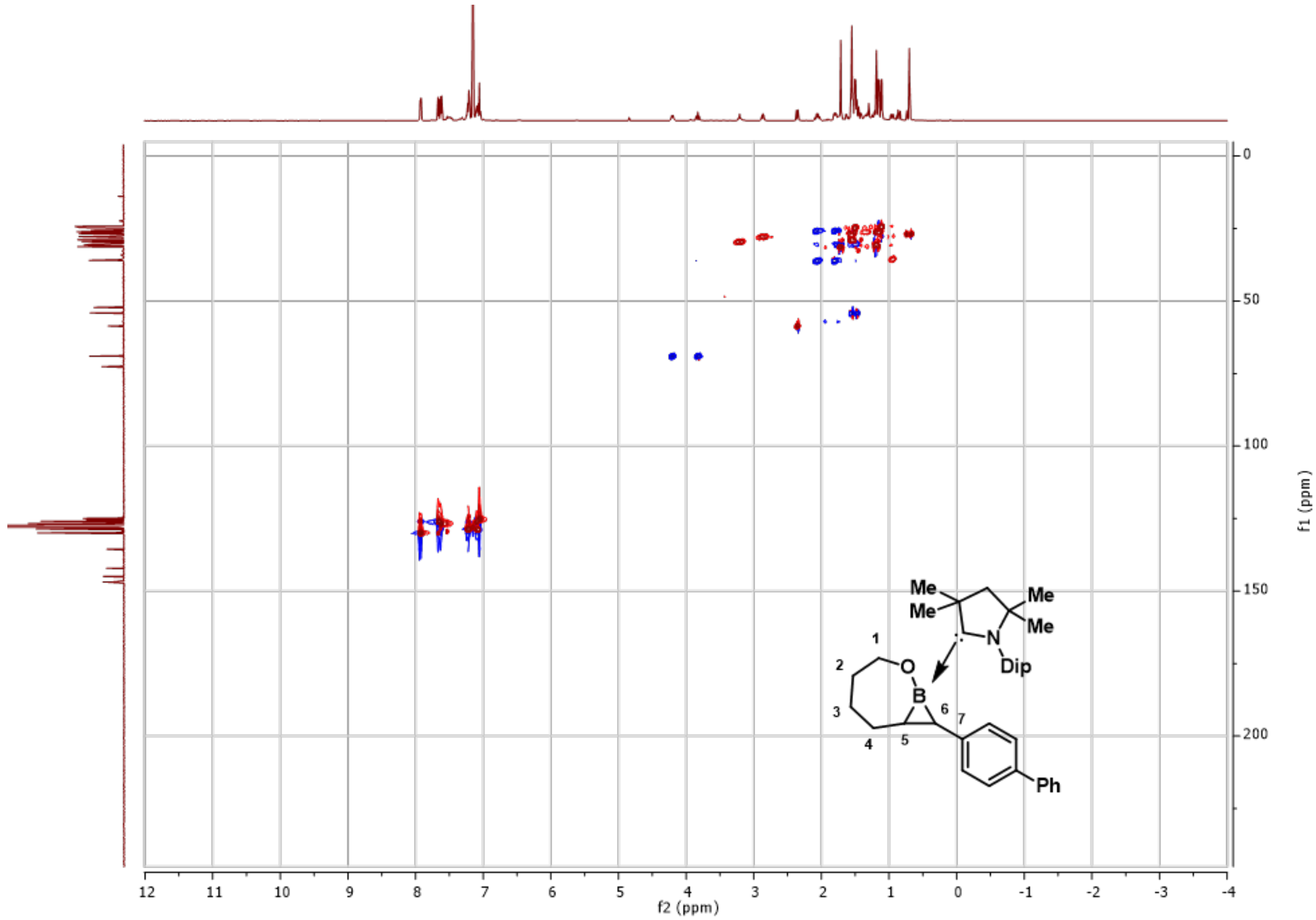

^{1}H -^{13}C HMBC (500 MHz, Benzene-d_6) of Compound 7g

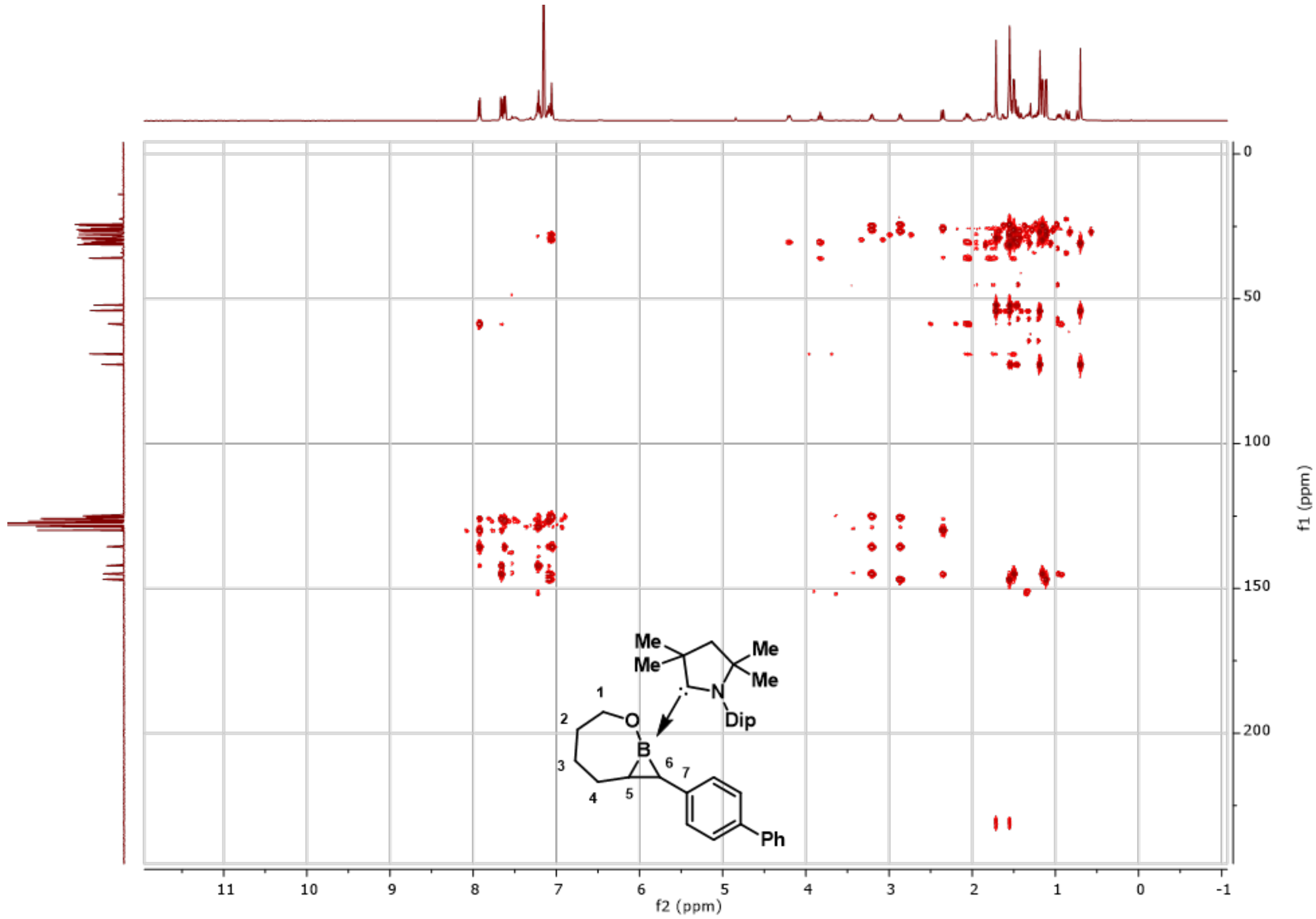

HRMS (ASAP) of Compound 7g

Elemental Composition Report Page 1

Single Mass Analysis
Tolerance = 50.0 PPM / DBE: min = -1.5, max = 500.0
Element prediction: Off
Number of isotope peaks used for i-FIT = 3

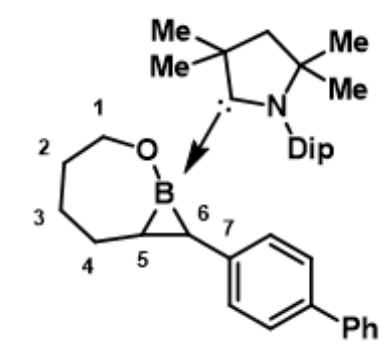

Chemical Formula: $C_{38}H_{50}BNO$
Exact Mass: 547.3985

Monoisotopic Mass, Even Electron Ions
1 formula(e) evaluated with 1 results within limits (up to 50 closest results for each mass)
Elements Used:
C: 38-38 H: 50-60 B: 0-2 N: 1-1 O: 1-1 Na: 0-1
DCM

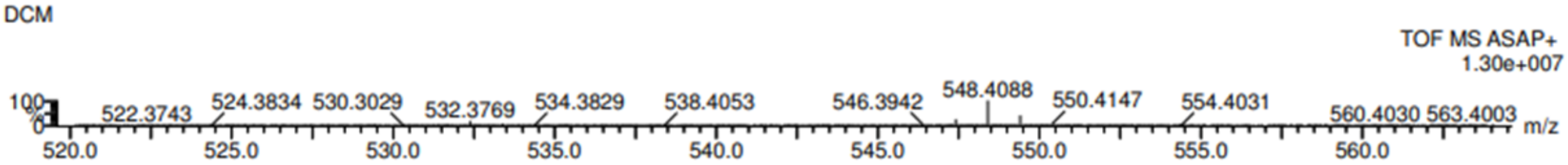

Minimum:				-1.5				
Maximum:		5.0	50.0	500.0				
Mass	Calc. Mass	mDa	PPM	DBE	i-FIT	Norm	Conf(%)	Formula
548.4088	548.4064	2.4	4.4	14.5	713.8	n/a	n/a	C38 H51 B N O

^{1}H NMR (500 MHz, Benzene-d_6) of Compound 7h

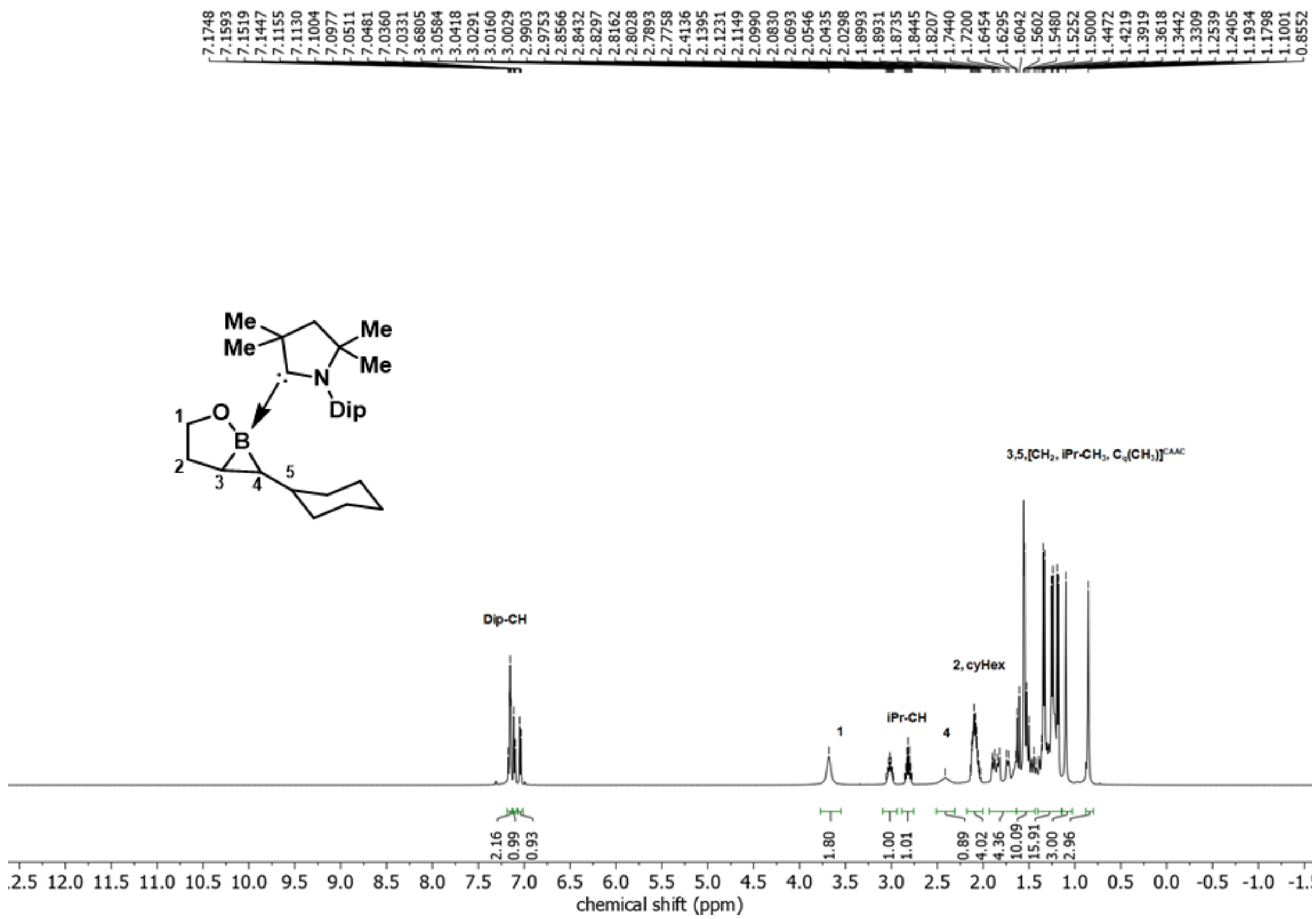

$^{13}C\{^{1}H\}$ NMR (126 MHz, Benzene-d_6) of Compound 7h

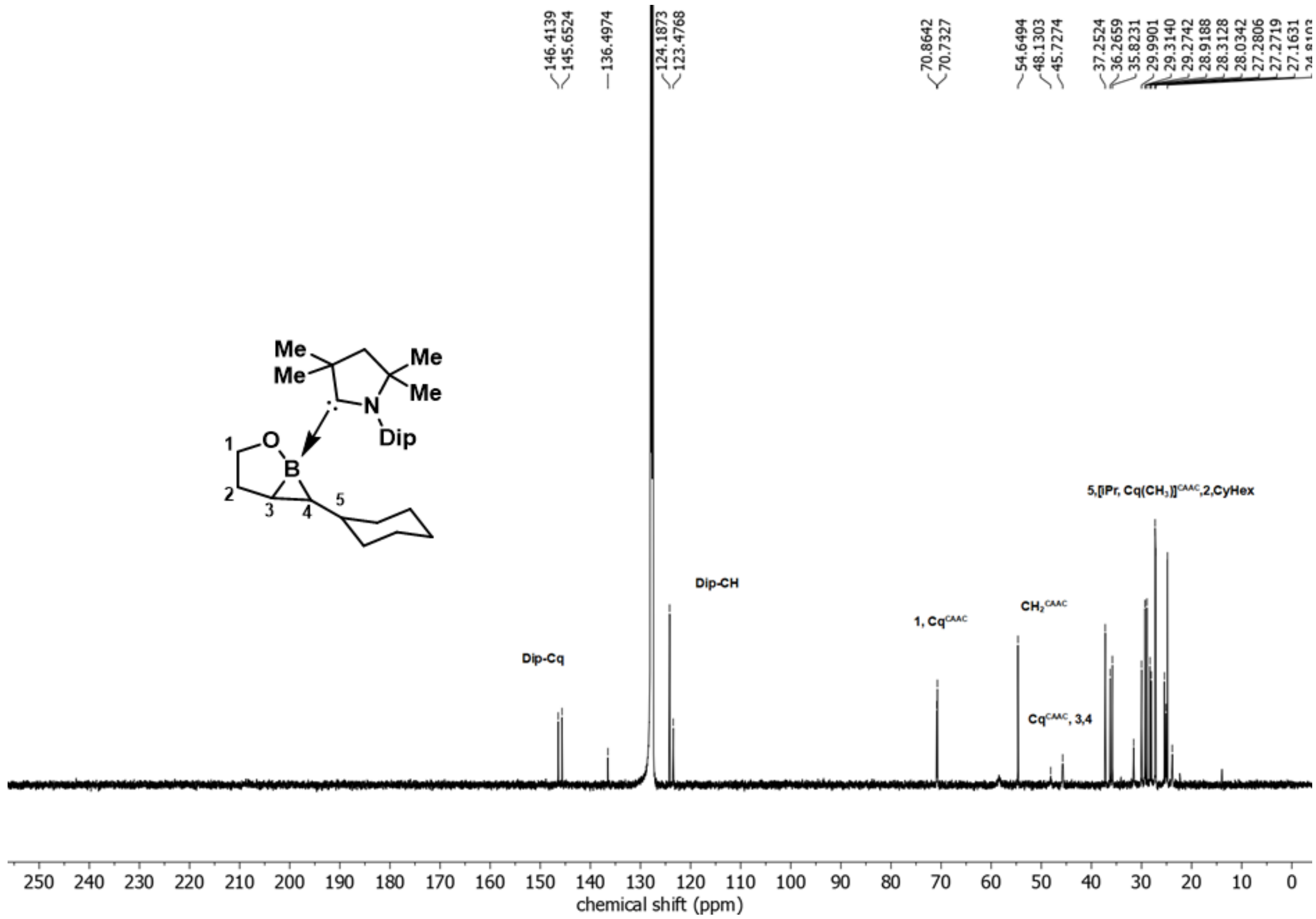

^{11}B NMR (160 MHz, Benzene-d_6) of Compound 7h

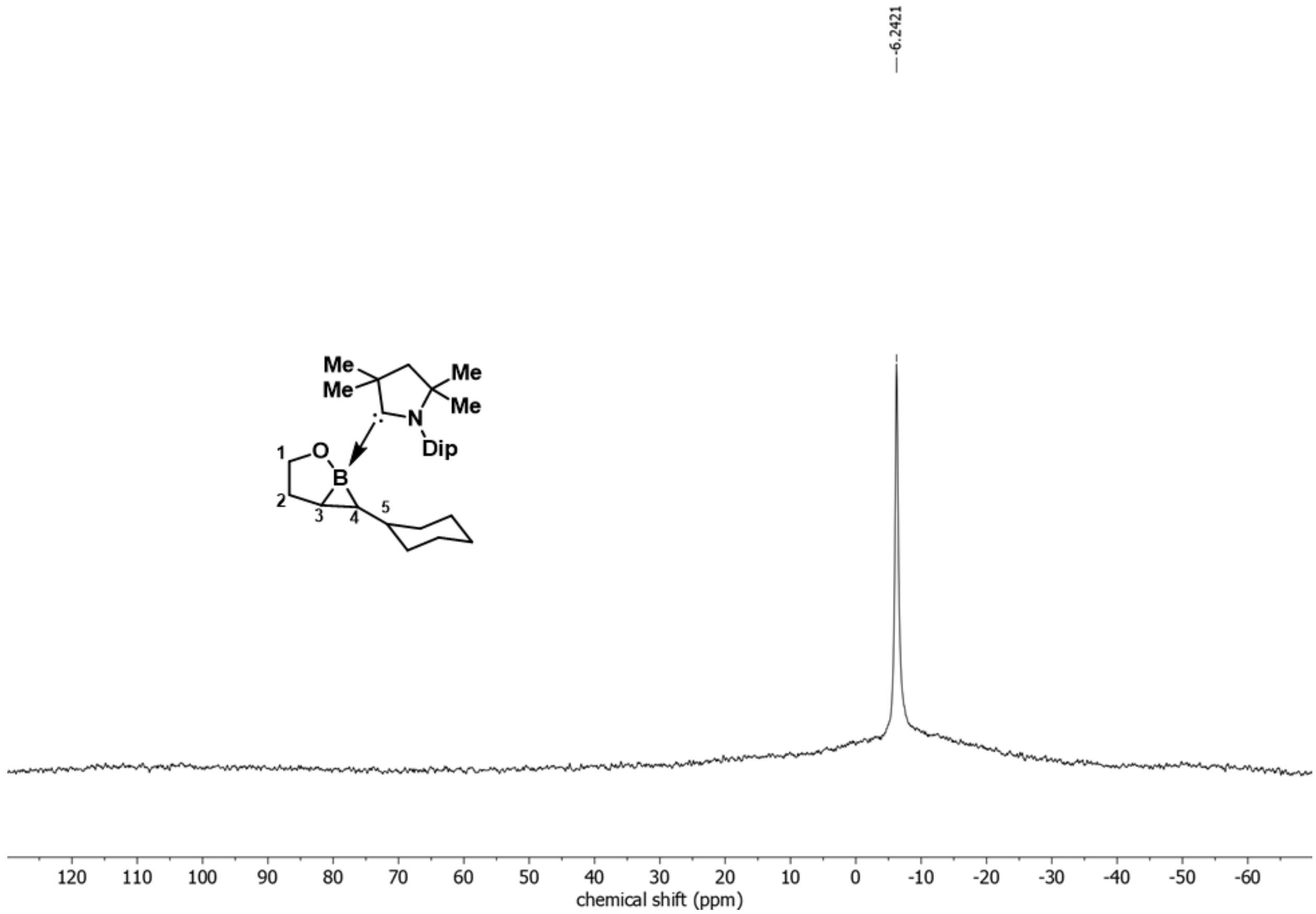

^1H -^1H COSY (500 MHz, Benzene-d_6) of Compound 7h

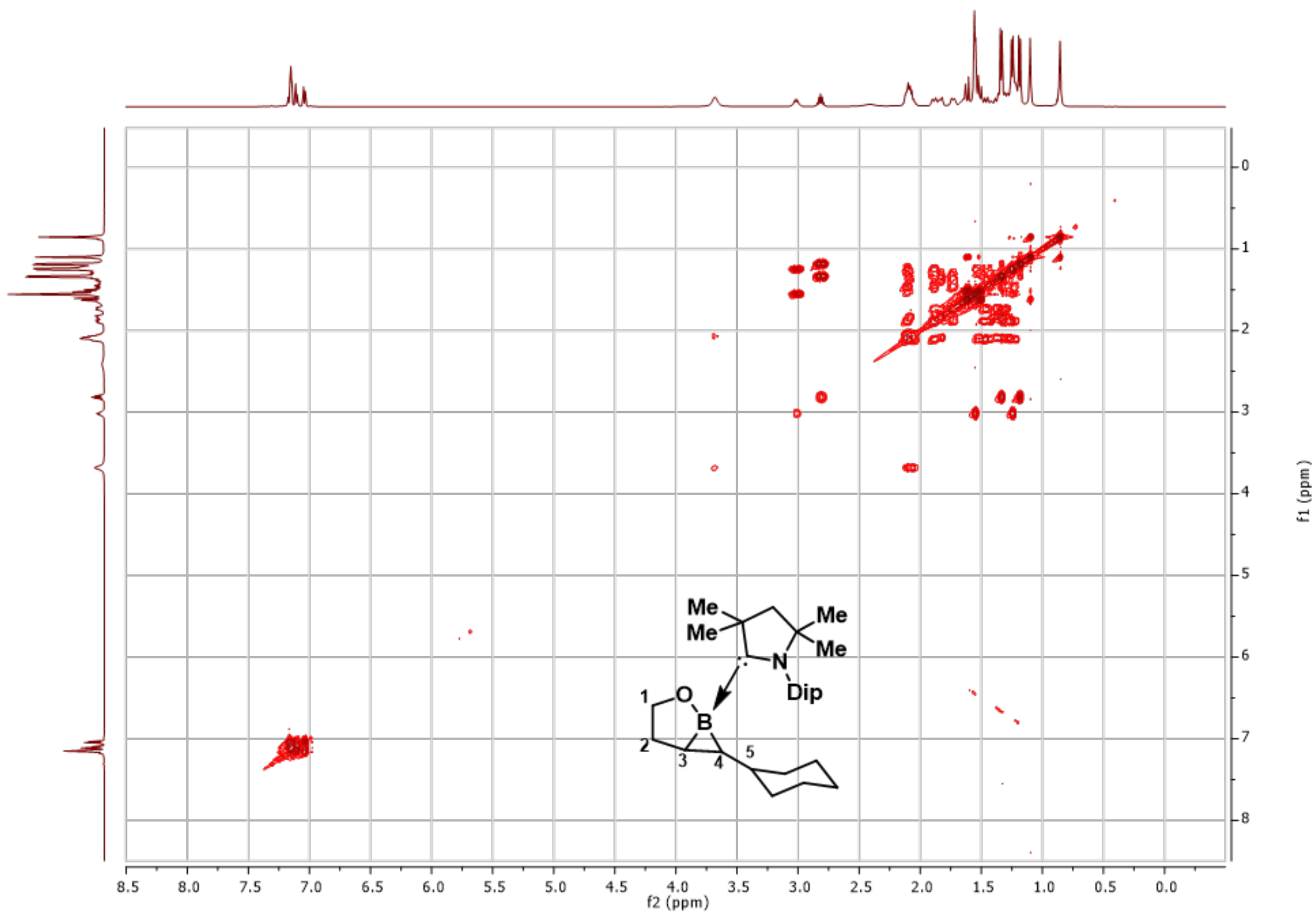

^{1}H - ^{13}C HSQC (500 MHz, Benzene-d_6) of Compound 7h

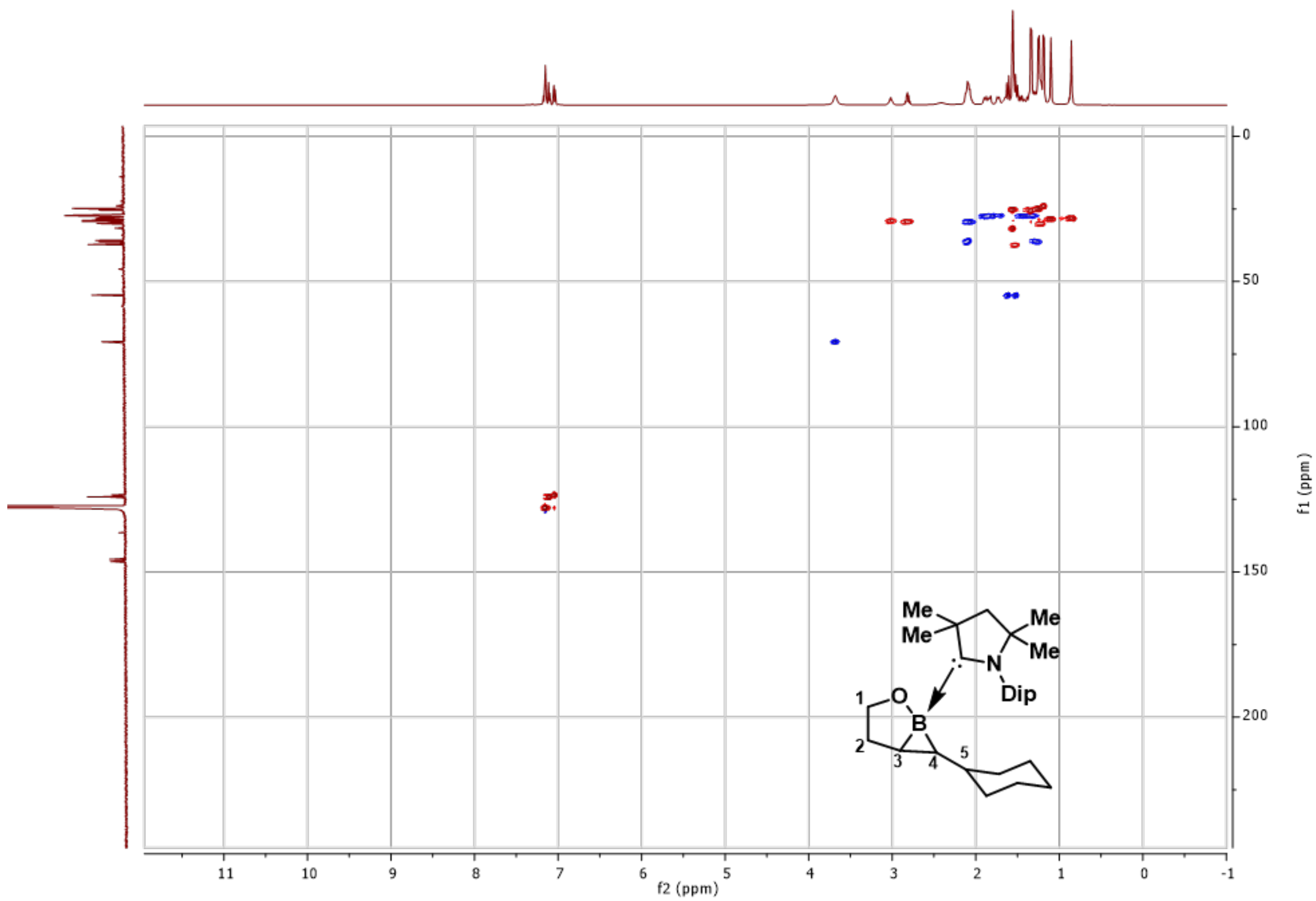

^{1}H -^{13}C HMBC (500 MHz, Benzene-d_6) of Compound 7h

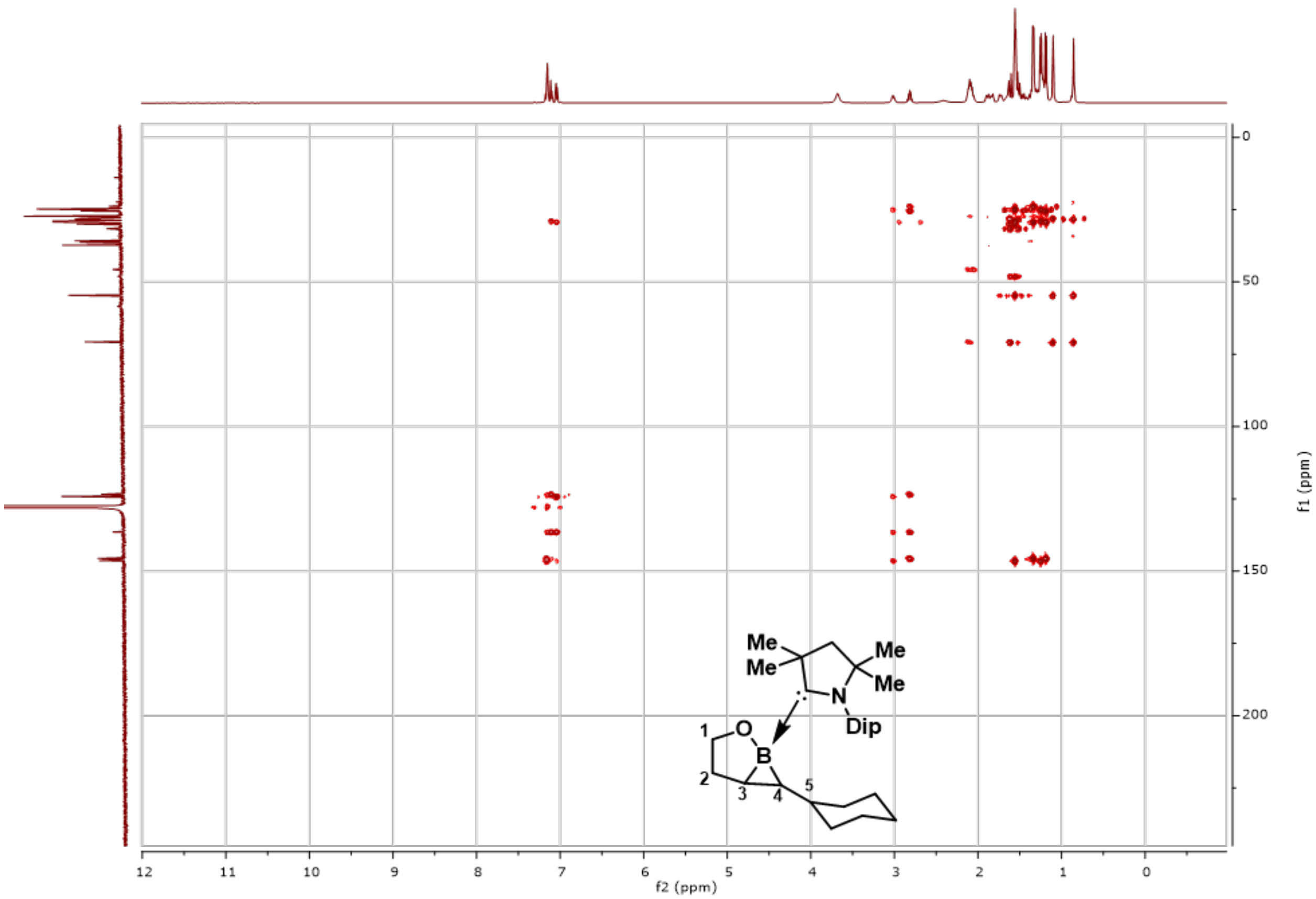

HRMS (ASAP) of Compound 7h

Elemental Composition Report Page 1

Single Mass Analysis
Tolerance = 50.0 PPM / DBE: min = -1.5, max = 500.0
Element prediction: Off
Number of isotope peaks used for i-FIT = 3

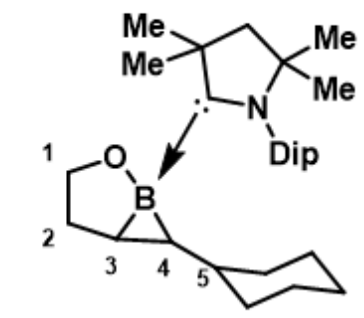

Chemical Formula: $C_{30}H_{48}BNO$
Exact Mass: 449.3829

Monoisotopic Mass, Even Electron Ions
1 formula(e) evaluated with 1 results within limits (up to 50 closest results for each mass)
Elements Used:
C: 30-30 H: 40-50 B: 0-2 N: 1-1 O: 1-1 Na: 0-1
DCM

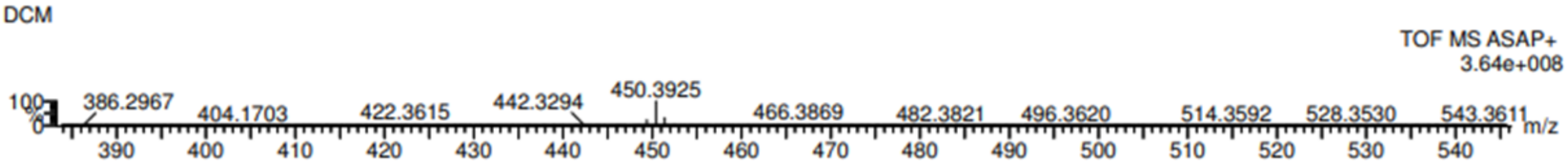

Minimum:				-1.5				
Maximum:		5.0	50.0	500.0				
Mass	Calc. Mass	mDa	PPM	DBE	i-FIT	Norm	Conf(%)	Formula
450.3925	450.3907	1.8	4.0	7.5	1196.6	n/a	n/a	C30 H49 B N O

^{1}H NMR (500 MHz, Chloroform-*d*) of Compound 8

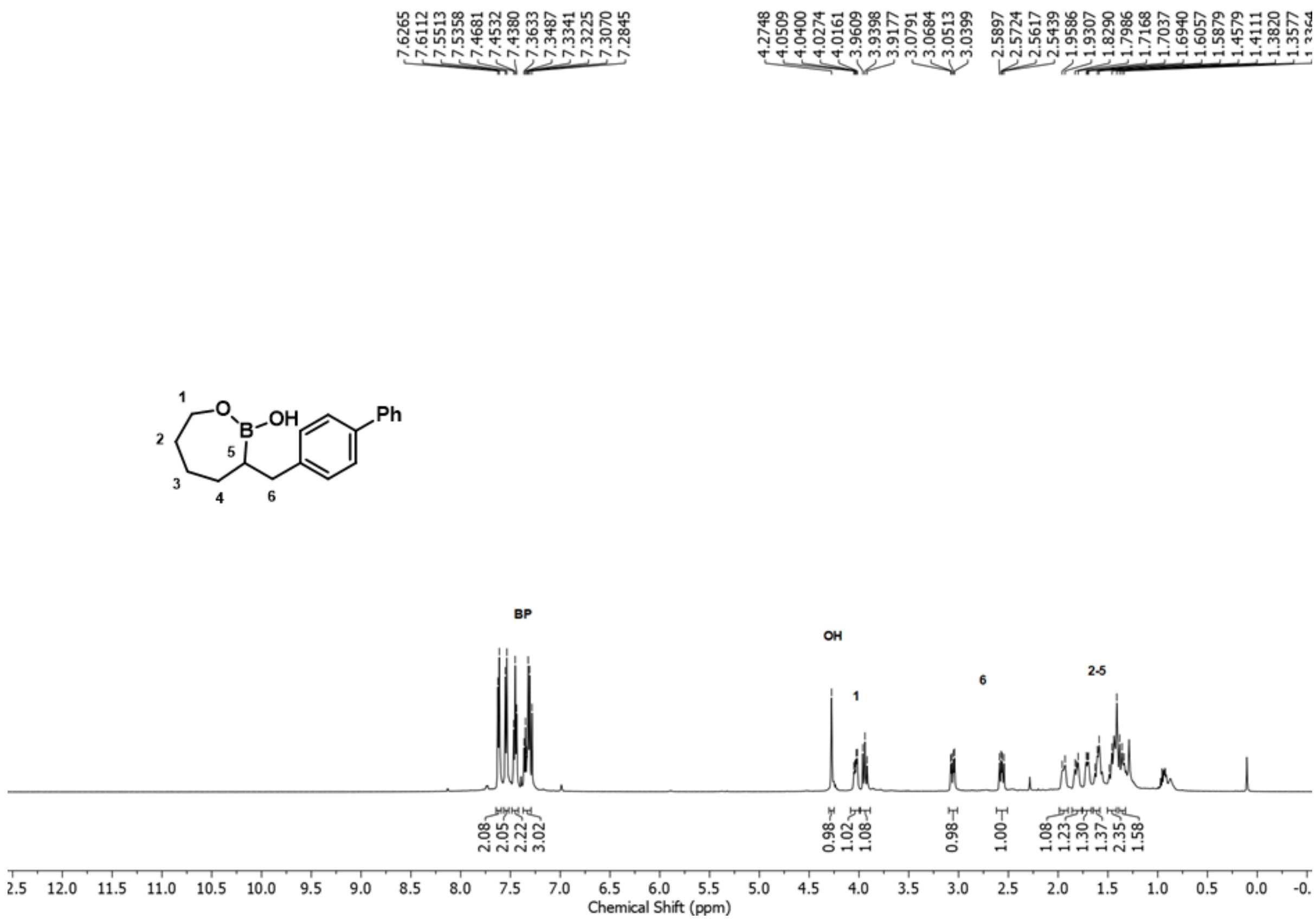

$^{13}C\{^{1}H\}$ NMR (126 MHz, Chloroform-*d*) of Compound 8

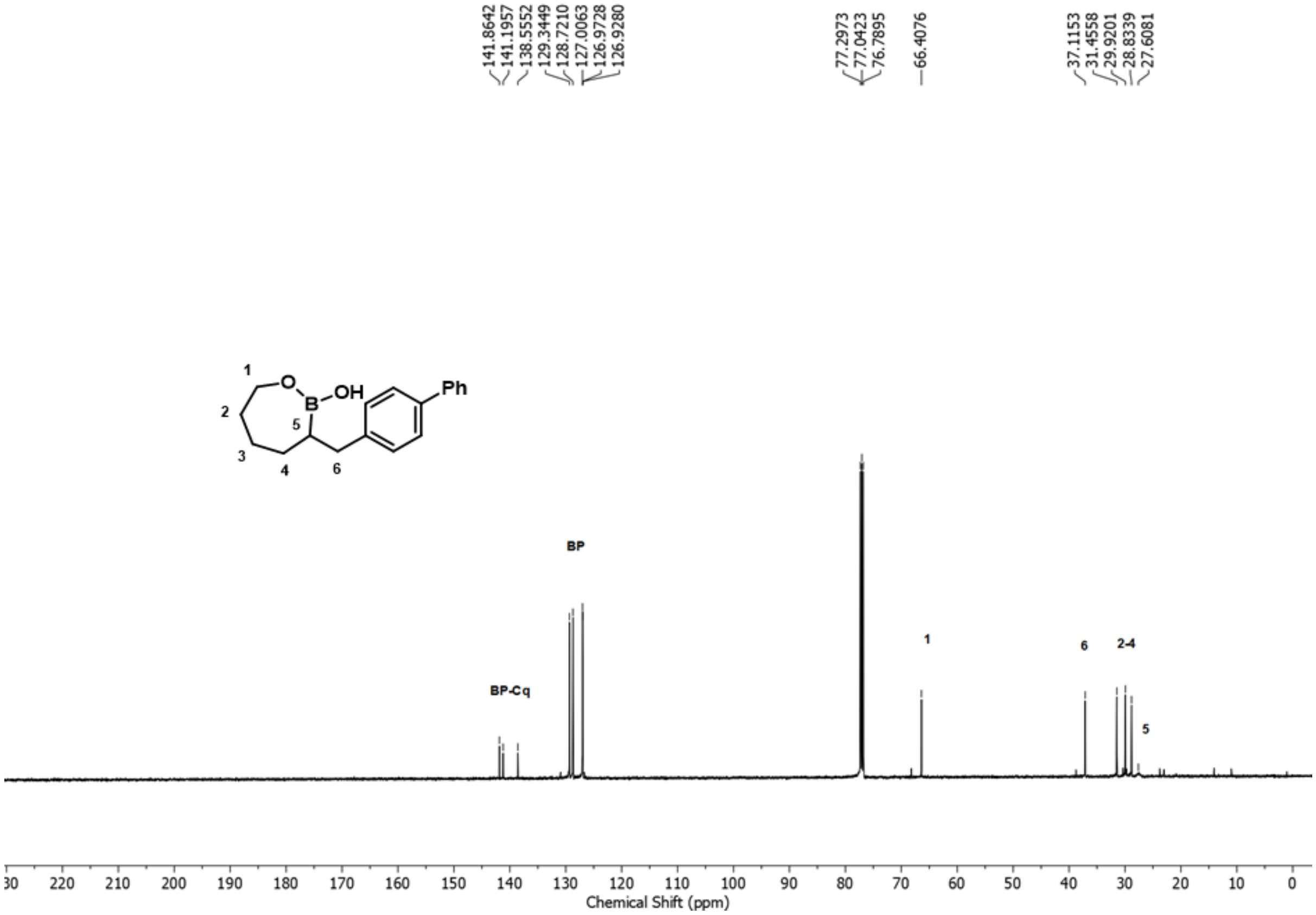

^{11}B NMR (160 MHz, Chloroform-*d*) of Compound 8

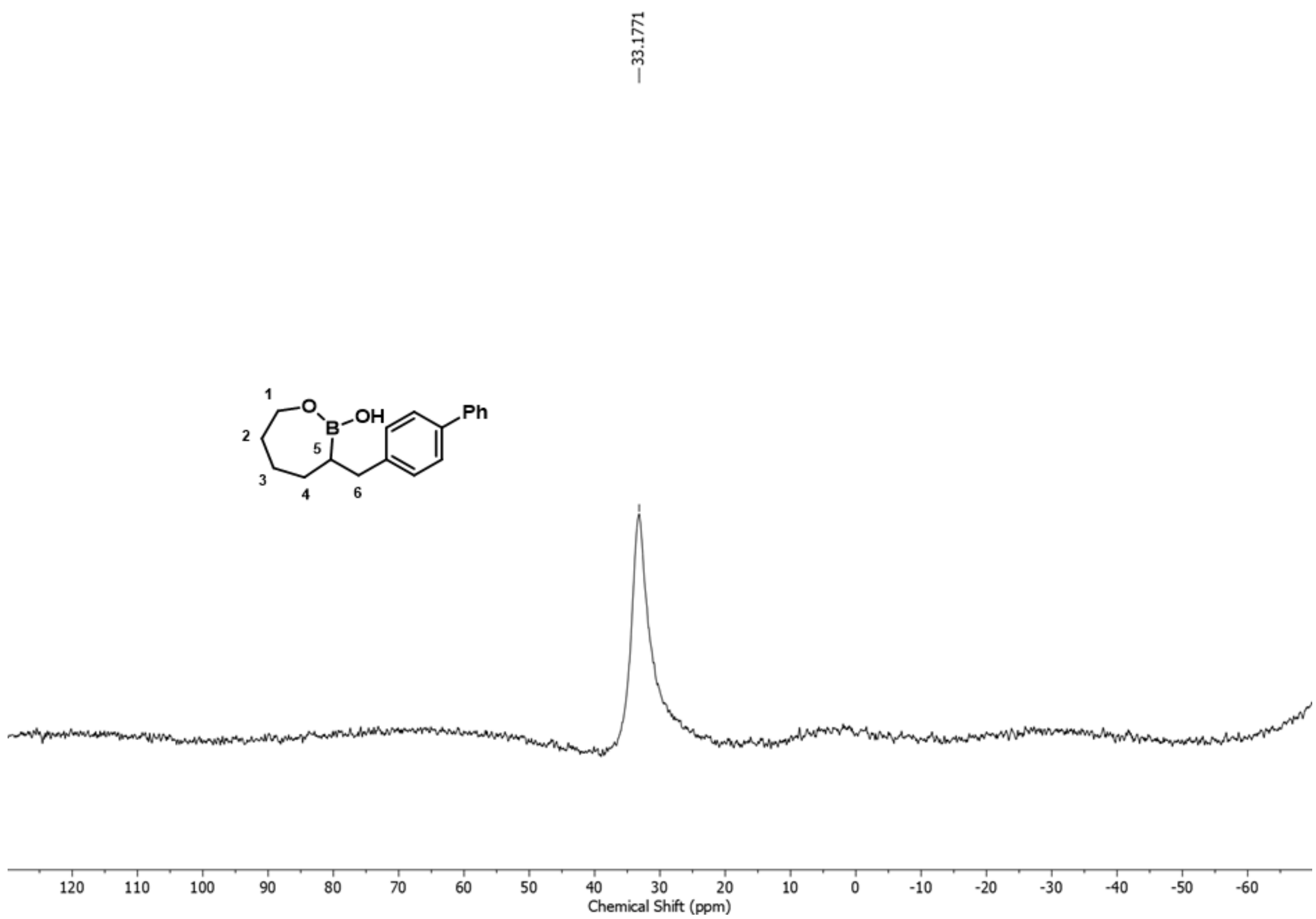

HRMS (ASAP) of Compound 8

Elemental Composition Report | **Page 1**

Single Mass Analysis
Tolerance = 5.0 PPM / DBE: min = -1.5, max = 500.0
Element prediction: Off
Number of isotope peaks used for i-FIT = 3

Monoisotopic Mass, Odd and Even Electron Ions
4 formula(e) evaluated with 1 results within limits (up to 50 closest results for each mass)
Elements Used:
C: 18-18 H: 20-30 B: 0-2 O: 1-2 Na: 0-1

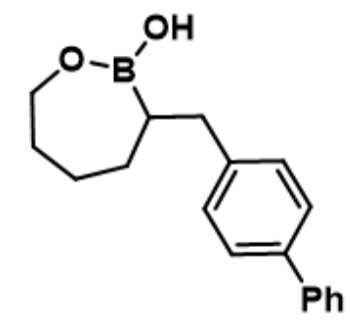

Chemical Formula: $C_{18}H_{21}BO_2$
Exact Mass: 280.1635

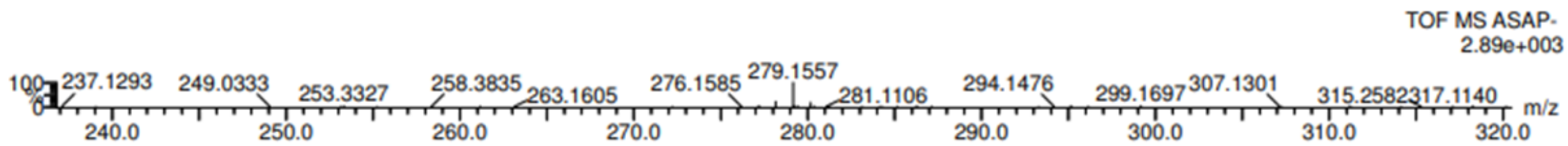

Minimum:				-1.5				
Maximum:		5.0	5.0	500.0				
Mass	Calc. Mass	mDa	PPM	DBE	i-FIT	Norm	Conf(%)	Formula
279.1557	279.1556	0.1	0.4	9.5	70.9	n/a	n/a	C18 H20 B O2

^{1}H NMR (500 MHz, Benzene-d_6) of Compound 9

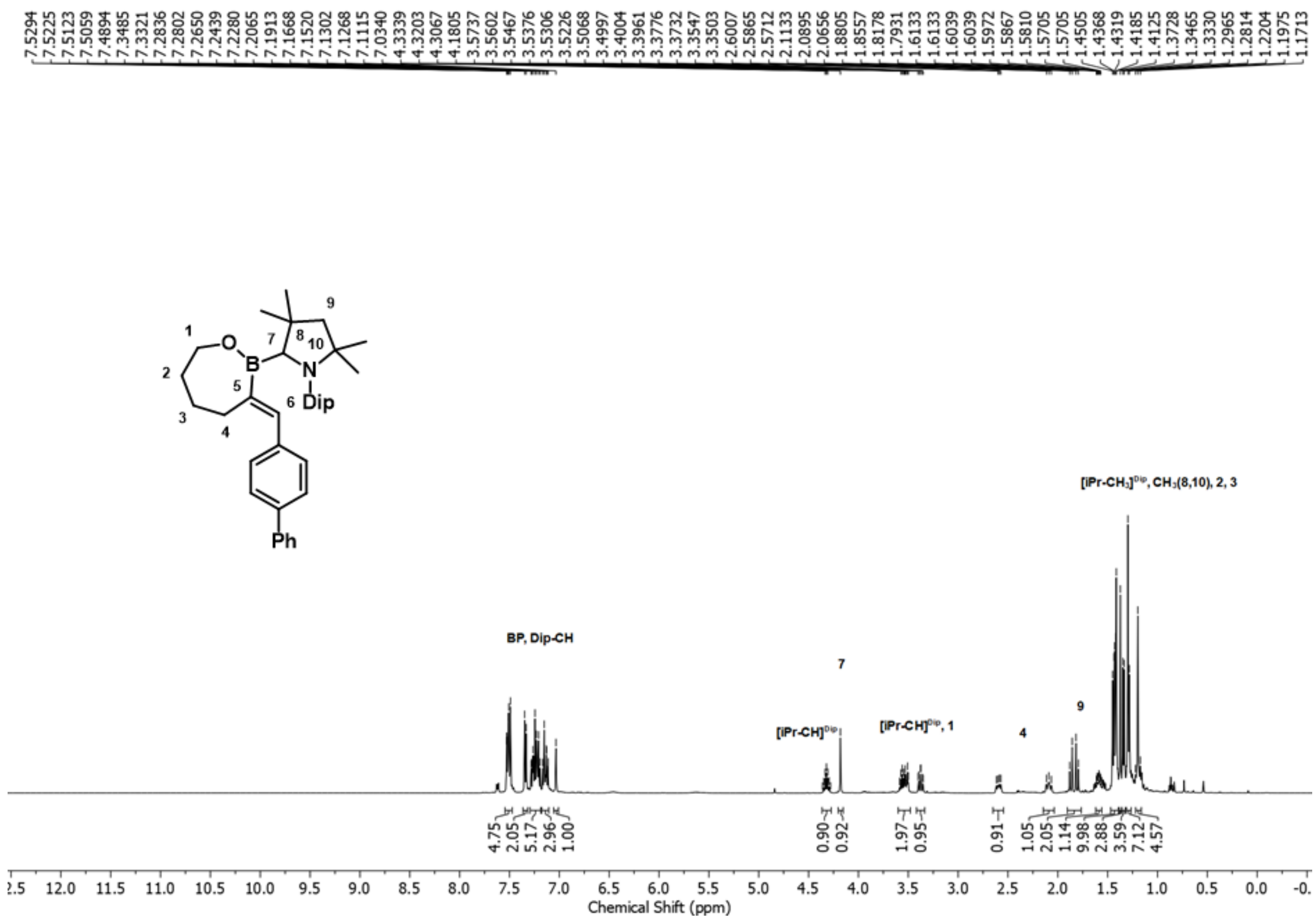

$^{13}C\{^{1}H\}$ NMR (126 MHz, Benzene-d_6) of Compound 9

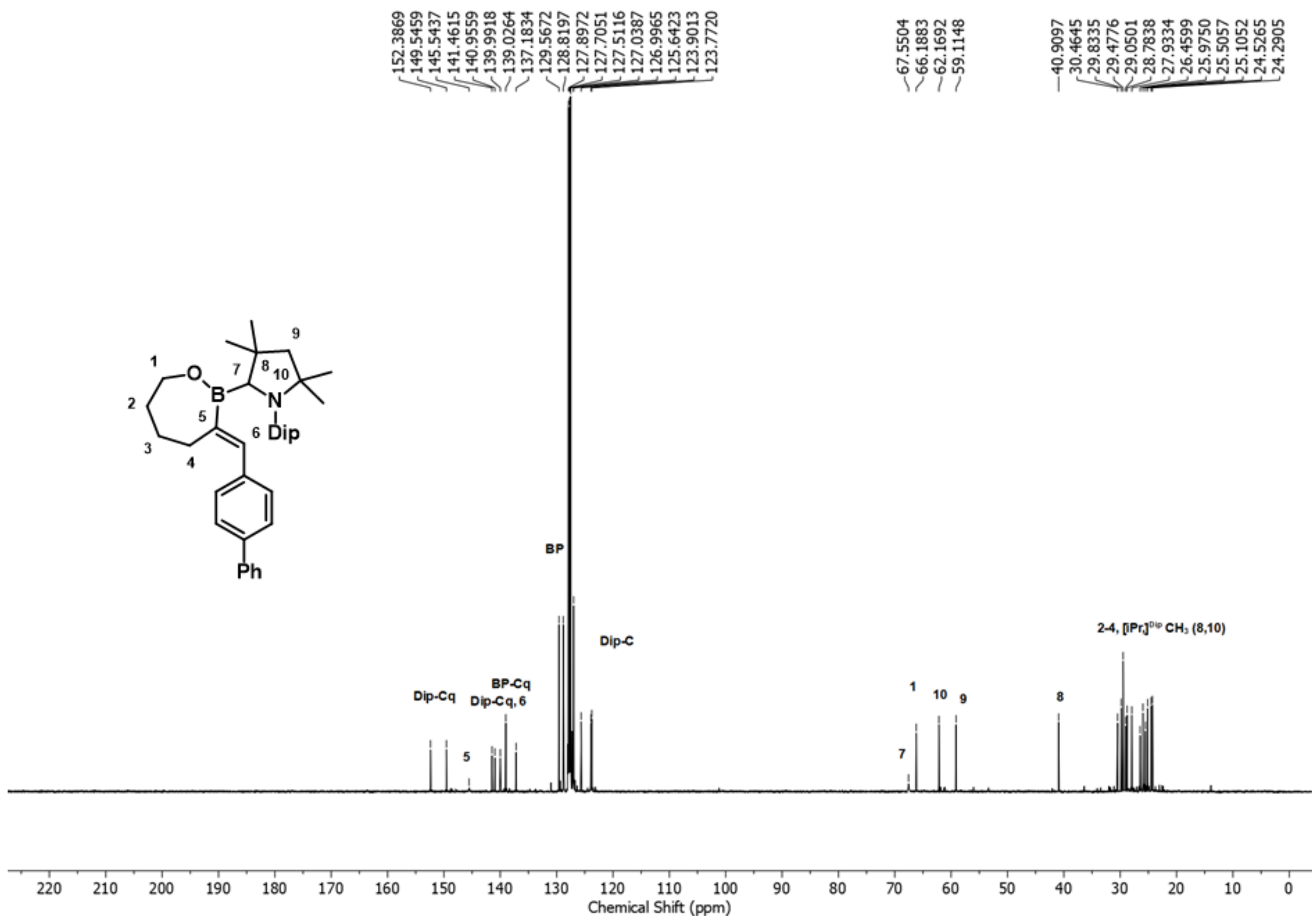

^{11}B NMR (160 MHz, Benzene-d_6) of Compound 9

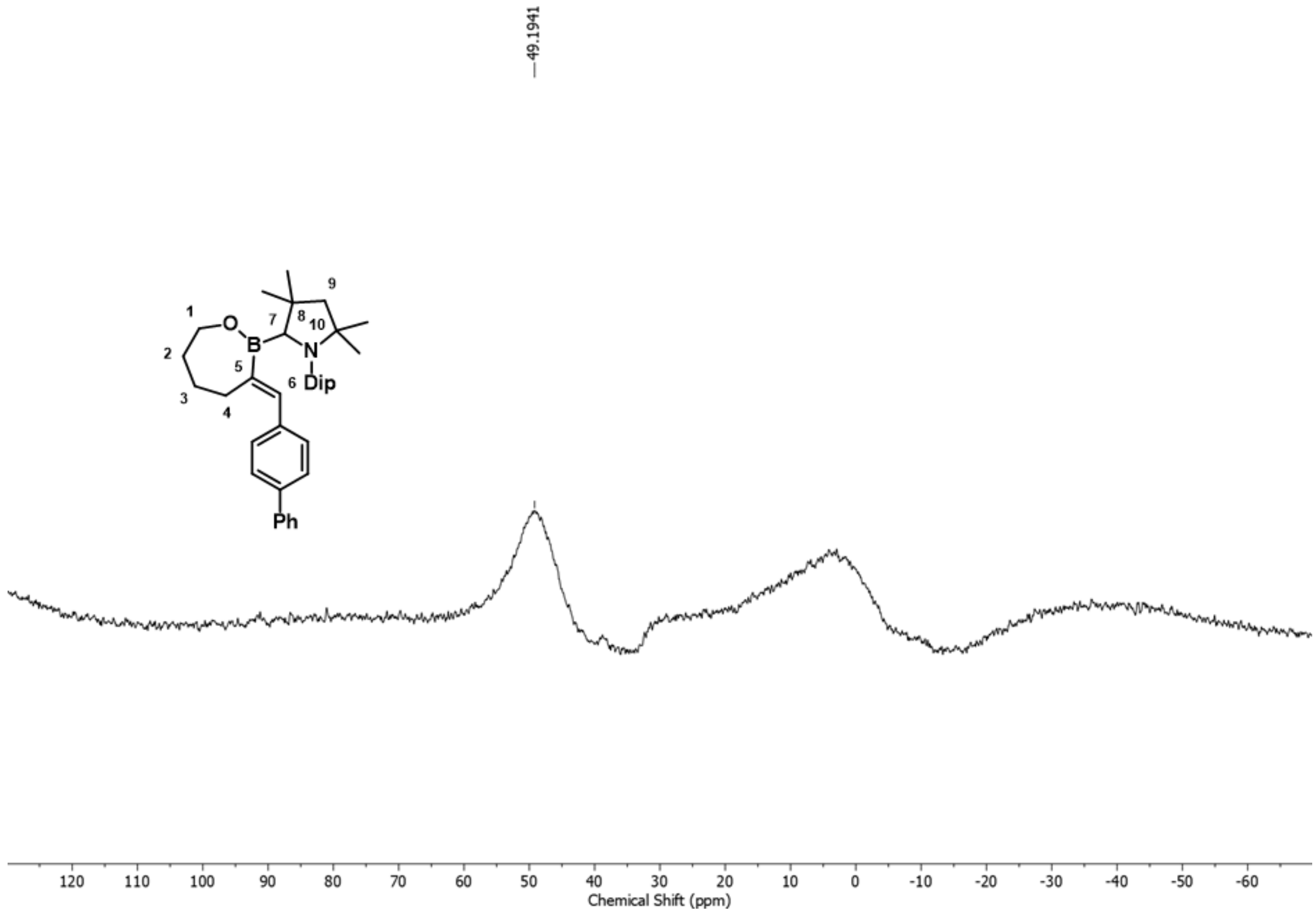

HRMS (ASAP) of Compound 9

Elemental Composition Report **Page 1**

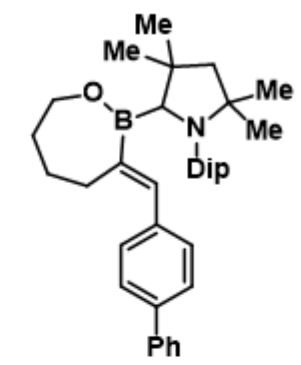

Chemical Formula: $C_{38}H_{50}BNO$
Exact Mass: 547.3985

Single Mass Analysis
Tolerance = 20.0 PPM / DBE: min = -1.5, max = 500.0
Element prediction: Off
Number of isotope peaks used for i-FIT = 3

Monoisotopic Mass, Even Electron Ions
1 formula(e) evaluated with 1 results within limits (up to 50 closest results for each mass)
Elements Used:
C: 38-38 H: 50-55 B: 0-2 N: 1-1 O: 1-2 Na: 0-1

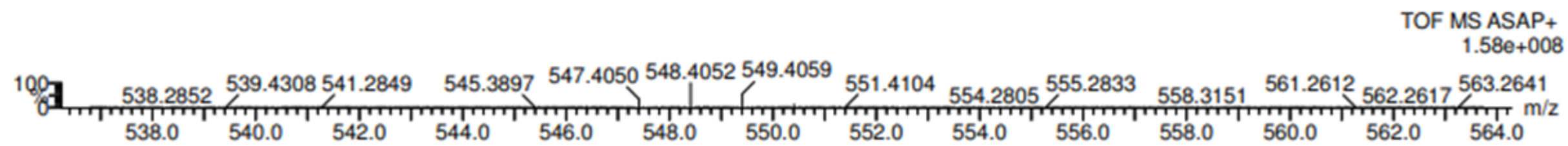

Minimum:				-1.5				
Maximum:		5.0	20.0	500.0				
Mass	Calc. Mass	mDa	PPM	DBE	i-FIT	Norm	Conf(%)	Formula
548.4052	548.4064	-1.2	-2.2	14.5	818.1	n/a	n/a	C38 H51 B N O

^{1}H NMR (500 MHz, Chloroform-*d*) of Compound 10

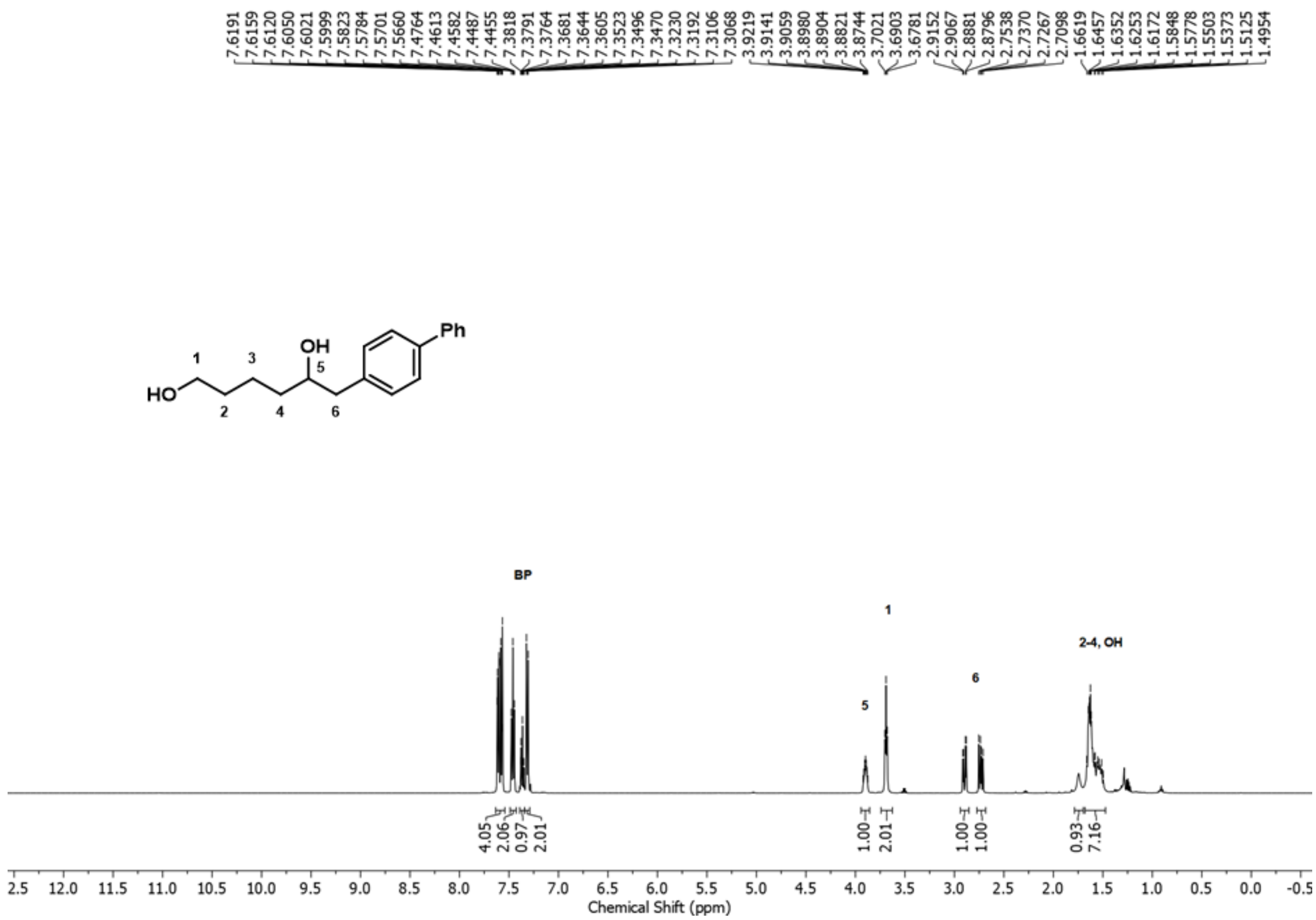

$^{13}C\{^1H\}$ NMR (126 MHz, Chloroform-*d*) of Compound 10

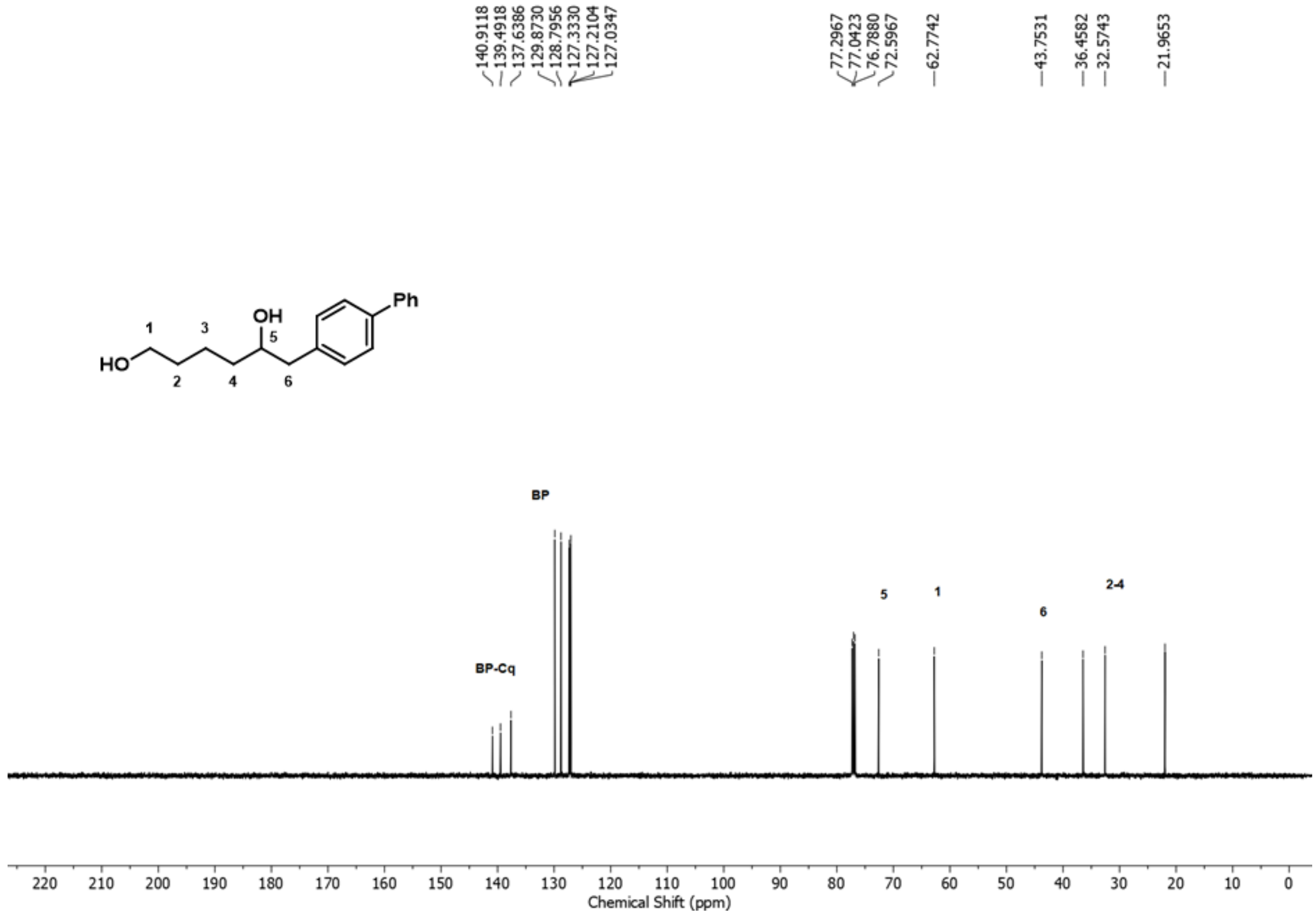

HRMS (ASAP) of Compound 10

Elemental Composition Report **Page 1**

Single Mass Analysis
Tolerance = 5.0 PPM / DBE: min = -1.5, max = 500.0
Element prediction: Off
Number of isotope peaks used for i-FIT = 3

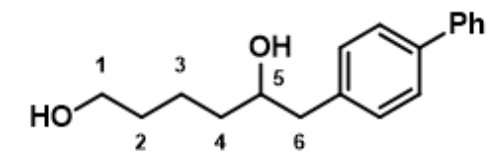

Chemical Formula: $C_{18}H_{22}O_2$
Exact Mass: 270.1620

Monoisotopic Mass, Even Electron Ions
7 formula(e) evaluated with 1 results within limits (up to 50 closest results for each mass)
Elements Used:
C: 11-18 H: 10-23 O: 2-5 Na: 0-1

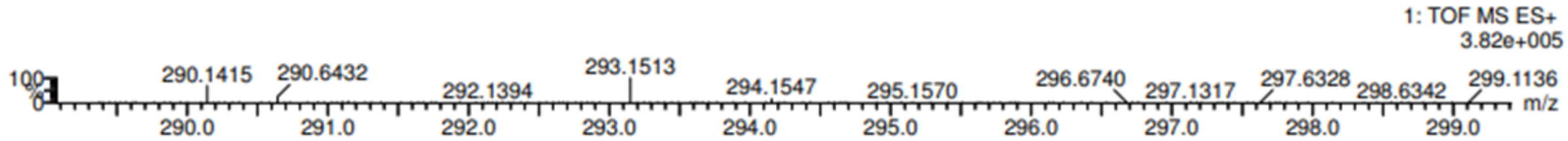

Minimum:				-1.5				
Maximum:		5.0	5.0	500.0				
Mass	Calc. Mass	mDa	PPM	DBE	i-FIT	Norm	Conf(%)	Formula
293.1513	293.1517	-0.4	-1.4	7.5	266.2	n/a	n/a	C18 H22 O2 Na

^{1}H NMR (500 MHz, Chloroform-*d*) of Compound 11

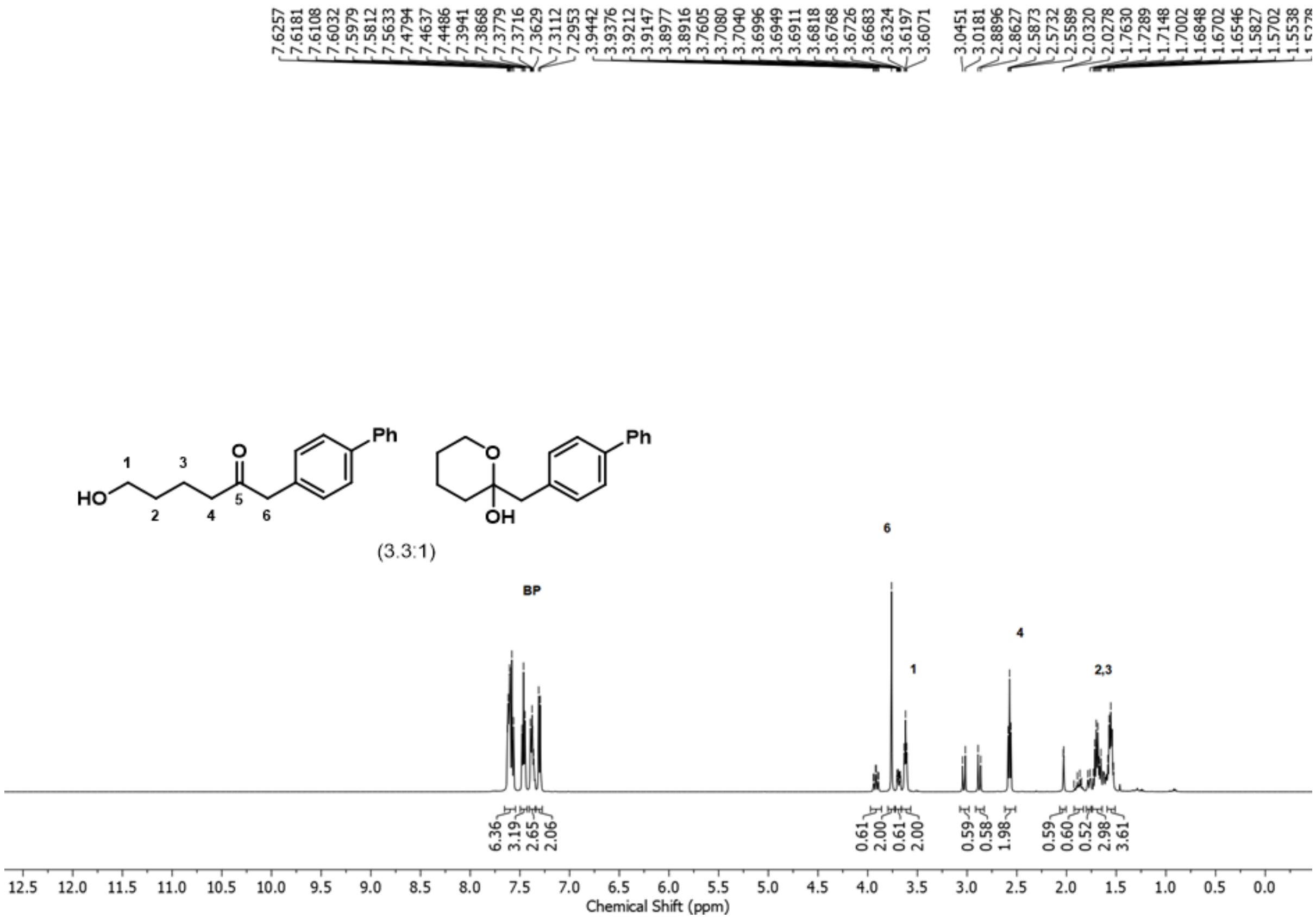

$^{13}C\{^{1}H\}$ NMR (126 MHz, Chloroform-*d*) of Compound 11

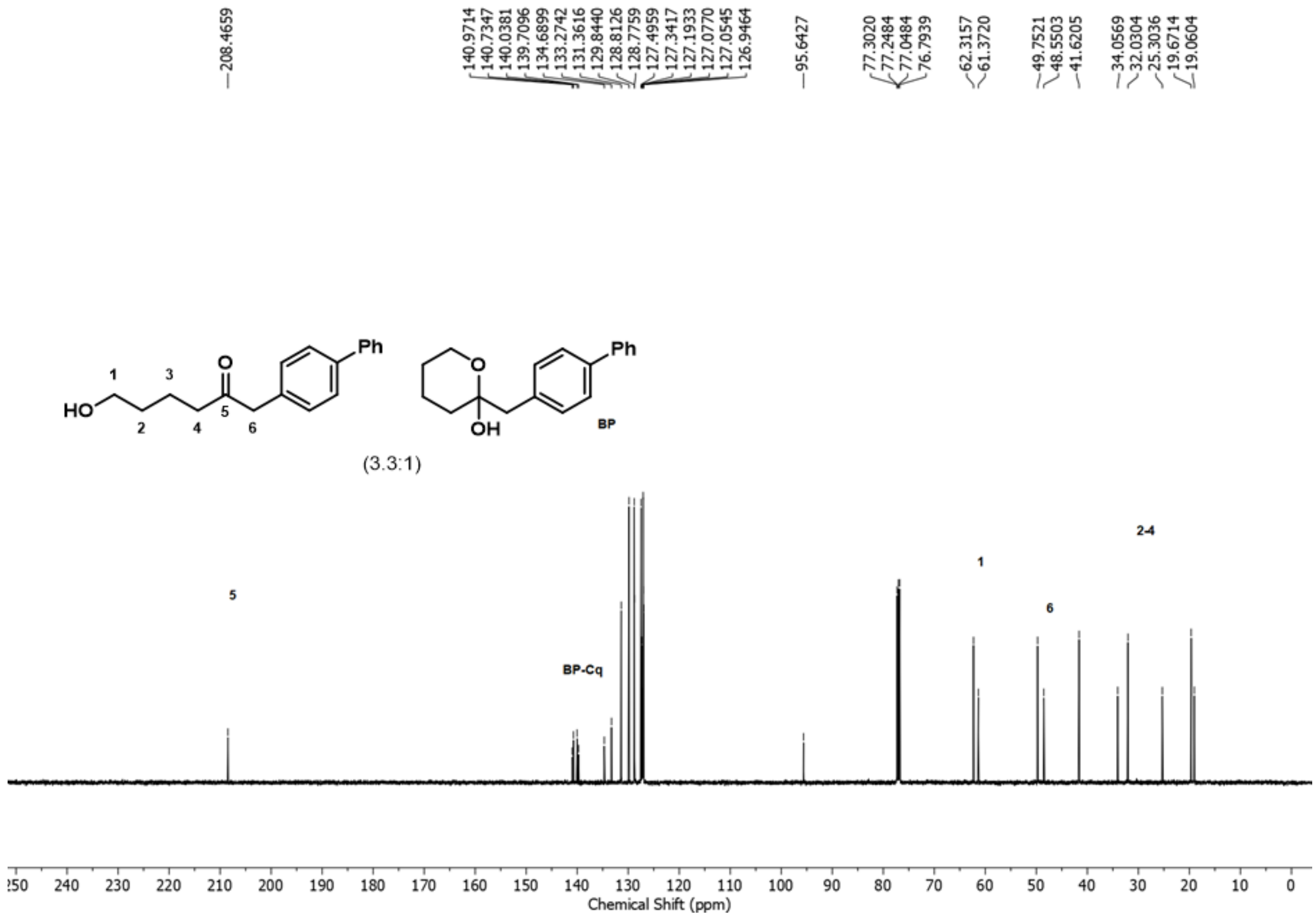

HRMS (ASAP) of Compound 11

Elemental Composition Report **Page 1**

Single Mass Analysis
Tolerance = 5.0 PPM / DBE: min = -1.5, max = 500.0
Element prediction: Off
Number of isotope peaks used for i-FIT = 3

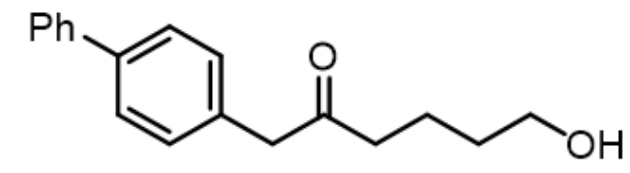

Chemical Formula: $C_{18}H_{20}O_2$
Exact Mass: 268.1463

Monoisotopic Mass, Even Electron Ions
6 formula(e) evaluated with 1 results within limits (up to 50 closest results for each mass)
Elements Used:
C: 18-18 H: 10-30 O: 1-5 Na: 0-1

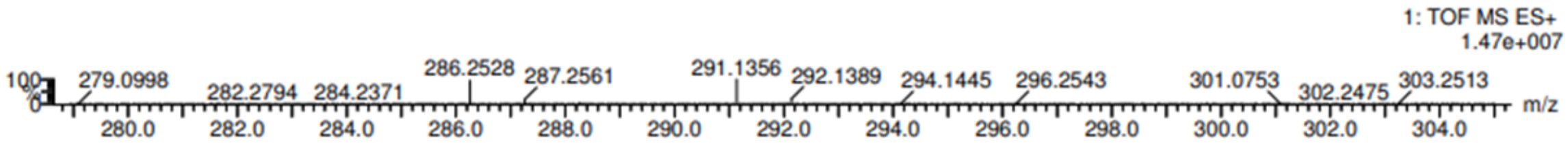

	Calc. Mass	mDa	PPM	DBE	i-FIT	Norm	Conf(%)	Formula
Minimum:				-1.5				
Maximum:		5.0	5.0	500.0				

Mass	Calc. Mass	mDa	PPM	DBE	i-FIT	Norm	Conf(%)	Formula
291.1356	291.1361	-0.5	-1.7	8.5	626.2	n/a	n/a	C18 H20 O2 Na

^{1}H NMR (300 MHz, Chloroform-*d*) of Compound 12

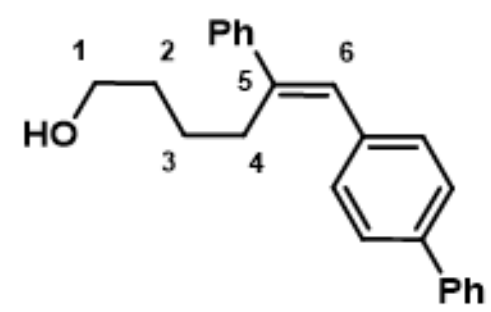

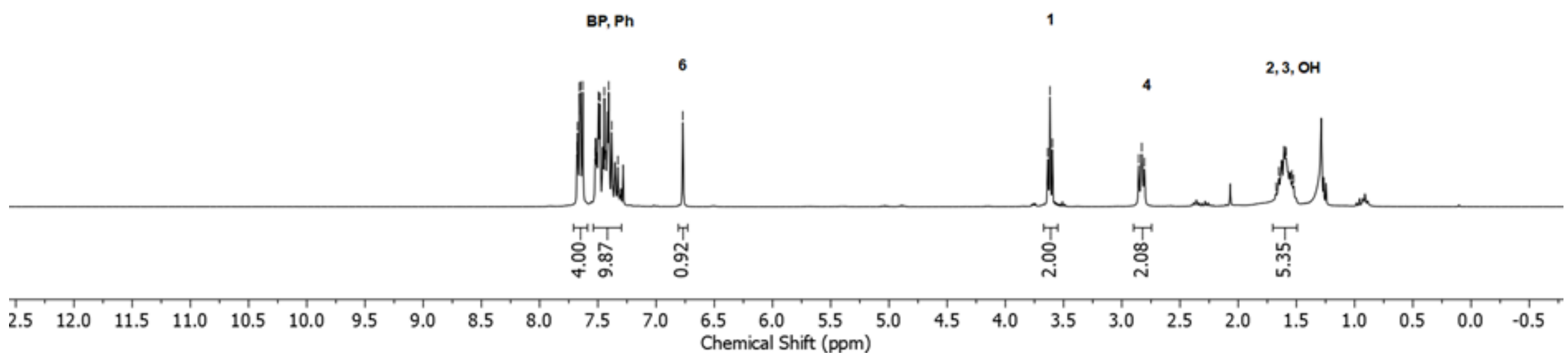

^{13}C NMR (101 MHz, Chloroform-*d*) of Compound 12

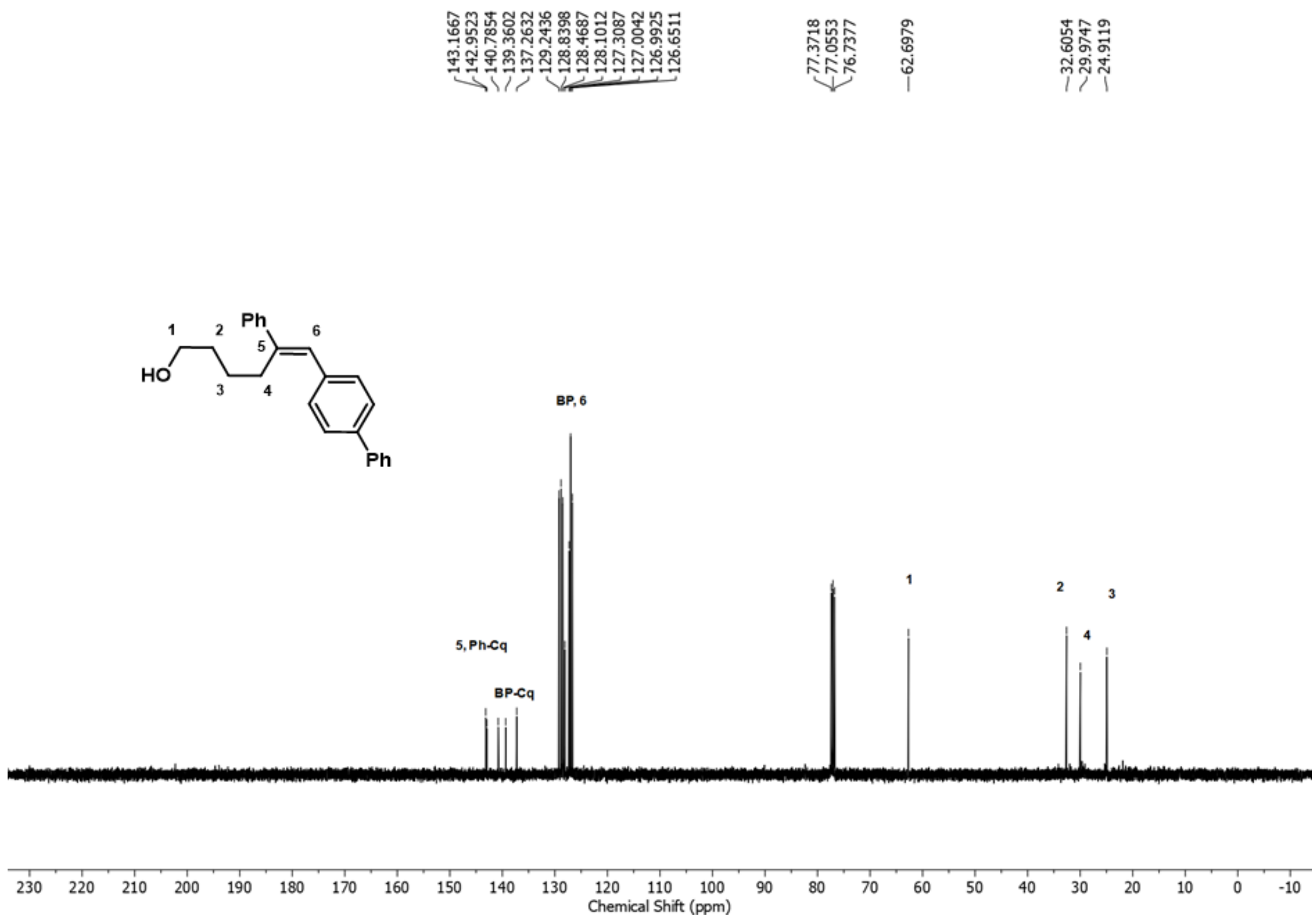

HRMS (ASAP) of Compound 12

Elemental Composition Report **Page 1**

Single Mass Analysis
Tolerance = 5.0 PPM / DBE: min = -1.5, max = 500.0
Element prediction: Off
Number of isotope peaks used for i-FIT = 3

Monoisotopic Mass, Odd and Even Electron Ions
14 formula(e) evaluated with 1 results within limits (up to 50 closest results for each mass)
Elements Used:
C: 20-24 H: 10-30 O: 1-3 Na: 0-1 Sn: 0-1

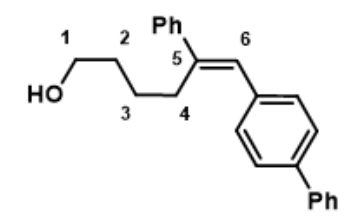

Chemical Formula: $C_{24}H_{24}O$
Exact Mass: 328.1827

DCM
BD-583 Dmitrii 2 223 (3.789) Cm (219:228)

TOF MS ASAP+
8.51e+006

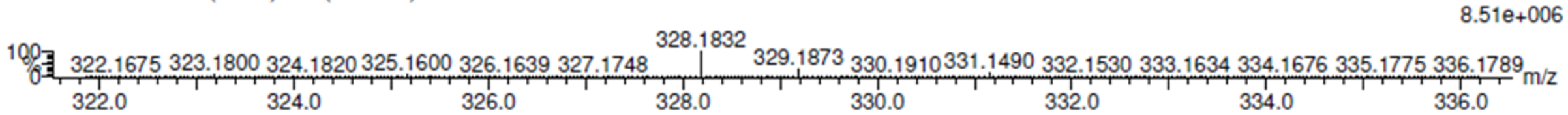

Minimum:				-1.5				
Maximum:		5.0	5.0	500.0				
Mass	Calc. Mass	mDa	PPM	DBE	i-FIT	Norm	Conf(%)	Formula
328.1832	328.1827	0.5	1.5	13.0	791.1	n/a	n/a	C24 H24 O

^{1}H NMR (500 MHz, Benzene-d_6) of Compound 14

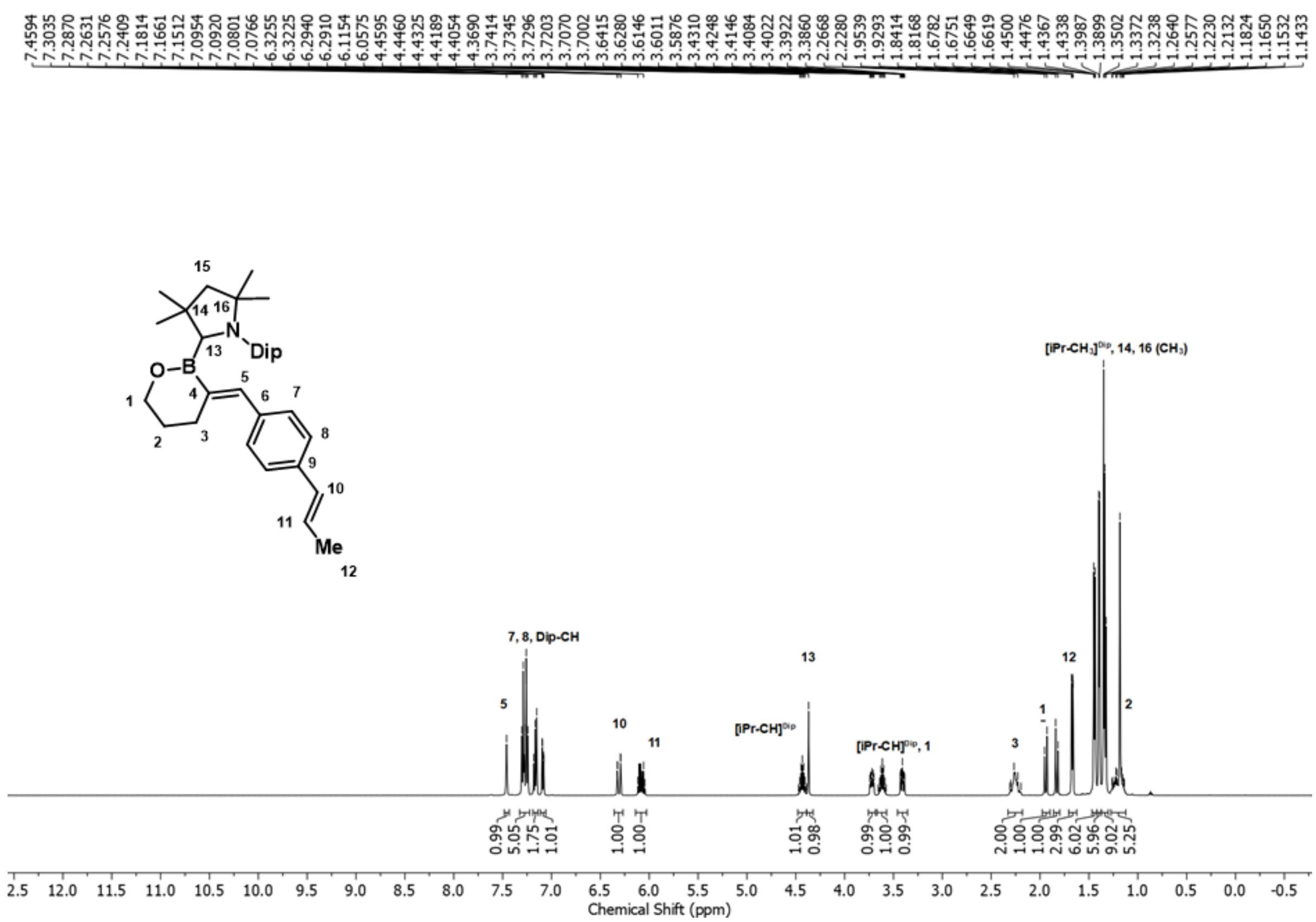

^{13}C NMR (126 MHz, Benzene-d_6) of Compound 14

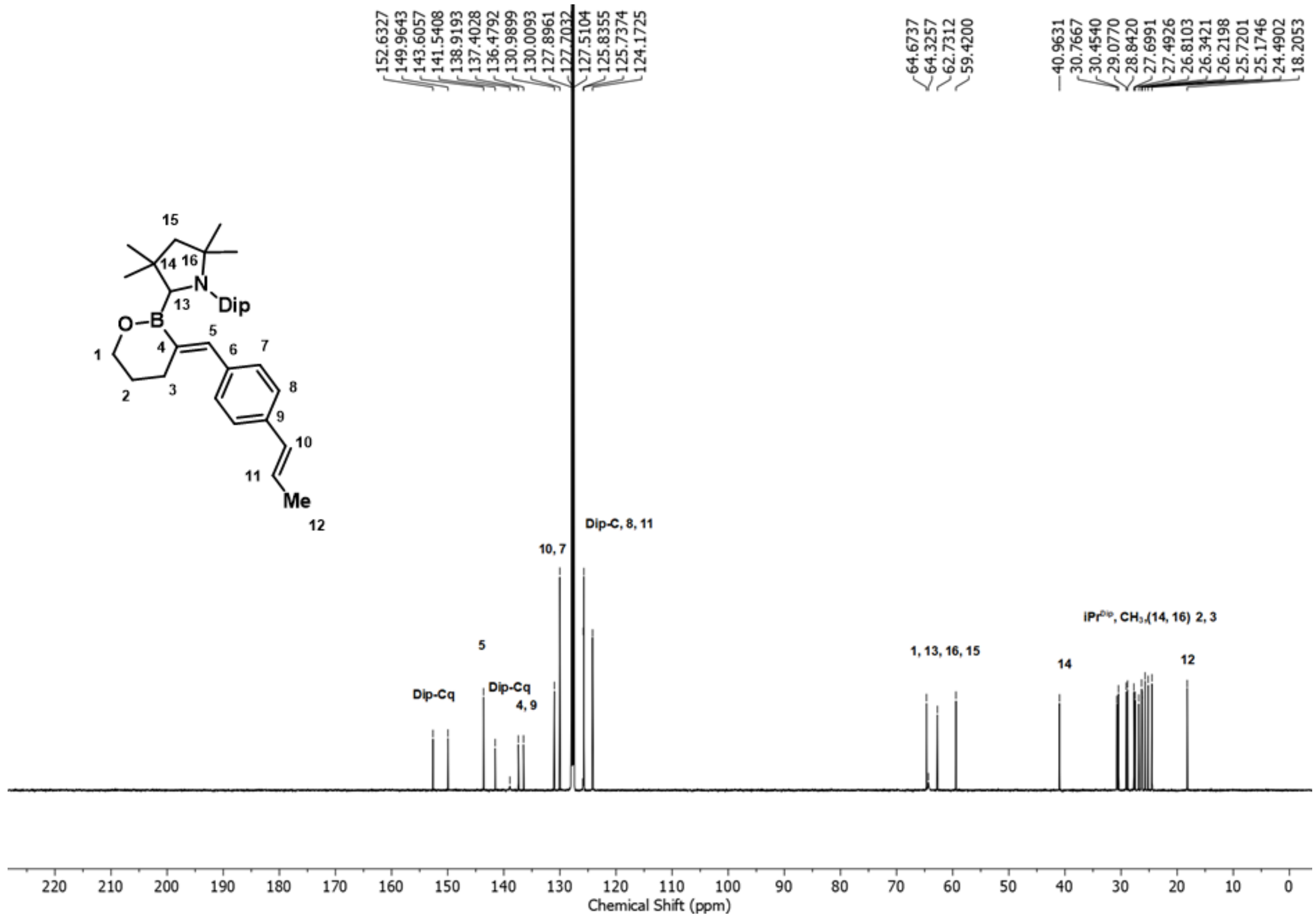

^{11}B NMR (471 MHz, Benzene-d_6) of Compound 14

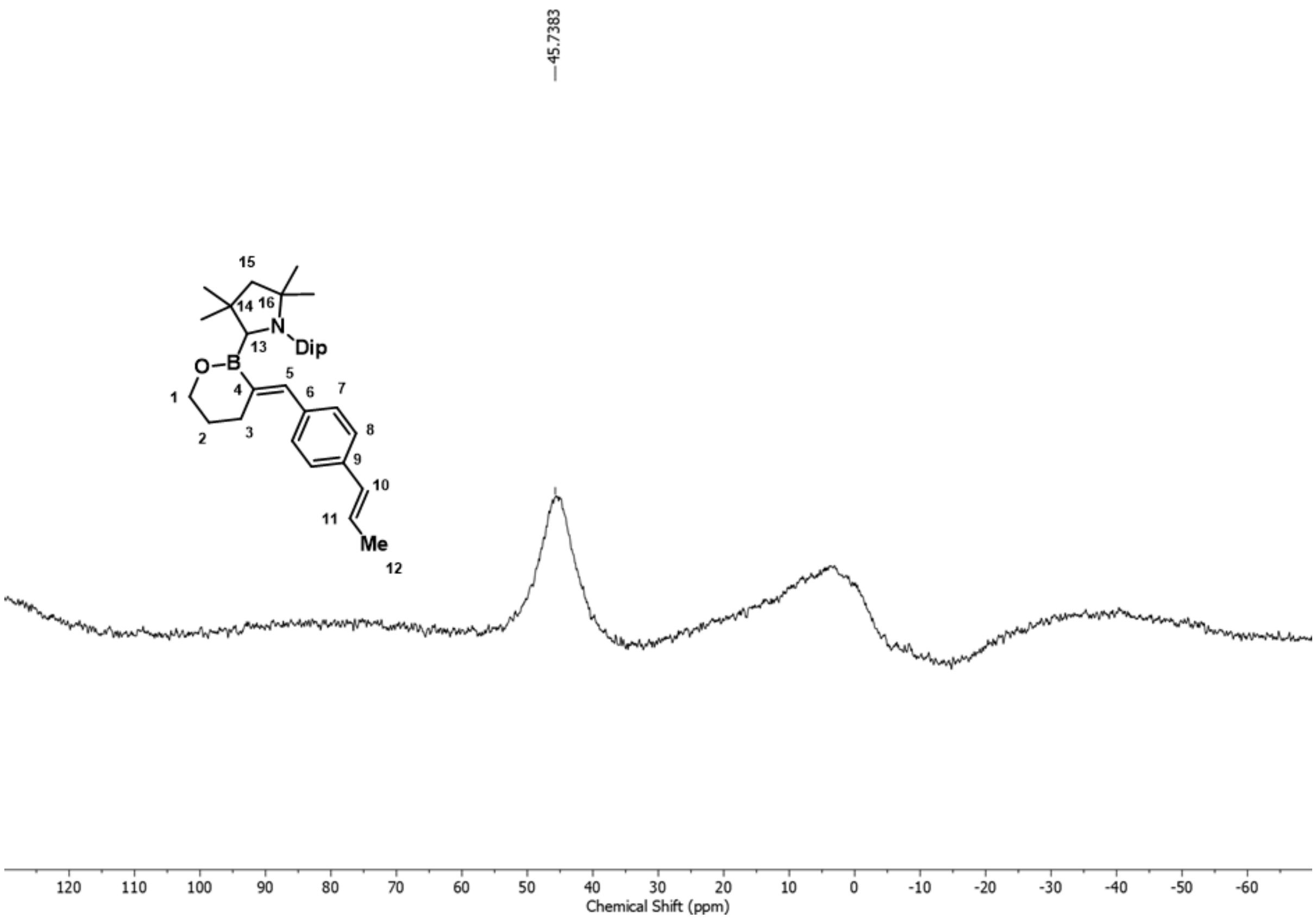

HRMS (ESI) of Compound 14

Elemental Composition Report **Page 1**

Single Mass Analysis
Tolerance = 5.0 PPM / DBE: min = -1.5, max = 500.0
Element prediction: Off
Number of isotope peaks used for i-FIT = 3

Monoisotopic Mass, Even Electron Ions
284 formula(e) evaluated with 1 results within limits (up to 50 closest results for each mass)
Elements Used:
C: 34-34 H: 5-50 B: 0-2 N: 1-5 O: 1-5 Na: 0-1 Sn: 0-1

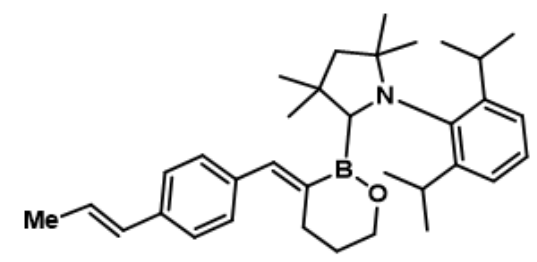

Chemical Formula: $C_{34}H_{48}BNO$
Exact Mass: 497.3829

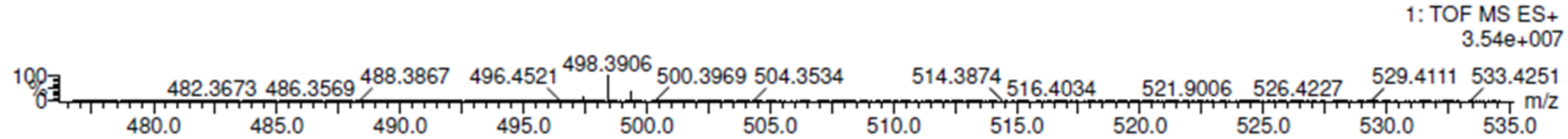

Minimum:				-1.5				
Maximum:		5.0	5.0	500.0				
Mass	Calc. Mass	mDa	PPM	DBE	i-FIT	Norm	Conf(%)	Formula
498.3906	498.3907	-0.1	-0.2	11.5	560.2	n/a	n/a	C34 H49 B N O

^{1}H NMR (500 MHz, Chloroform-*d*) of Compound 15

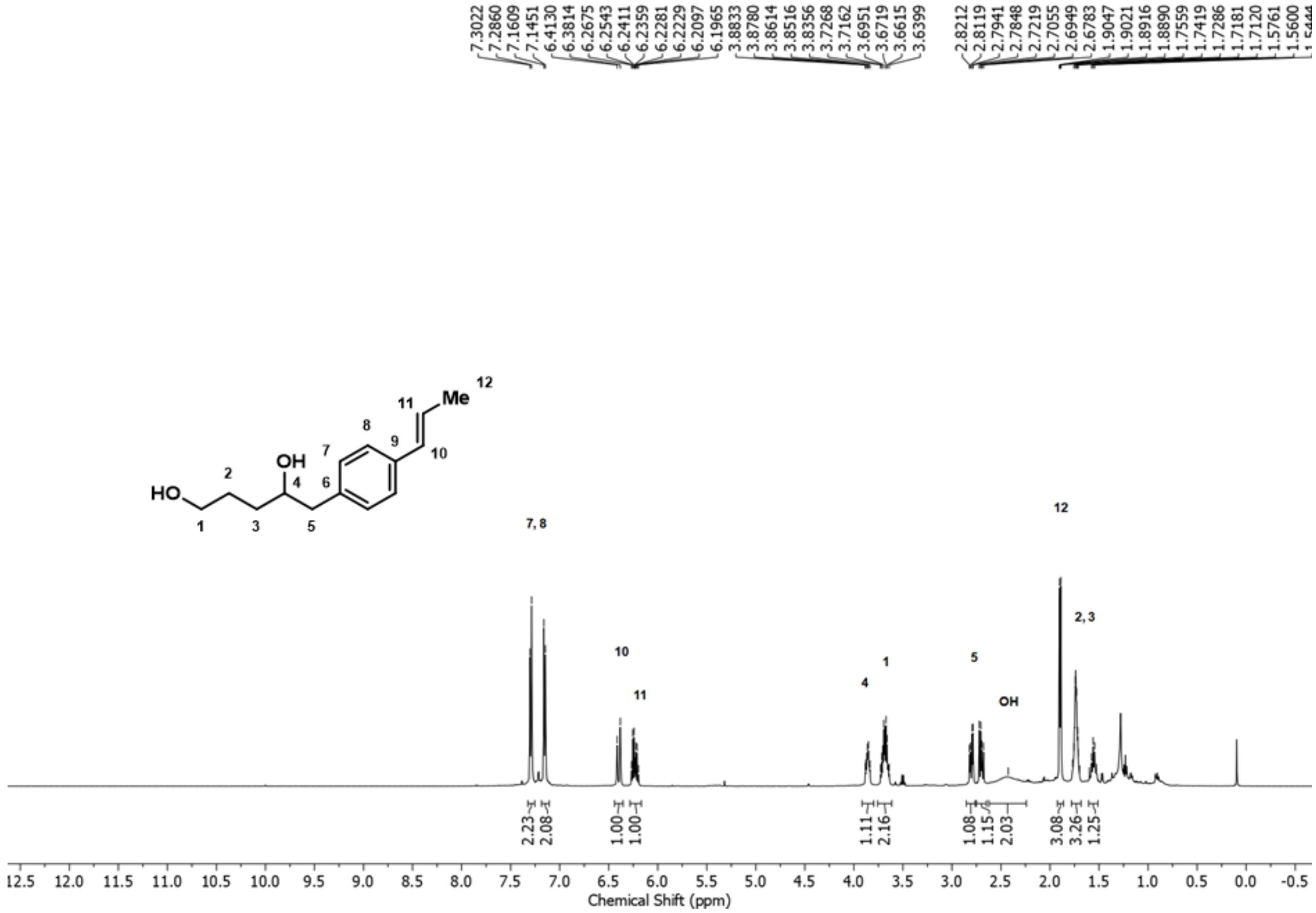

^{13}C NMR (126 MHz, Chloroform-*d*) of Compound 15

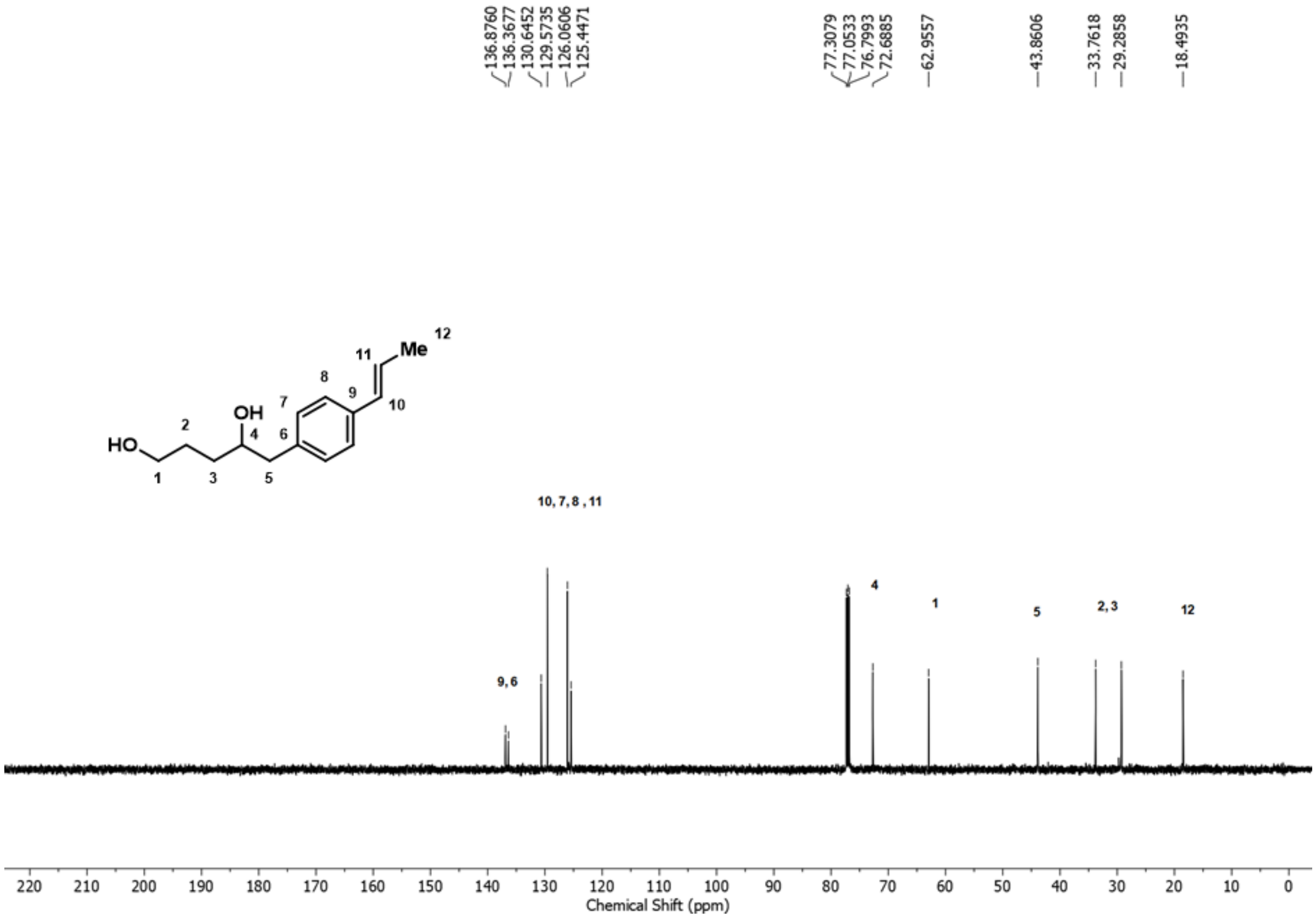

HRMS (ESI) of Compound 15

Elemental Composition Report **Page 1**

Single Mass Analysis
Tolerance = 5.0 PPM / DBE: min = -1.5, max = 500.0
Element prediction: Off
Number of isotope peaks used for i-FIT = 3

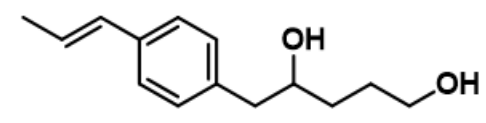

Monoisotopic Mass, Even Electron Ions
64 formula(e) evaluated with 1 results within limits (up to 50 closest results for each mass)
Elements Used:
C: 14-18 H: 5-25 B: 0-2 O: 1-5 Na: 0-1 Sn: 0-1

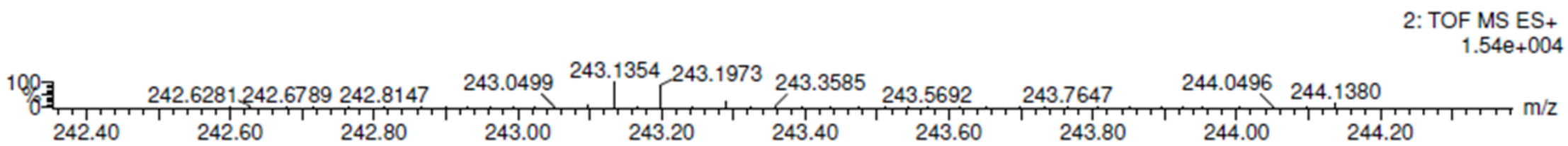

Minimum:				-1.5				
Maximum:		5.0	5.0	500.0				
Mass	Calc. Mass	mDa	PPM	DBE	i-FIT	Norm	Conf(%)	Formula
243.1354	243.1361	-0.7	-2.9	4.5	308.9	n/a	n/a	C14 H20 O2 Na

^{1}H NMR (500 MHz, Chloroform-*d*) of Compound 17

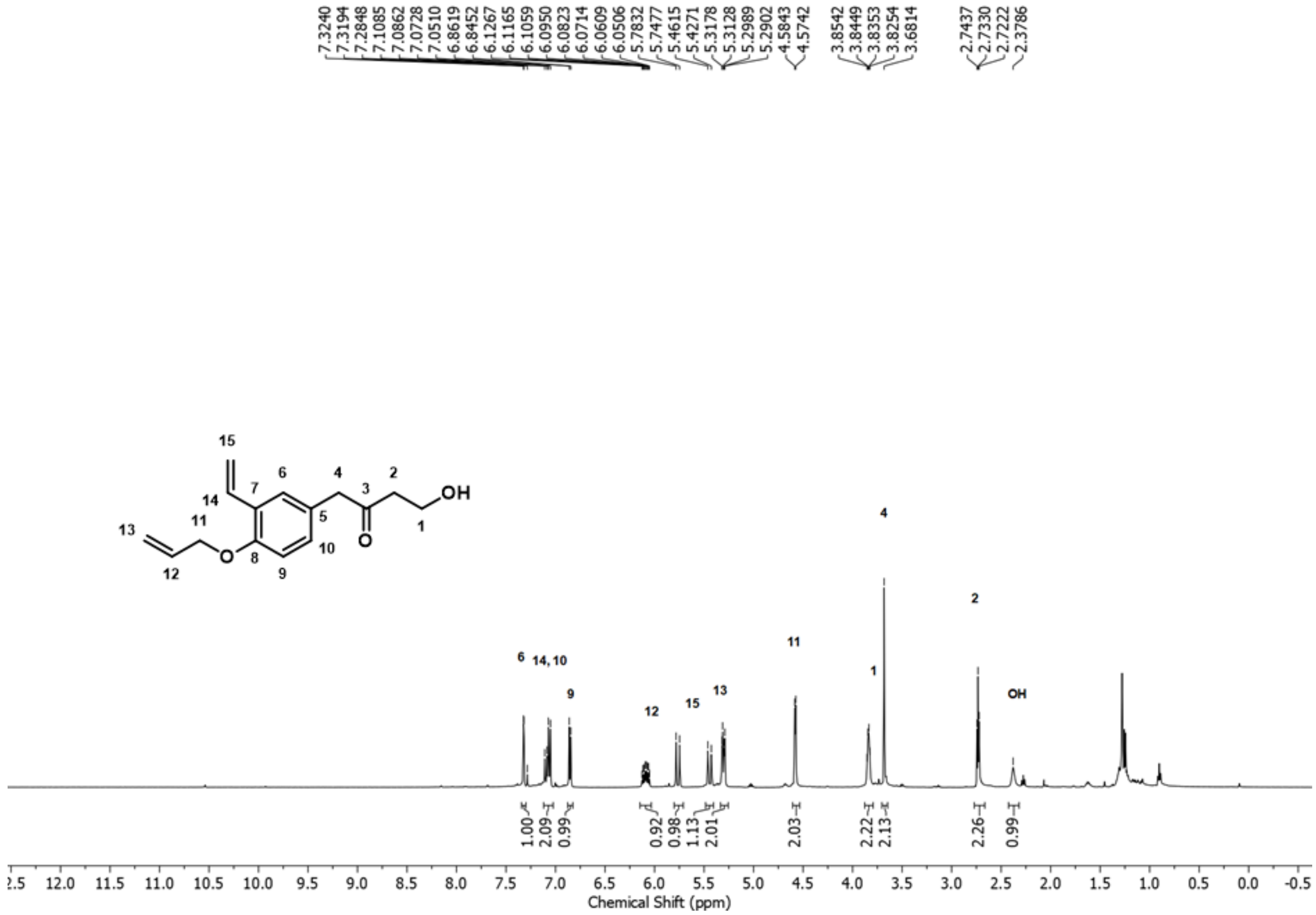

^{13}C NMR (126 MHz, Chloroform-*d*) of Compound 17

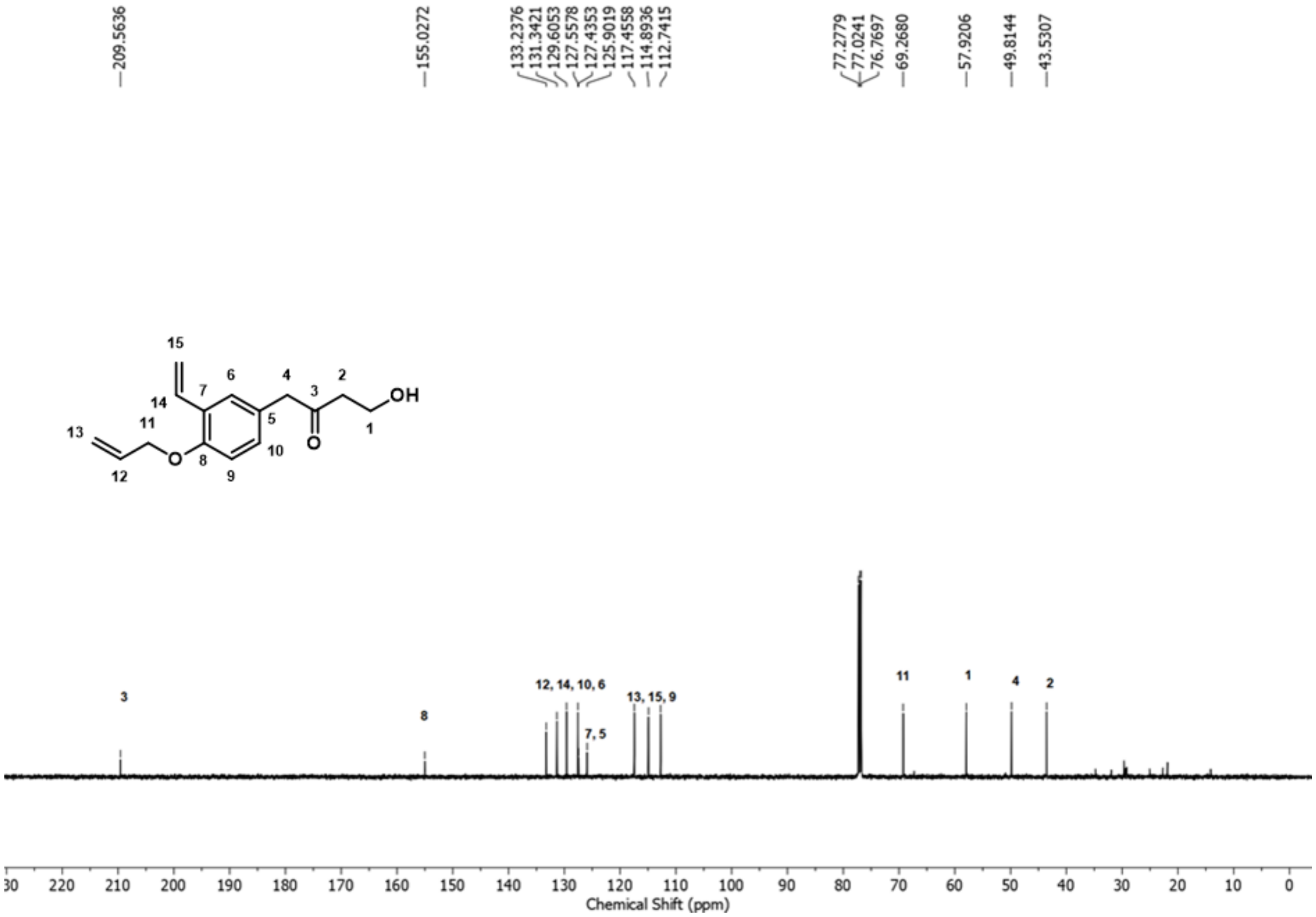

HRMS (ESI) of Compound 17

Elemental Composition Report

Single Mass Analysis
Tolerance = 50.0 PPM / DBE: min = -1.5, max = 500.0
Element prediction: Off
Number of isotope peaks used for i-FIT = 3

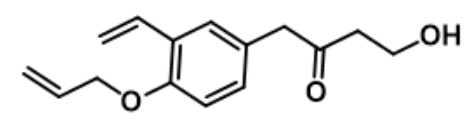

Chemical Formula: $C_{15}H_{18}O_3$
Exact Mass: 246.1256

Monoisotopic Mass, Even Electron Ions
17 formula(e) evaluated with 1 results within limits (up to 50 closest results for each mass)
Elements Used:
C: 15-15 H: 1-20 O: 1-5 Na: 0-1 Sn: 0-1

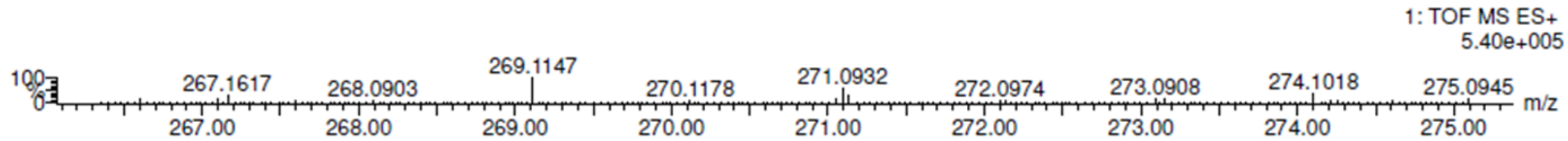

	Mass	Calc. Mass	mDa	PPM	DBE	i-FIT	Norm	Conf(%)	Formula
Minimum:					-1.5				
Maximum:			5.0	50.0	500.0				
	269.1147	269.1154	-0.7	-2.6	6.5	881.2	n/a	n/a	C15 H18 O3 Na

^{1}H NMR (500 MHz, Chloroform-*d*) of Compound 19

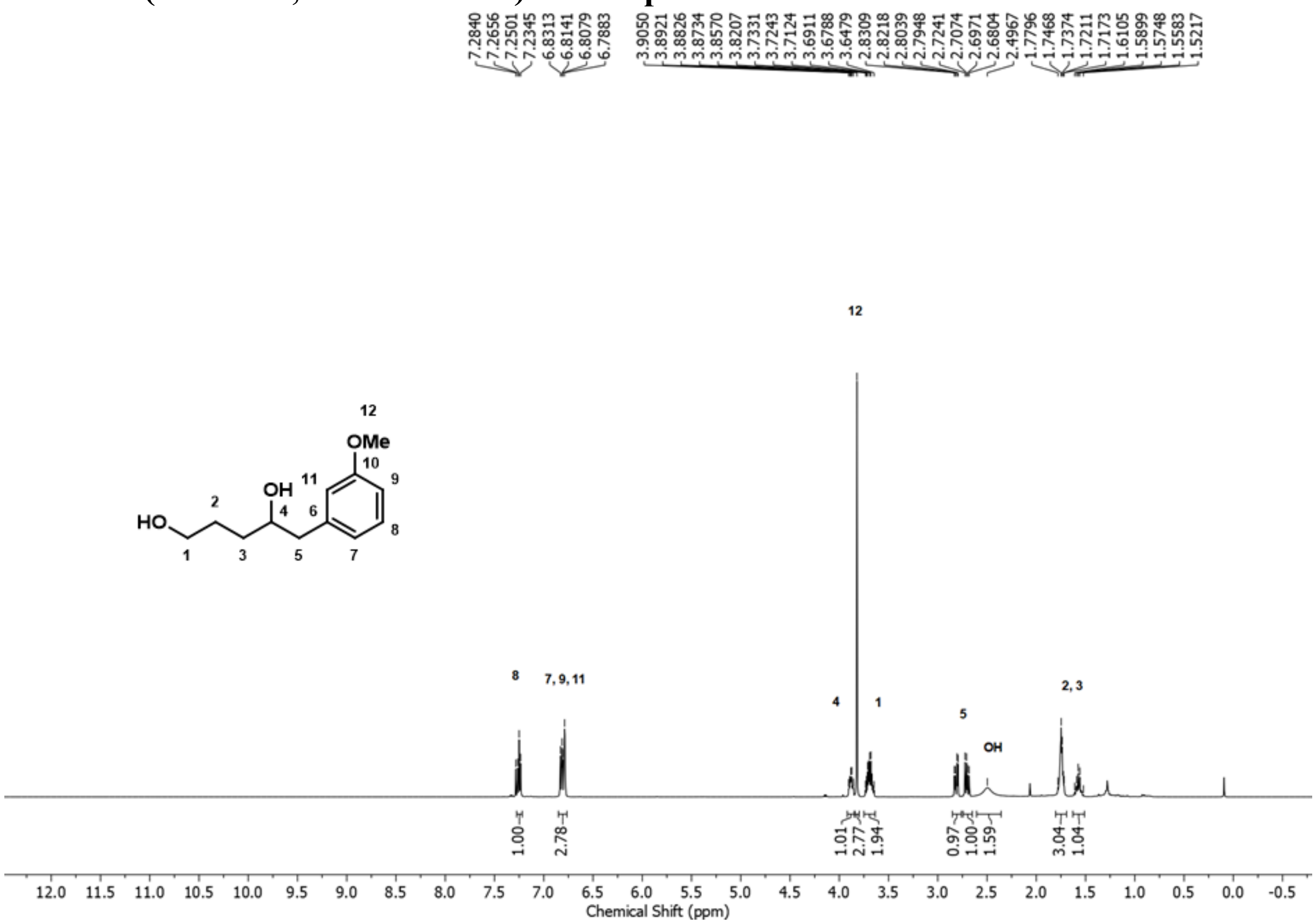

^{13}C NMR (126 MHz, Chloroform-*d*) of Compound 19

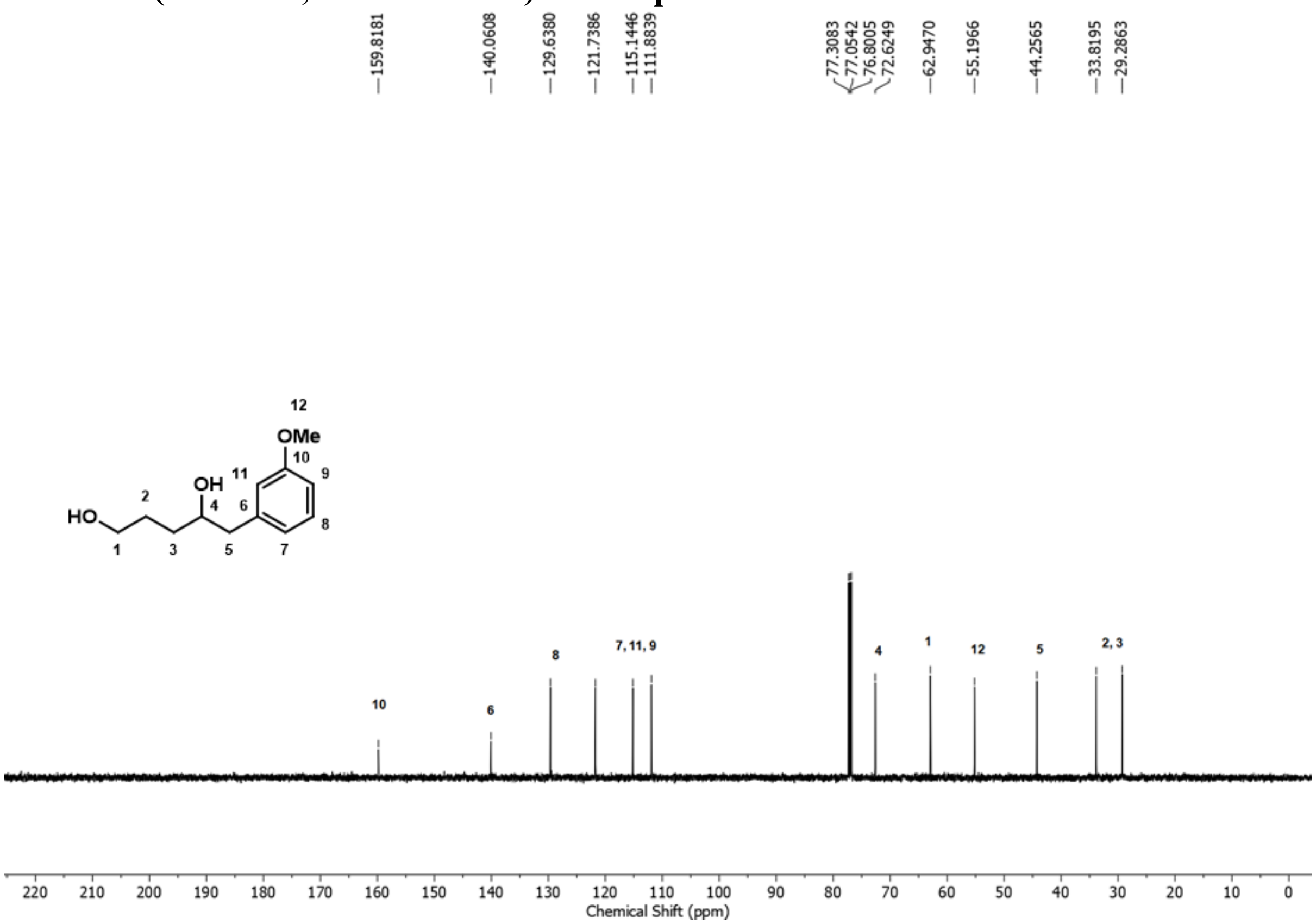

^{1}H NMR (400 MHz, Chloroform-*d*) of keto alcohol 20a

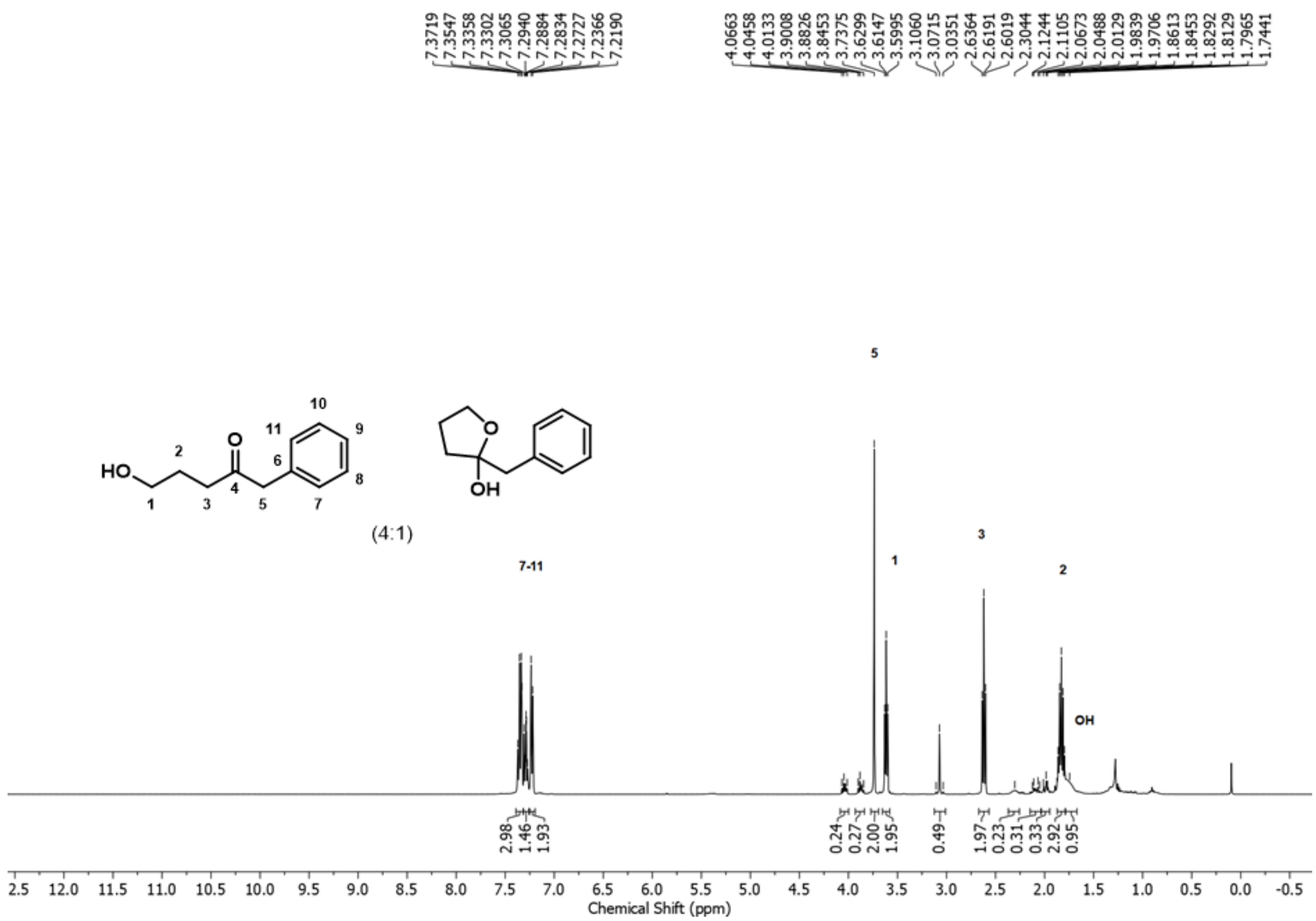

^{13}C NMR (101 MHz, Chloroform-*d*) of keto alcohol 20a**

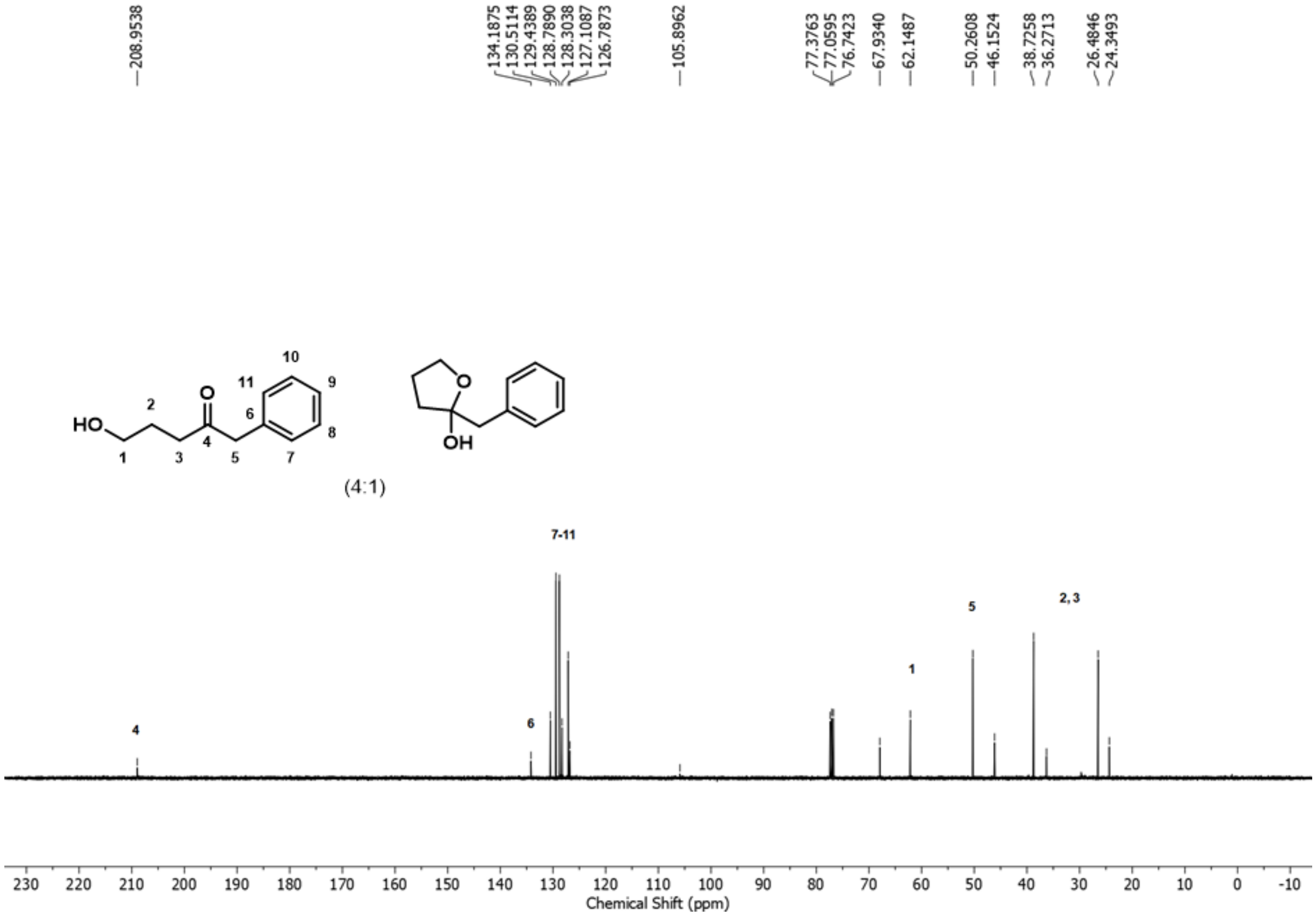

^{1}H NMR (500 MHz, Chloroform-*d*) of compound 21

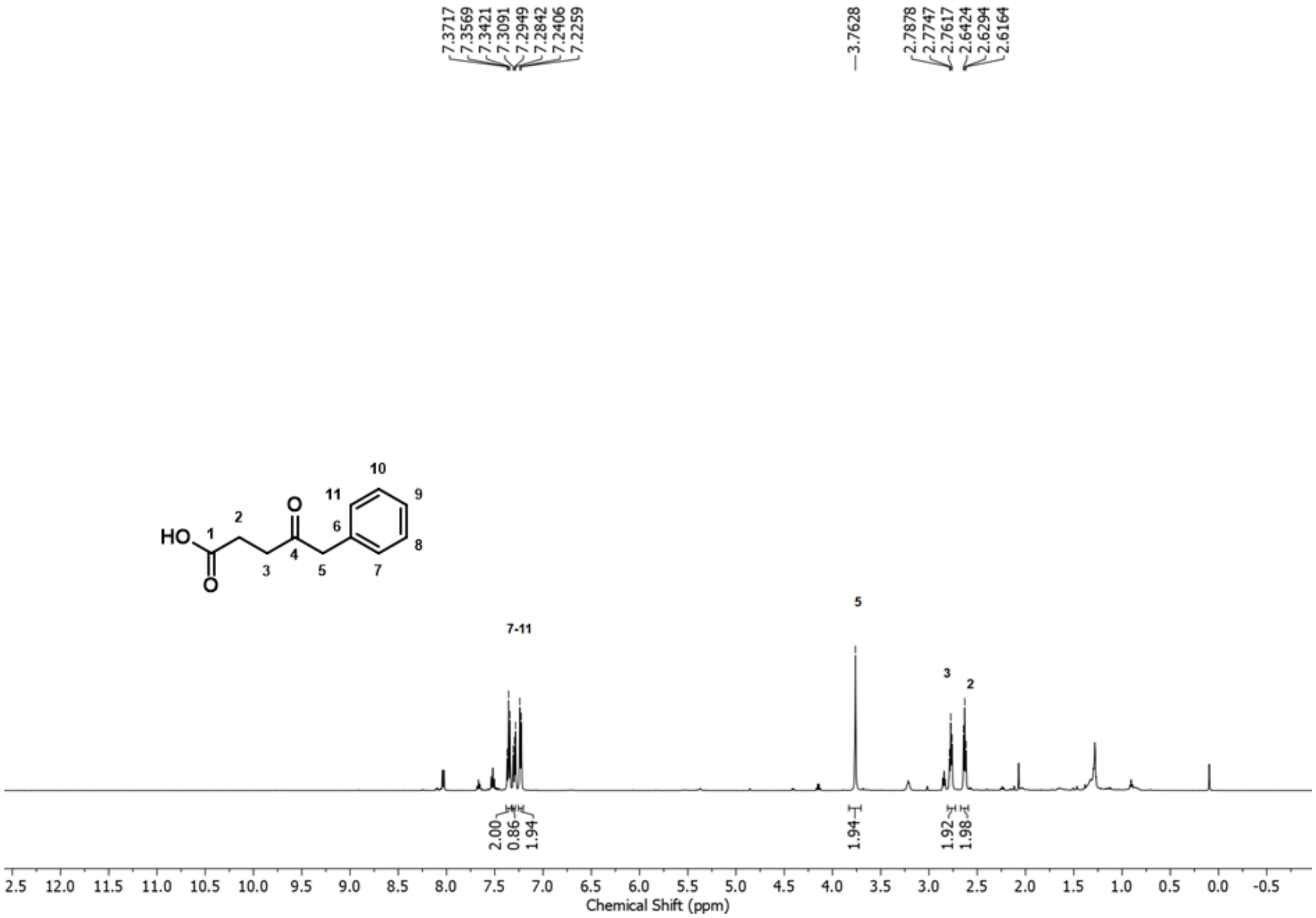

^{13}C NMR (126 MHz, Chloroform-*d*) of compound 21

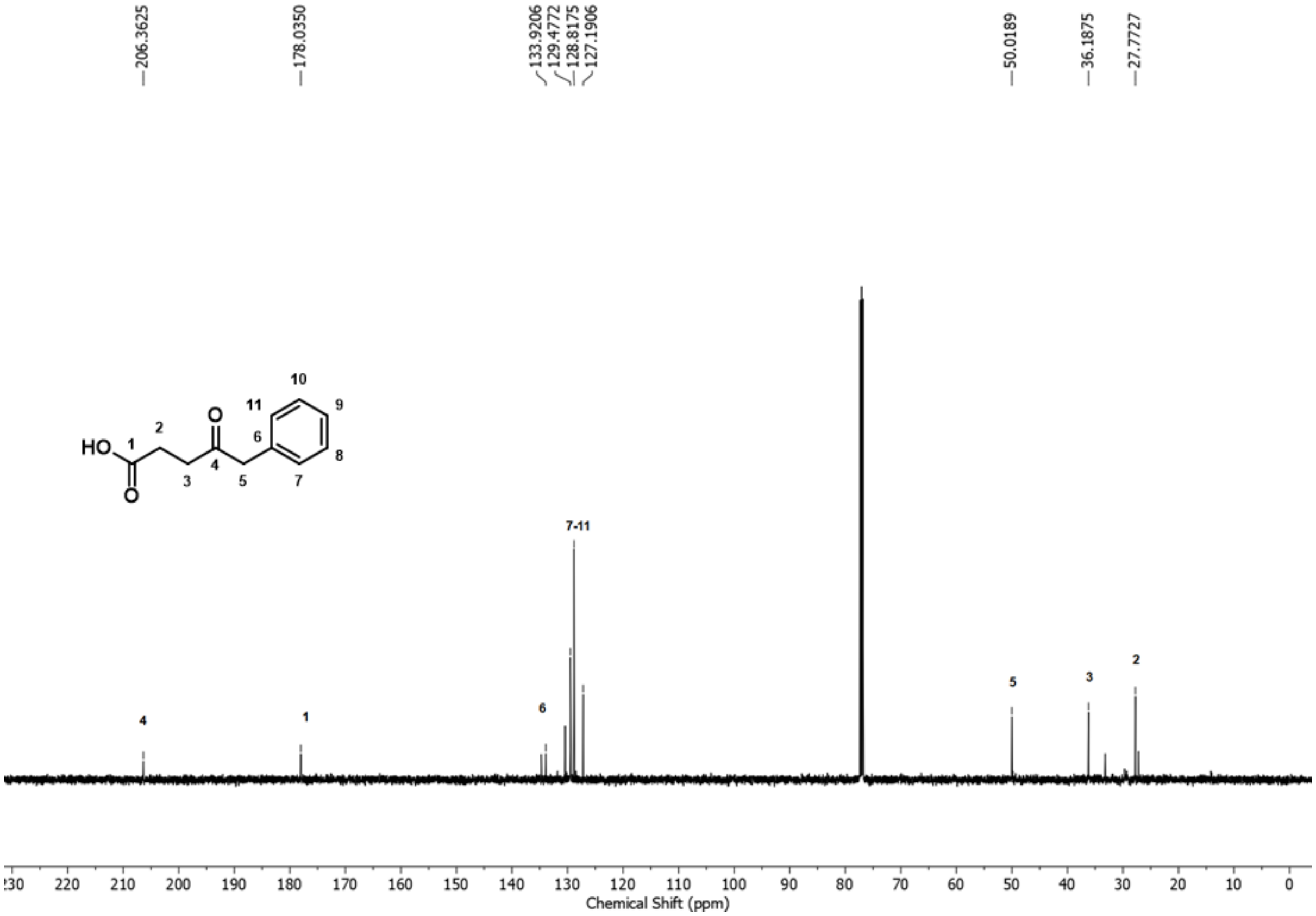

6. References

1. Jana, S.; He, F.; Koenigs, R. M. Gold-Catalyzed Carbazolation Reactions of Alkynes. *Org. Lett.* **2020**, *22*, 4873-4877.
2. Polgar, A. M.; Zhang, A.; Mack, F.; Weigend, F.; Lebedkin, S.; Stillman, M. J.; Corrigan, J. F. Tuning the Metal/Chalcogen Composition in Copper(I)–Chalcogenide Clusters with Cyclic (Alkyl)(amino)carbene Ligands. *Inorg. Chem.* **2019**, *58*, 3338-3348.
3. Lavallo, V.; Canac, Y.; Präsang, C.; Donnadieu, B.; Bertrand, G. Stable Cyclic (Alkyl)(Amino)Carbenes as Rigid or Flexible, Bulky, Electron-Rich Ligands for Transition-Metal Catalysts: A Quaternary Carbon Atom Makes the Difference. *Angew. Chem. Int. Ed.* **2005**, *44*, 5705-5709.
4. Xiao, Y.; Liu, Z.; Liang, J.; Yang, K.; Huang, W. Trivalent Rare-Earth Metal Cyclic (Alkyl)(Amino)Carbene Complexes. *Dalton Trans*. **2022**, *51*, 15873-15882.
5. Lu, X.-Y.; Li, J.-S.; Wang, J.-Y.; Wang, S.-Q.; Li, Y.-M.; Zhu, Y.-J.; Zhou, R.; Ma, W.-J. Cu-Catalyzed Cross-Coupling Reactions of Vinyl Epoxide with Organoboron Compounds: Access to Homoallylic Alcohols. *RSC Adv.* **2018**, *8*, 41561-41565.
6. Zeng, X.; Miao, C.; Wang, S.; Xia, C.; Sun, W. Asymmetric 5-endo Chloroetherification of Homoallylic Alcohols Toward the Synthesis of Chiral β-Chlorotetrahydrofurans. *Chem. Commun.* **2013**, *49*, 2418-2420.
7. Lin, R.; Sun, H.; Yang, C.; Xia, W. Visible-Light-Induced Intramolecular Chloroetherification of Electron-Enriched Styrenes. Asian *J. Org. Chem.* **2017**, *6*, 418-421.
8. Wang, Y.; Jiang, M.; Liu, J.-T. Copper-Catalyzed Diastereoselective Synthesis of Trifluoromethylated Tetrahydrofurans. *Adv. Synth. Catal.* **2016**, *358*, 1322-1327.
9. Cornella, J.; Martin, R. Ni-Catalyzed Stereoselective Arylation of Inert C–O bonds at Low Temperatures. *Org. Lett.* **2013**, *15*, 6298-6301.
10. Fanourakis, A.; Hodson, N. J.; Lit, A. R.; Phipps, R. J. Substrate-Directed Enantioselective Aziridination of Alkenyl Alcohols Controlled by a Chiral Cation. *J. Am. Chem. Soc.* **2023**, *145*, 7516-7527.
11. He, C.; Wang, Y.; Li, Q.-X.; Yan, Z.; Zhang, K.; Ni, S.-F.; Duan, X.-H.; Liu, L. Alkylarylation of Alkenes with Arylsulfonylacetate as Bifunctional Reagent via Photoredox Radical Addition/Smiles Rearrangement Cascade. *Chinese Chemical Letters* **2025**, *36*, 110253.

12. Colomer, I.; Batchelor-McAuley, C.; Odell, B.; Donohoe, T. J.; Compton, R. G. Hydrogen Bonding to Hexafluoroisopropanol Controls the Oxidative Strength of Hypervalent Iodine Reagents. *J. Am. Chem. Soc.* **2016**, *138*, 8855-8861.
13. Tan, E. H. P.; Lloyd-Jones, G. C.; Harvey, J. N.; Lennox, A. J. J.; Mills, B. M. [$(RCN)_2PdCl_2$]-Catalyzed E/Z Isomerization of Alkenes: A Non-Hydride Binuclear Addition–Elimination Pathway. *Angew. Chem. Int. Ed.* **2011**, *50*, 9602-9606.
14. Dong, M.; Jia, S.; Chen, X.; Jiao, J.; Xue, C.; Xia, Z.; Cheng, H.; Deng, T.; Chen, C.; Dong, K.; Wu, H.; He, M.; Han, B. Cathode-Induced C–H Bond Heterolysis for Olefin Isomerization and Applications in Electrocarboxylation. *J. Am. Chem. Soc.* **2025**, *147*, 19976-19985.
15. Migglautsch, A. K.; Willim, M.; Schweda, B.; Glieder, A.; Breinbauer, R.; Winkler, M. Aliphatic Hydroxylation and Epoxidation of Capsaicin by Cytochrome P450 CYP505X. *Tetrahedron* **2018**, *74*, 6199-6204.
16. Li, M.-B.; Li, H.; Wang, J.; Liu, C.-R.; Tian, S.-K. Catalytic Stereospecific Alkylation of Malononitriles with Enantioenriched Primary Allylic Amines. *Chem. Commun.* **2013**, *49*, 8190-8192.
17. Bai, N.; Wang, X.; Wang, Z.; Liu, F.; Rong, Z.-Q. Redox-Neutral Remote Amidation of Alkenyl Alcohols via Long-Range Isomerization/Transformation. *Org. Chem. Front.* **2022**, *9*, 5942-5948.
18. Schmidt, B.; Krehl, S.; Sotelo-Meza, V. Synthesis of Chromanes through RCM-Transfer Hydrogenation. *Synthesis* **2012**, *44*, 1603–1613.
19. Fanourakis, A.; Hodson, N. J.; Lit, A. R.; Phipps, R. J. Substrate-Directed Enantioselective Aziridination of Alkenyl Alcohols Controlled by a Chiral Cation. *J. Am. Chem. Soc.* **2023**, *145*, 7516–7527.
20. Hofmann, A.; Légaré, M.-A.; Wüst, L.; Braunschweig, H. Heterodiatomic Multiple Bonding in Group 13: A Complex with a Boron–Aluminum π Bond Reduces CO_2. *Angew. Chem. Int. Ed.* **2019**, *58*, 9776-9781.
21. Meng, F.; Jang, H.; Hoveyda, A. H. Exceptionally E- and β-Selective NHC-Cu-Catalyzed Proto-Silyl Additions to Terminal Alkynes and Site- and Enantioselective Proto-Boryl Additions to the Resulting Vinylsilanes: Synthesis of Enantiomerically Enriched Vicinal and Geminal Borosilanes. *Chem. Eur. J.* **2013**, *19*, 3204–3214.

22. Zong, Y.; Zou, X.; Tao, H.; Huang, Q.; Chen, G.-Q.; Zhang, X. Highly Efficient and Enantioselective Iridium-Catalyzed Asymmetric Reductive Cycloetherification. *J. Am. Chem. Soc.* **2024**, *146*, 34107–34117.
23. Ketterer, C.; Wünsch, B. Lipase-catalyzed Kinetic Resolution of 2-phenylethanol Derivatives and Chiral oxa-Pictet–Spengler Reaction as the Key Steps in the Synthesis of Enantiomerically Pure Tricyclic Amines. *European J. Org. Chem.* **2012**, *2012*, 2428–2444.
24. Sheldrick, G. M. SHELXT – Integrated Space-Group and Crystal-Structure Determination. *Acta Crystallogr., Sect. A* **2015**, *71*, 3–8.
25. Sheldrick, G. M. Crystal Structure Refinement with SHELXL. *Acta Crystallogr., Sect. C* **2015**, *71*, 3–8.
26. Dolomanov, O. V.; Bourhis, L. J.; Gildea, R. J.; Howard, J. A. K.; Puschmann, H. OLEX2: A Complete Structure Solution, Refinement and Analysis Program. *J. Appl. Crystallogr.* **2009**, *42*, 339–341.
27. Neese, F. Software Update: The ORCA Program System, Version 6.0. *WIREs Computational Molecular Science* **2025**, *15*, e70019.
28. Lin, Y.-S.; Li, G.-D.; Mao, S.-P.; Chai, J.-D. Long Range Corrected Hybrid Density Functionals with Improved Dispersion Corrections. *Journal of Chemical Theory and Computation* **2013**, *9*, 263–272.
29. Grimme, S.; Antony, J.; Ehrlich, S.; Krieg, H. A Consistent and Accurate ab Initio Parametrization of Density Functional Dispersion Correction DFT-D for the 94 Elements H–Pu. *Journal of Chemical Physics* **2010**, *132*, 154104.
30. Dunning, T. H., Jr. Gaussian Basis Sets for Use in Correlated Molecular Calculations. I. The Atoms Boron through Neon and Hydrogen. *Journal of Chemical Physics* **1989**, *90*, 1007–1023.
31. Barone, V.; Cossi, M. Quantum Calculation of Molecular Energies and Energy Gradients in Solution by a Conductor Solvent Model. *Journal of Physical Chemistry A* **1998**, *102*, 1995–2001.
32. Henkelman, G.; Uberuaga, B. P.; Jónsson, H. A Climbing Image Nudged Elastic Band Method for Finding Saddle Points and Minimum Energy Paths. *Journal of Chemical Physics* **2000**, *113*, 9901–9904.

33. Ásgeirsson, V.; Birgisson, B. O.; Bjornsson, R.; Becker, U.; Neese, F.; Riplinger, C.; Jónsson, H. Nudged Elastic Band Method for Molecular Reactions Using Energy Weighted Springs Combined with Eigenvector Following. *Journal of Chemical Theory and Computation* **2021**, *17*, 4929–4945.

34. Weigend, F.; Ahlrichs, R. Balanced Basis Sets of Split Valence, Triple Zeta Valence and Quadruple Zeta Valence Quality for H to Rn: Design and Assessment of Accuracy. *Physical Chemistry Chemical Physics* **2005**, *7*, 3297–3305.

35. Fukui, K. The Path of Chemical Reactions: The IRC Approach. *Accounts of Chemical Research* **1981**, *14*, 363–368.

36. Helgaker, T.; Klopper, W.; Koch, H.; Noga, J. Basis Set Convergence of Correlated Calculations on Water. *Journal of Chemical Physics* **1997**, *106*, 9639–9646.

37. Grimme, S. Supramolecular Binding Thermodynamics by Dispersion Corrected Density Functional Theory. *Chemistry A European Journal* **2012**, *18*, 9955–9964.

38. Camaioni, D. M.; Schwerdtfeger, C. A. Comment on “Accurate Experimental Values for the Free Energies of Hydration of H^+, OH^-, and H_3O^+”. *Journal of Physical Chemistry A* **2005**, *109*, 10795–10797.
